# Supplementary material for: Cell Senescence-Independent Changes of Human Skin Fibroblasts with Age
Source: Cells. 2024 Apr 9;13(8):659. doi: 10.3390/cells13080659 (PMC11048776; doi:10.3390/cells13080659)
Supplement: Supplementary file 1 [file cells-13-00659-s001.zip › cells-2907289-supplementary figures and table.pdf]

Supplementary Figures

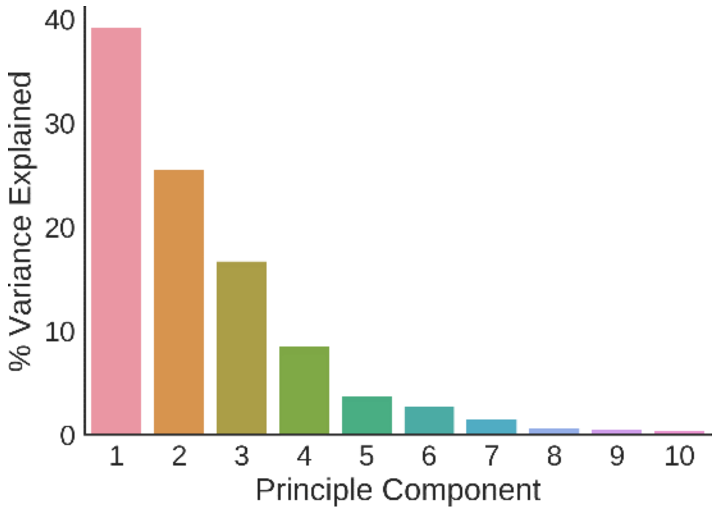

Figure S1 | Plot showing the percent of variance explained for the first 10 principal components.

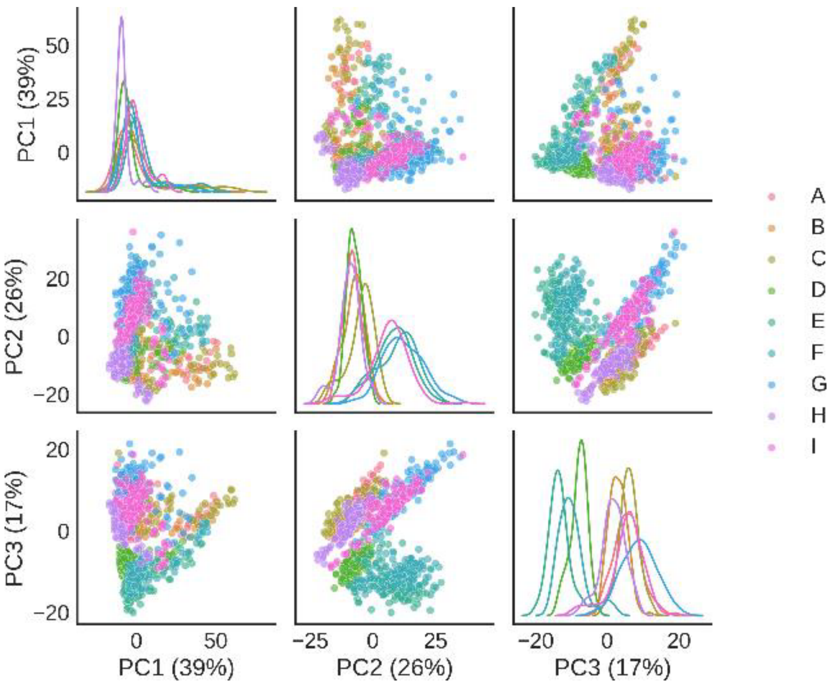

Figure S2 |Scatter matrix of first three principle components coloured by cell id.

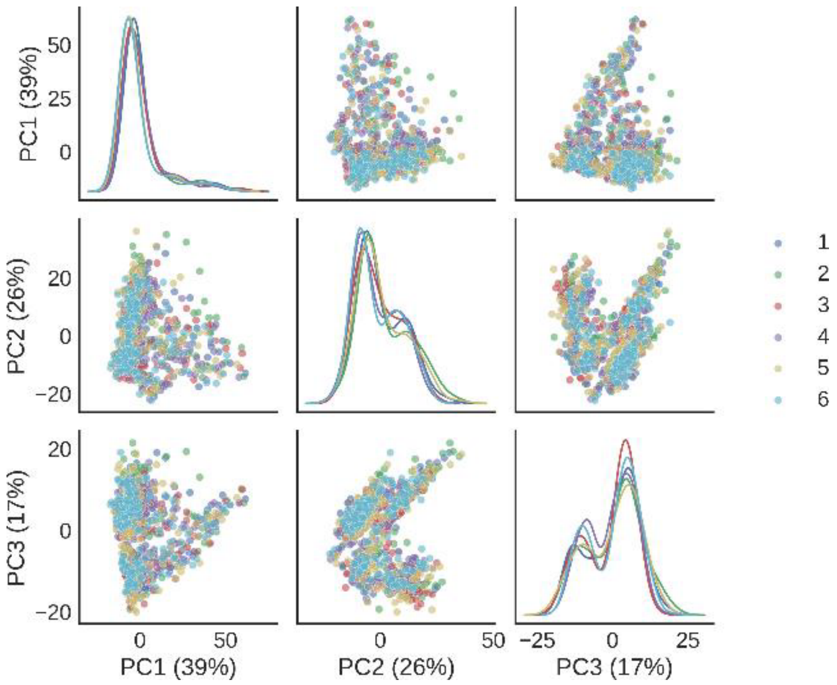

Figure S3 | Scatter matrix of first three principle components coloured by replicate.

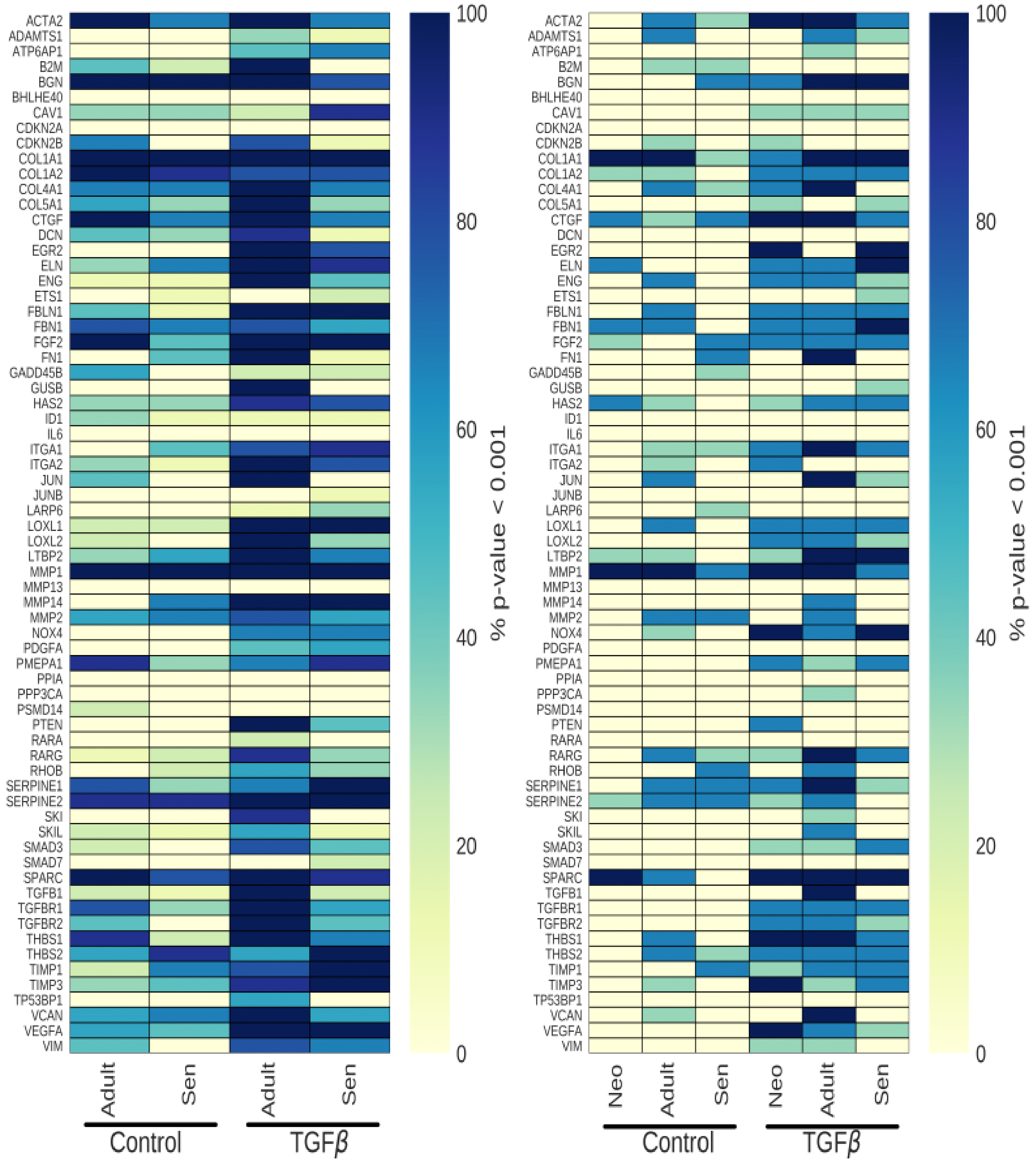

Figure S4 | Differential expression analysis for (a) between groups comparisons and (b) within groups comparisons. Colours indicate the percentage of the time of all combinations (9 for between groups and 3 for within groups) of LIMMA analysis conducted that a time series was differentially expressed with a FDR corrected p-value<0.001.

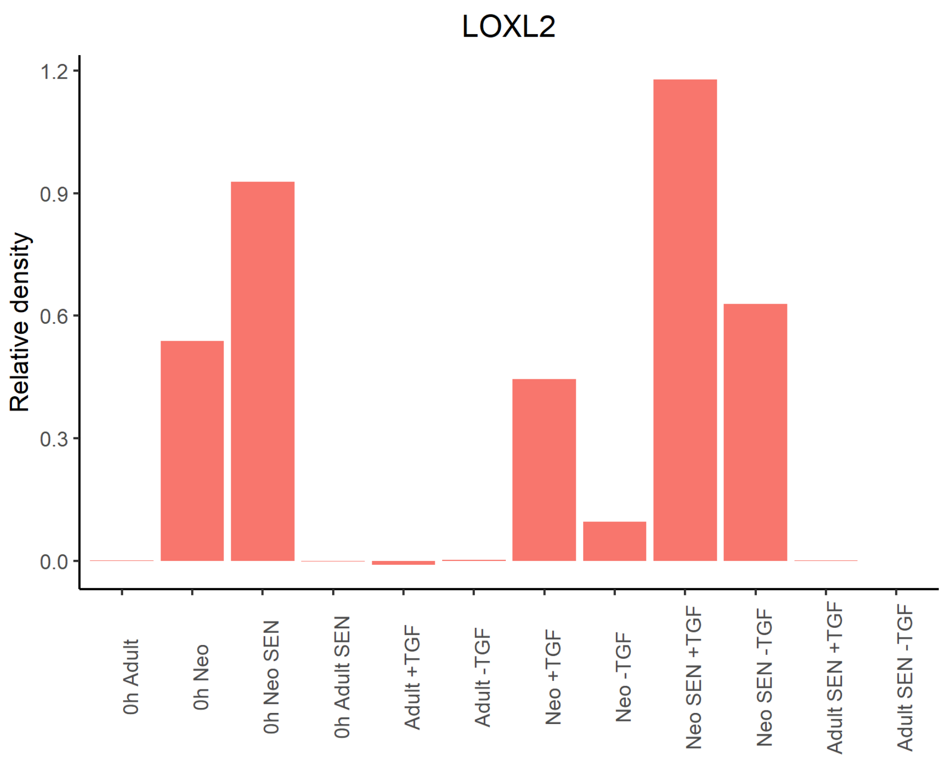

Figure S5 | Densitometry for αSMA and TAGLN for blots found in Figure 2.

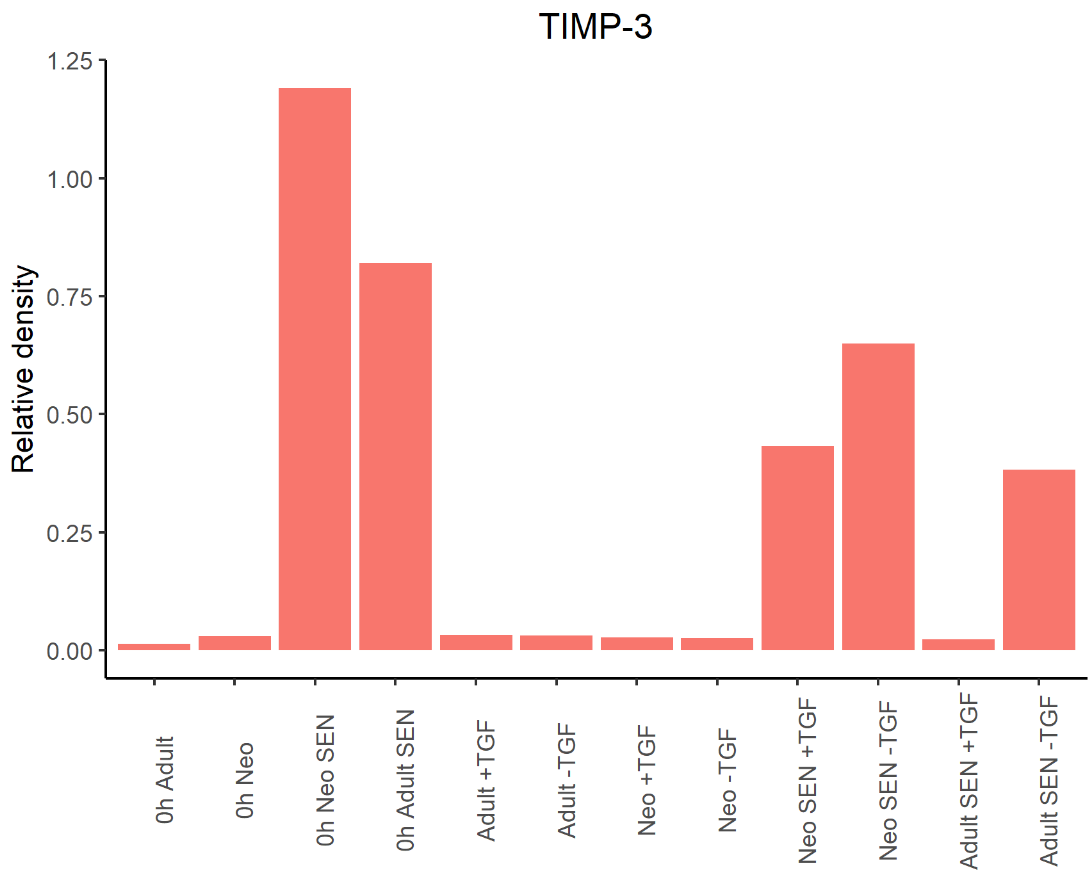

Figure S6 | Densitometry for TIMP3 for blots found in Figure 3.

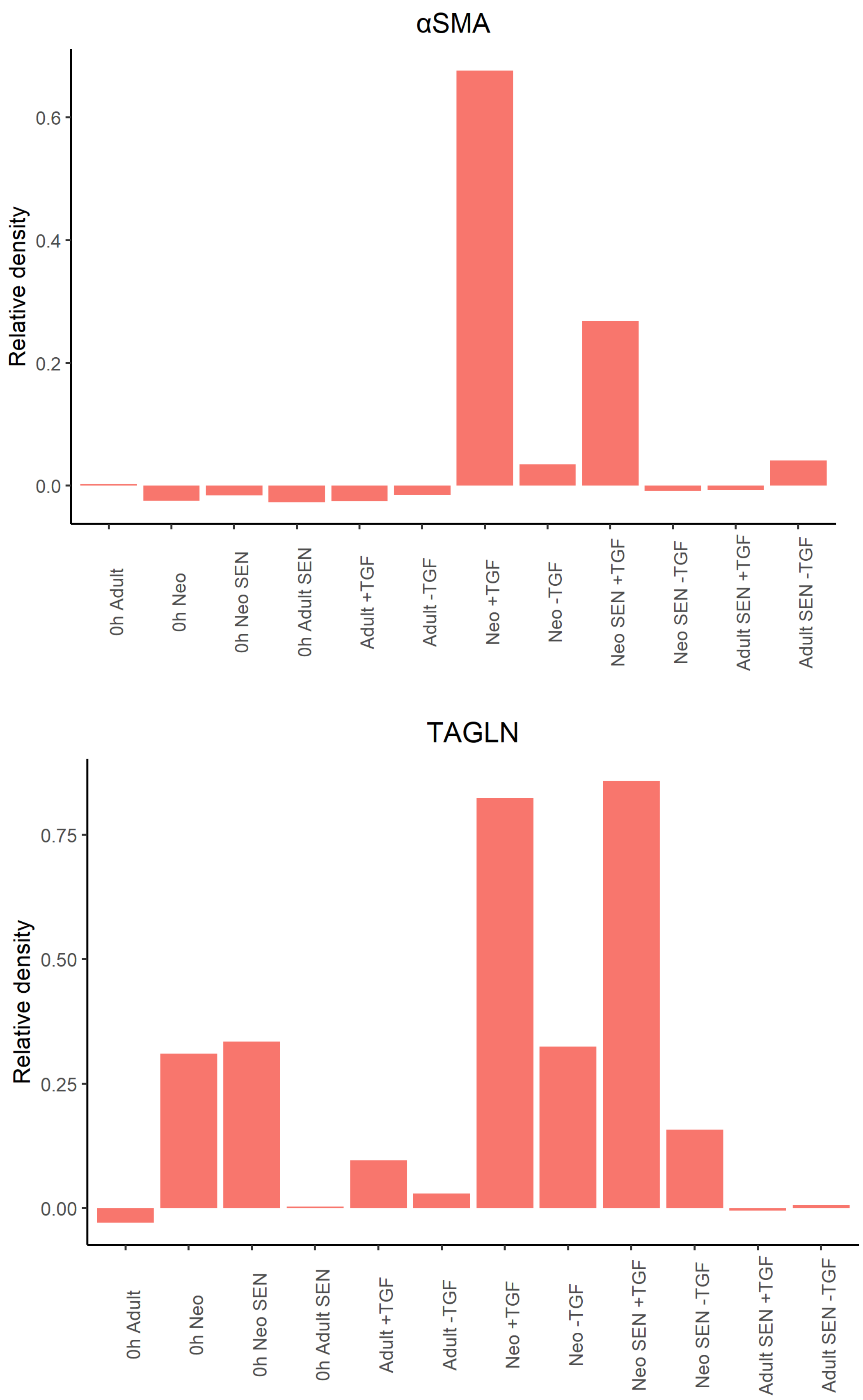

Figure S7 | Densitometry for αSMA and TAGLN for blots found in Figure 7.

Comparison	Control	TGF-β
Adult Vs neonatal	16	46
Senescent Vs neonatal	16	30
Neonatal Vs neonatal	7	28
Adult Vs adult	17	37
Senescent Vs senescent	10	25

Table S1 | Table summarising the number of genes per comparison that were differentially expressed with a (FDR corrected) p-value <0.001 in over 60% of LIMMA analyses per comparison.
