# Supplementary material for: Cell Senescence-Independent Changes of Human Skin Fibroblasts with Age
Source: Cells. 2024 Apr 9;13(8):659. doi: 10.3390/cells13080659 (PMC11048776; doi:10.3390/cells13080659)
Supplement: Supplementary file 1 [file cells-13-00659-s001.zip › Supplementary file 2.pdf]

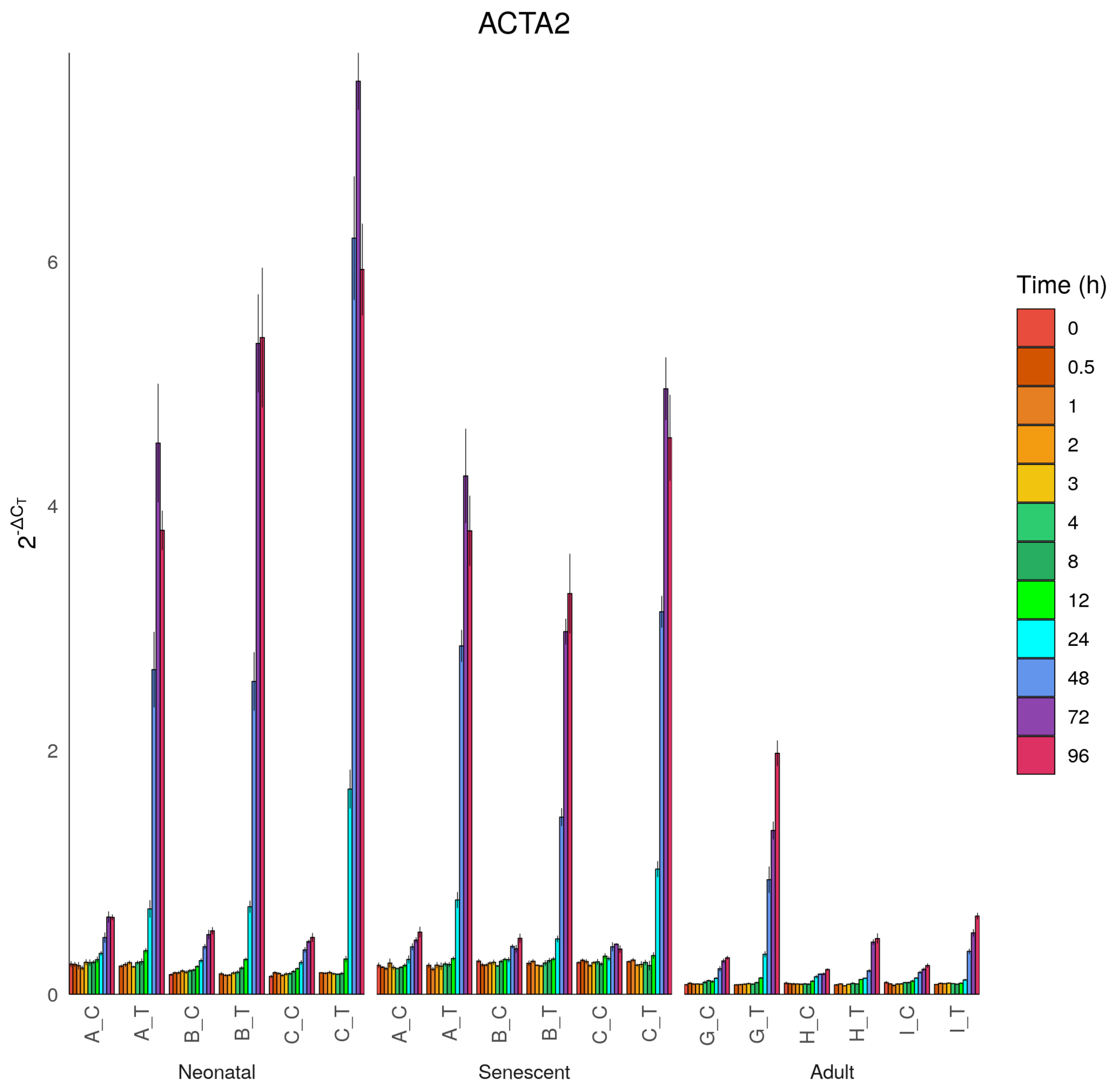
ACTA2
2^-ΔCT
0
2
4
6
A_C
A_T
B_C
B_T
C_C
C_T
G_C
G_T
H_C
H_T
I_C
I_T
Neonatal
Senescent
Adult
Time (h)
0
0.5
1
2
3
4
8
12
24
48
72
96

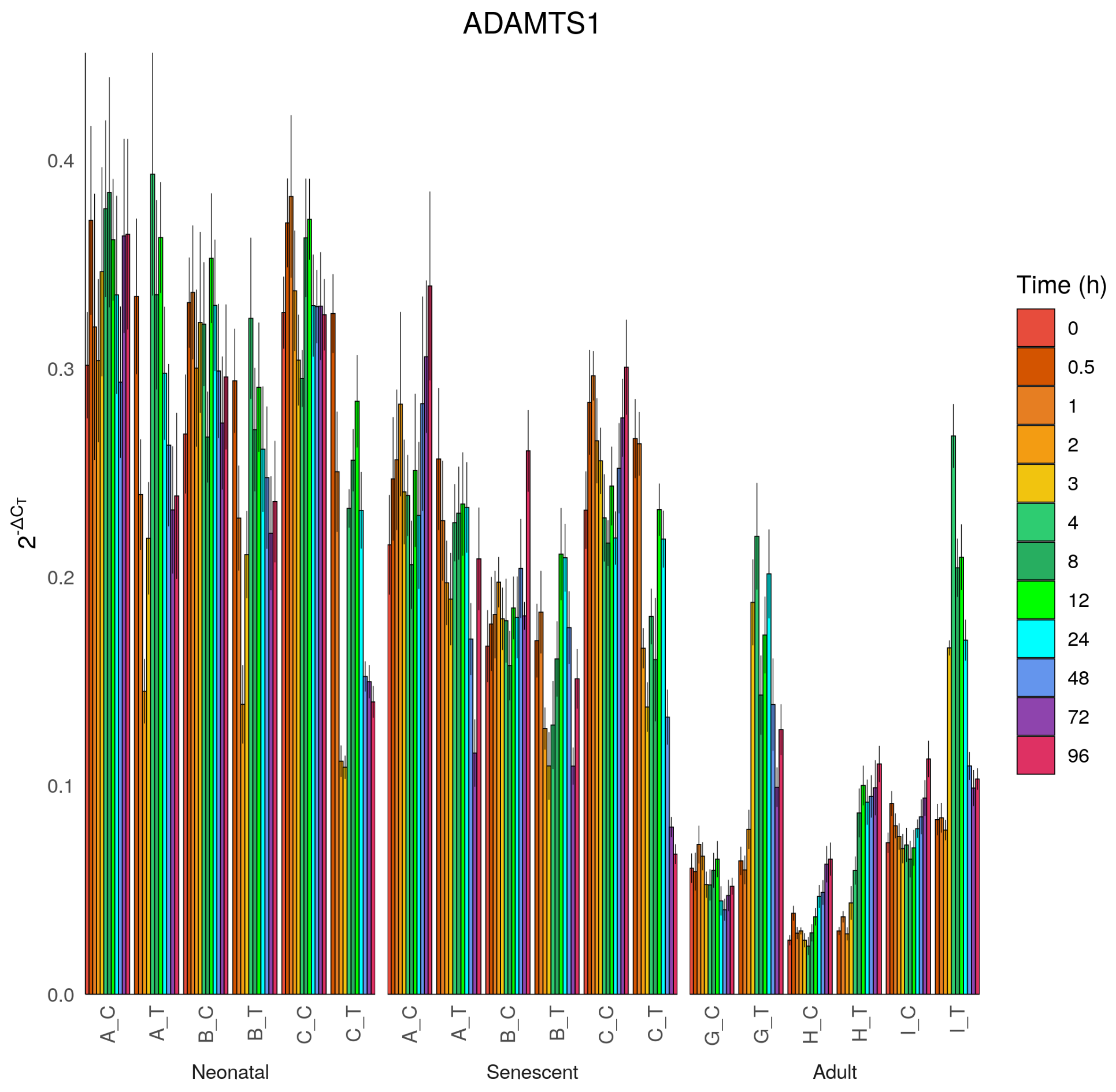
ADAMTS1
Time (h)
0
0.5
1
2
3
4
8
12
24
48
72
96
$2^{-\Delta C_T}$
0.0
0.1
0.2
0.3
0.4
A_C
A_T
B_C
B_T
C_C
C_T
G_C
G_T
H_C
H_T
I_C
I_T
Neonatal
Senescent
Adult

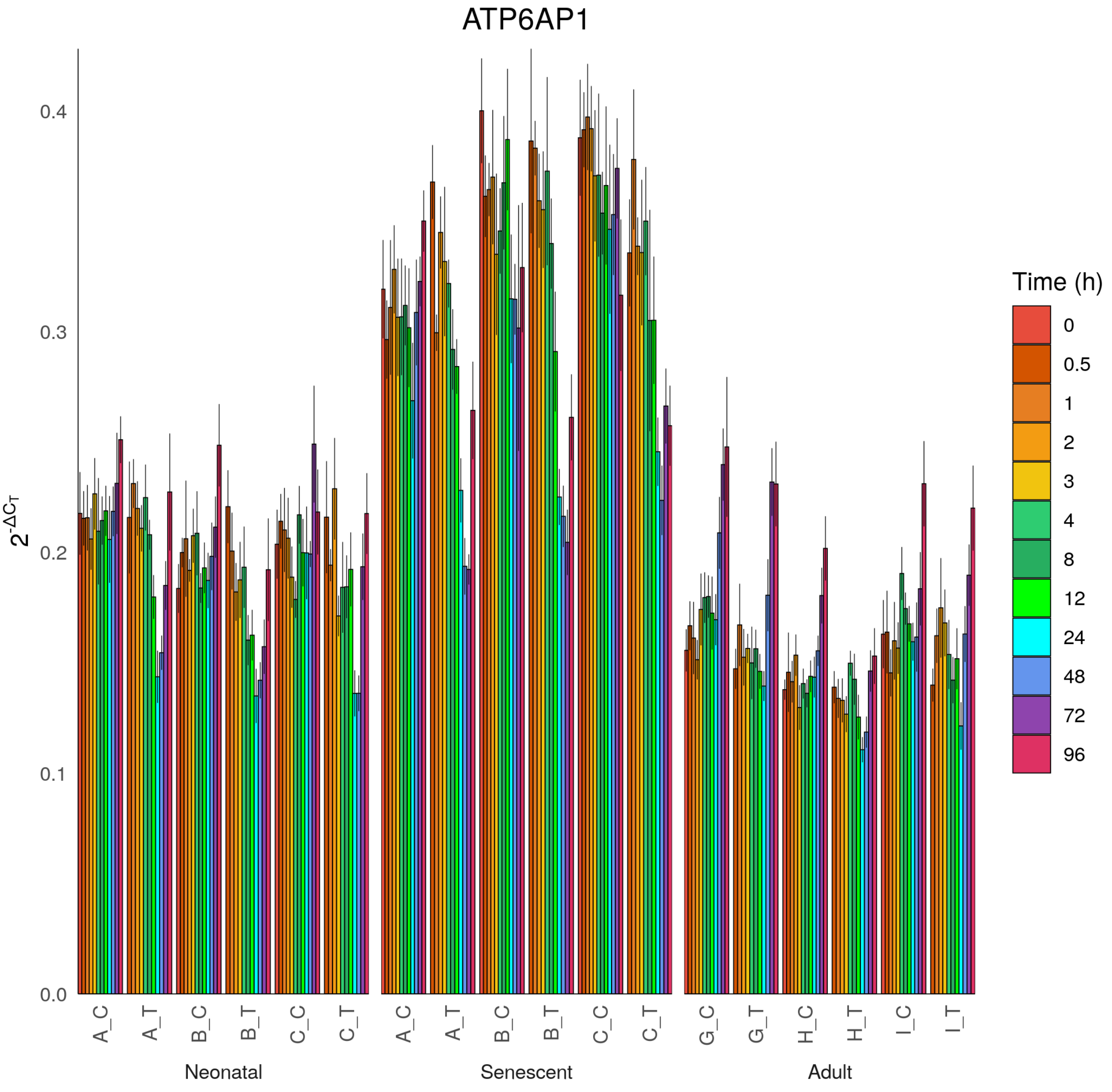

ATP6AP1
$2^{-\Delta C_T}$
0.0
0.1
0.2
0.3
0.4
A_C
A_T
B_C
B_T
C_C
C_T
A_C
A_T
B_C
B_T
C_C
C_T
G_C
G_T
H_C
H_T
I_C
I_T
Neonatal
Senescent
Adult
Time (h)
0
0.5
1
2
3
4
8
12
24
48
72
96

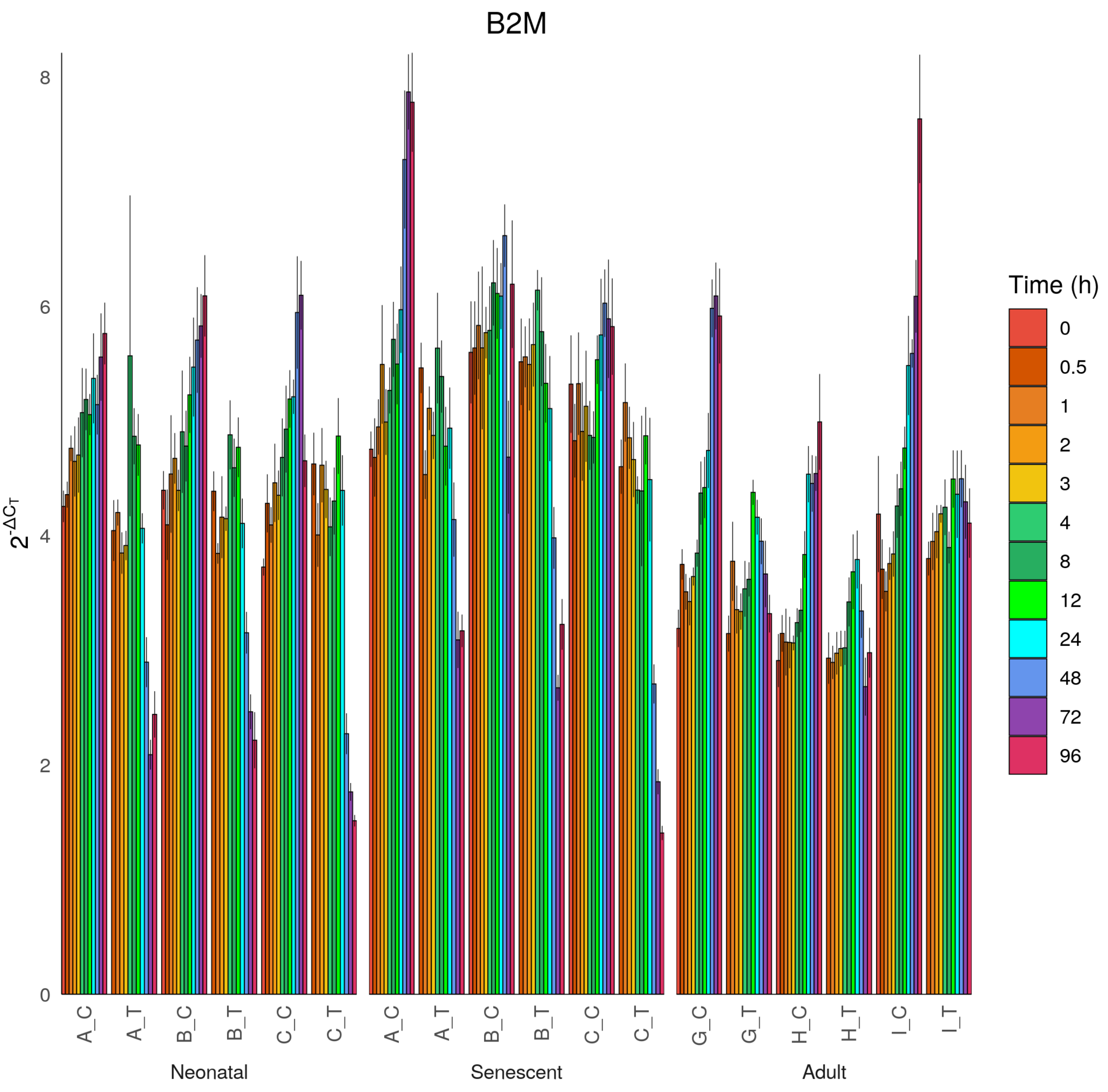

B2M
$2^{-\Delta C_T}$
0
2
4
6
8
A_C
A_T
B_C
B_T
C_C
C_T
A_C
A_T
B_C
B_T
C_C
C_T
G_C
G_T
H_C
H_T
I_C
I_T
Neonatal
Senescent
Adult
Time (h)
0
0.5
1
2
3
4
8
12
24
48
72
96

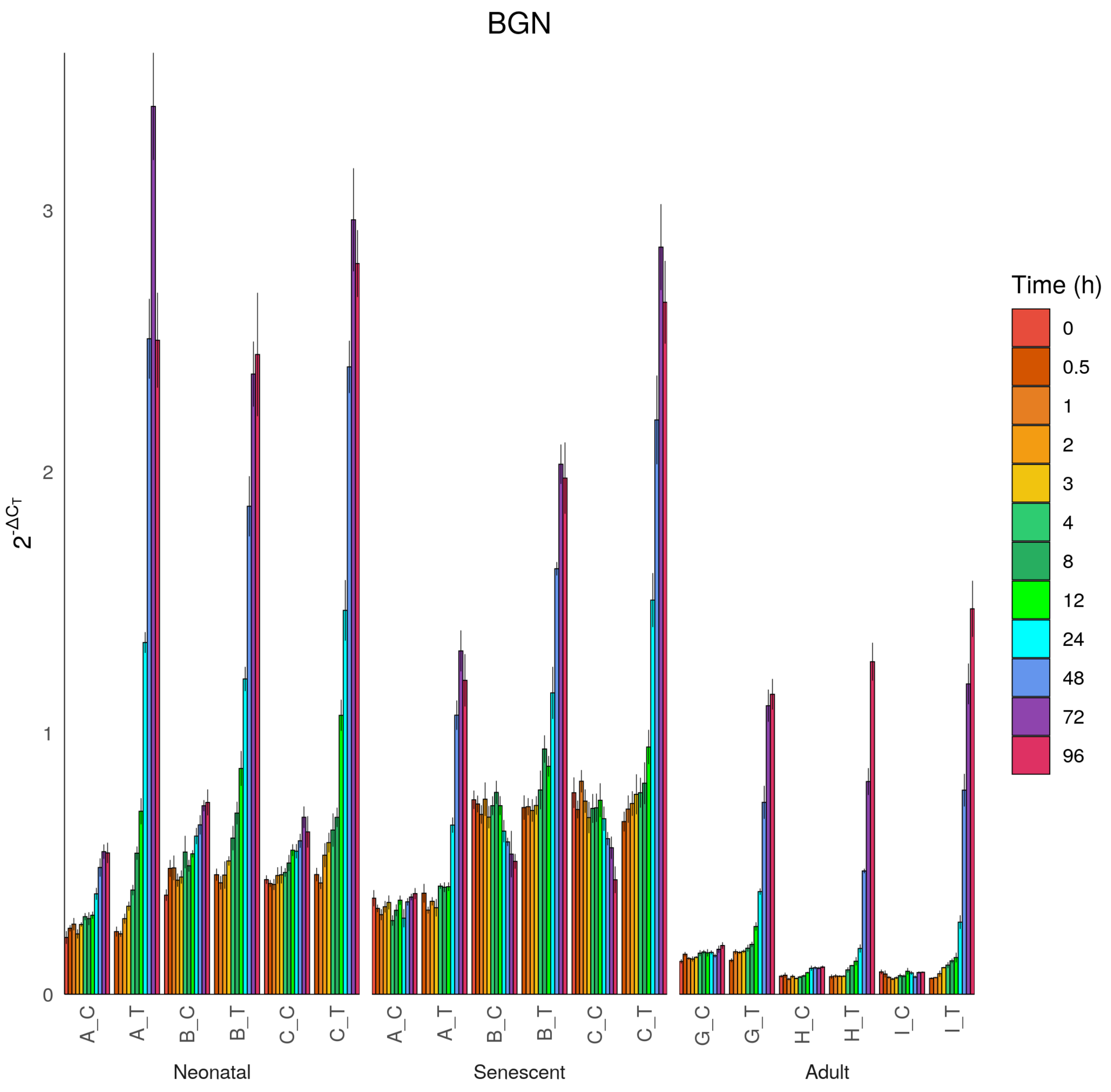
BGN
$2^{-\Delta C_T}$
0
1
2
3
A_C
A_T
B_C
B_T
C_C
C_T
G_C
G_T
H_C
H_T
I_C
I_T
Neonatal
Senescent
Adult
Time (h)
0
0.5
1
2
3
4
8
12
24
48
72
96

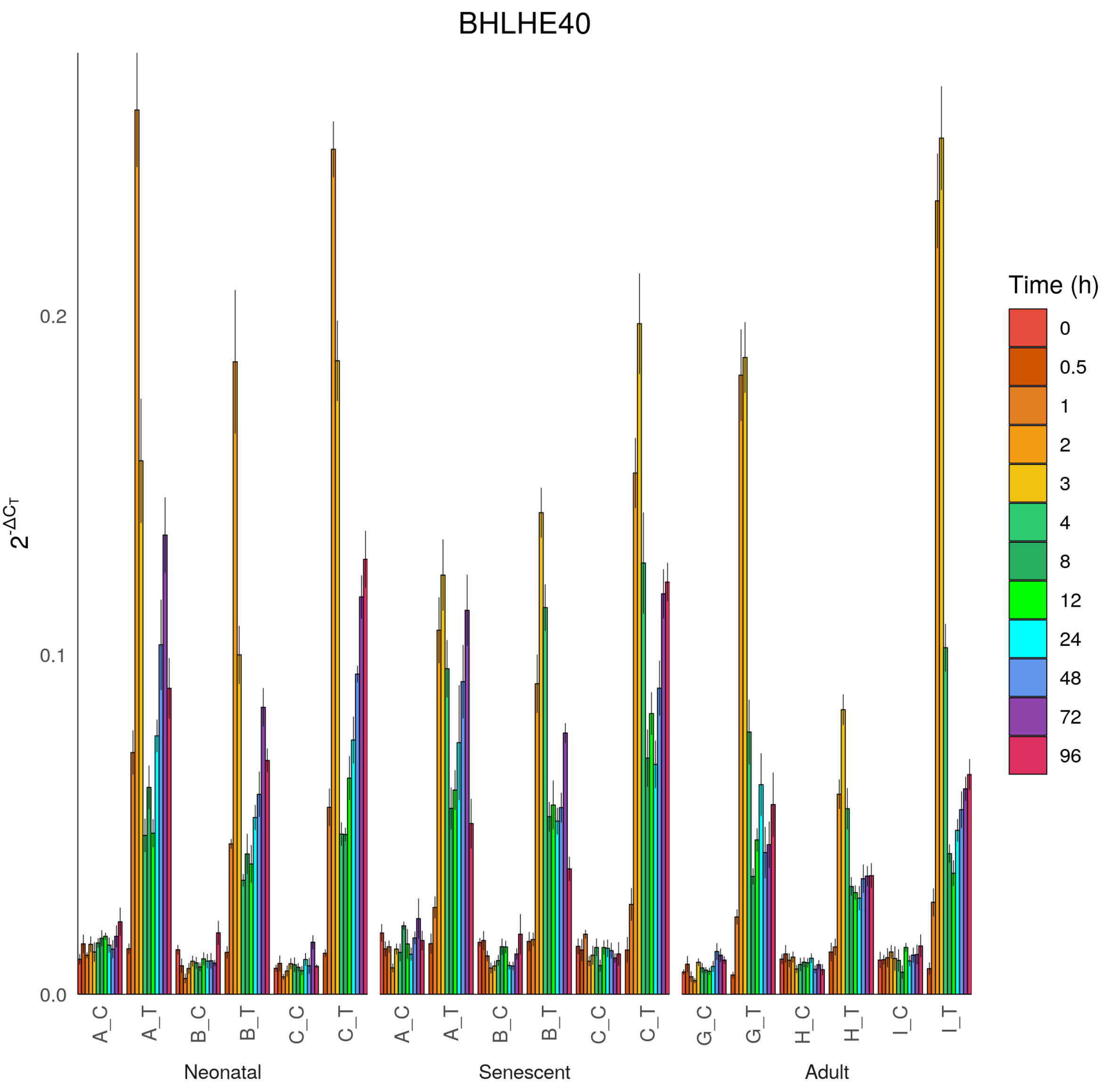

BHLHE40
$2^{-\Delta C_T}$
0.0
0.1
0.2
A_C
A_T
B_C
B_T
C_C
C_T
A_C
A_T
B_C
B_T
C_C
C_T
G_C
G_T
H_C
H_T
I_C
I_T
Neonatal
Senescent
Adult
Time (h)
0
0.5
1
2
3
4
8
12
24
48
72
96

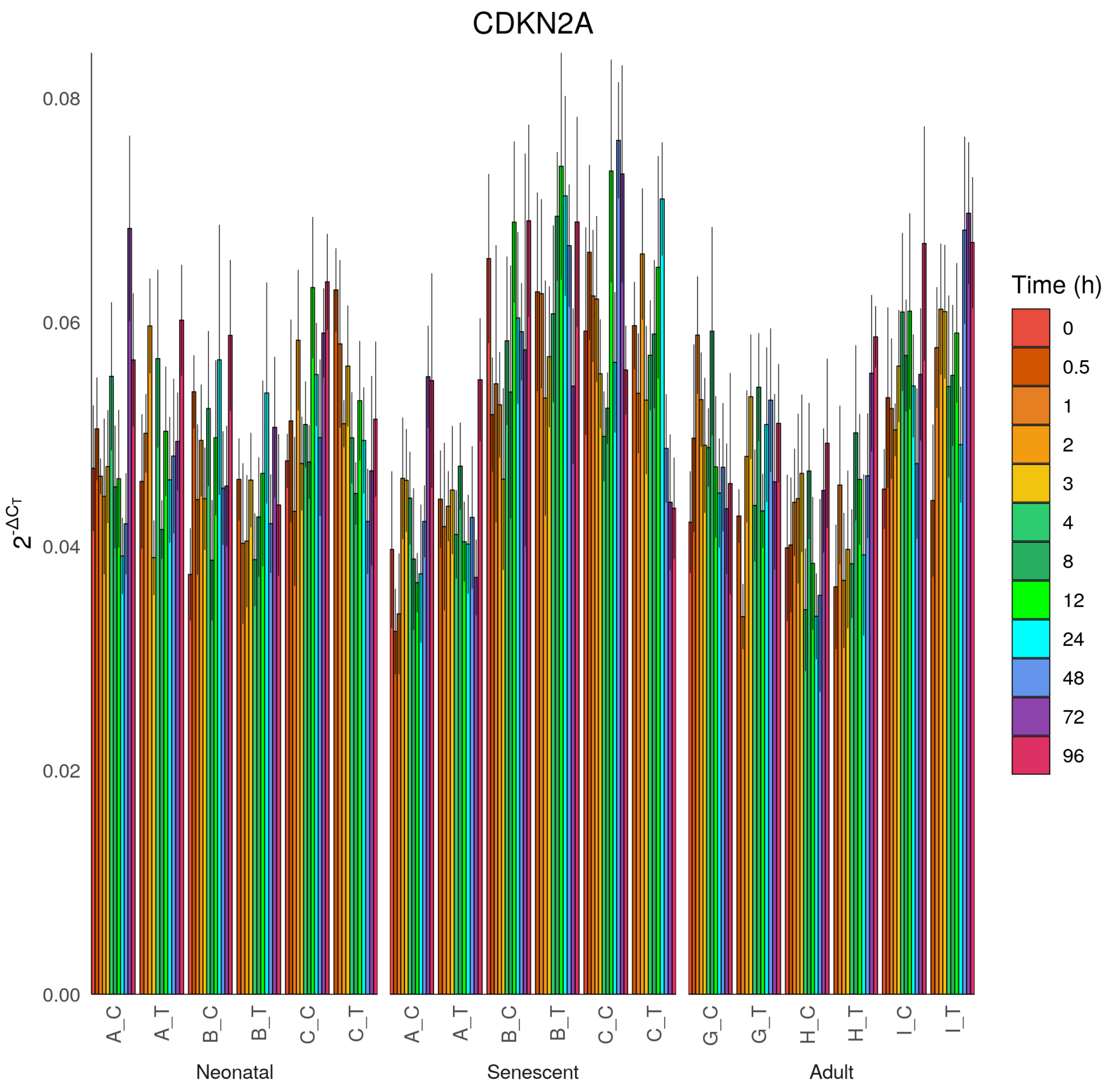

CDKN2A
0.08
0.06
0.04
0.02
0.00
$2^{-\Delta C_T}$
A_C
A_T
B_C
B_T
C_C
C_T
G_C
G_T
H_C
H_T
I_C
I_T
Neonatal
Senescent
Adult
Time (h)
0
0.5
1
2
3
4
8
12
24
48
72
96

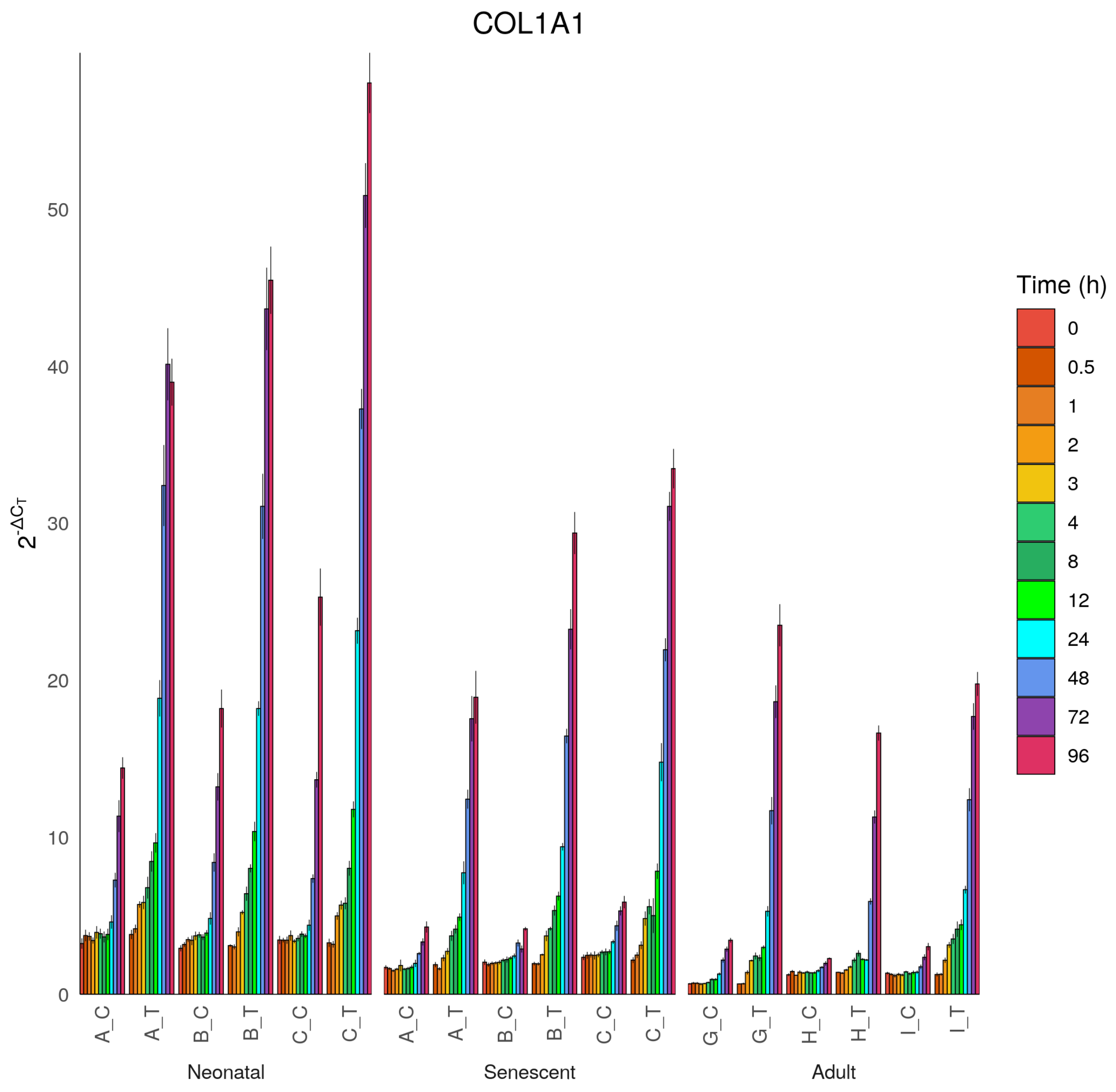
COL1A1
2^-ΔCT
0
10
20
30
40
50
A_C
A_T
B_C
B_T
C_C
C_T
Neonatal
A_C
A_T
B_C
B_T
C_C
C_T
Senescent
G_C
G_T
H_C
H_T
I_C
I_T
Adult
Time (h)
0
0.5
1
2
3
4
8
12
24
48
72
96

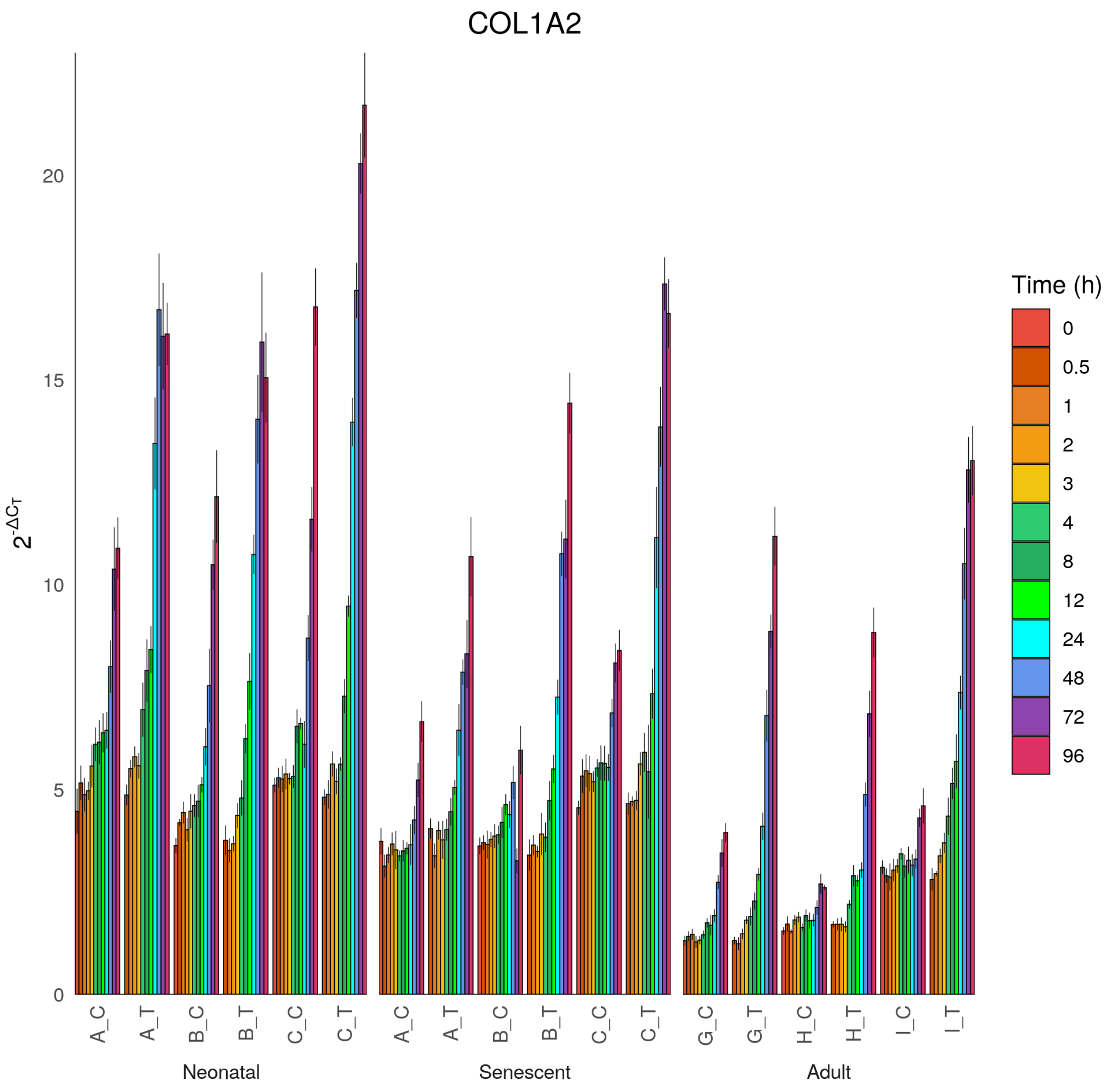
COL1A2
$2^{-\Delta C_T}$
0
5
10
15
20
A_C
A_T
B_C
B_T
C_C
C_T
A_C
A_T
B_C
B_T
C_C
C_T
G_C
G_T
H_C
H_T
I_C
I_T
Neonatal
Senescent
Adult
Time (h)
0
0.5
1
2
3
4
8
12
24
48
72
96

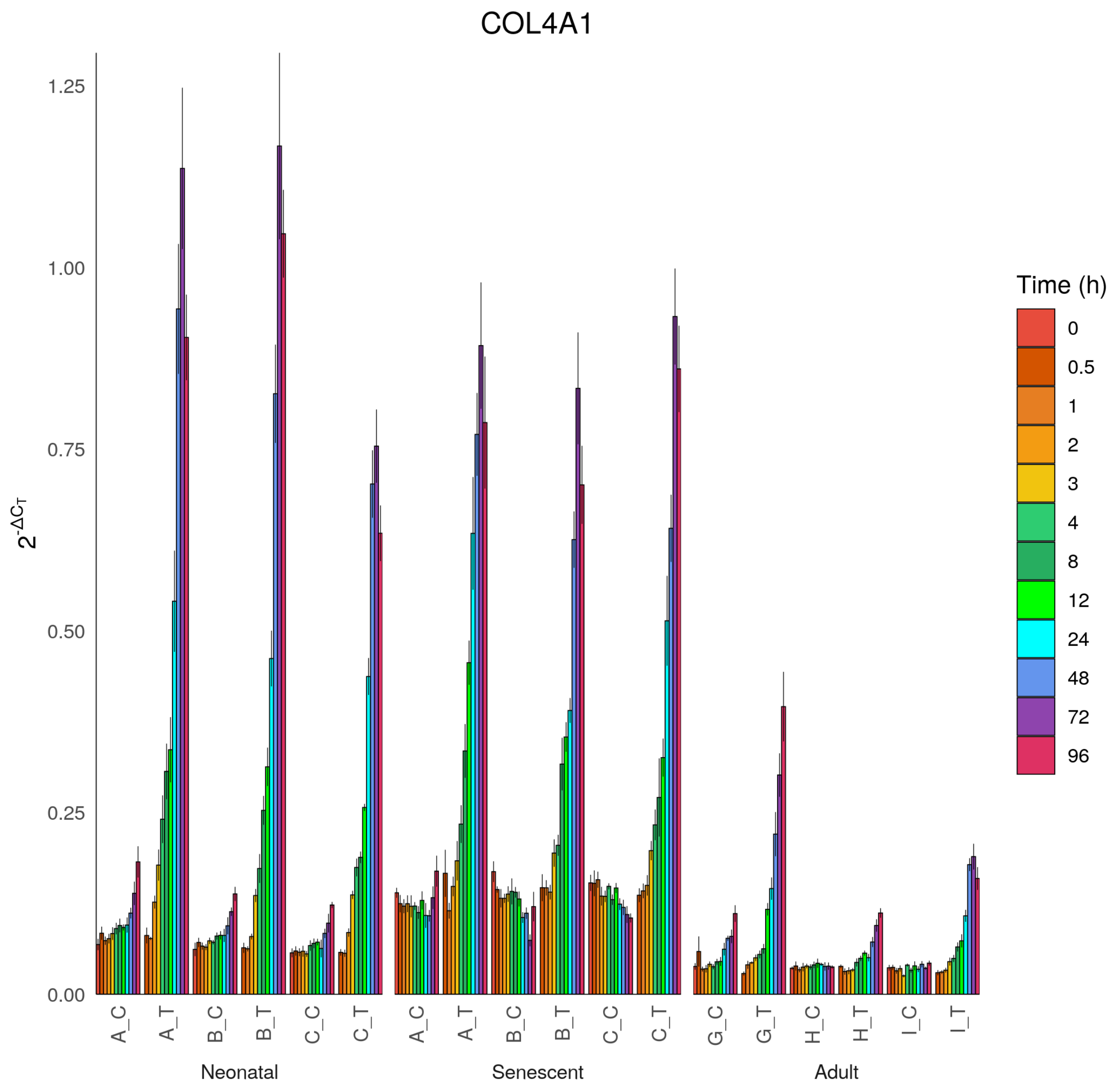

COL4A1
2^-ΔC_T
1.25
1.00
0.75
0.50
0.25
0.00
A_C
A_T
B_C
B_T
C_C
C_T
G_C
G_T
H_C
H_T
I_C
I_T
Neonatal
Senescent
Adult
Time (h)
0
0.5
1
2
3
4
8
12
24
48
72
96

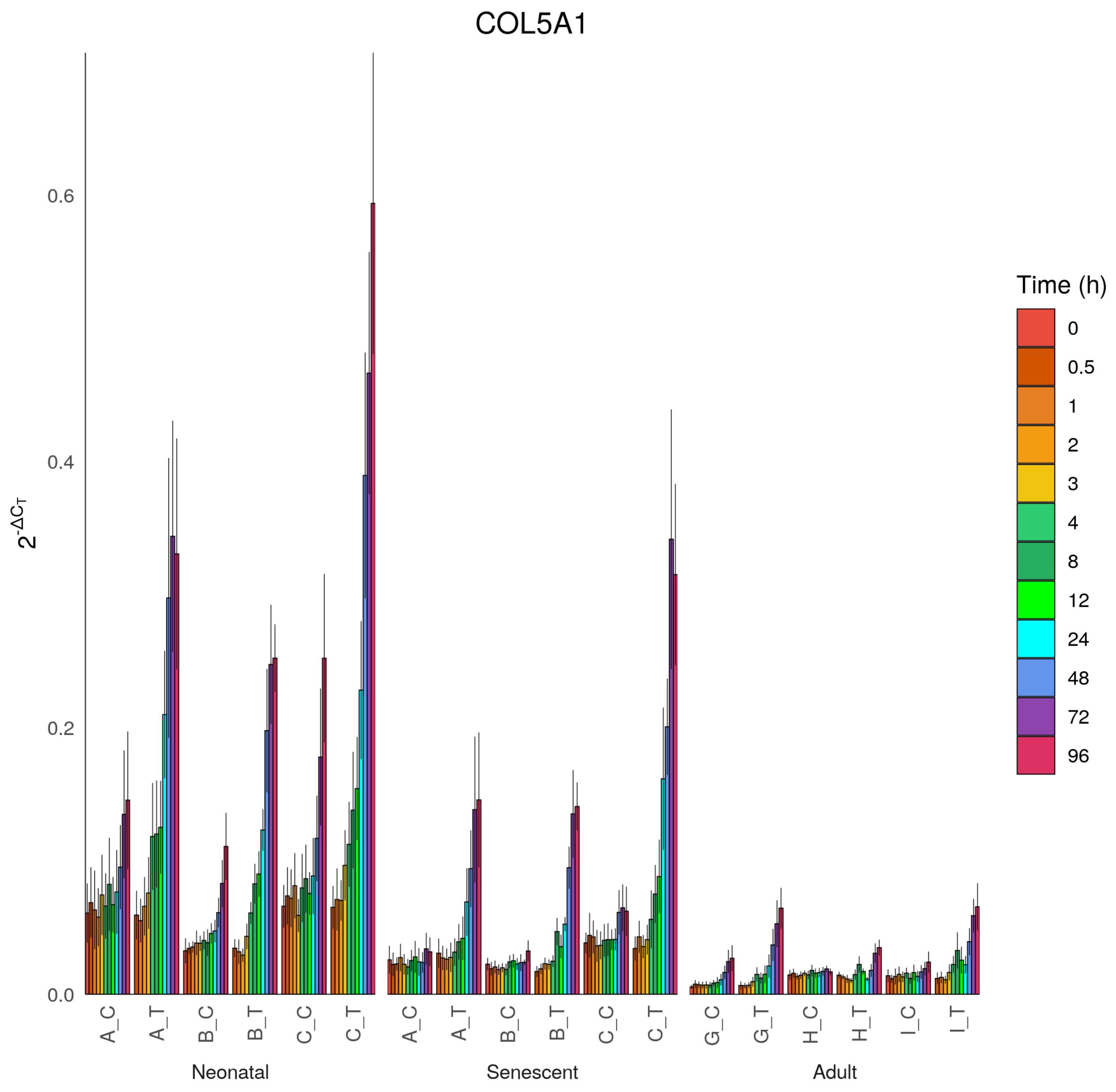

COL5A1
0.6
0.4
0.2
0.0
$2^{-\Delta C_T}$
A_C
A_T
B_C
B_T
C_C
C_T
G_C
G_T
H_C
H_T
I_C
I_T
Neonatal
Senescent
Adult
Time (h)
0
0.5
1
2
3
4
8
12
24
48
72
96

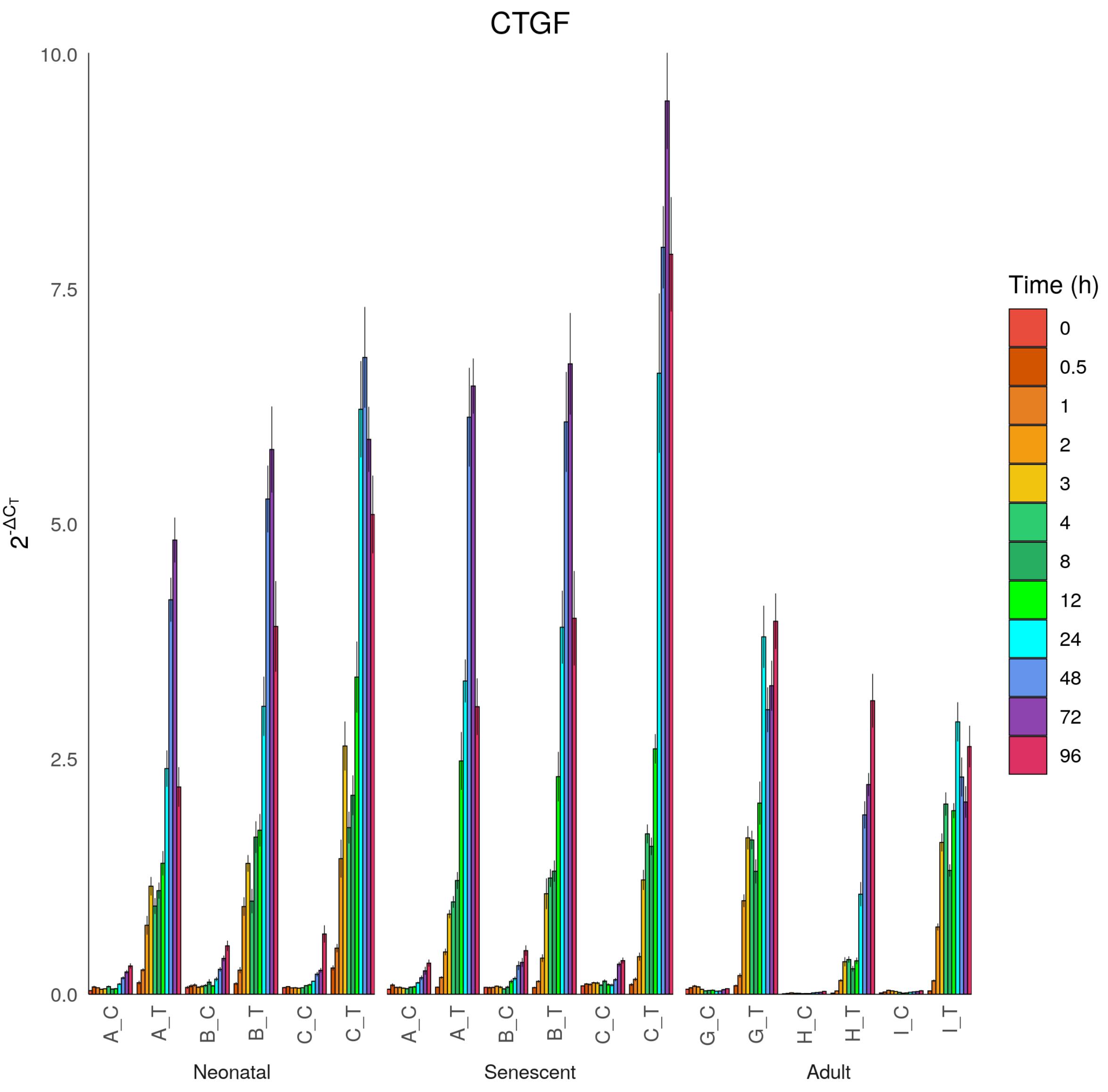
CTGF
$2^{-\Delta C_T}$
10.0
7.5
5.0
2.5
0.0
A_C
A_T
B_C
B_T
C_C
C_T
G_C
G_T
H_C
H_T
I_C
I_T
Neonatal
Senescent
Adult
Time (h)
0
0.5
1
2
3
4
8
12
24
48
72
96

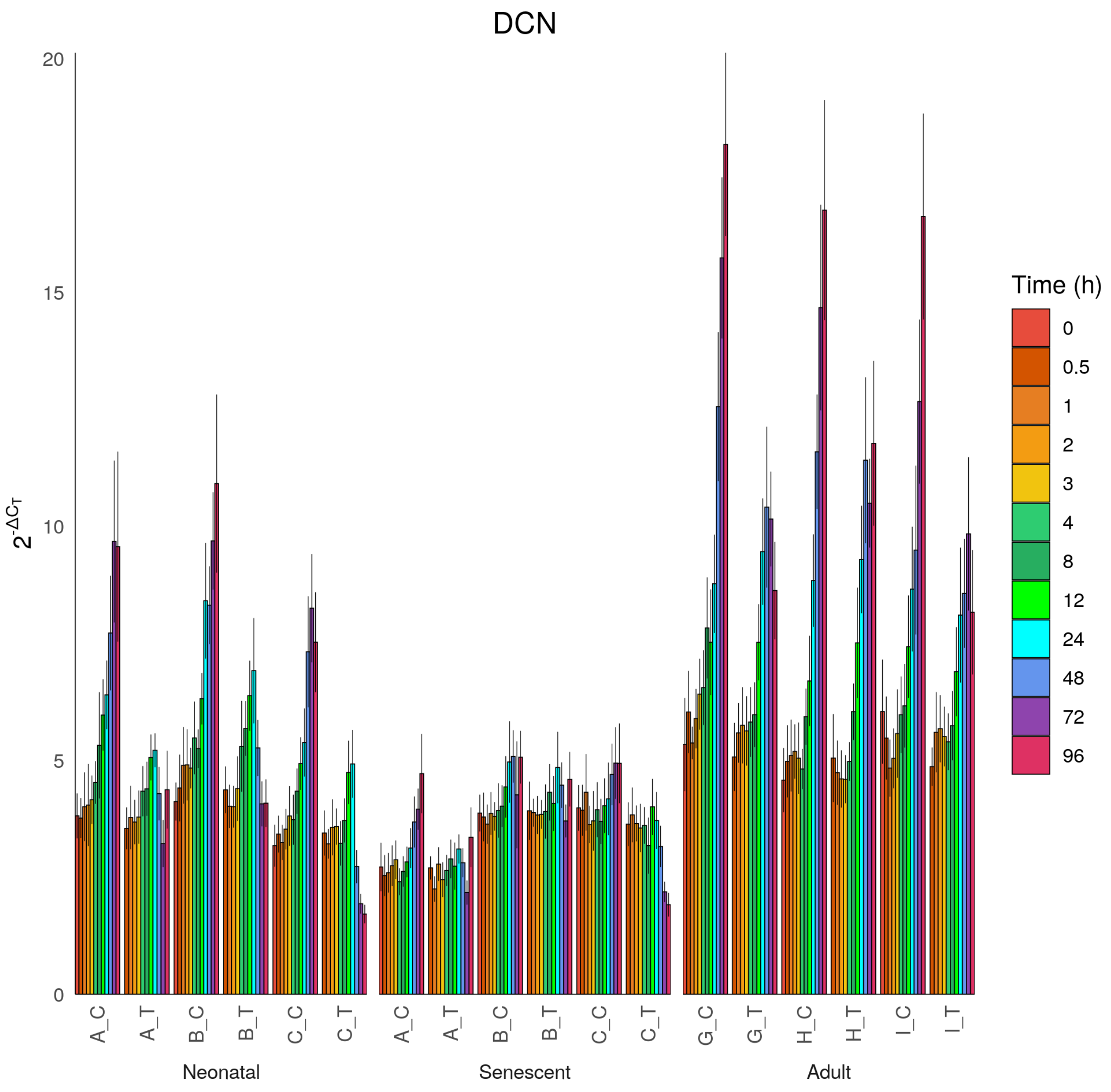

DCN
$2^{-\Delta C_T}$
20
15
10
5
0
A_C
A_T
B_C
B_T
C_C
C_T
G_C
G_T
H_C
H_T
I_C
I_T
Neonatal
Senescent
Adult
Time (h)
0
0.5
1
2
3
4
8
12
24
48
72
96

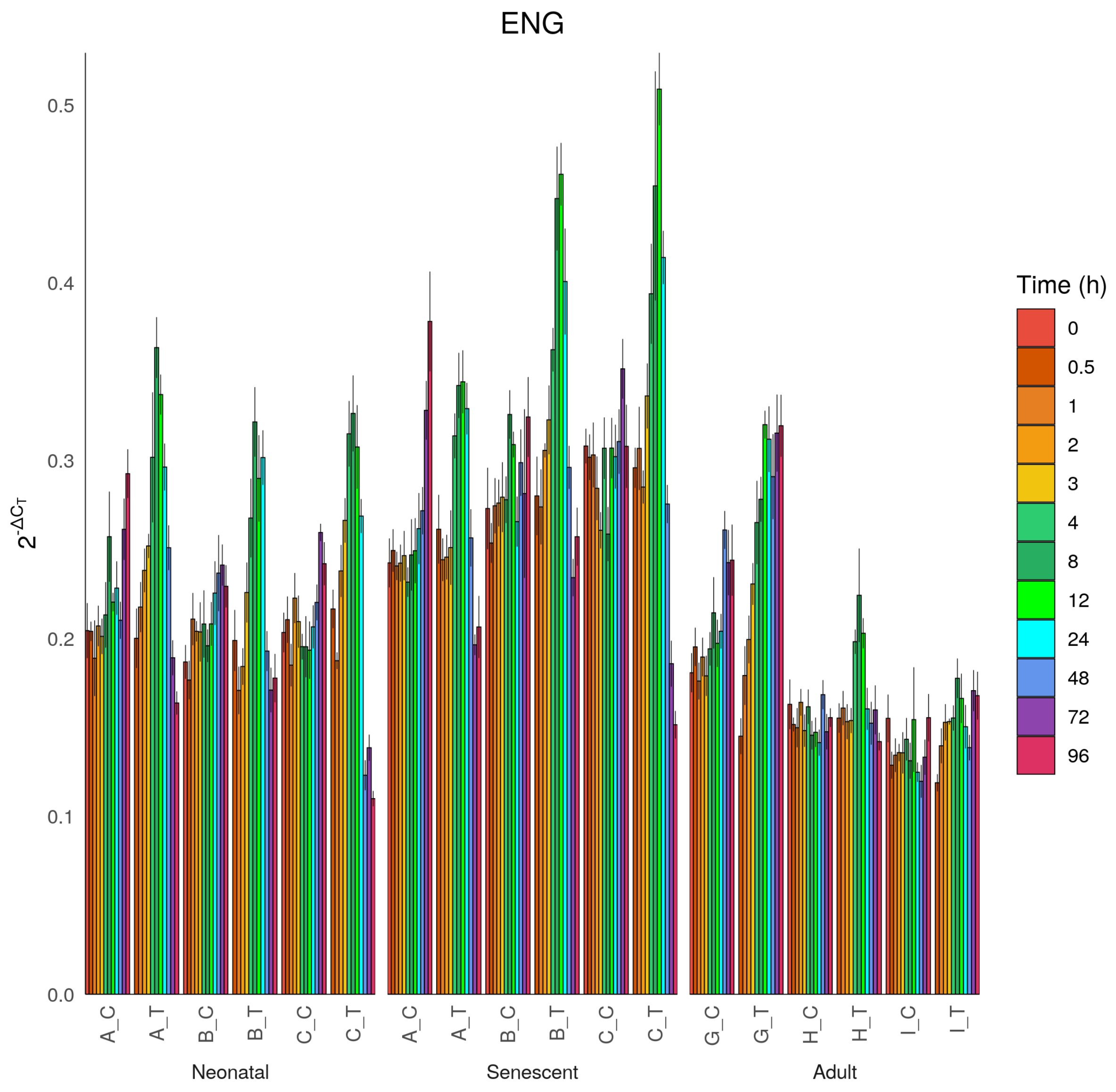
ENG
2^-ΔCT
0.0
0.1
0.2
0.3
0.4
0.5
A_C
A_T
B_C
B_T
C_C
C_T
G_C
G_T
H_C
H_T
I_C
I_T
Neonatal
Senescent
Adult
Time (h)
0
0.5
1
2
3
4
8
12
24
48
72
96

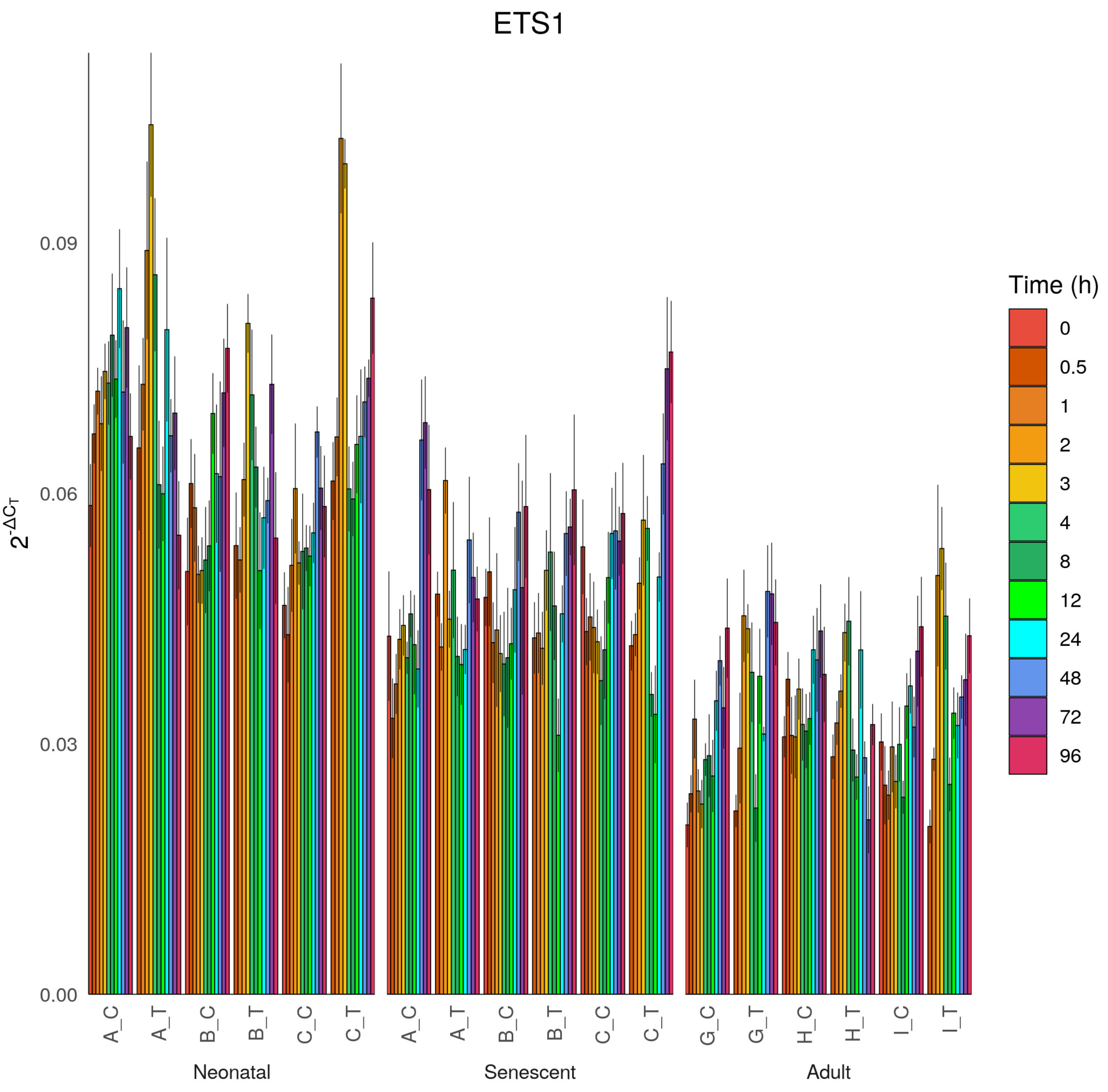
ETS1
$2^{-\Delta C_T}$
0.00
0.03
0.06
0.09
A_C
A_T
B_C
B_T
C_C
C_T
G_C
G_T
H_C
H_T
I_C
I_T
Neonatal
Senescent
Adult
Time (h)
0
0.5
1
2
3
4
8
12
24
48
72
96

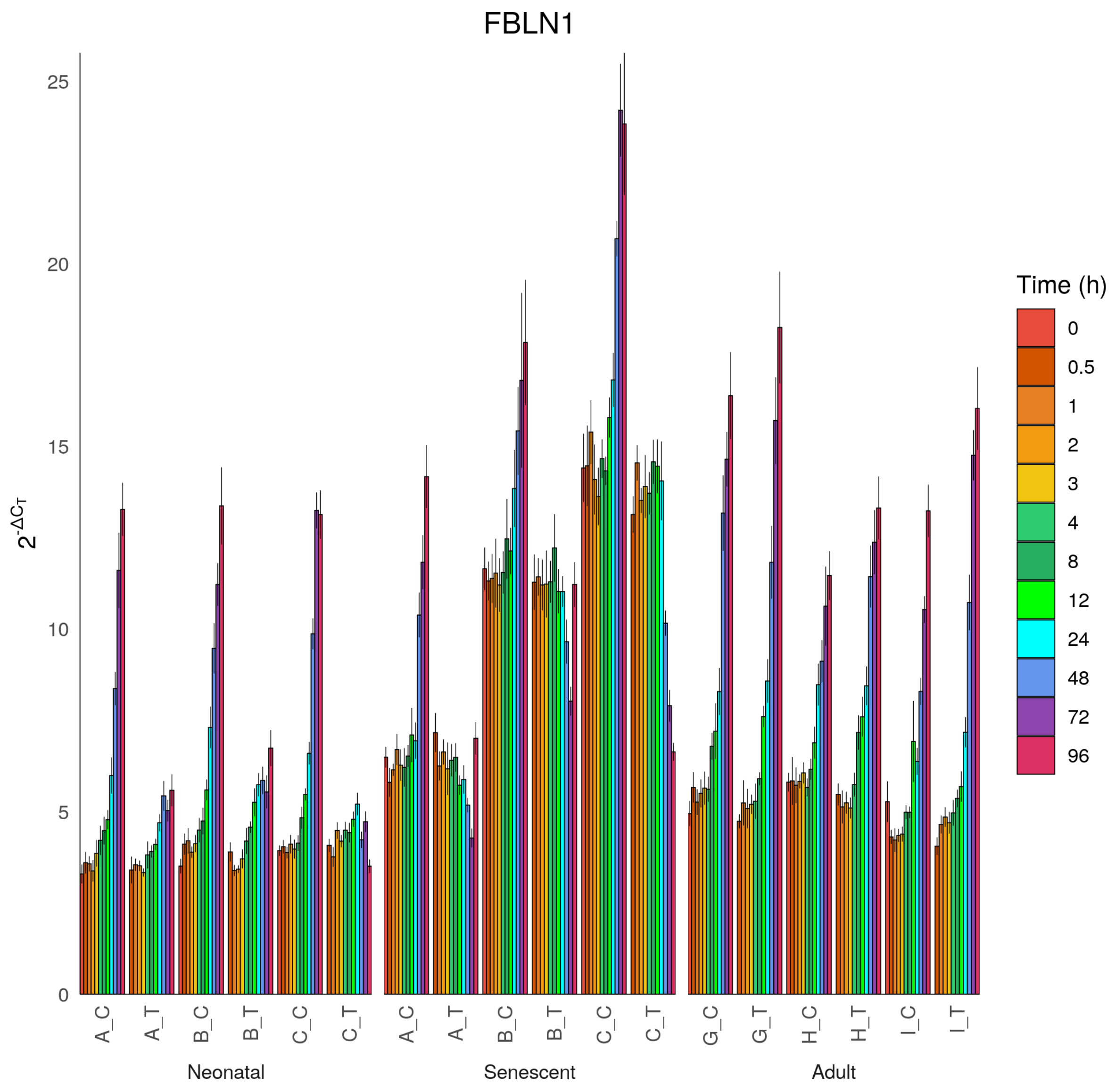
FBLN1
$2^{-\Delta C_T}$
25
20
15
10
5
0
A_C
A_T
B_C
B_T
C_C
C_T
A_C
A_T
B_C
B_T
C_C
C_T
G_C
G_T
H_C
H_T
I_C
I_T
Neonatal
Senescent
Adult
Time (h)
0
0.5
1
2
3
4
8
12
24
48
72
96

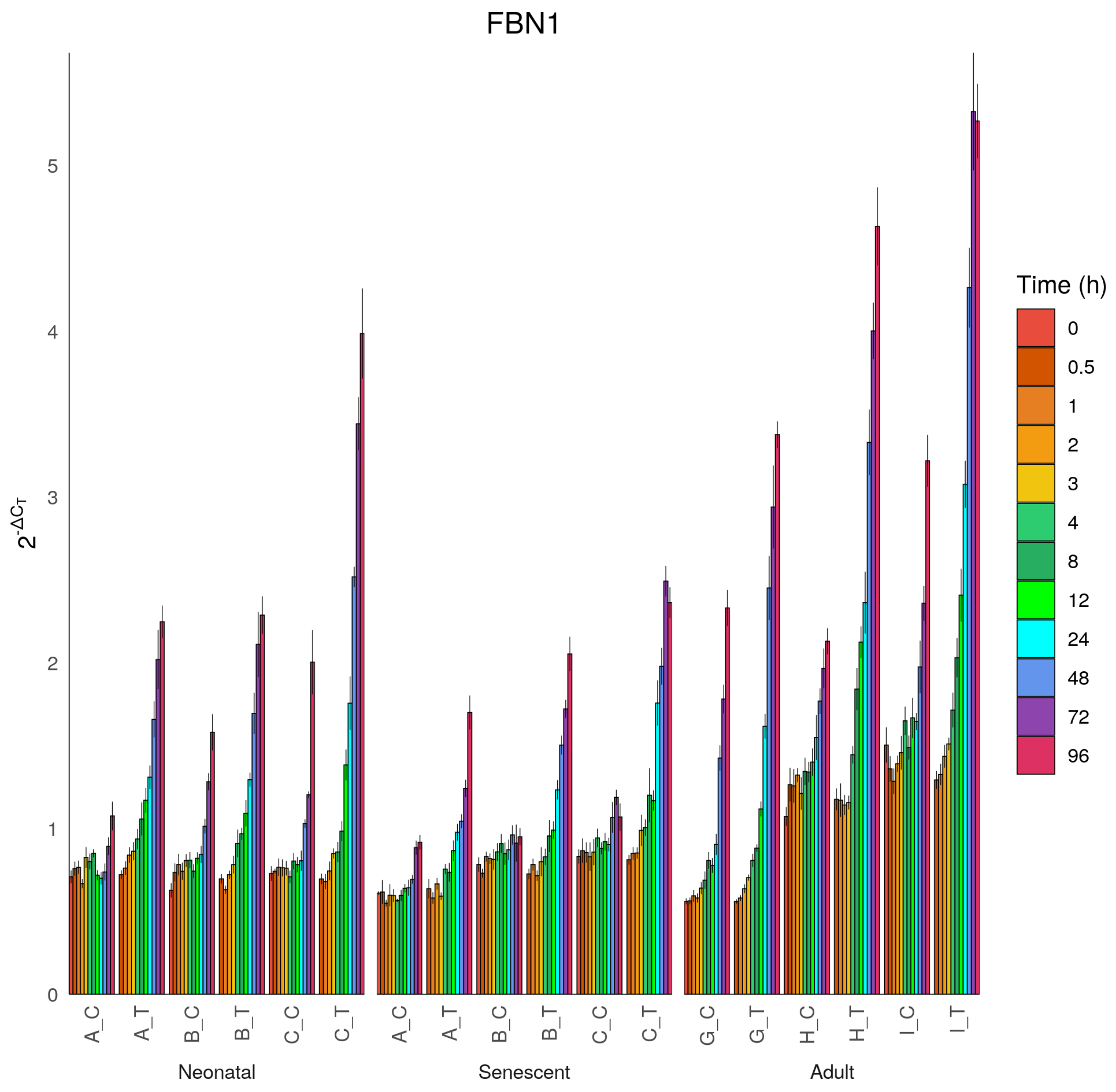
FBN1
$2^{-\Delta C_T}$
0
1
2
3
4
5
A_C
A_T
B_C
B_T
C_C
C_T
Neonatal
A_C
A_T
B_C
B_T
C_C
C_T
Senescent
G_C
G_T
H_C
H_T
I_C
I_T
Adult
Time (h)
0
0.5
1
2
3
4
8
12
24
48
72
96

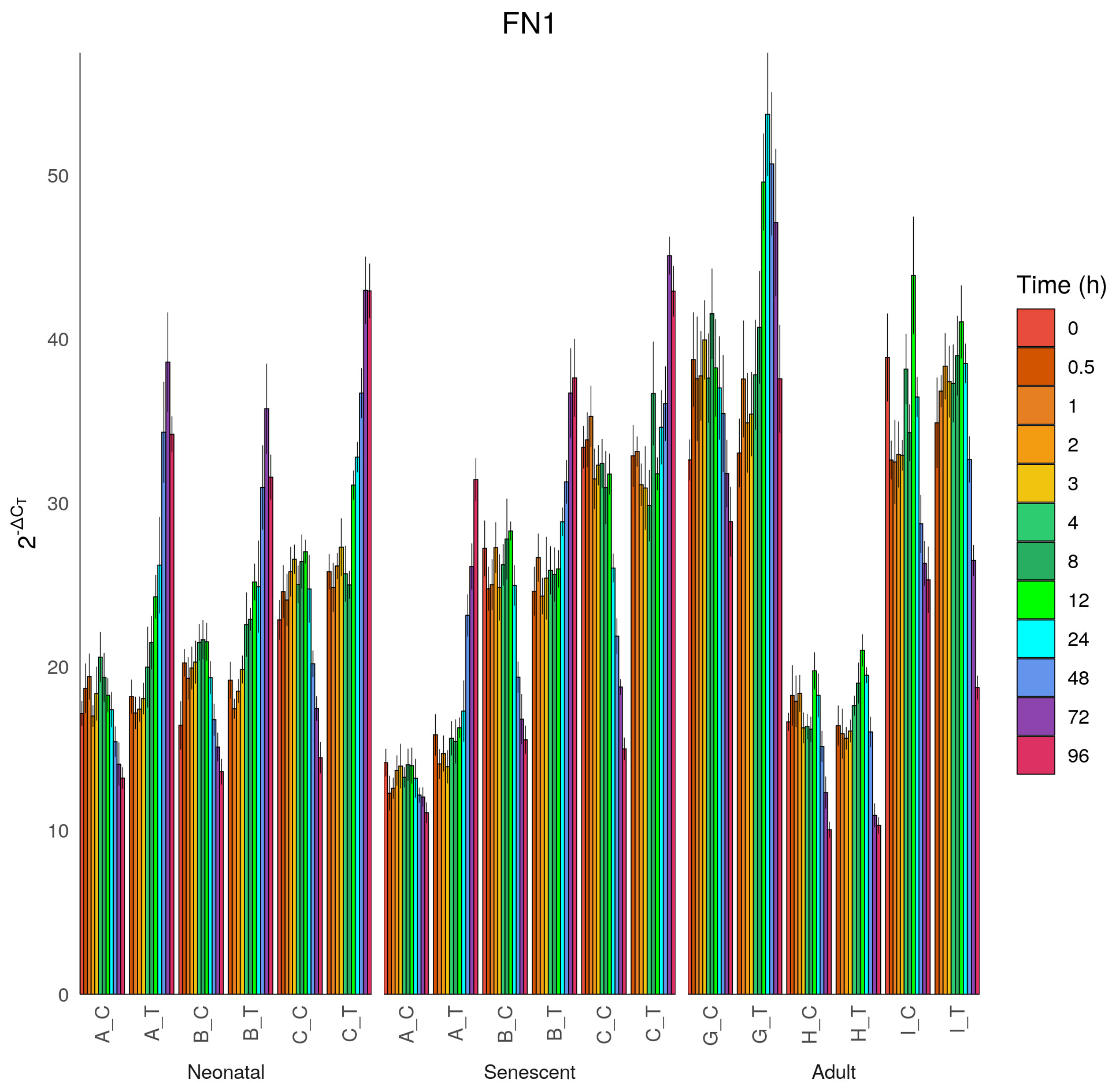
FN1
2^-ΔCT
0
10
20
30
40
50
A_C
A_T
B_C
B_T
C_C
C_T
A_C
A_T
B_C
B_T
C_C
C_T
G_C
G_T
H_C
H_T
I_C
I_T
Neonatal
Senescent
Adult
Time (h)
0
0.5
1
2
3
4
8
12
24
48
72
96

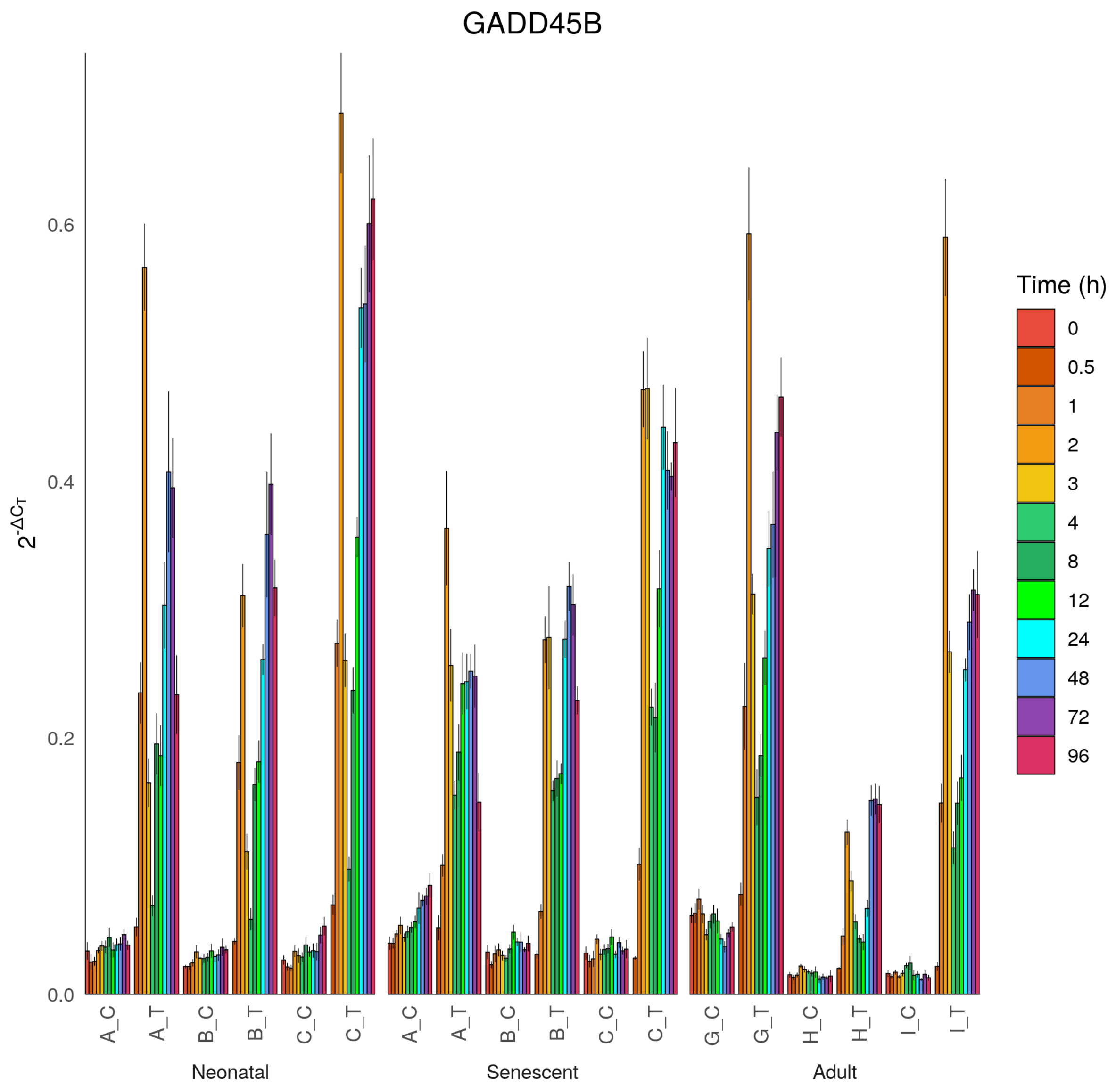
GADD45B
2^-ΔCT
0.0
0.2
0.4
0.6
A_C
A_T
B_C
B_T
C_C
C_T
G_C
G_T
H_C
H_T
I_C
I_T
Neonatal
Senescent
Adult
Time (h)
0
0.5
1
2
3
4
8
12
24
48
72
96

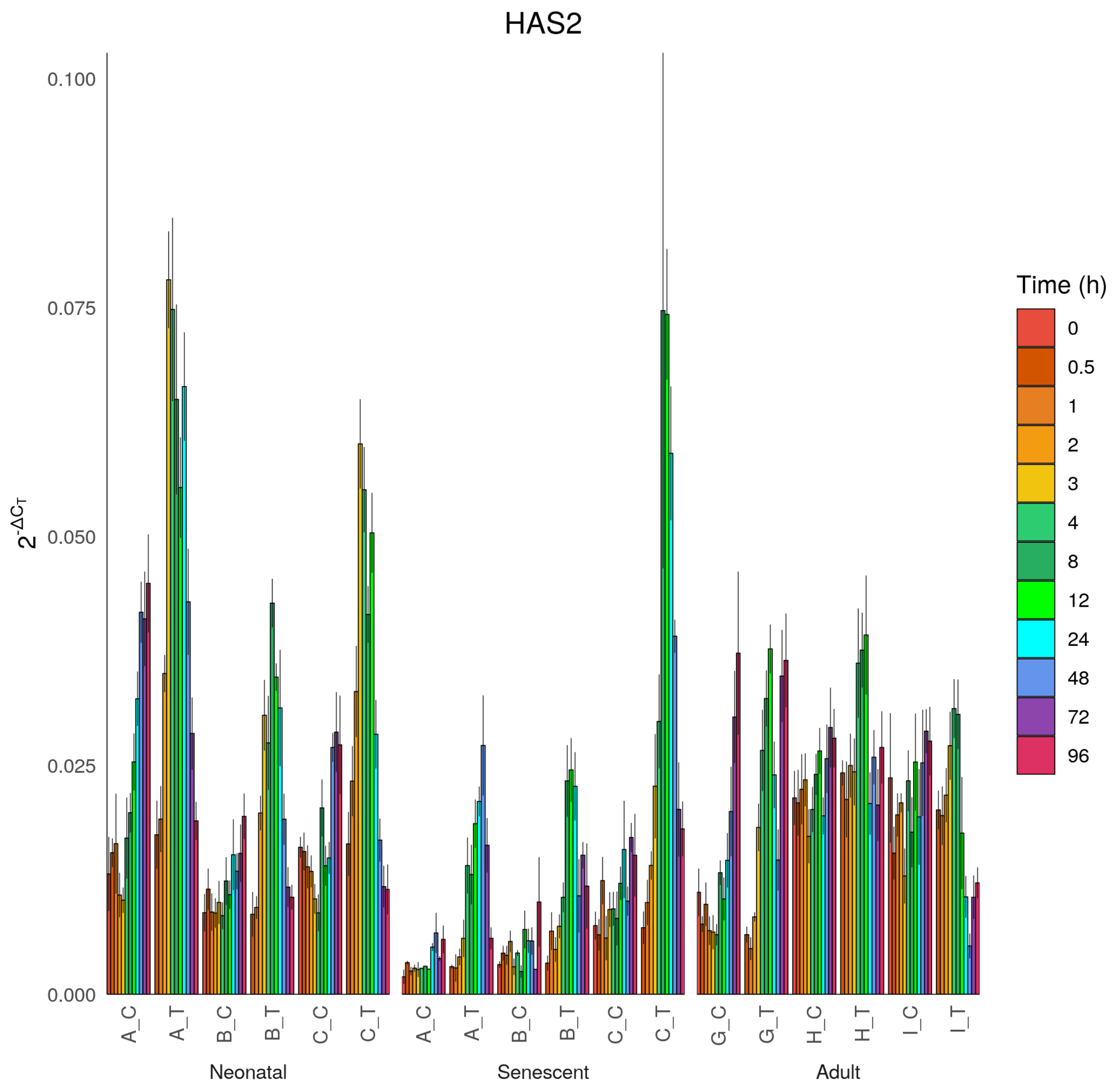
HAS2
$2^{-\Delta C_T}$
0.100
0.075
0.050
0.025
0.000
A_C
A_T
B_C
B_T
C_C
C_T
G_C
G_T
H_C
H_T
I_C
I_T
Neonatal
Senescent
Adult
Time (h)
0
0.5
1
2
3
4
8
12
24
48
72
96

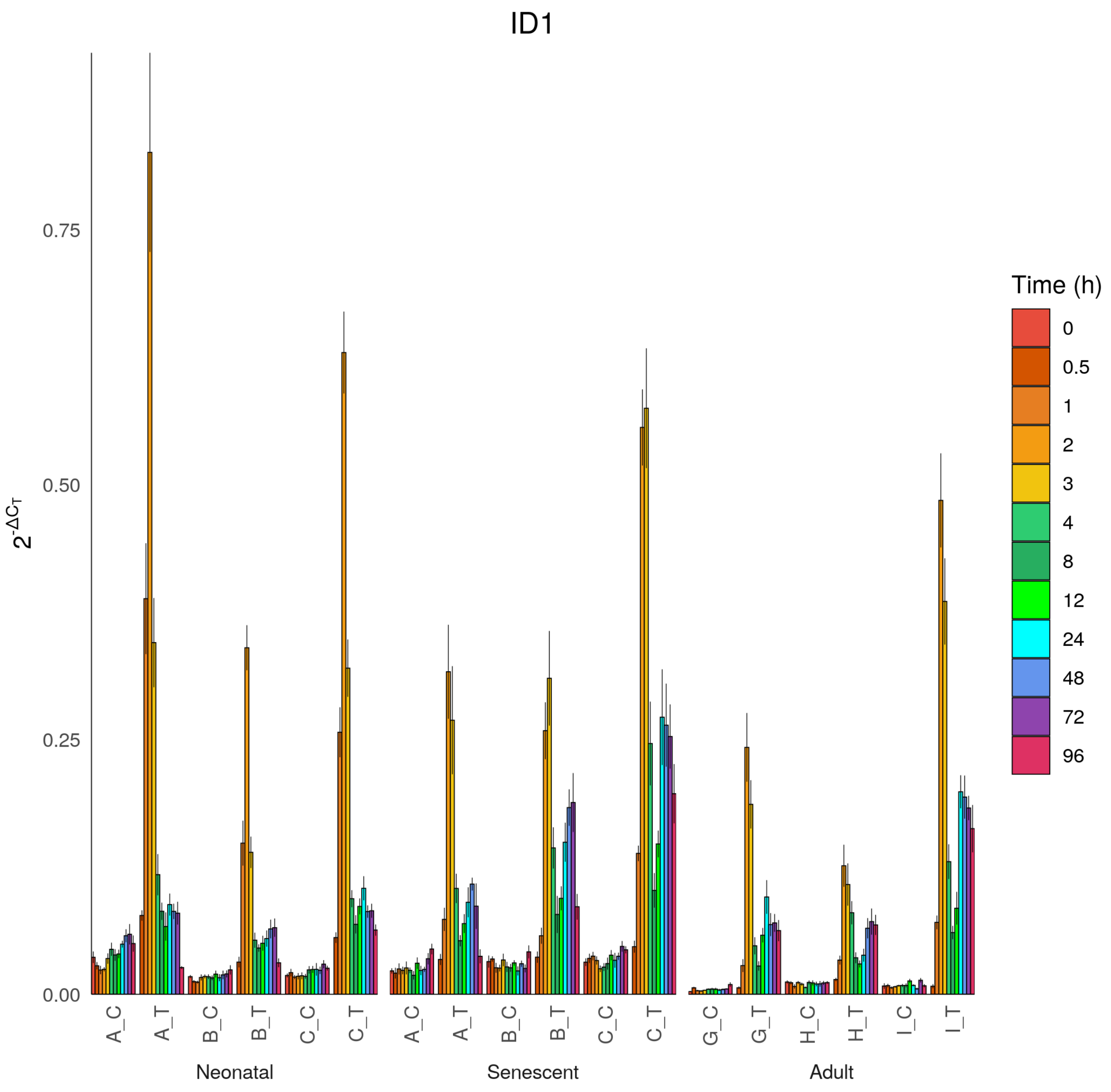
ID1
$2^{-\Delta C_T}$
0.00
0.25
0.50
0.75
A_C
A_T
B_C
B_T
C_C
C_T
A_C
A_T
B_C
B_T
C_C
C_T
G_C
G_T
H_C
H_T
I_C
I_T
Neonatal
Senescent
Adult
Time (h)
0
0.5
1
2
3
4
8
12
24
48
72
96

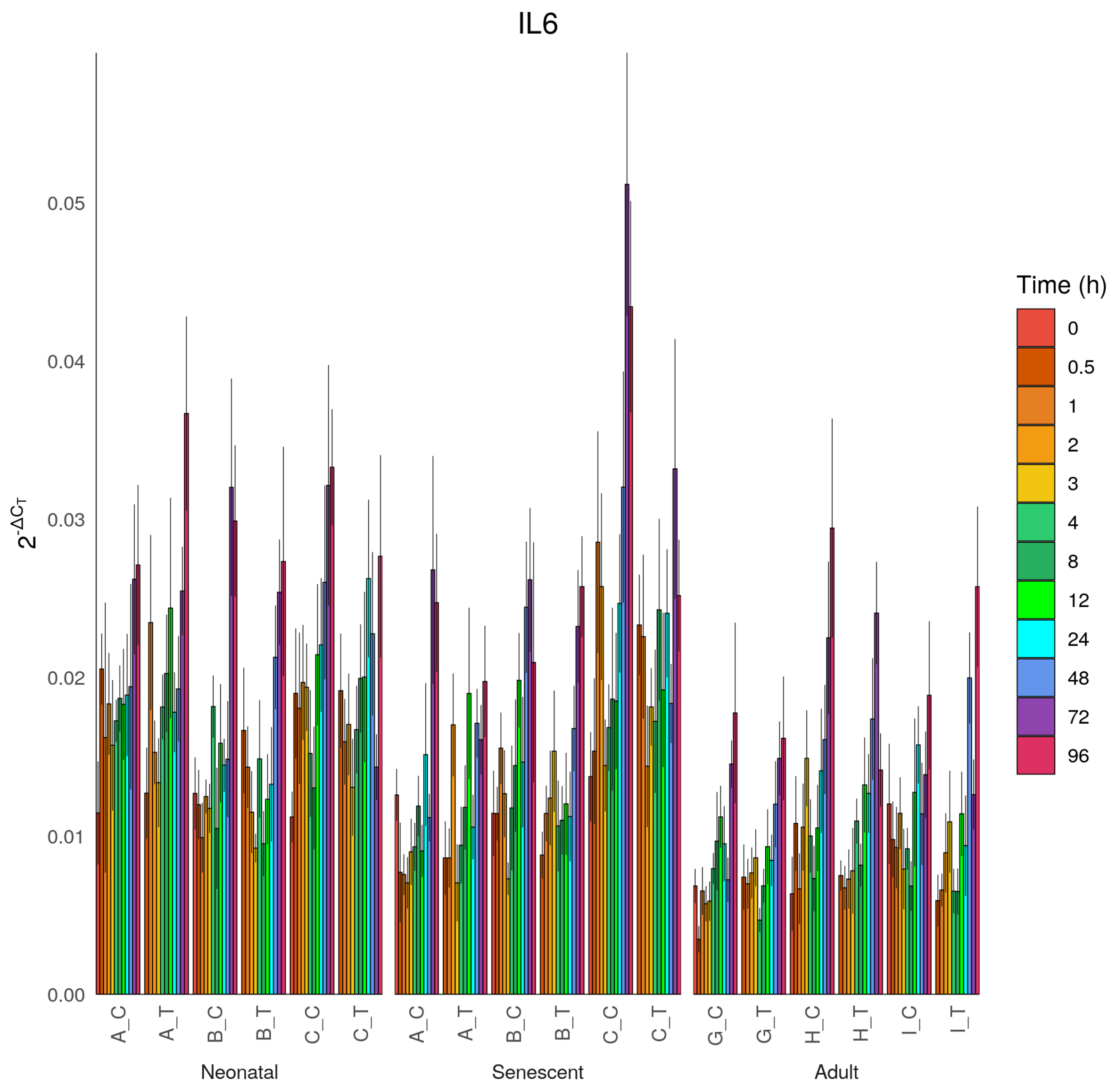

IL6
Time (h)
0
0.5
1
2
3
4
8
12
24
48
72
96
$2^{-\Delta C_T}$
0.00
0.01
0.02
0.03
0.04
0.05
A_C
A_T
B_C
B_T
C_C
C_T
G_C
G_T
H_C
H_T
I_C
I_T
Neonatal
Senescent
Adult

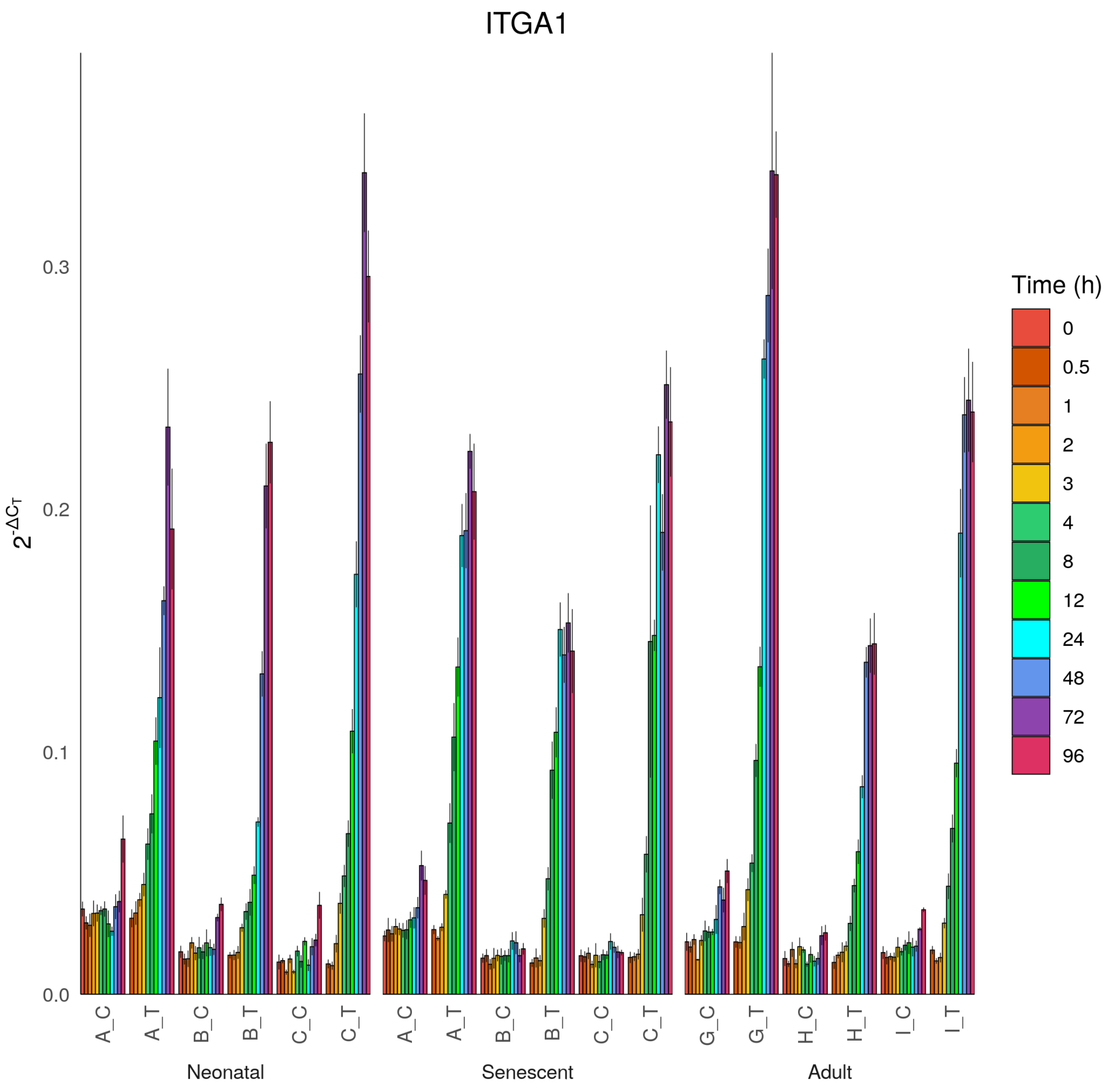

ITGA1
$2^{-\Delta C_T}$
0.0
0.1
0.2
0.3
A_C
A_T
B_C
B_T
C_C
C_T
G_C
G_T
H_C
H_T
I_C
I_T
Neonatal
Senescent
Adult
Time (h)
0
0.5
1
2
3
4
8
12
24
48
72
96

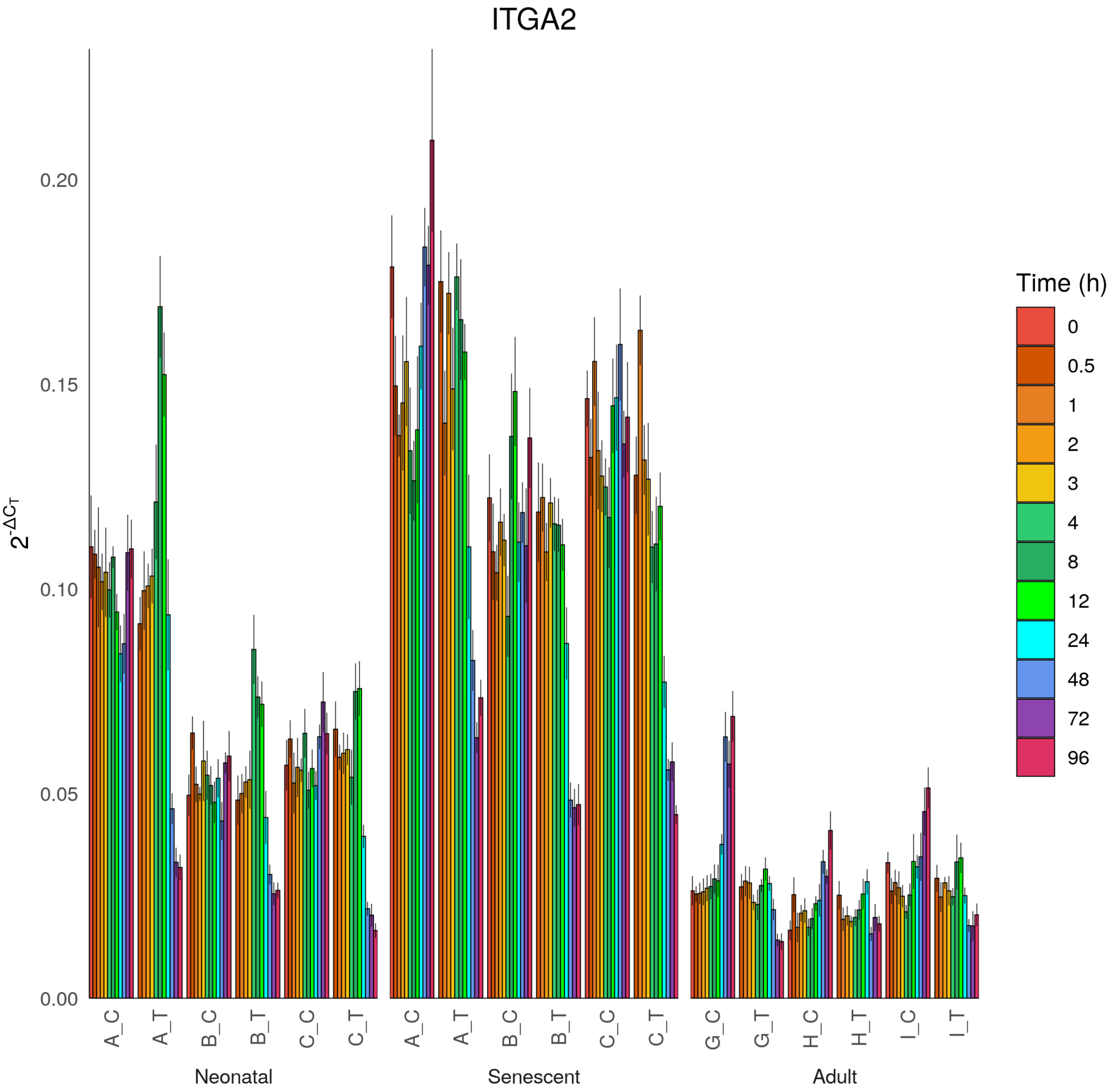

ITGA2
$2^{-\Delta C_T}$
0.20
0.15
0.10
0.05
0.00
A_C
A_T
B_C
B_T
C_C
C_T
G_C
G_T
H_C
H_T
I_C
I_T
Neonatal
Senescent
Adult
Time (h)
0
0.5
1
2
3
4
8
12
24
48
72
96

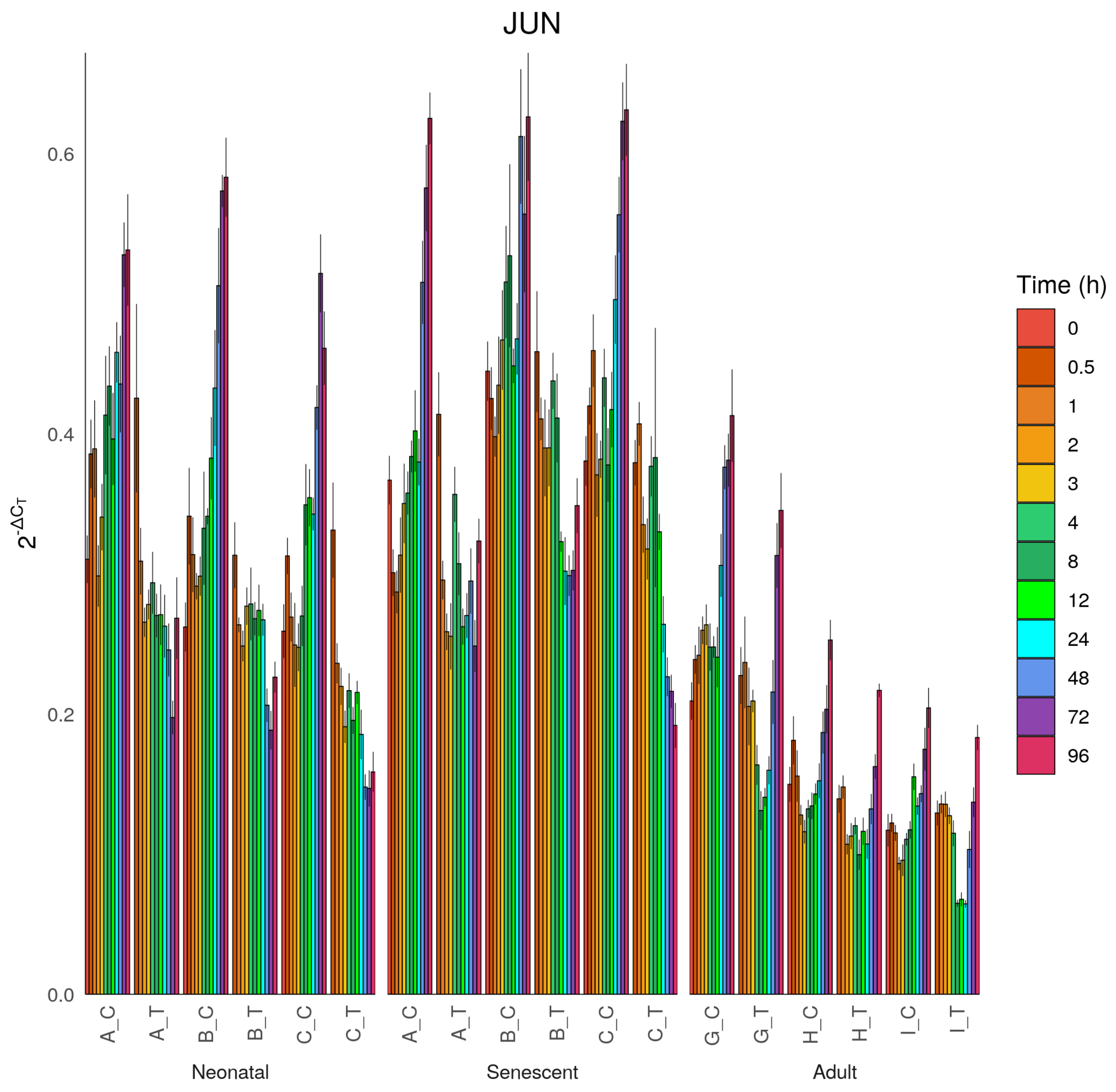

JUN
$2^{-\Delta C_T}$
0.0
0.2
0.4
0.6
A_C
A_T
B_C
B_T
C_C
C_T
G_C
G_T
H_C
H_T
I_C
I_T
Neonatal
Senescent
Adult
Time (h)
0
0.5
1
2
3
4
8
12
24
48
72
96

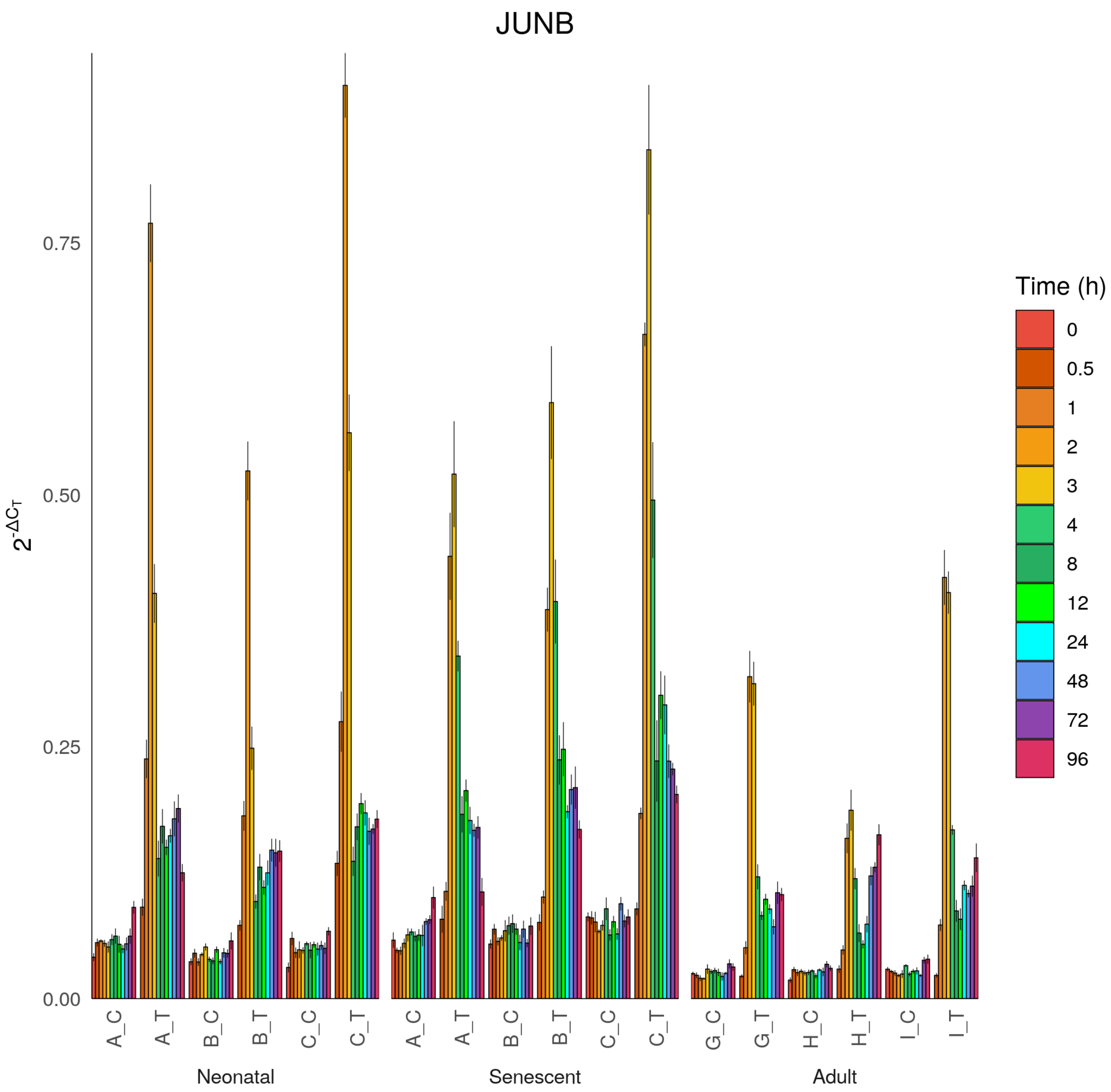
JUNB
$2^{-\Delta C_T}$
0.00
0.25
0.50
0.75
A_C
A_T
B_C
B_T
C_C
C_T
A_C
A_T
B_C
B_T
C_C
C_T
G_C
G_T
H_C
H_T
I_C
I_T
Neonatal
Senescent
Adult
Time (h)
0
0.5
1
2
3
4
8
12
24
48
72
96

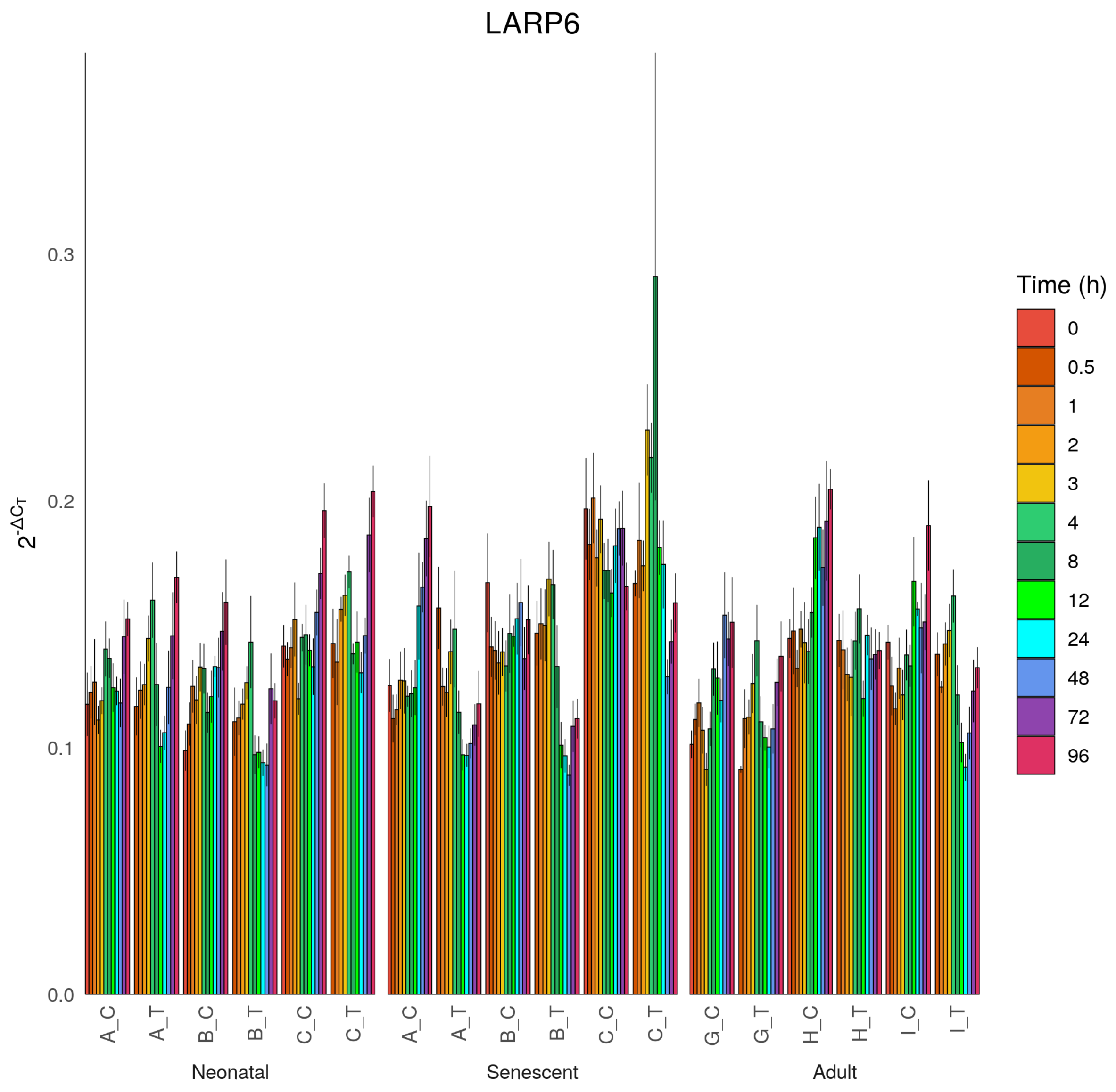
LARP6
Time (h)
0
0.5
1
2
3
4
8
12
24
48
72
96
$2^{-\Delta C_T}$
0.0
0.1
0.2
0.3
A_C
A_T
B_C
B_T
C_C
C_T
G_C
G_T
H_C
H_T
I_C
I_T
Neonatal
Senescent
Adult

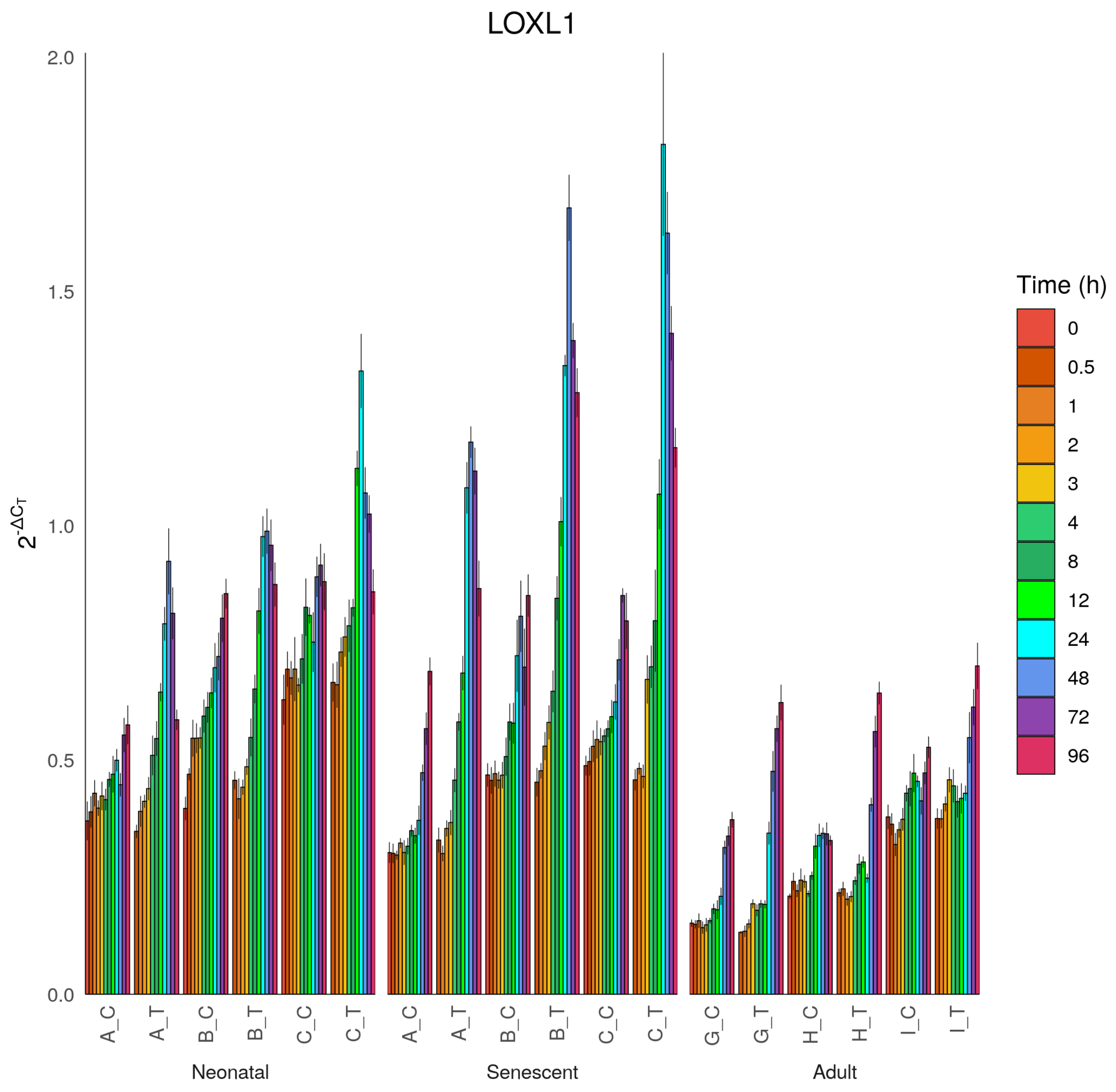
LOXL1
2.0
1.5
1.0
0.5
0.0
$2^{-\Delta C_T}$
A_C
A_T
B_C
B_T
C_C
C_T
G_C
G_T
H_C
H_T
I_C
I_T
Neonatal
Senescent
Adult
Time (h)
0
0.5
1
2
3
4
8
12
24
48
72
96

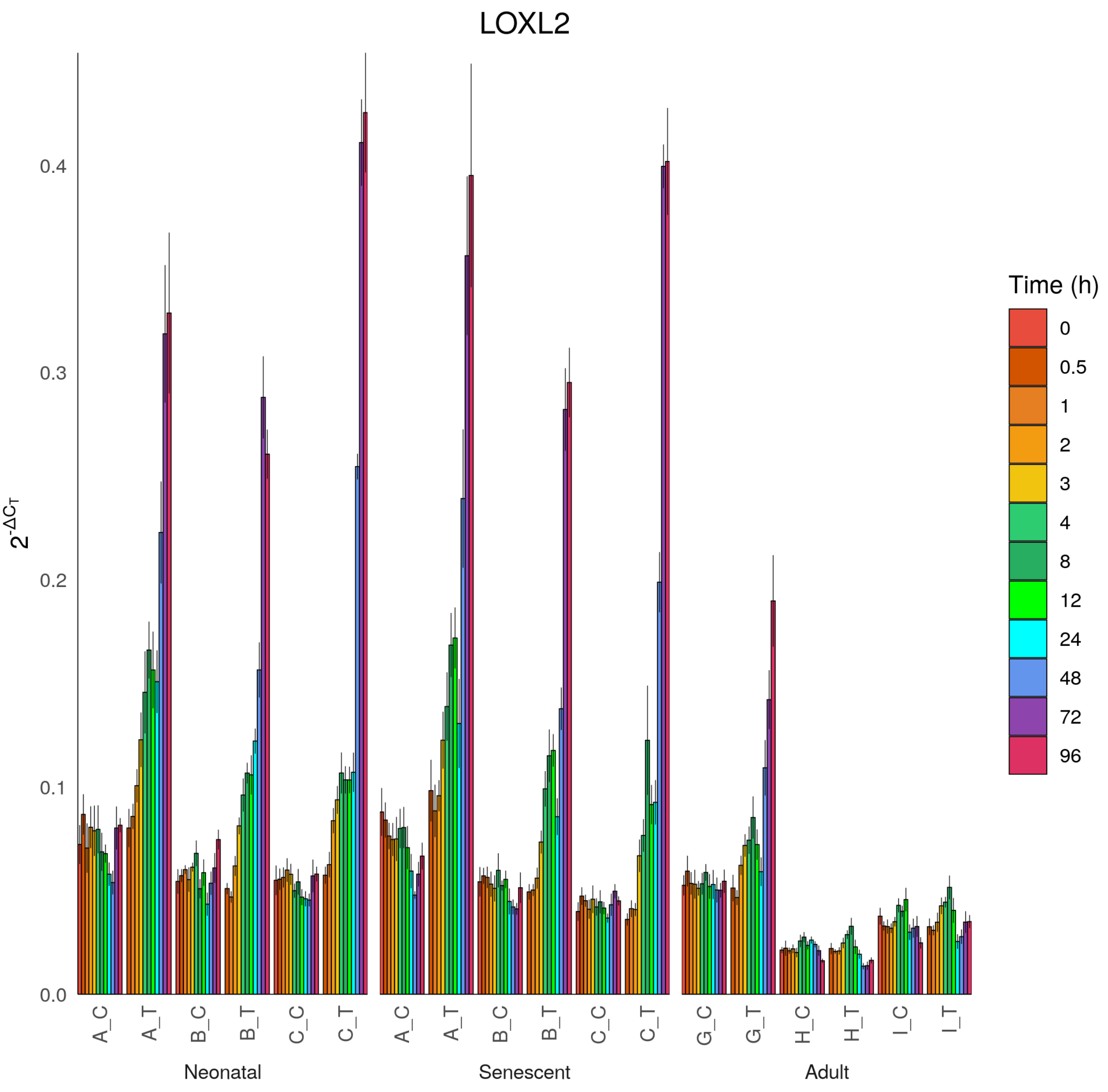

LOXL2
Time (h)
0
0.5
1
2
3
4
8
12
24
48
72
96
$2^{-\Delta C_T}$
0.0
0.1
0.2
0.3
0.4
A_C
A_T
B_C
B_T
C_C
C_T
G_C
G_T
H_C
H_T
I_C
I_T
Neonatal
Senescent
Adult

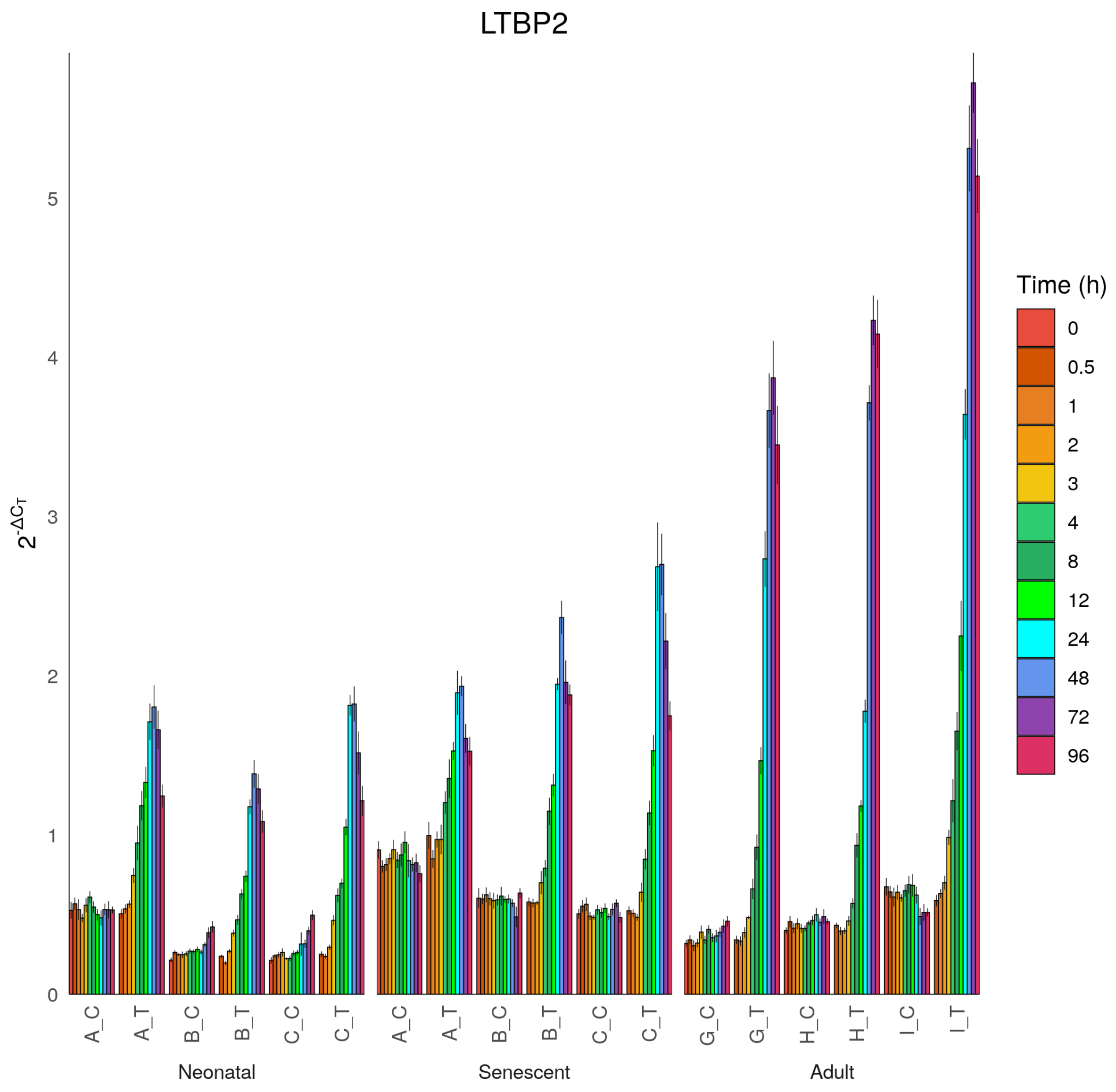

LTBP2
$2^{-\Delta C_T}$
0
1
2
3
4
5
A_C
A_T
B_C
B_T
C_C
C_T
G_C
G_T
H_C
H_T
I_C
I_T
Neonatal
Senescent
Adult
Time (h)
0
0.5
1
2
3
4
8
12
24
48
72
96

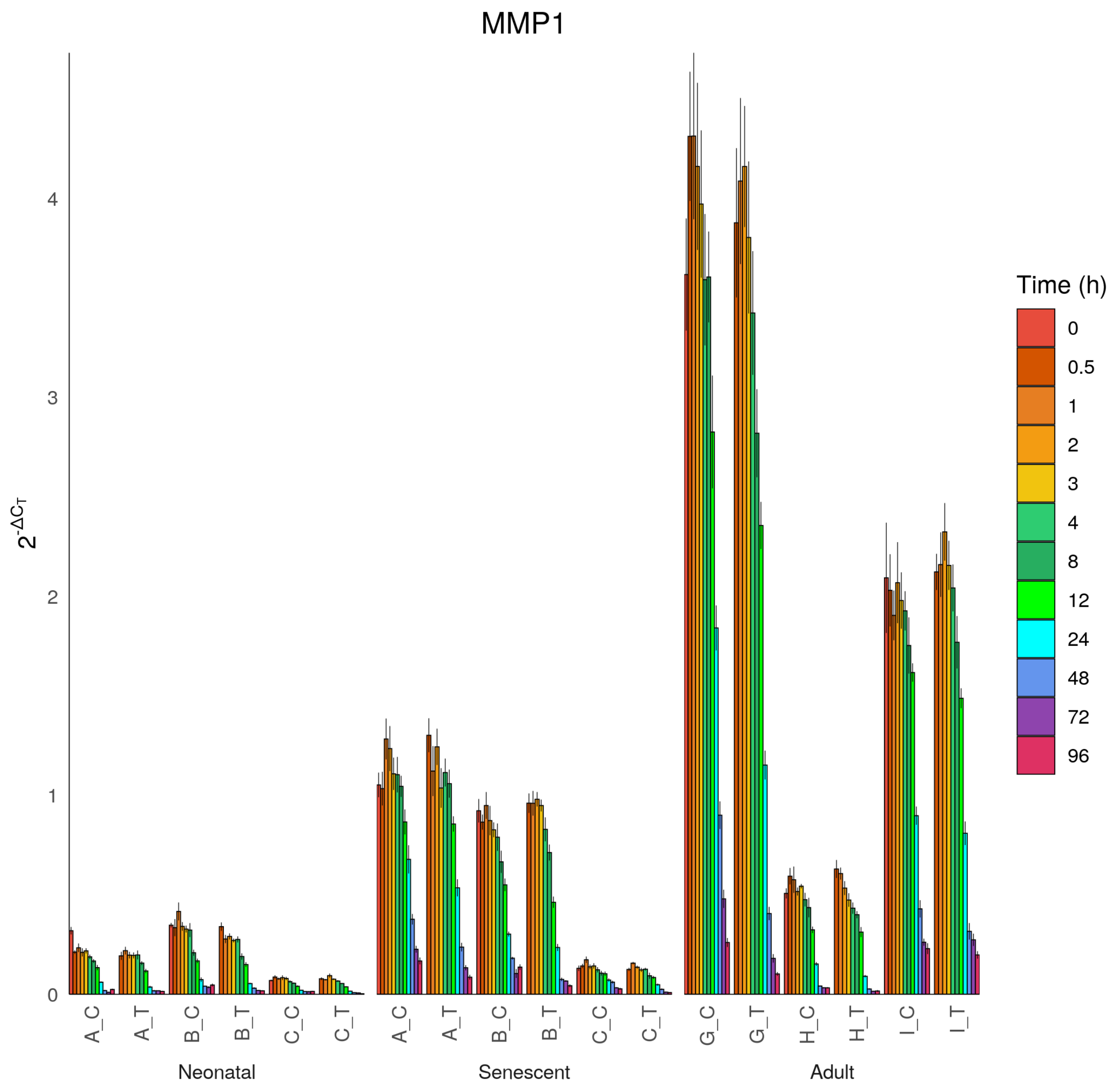

MMP1
$2^{-\Delta C_T}$
0
1
2
3
4
A_C
A_T
B_C
B_T
C_C
C_T
Neonatal
A_C
A_T
B_C
B_T
C_C
C_T
Senescent
G_C
G_T
H_C
H_T
I_C
I_T
Adult
Time (h)
0
0.5
1
2
3
4
8
12
24
48
72
96

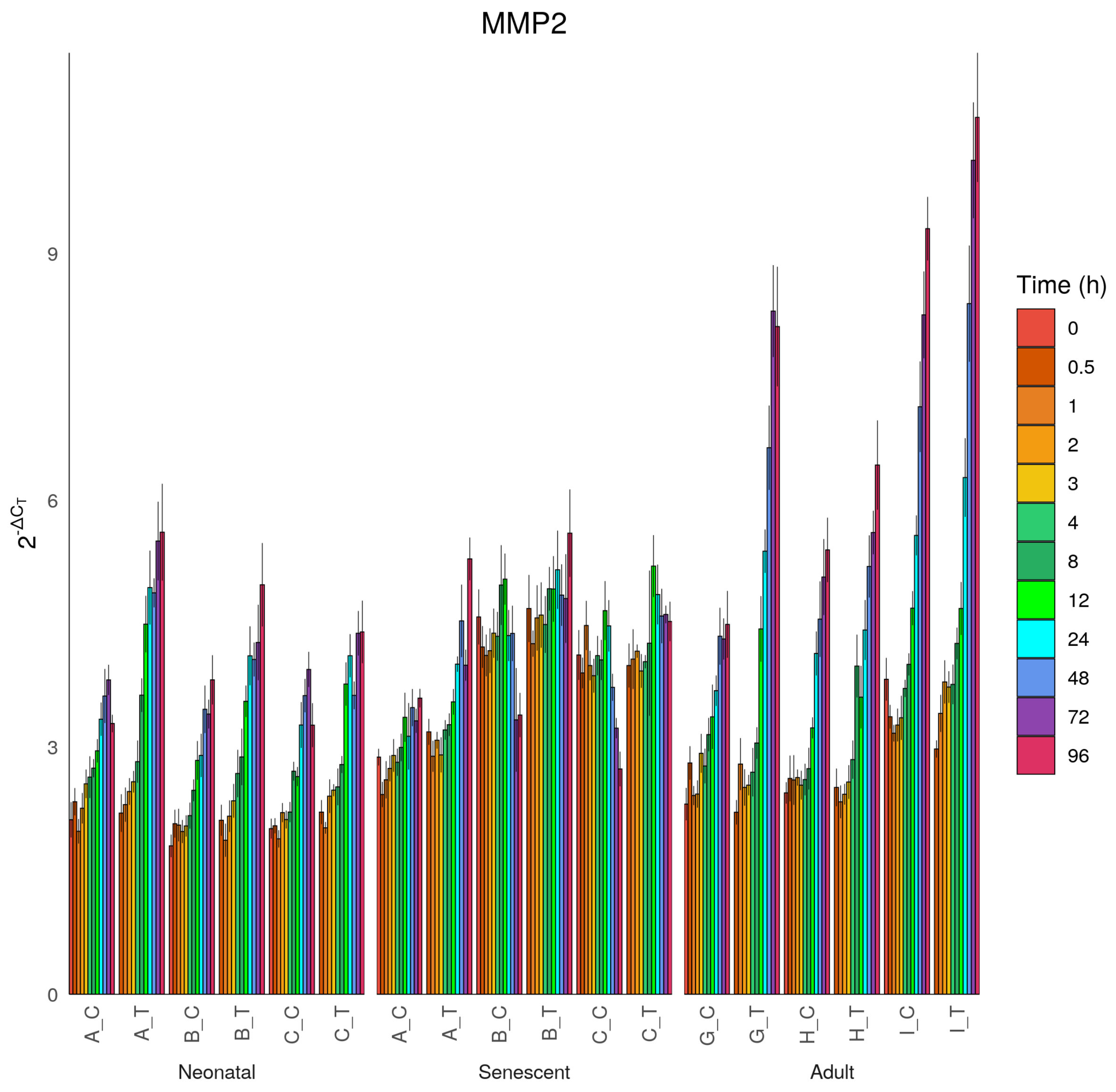
MMP2
$2^{-\Delta C_T}$
0
3
6
9
A_C
A_T
B_C
B_T
C_C
C_T
G_C
G_T
H_C
H_T
I_C
I_T
Neonatal
Senescent
Adult
Time (h)
0
0.5
1
2
3
4
8
12
24
48
72
96

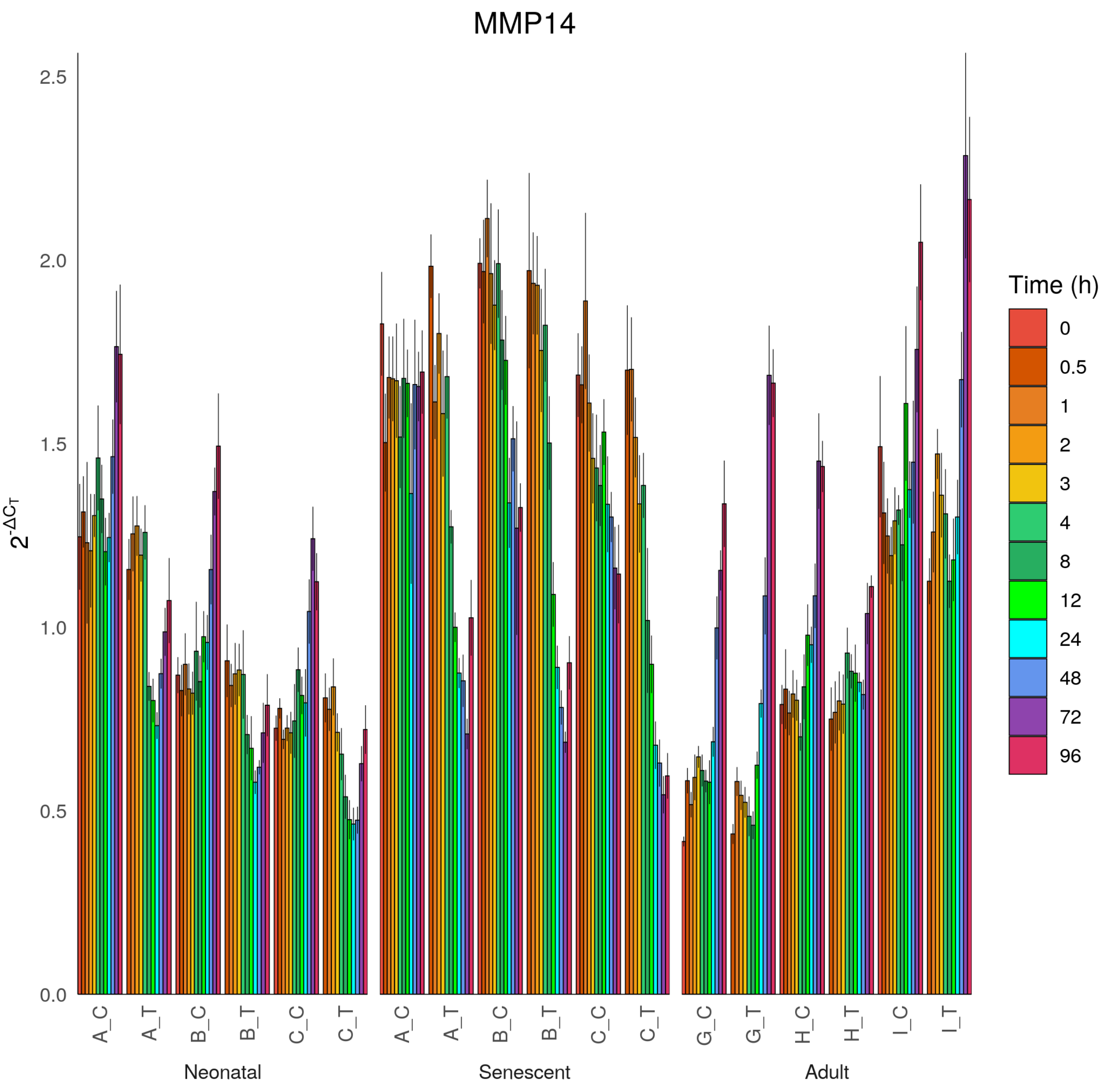
MMP14
$2^{-\Delta C_T}$
2.5
2.0
1.5
1.0
0.5
0.0
A_C
A_T
B_C
B_T
C_C
C_T
G_C
G_T
H_C
H_T
I_C
I_T
Neonatal
Senescent
Adult
Time (h)
0
0.5
1
2
3
4
8
12
24
48
72
96

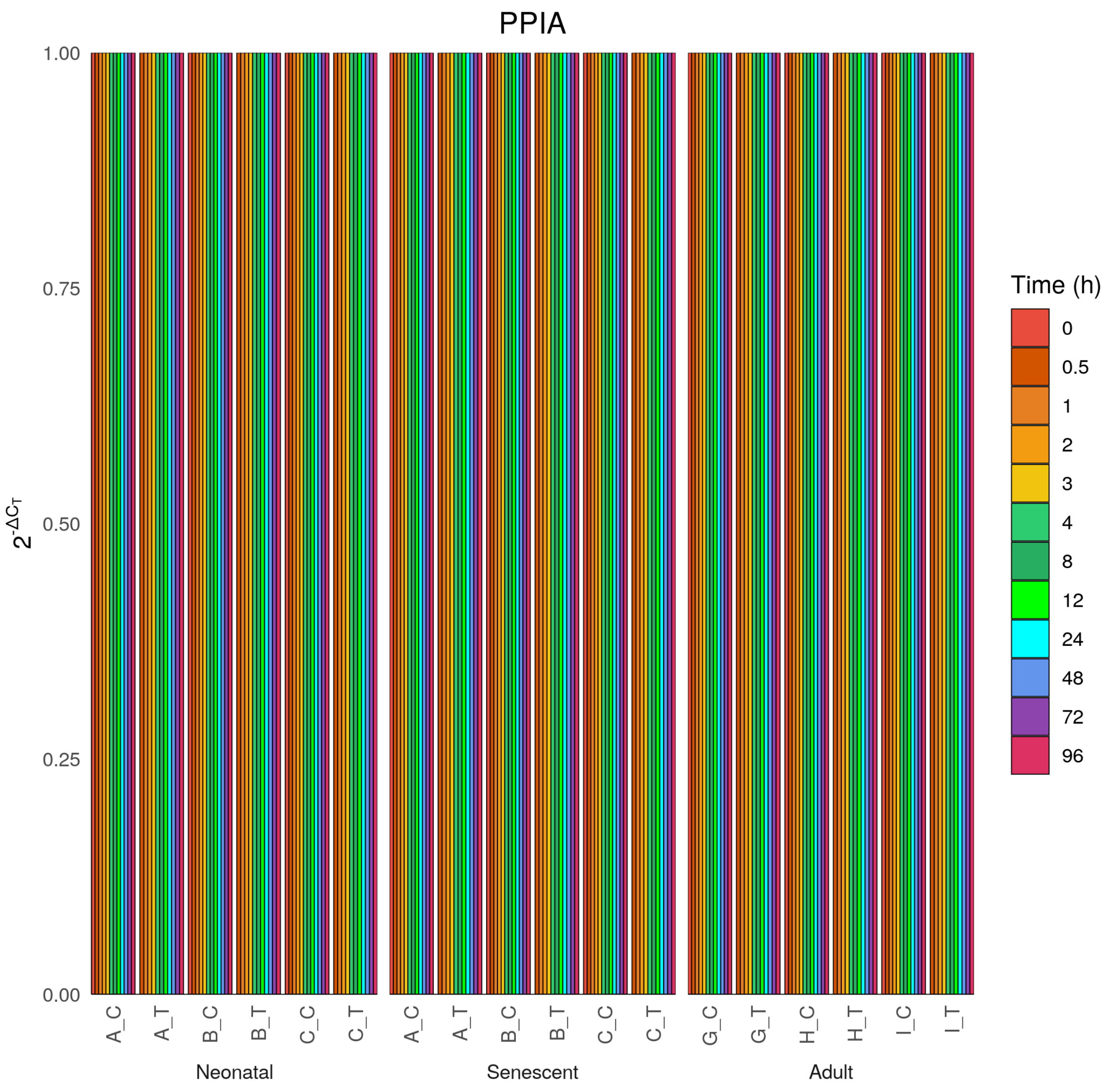

PPIA
$2^{-\Delta C_T}$
1.00
0.75
0.50
0.25
0.00
A_C
A_T
B_C
B_T
C_C
C_T
A_C
A_T
B_C
B_T
C_C
C_T
G_C
G_T
H_C
H_T
I_C
I_T
Neonatal
Senescent
Adult
Time (h)
0
0.5
1
2
3
4
8
12
24
48
72
96

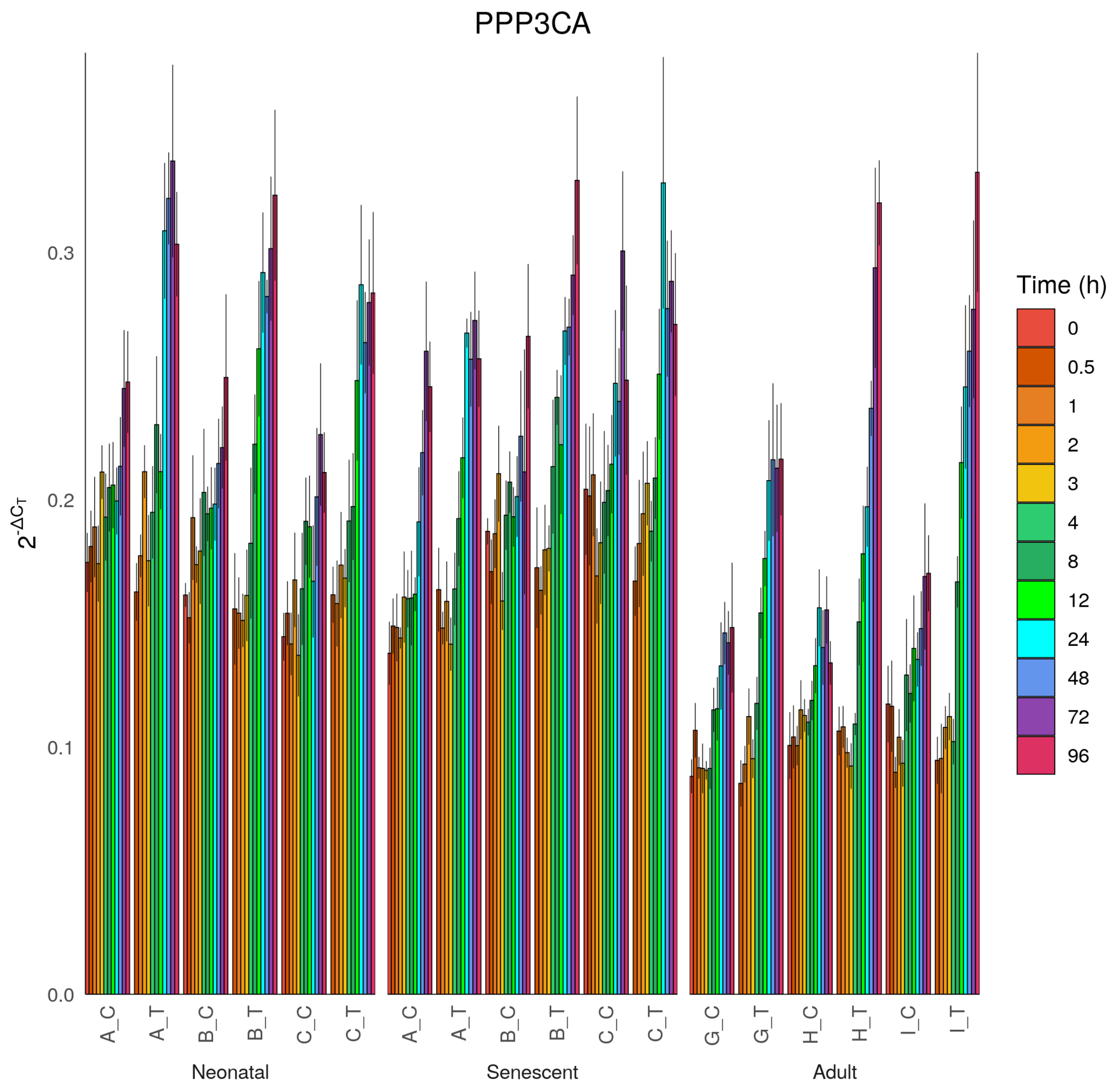
PPP3CA
$2^{-\Delta C_T}$
0.0
0.1
0.2
0.3
A_C
A_T
B_C
B_T
C_C
C_T
G_C
G_T
H_C
H_T
I_C
I_T
Neonatal
Senescent
Adult
Time (h)
0
0.5
1
2
3
4
8
12
24
48
72
96

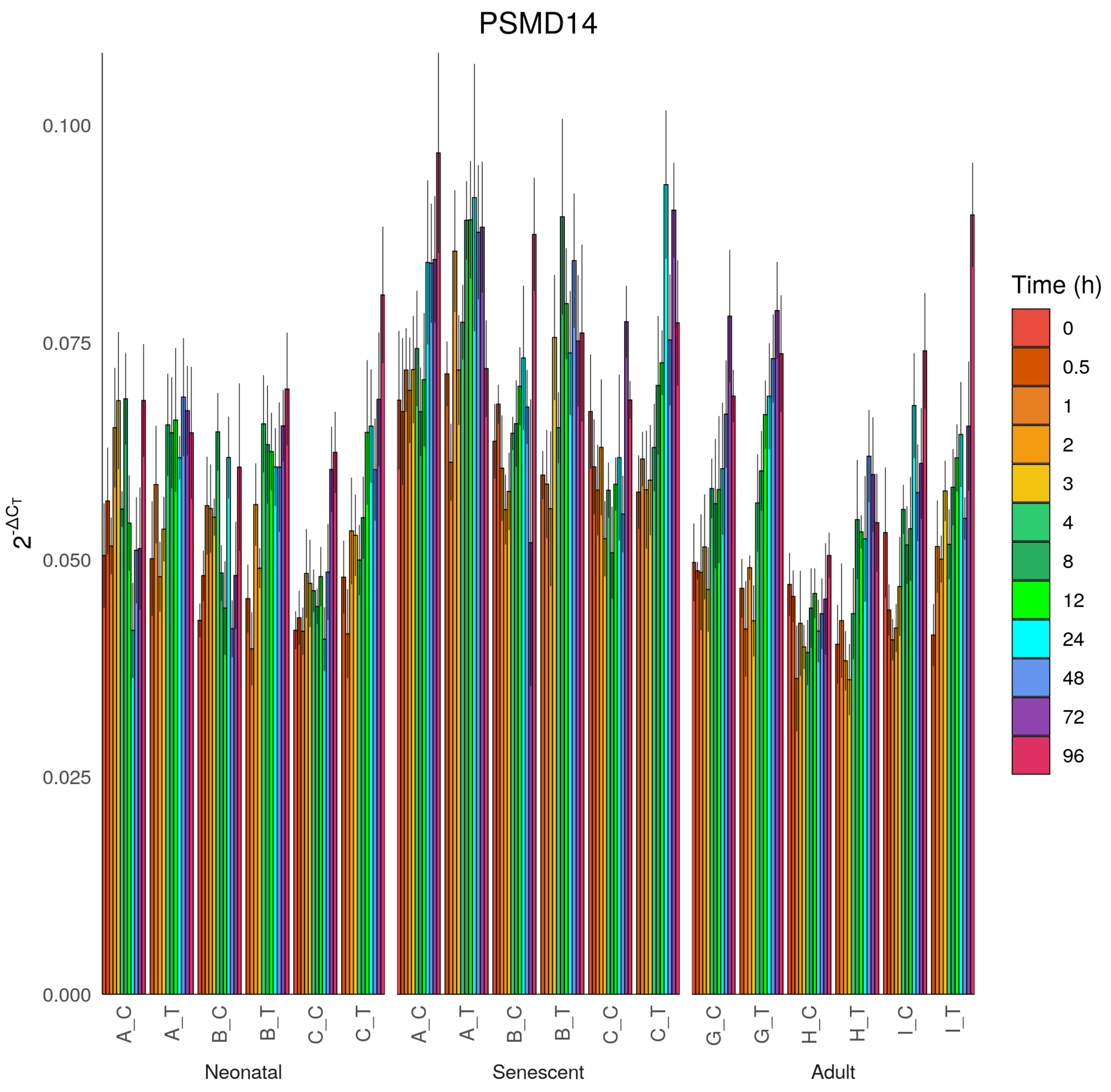
PSMD14
0.100
0.075
0.050
0.025
0.000
$2^{-\Delta C_T}$
A_C
A_T
B_C
B_T
C_C
C_T
A_C
A_T
B_C
B_T
C_C
C_T
G_C
G_T
H_C
H_T
I_C
I_T
Neonatal
Senescent
Adult
Time (h)
0
0.5
1
2
3
4
8
12
24
48
72
96

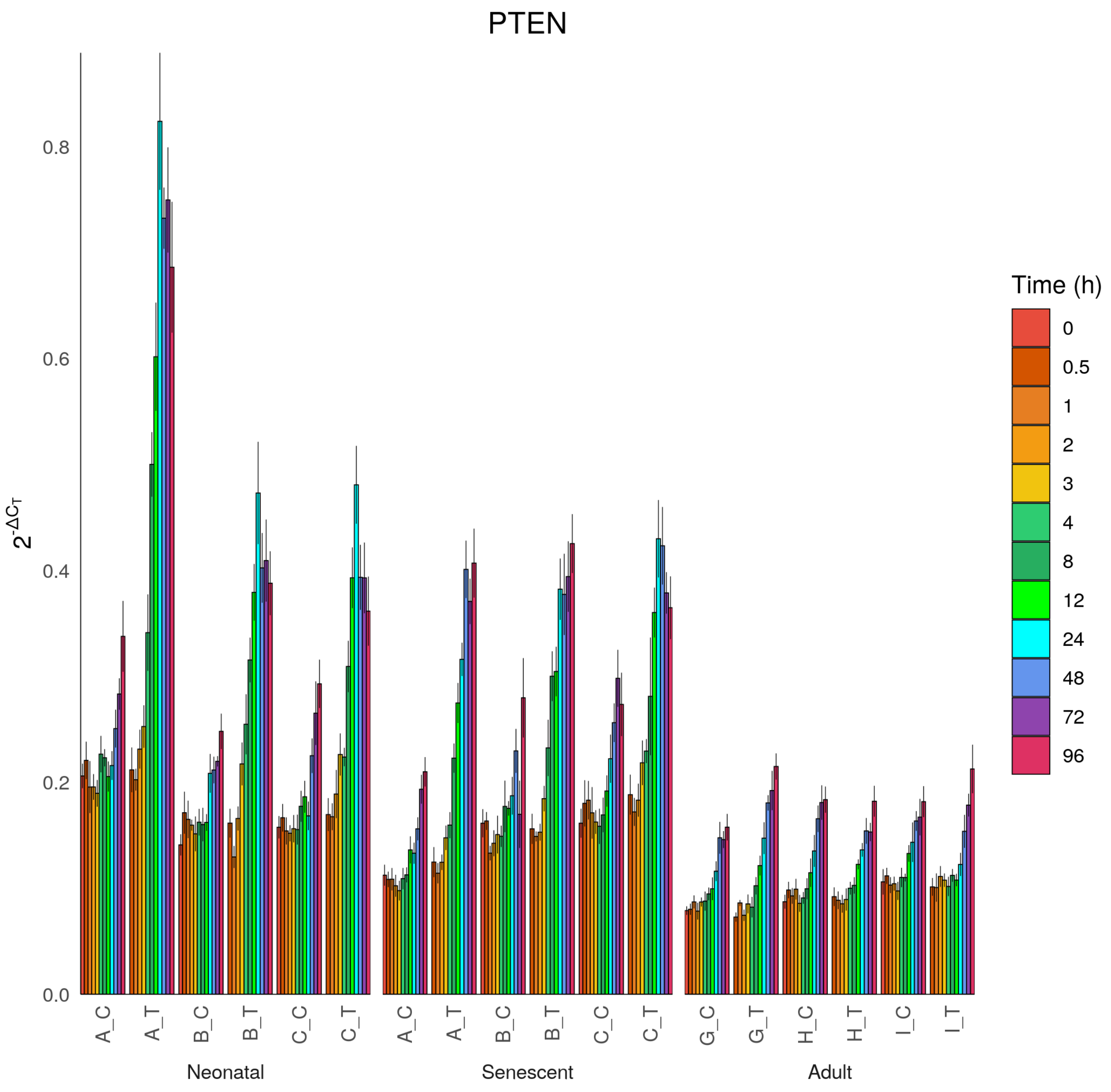

PTEN
$2^{-\Delta C_T}$
0.0
0.2
0.4
0.6
0.8
A_C
A_T
B_C
B_T
C_C
C_T
G_C
G_T
H_C
H_T
I_C
I_T
Neonatal
Senescent
Adult
Time (h)
0
0.5
1
2
3
4
8
12
24
48
72
96

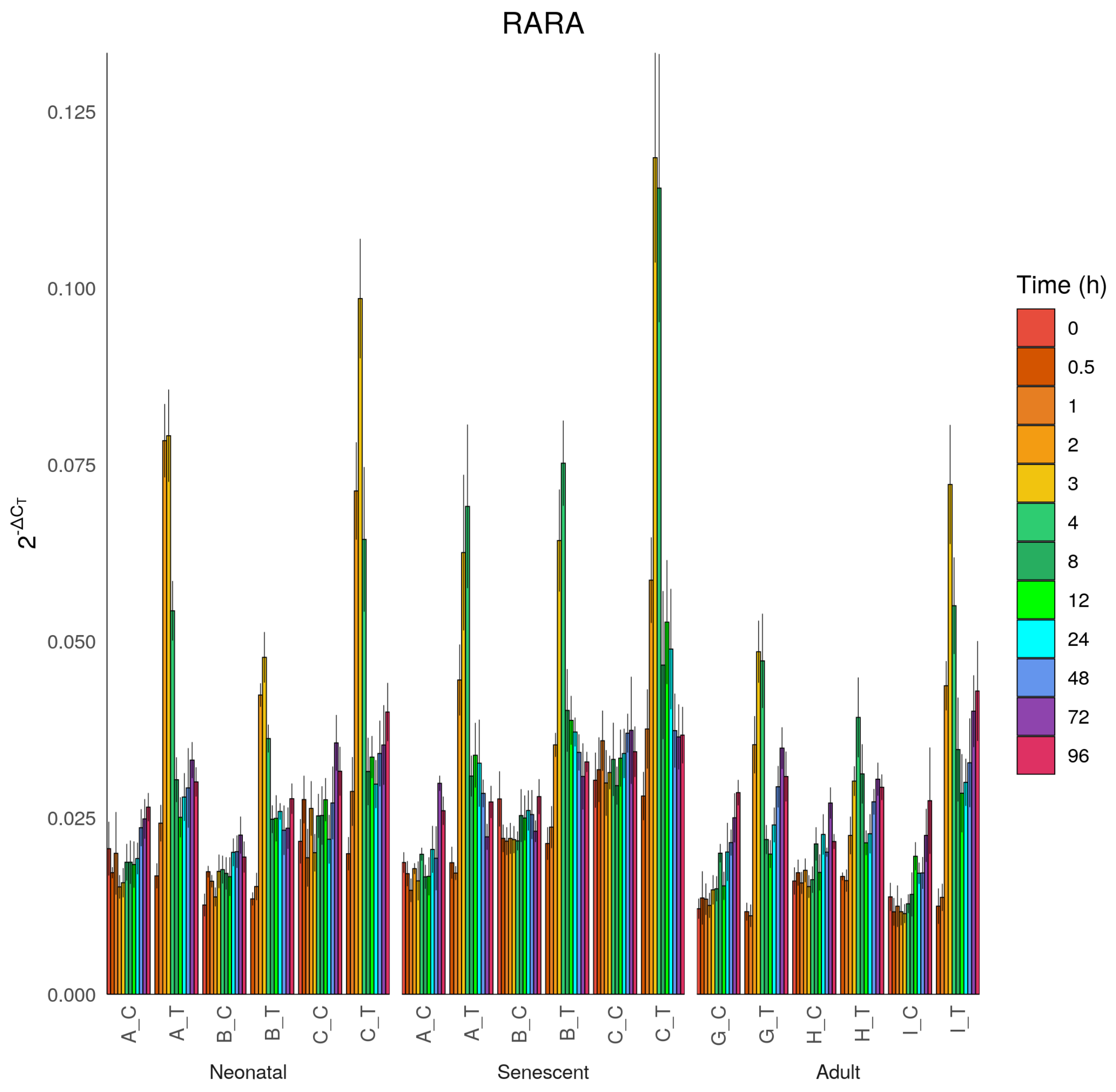

RARA
$2^{-\Delta C_T}$
0.000
0.025
0.050
0.075
0.100
0.125
A_C
A_T
B_C
B_T
C_C
C_T
G_C
G_T
H_C
H_T
I_C
I_T
Neonatal
Senescent
Adult
Time (h)
0
0.5
1
2
3
4
8
12
24
48
72
96

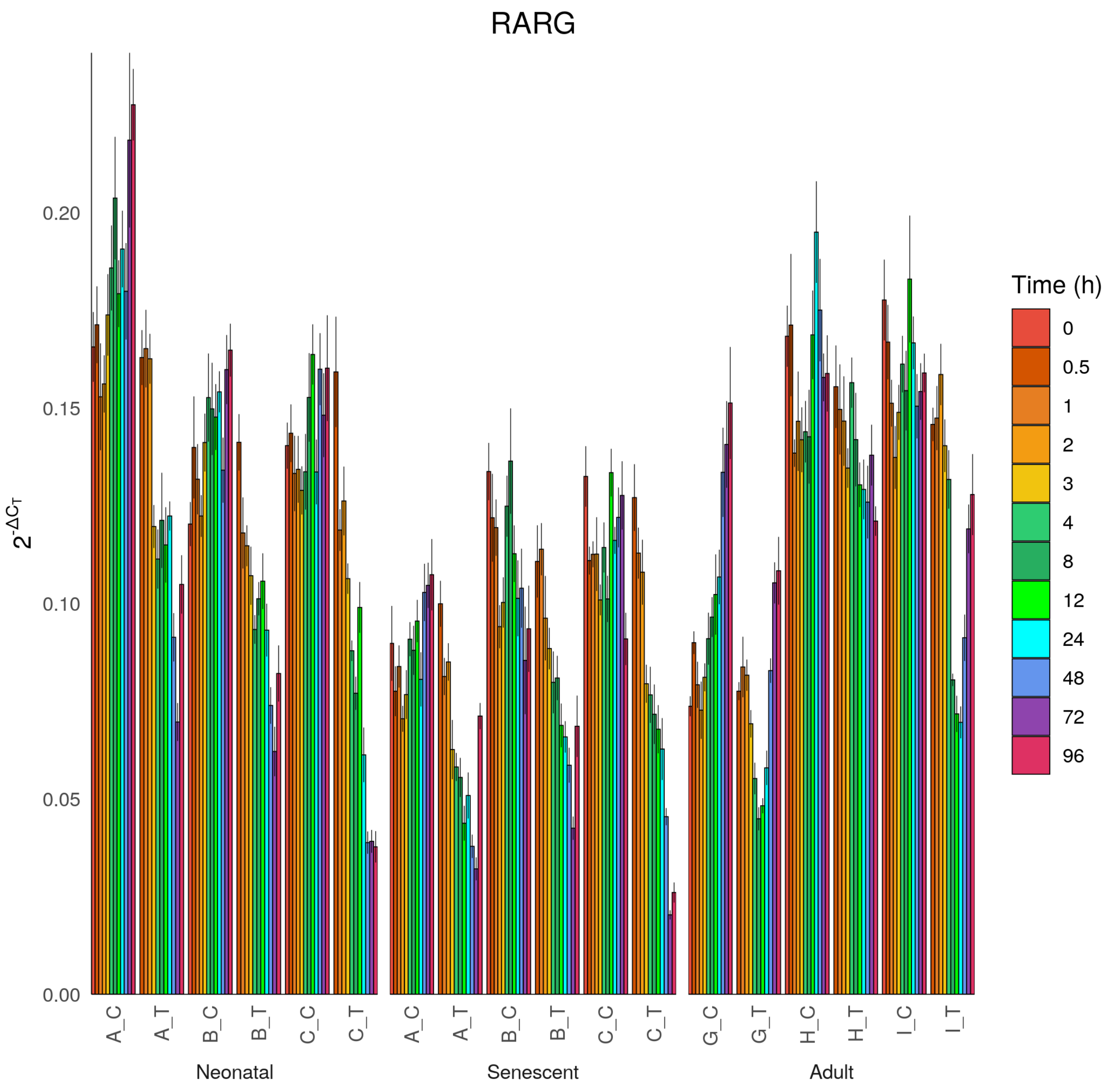
RARG
$2^{-\Delta C_T}$
0.00
0.05
0.10
0.15
0.20
A_C
A_T
B_C
B_T
C_C
C_T
Neonatal
A_C
A_T
B_C
B_T
C_C
C_T
Senescent
G_C
G_T
H_C
H_T
I_C
I_T
Adult
Time (h)
0
0.5
1
2
3
4
8
12
24
48
72
96

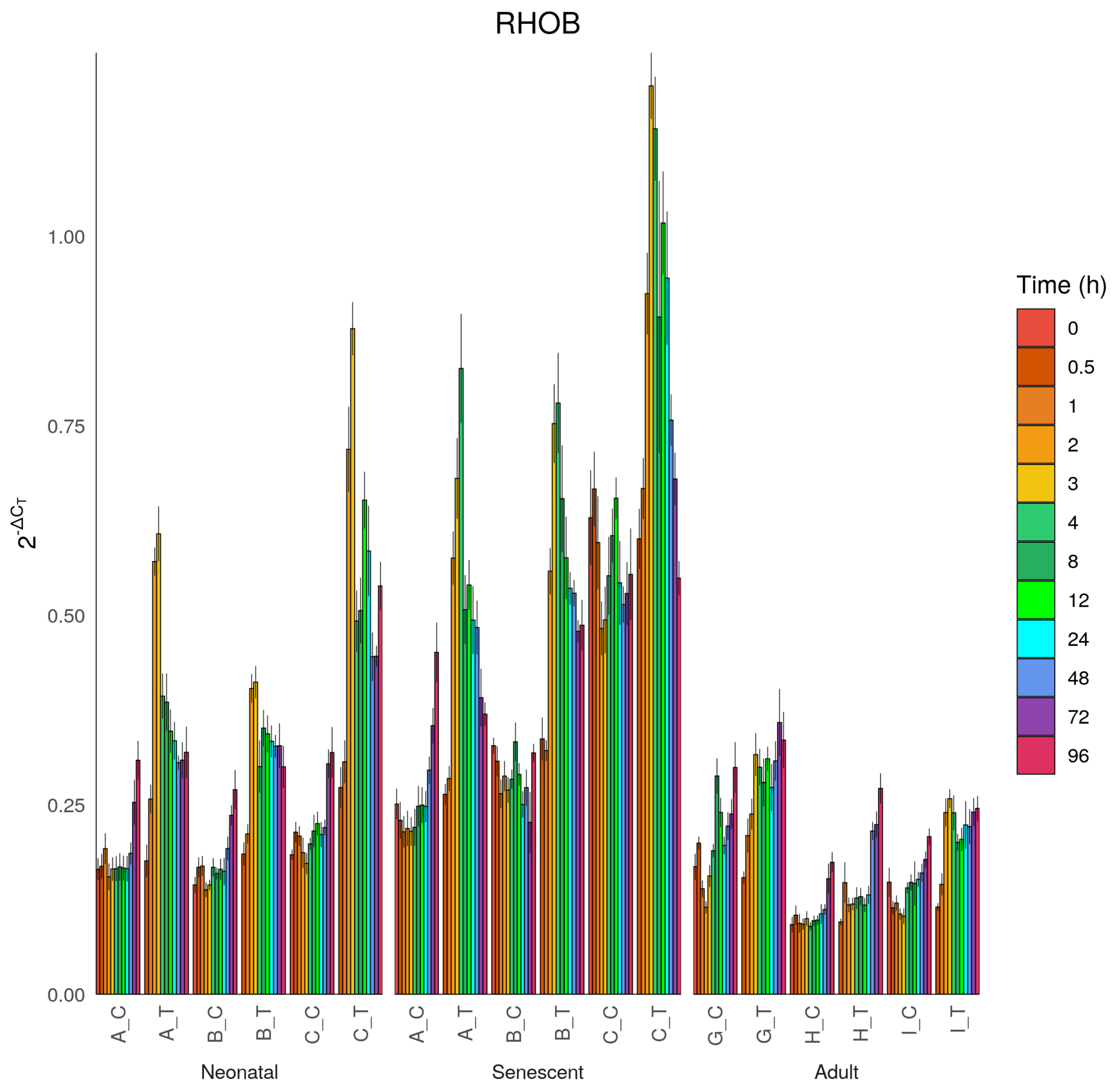
RHOB
Time (h)
0
0.5
1
2
3
4
8
12
24
48
72
96
1.00
0.75
0.50
0.25
0.00
A_C
A_T
B_C
B_T
C_C
C_T
A_C
A_T
B_C
B_T
C_C
C_T
G_C
G_T
H_C
H_T
I_C
I_T
Neonatal
Senescent
Adult

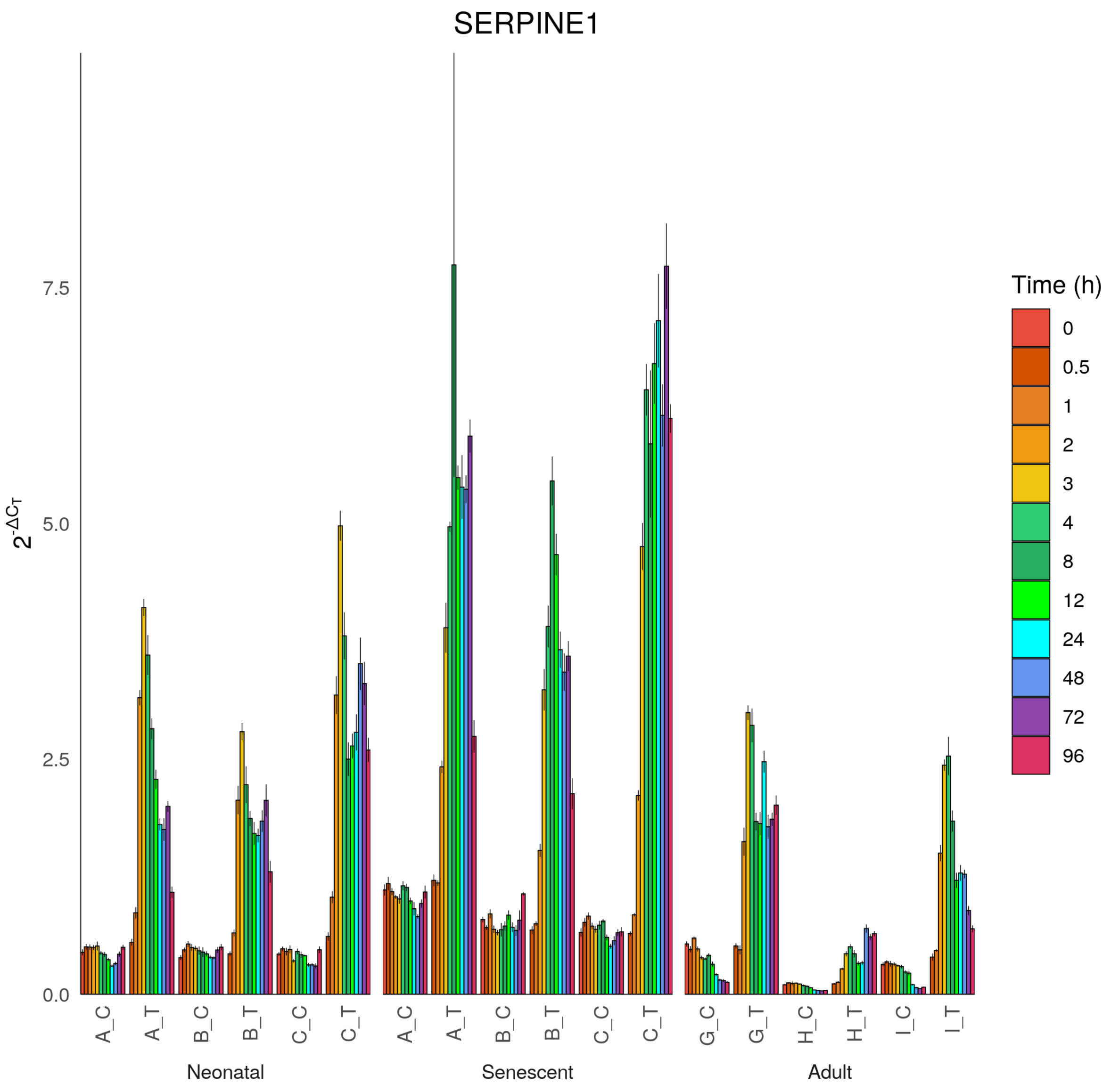
SERPINE1
$2^{-\Delta C_T}$
0.0
2.5
5.0
7.5
A_C
A_T
B_C
B_T
C_C
C_T
A_C
A_T
B_C
B_T
C_C
C_T
G_C
G_T
H_C
H_T
I_C
I_T
Neonatal
Senescent
Adult
Time (h)
0
0.5
1
2
3
4
8
12
24
48
72
96

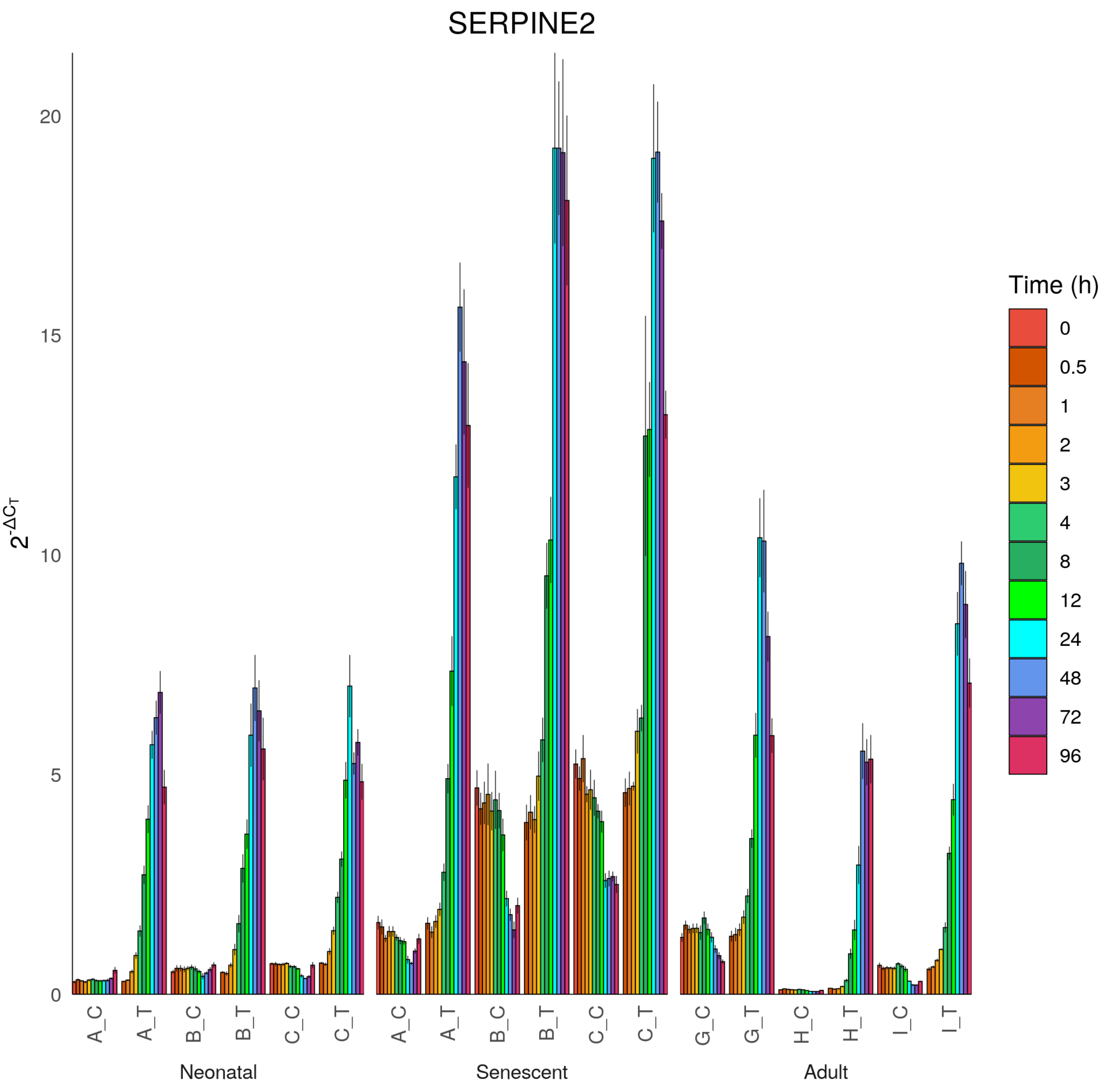
SERPINE2
$2^{-\Delta C_T}$
0
5
10
15
20
A_C
A_T
B_C
B_T
C_C
C_T
G_C
G_T
H_C
H_T
I_C
I_T
Neonatal
Senescent
Adult
Time (h)
0
0.5
1
2
3
4
8
12
24
48
72
96

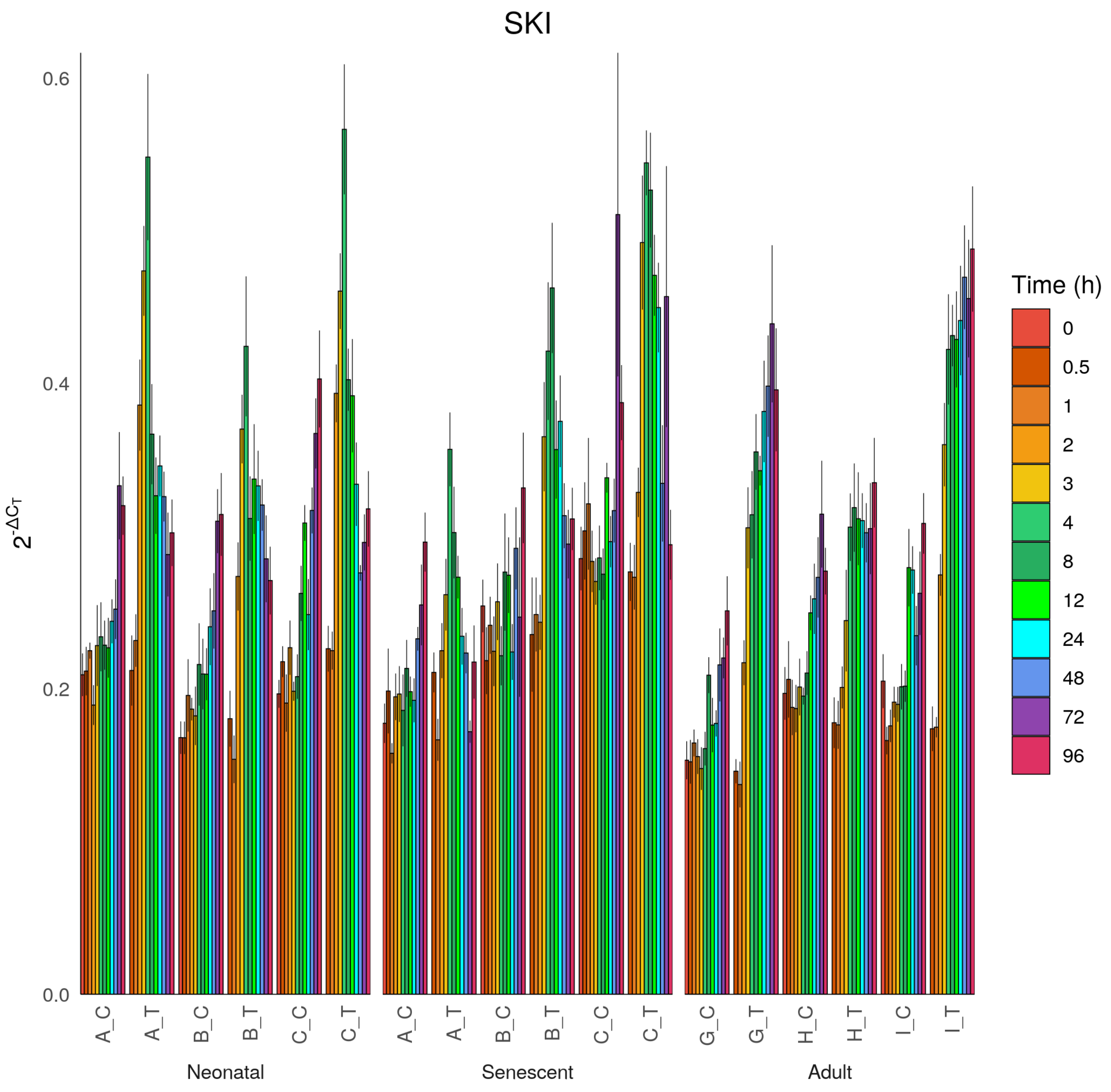

SKI
$2^{-\Delta C_T}$
0.6
0.4
0.2
0.0
A_C
A_T
B_C
B_T
C_C
C_T
G_C
G_T
H_C
H_T
I_C
I_T
Neonatal
Senescent
Adult
Time (h)
0
0.5
1
2
3
4
8
12
24
48
72
96

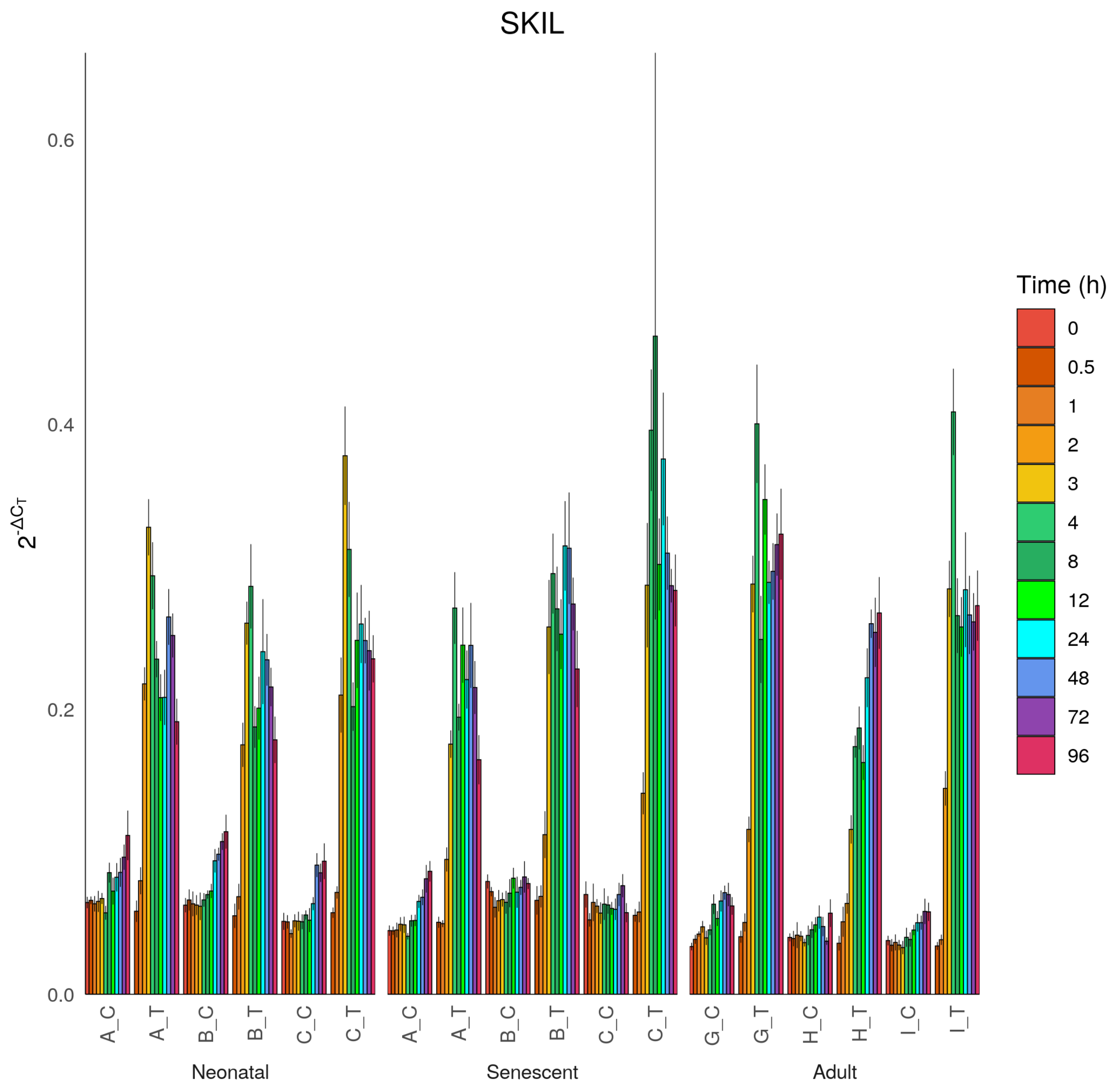
SKIL
0.0
0.2
0.4
0.6
$2^{-\Delta C_T}$
A_C
A_T
B_C
B_T
C_C
C_T
Neonatal
A_C
A_T
B_C
B_T
C_C
C_T
Senescent
G_C
G_T
H_C
H_T
I_C
I_T
Adult
Time (h)
0
0.5
1
2
3
4
8
12
24
48
72
96

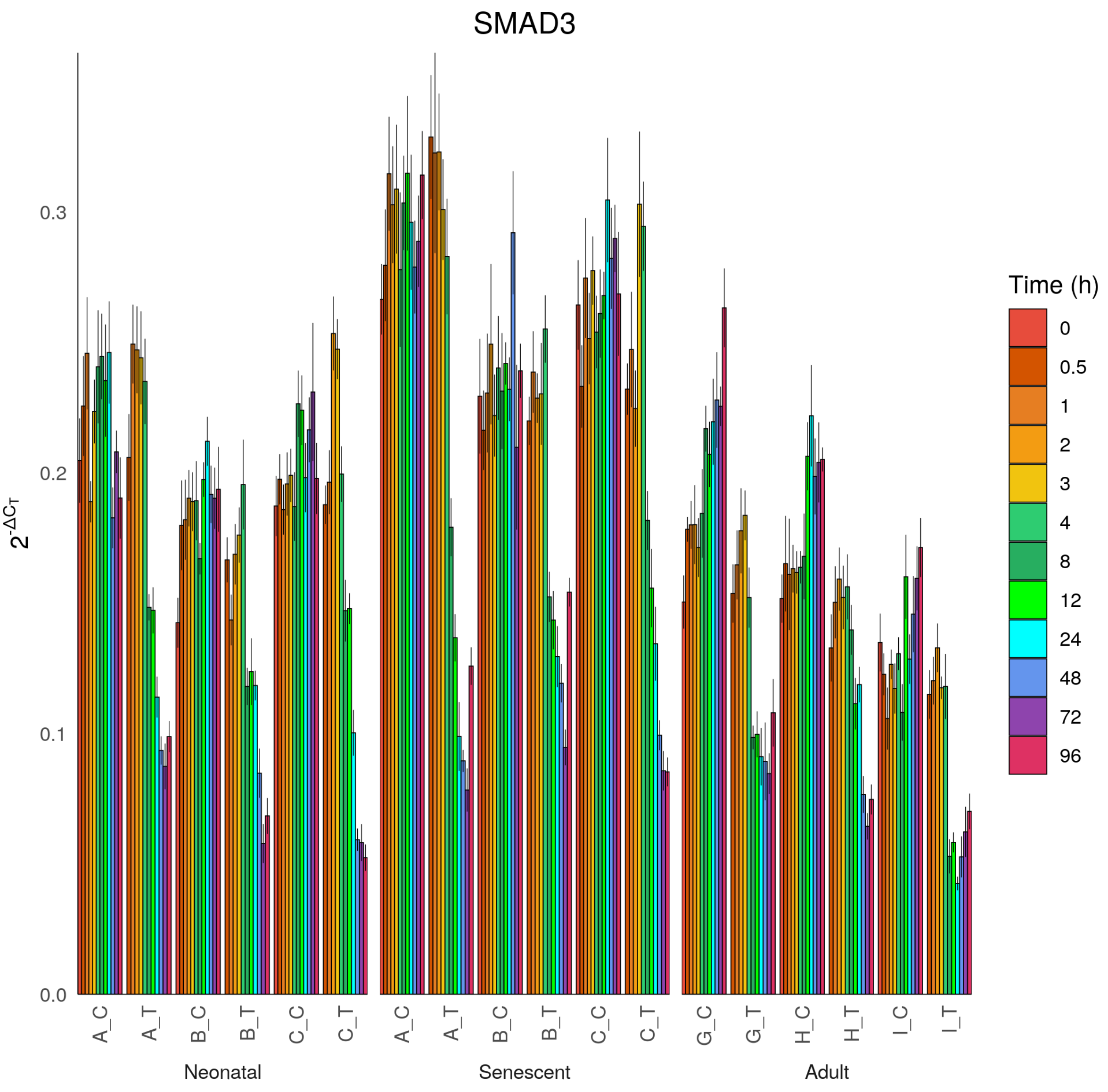
SMAD3
$2^{-\Delta C_T}$
0.0
0.1
0.2
0.3
A_C
A_T
B_C
B_T
C_C
C_T
A_C
A_T
B_C
B_T
C_C
C_T
G_C
G_T
H_C
H_T
I_C
I_T
Neonatal
Senescent
Adult
Time (h)
0
0.5
1
2
3
4
8
12
24
48
72
96

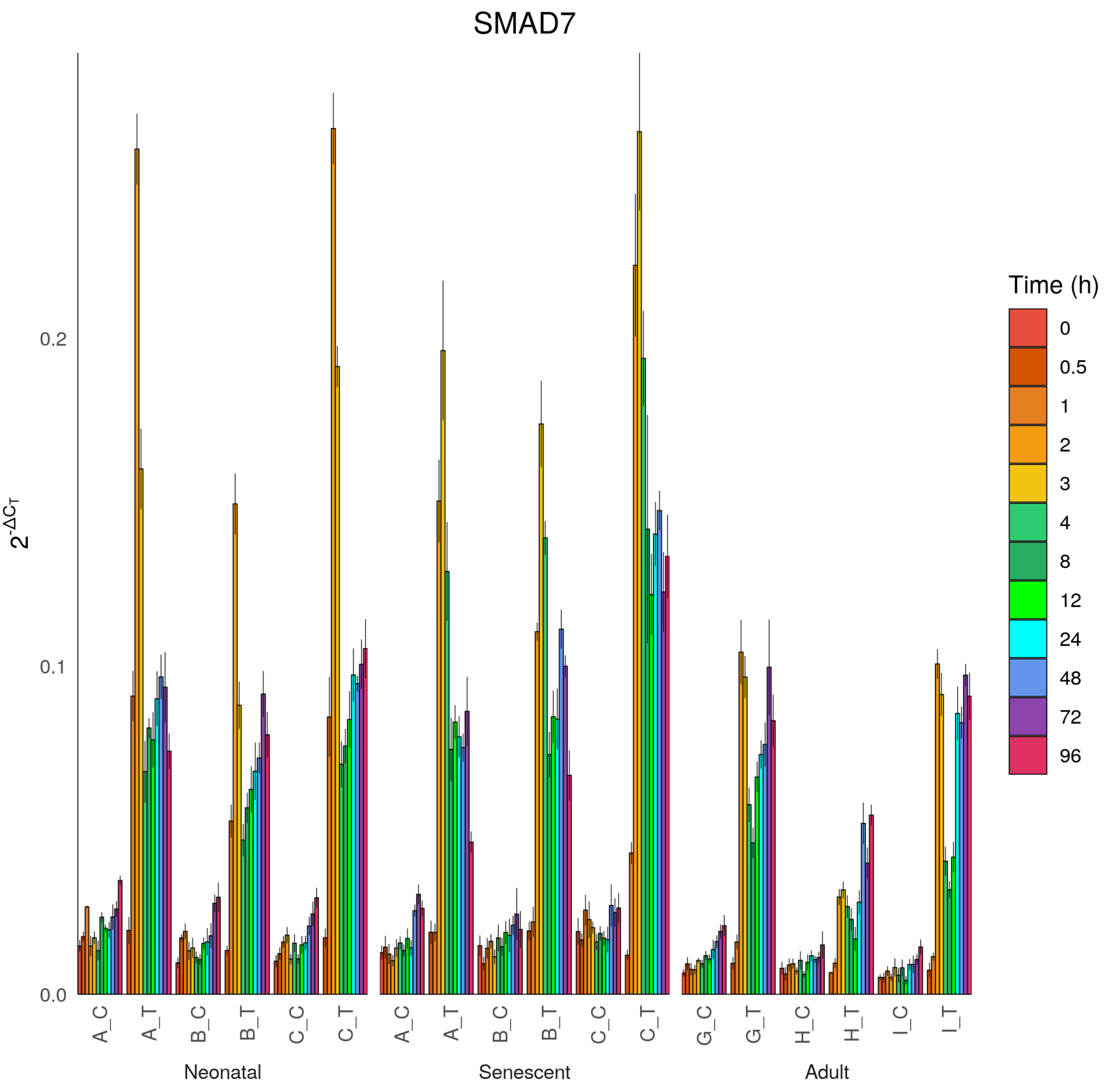

SMAD7
2^-ΔCT
0.0
0.1
0.2
A_C
A_T
B_C
B_T
C_C
C_T
G_C
G_T
H_C
H_T
I_C
I_T
Neonatal
Senescent
Adult
Time (h)
0
0.5
1
2
3
4
8
12
24
48
72
96

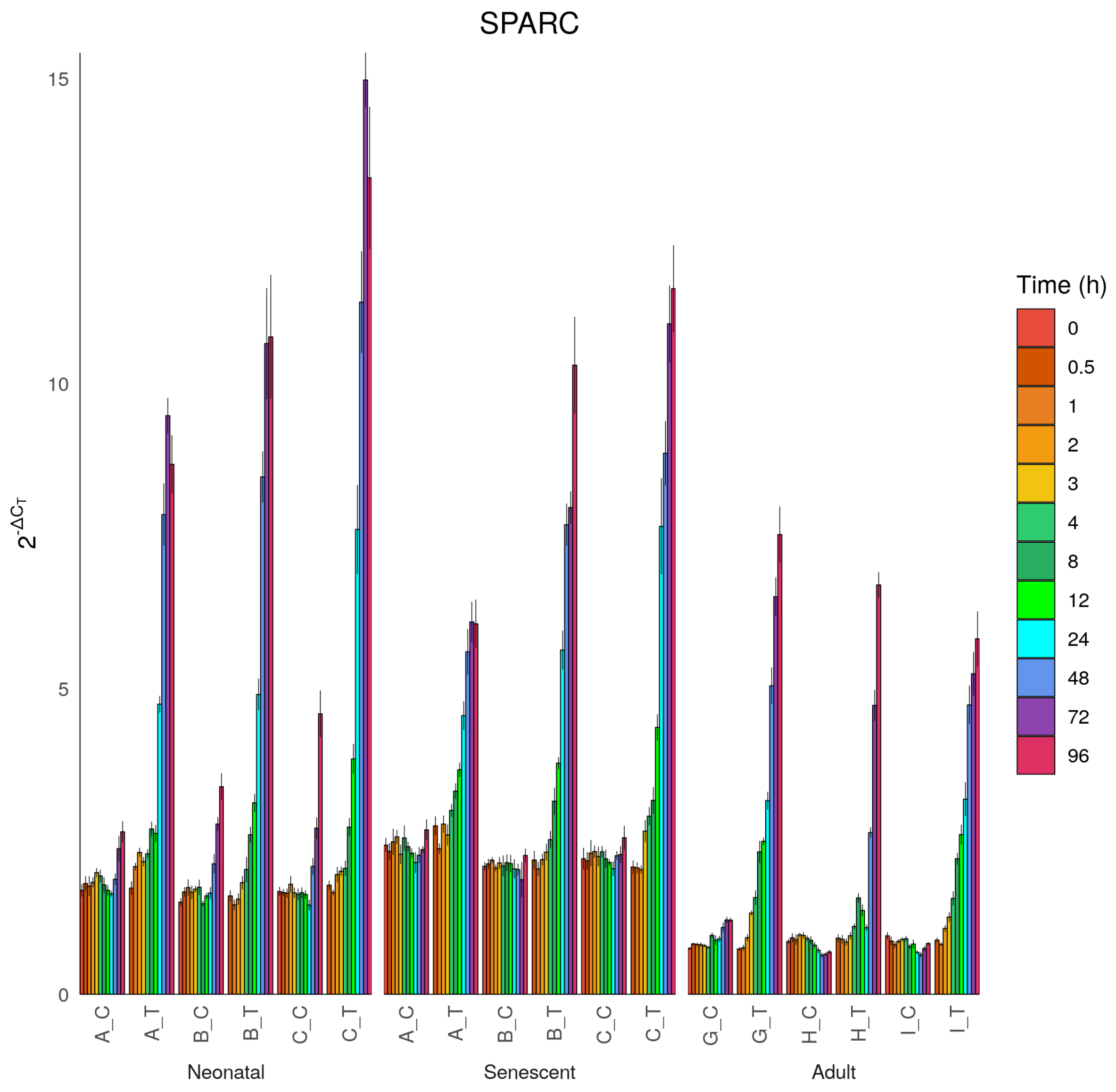

SPARC
$2^{-\Delta C_T}$
0
5
10
15
A_C
A_T
B_C
B_T
C_C
C_T
G_C
G_T
H_C
H_T
I_C
I_T
Neonatal
Senescent
Adult
Time (h)
0
0.5
1
2
3
4
8
12
24
48
72
96

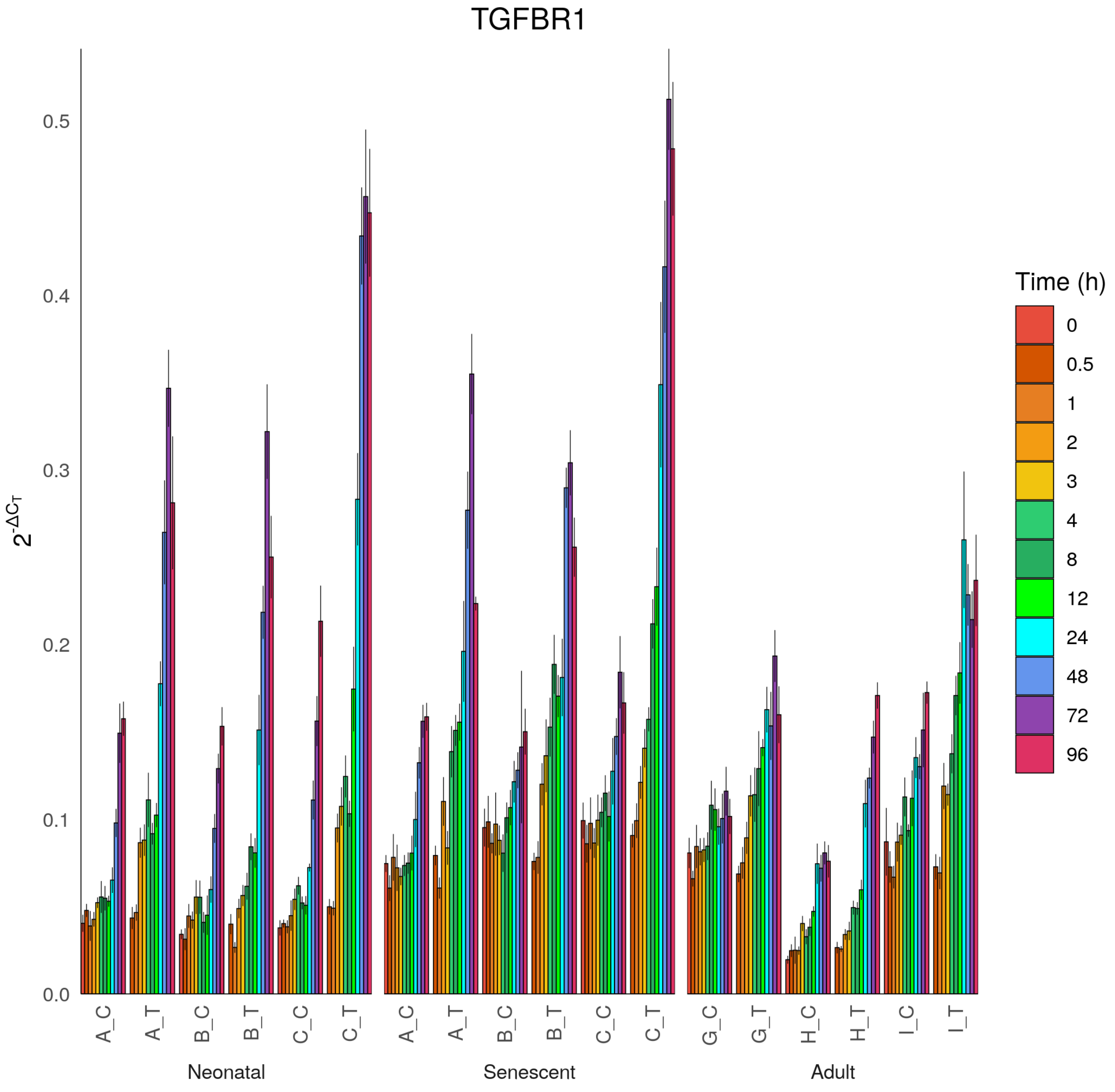

TGFBR1
$2^{-\Delta C_T}$
0.0
0.1
0.2
0.3
0.4
0.5
A_C
A_T
B_C
B_T
C_C
C_T
G_C
G_T
H_C
H_T
I_C
I_T
Neonatal
Senescent
Adult
Time (h)
0
0.5
1
2
3
4
8
12
24
48
72
96

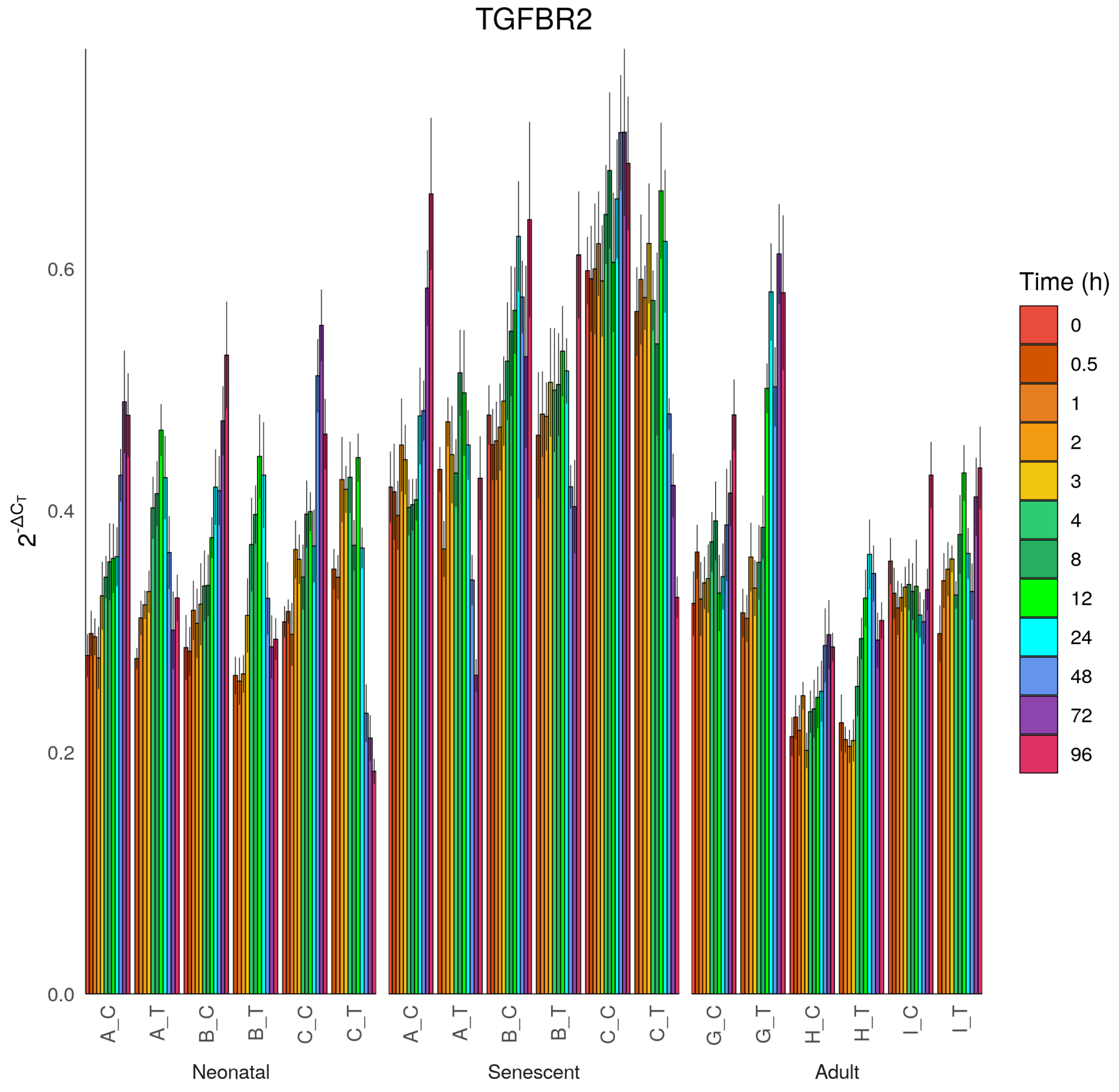
TGFBR2
$2^{-\Delta C_T}$
0.0
0.2
0.4
0.6
A_C
A_T
B_C
B_T
C_C
C_T
Neonatal
Senescent
G_C
G_T
H_C
H_T
I_C
I_T
Adult
Time (h)
0
0.5
1
2
3
4
8
12
24
48
72
96

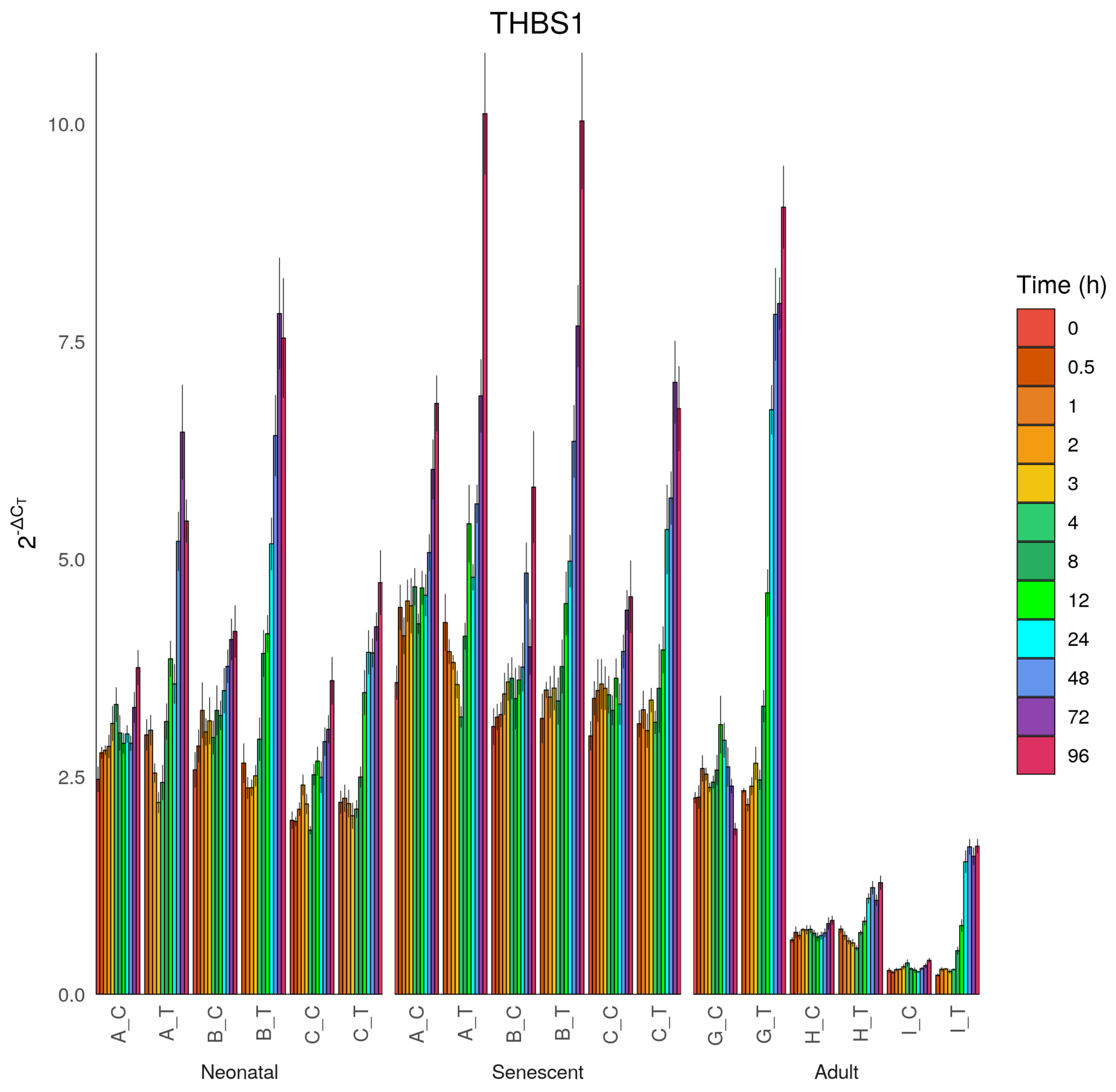
THBS1
$2^{-\Delta C_T}$
0.0
2.5
5.0
7.5
10.0
A_C
A_T
B_C
B_T
C_C
C_T
G_C
G_T
H_C
H_T
I_C
I_T
Neonatal
Senescent
Adult
Time (h)
0
0.5
1
2
3
4
8
12
24
48
72
96

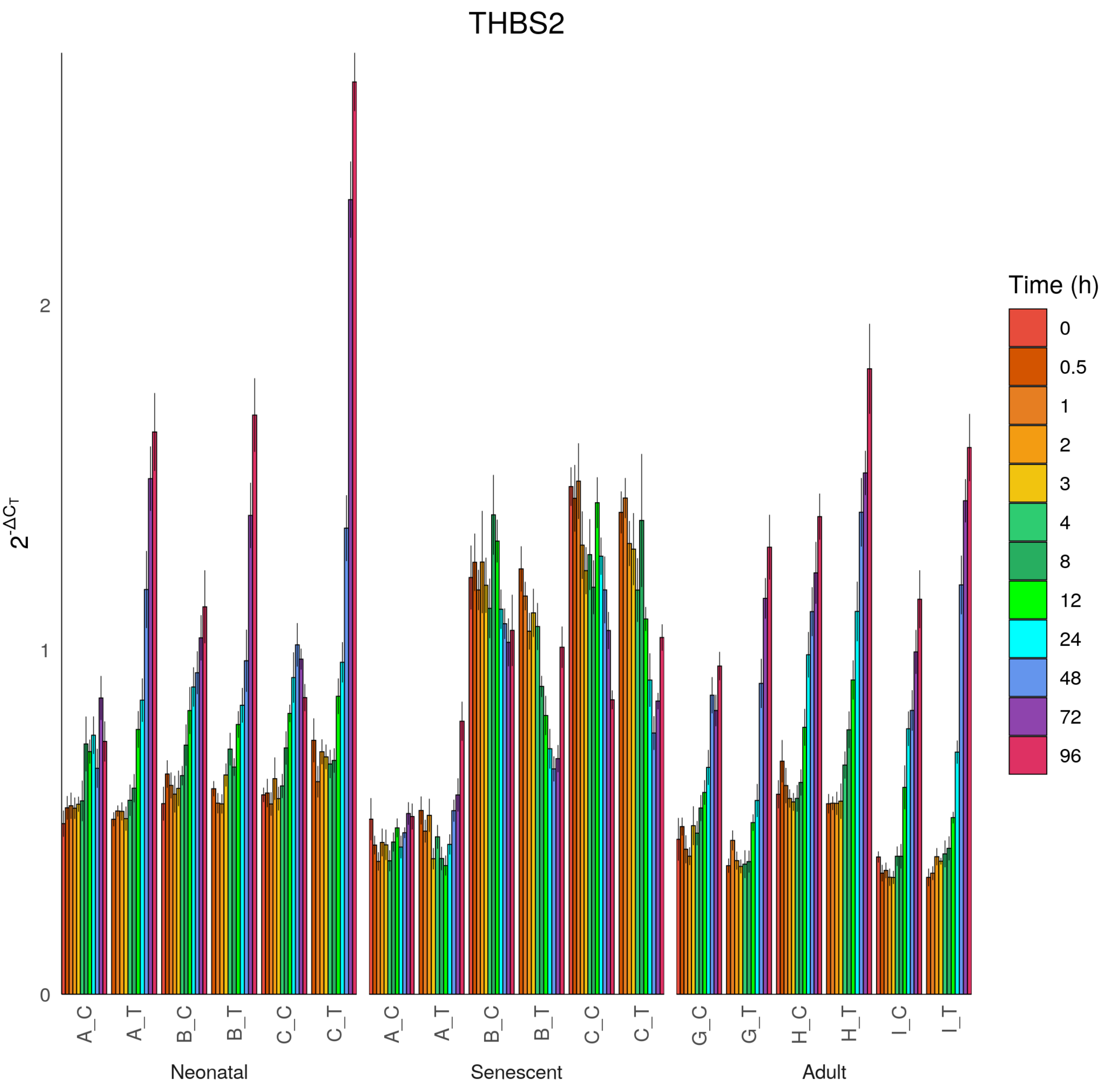
THBS2
$2^{-\Delta C_T}$
0
1
2
A_C
A_T
B_C
B_T
C_C
C_T
A_C
A_T
B_C
B_T
C_C
C_T
G_C
G_T
H_C
H_T
I_C
I_T
Neonatal
Senescent
Adult
Time (h)
0
0.5
1
2
3
4
8
12
24
48
72
96

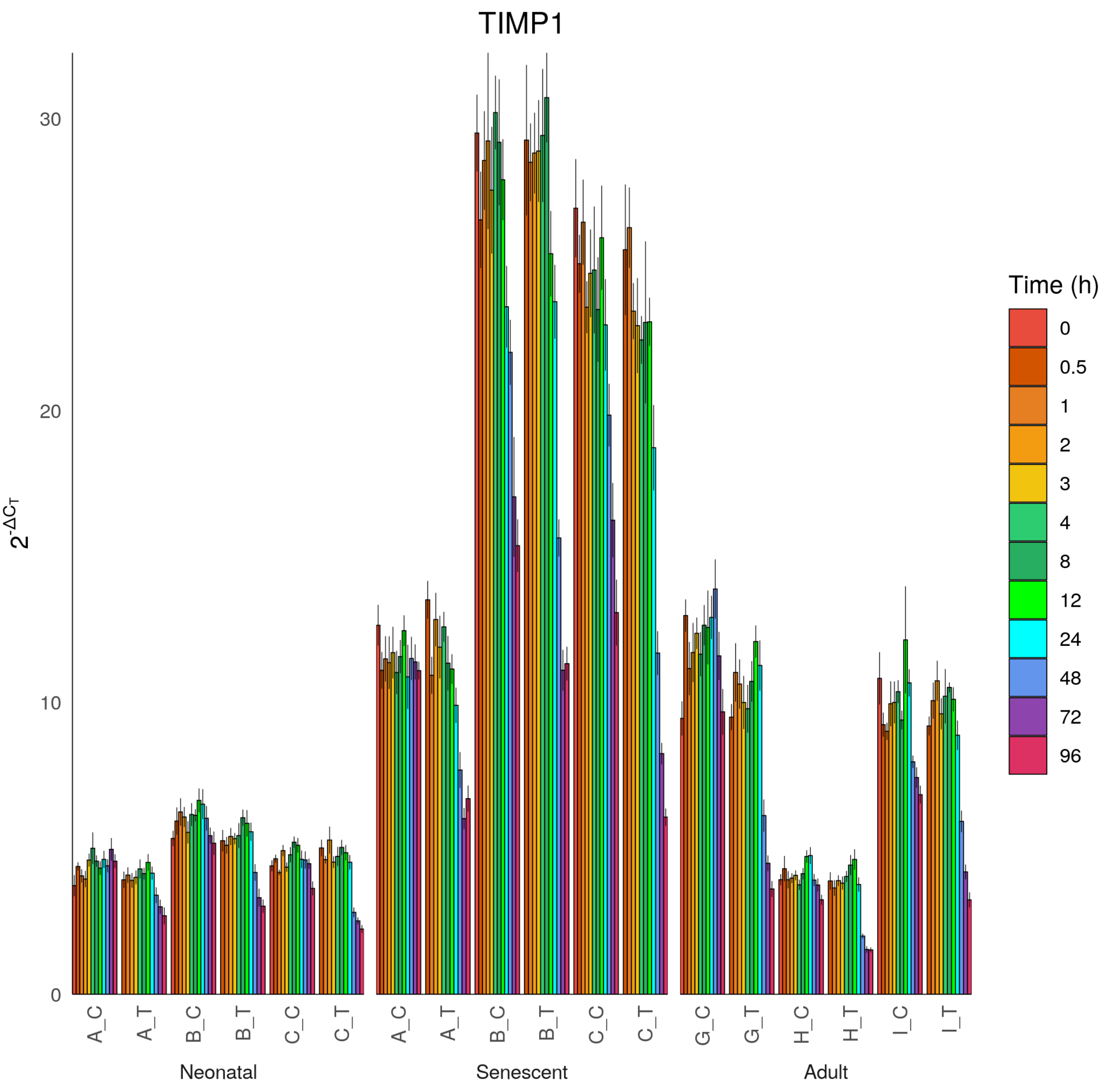
TIMP1
2^-ΔCT
0
10
20
30
A_C
A_T
B_C
B_T
C_C
C_T
G_C
G_T
H_C
H_T
I_C
I_T
Neonatal
Senescent
Adult
Time (h)
0
0.5
1
2
3
4
8
12
24
48
72
96

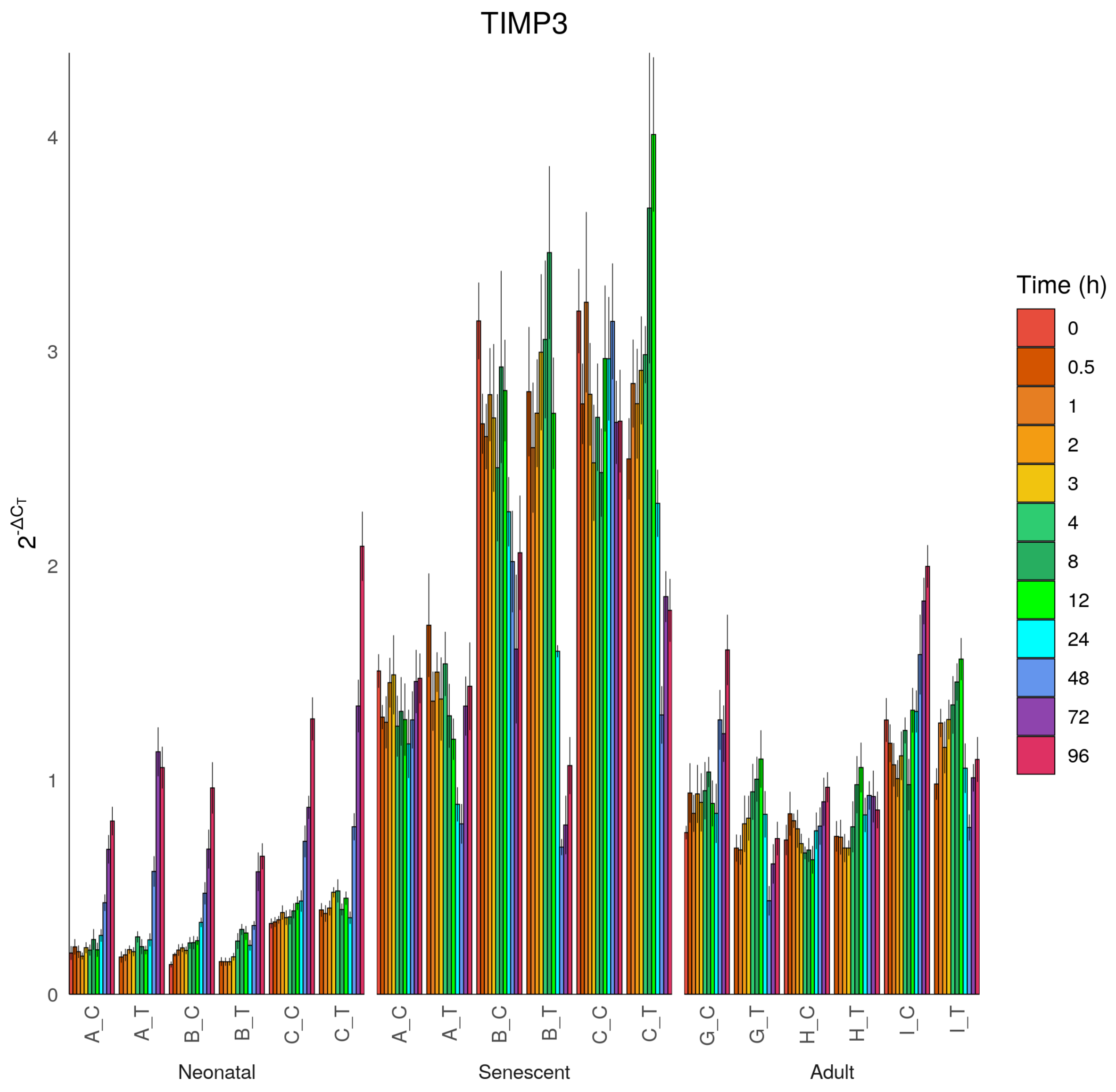
TIMP3
$2^{-\Delta C_T}$
0
1
2
3
4
A_C
A_T
B_C
B_T
C_C
C_T
Neonatal
Senescent
G_C
G_T
H_C
H_T
I_C
I_T
Adult
Time (h)
0
0.5
1
2
3
4
8
12
24
48
72
96

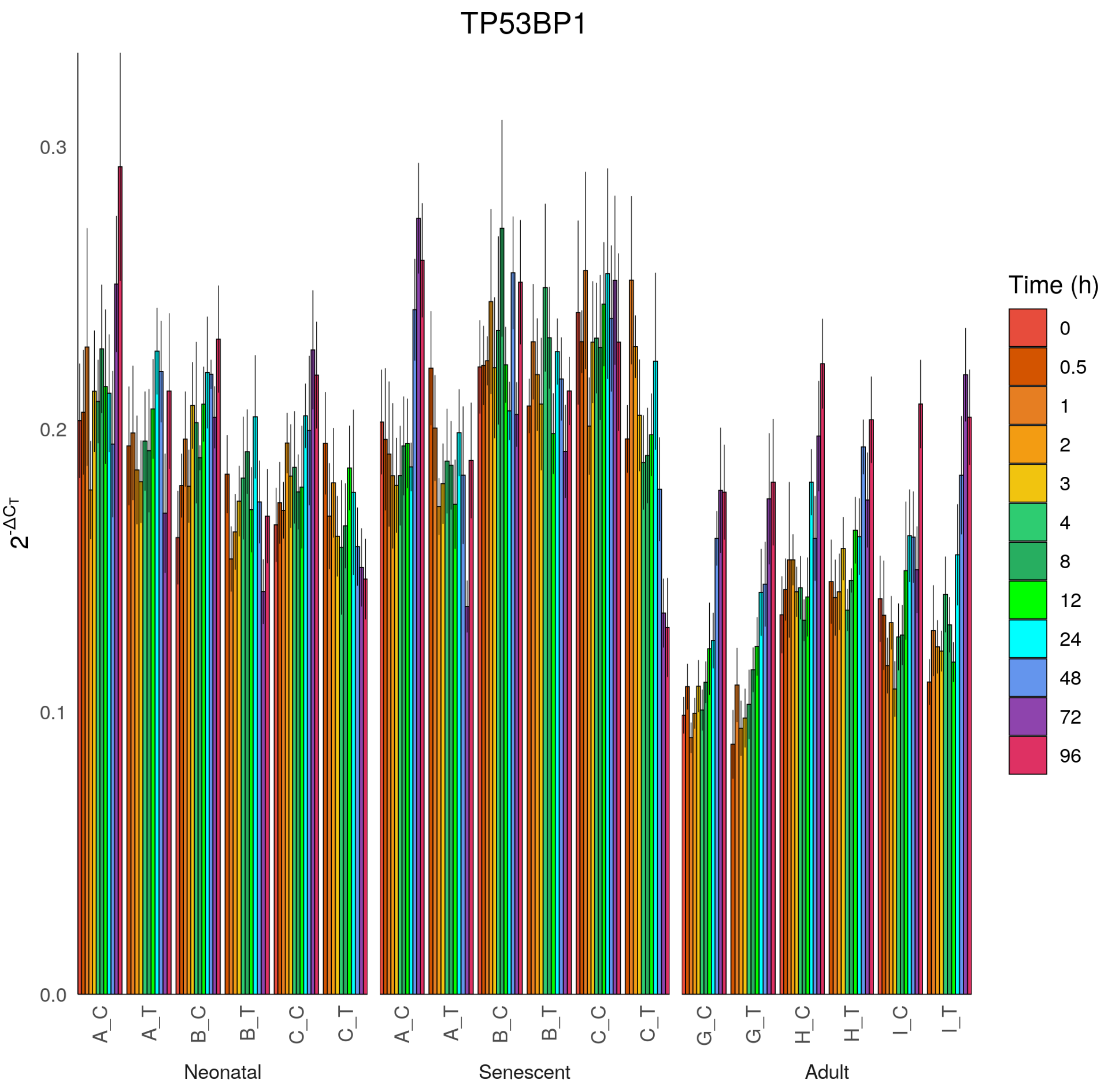
TP53BP1
$2^{-\Delta C_T}$
0.0
0.1
0.2
0.3
A_C
A_T
B_C
B_T
C_C
C_T
A_C
A_T
B_C
B_T
C_C
C_T
G_C
G_T
H_C
H_T
I_C
I_T
Neonatal
Senescent
Adult
Time (h)
0
0.5
1
2
3
4
8
12
24
48
72
96

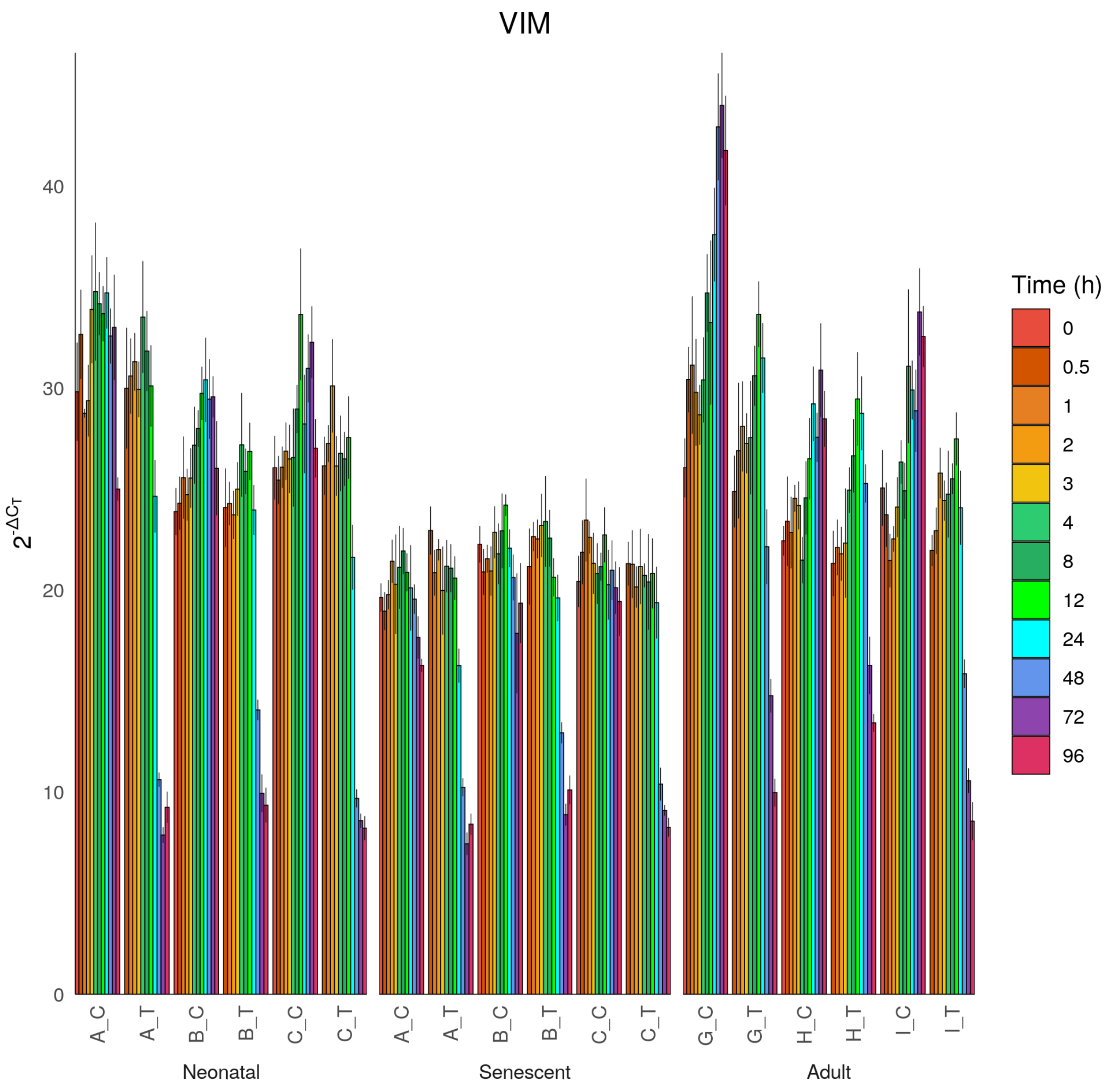

VIM
$2^{-\Delta C_T}$
0
10
20
30
40
A_C
A_T
B_C
B_T
C_C
C_T
A_C
A_T
B_C
B_T
C_C
C_T
G_C
G_T
H_C
H_T
I_C
I_T
Neonatal
Senescent
Adult
Time (h)
0
0.5
1
2
3
4
8
12
24
48
72
96
