# Supplementary material for: Cell Senescence-Independent Changes of Human Skin Fibroblasts with Age
Source: Cells. 2024 Apr 9;13(8):659. doi: 10.3390/cells13080659 (PMC11048776; doi:10.3390/cells13080659)
Supplement: Supplementary file 1 [file cells-13-00659-s001.zip › Supplementary file 3.pdf]

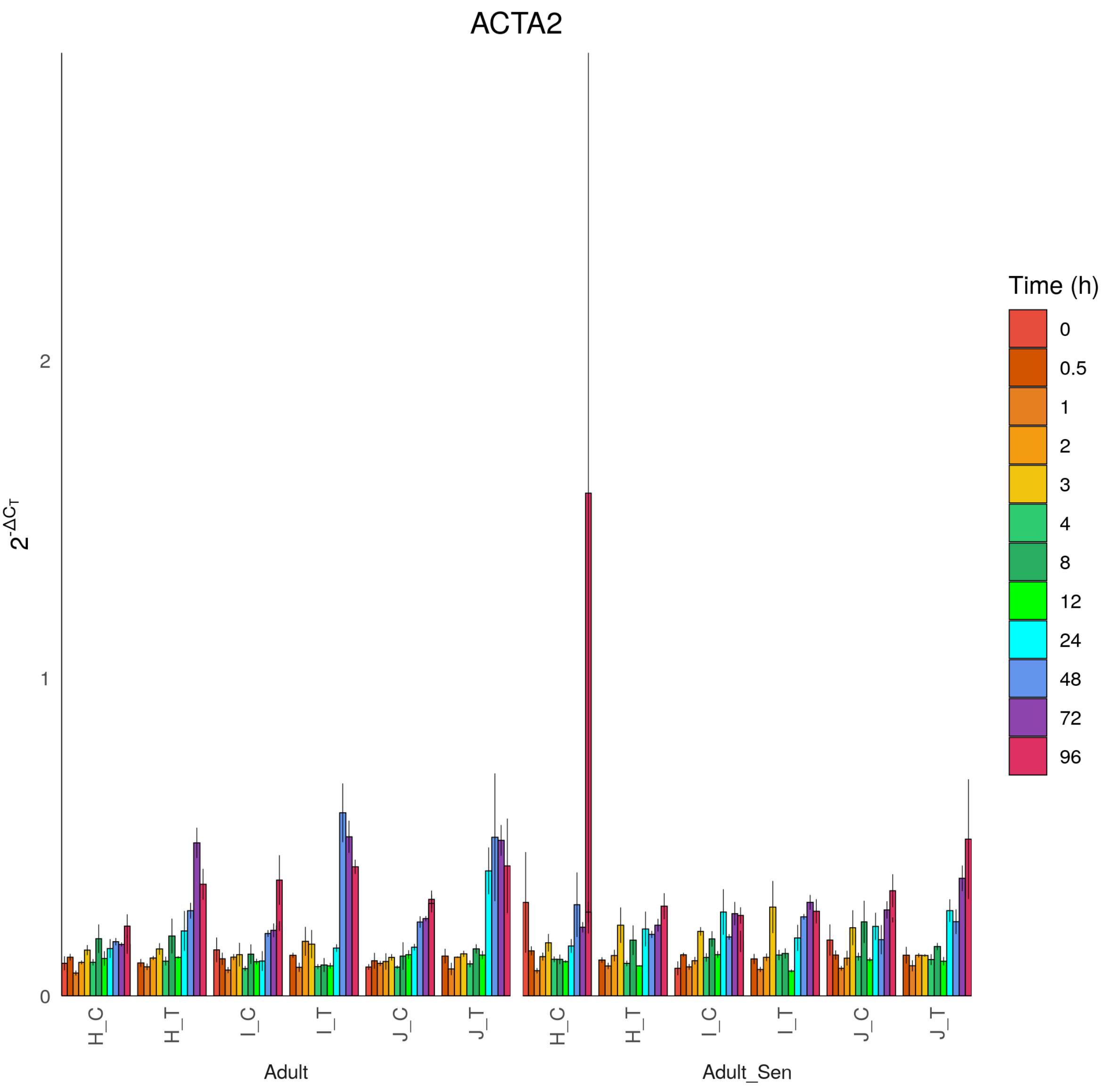
ACTA2
$2^{-\Delta C_T}$
0
1
2
H_C
H_T
I_C
I_T
J_C
J_T
Adult
Adult_Sen
Time (h)
0
0.5
1
2
3
4
8
12
24
48
72
96

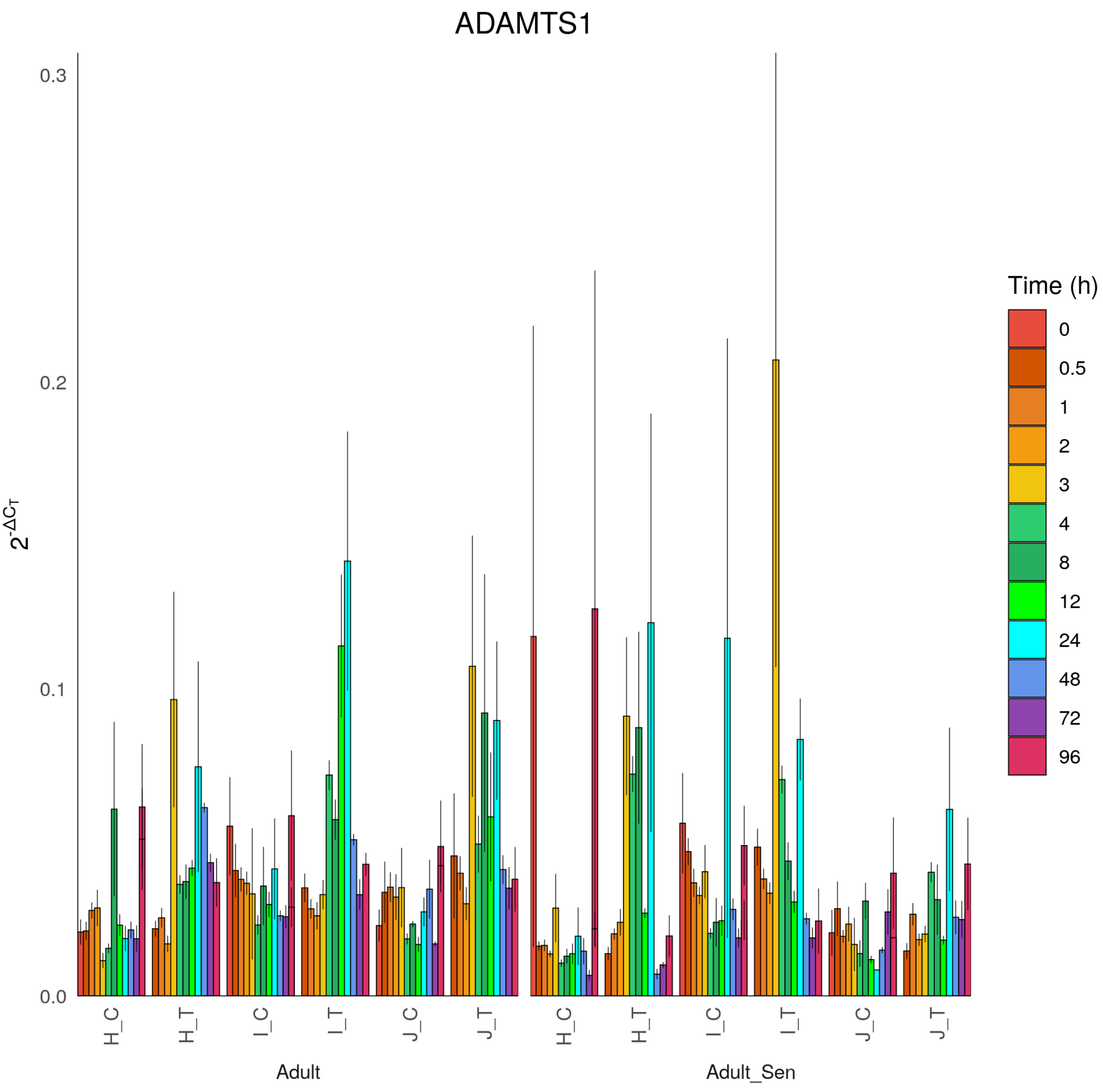
ADAMTS1
$2^{-\Delta C_T}$
0.0
0.1
0.2
0.3
H_C
H_T
I_C
I_T
J_C
J_T
Adult
Adult_Sen
Time (h)
0
0.5
1
2
3
4
8
12
24
48
72
96

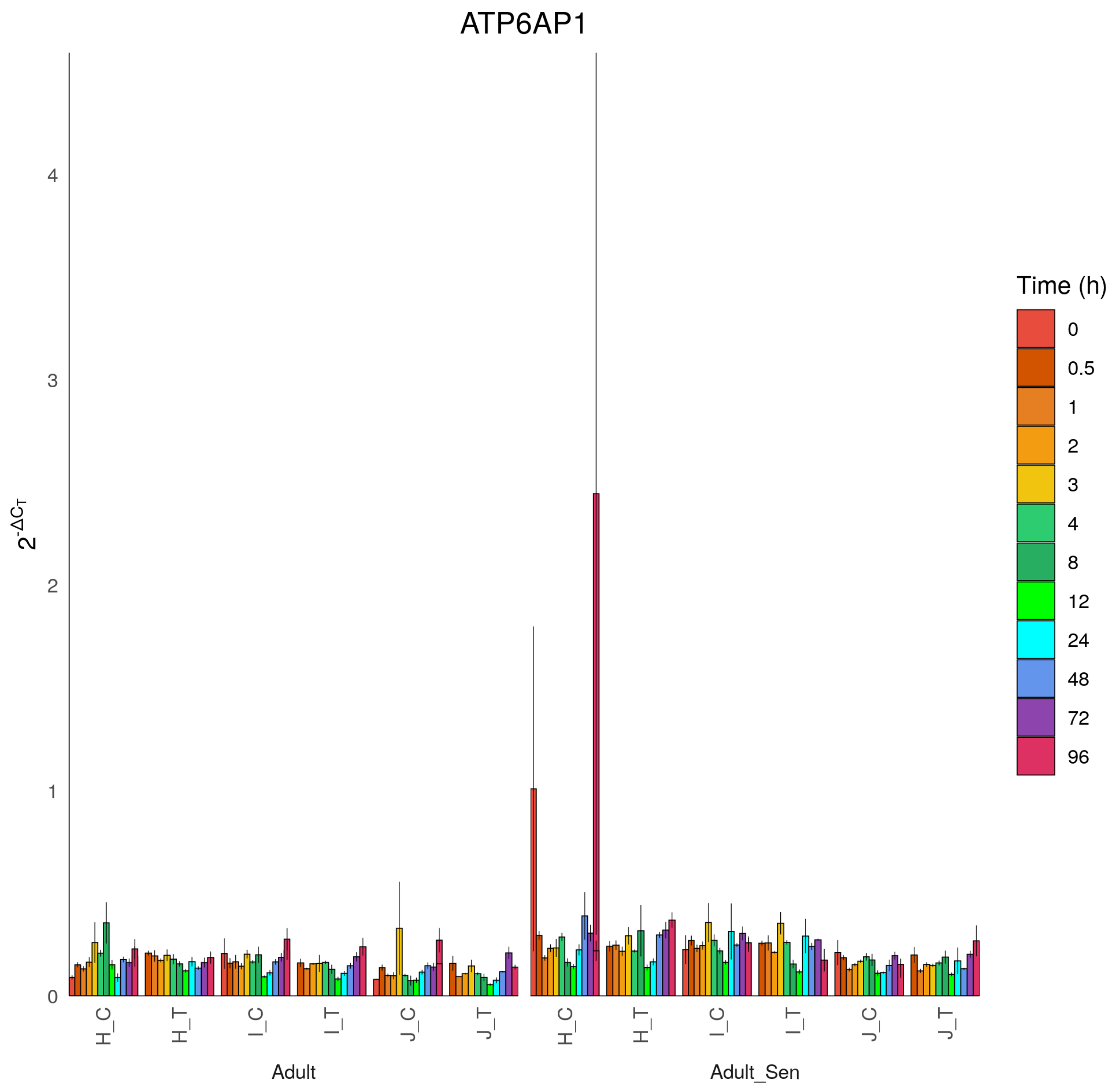
ATP6AP1
$2^{-\Delta C_T}$
0
1
2
3
4
H_C
H_T
I_C
I_T
J_C
J_T
Adult
Adult_Sen
Time (h)
0
0.5
1
2
3
4
8
12
24
48
72
96

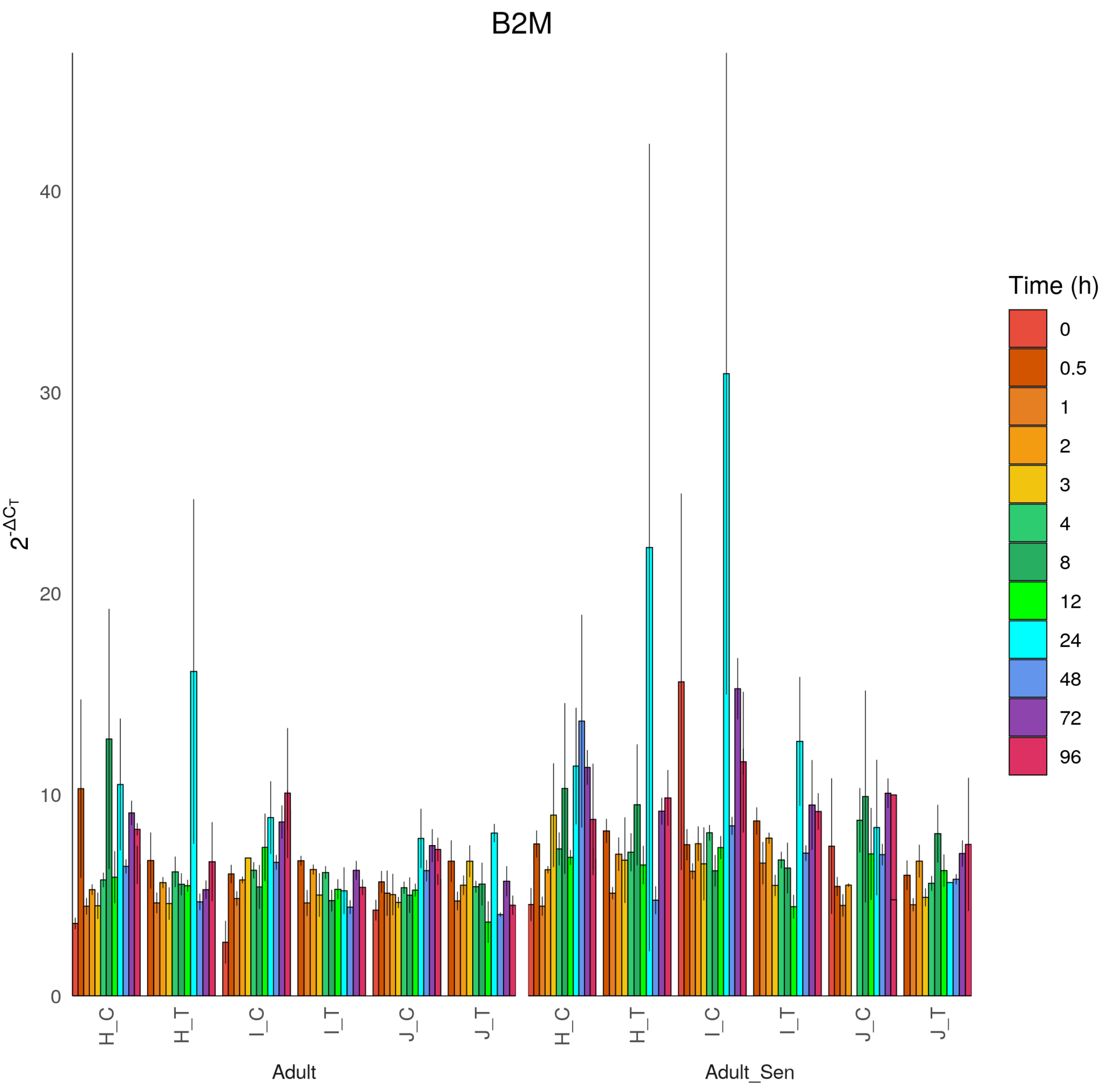
B2M
$2^{-\Delta C_T}$
0
10
20
30
40
H_C
H_T
I_C
I_T
J_C
J_T
Adult
Adult_Sen
Time (h)
0
0.5
1
2
3
4
8
12
24
48
72
96

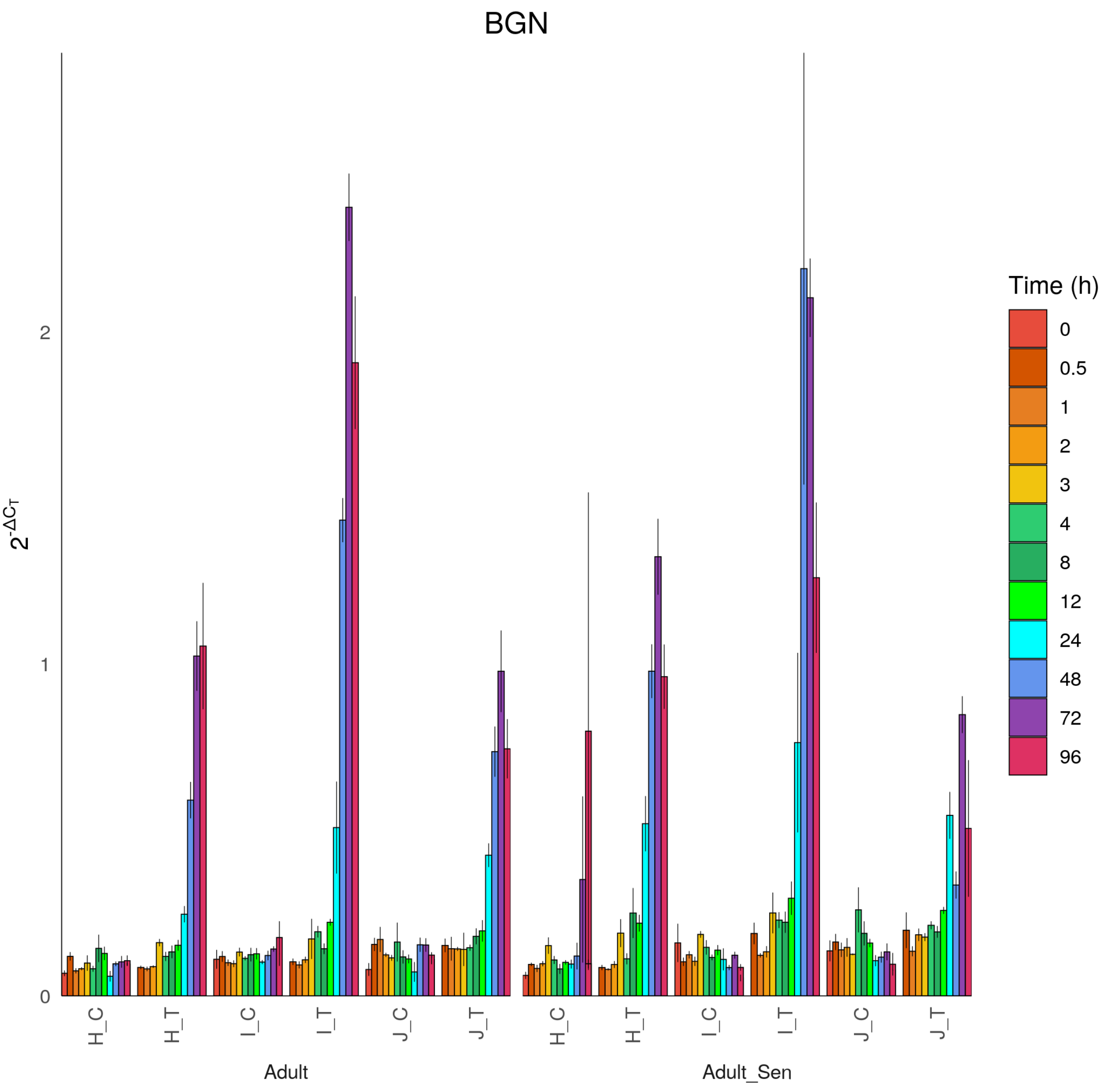

BGN
$2^{-\Delta C_T}$
0
1
2
H_C
H_T
I_C
I_T
J_C
J_T
Adult
Adult_Sen
Time (h)
0
0.5
1
2
3
4
8
12
24
48
72
96

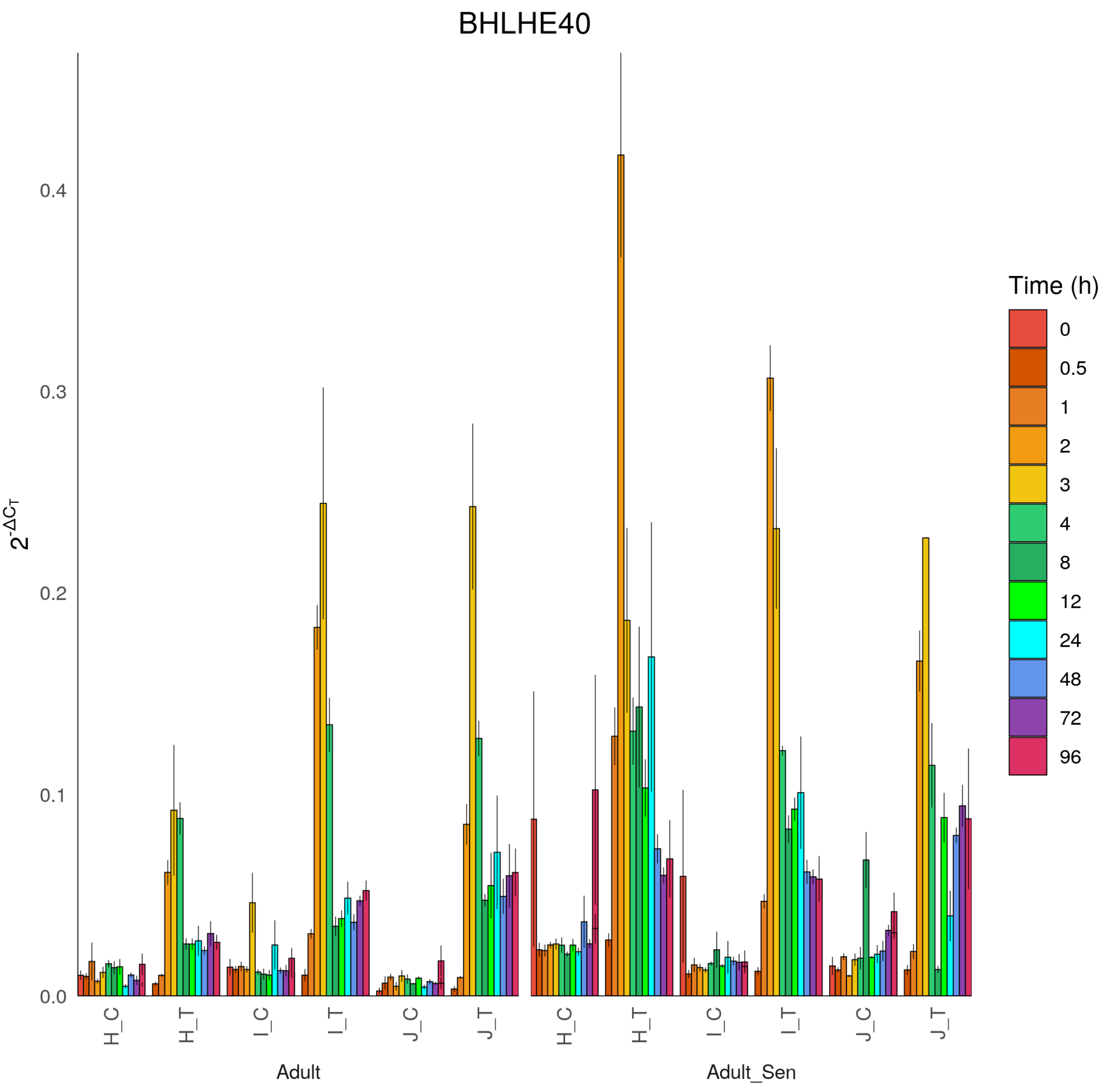

BHLHE40
$2^{-\Delta C_T}$
0.0
0.1
0.2
0.3
0.4
H_C
H_T
I_C
I_T
J_C
J_T
Adult
Adult_Sen
Time (h)
0
0.5
1
2
3
4
8
12
24
48
72
96

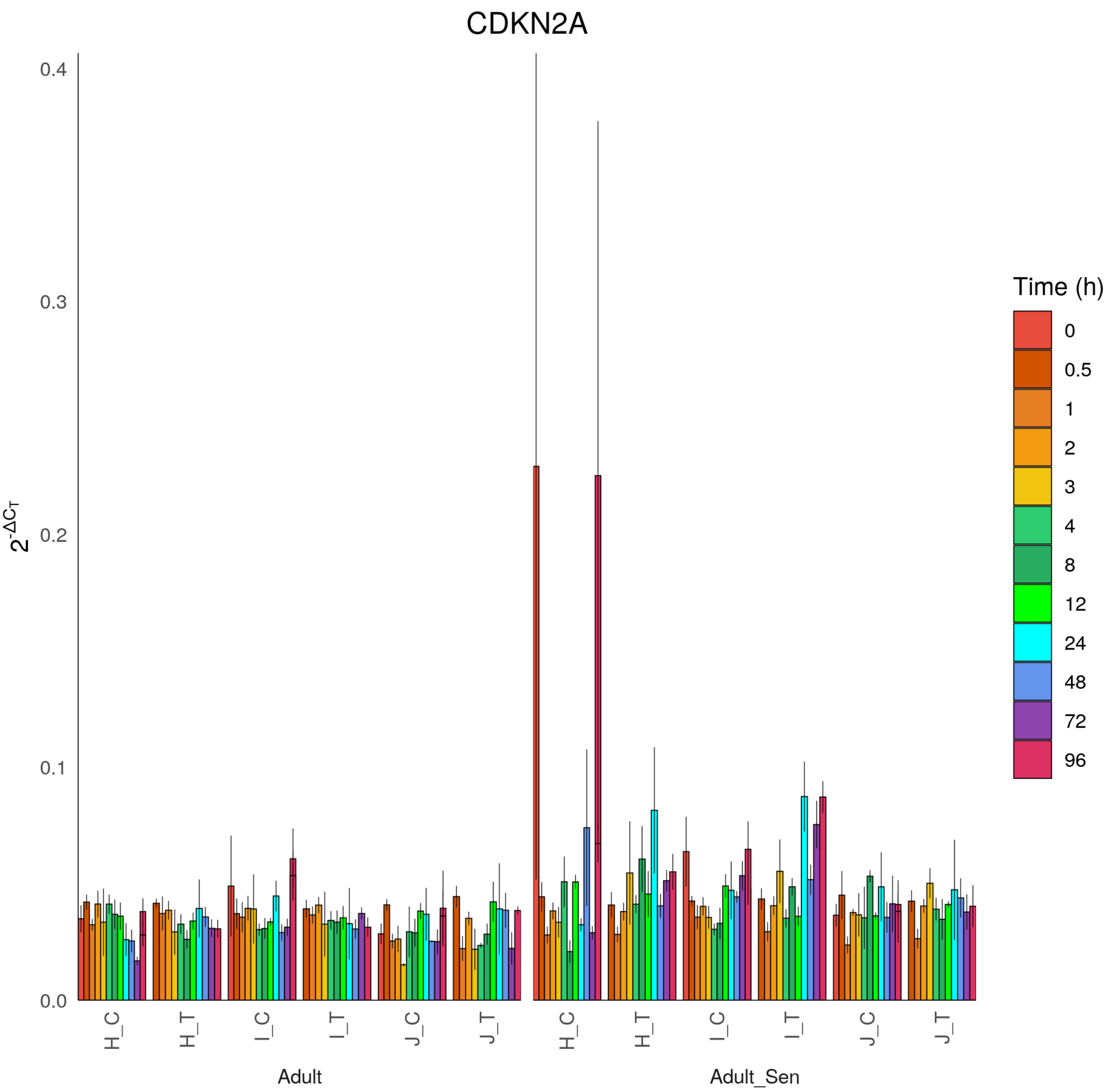

CDKN2A
$2^{-\Delta C_T}$
0.0
0.1
0.2
0.3
0.4
H_C
H_T
I_C
I_T
J_C
J_T
Adult
Adult_Sen
Time (h)
0
0.5
1
2
3
4
8
12
24
48
72
96

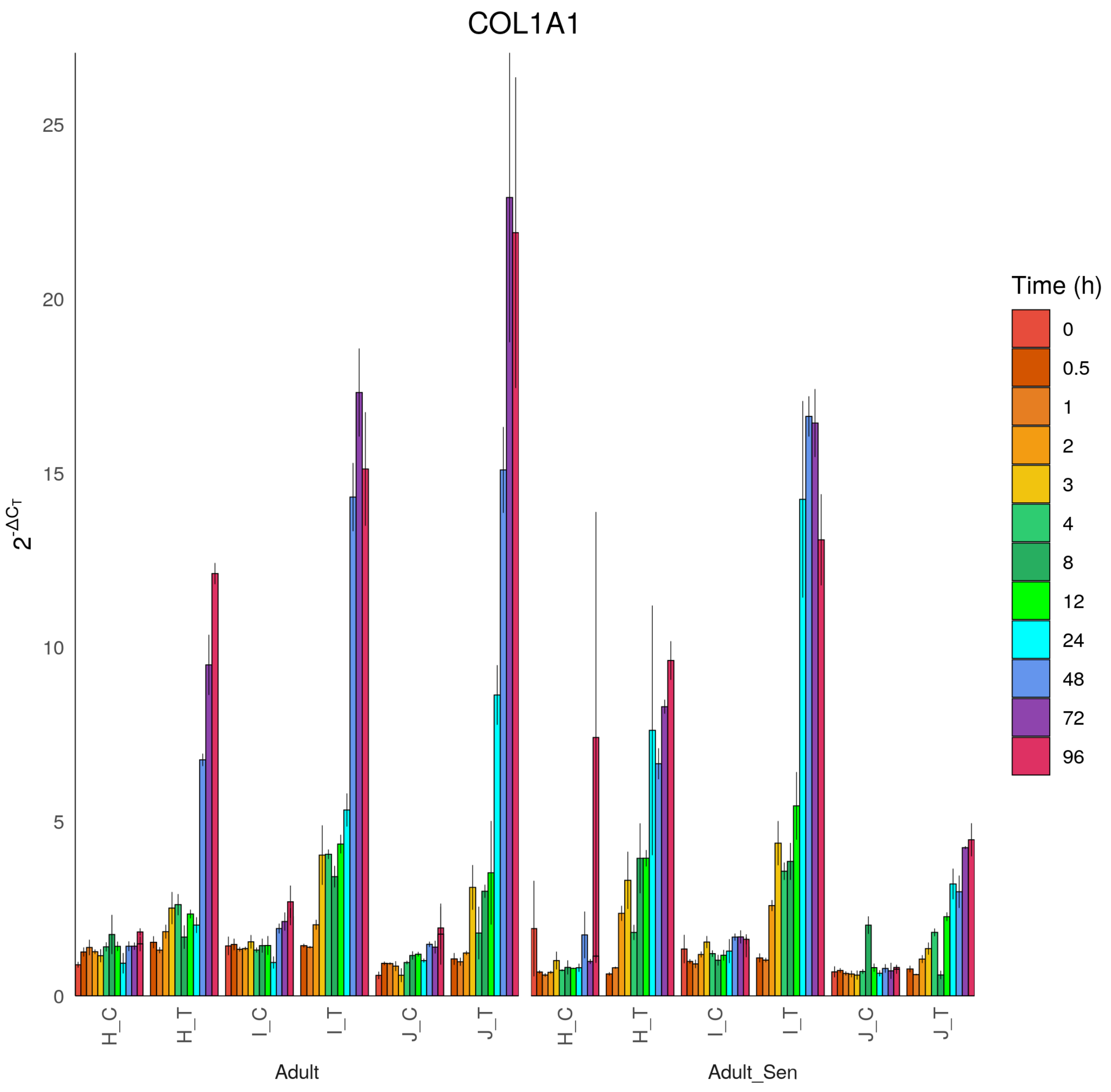

COL1A1
$2^{-\Delta C_T}$
0
5
10
15
20
25
H_C
H_T
I_C
I_T
J_C
J_T
Adult
Adult_Sen
Time (h)
0
0.5
1
2
3
4
8
12
24
48
72
96

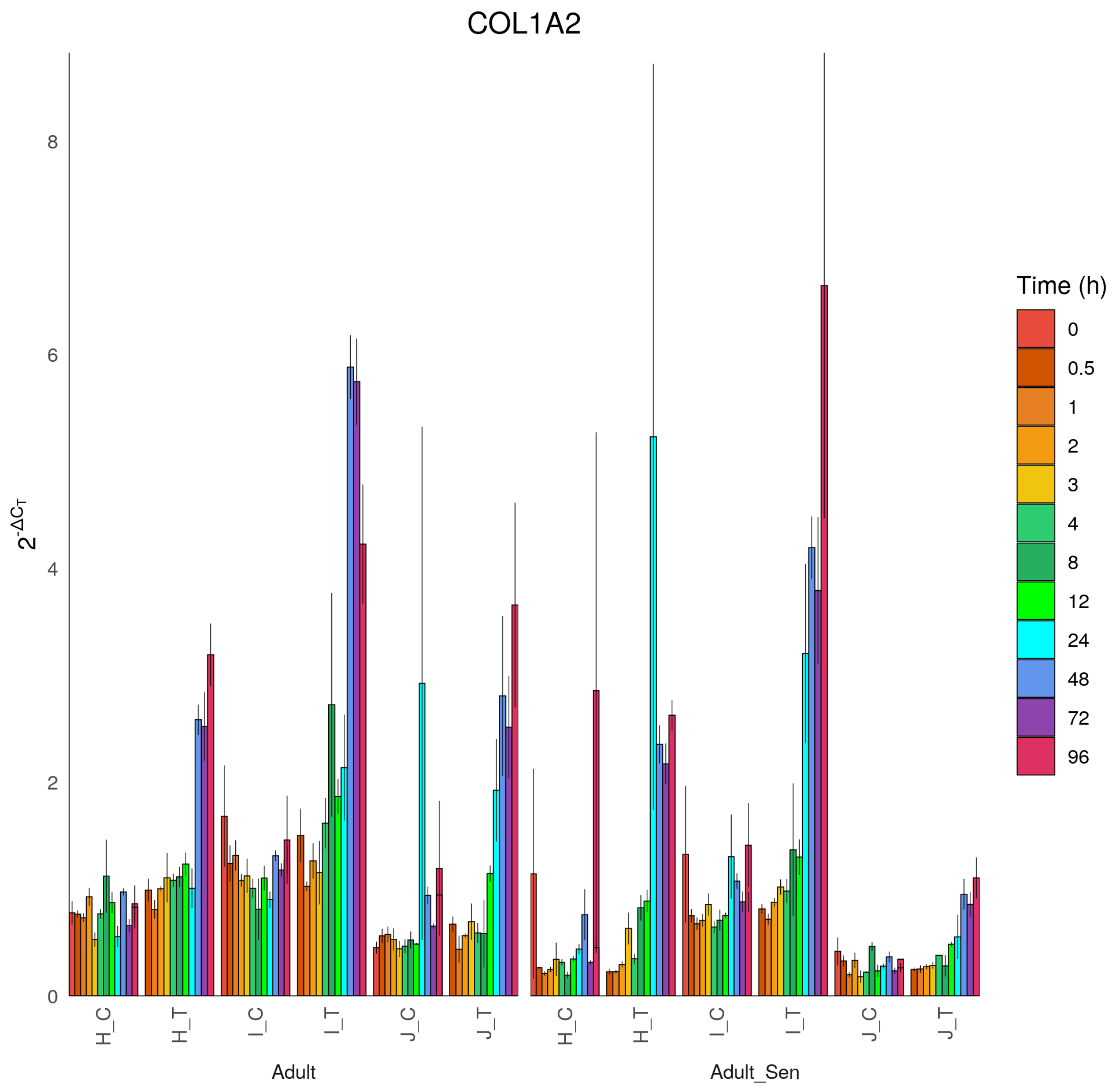

COL1A2
$2^{-\Delta C_T}$
0
2
4
6
8
H_C
H_T
I_C
I_T
J_C
J_T
Adult
Adult_Sen
Time (h)
0
0.5
1
2
3
4
8
12
24
48
72
96

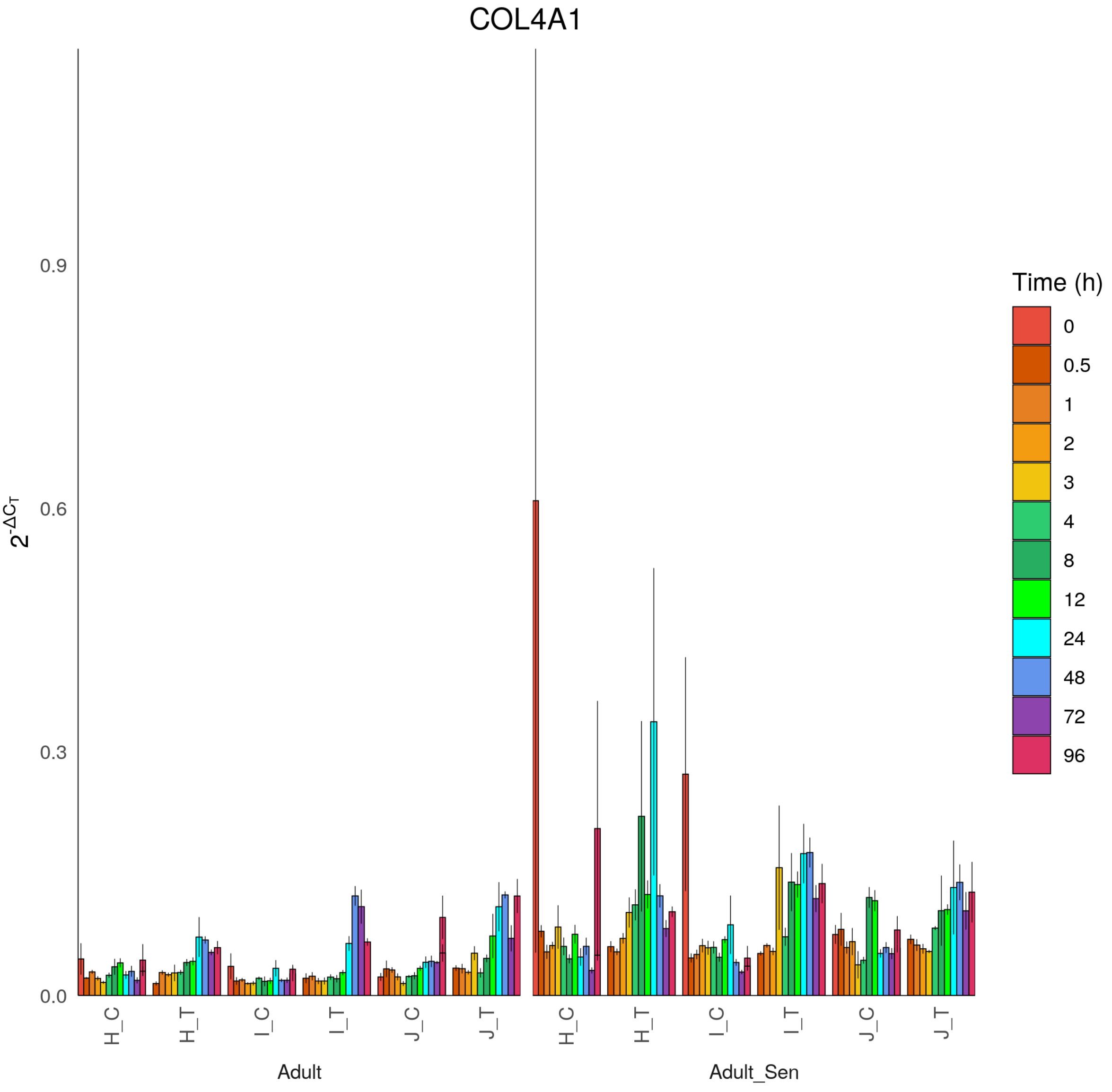
COL4A1
$2^{-\Delta C_T}$
0.0
0.3
0.6
0.9
H_C
H_T
I_C
I_T
J_C
J_T
Adult
Adult_Sen
Time (h)
0
0.5
1
2
3
4
8
12
24
48
72
96

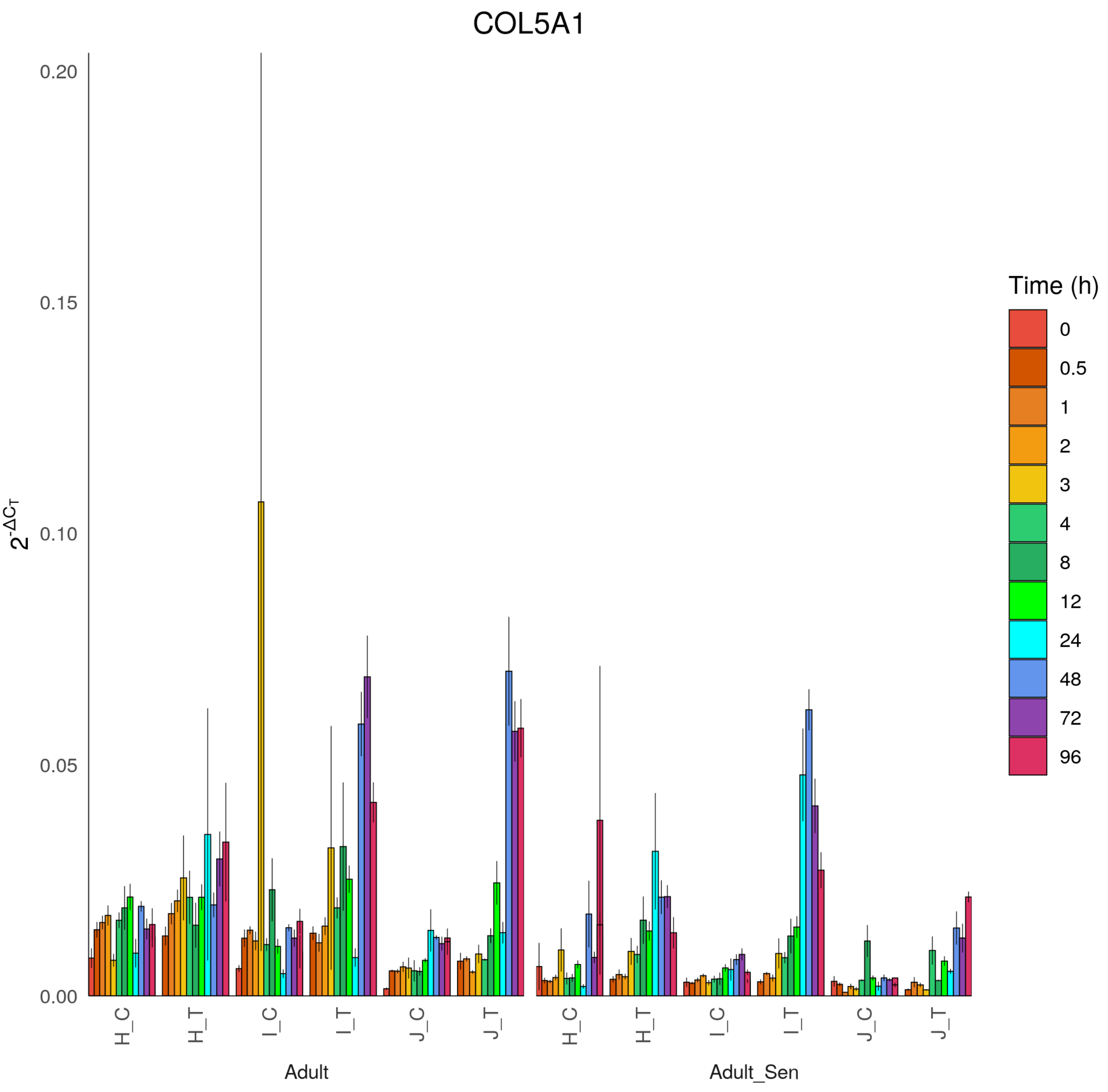
COL5A1
$2^{-\Delta C_T}$
0.20
0.15
0.10
0.05
0.00
H_C
H_T
I_C
I_T
J_C
J_T
H_C
H_T
I_C
I_T
J_C
J_T
Adult
Adult_Sen
Time (h)
0
0.5
1
2
3
4
8
12
24
48
72
96

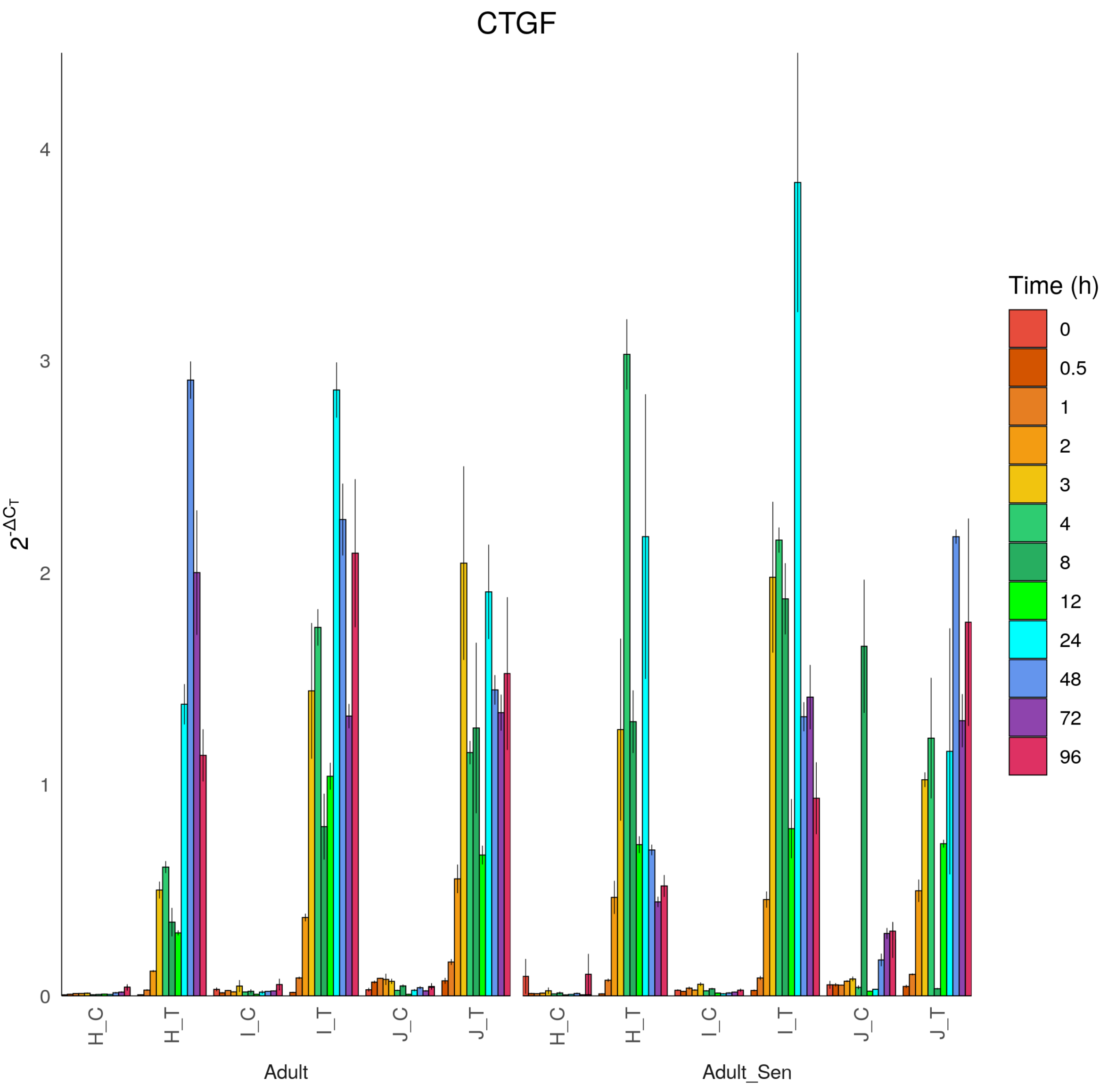

CTGF
$2^{-\Delta C_T}$
0
1
2
3
4
H_C
H_T
I_C
I_T
J_C
J_T
Adult
Adult_Sen
Time (h)
0
0.5
1
2
3
4
8
12
24
48
72
96

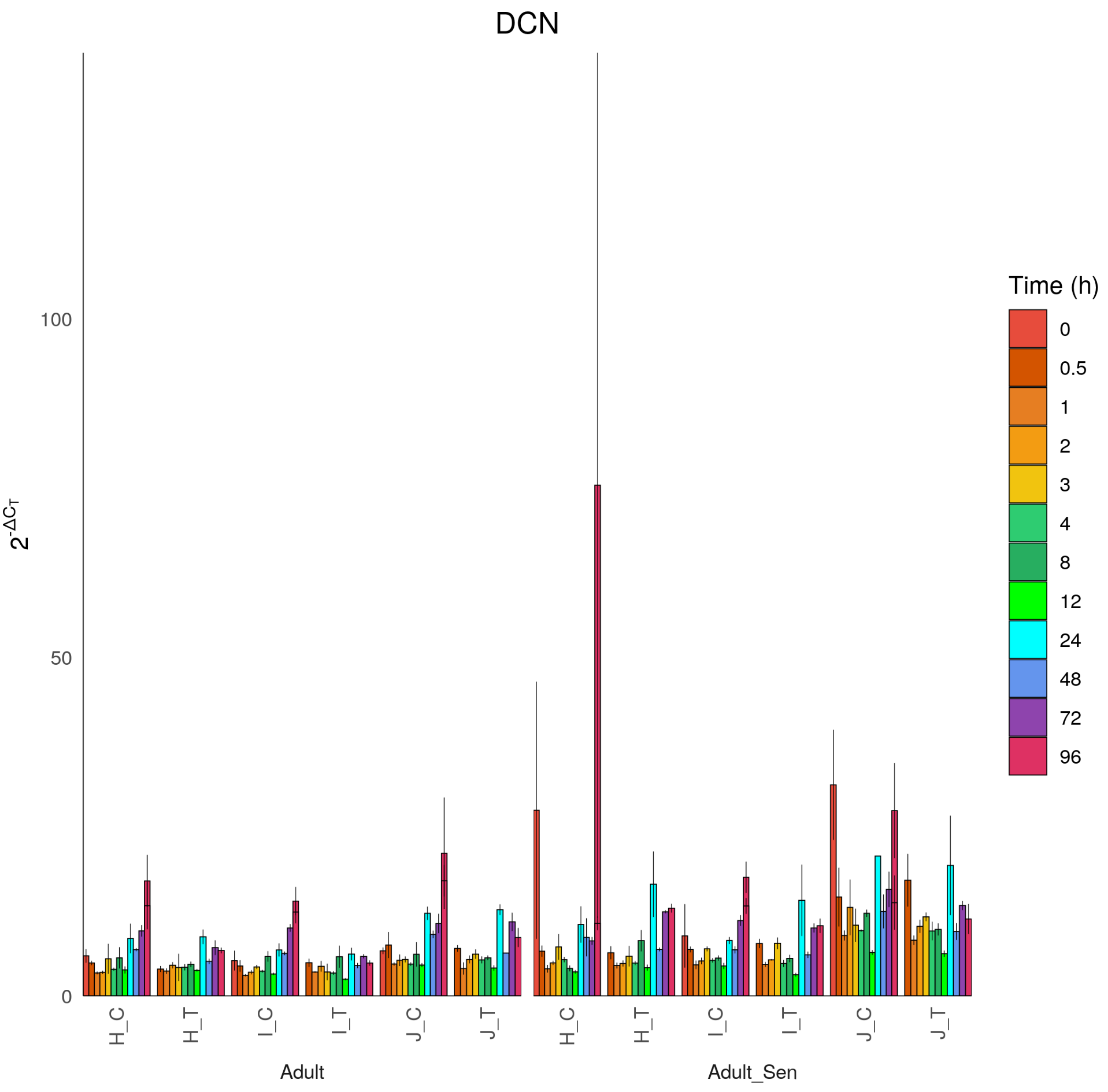

DCN
$2^{-\Delta C_T}$
100
50
0
H_C
H_T
I_C
I_T
J_C
J_T
Adult
Adult_Sen
Time (h)
0
0.5
1
2
3
4
8
12
24
48
72
96

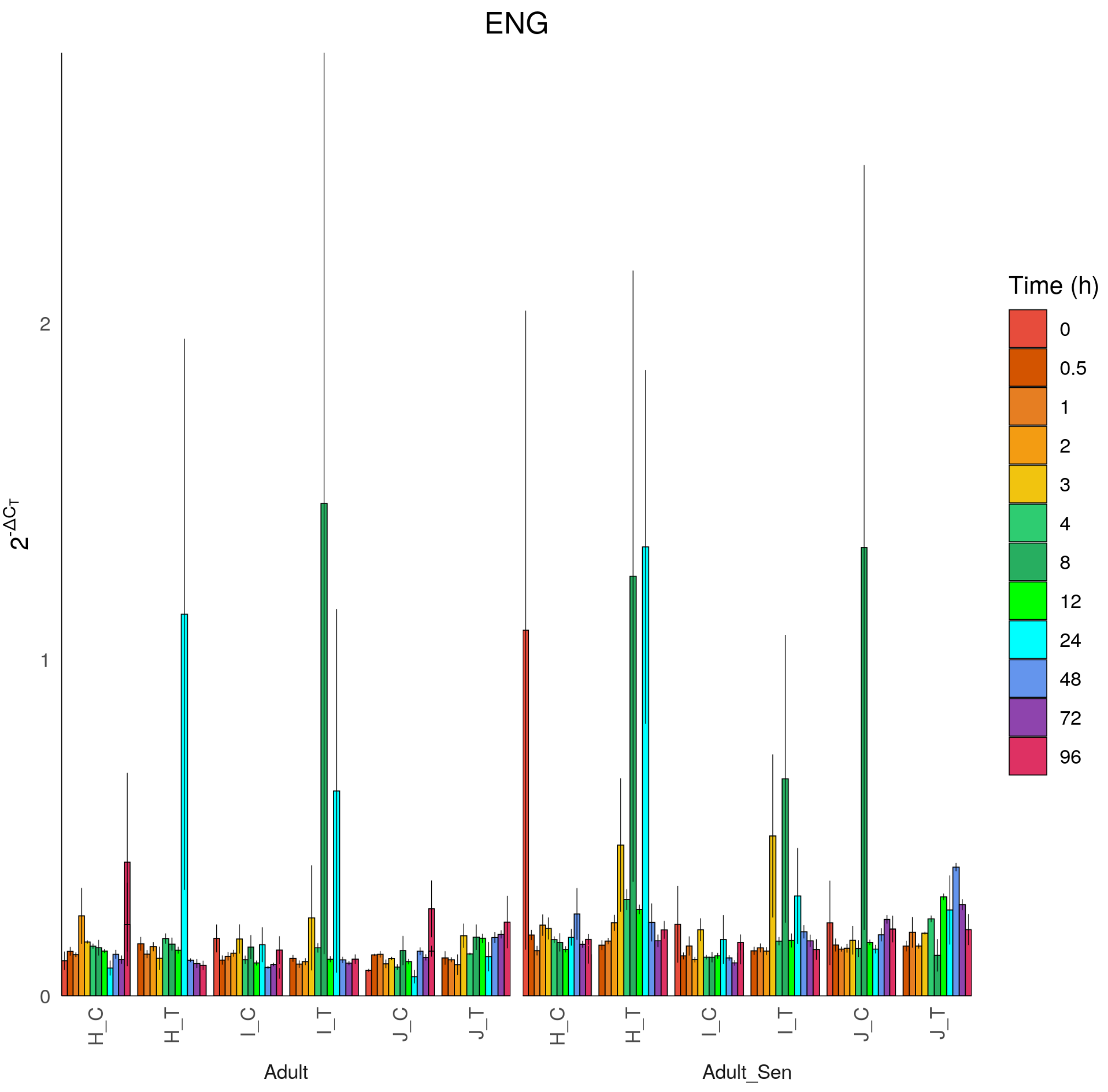

ENG
$2^{-\Delta C_T}$
0
1
2
H_C
H_T
I_C
I_T
J_C
J_T
Adult
Adult_Sen
Time (h)
0
0.5
1
2
3
4
8
12
24
48
72
96

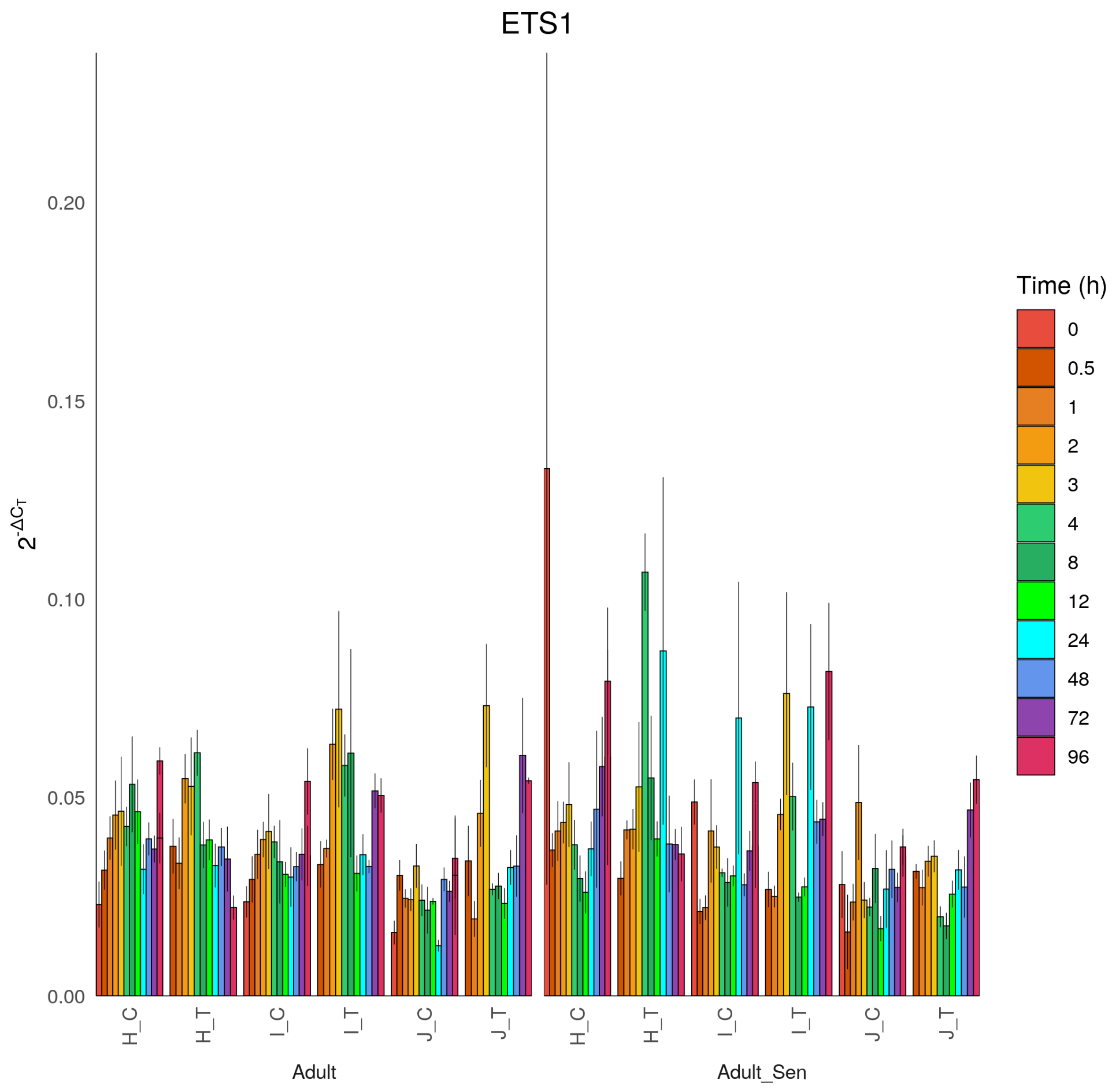

ETS1
$2^{-\Delta C_T}$
0.00
0.05
0.10
0.15
0.20
H_C
H_T
I_C
I_T
J_C
J_T
Adult
H_C
H_T
I_C
I_T
J_C
J_T
Adult_Sen
Time (h)
0
0.5
1
2
3
4
8
12
24
48
72
96

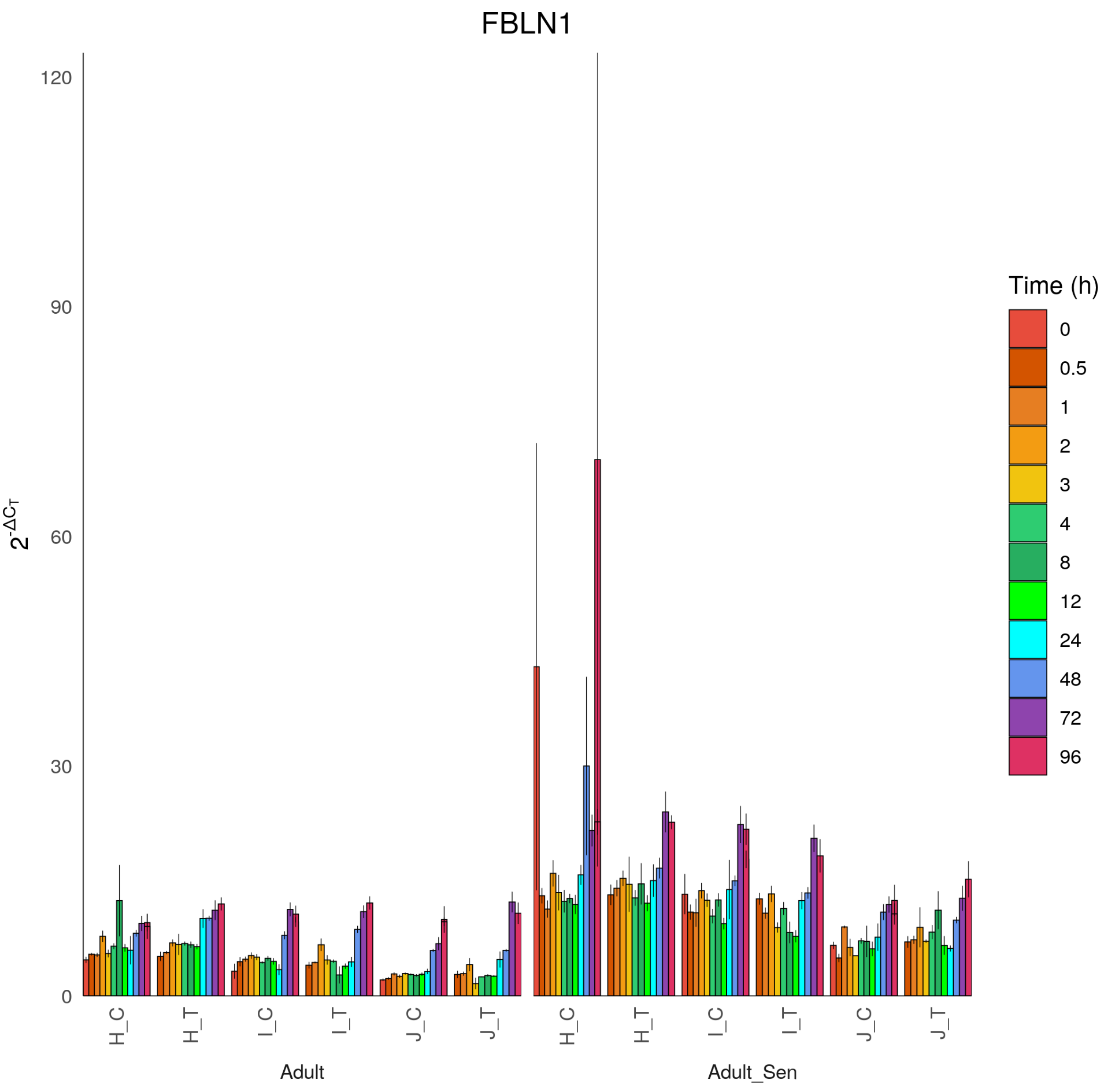

FBLN1
2^-ΔCT
0
30
60
90
120
H_C
H_T
I_C
I_T
J_C
J_T
H_C
H_T
I_C
I_T
J_C
J_T
Adult
Adult_Sen
Time (h)
0
0.5
1
2
3
4
8
12
24
48
72
96

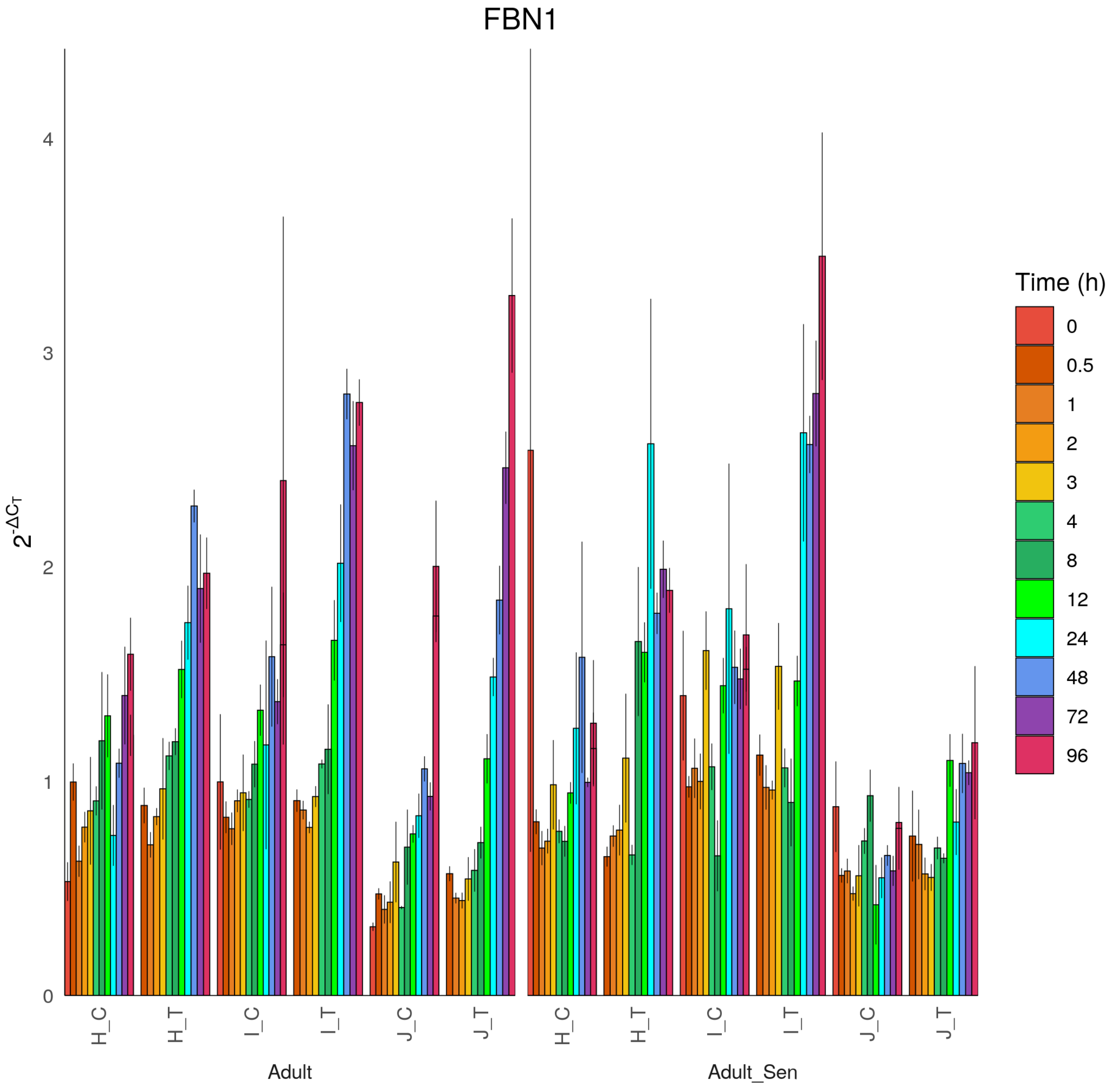
FBN1
$2^{-\Delta C_T}$
0
1
2
3
4
H_C
H_T
I_C
I_T
J_C
J_T
Adult
Adult_Sen
Time (h)
0
0.5
1
2
3
4
8
12
24
48
72
96

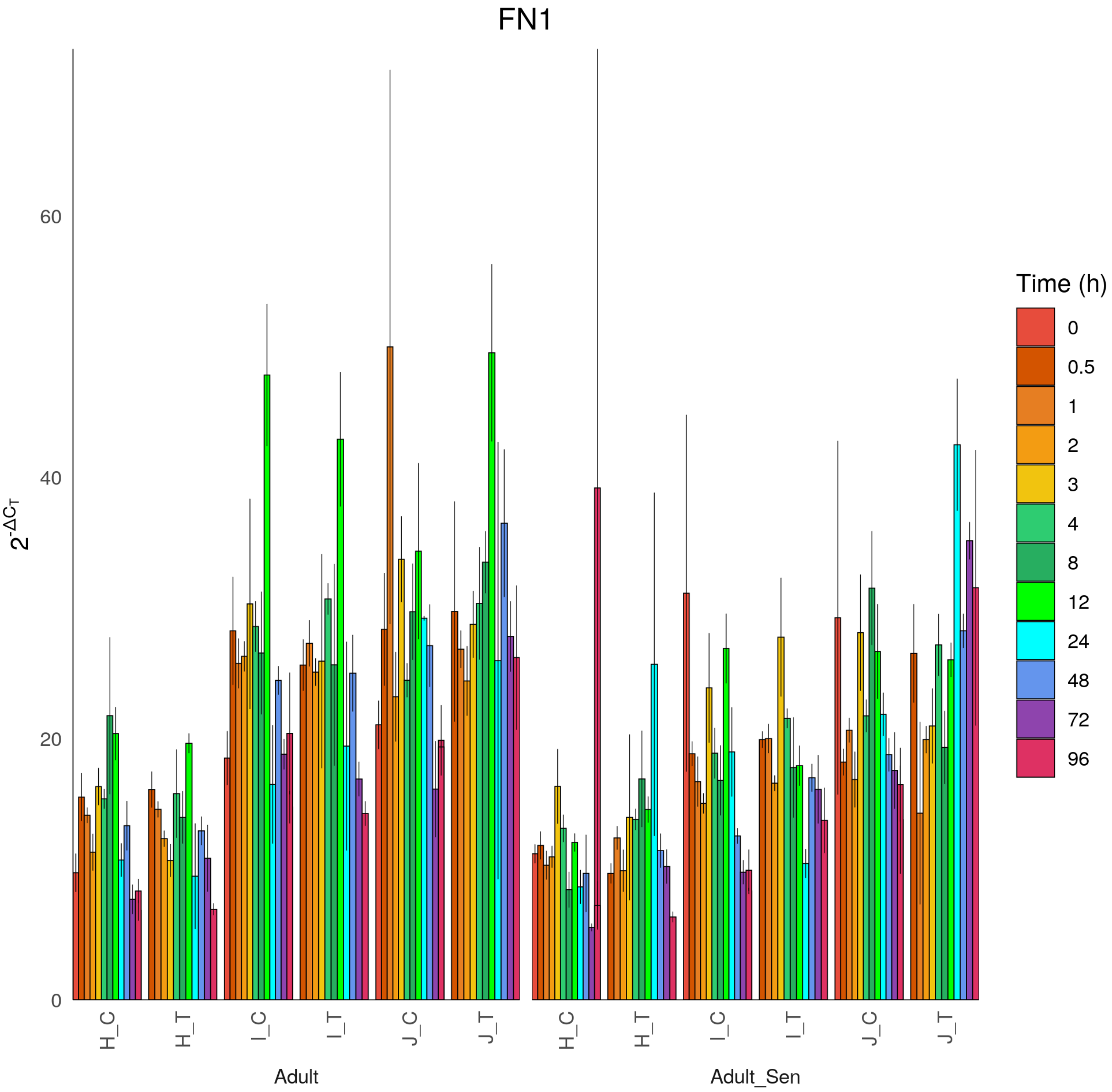
FN1
$2^{-\Delta C_T}$
0
20
40
60
H_C
H_T
I_C
I_T
J_C
J_T
Adult
Adult_Sen
Time (h)
0
0.5
1
2
3
4
8
12
24
48
72
96

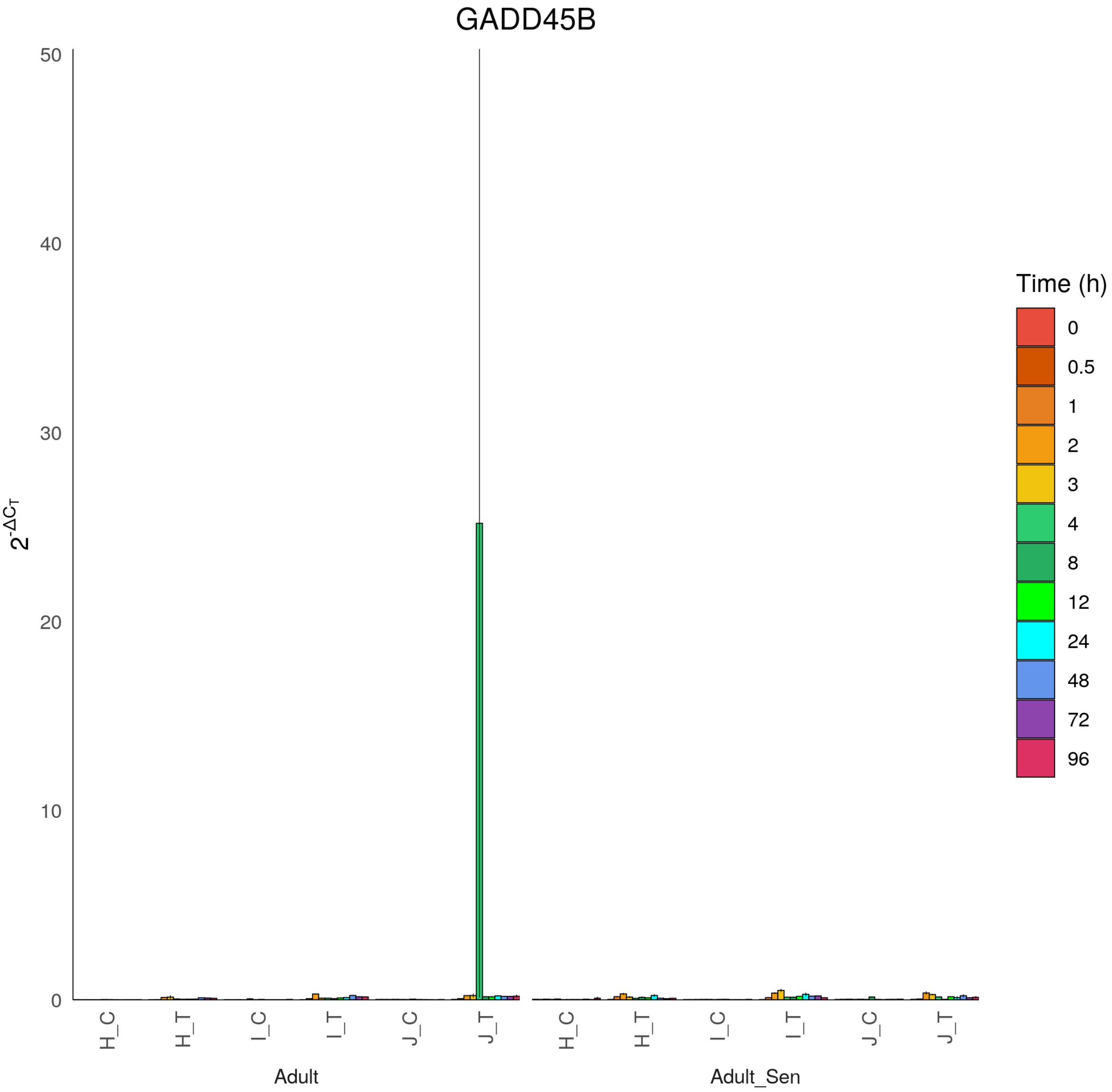
GADD45B
50
40
30
20
10
0
$2^{-\Delta C_T}$
H_C
H_T
I_C
I_T
J_C
J_T
Adult
H_C
H_T
I_C
I_T
J_C
J_T
Adult_Sen
Time (h)
0
0.5
1
2
3
4
8
12
24
48
72
96

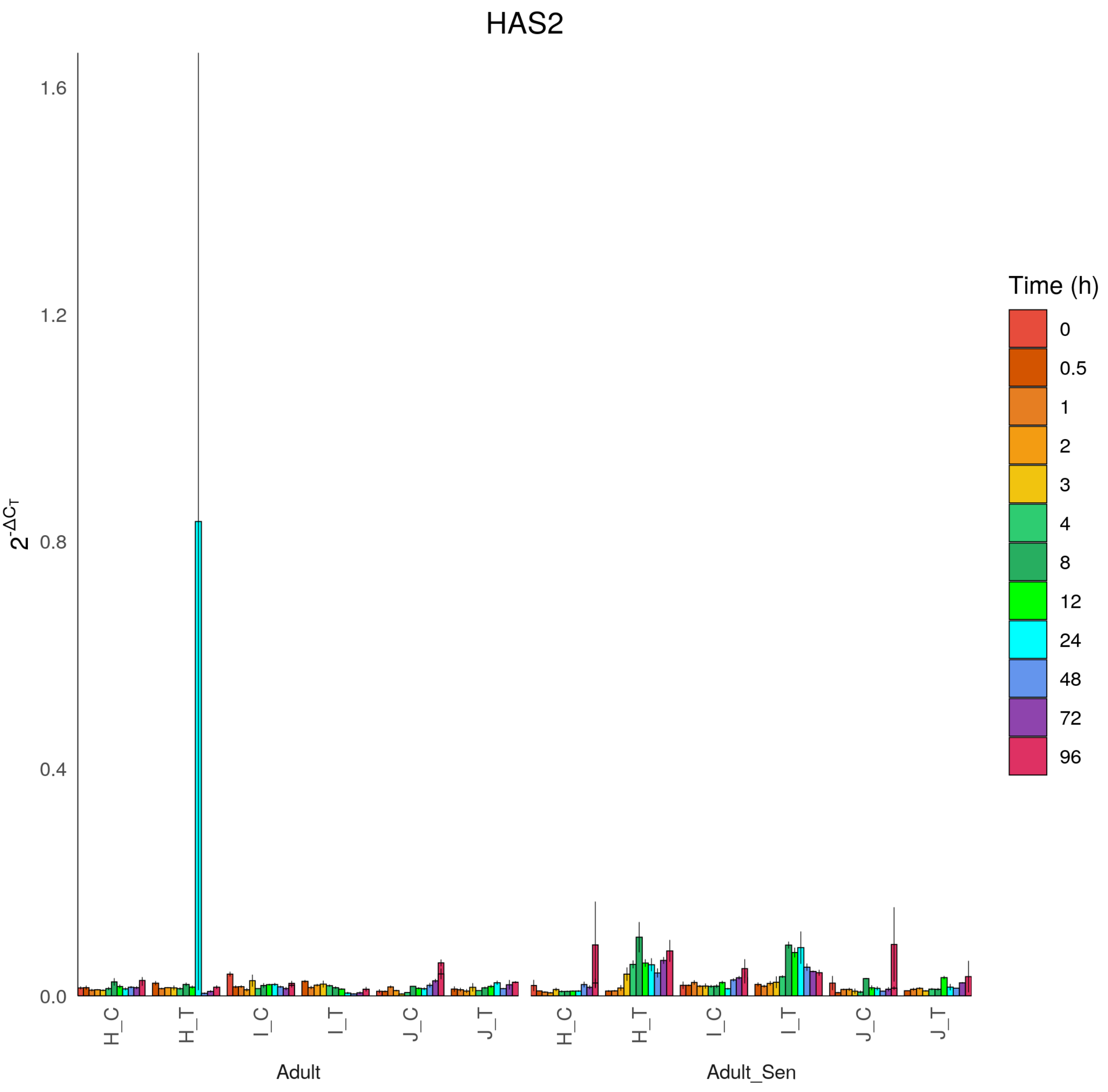

HAS2
$2^{-\Delta C_T}$
1.6
1.2
0.8
0.4
0.0
H_C
H_T
I_C
I_T
J_C
J_T
Adult
Adult_Sen
Time (h)
0
0.5
1
2
3
4
8
12
24
48
72
96

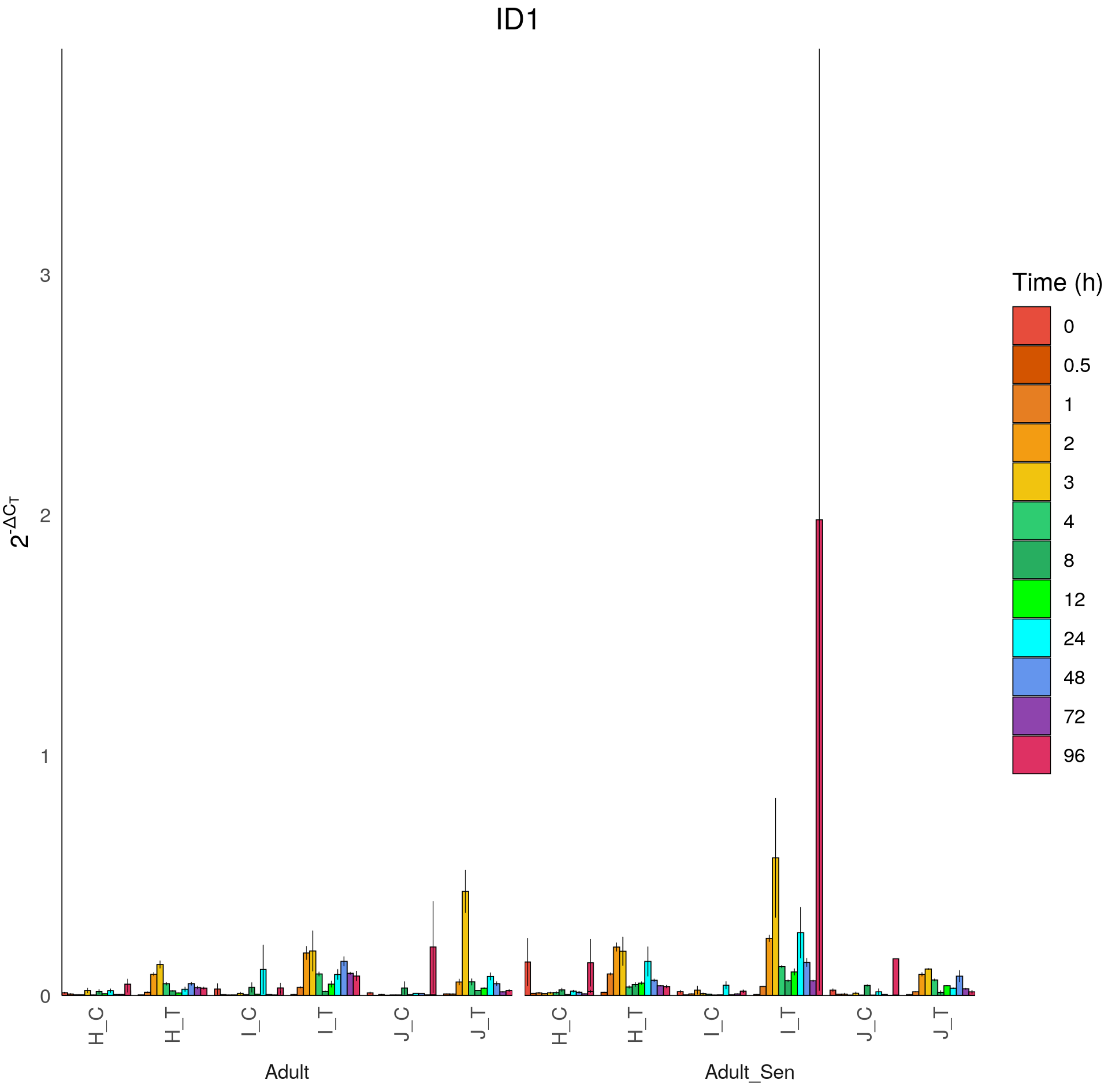
ID1
$2^{-\Delta C_T}$
0
1
2
3
H_C
H_T
I_C
I_T
J_C
J_T
Adult
Adult_Sen
Time (h)
0
0.5
1
2
3
4
8
12
24
48
72
96

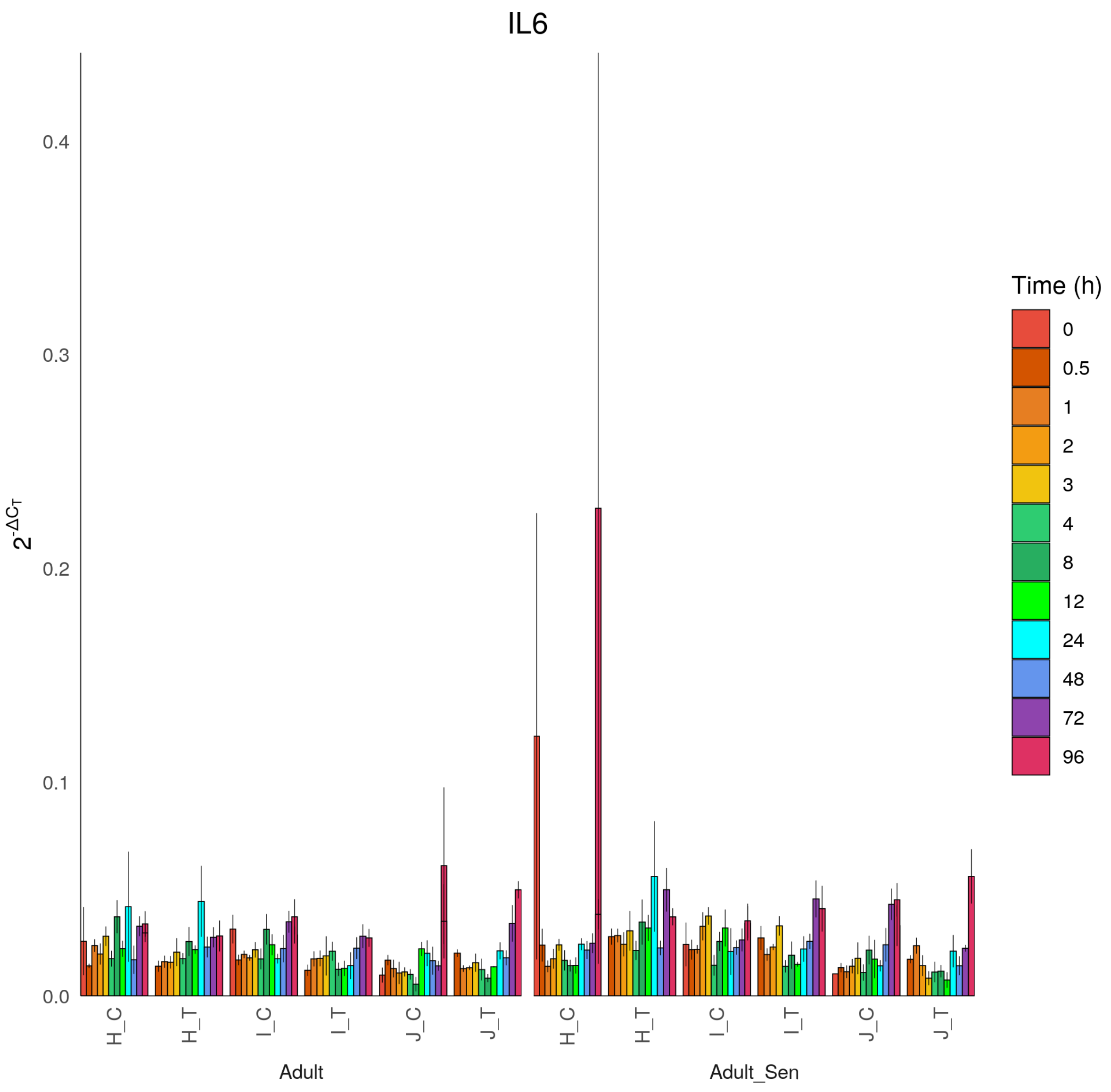
IL6
$2^{-\Delta C_T}$
0.4
0.3
0.2
0.1
0.0
H_C
H_T
I_C
I_T
J_C
J_T
Adult
Adult_Sen
Time (h)
0
0.5
1
2
3
4
8
12
24
48
72
96

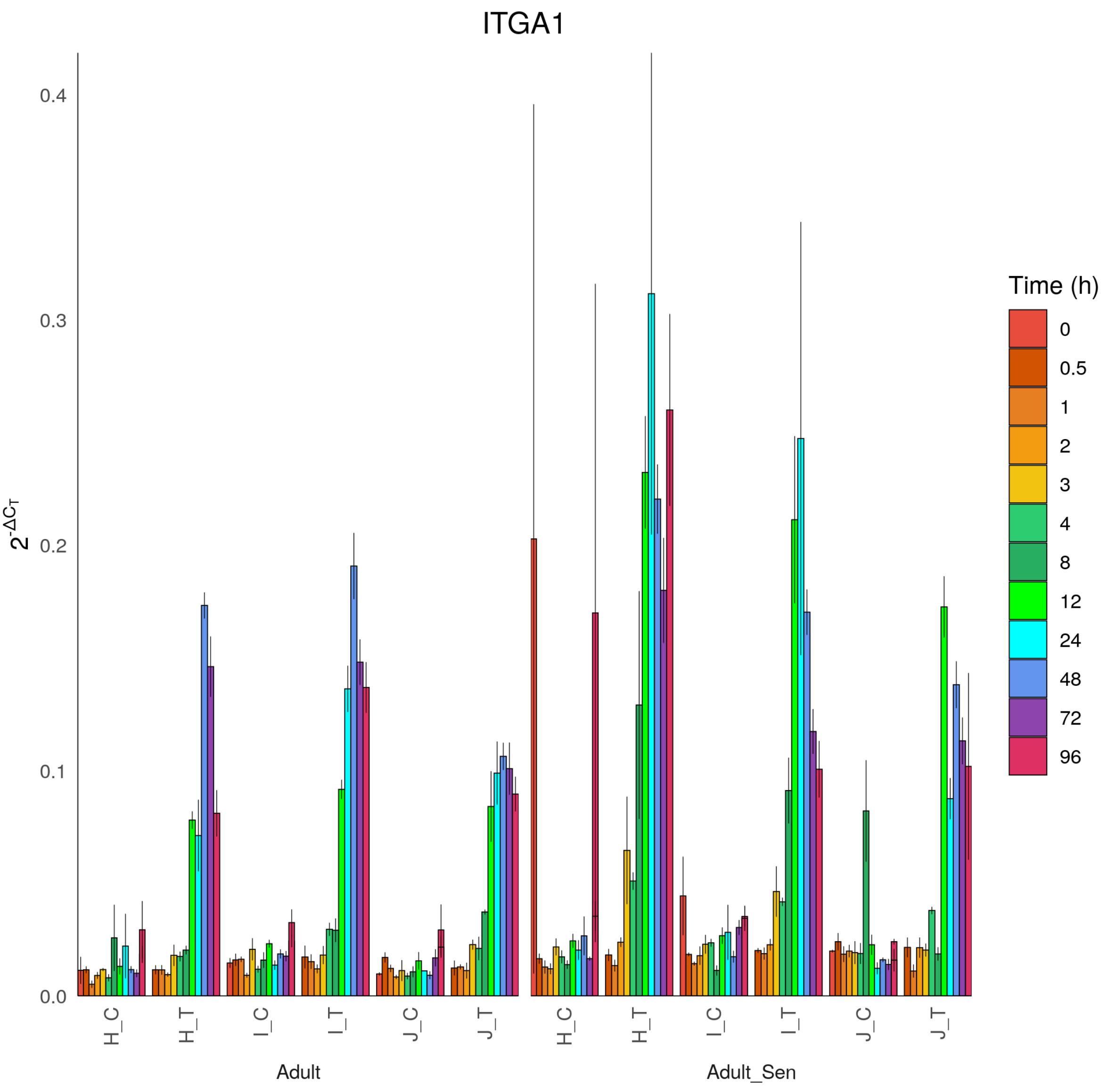
ITGA1
$2^{-\Delta C_T}$
0.0
0.1
0.2
0.3
0.4
H_C
H_T
I_C
I_T
J_C
J_T
Adult
Adult_Sen
Time (h)
0
0.5
1
2
3
4
8
12
24
48
72
96

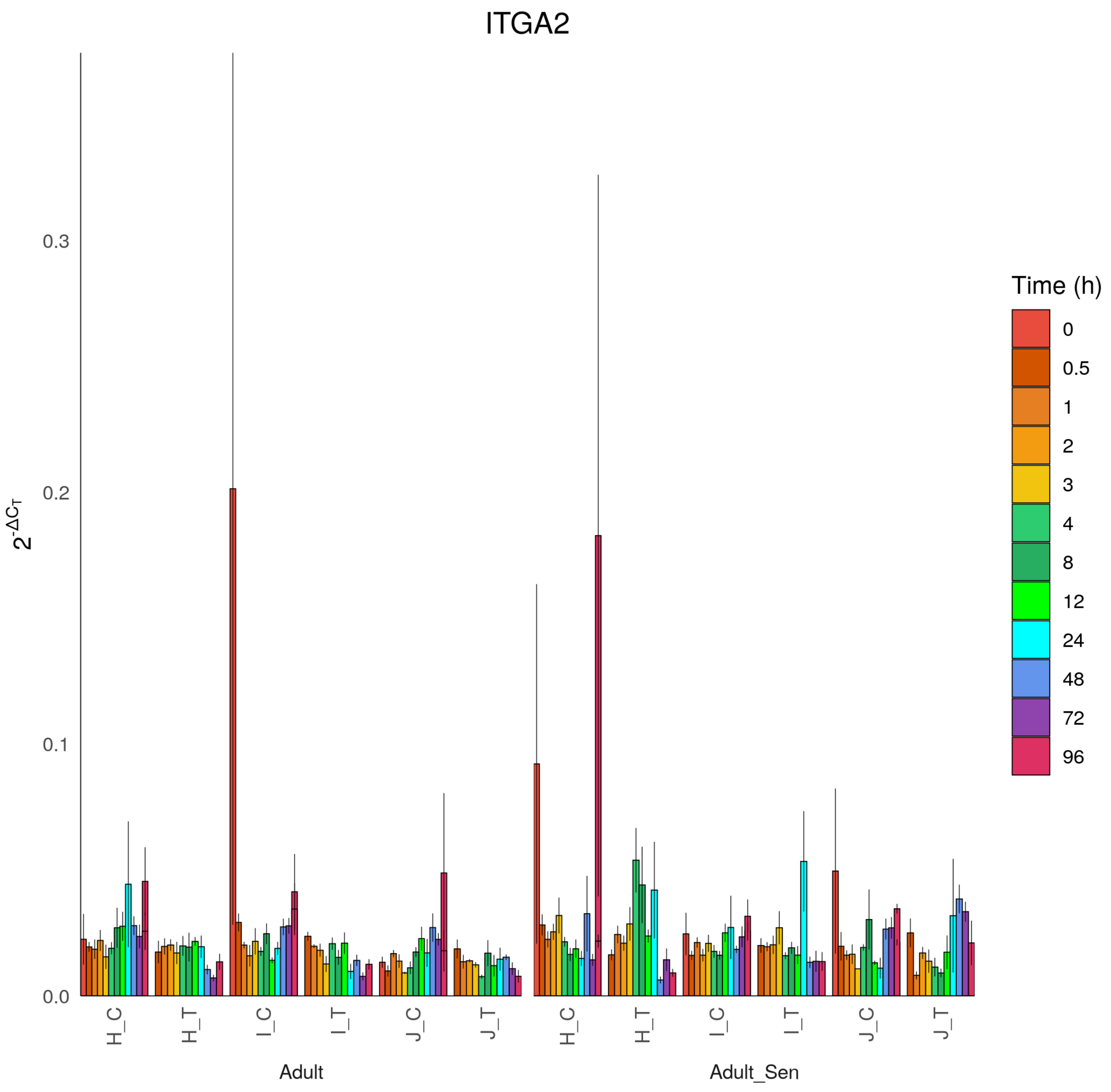
ITGA2
$2^{-\Delta C_T}$
0.0
0.1
0.2
0.3
H_C
H_T
I_C
I_T
J_C
J_T
Adult
Adult_Sen
Time (h)
0
0.5
1
2
3
4
8
12
24
48
72
96

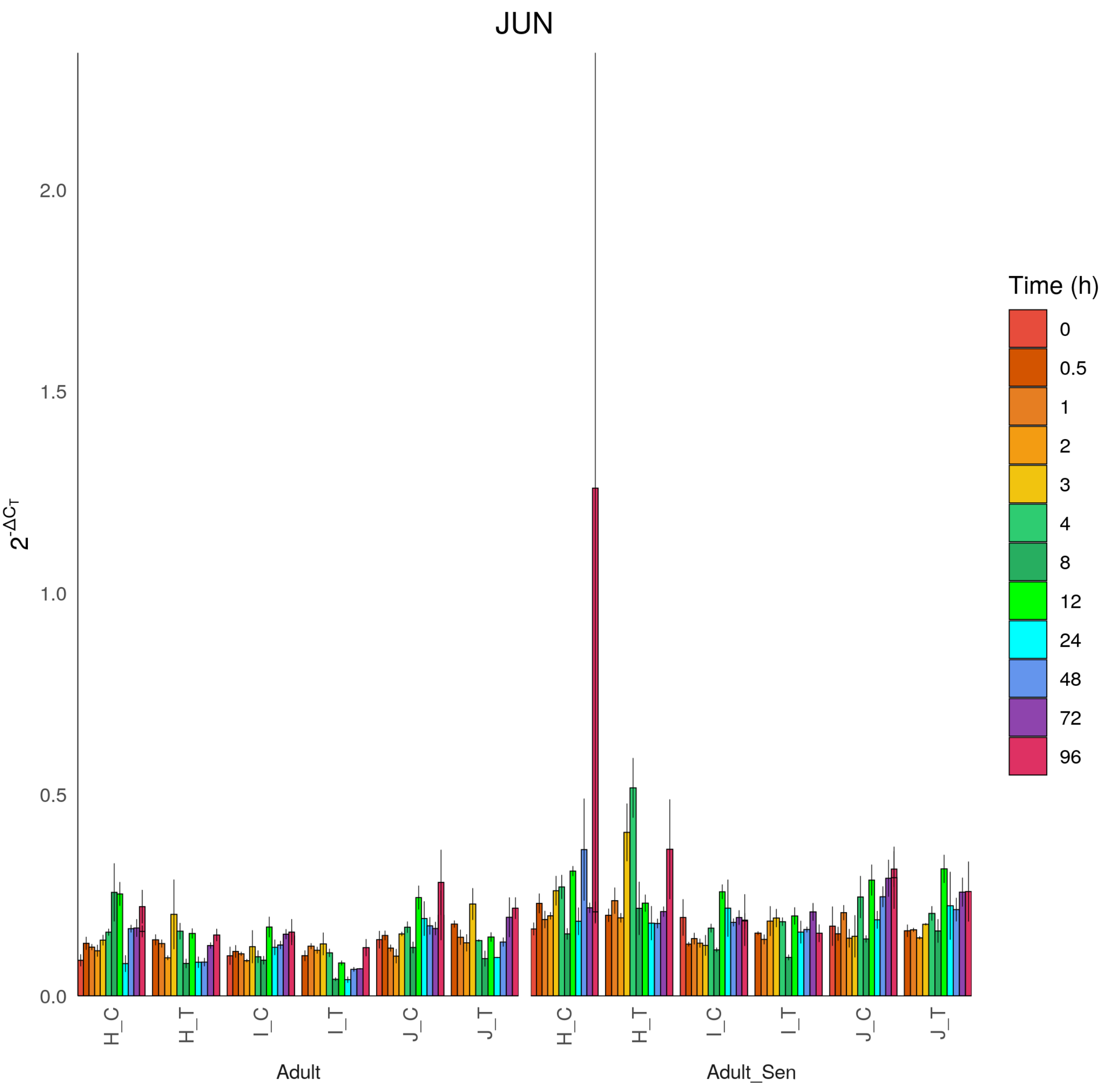
JUN
2.0
1.5
1.0
0.5
0.0
$2^{-\Delta C_T}$
H_C
H_T
I_C
I_T
J_C
J_T
Adult
Adult_Sen
Time (h)
0
0.5
1
2
3
4
8
12
24
48
72
96

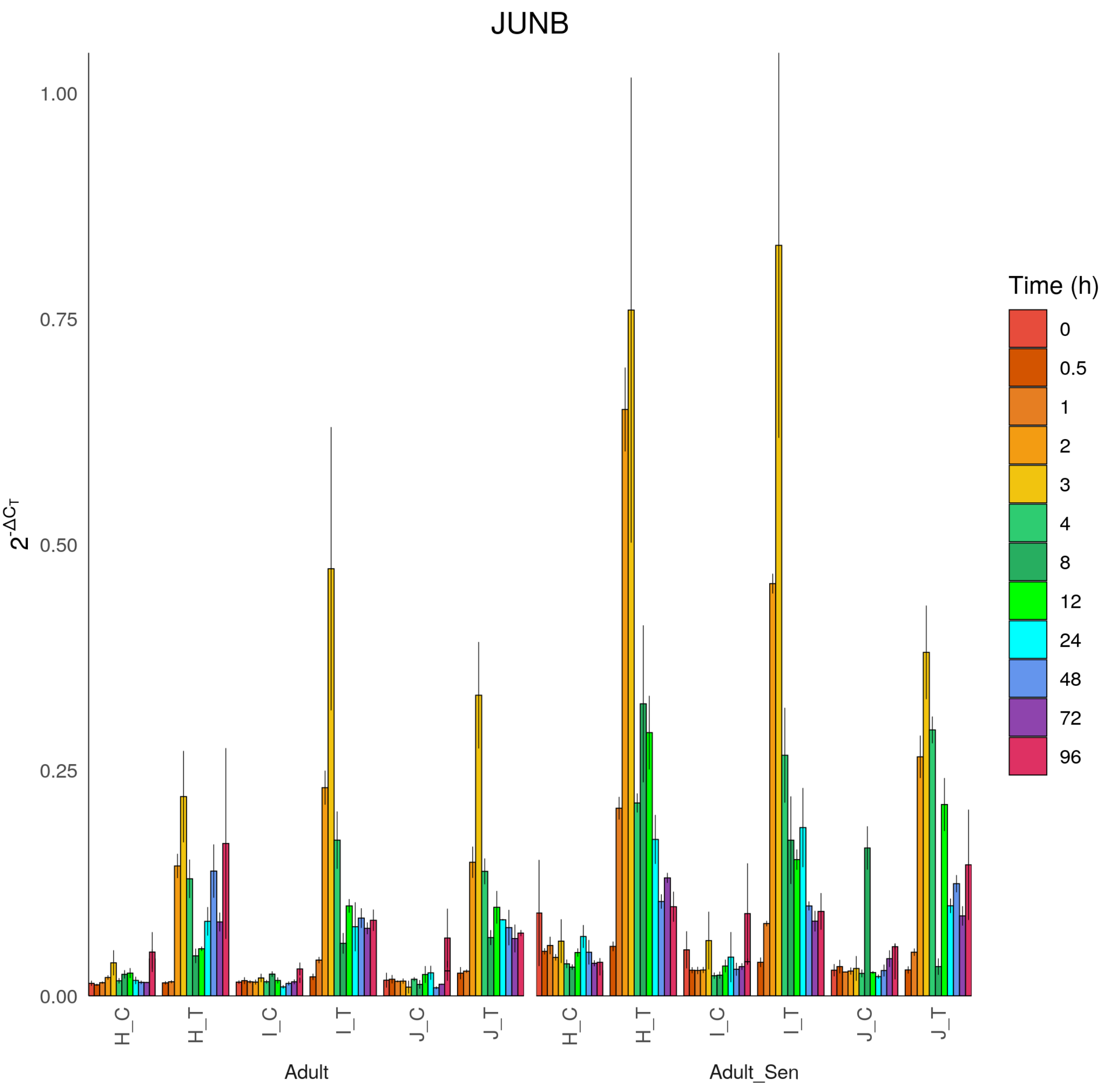
JUNB
$2^{-\Delta C_T}$
1.00
0.75
0.50
0.25
0.00
H_C
H_T
I_C
I_T
J_C
J_T
Adult
Adult_Sen
Time (h)
0
0.5
1
2
3
4
8
12
24
48
72
96

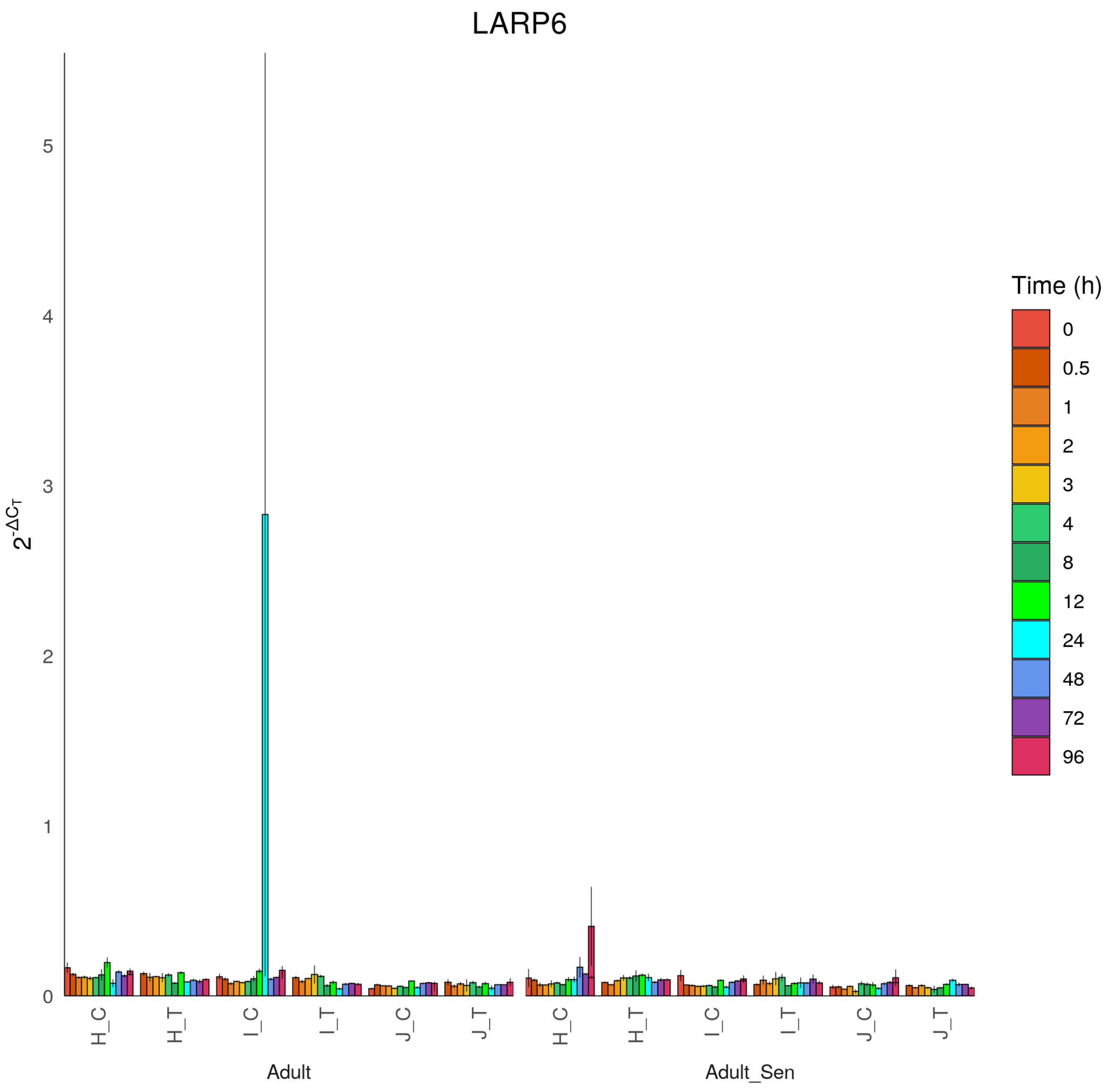

LARP6
2^-ΔCT
0
1
2
3
4
5
H_C
H_T
I_C
I_T
J_C
J_T
Adult
Adult_Sen
Time (h)
0
0.5
1
2
3
4
8
12
24
48
72
96

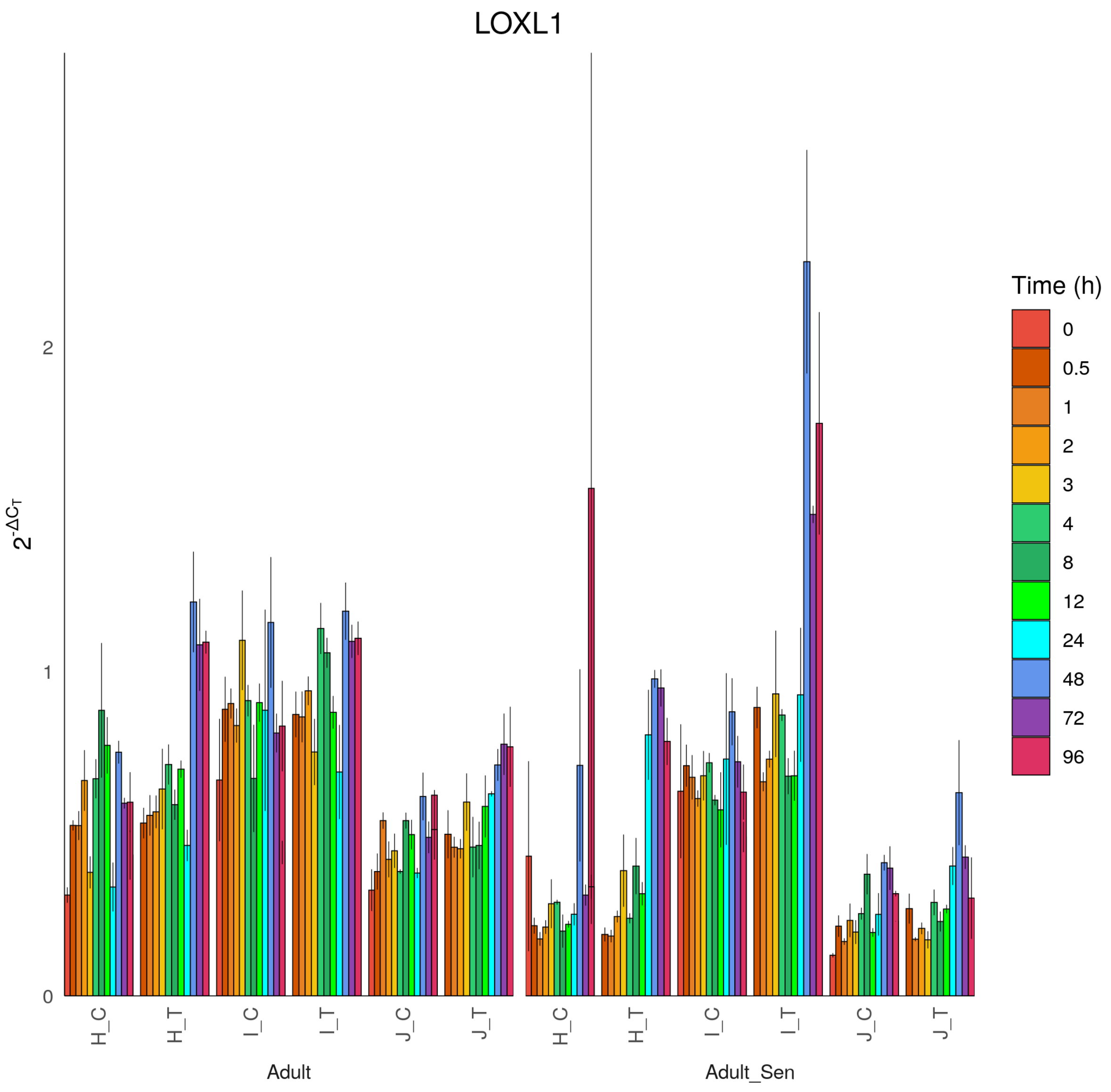

LOXL1
Time (h)
0
0.5
1
2
3
4
8
12
24
48
72
96
$2^{-\Delta C_T}$
0
1
2
H_C
H_T
I_C
I_T
J_C
J_T
Adult
Adult_Sen

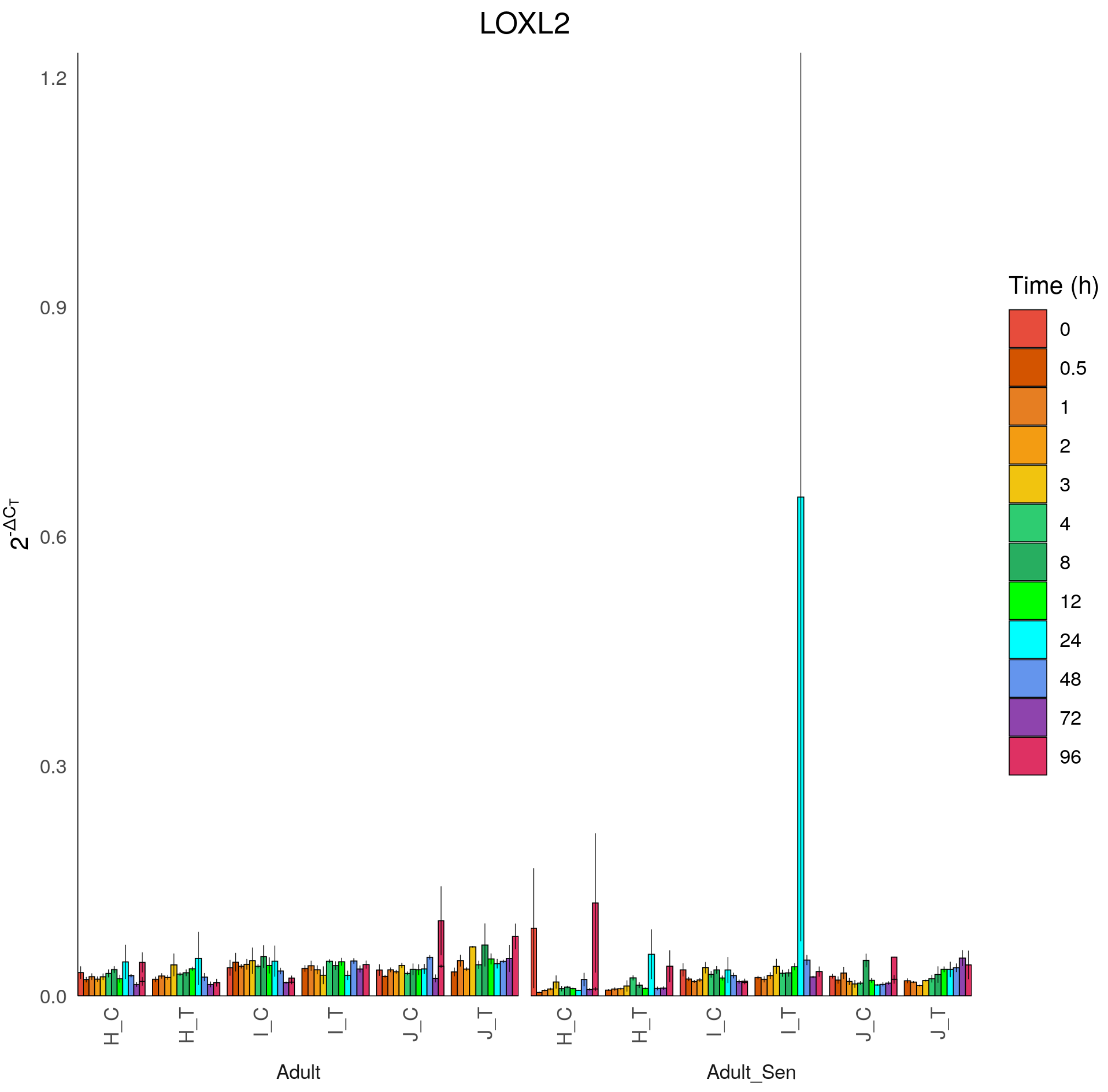

LOXL2
Time (h)
0
0.5
1
2
3
4
8
12
24
48
72
96
$2^{-\Delta C_T}$
1.2
0.9
0.6
0.3
0.0
H_C
H_T
I_C
I_T
J_C
J_T
Adult
H_C
H_T
I_C
I_T
J_C
J_T
Adult_Sen

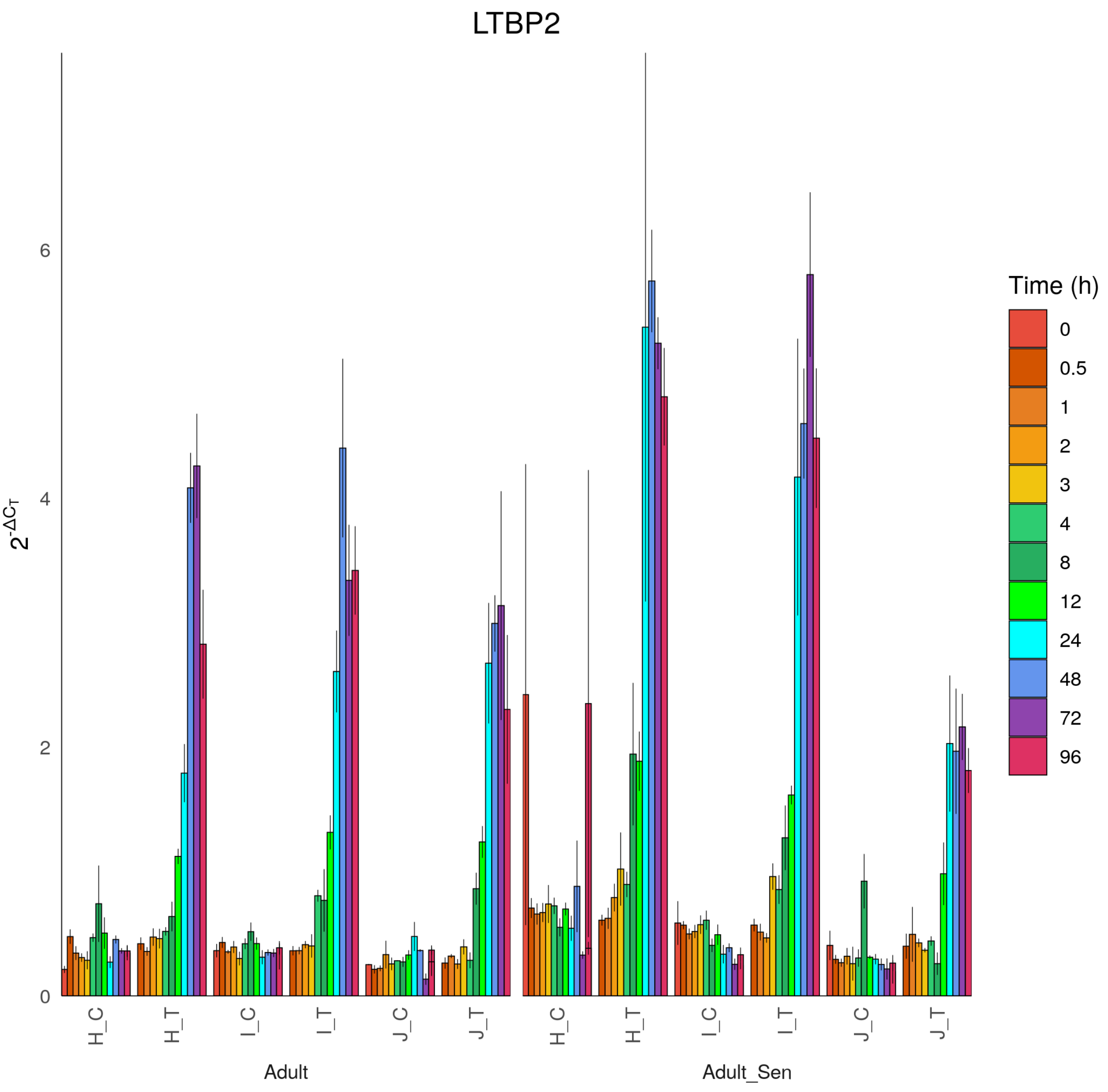

LTBP2
$2^{-\Delta C_T}$
0
2
4
6
H_C
H_T
I_C
I_T
J_C
J_T
Adult
Adult_Sen
Time (h)
0
0.5
1
2
3
4
8
12
24
48
72
96

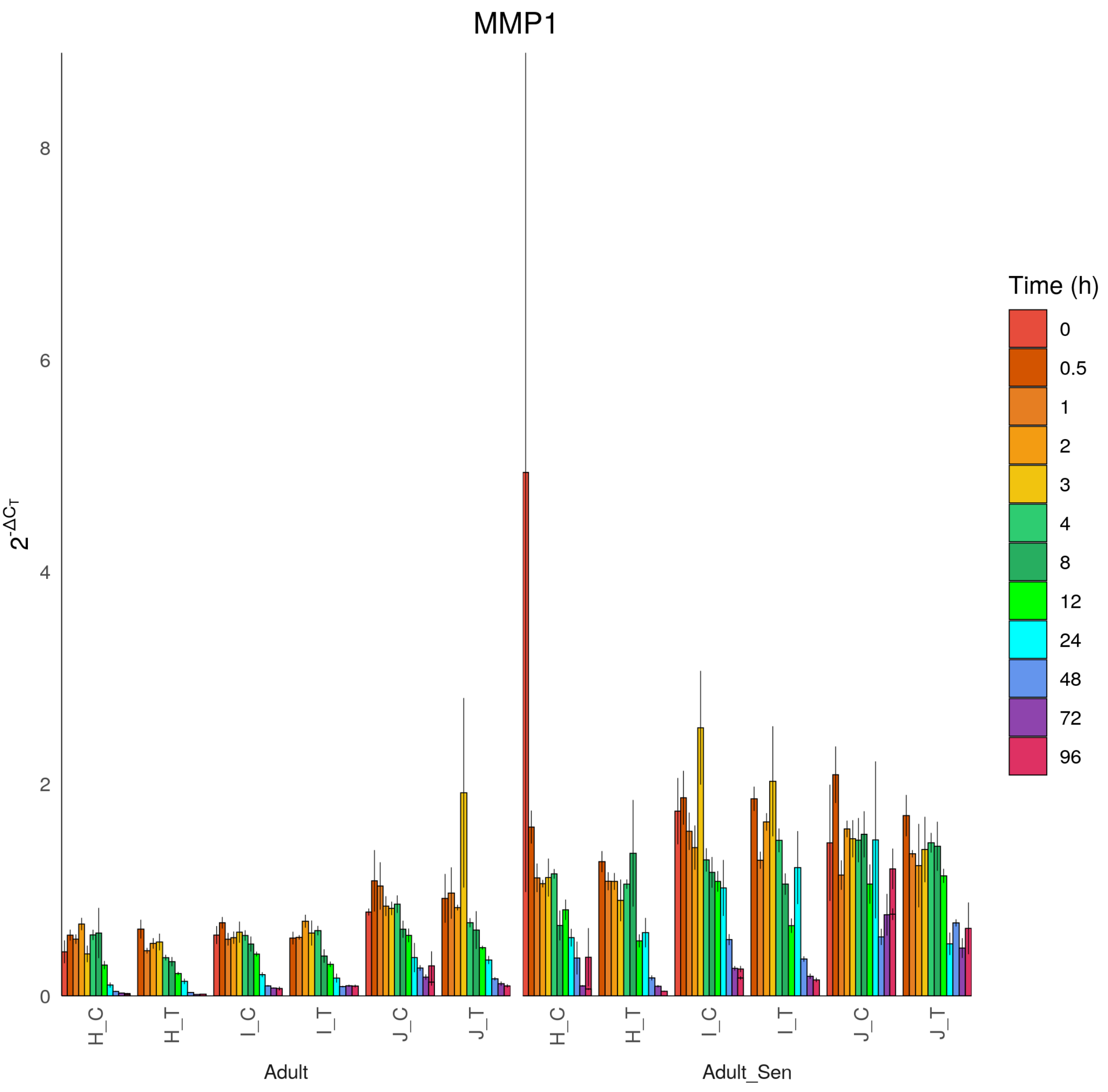
MMP1
$2^{-\Delta C_T}$
0
2
4
6
8
H_C
H_T
I_C
I_T
J_C
J_T
H_C
H_T
I_C
I_T
J_C
J_T
Adult
Adult_Sen
Time (h)
0
0.5
1
2
3
4
8
12
24
48
72
96

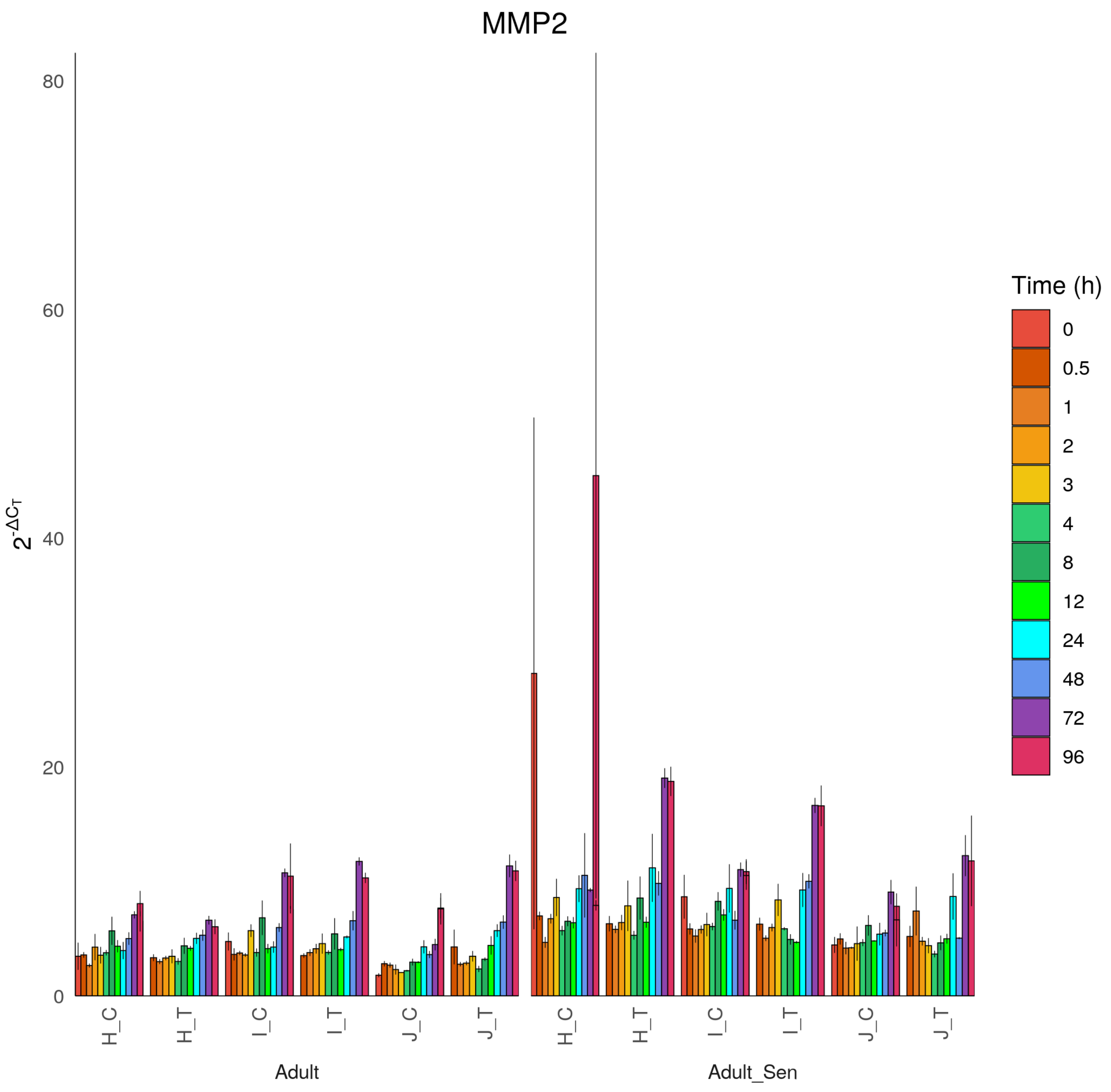
MMP2
$2^{-\Delta C_T}$
0
20
40
60
80
H_C
H_T
I_C
I_T
J_C
J_T
Adult
Adult_Sen
Time (h)
0
0.5
1
2
3
4
8
12
24
48
72
96

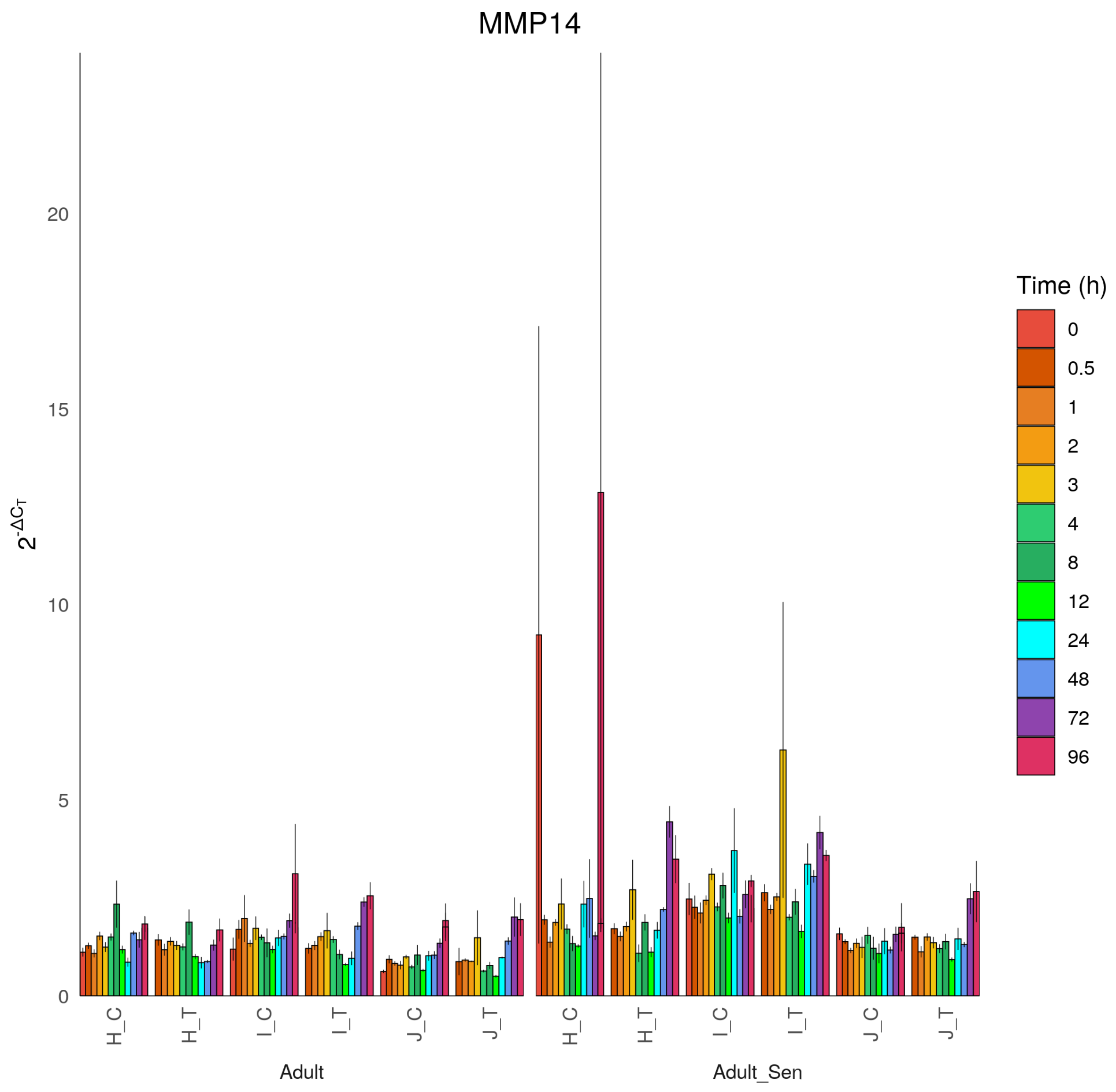

MMP14
$2^{-\Delta C_T}$
0
5
10
15
20
H_C
H_T
I_C
I_T
J_C
J_T
Adult
Adult_Sen
Time (h)
0
0.5
1
2
3
4
8
12
24
48
72
96

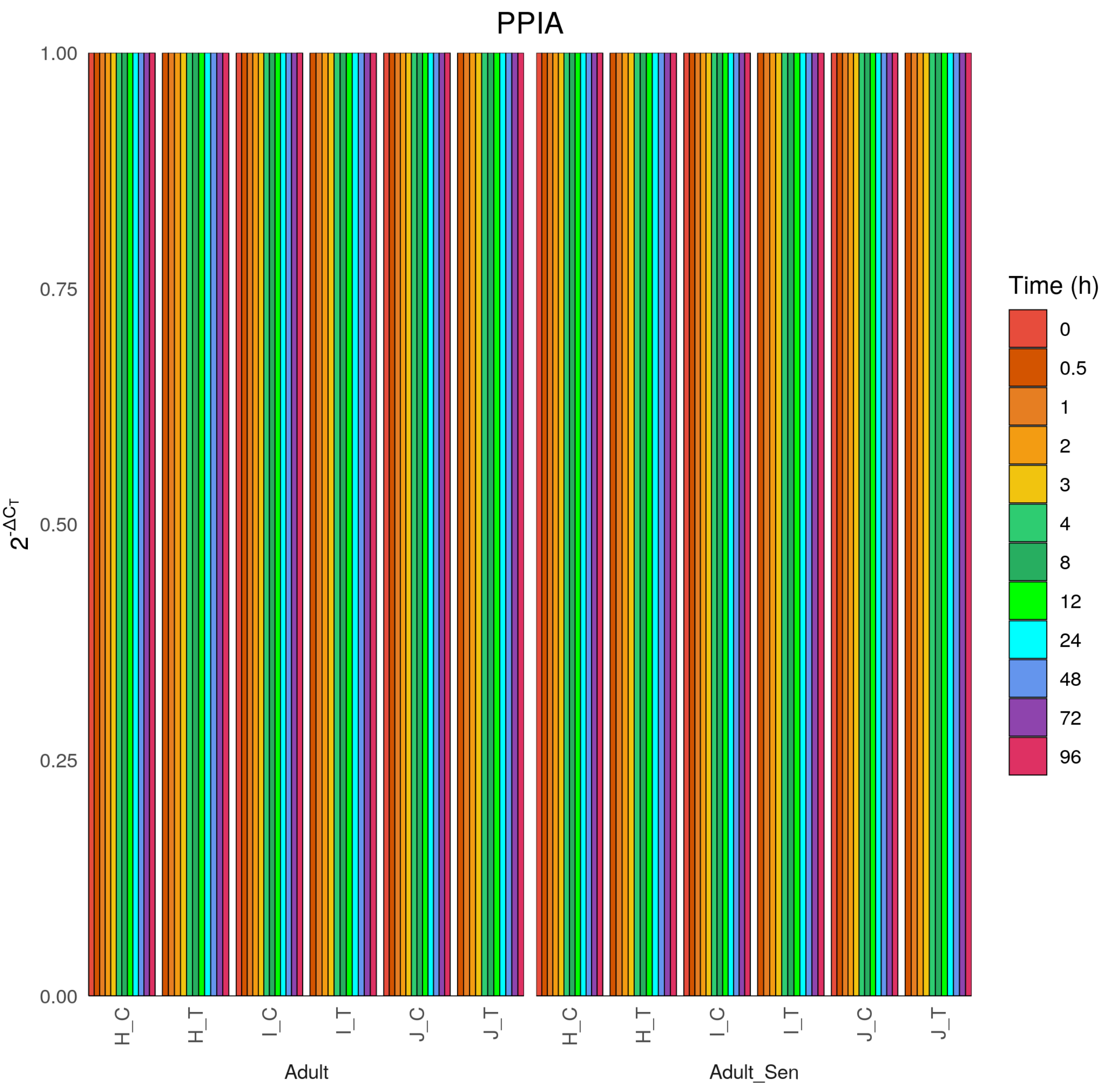

PPIA
1.00
0.75
0.50
0.25
0.00
$2^{-\Delta C_T}$
H_C
H_T
I_C
I_T
J_C
J_T
H_C
H_T
I_C
I_T
J_C
J_T
Adult
Adult_Sen
Time (h)
0
0.5
1
2
3
4
8
12
24
48
72
96

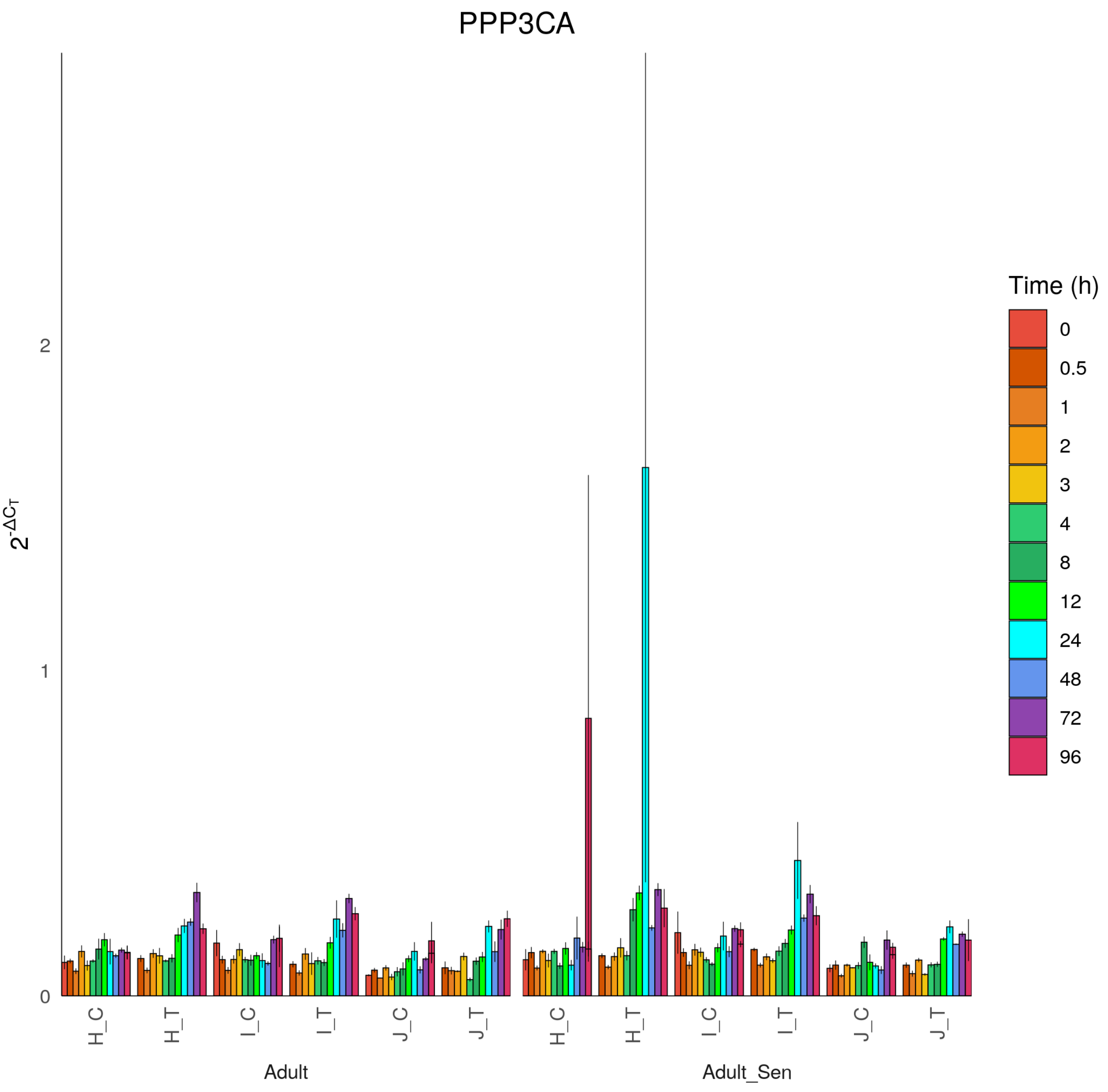
PPP3CA
2
1
0
$2^{-\Delta C_T}$
H_C
H_T
I_C
I_T
J_C
J_T
H_C
H_T
I_C
I_T
J_C
J_T
Adult
Adult_Sen
Time (h)
0
0.5
1
2
3
4
8
12
24
48
72
96

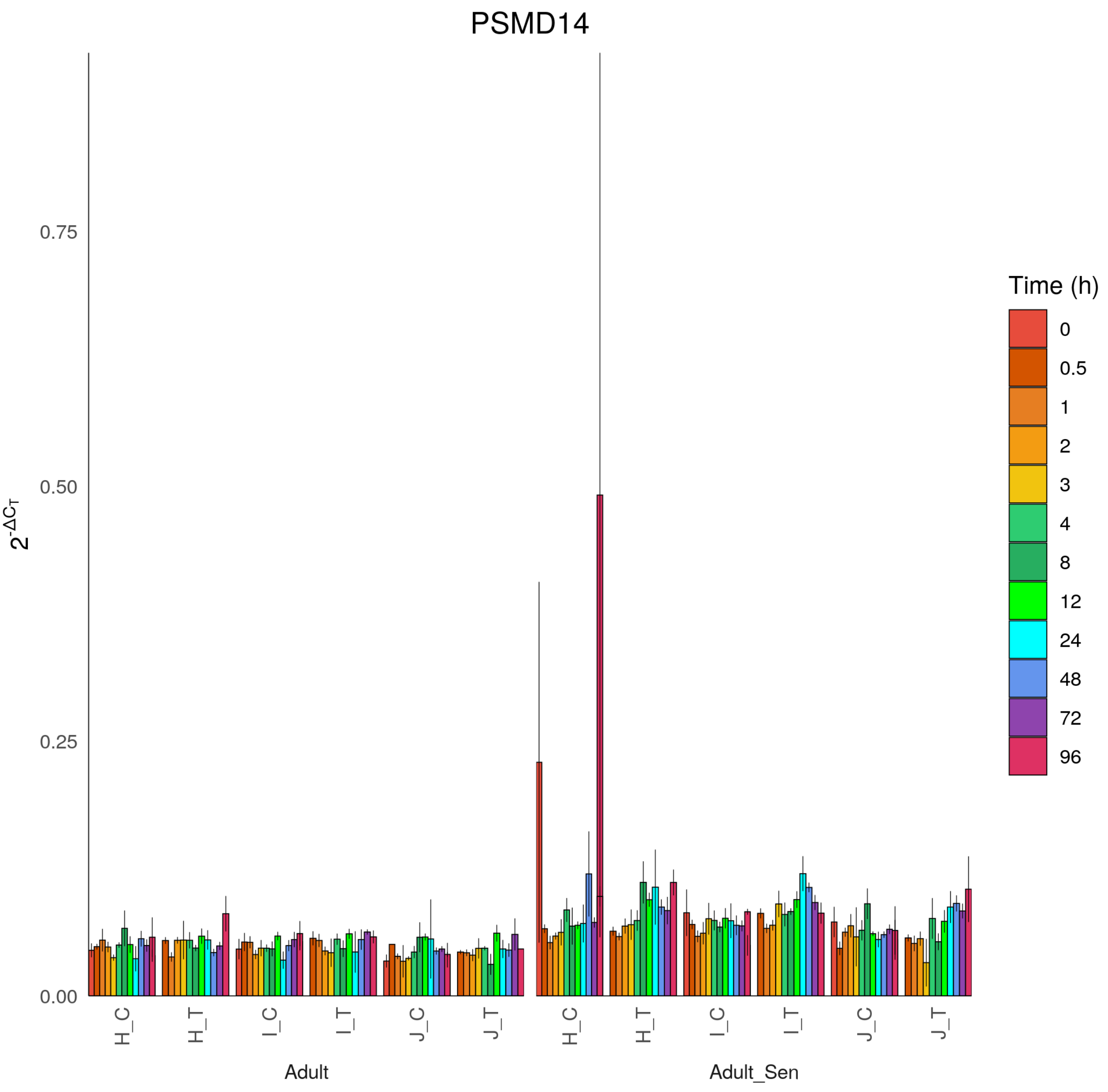

PSMD14
$2^{-\Delta C_T}$
0.00
0.25
0.50
0.75
H_C
H_T
I_C
I_T
J_C
J_T
Adult
Adult_Sen
Time (h)
0
0.5
1
2
3
4
8
12
24
48
72
96

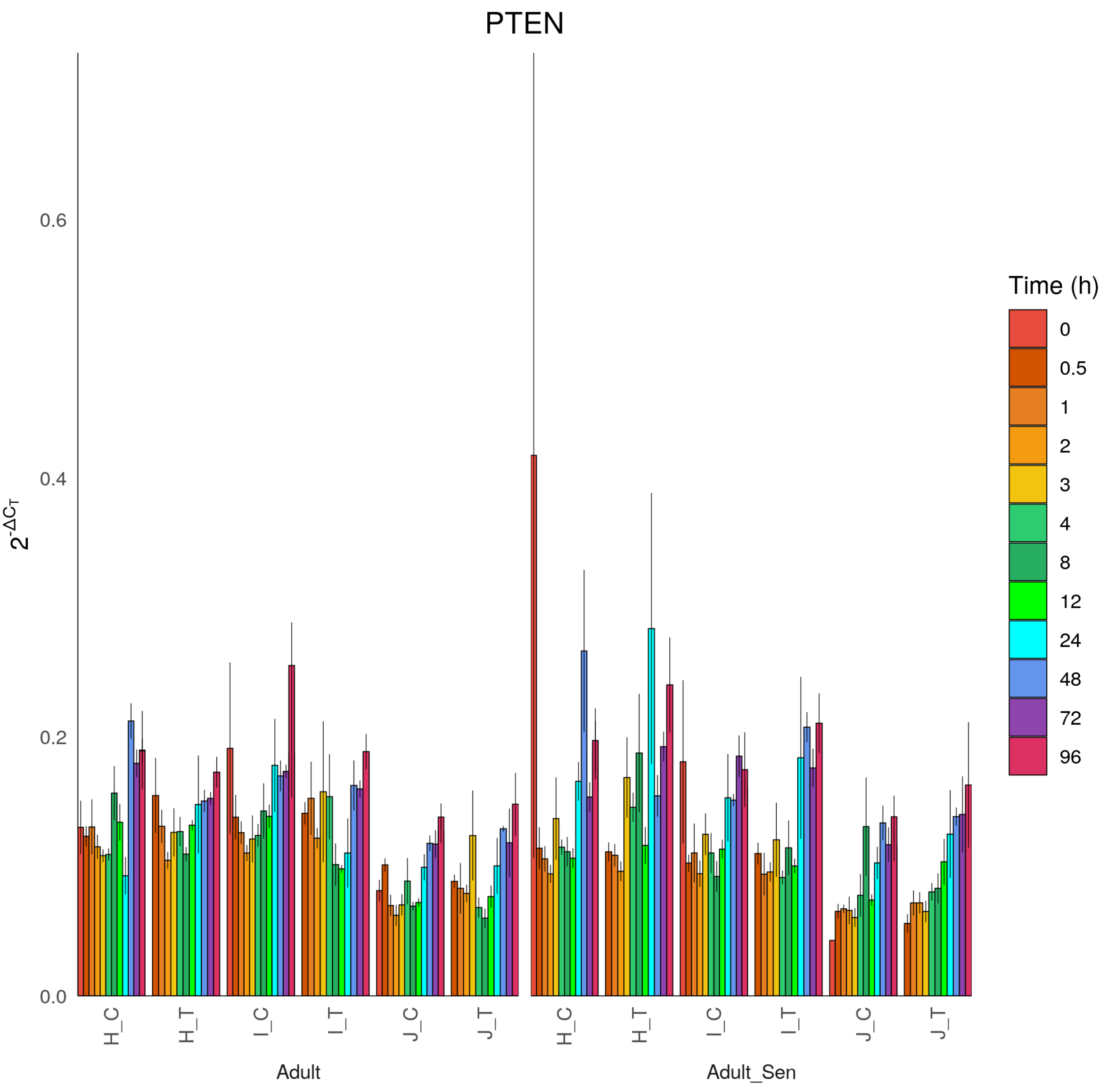

PTEN
$2^{-\Delta C_T}$
0.0
0.2
0.4
0.6
H_C
H_T
I_C
I_T
J_C
J_T
Adult
Adult_Sen
Time (h)
0
0.5
1
2
3
4
8
12
24
48
72
96

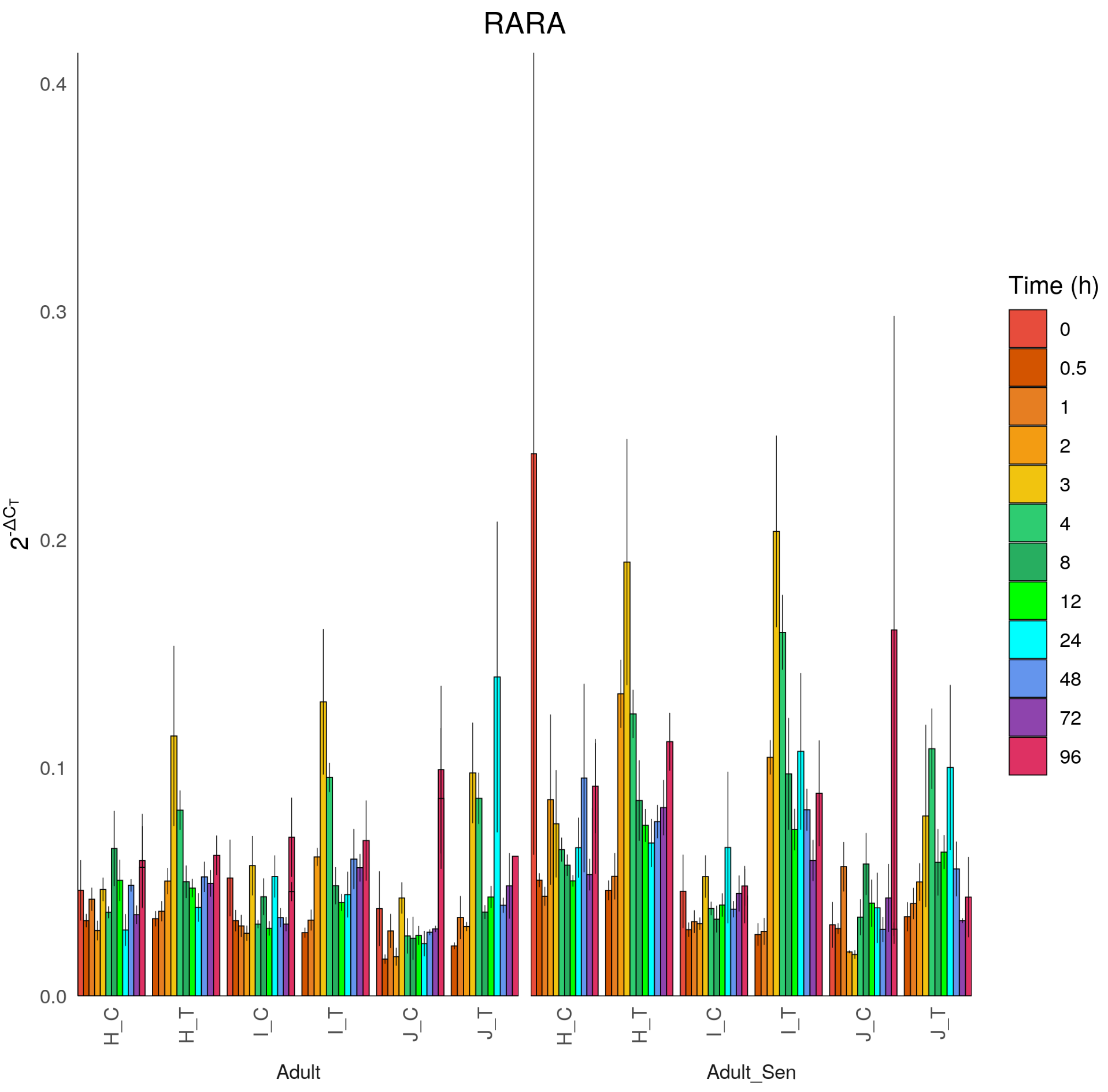

RARA
$2^{-\Delta C_T}$
0.0
0.1
0.2
0.3
0.4
H_C
H_T
I_C
I_T
J_C
J_T
Adult
Adult_Sen
Time (h)
0
0.5
1
2
3
4
8
12
24
48
72
96

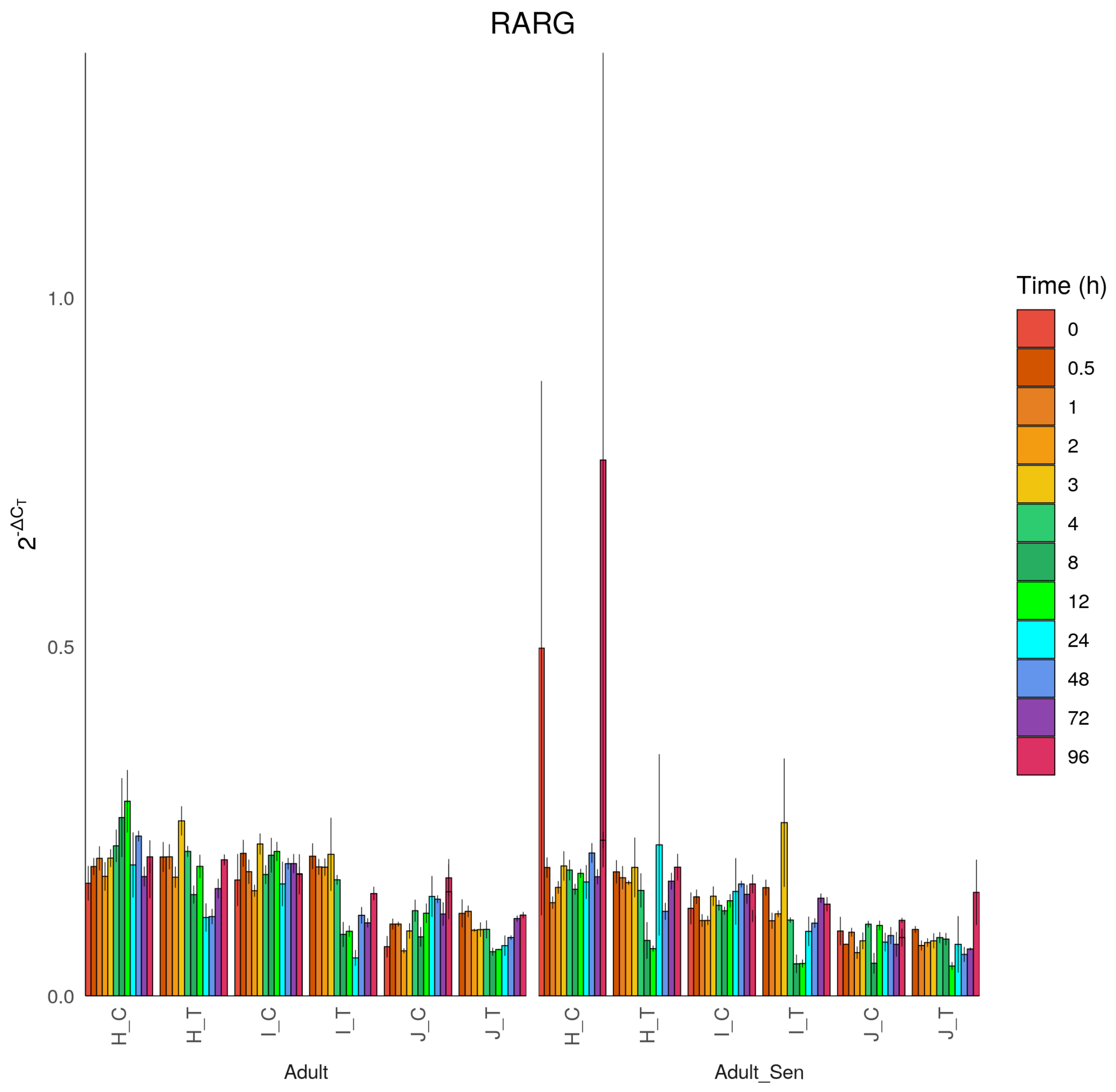
RARG
$2^{-\Delta C_T}$
1.0
0.5
0.0
H_C
H_T
I_C
I_T
J_C
J_T
Adult
Adult_Sen
Time (h)
0
0.5
1
2
3
4
8
12
24
48
72
96

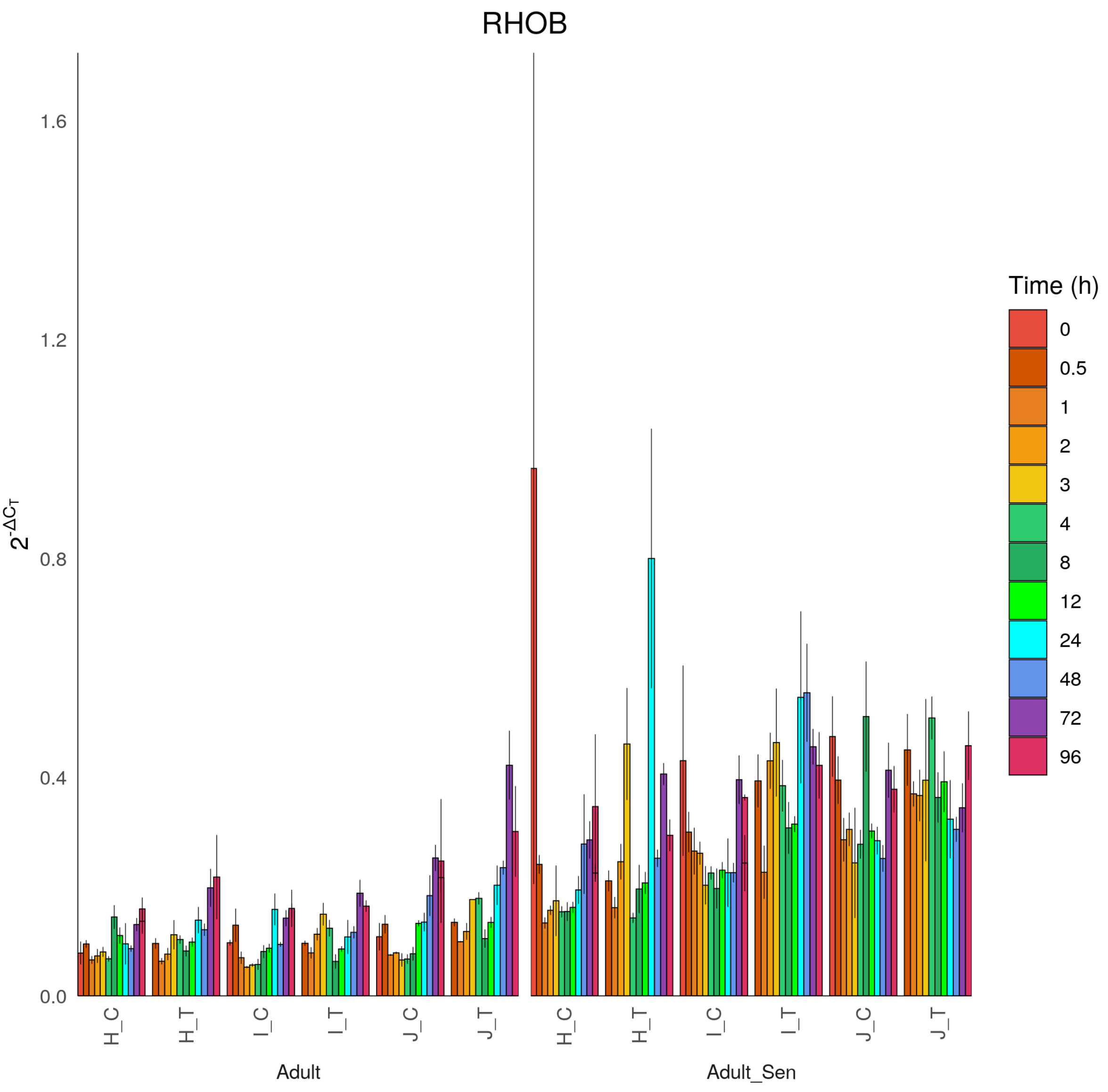

RHOB
$2^{-\Delta C_T}$
0.0
0.4
0.8
1.2
1.6
H_C
H_T
I_C
I_T
J_C
J_T
Adult
Adult_Sen
Time (h)
0
0.5
1
2
3
4
8
12
24
48
72
96

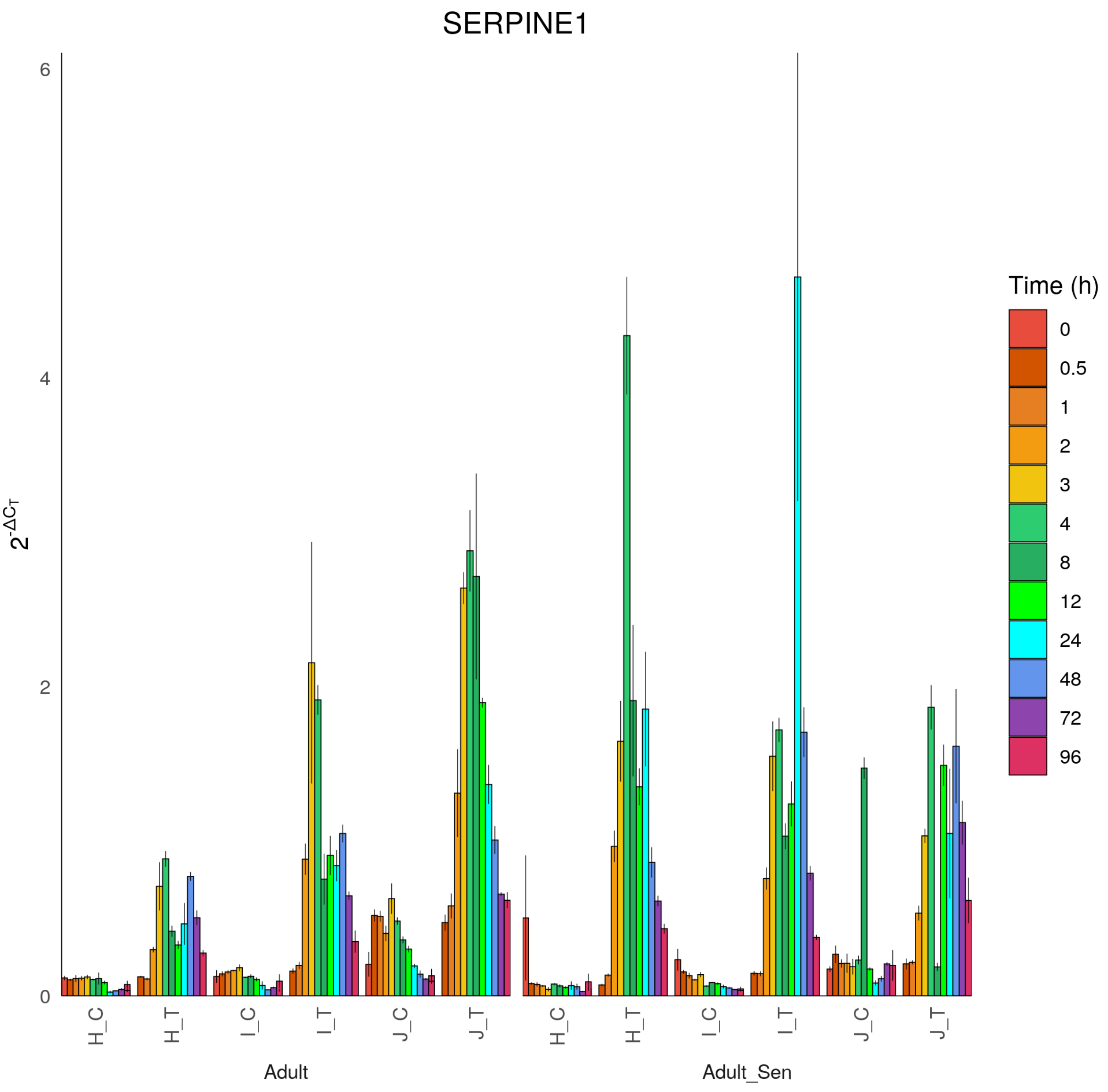
SERPINE1
$2^{-\Delta C_T}$
6
4
2
0
H_C
H_T
I_C
I_T
J_C
J_T
Adult
Adult_Sen
Time (h)
0
0.5
1
2
3
4
8
12
24
48
72
96

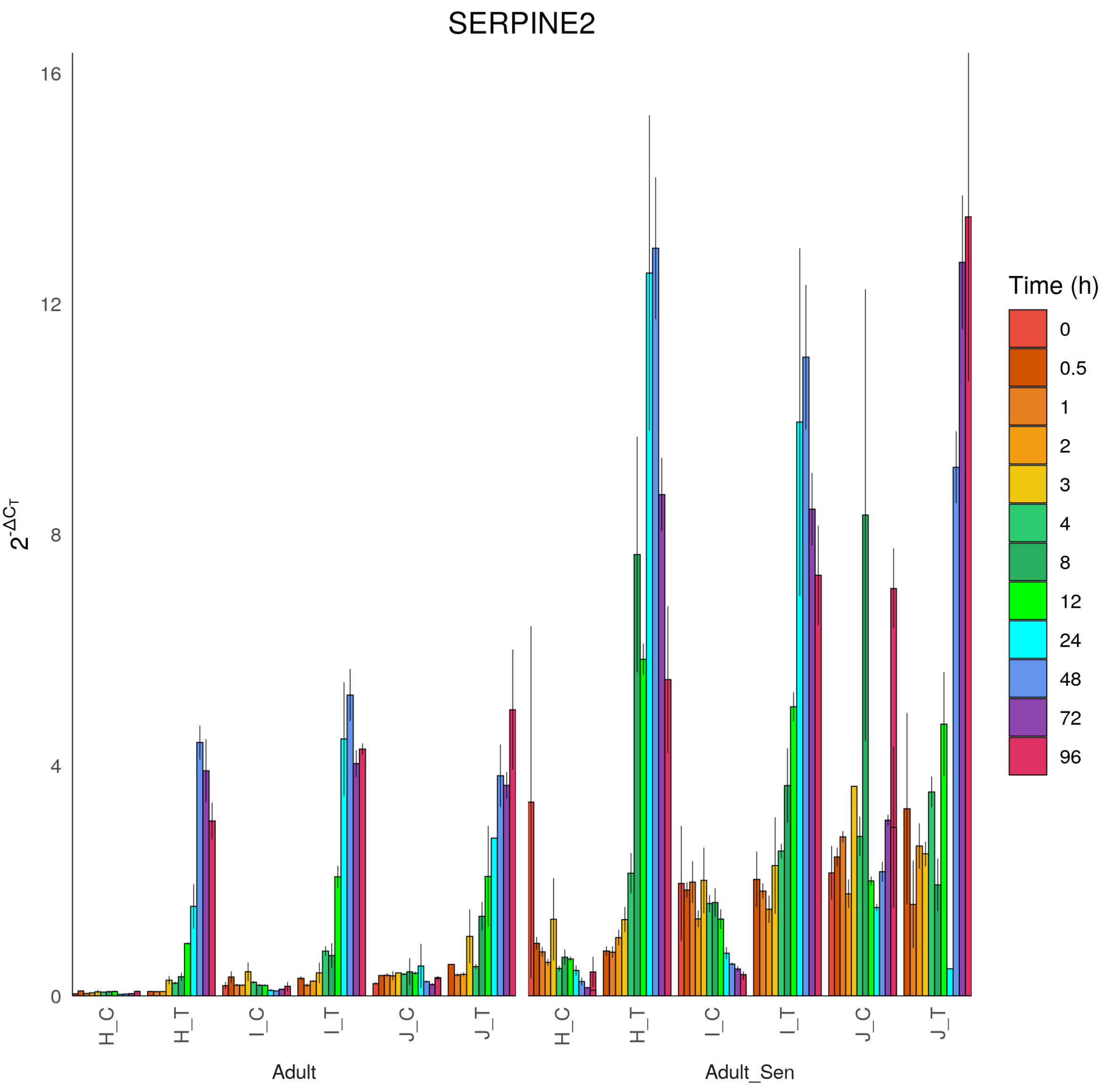

SERPINE2
2^-ΔCT
16
12
8
4
0
H_C
H_T
I_C
I_T
J_C
J_T
Adult
Adult_Sen
Time (h)
0
0.5
1
2
3
4
8
12
24
48
72
96

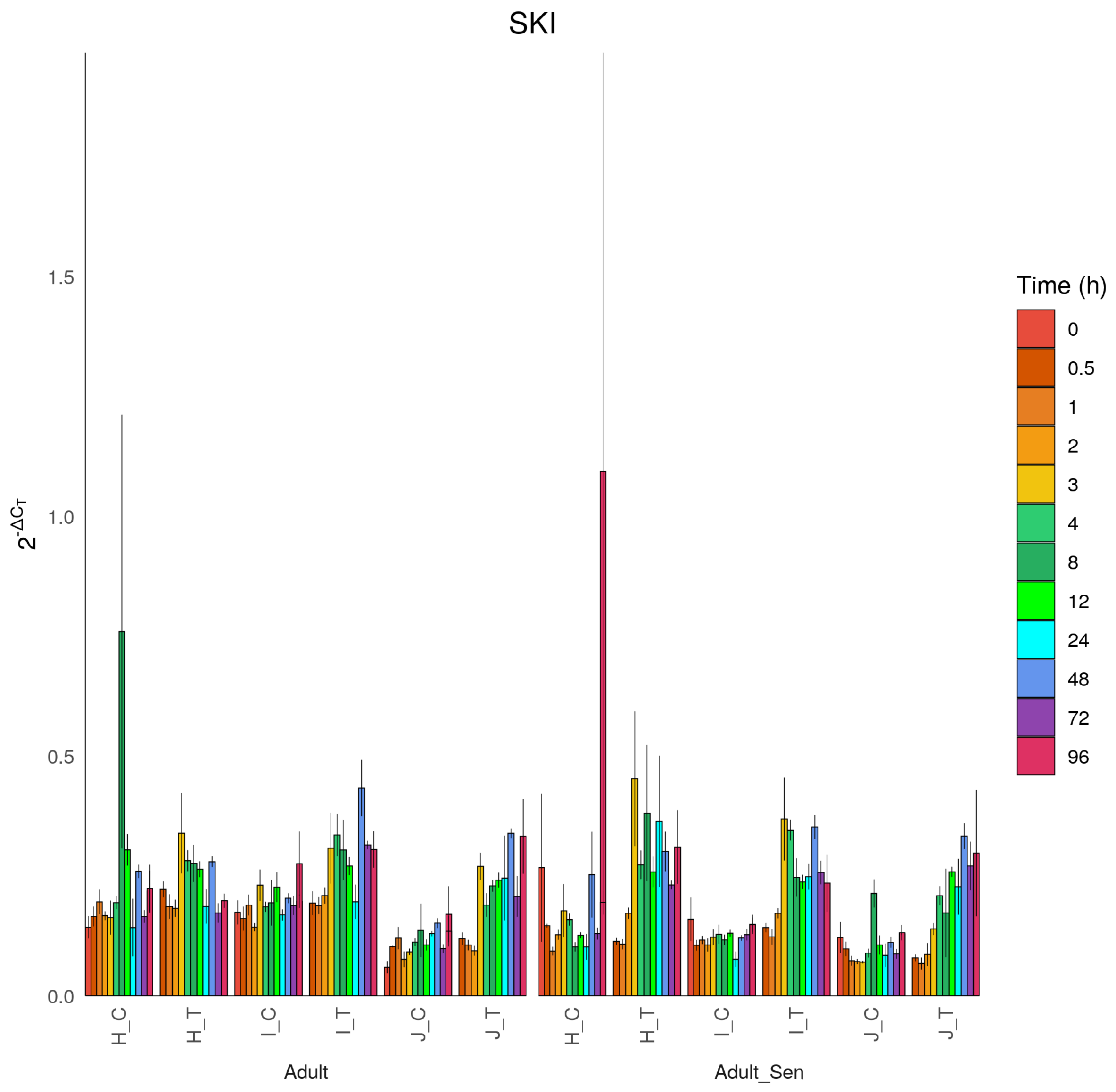
SKI
$2^{-\Delta C_T}$
0.0
0.5
1.0
1.5
H_C
H_T
I_C
I_T
J_C
J_T
Adult
Adult_Sen
Time (h)
0
0.5
1
2
3
4
8
12
24
48
72
96

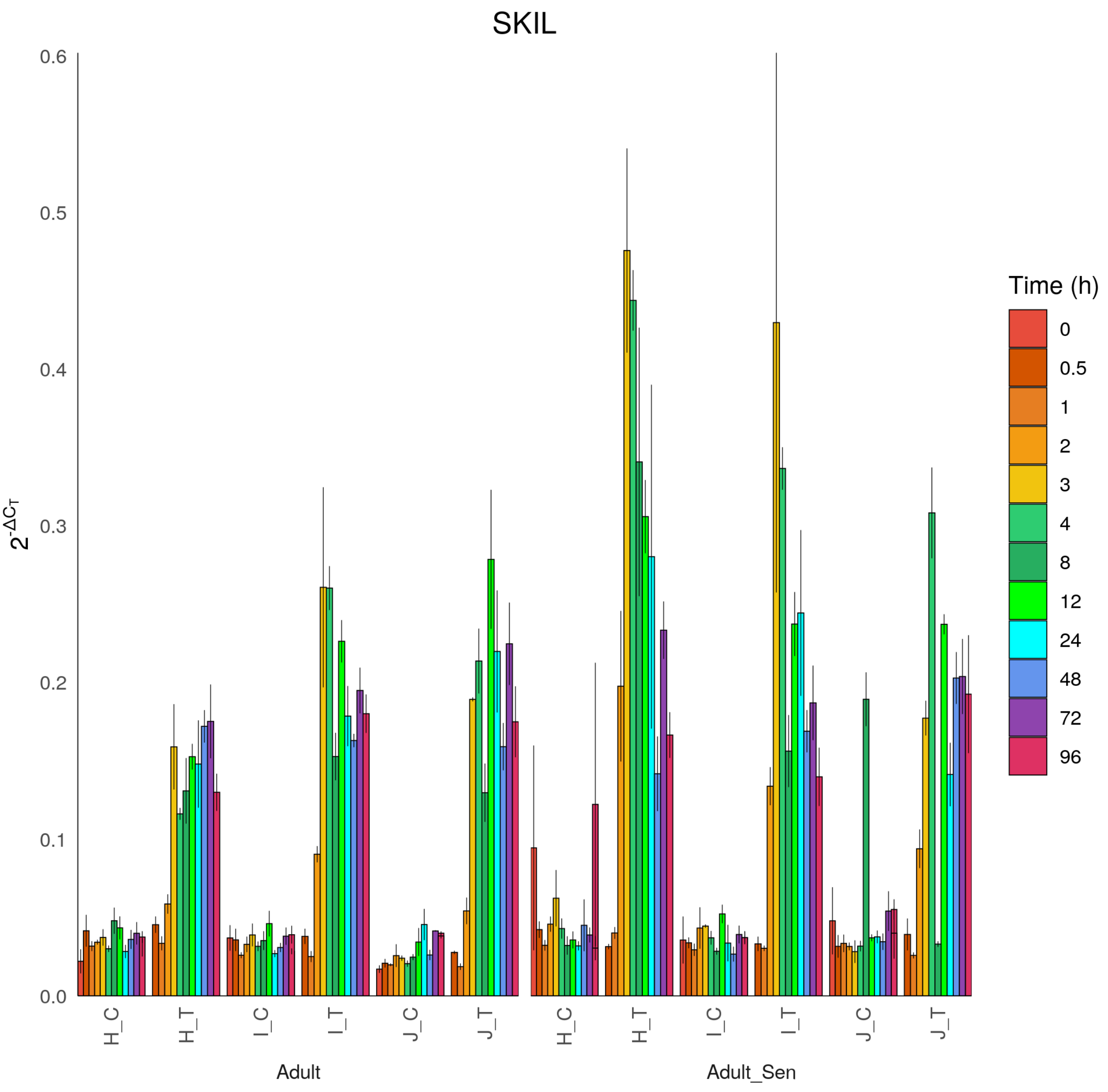

SKIL
$2^{-\Delta C_T}$
0.0
0.1
0.2
0.3
0.4
0.5
0.6
H_C
H_T
I_C
I_T
J_C
J_T
Adult
Adult_Sen
Time (h)
0
0.5
1
2
3
4
8
12
24
48
72
96

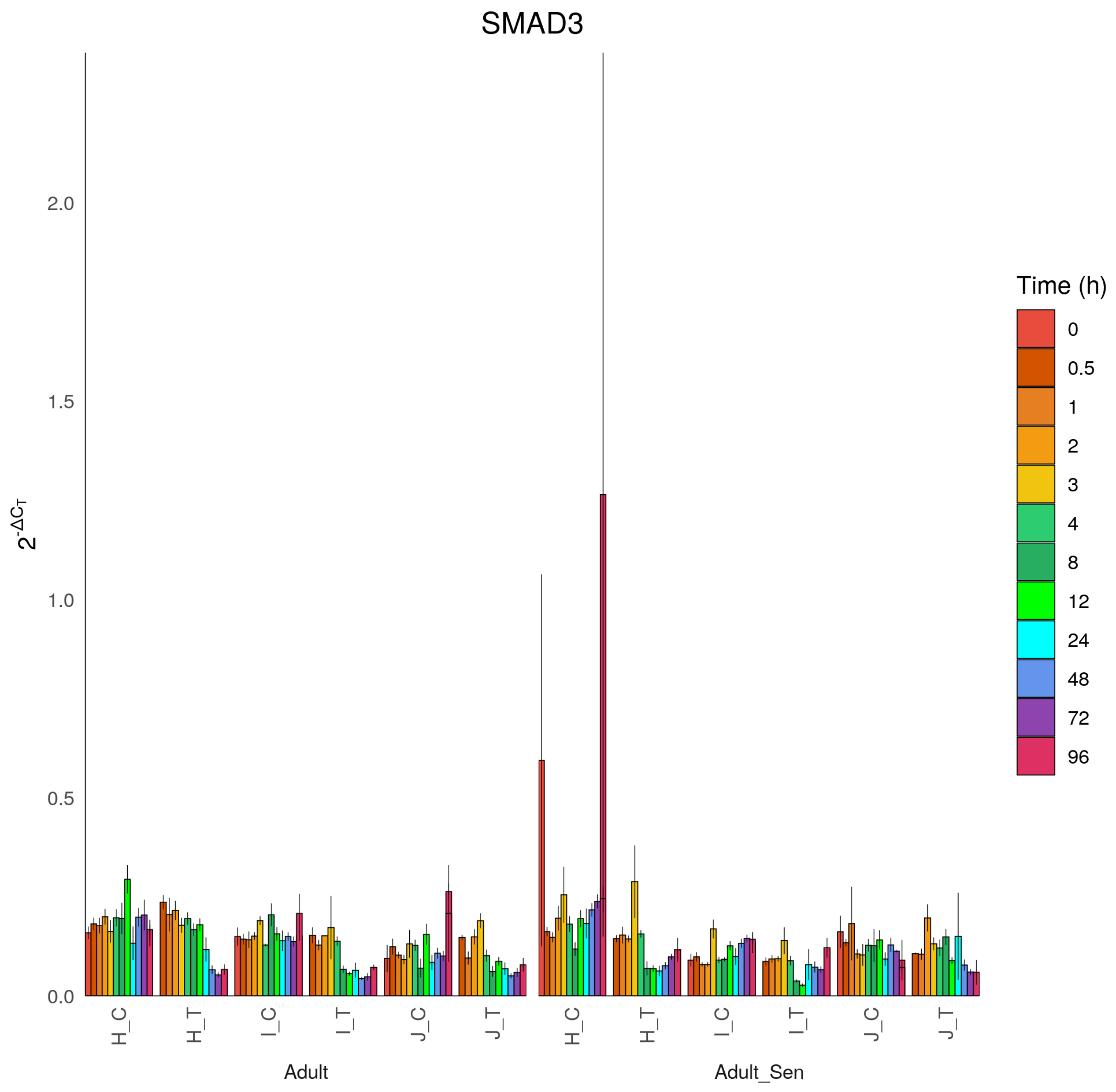

SMAD3
$2^{-\Delta C_T}$
2.0
1.5
1.0
0.5
0.0
H_C
H_T
I_C
I_T
J_C
J_T
Adult
Adult_Sen
Time (h)
0
0.5
1
2
3
4
8
12
24
48
72
96

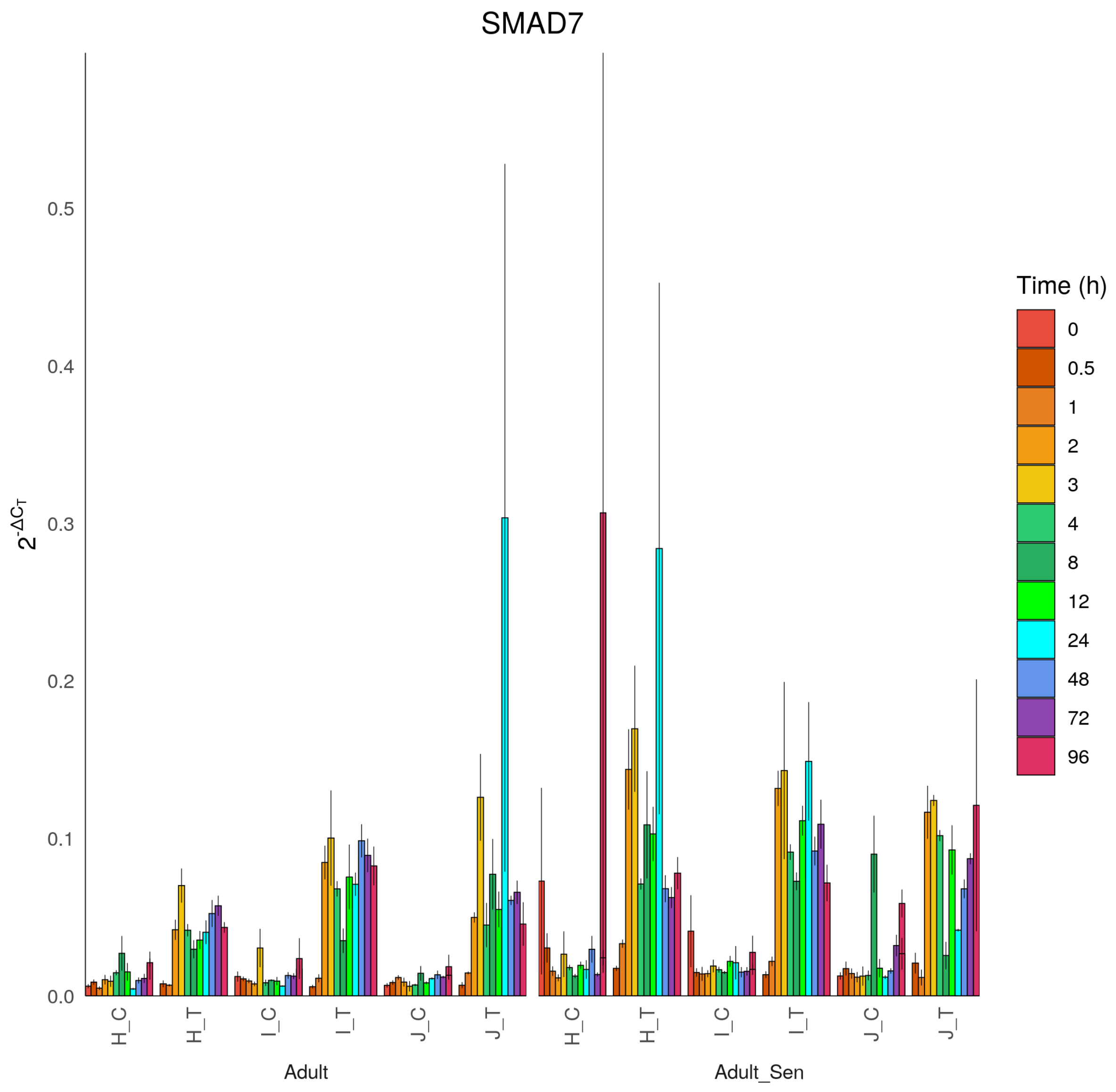

SMAD7
$2^{-\Delta C_T}$
0.0
0.1
0.2
0.3
0.4
0.5
H_C
H_T
I_C
I_T
J_C
J_T
Adult
Adult_Sen
Time (h)
0
0.5
1
2
3
4
8
12
24
48
72
96

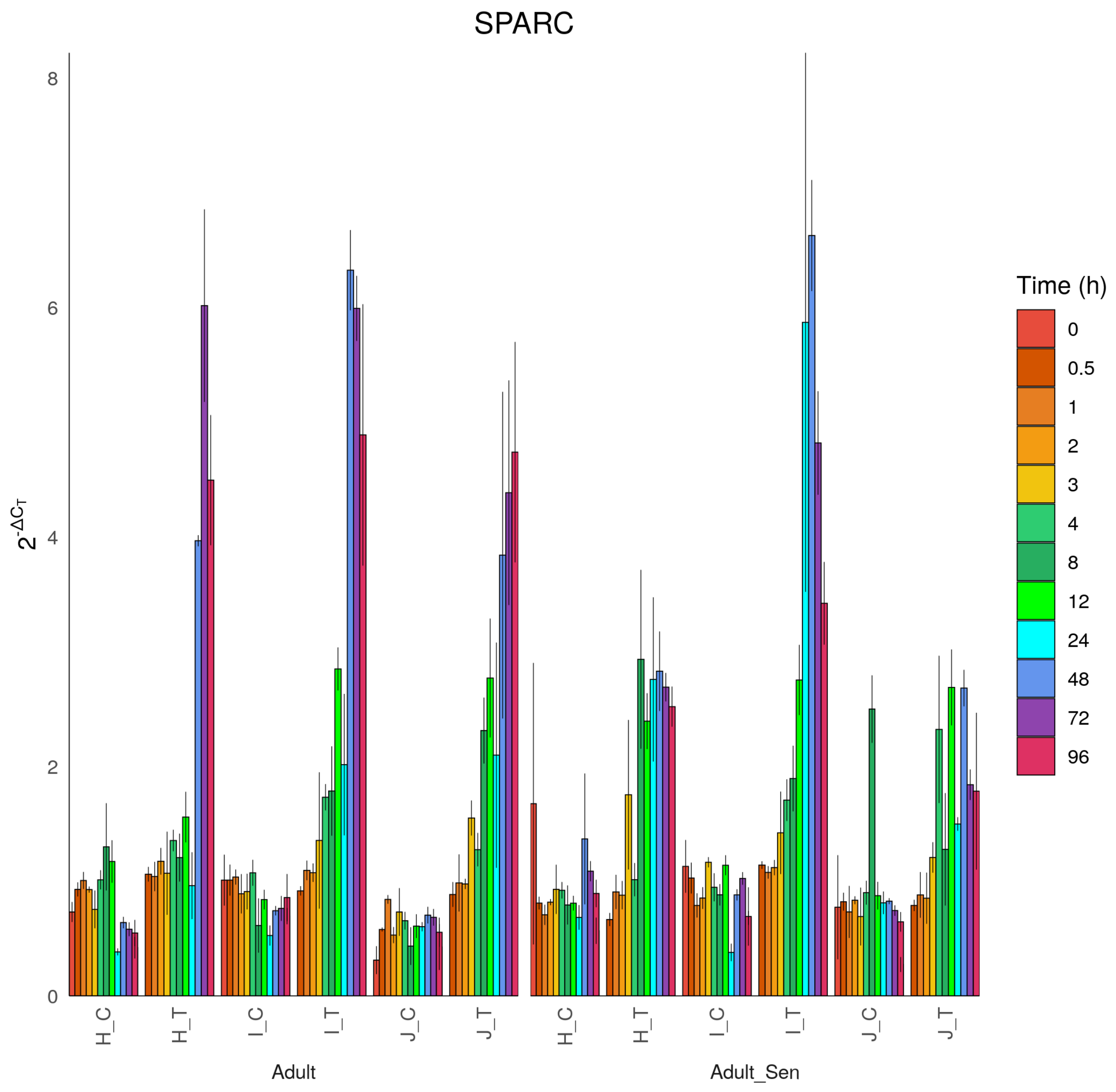

SPARC
$2^{-\Delta C_T}$
0
2
4
6
8
H_C
H_T
I_C
I_T
J_C
J_T
Adult
Adult_Sen
Time (h)
0
0.5
1
2
3
4
8
12
24
48
72
96

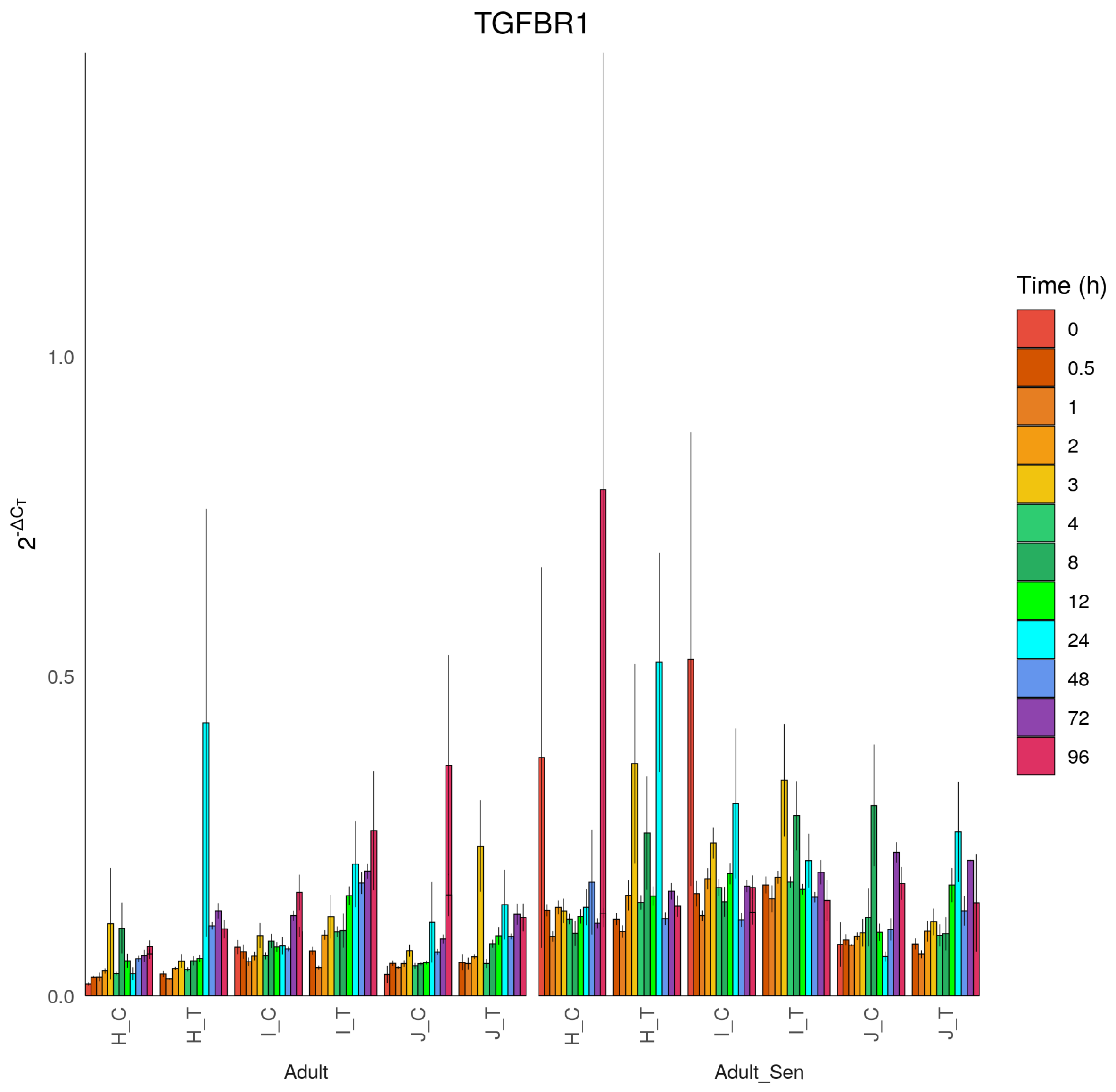

TGFBR1
$2^{-\Delta C_T}$
1.0
0.5
0.0
H_C
H_T
I_C
I_T
J_C
J_T
H_C
H_T
I_C
I_T
J_C
J_T
Adult
Adult_Sen
Time (h)
0
0.5
1
2
3
4
8
12
24
48
72
96

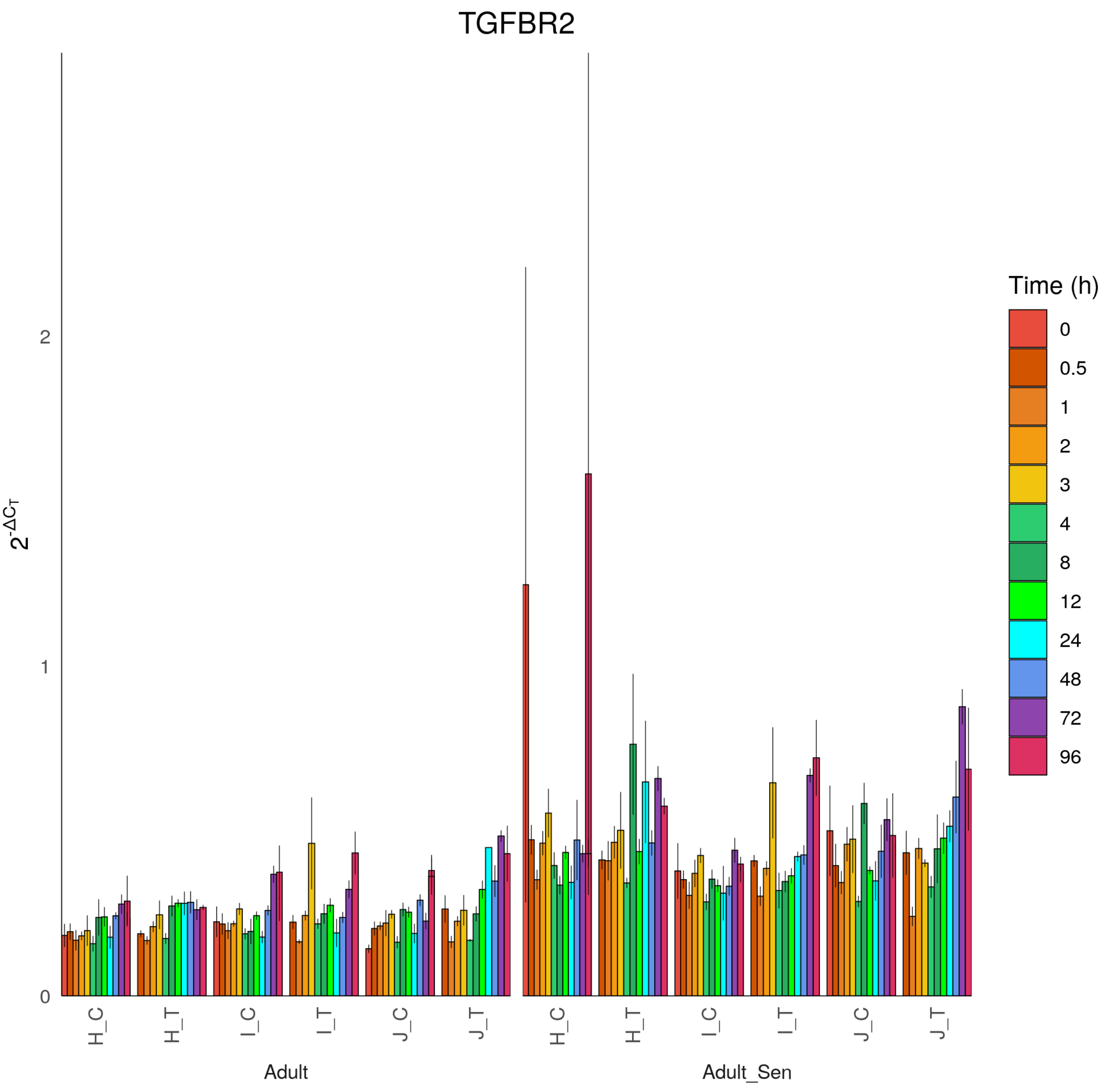
TGFBR2
$2^{-\Delta C_T}$
0
1
2
H_C
H_T
I_C
I_T
J_C
J_T
Adult
Adult_Sen
Time (h)
0
0.5
1
2
3
4
8
12
24
48
72
96

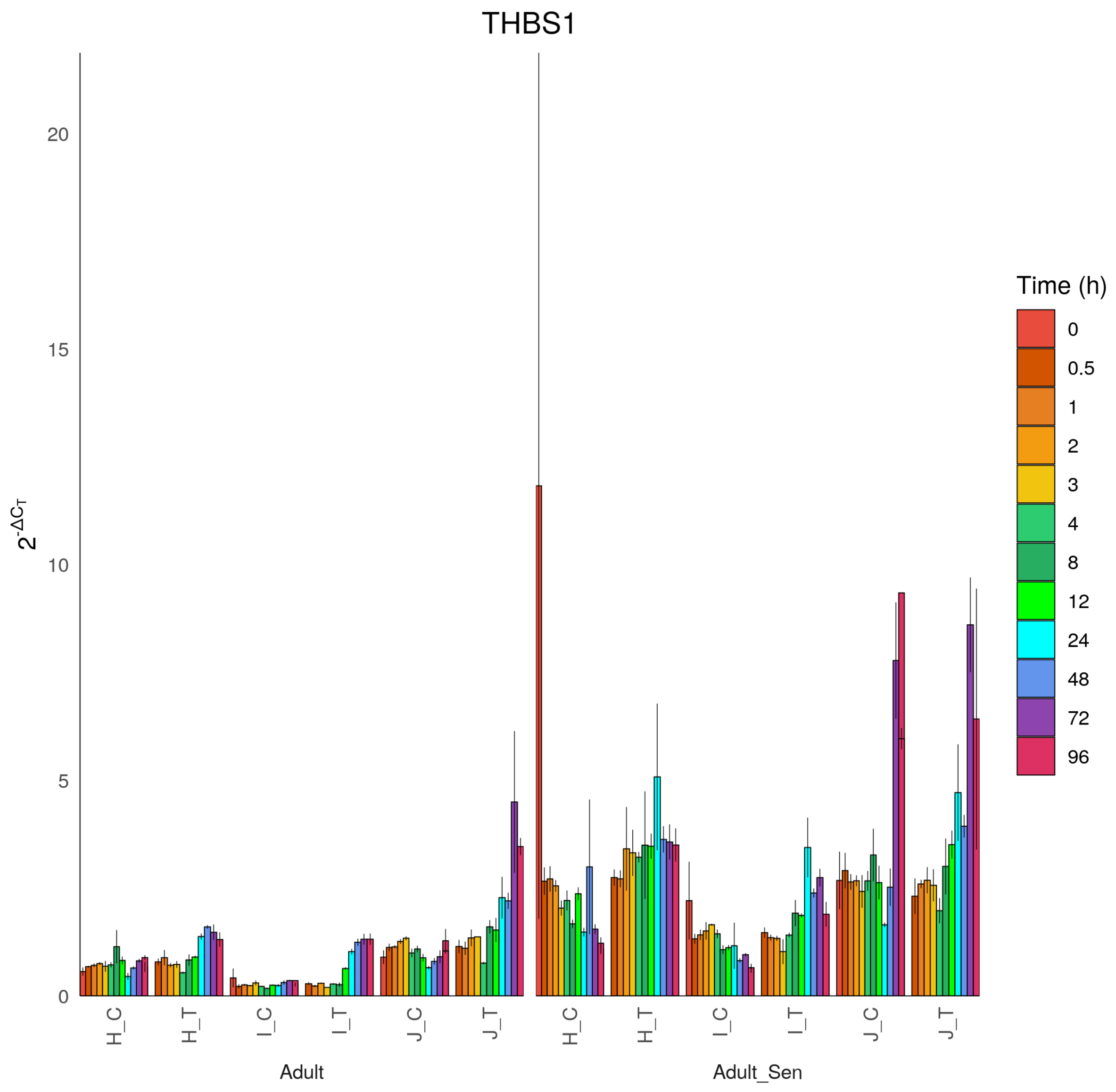

THBS1
$2^{-\Delta C_T}$
20
15
10
5
0
H_C
H_T
I_C
I_T
J_C
J_T
Adult
Adult_Sen
Time (h)
0
0.5
1
2
3
4
8
12
24
48
72
96

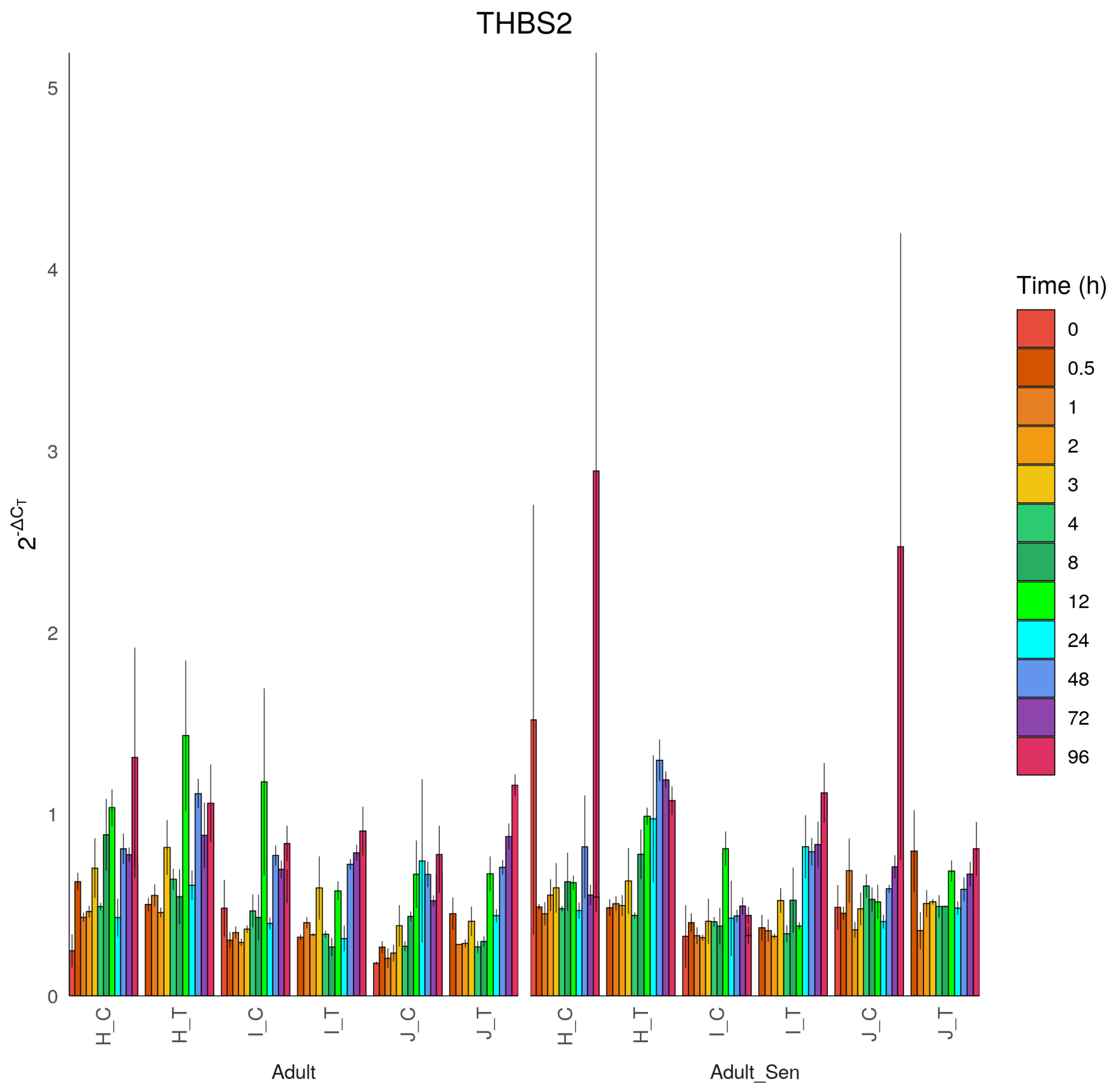
THBS2
$2^{-\Delta C_T}$
0
1
2
3
4
5
H_C
H_T
I_C
I_T
J_C
J_T
Adult
Adult_Sen
Time (h)
0
0.5
1
2
3
4
8
12
24
48
72
96

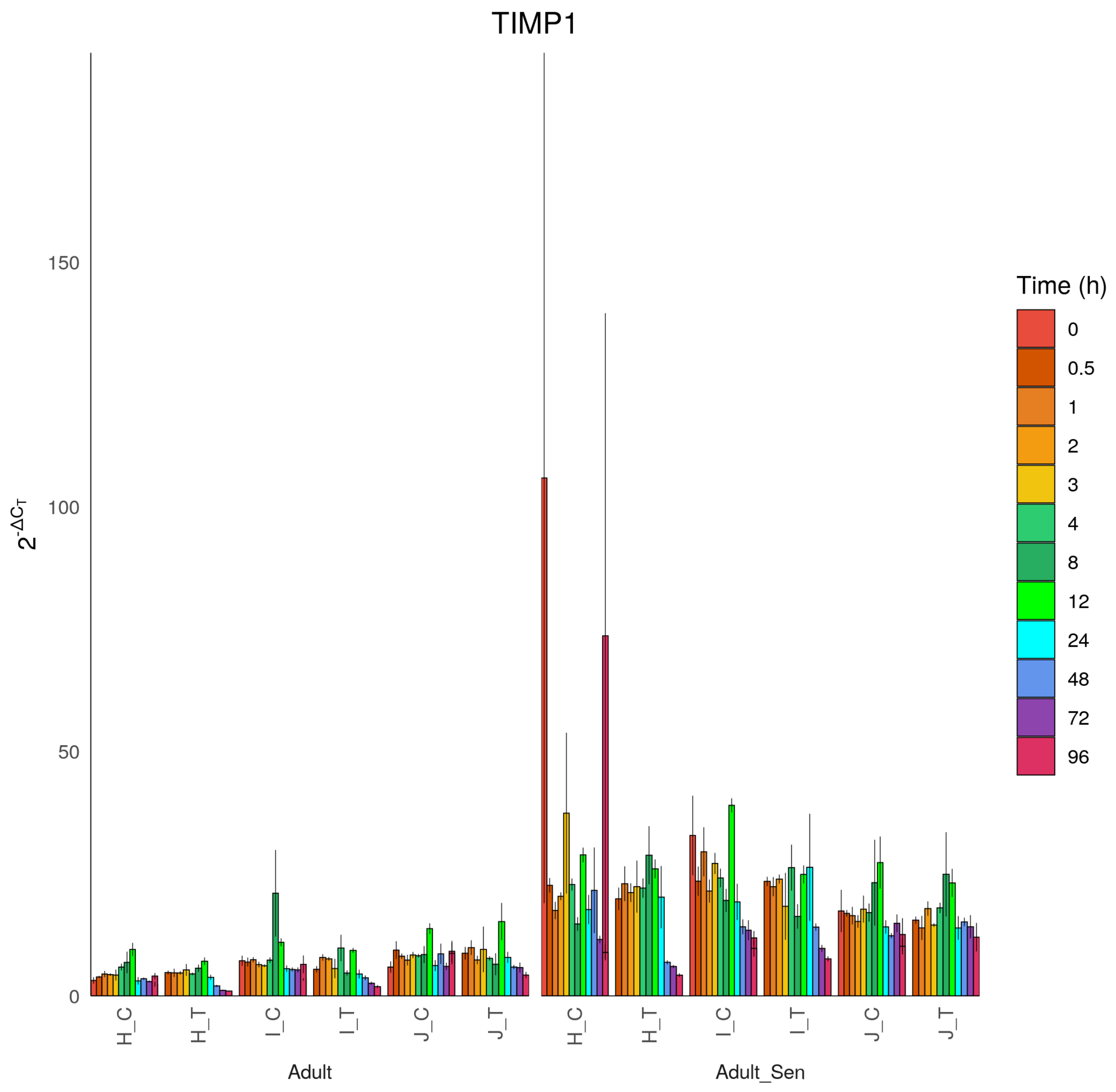
TIMP1
$2^{-\Delta C_T}$
0
50
100
150
H_C
H_T
I_C
I_T
J_C
J_T
Adult
Adult_Sen
Time (h)
0
0.5
1
2
3
4
8
12
24
48
72
96

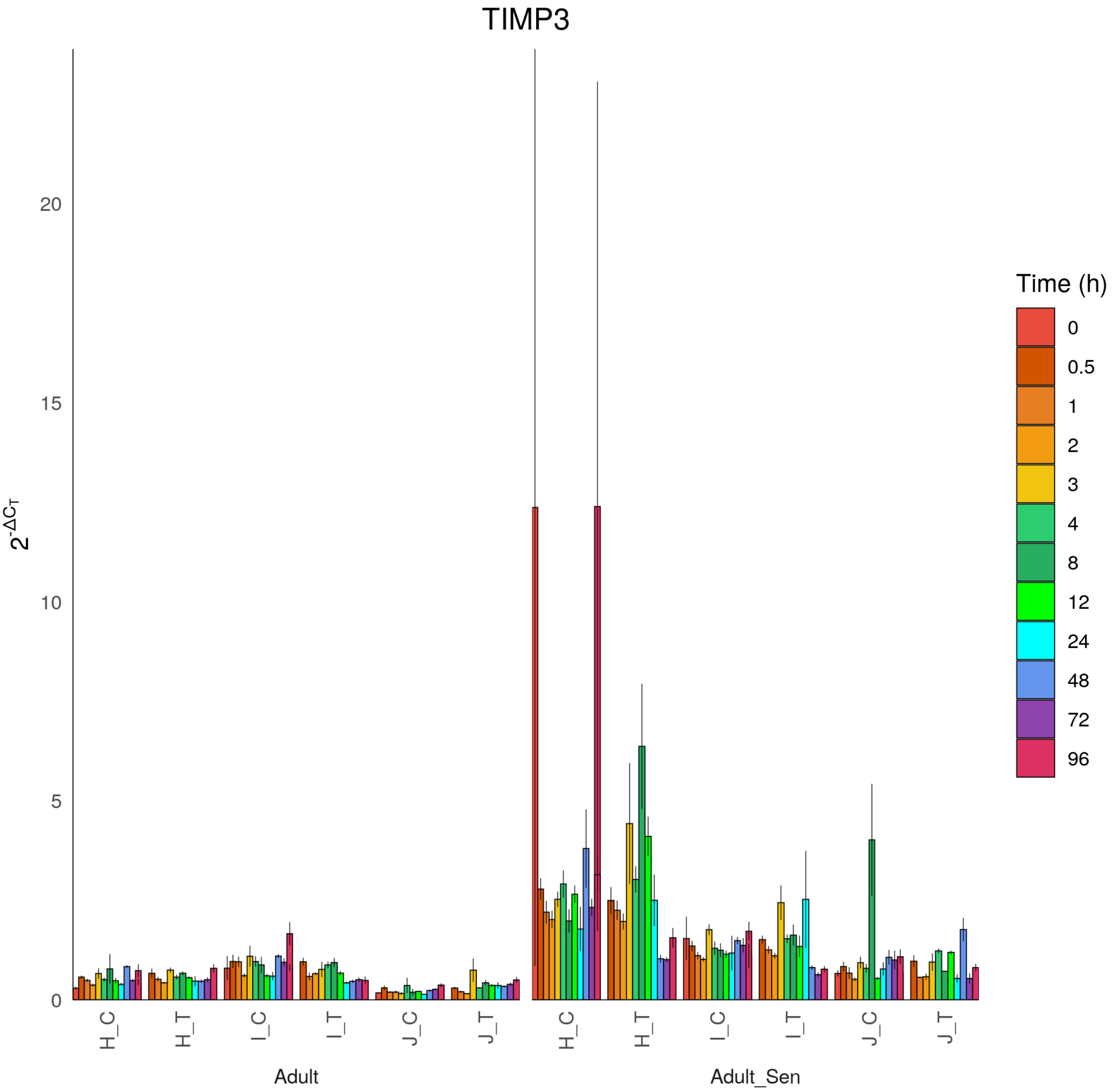

TIMP3
$2^{-\Delta C_T}$
0
5
10
15
20
H_C
H_T
I_C
I_T
J_C
J_T
Adult
Adult_Sen
Time (h)
0
0.5
1
2
3
4
8
12
24
48
72
96

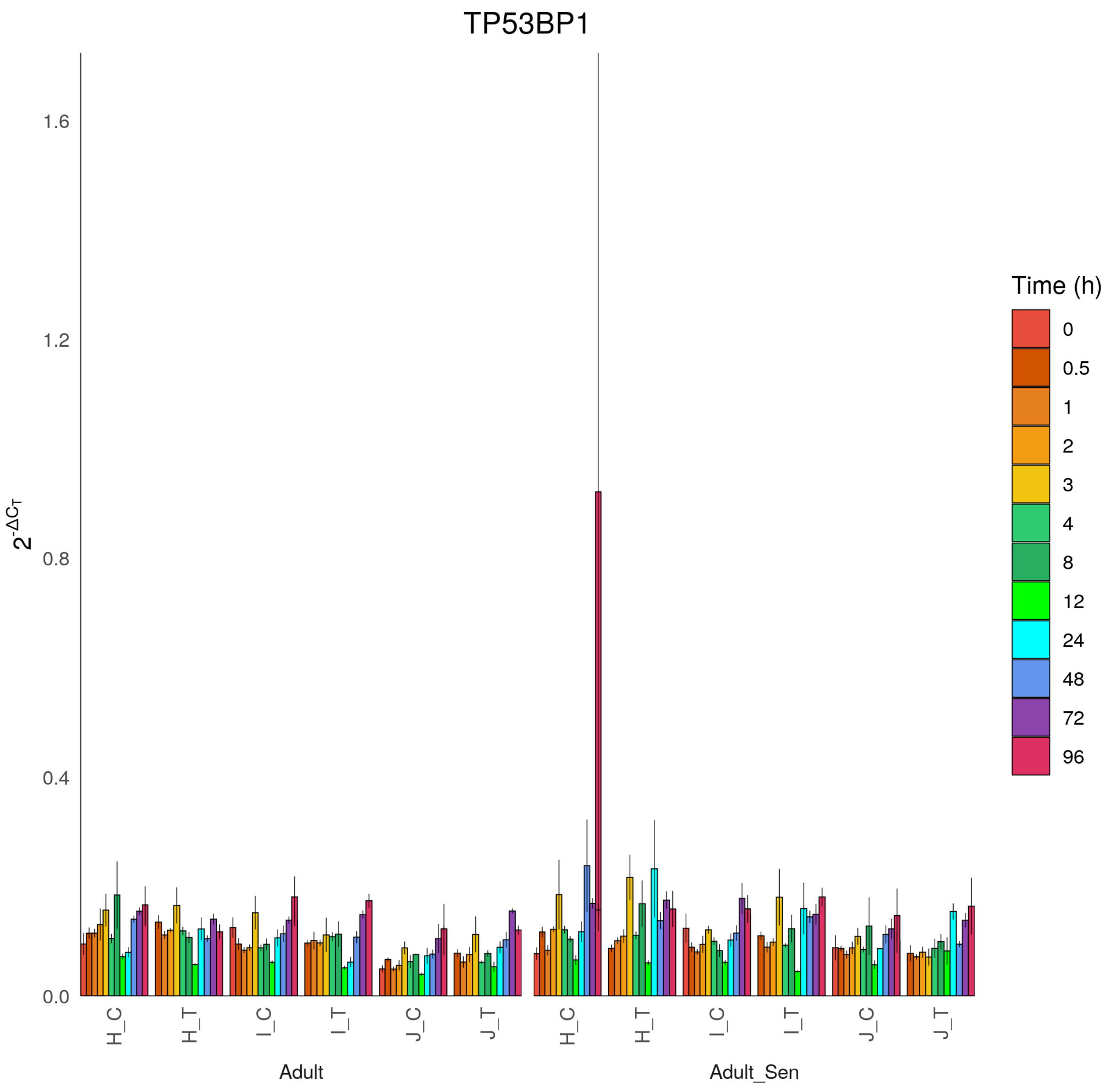

TP53BP1
$2^{-\Delta C_T}$
1.6
1.2
0.8
0.4
0.0
H_C
H_T
I_C
I_T
J_C
J_T
Adult
Adult_Sen
Time (h)
0
0.5
1
2
3
4
8
12
24
48
72
96

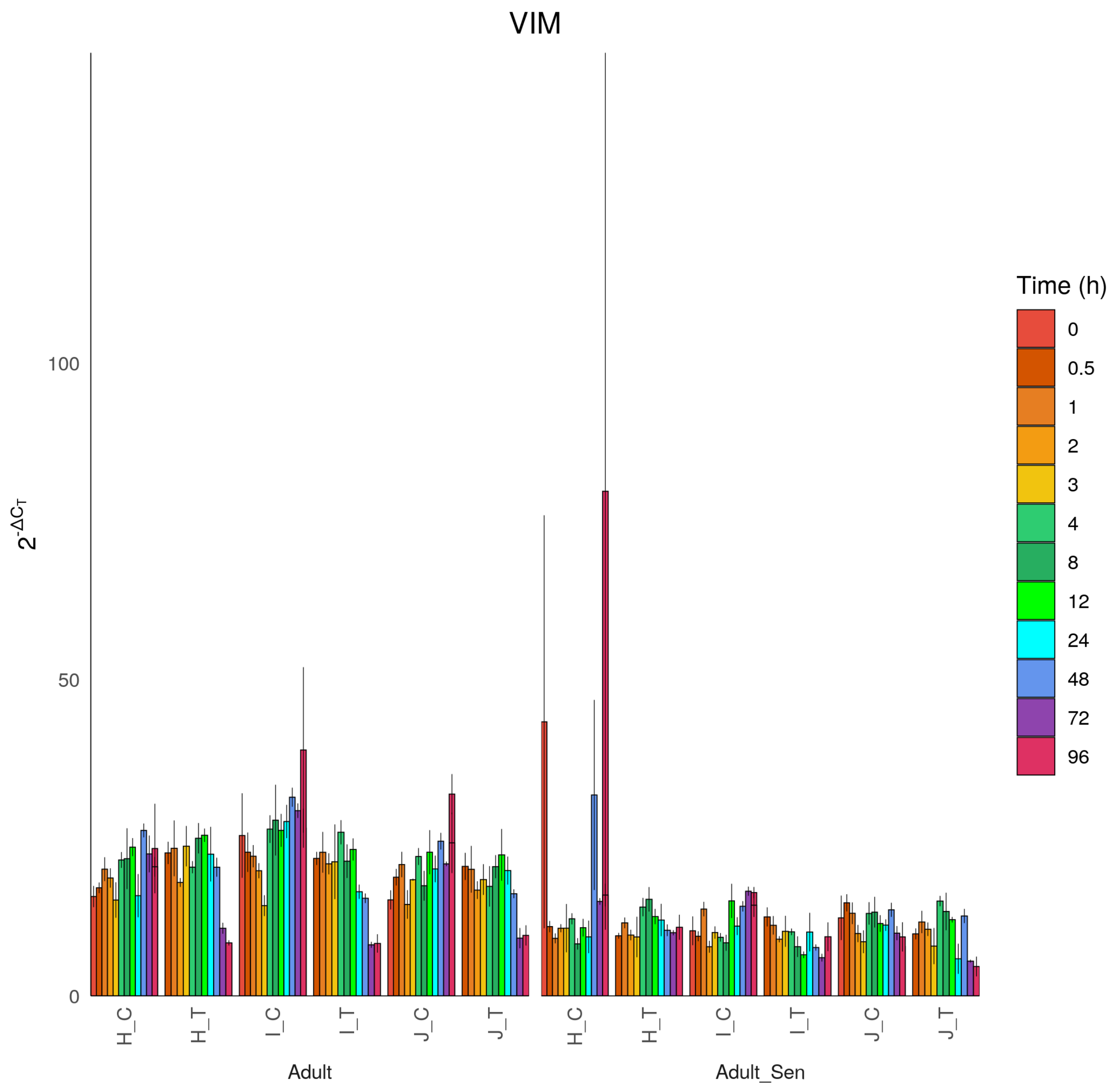

VIM
$2^{-\Delta C_T}$
100
50
0
H_C
H_T
I_C
I_T
J_C
J_T
H_C
H_T
I_C
I_T
J_C
J_T
Adult
Adult_Sen
Time (h)
0
0.5
1
2
3
4
8
12
24
48
72
96
