# Supplementary figures and images for: Cell Senescence-Independent Changes of Human Skin Fibroblasts with Age
Source: Cells. 2024 Apr 9;13(8):659. doi: 10.3390/cells13080659 (PMC11048776; doi:10.3390/cells13080659)

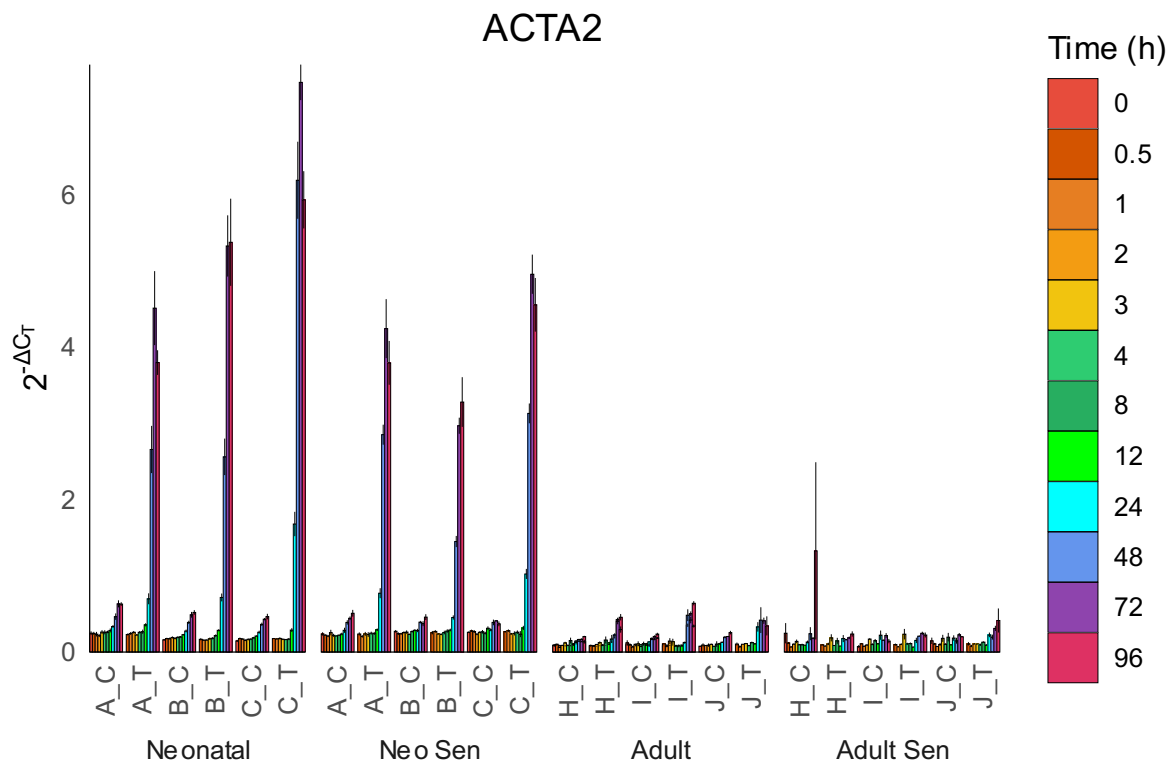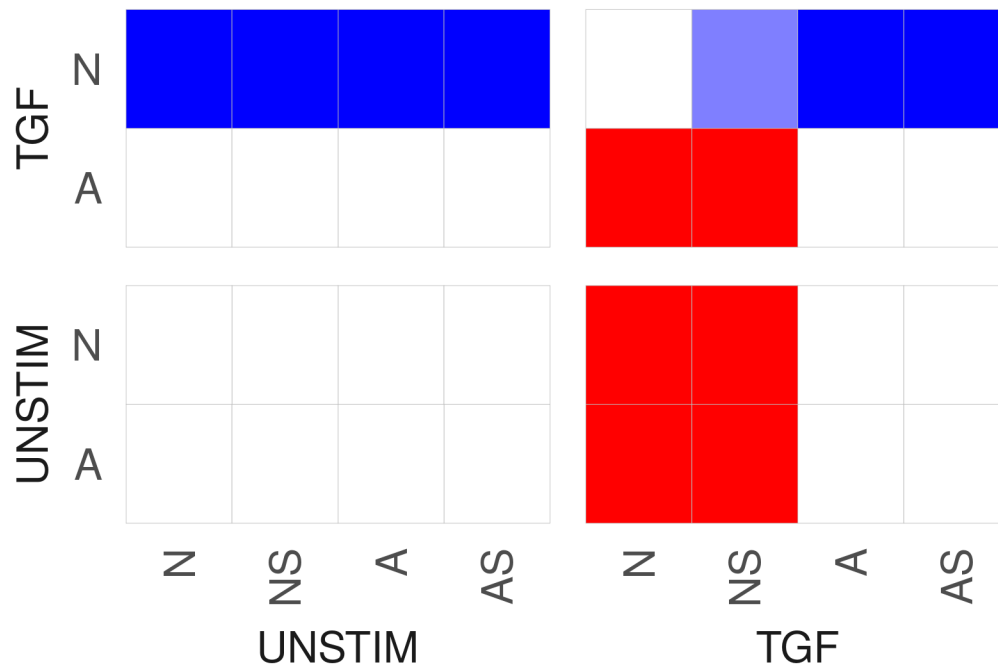

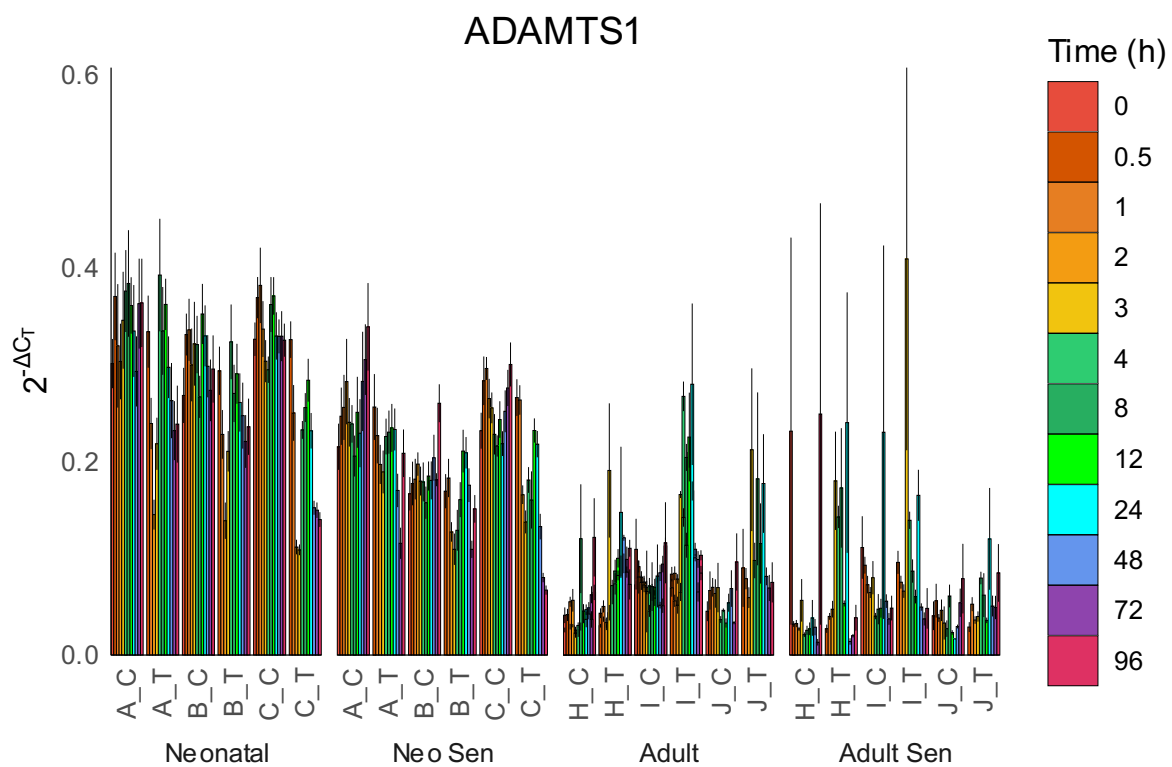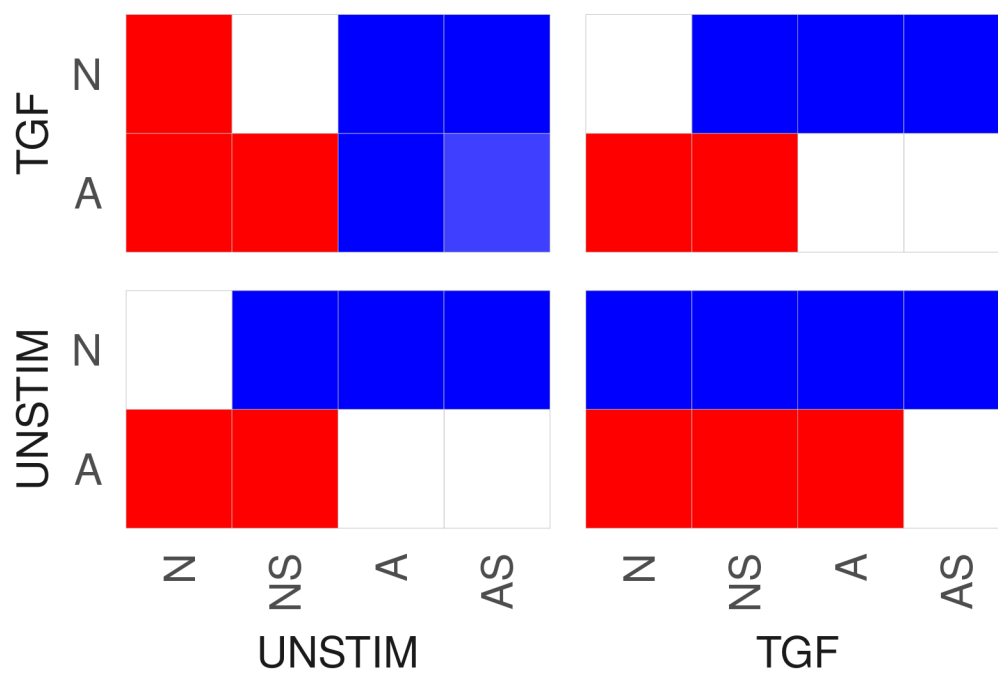

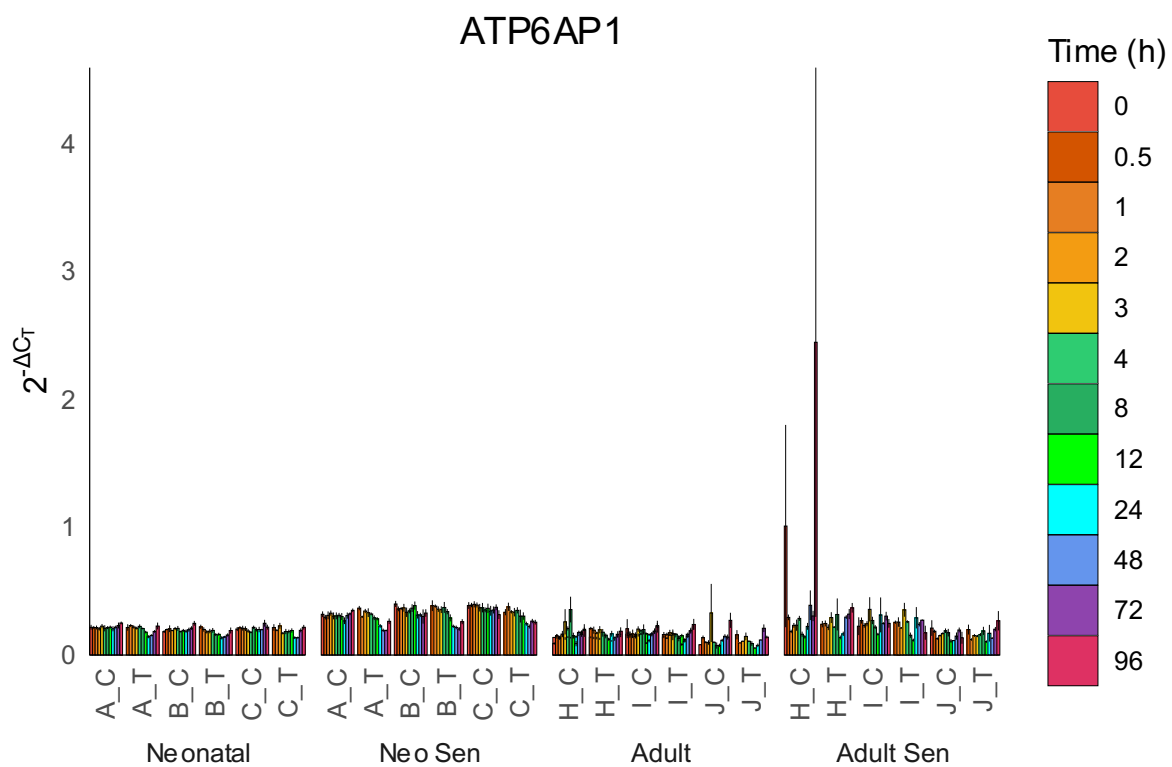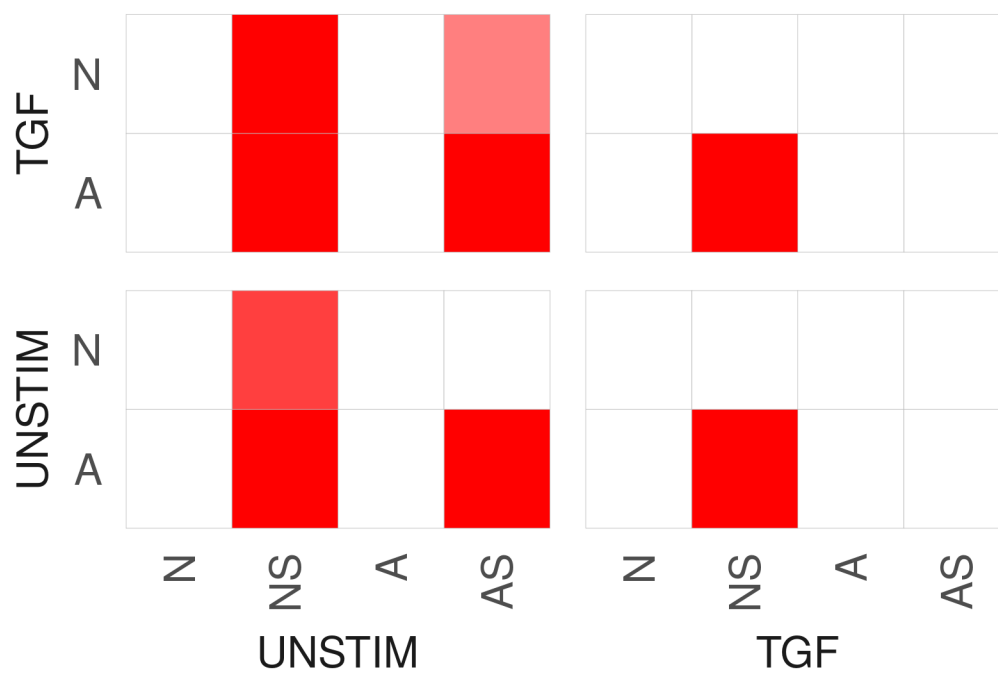



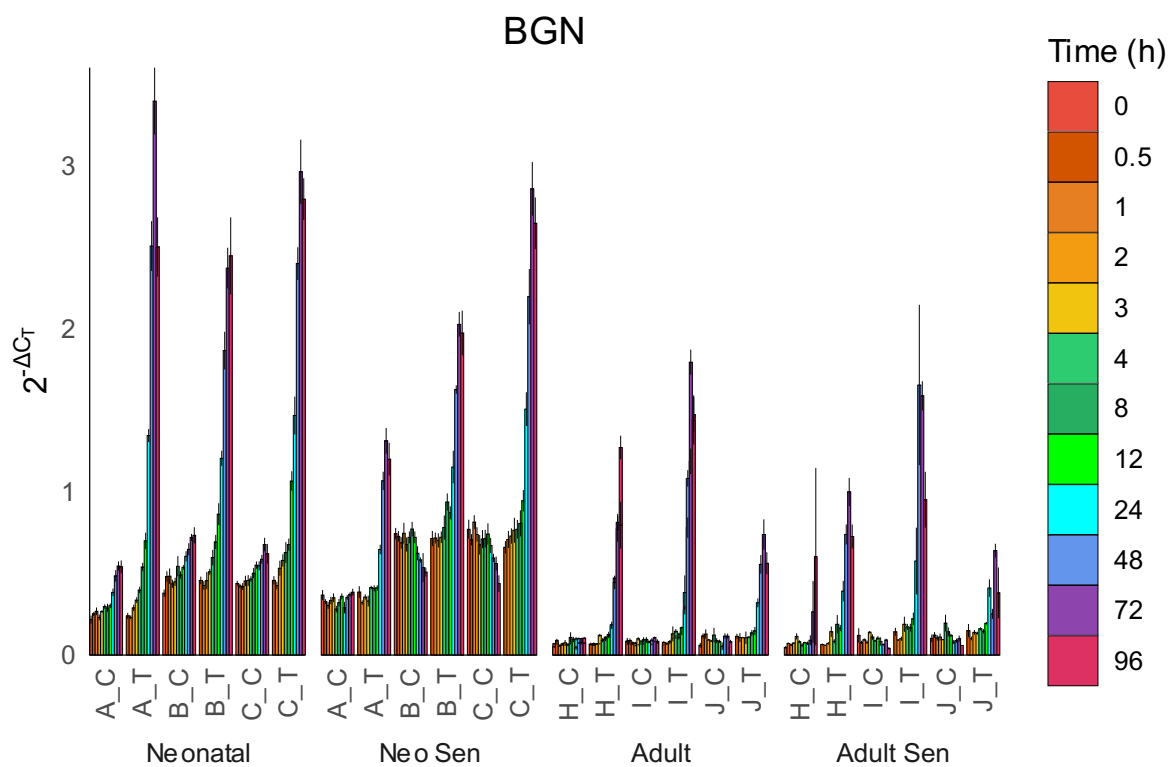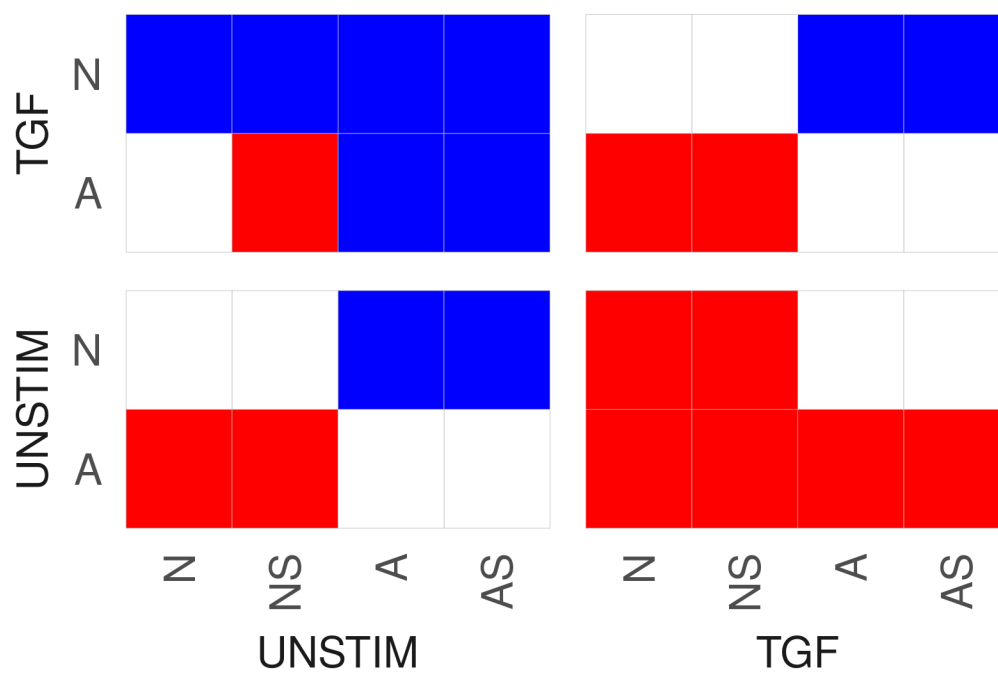

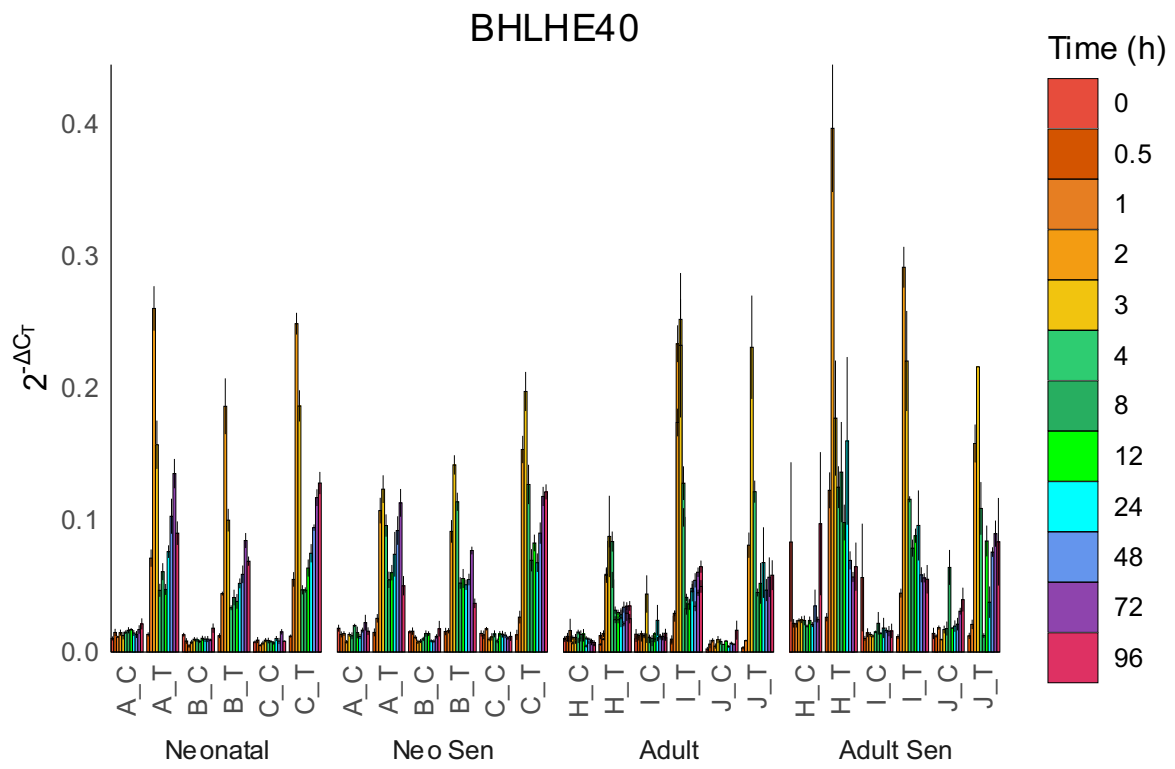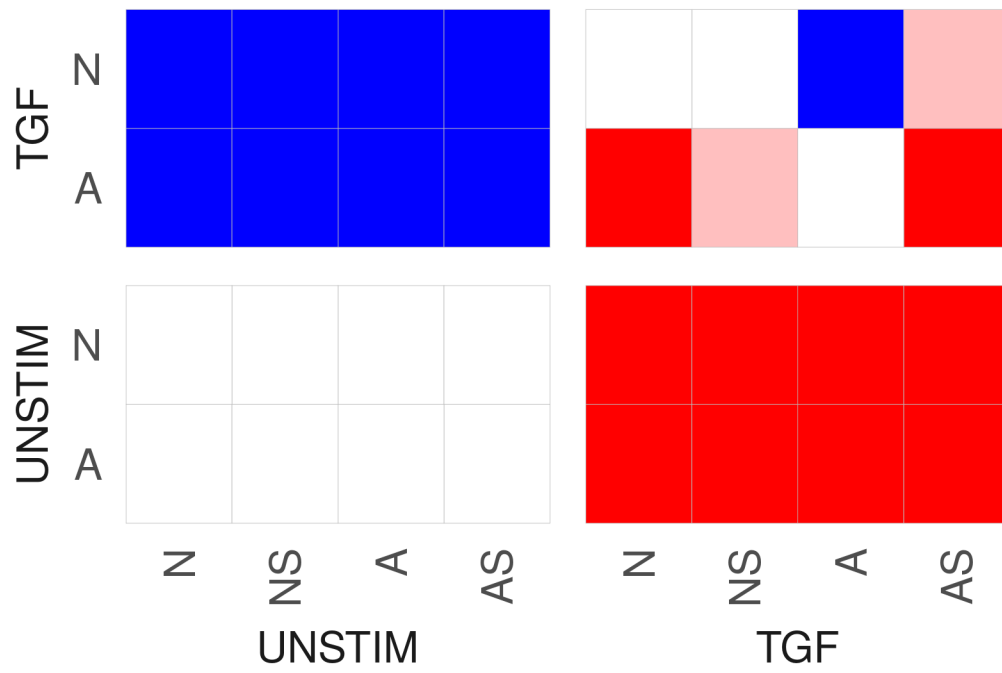

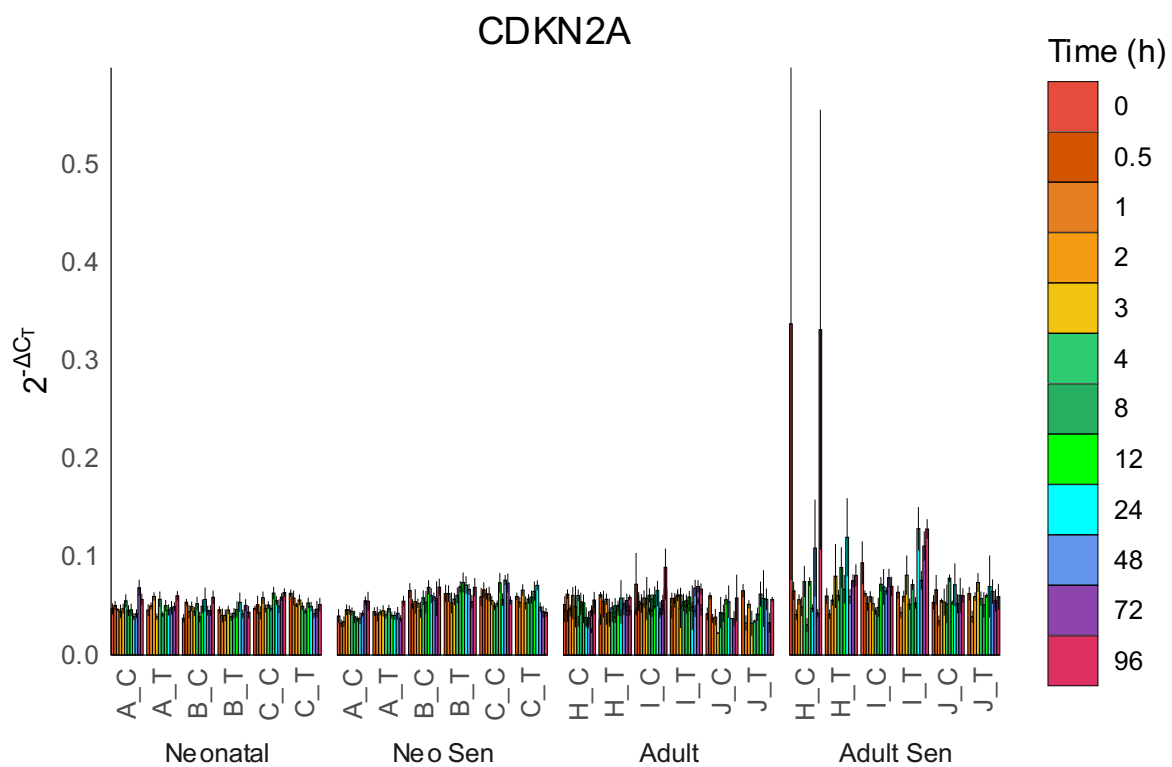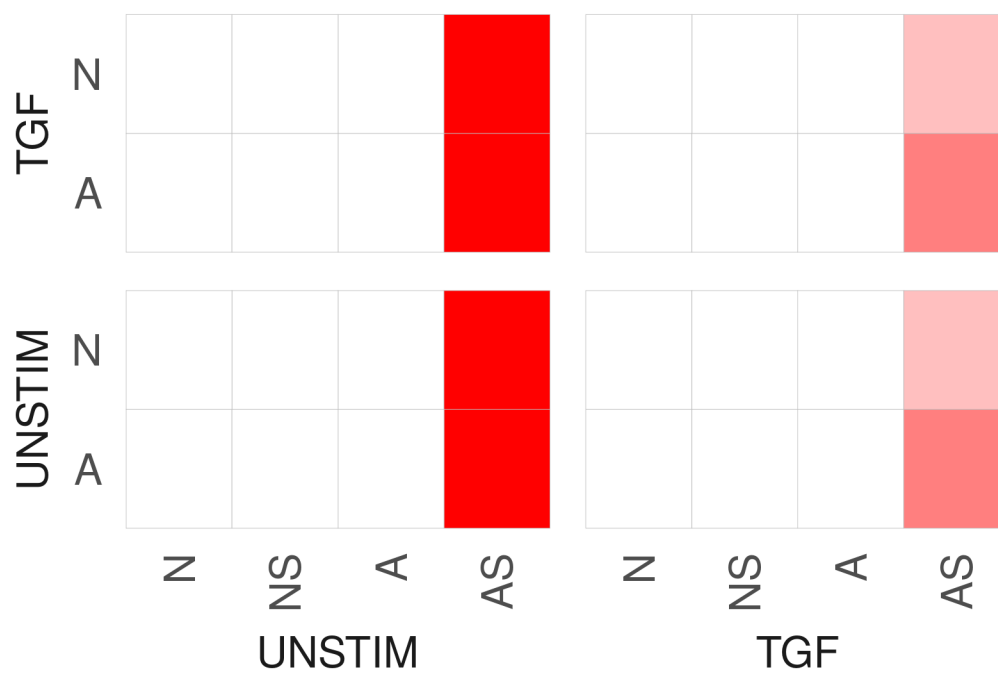

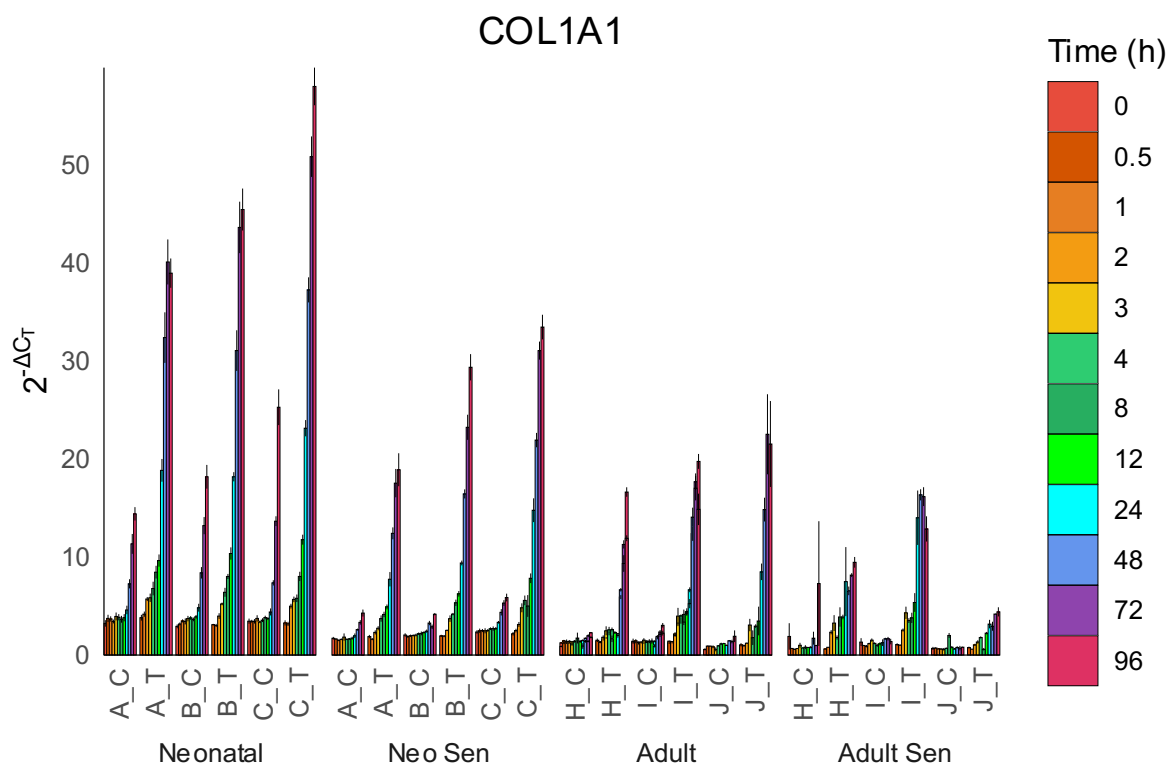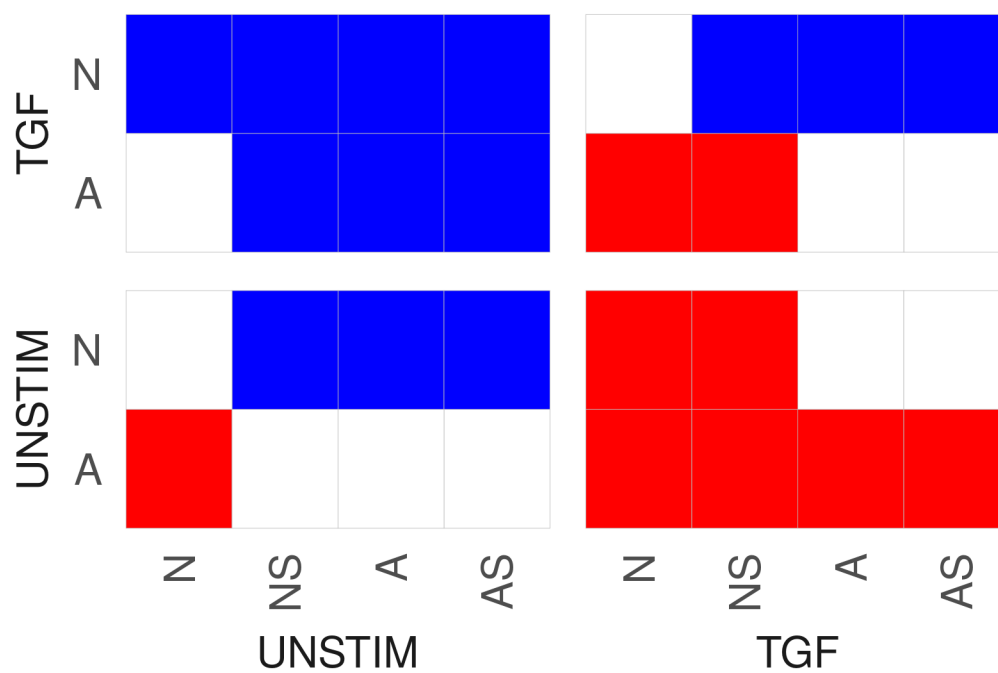

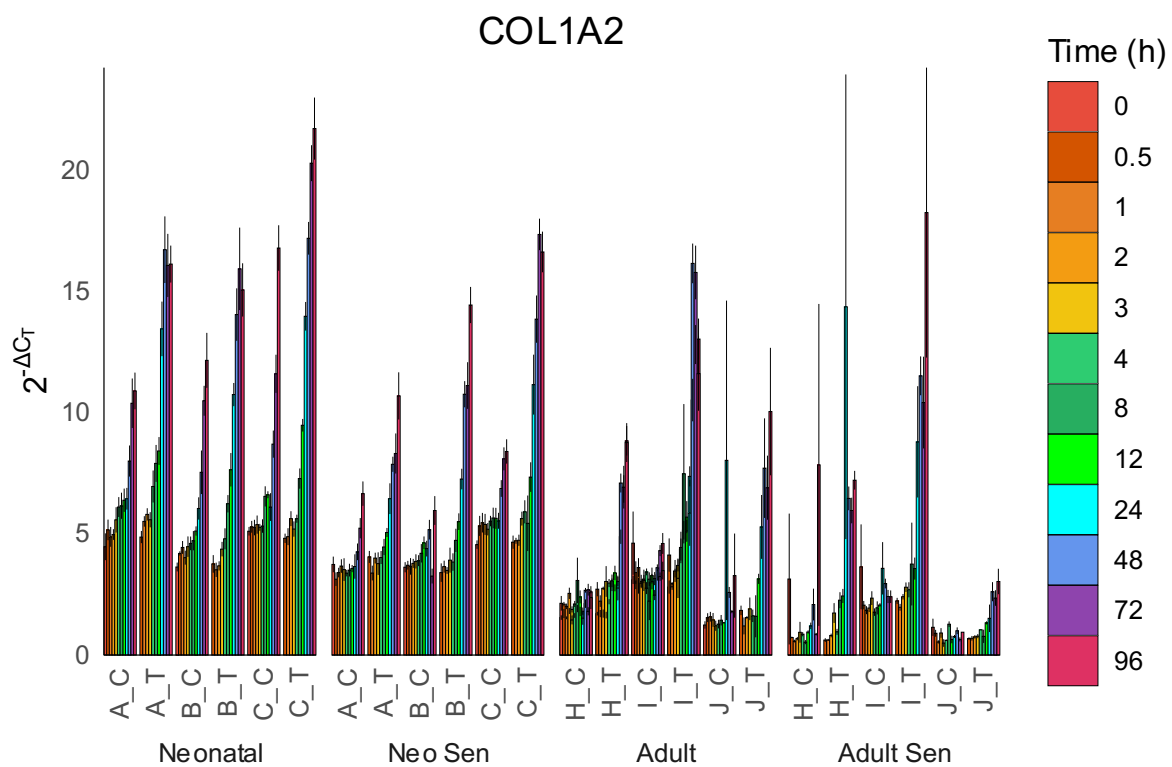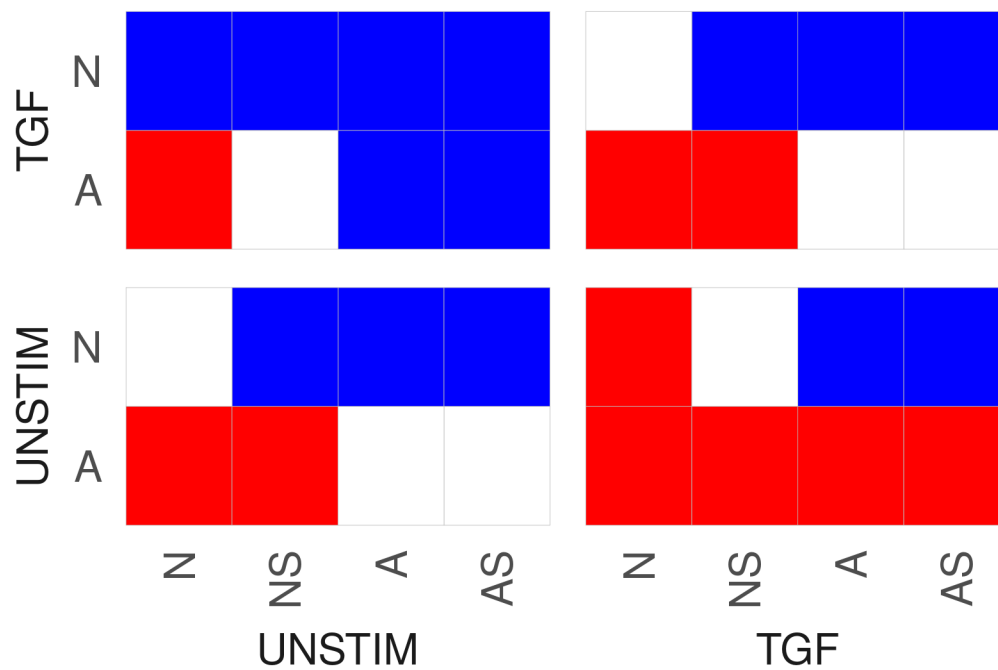

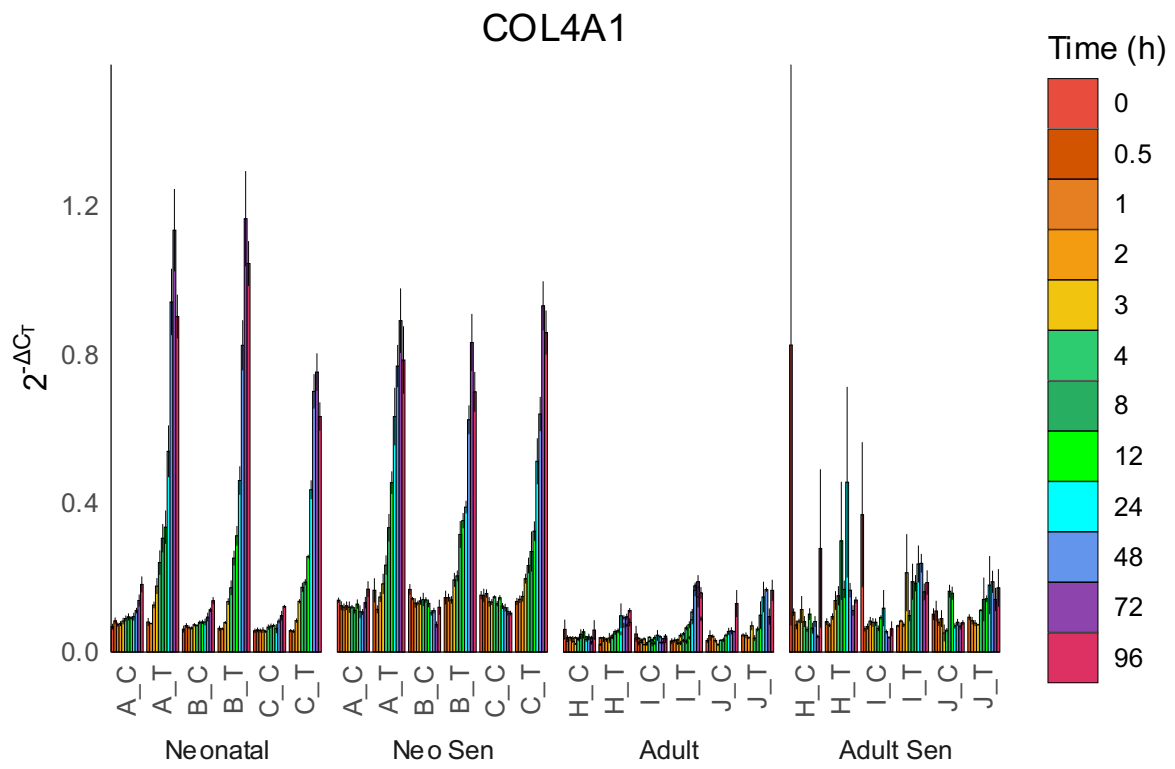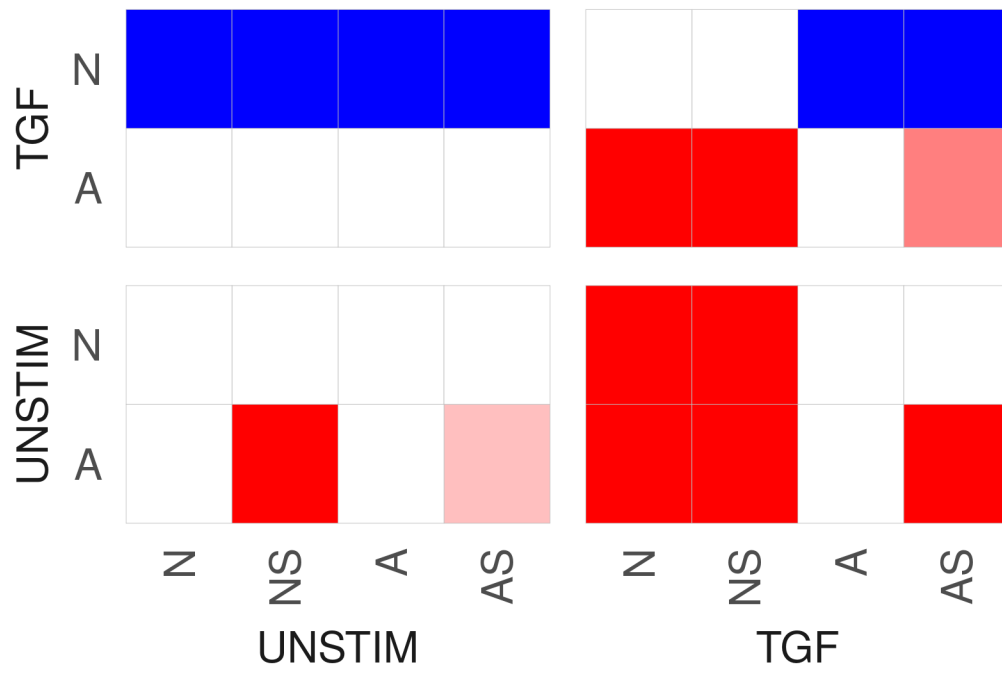

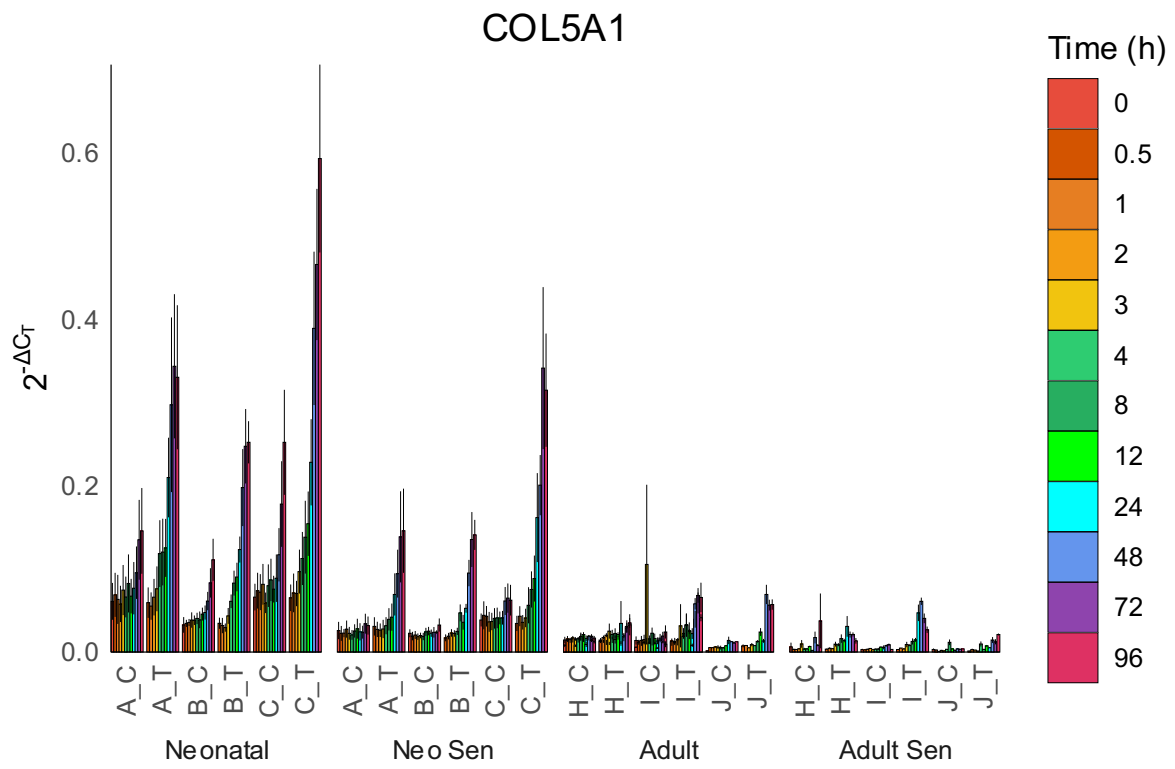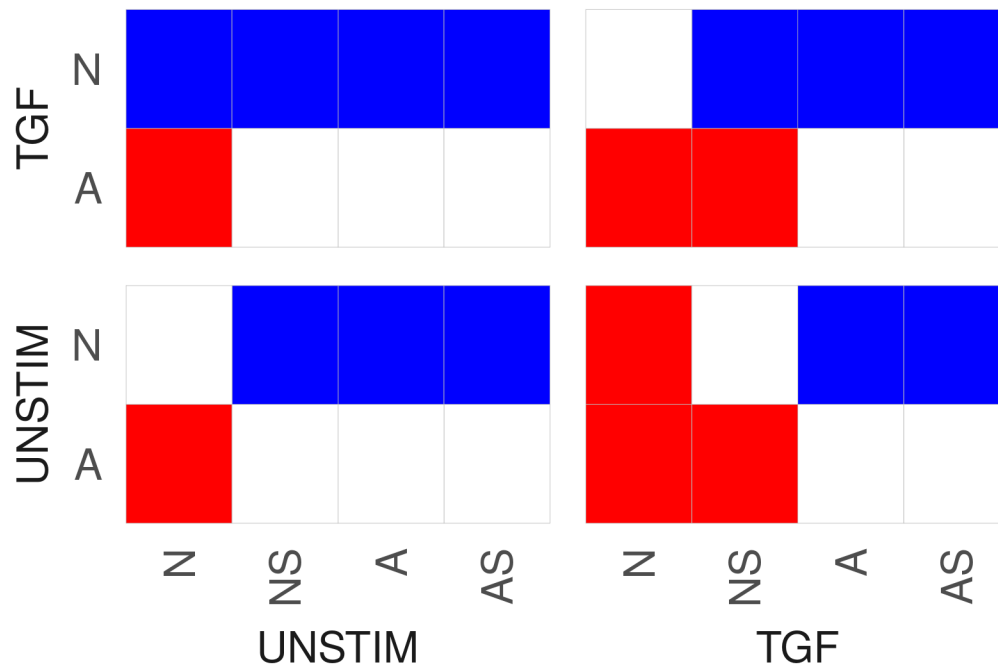

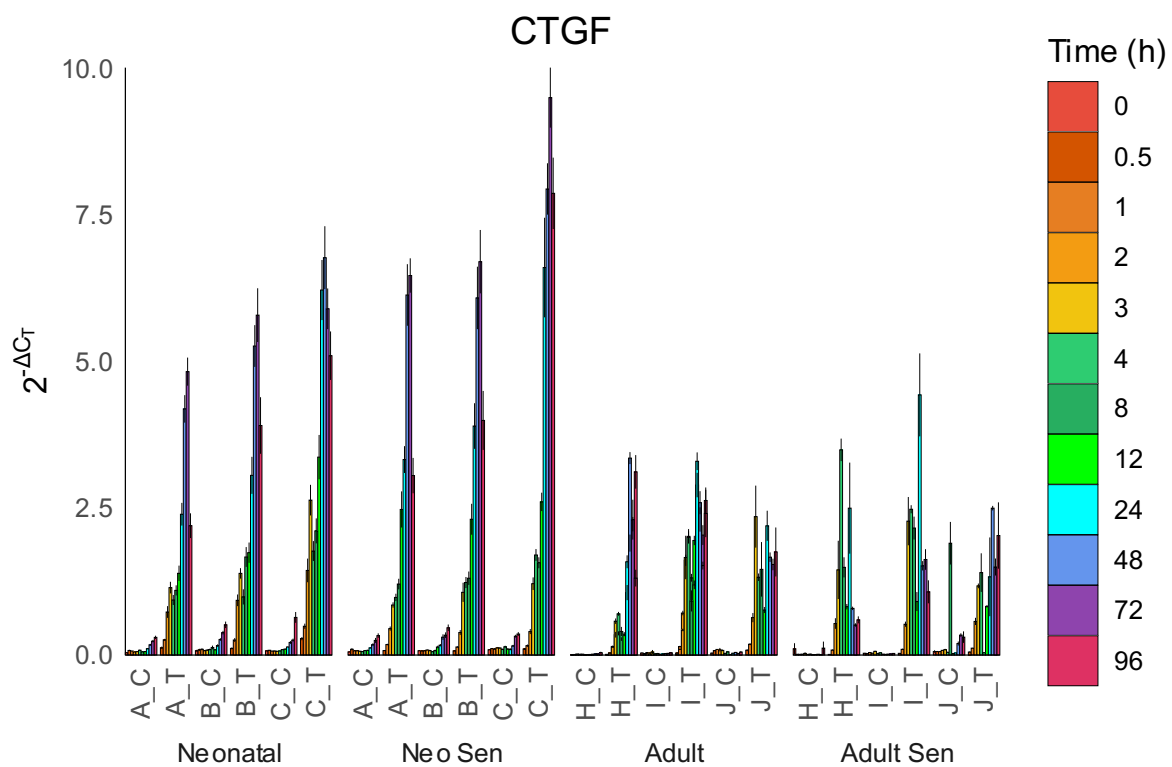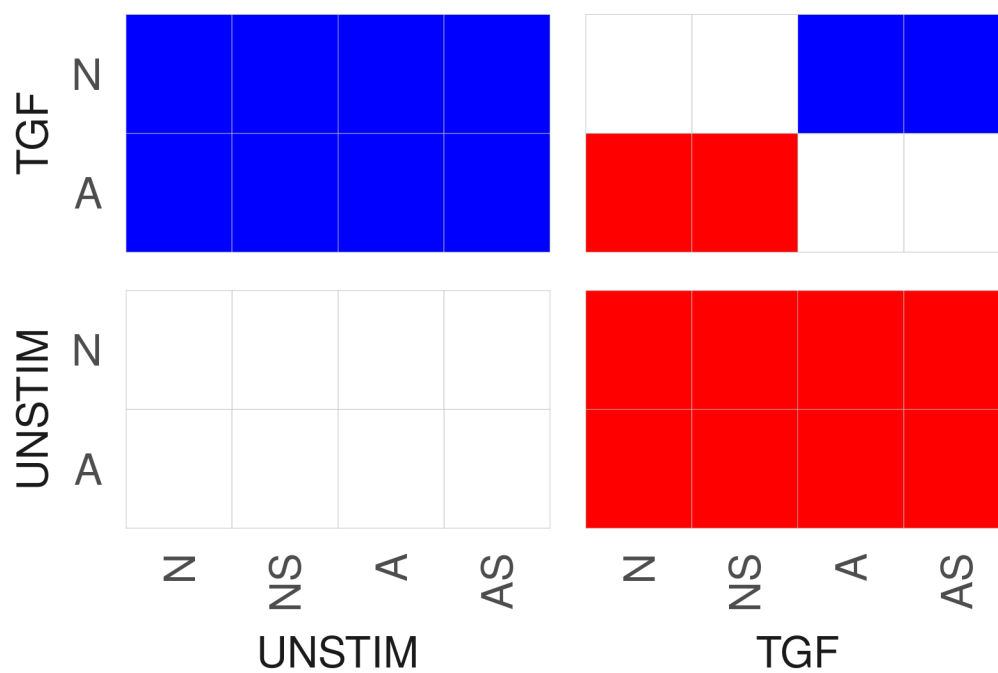

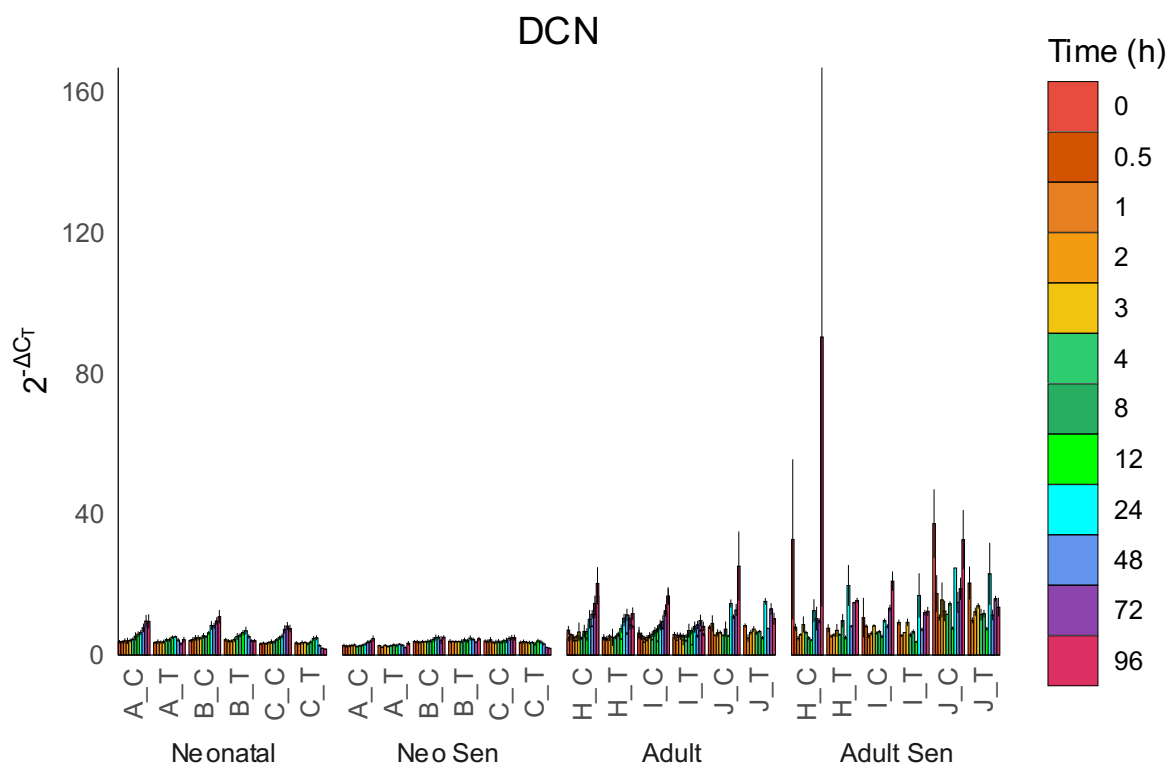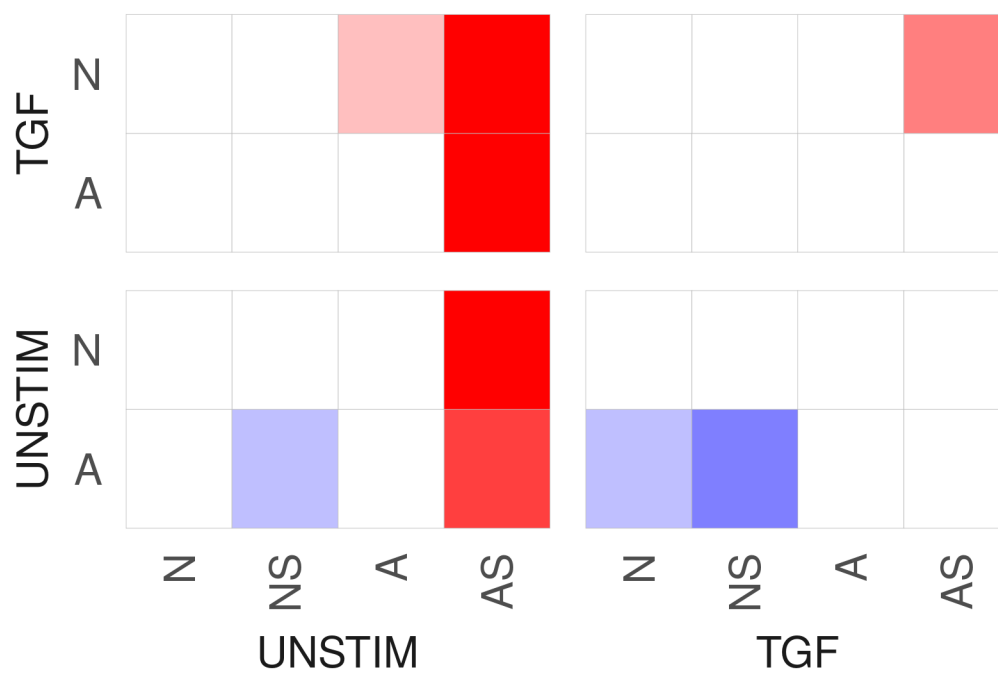

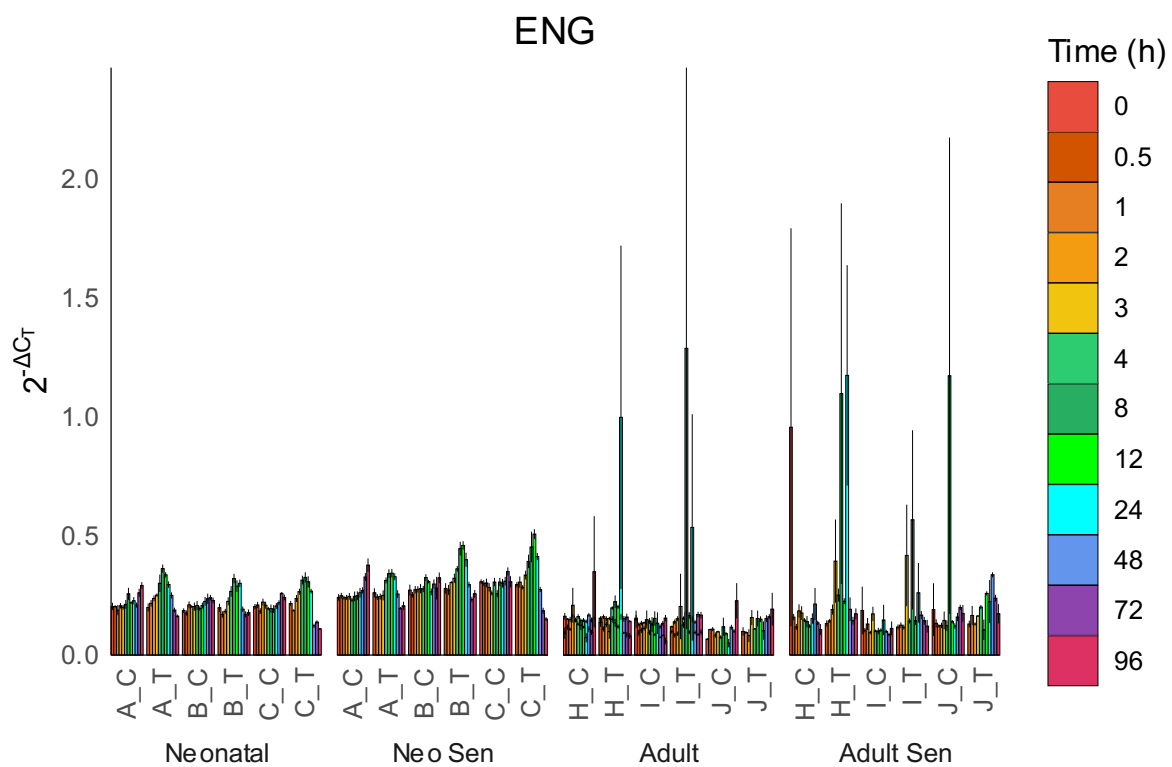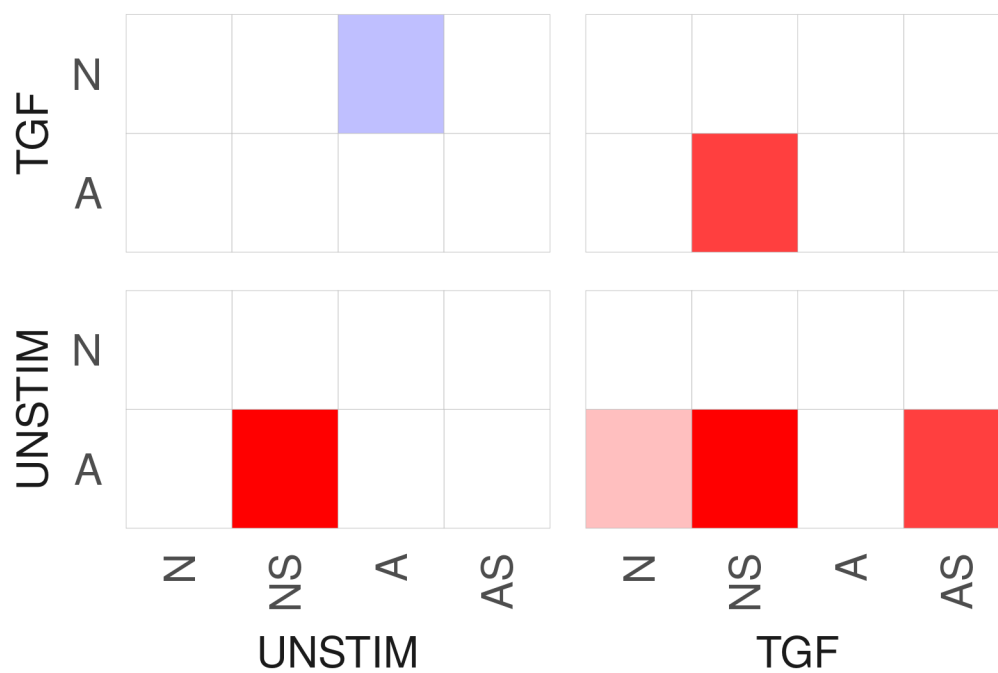

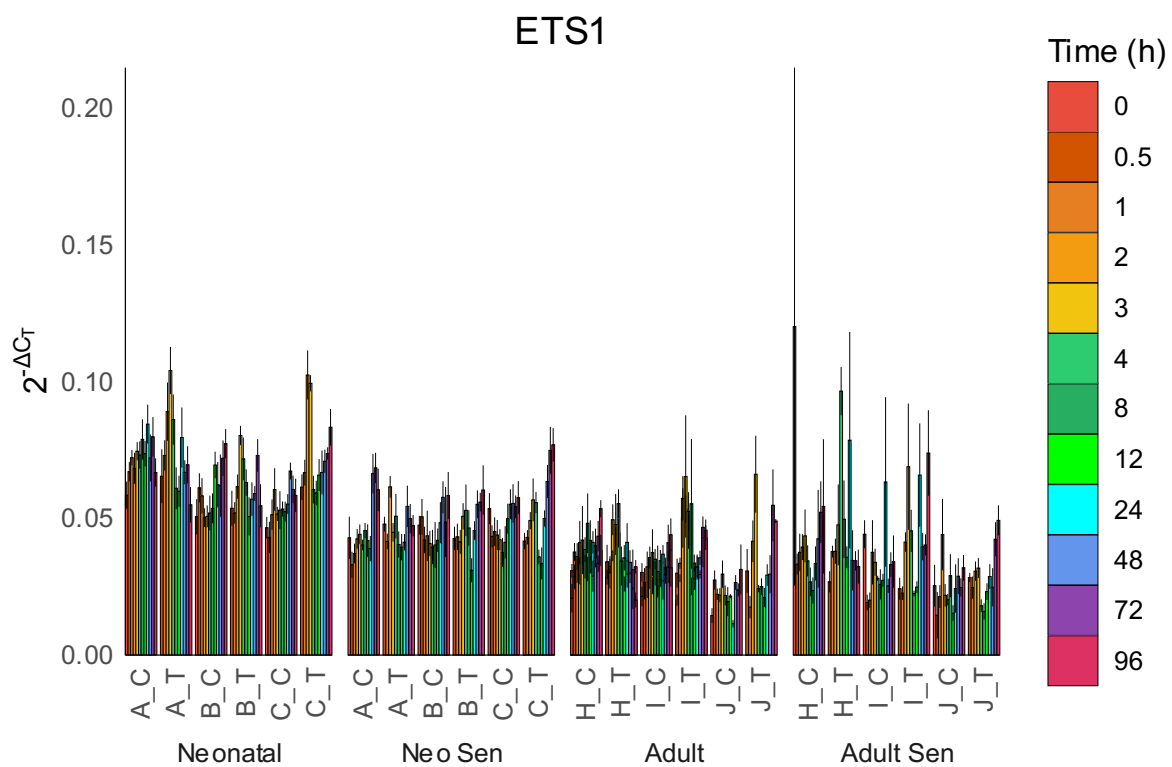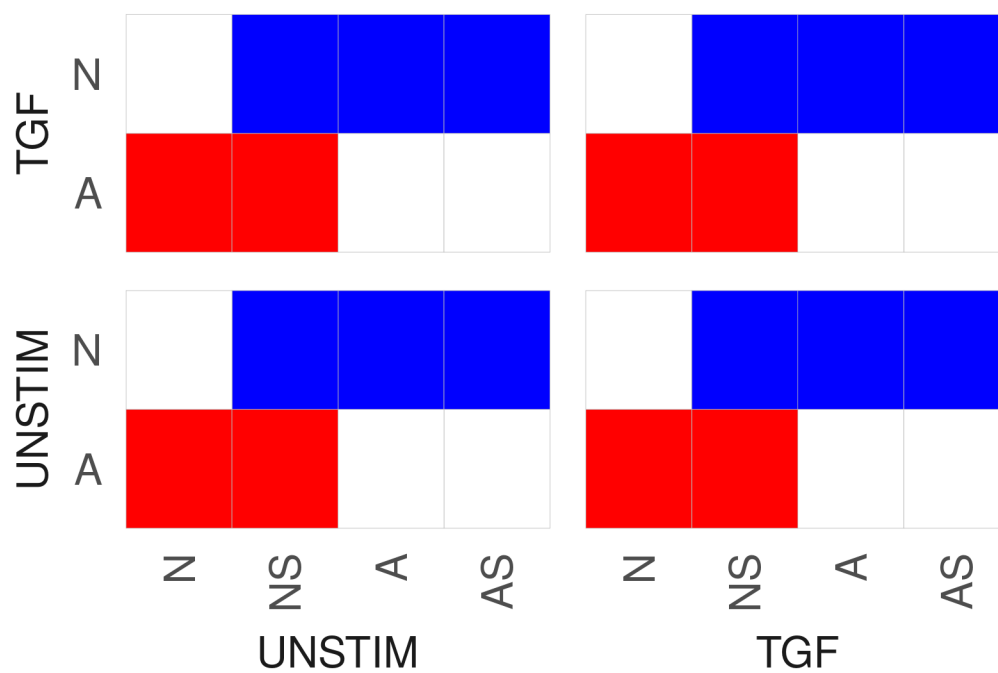

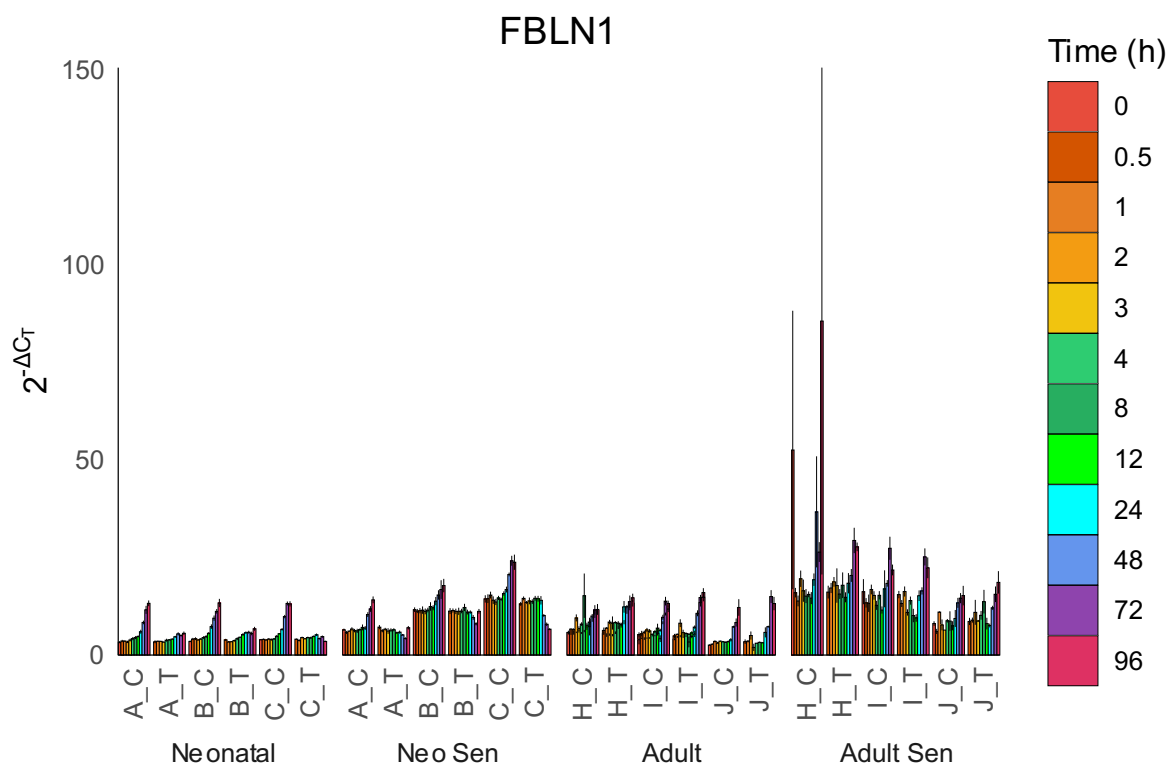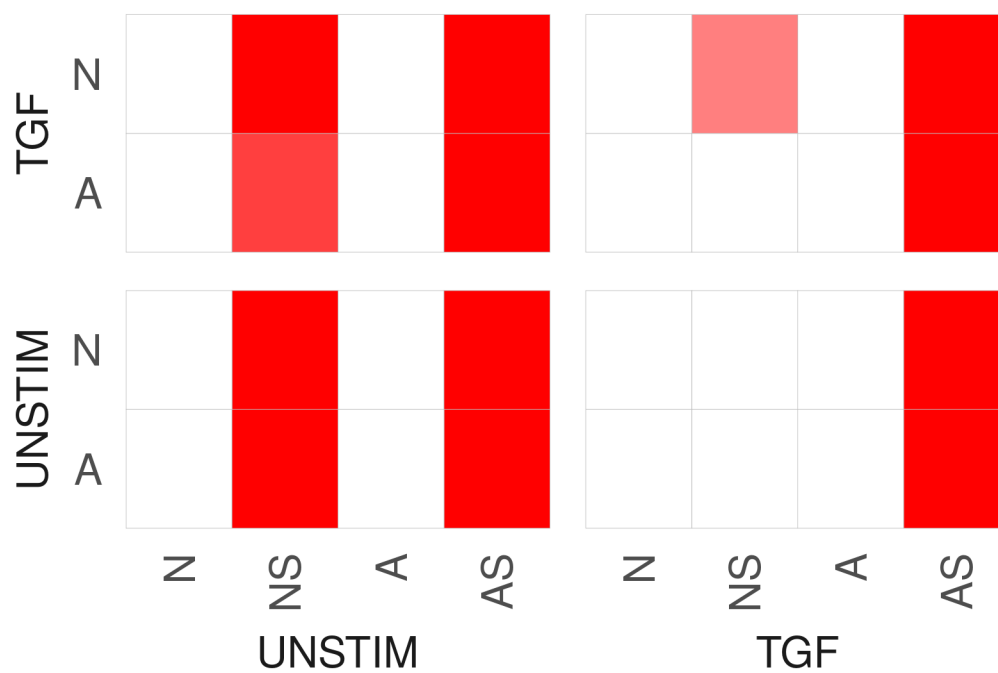

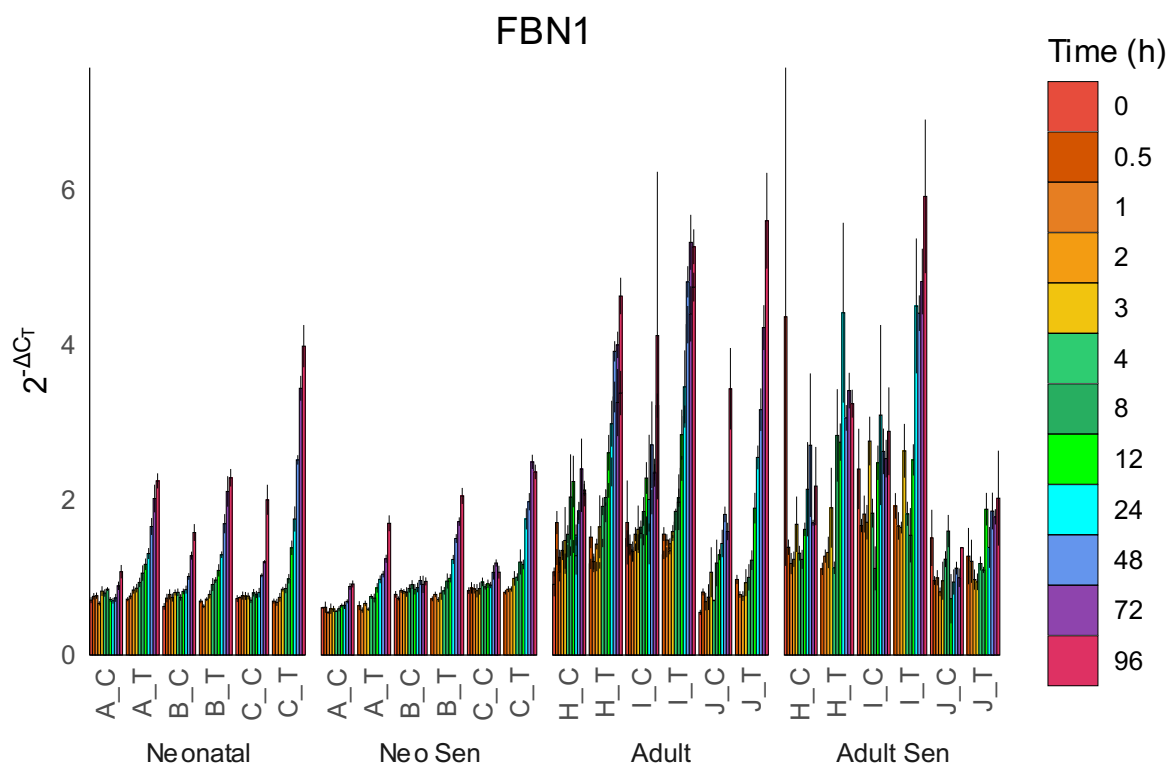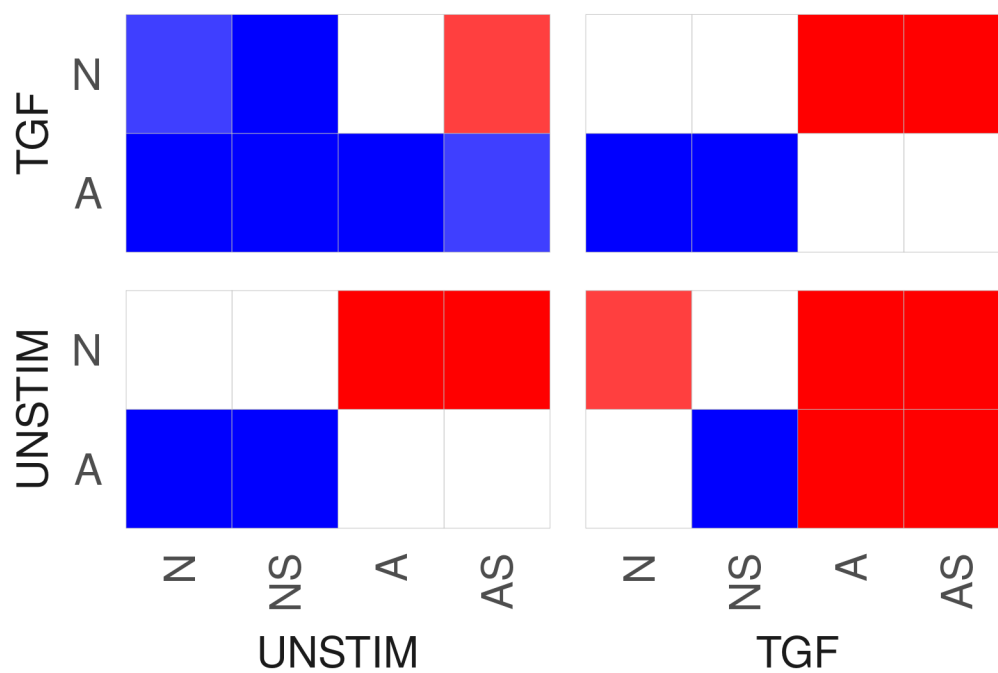

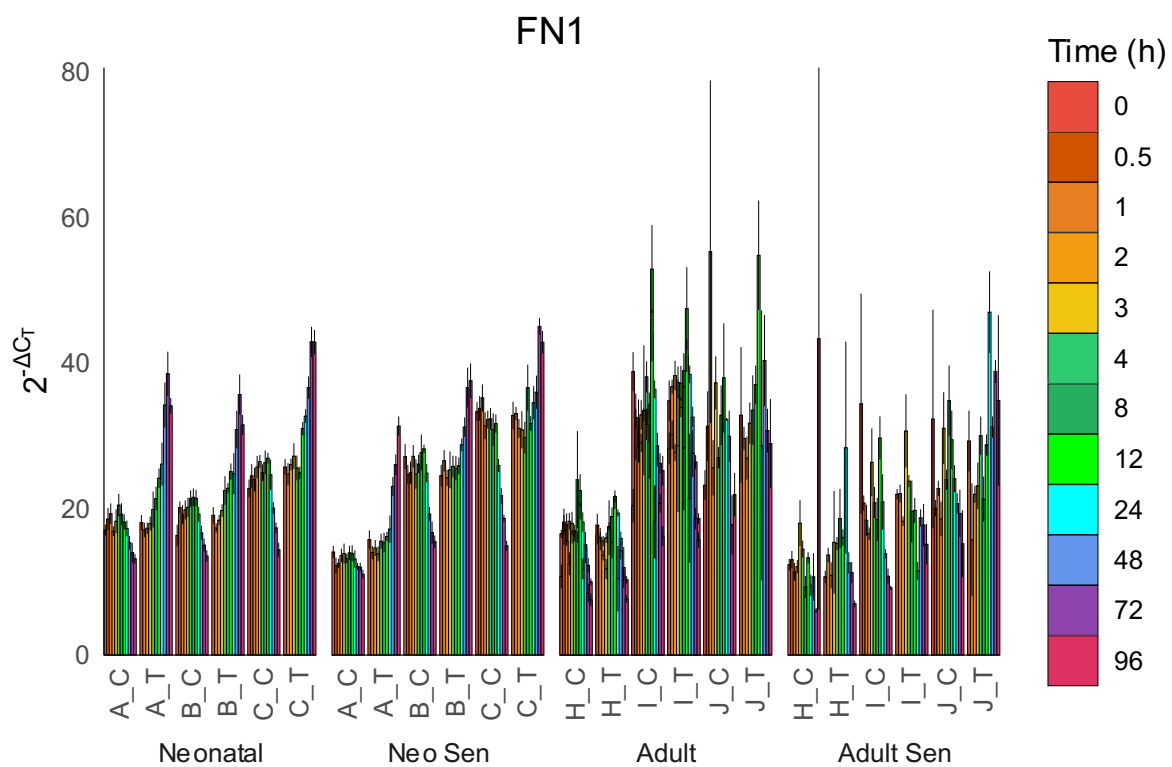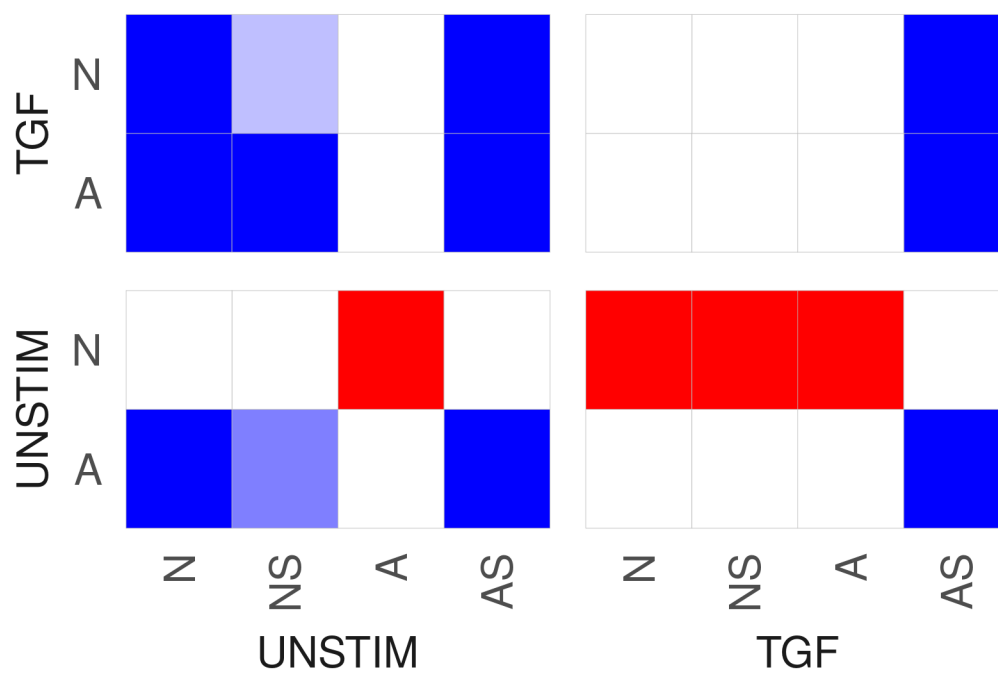

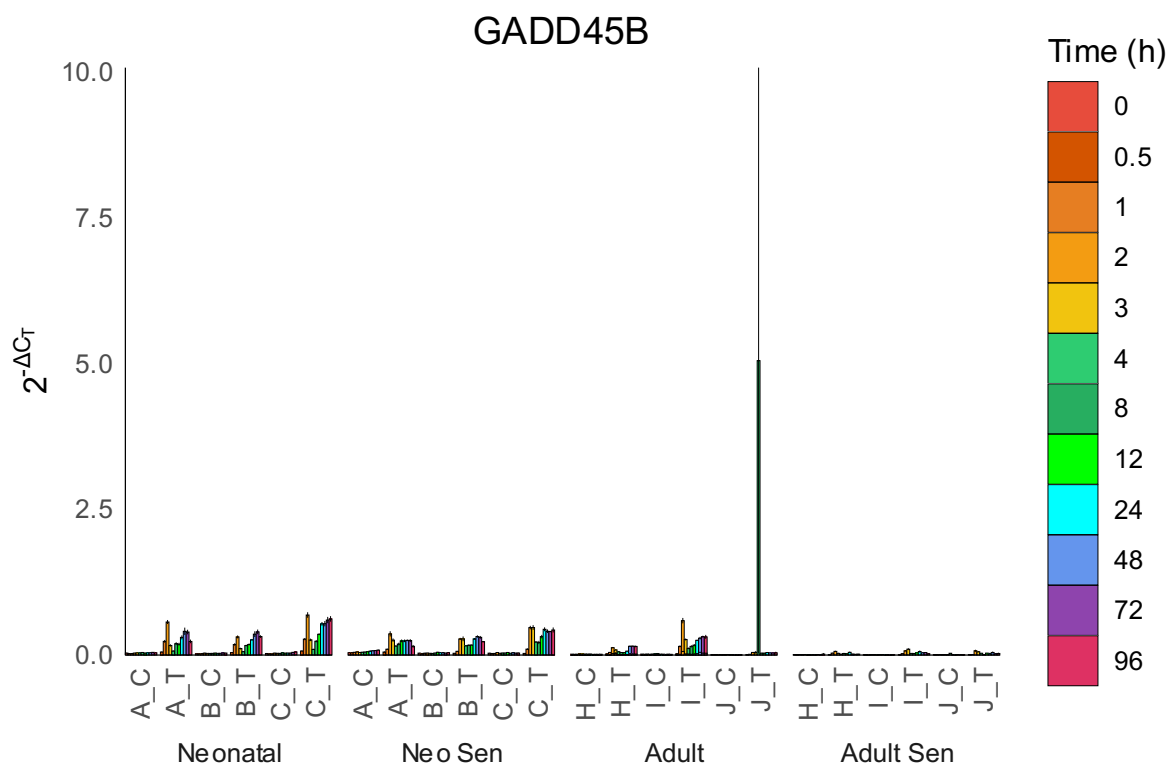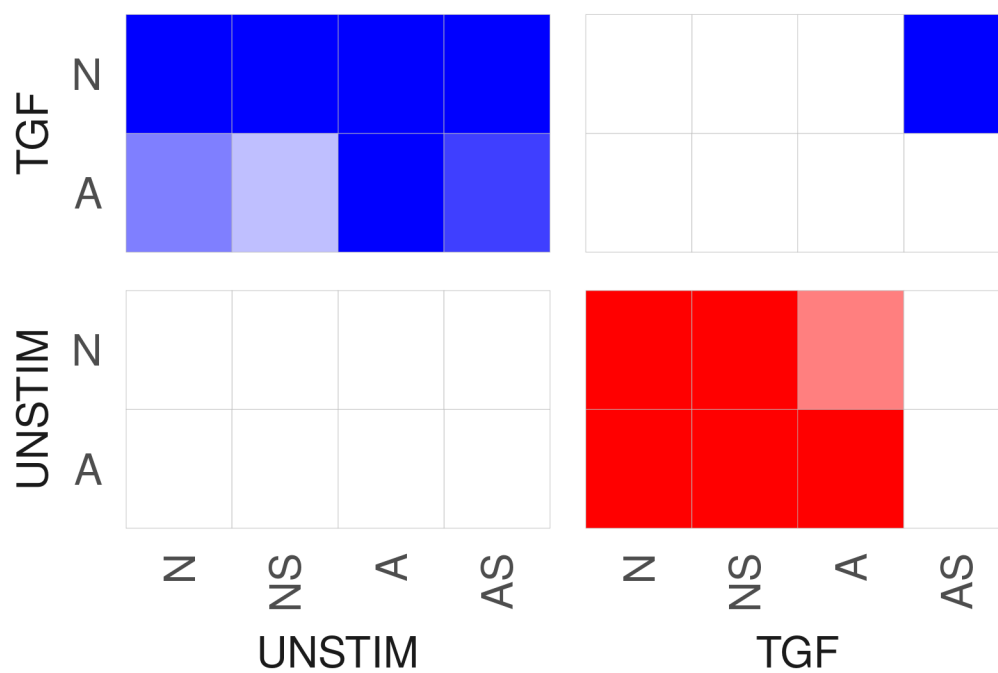

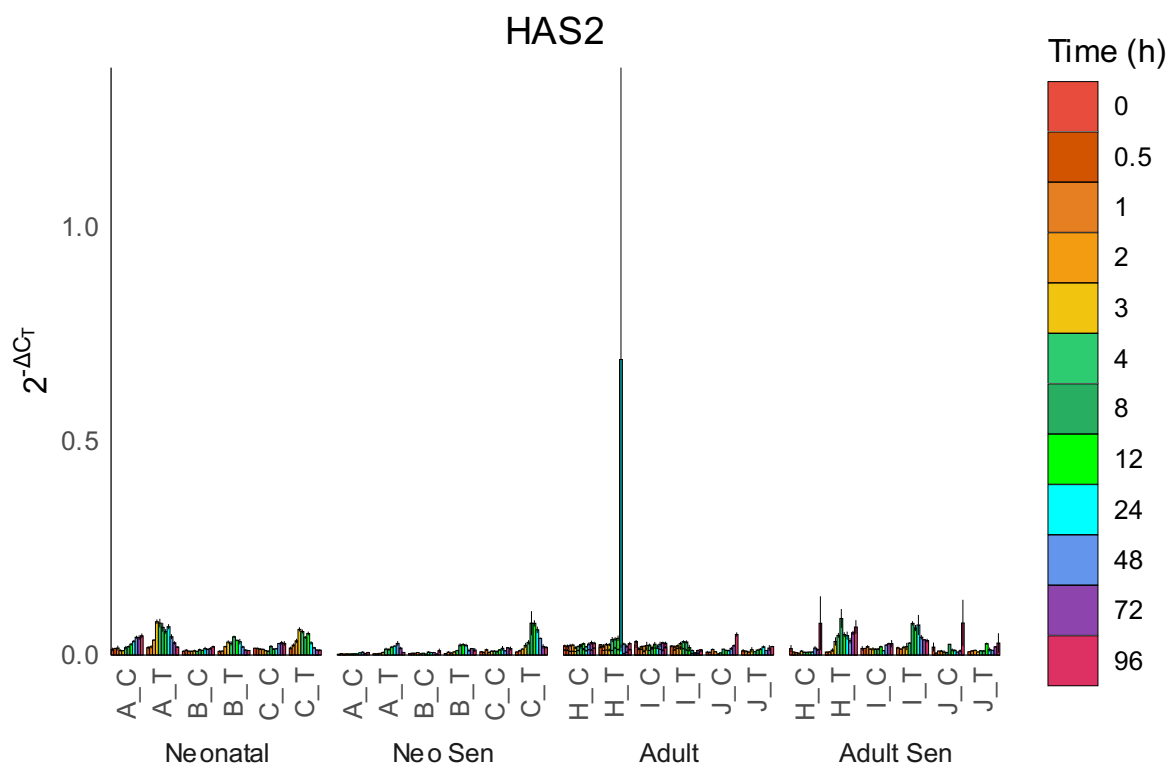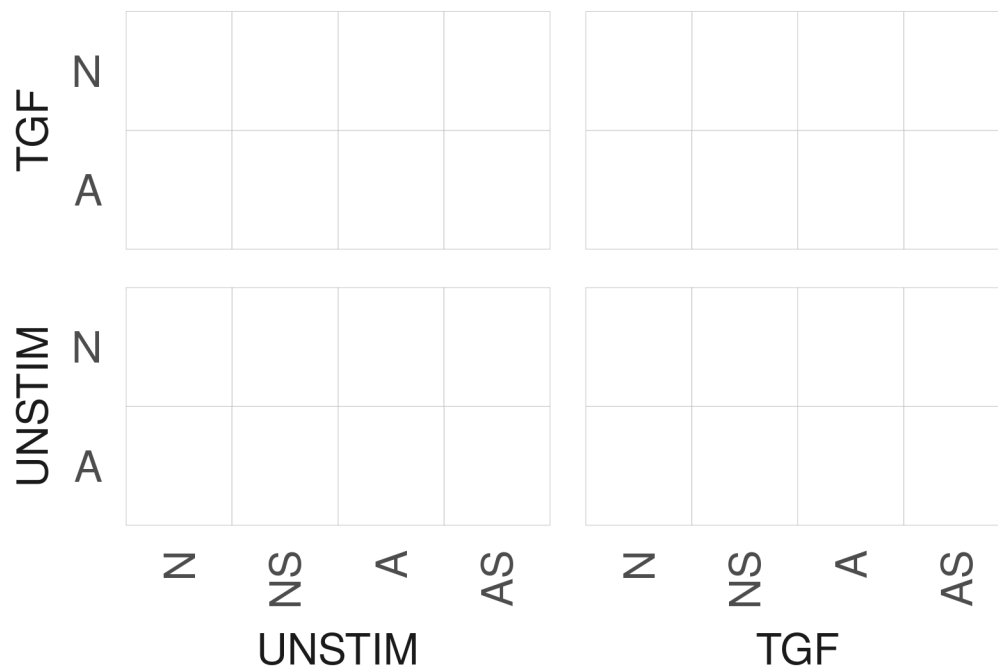

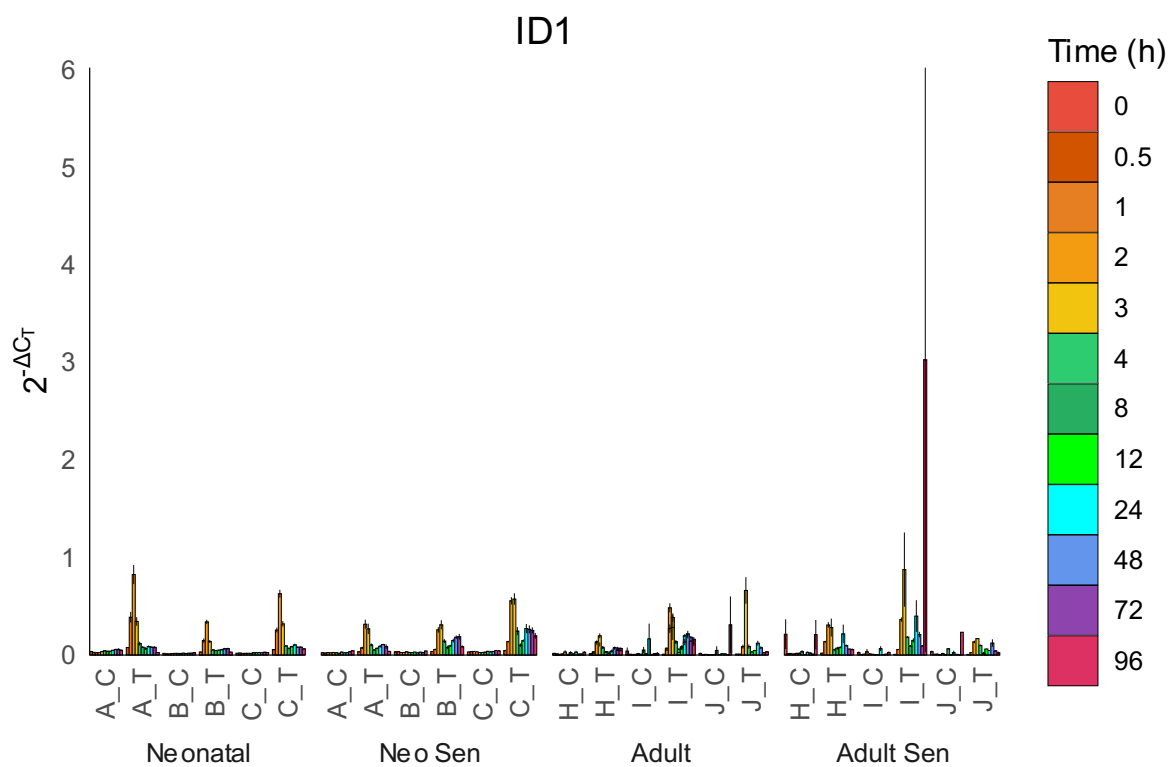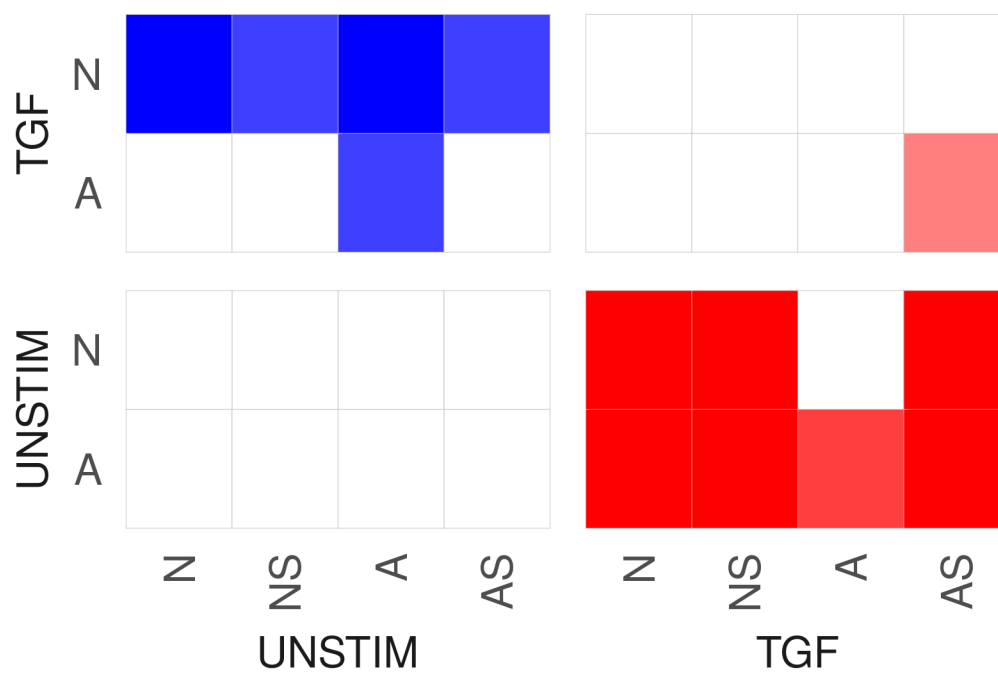

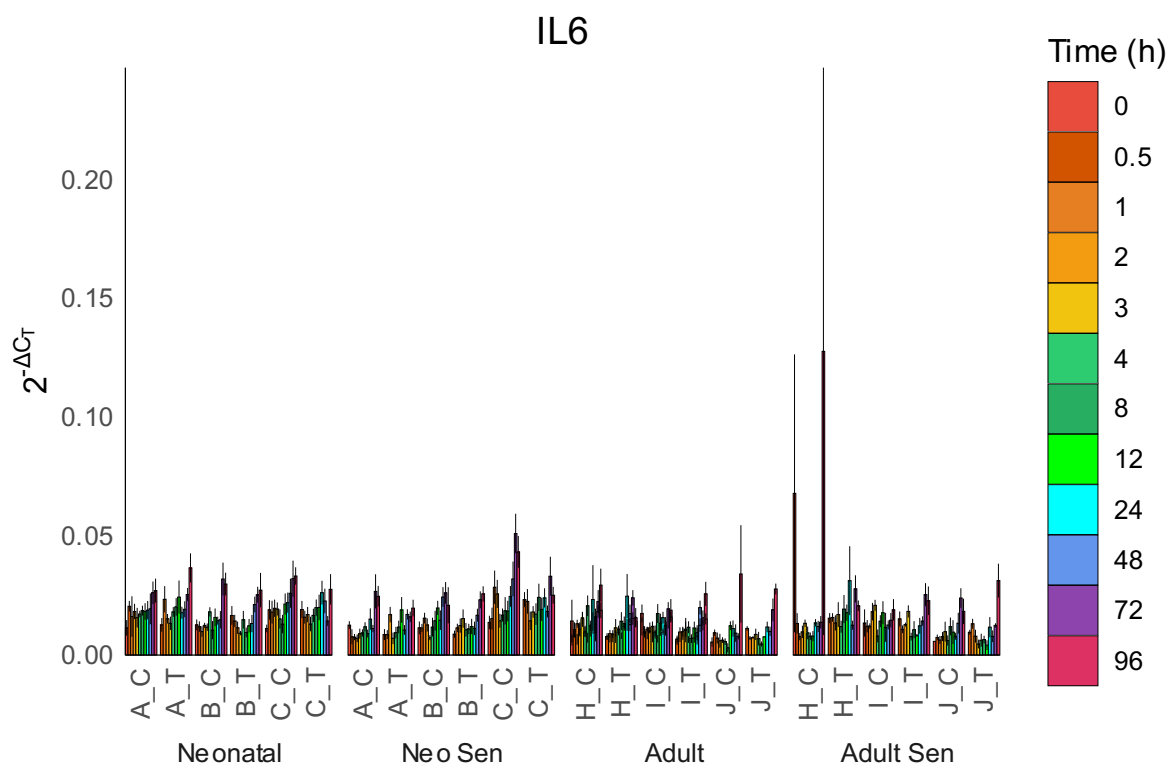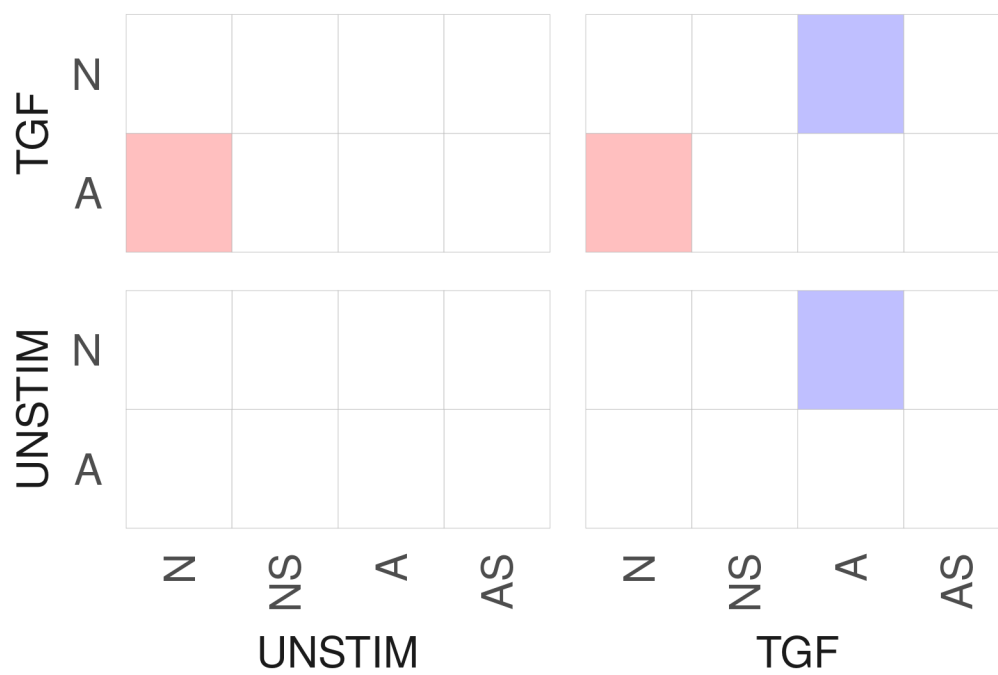

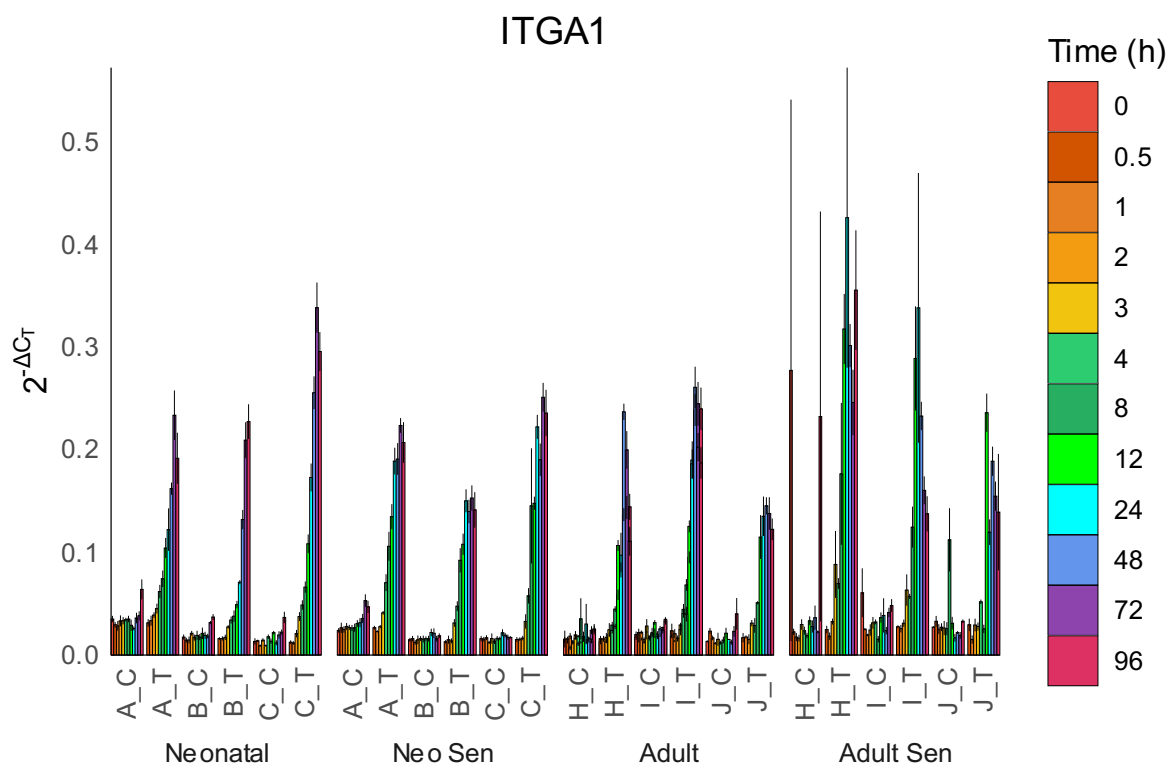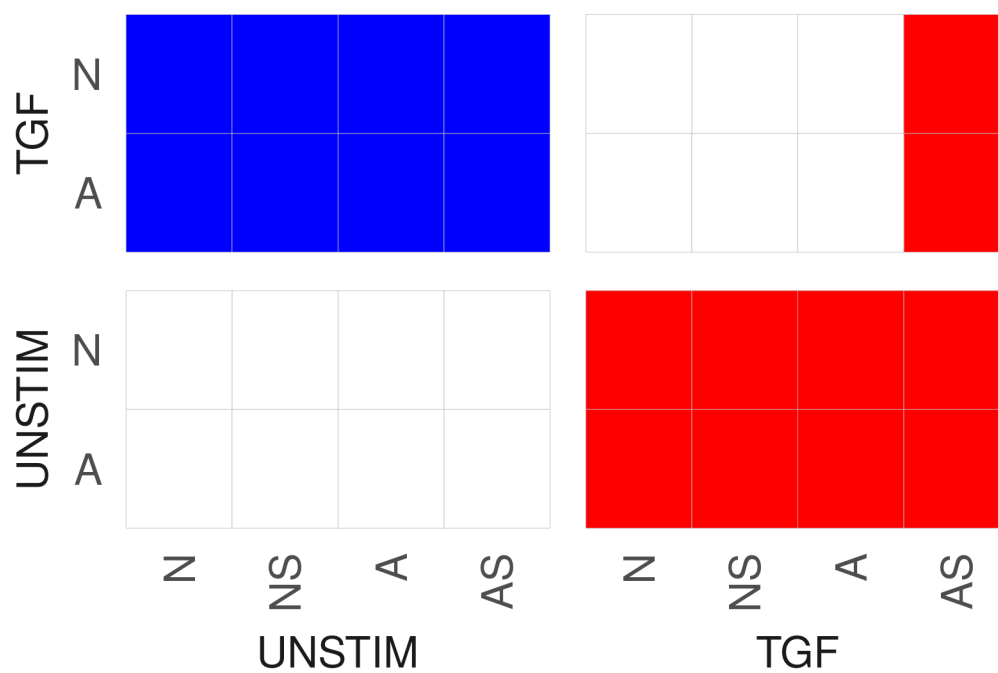

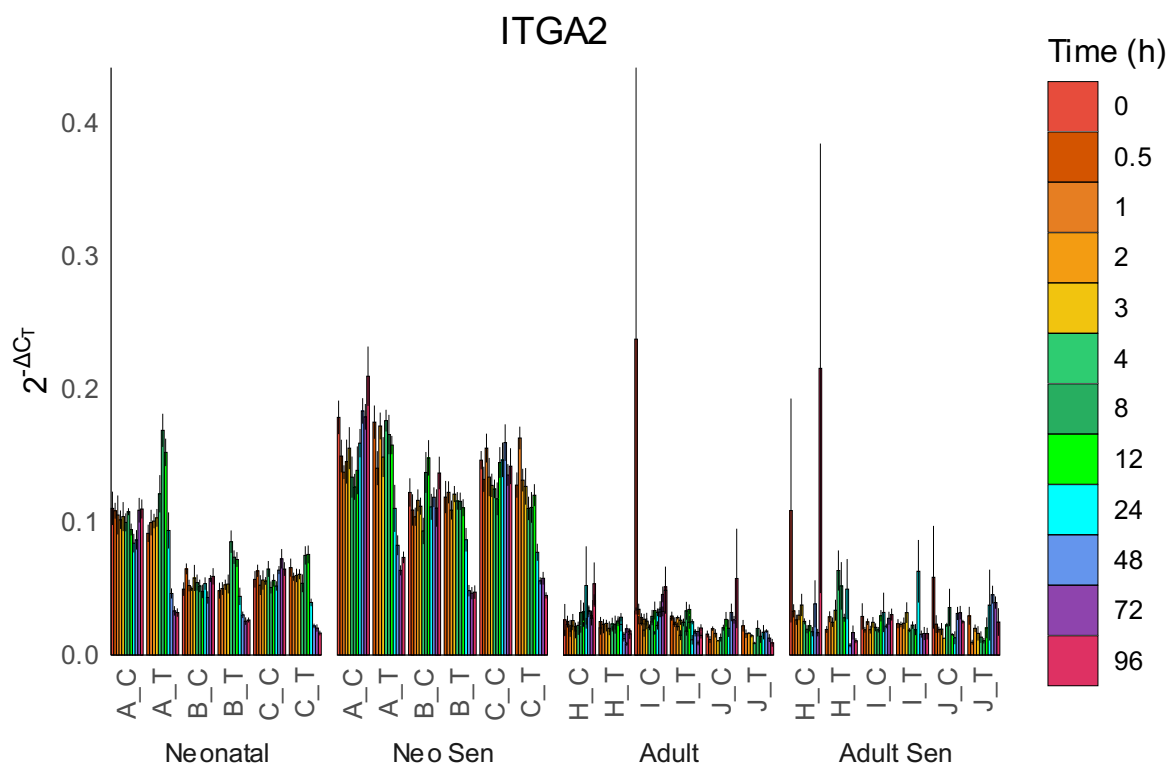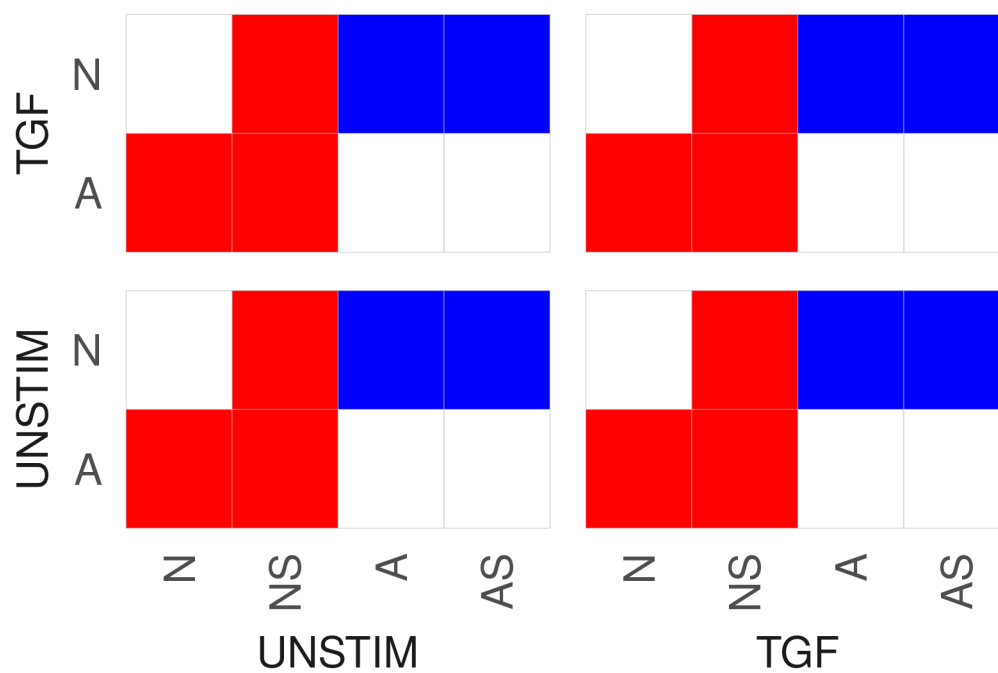

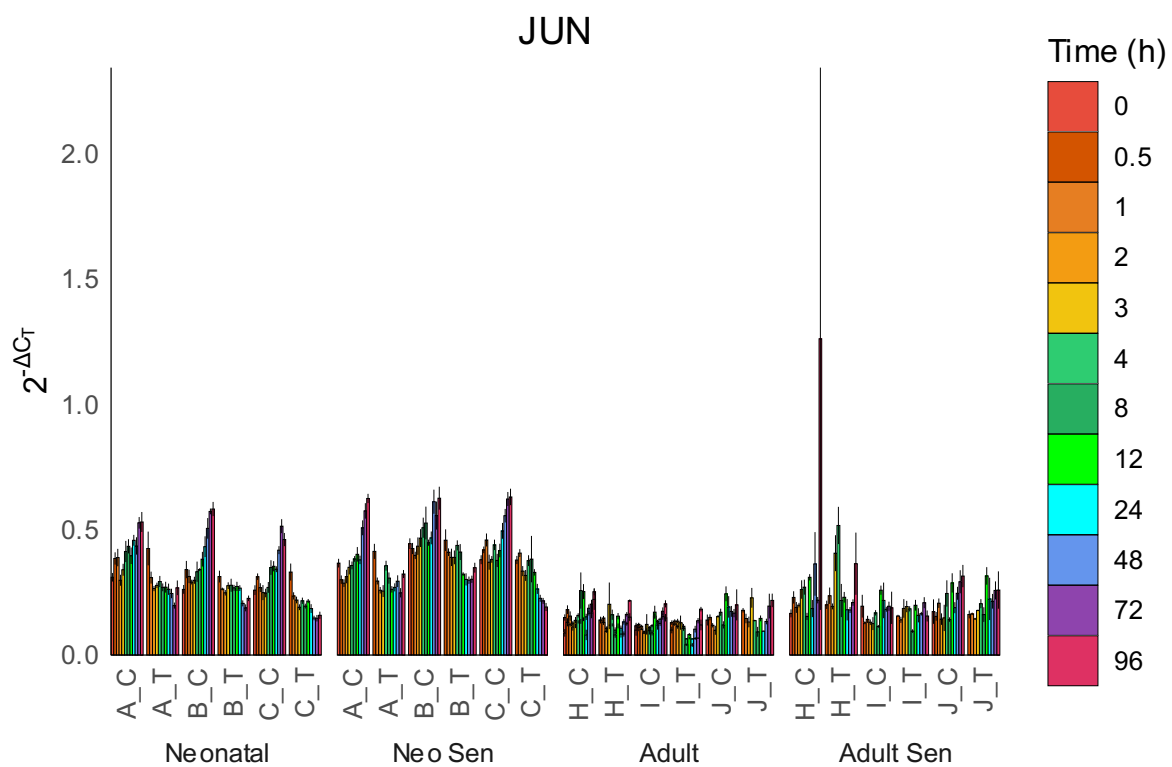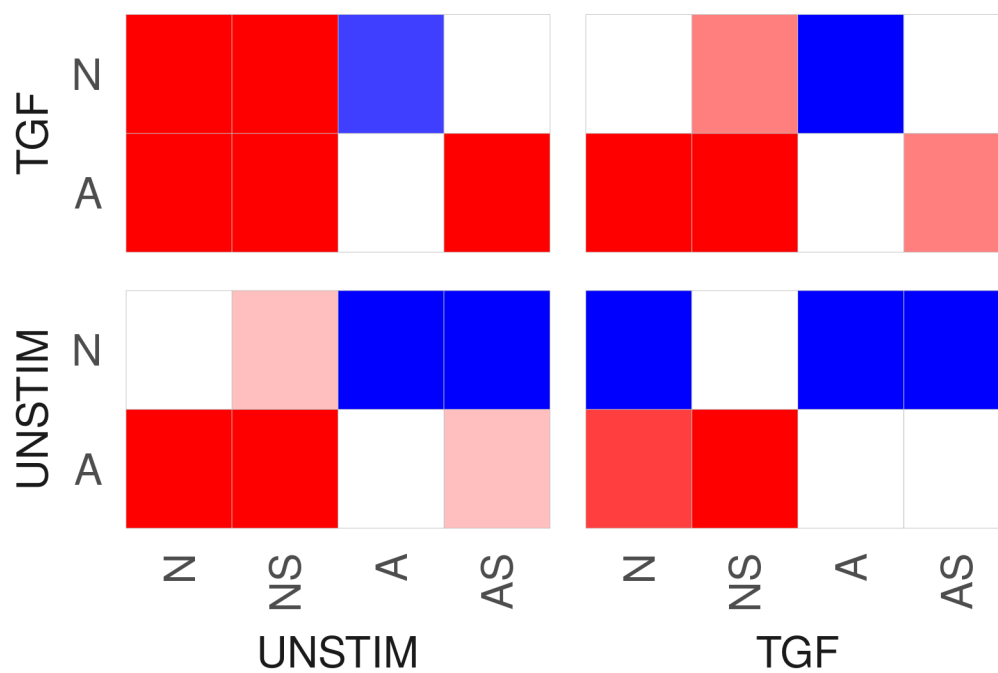

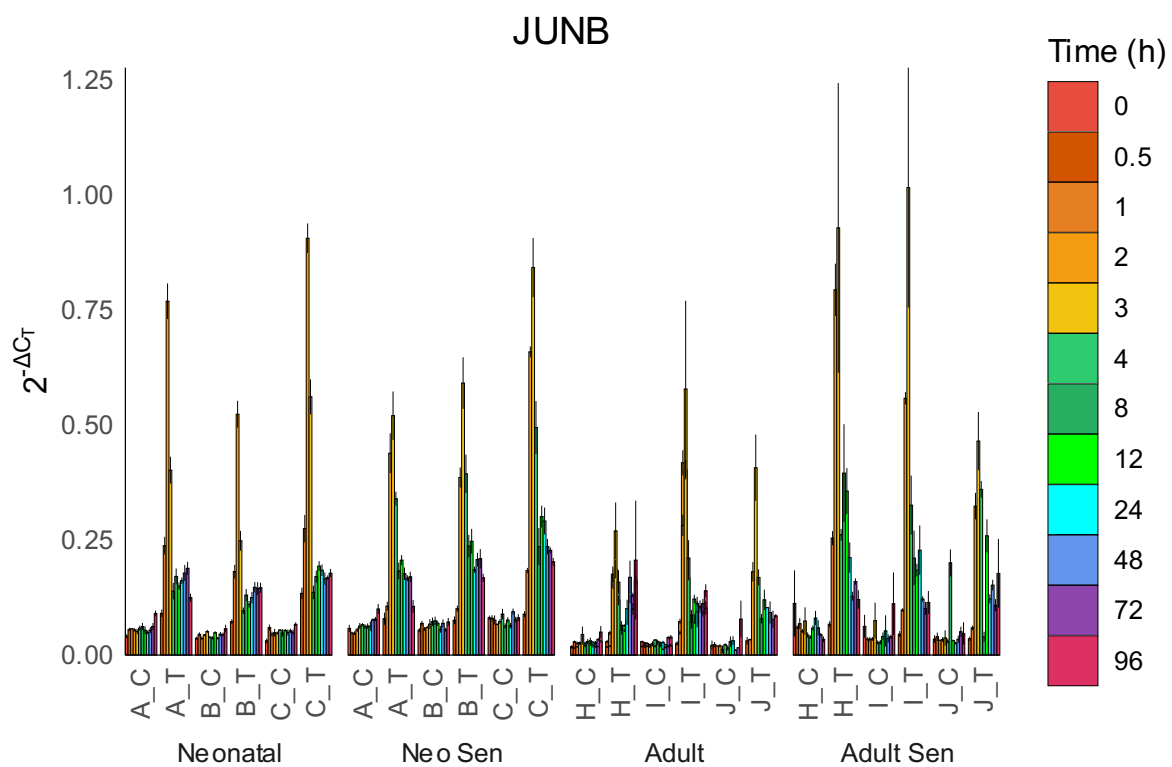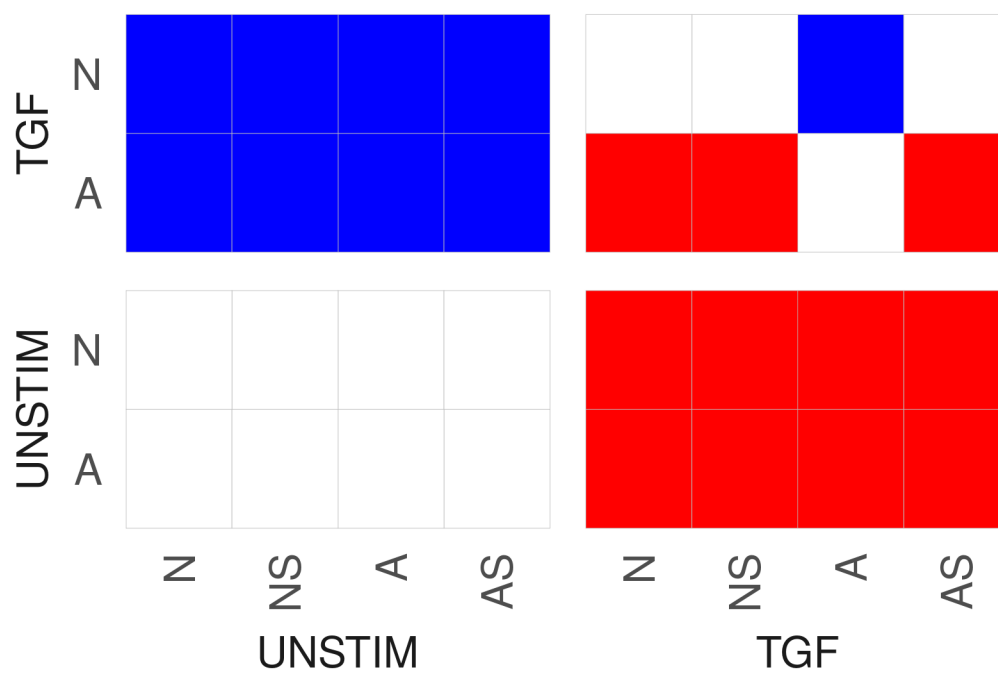

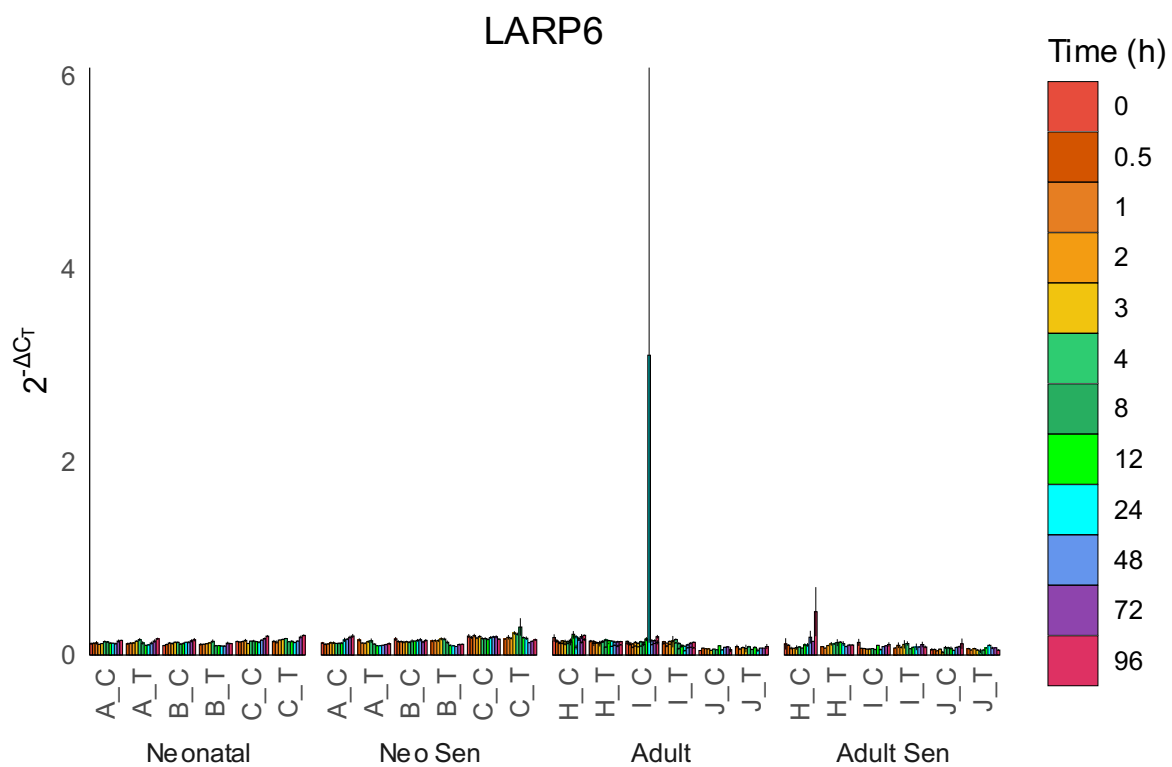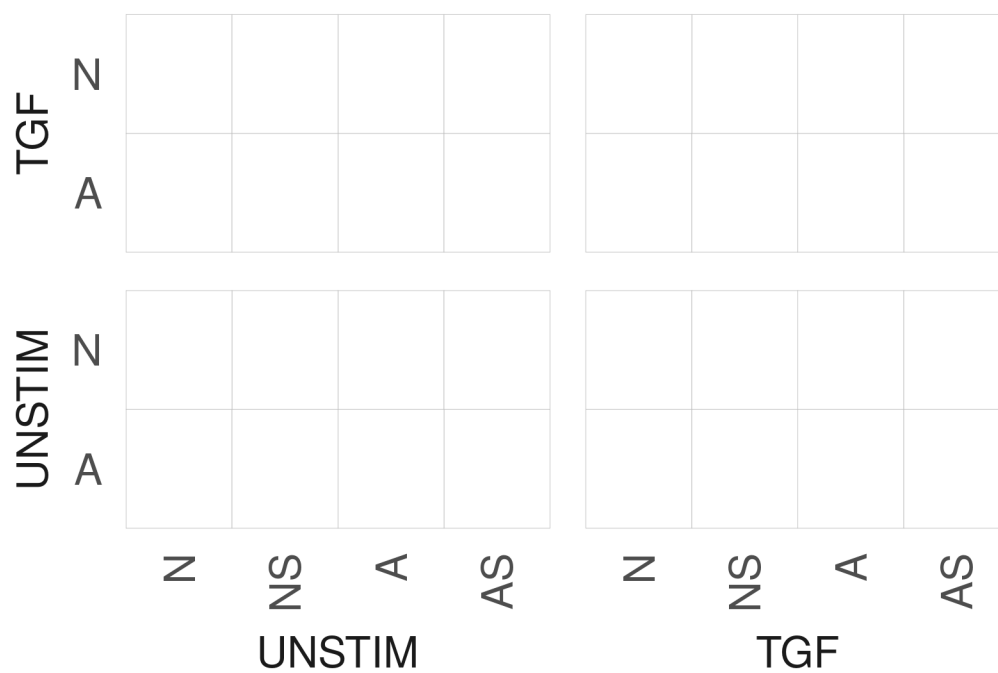

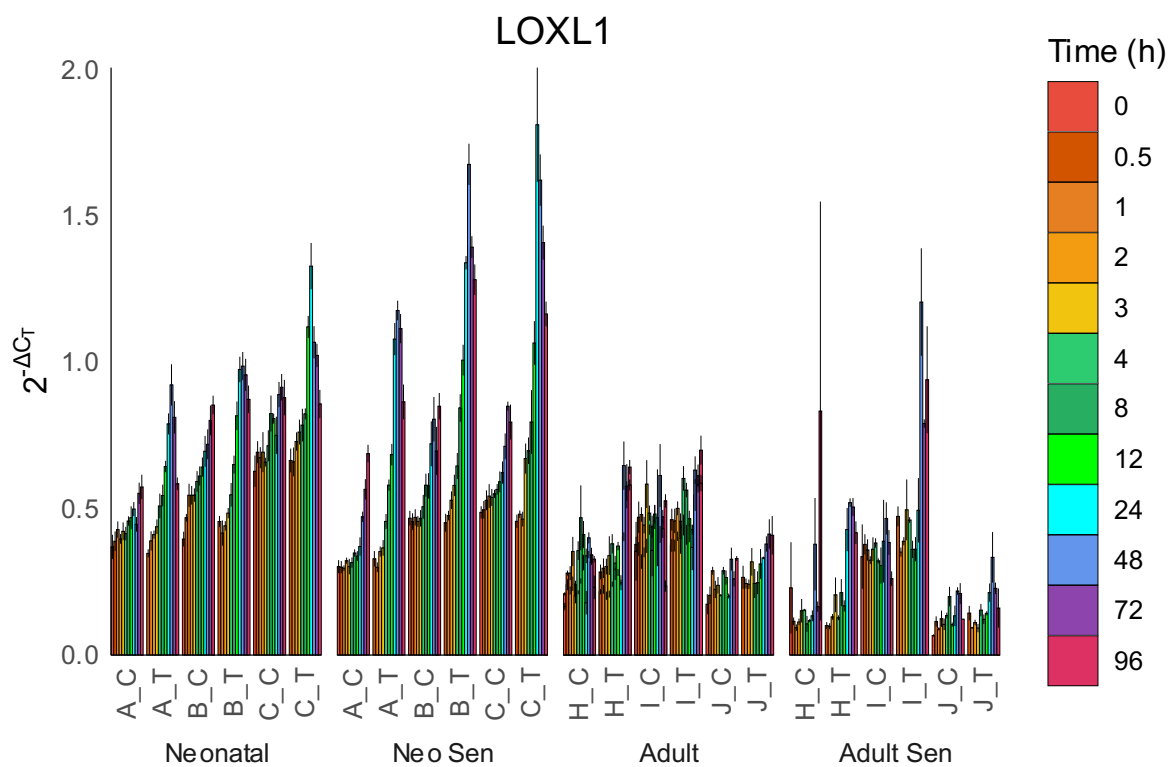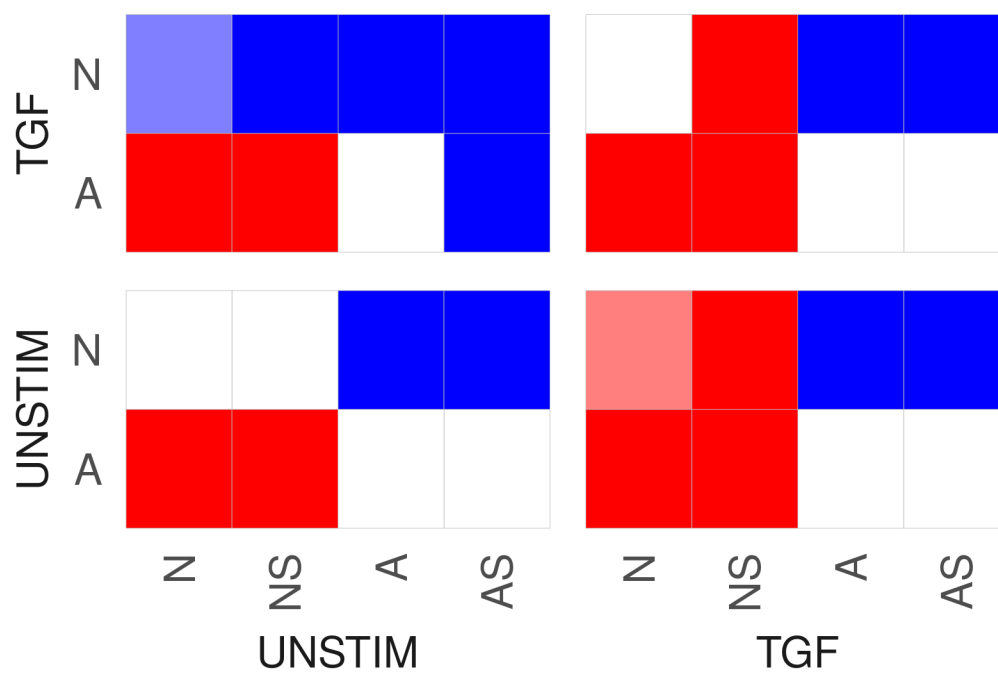

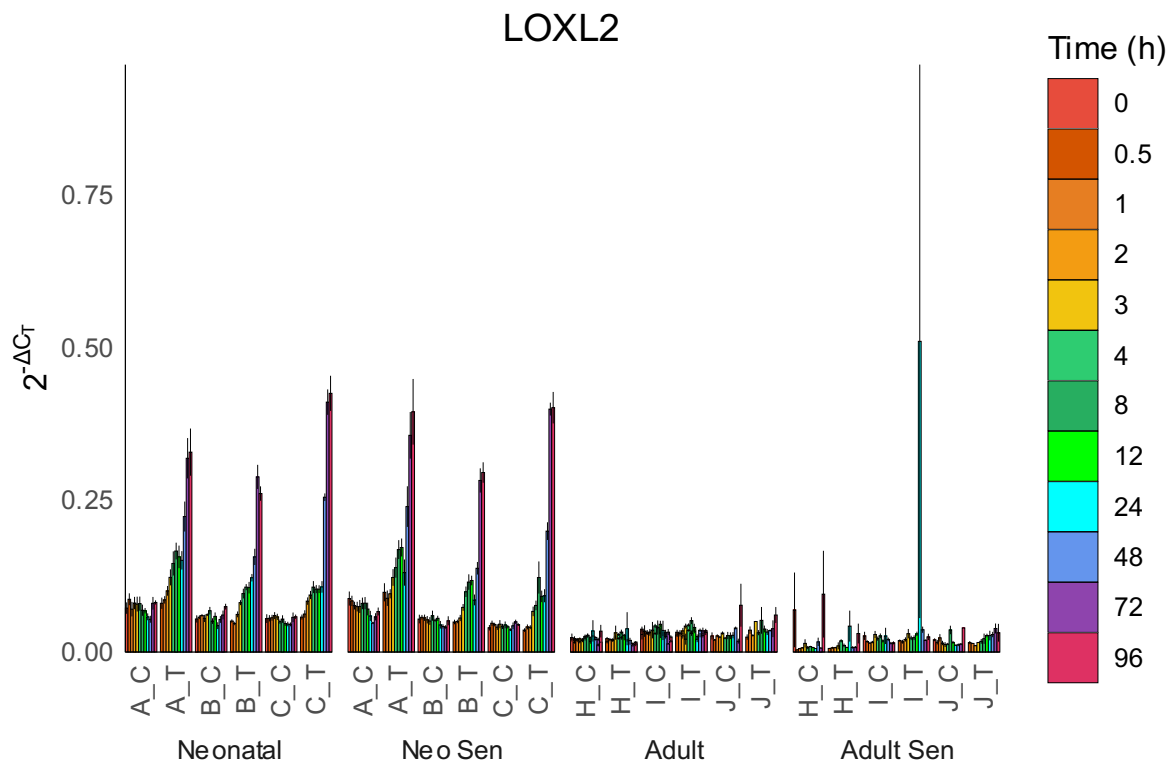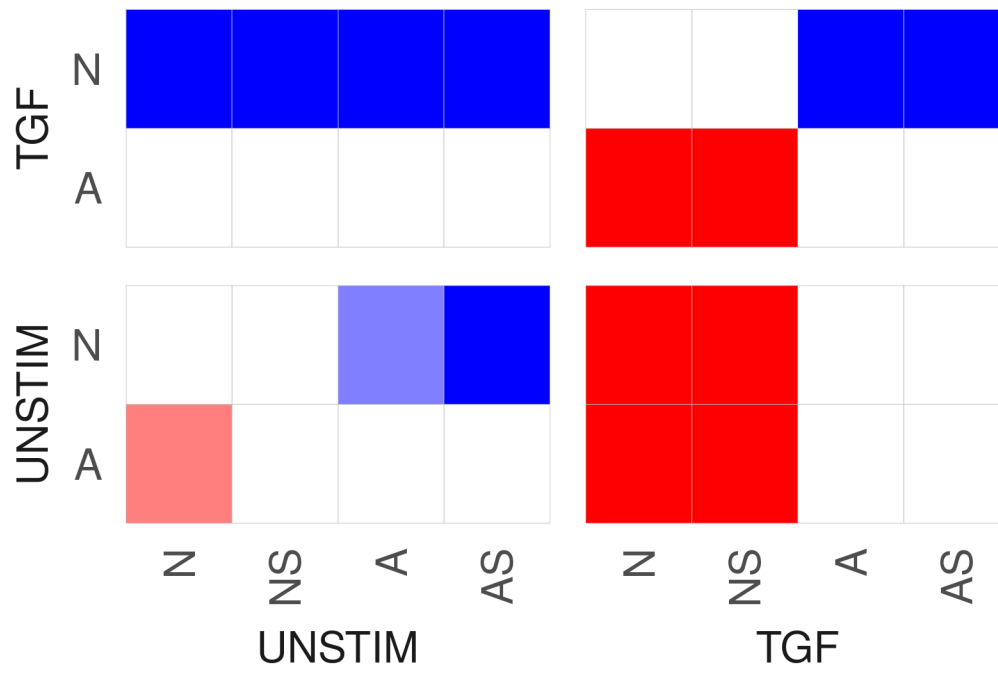

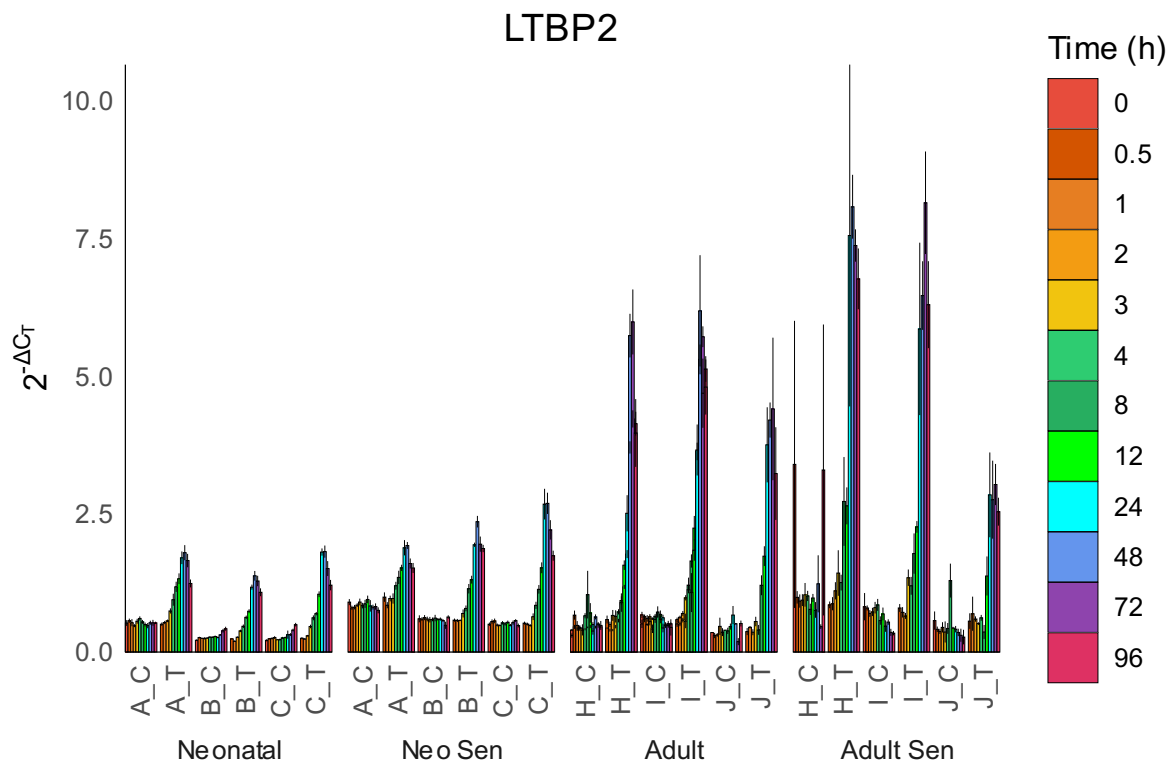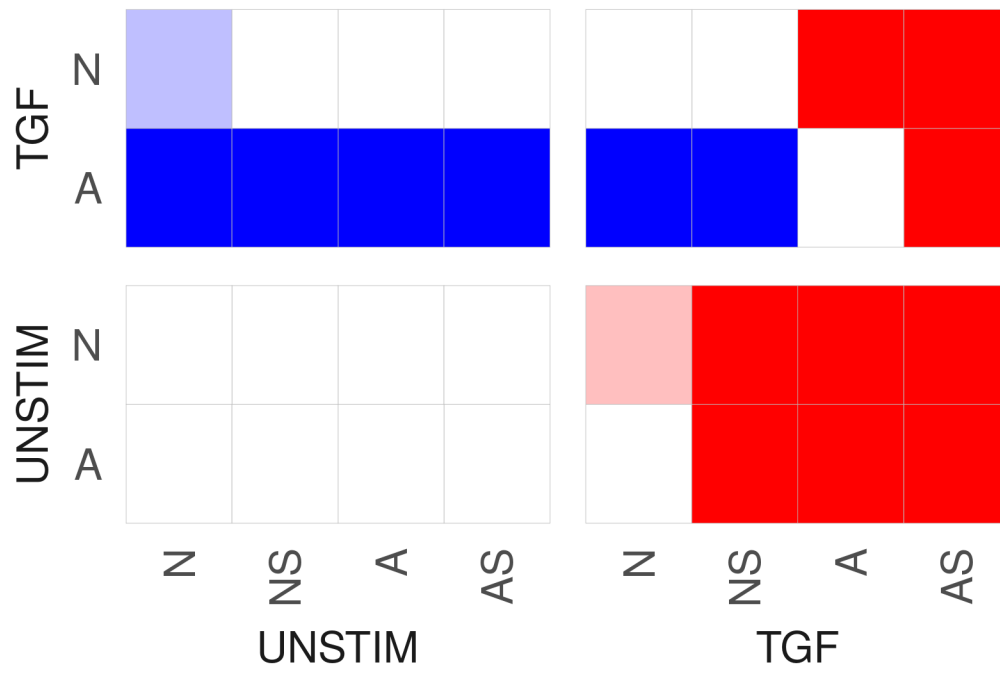

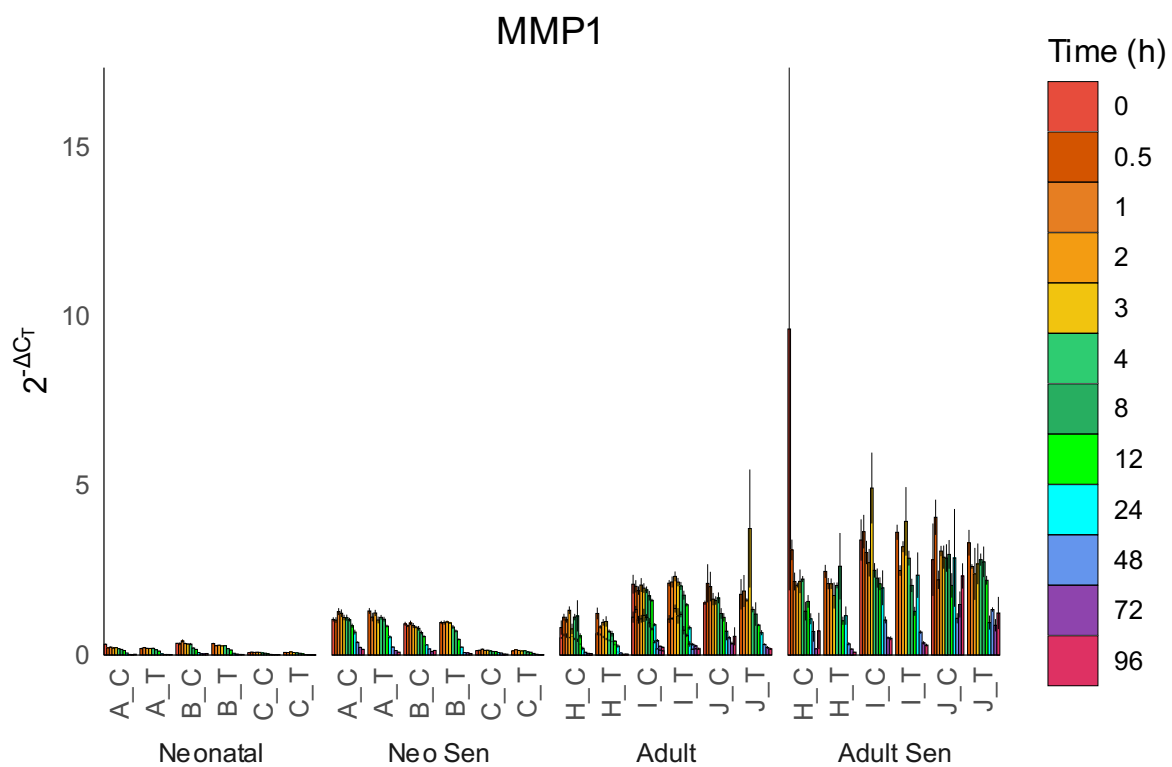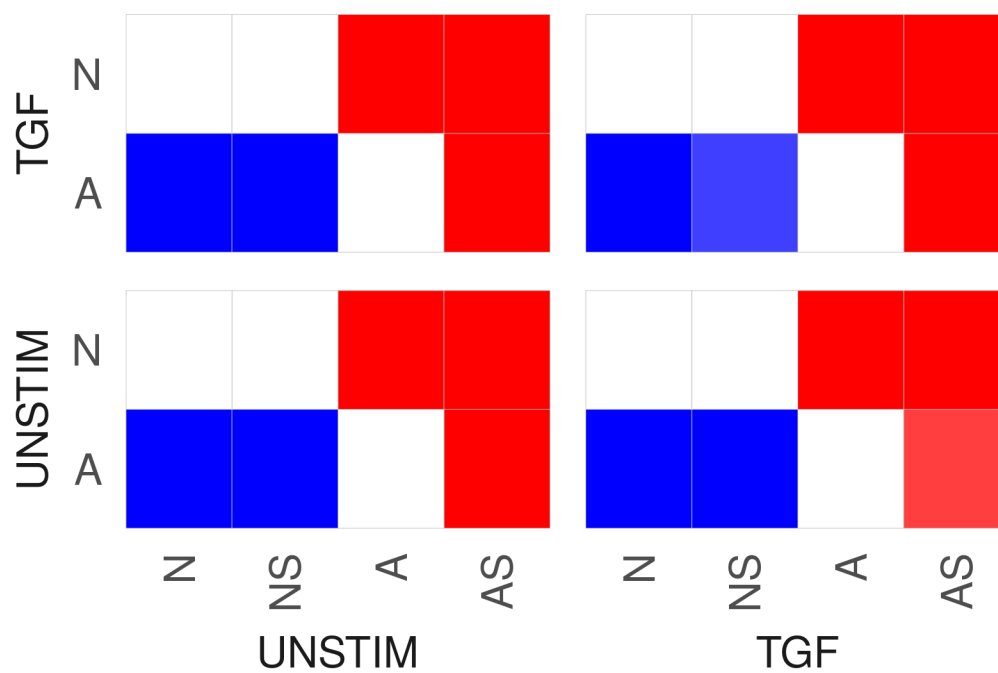



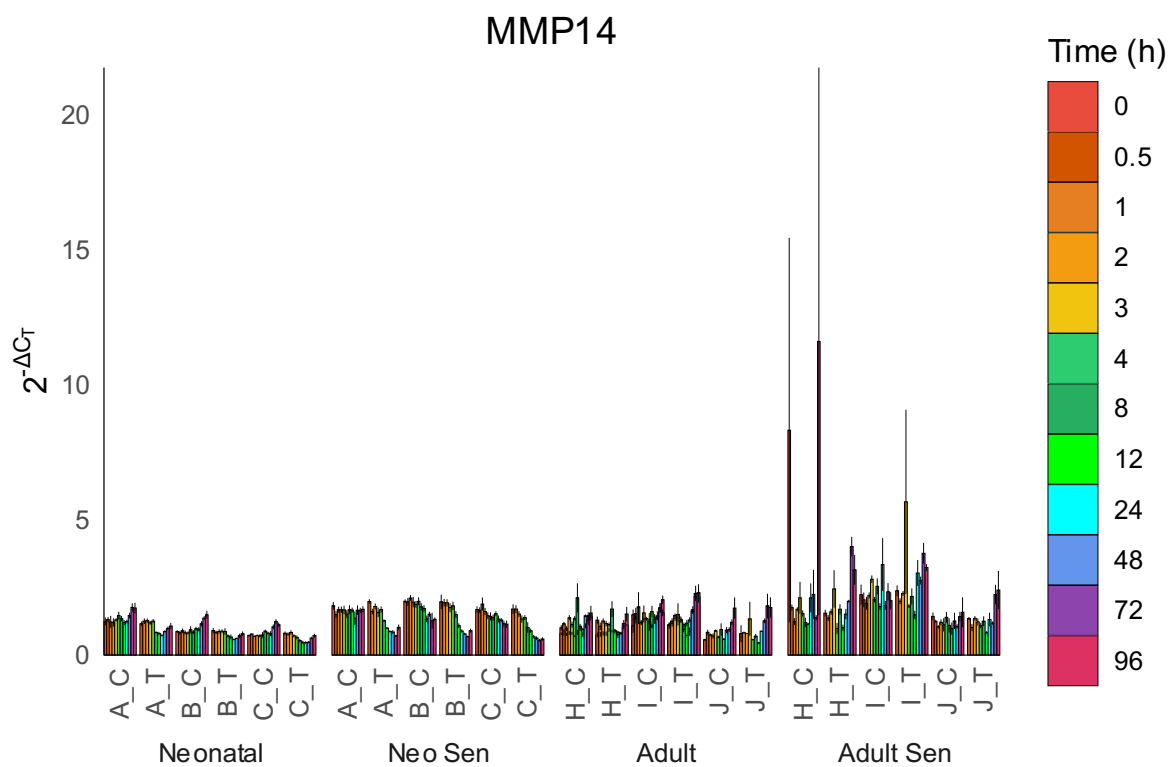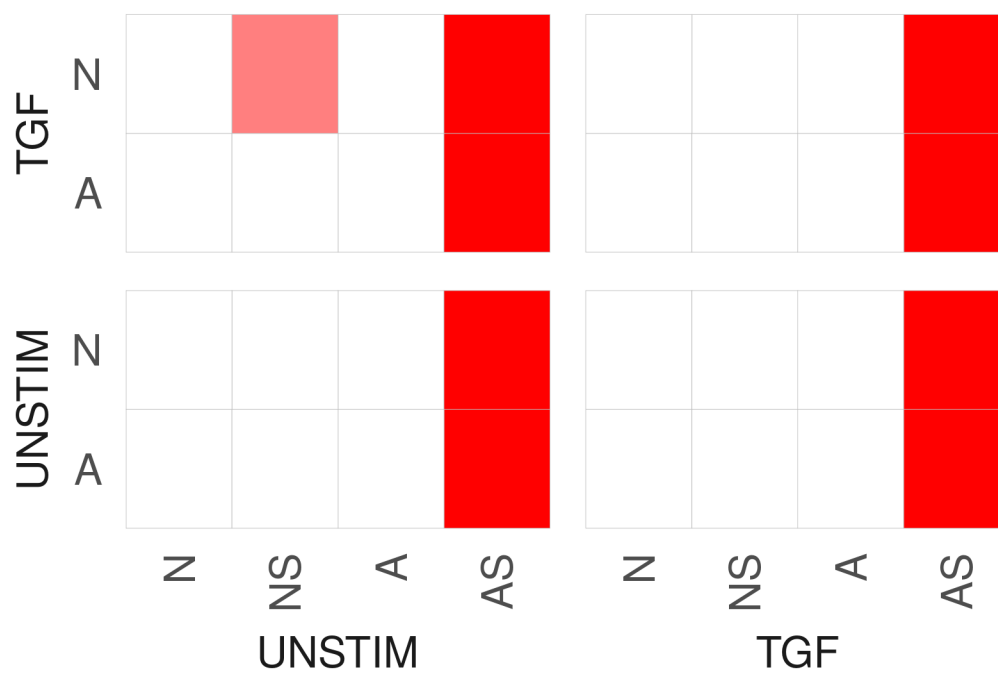

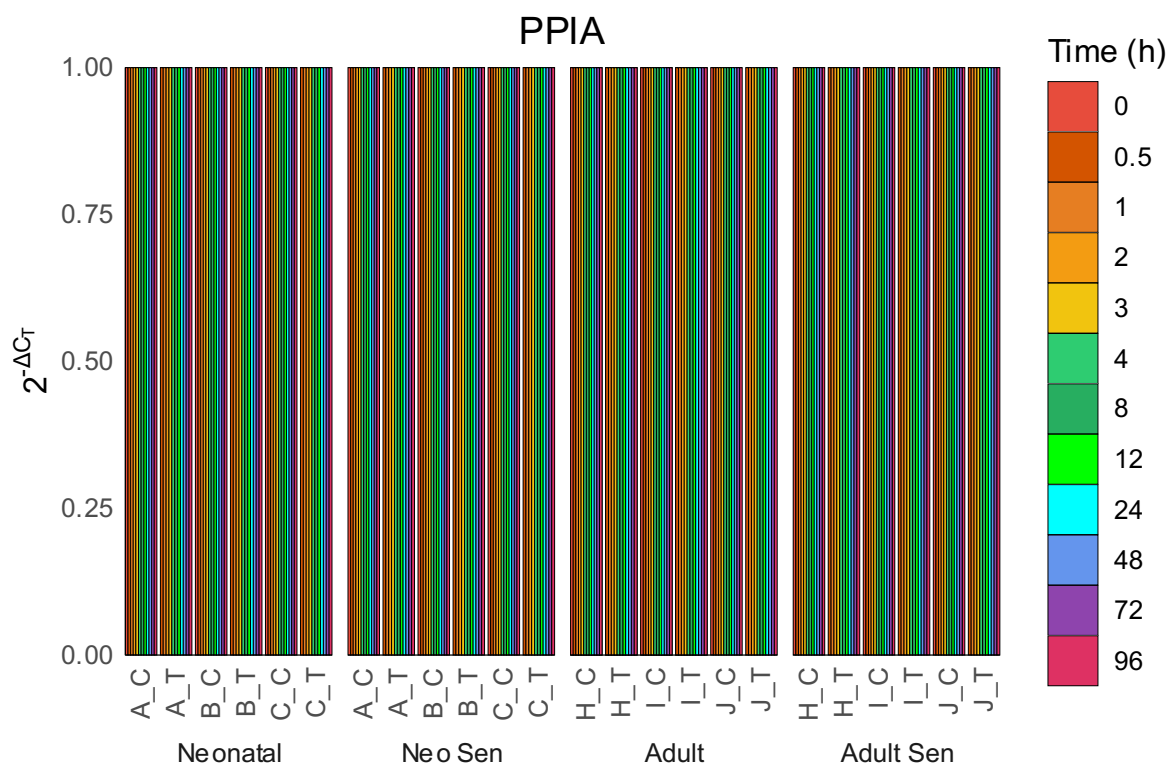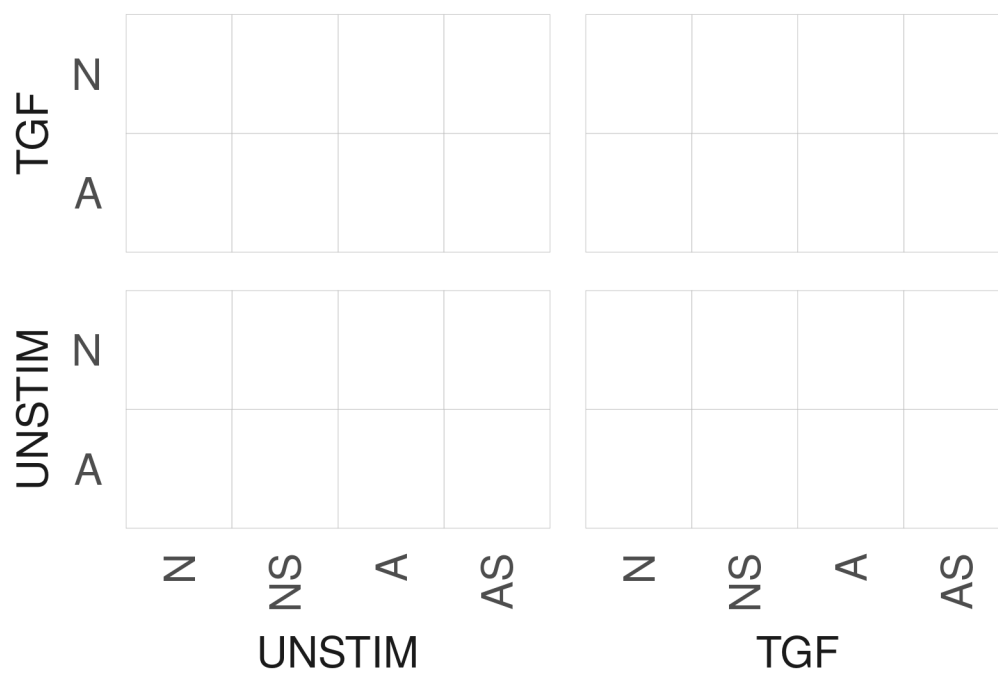

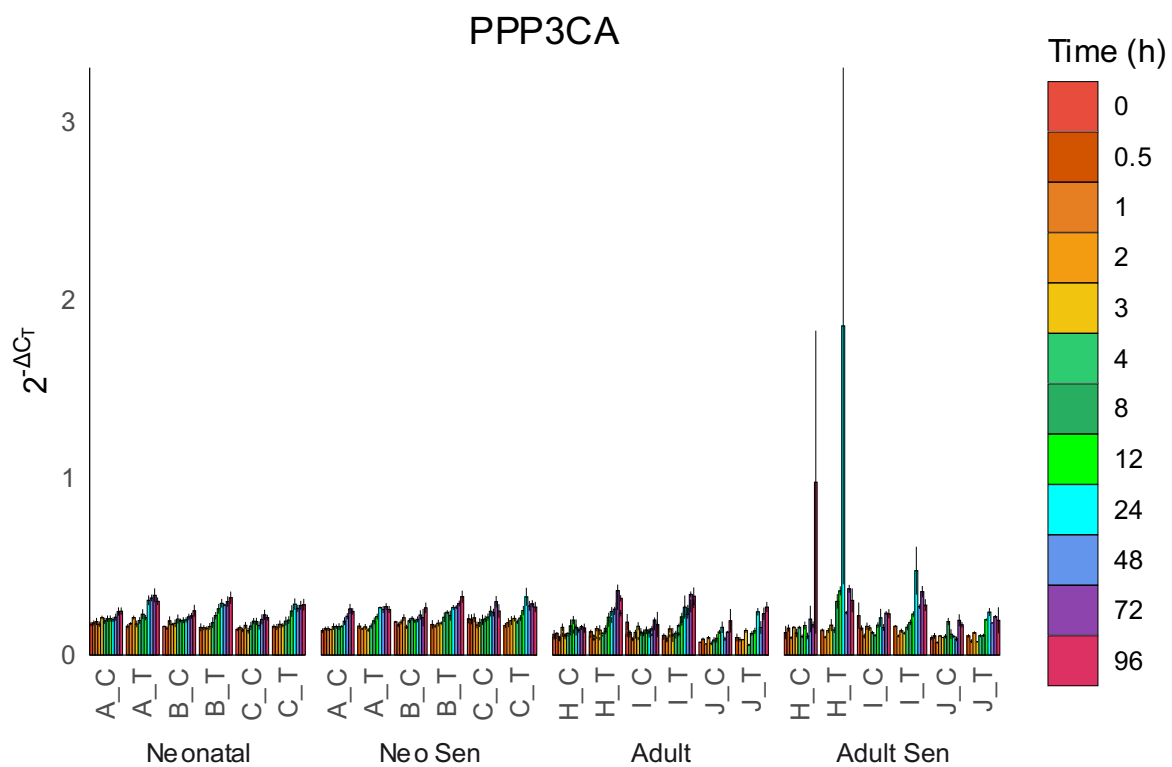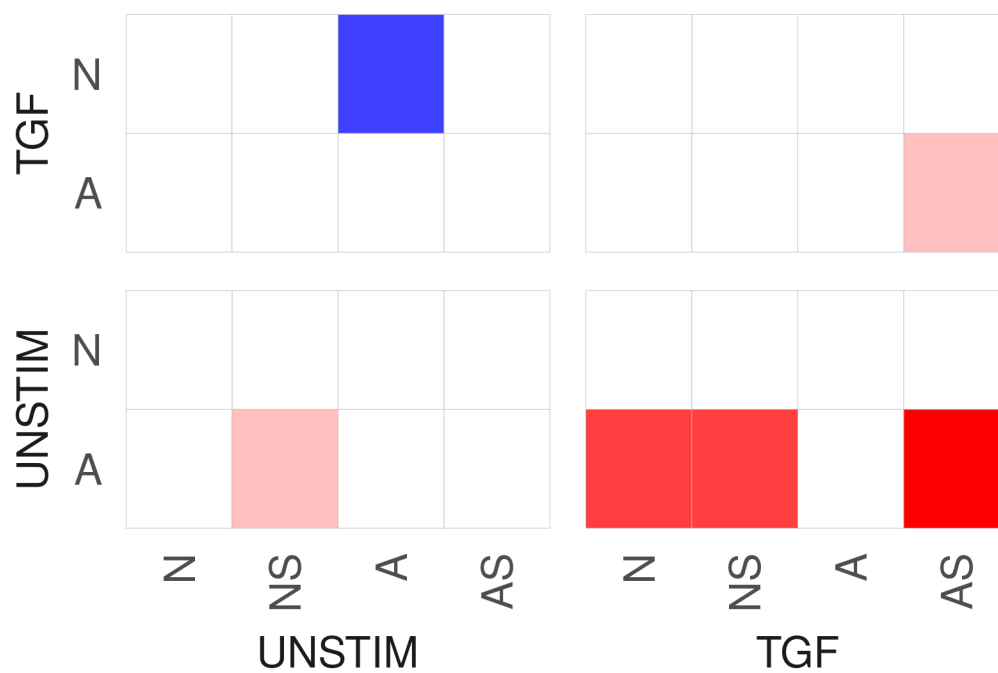

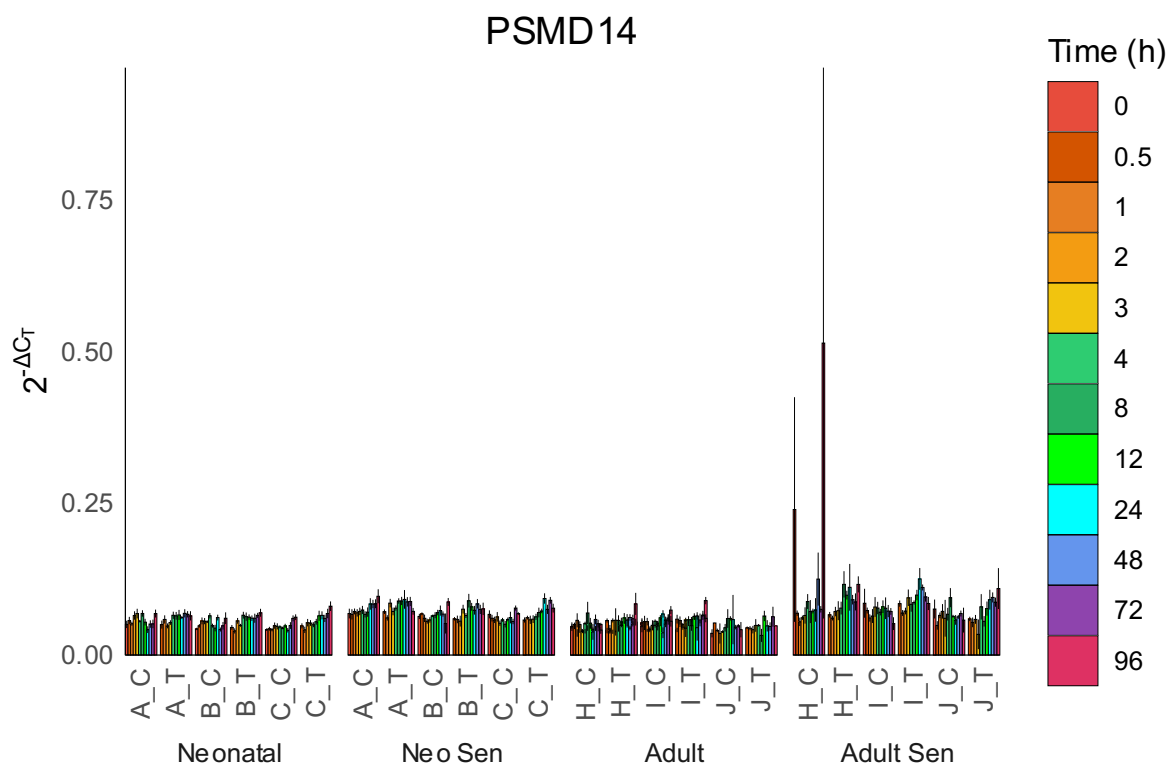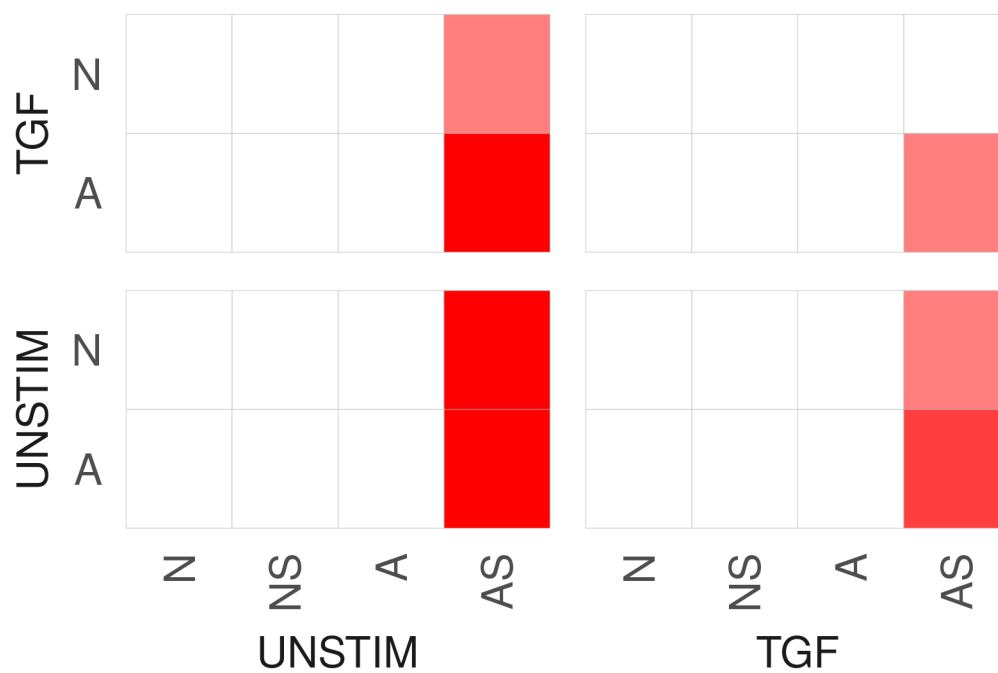

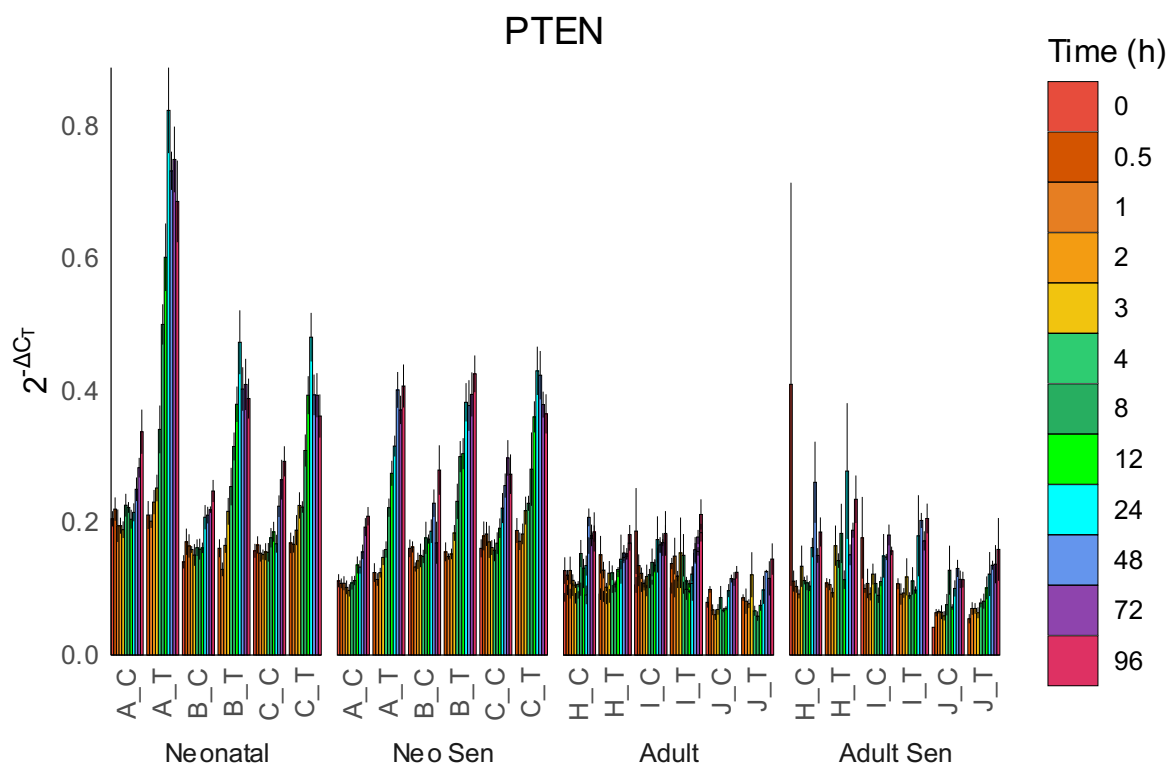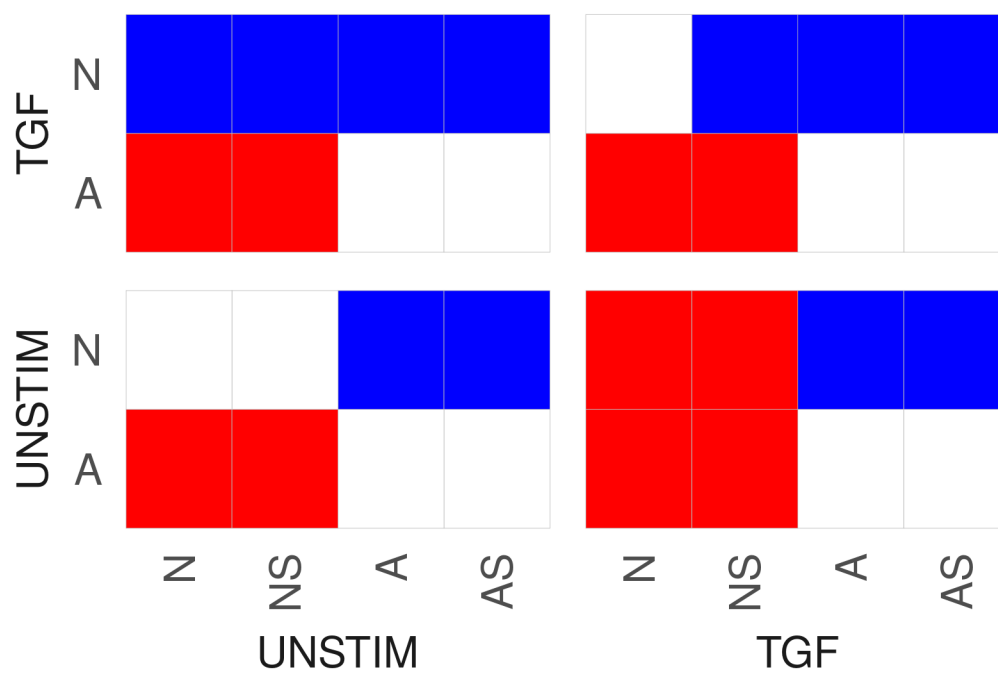

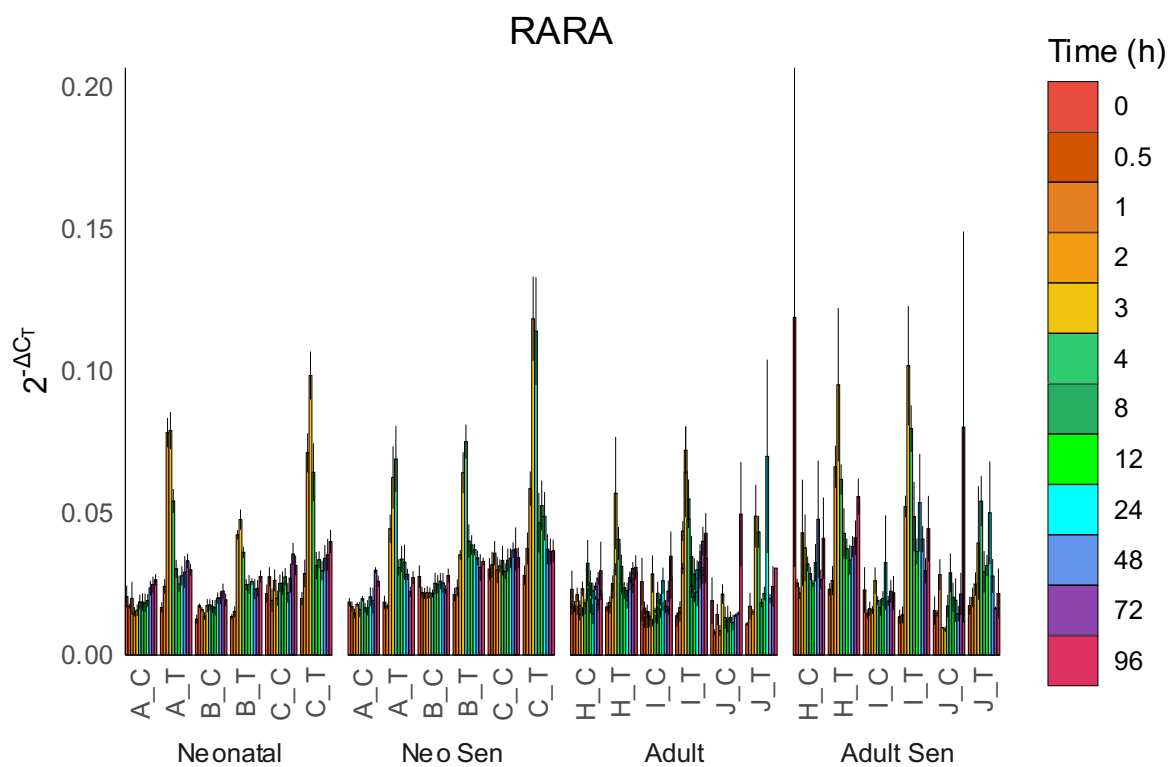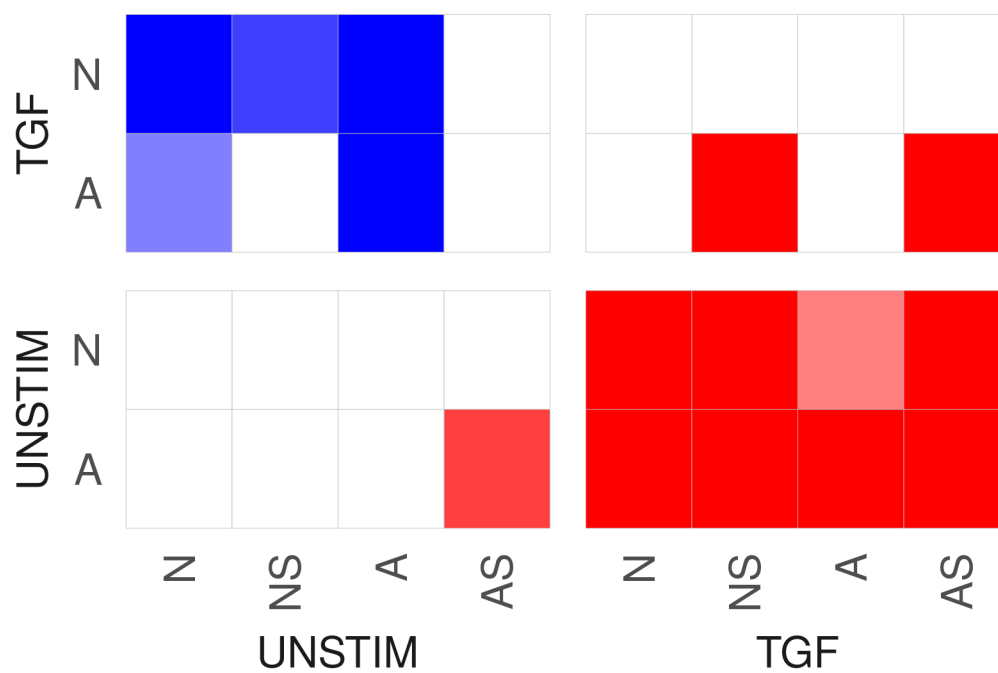

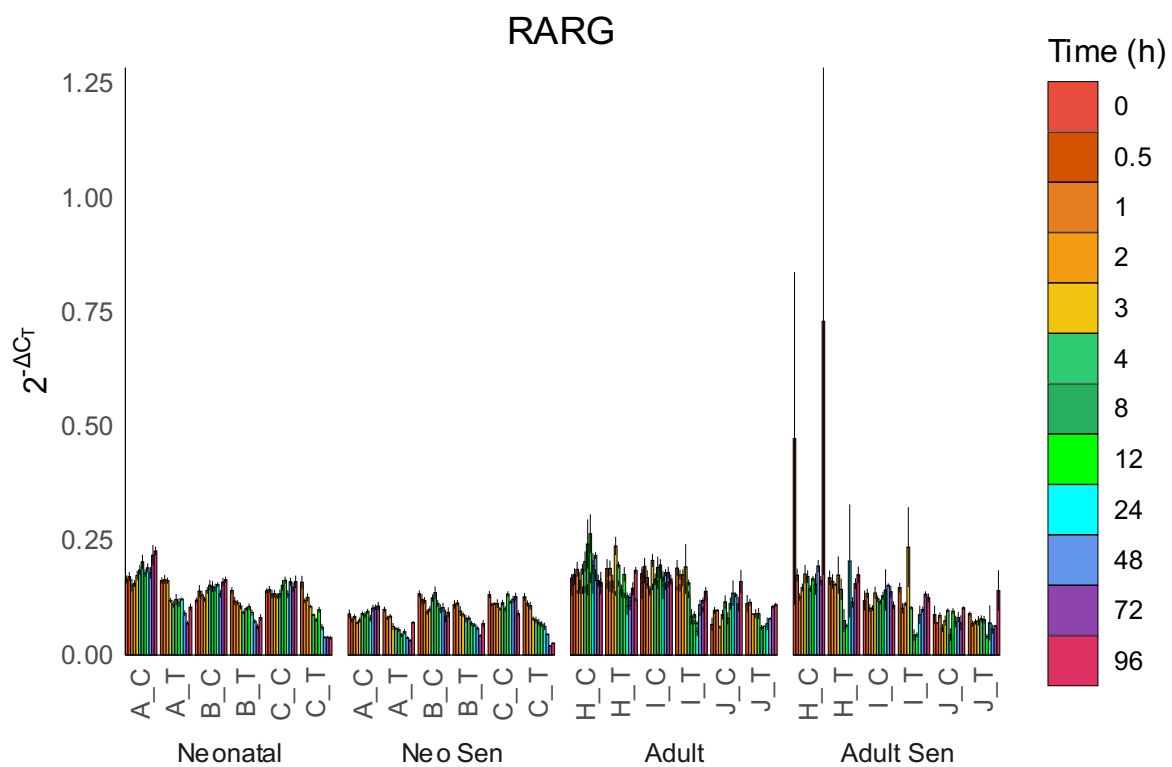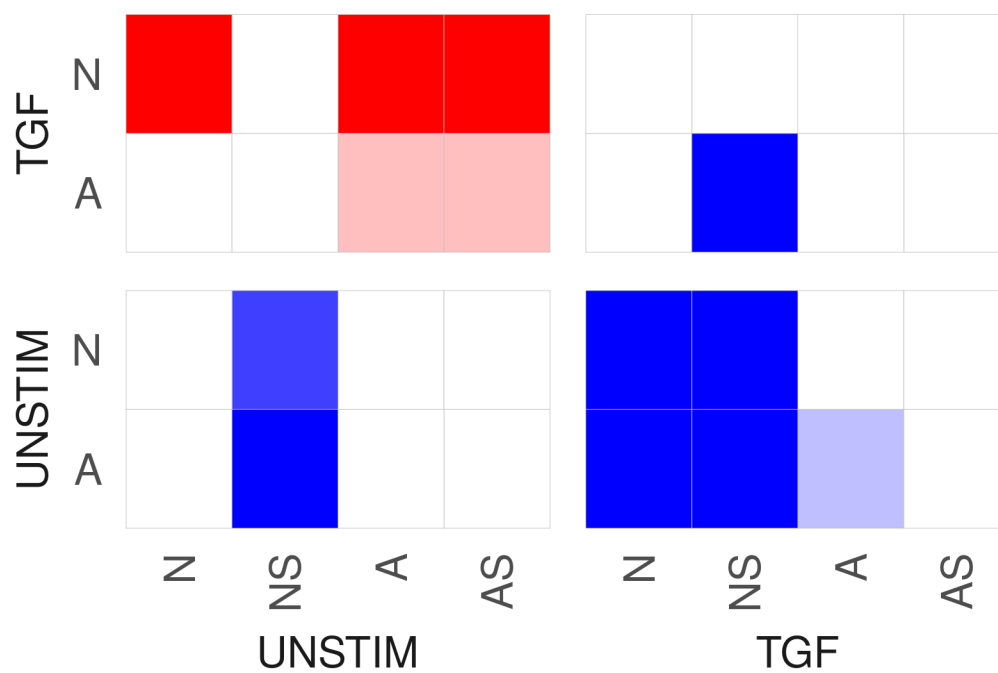

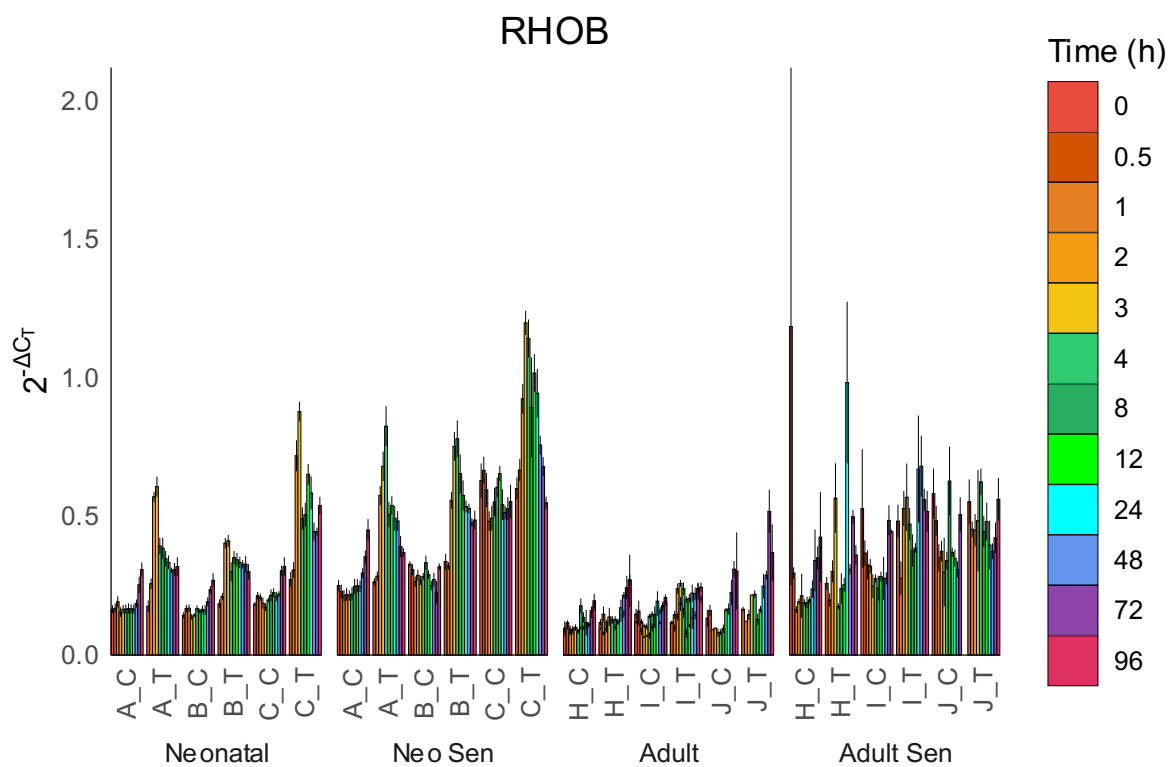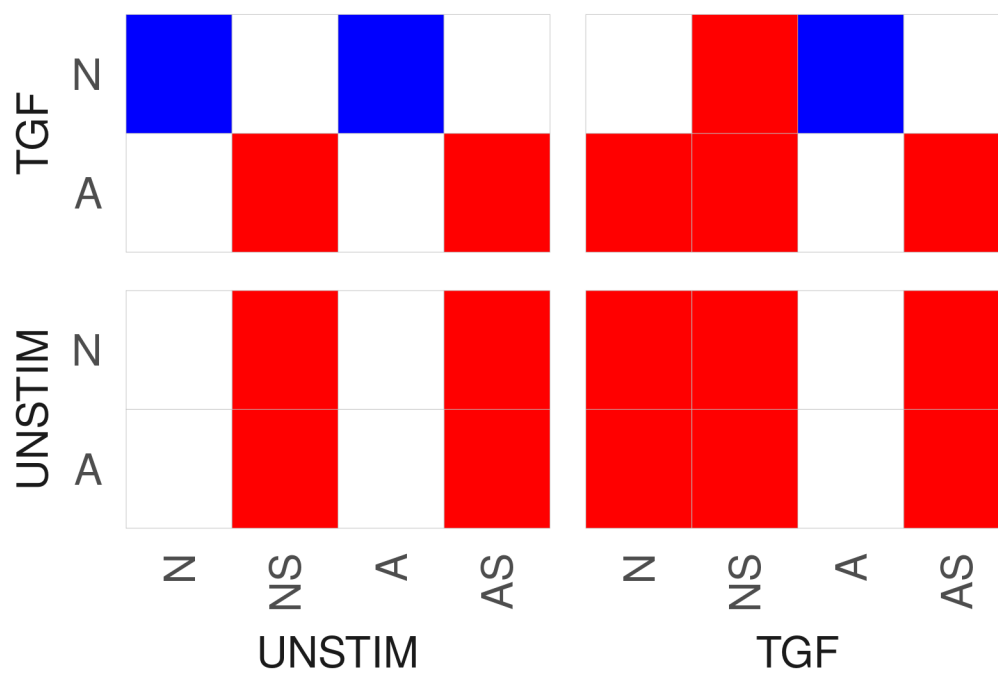

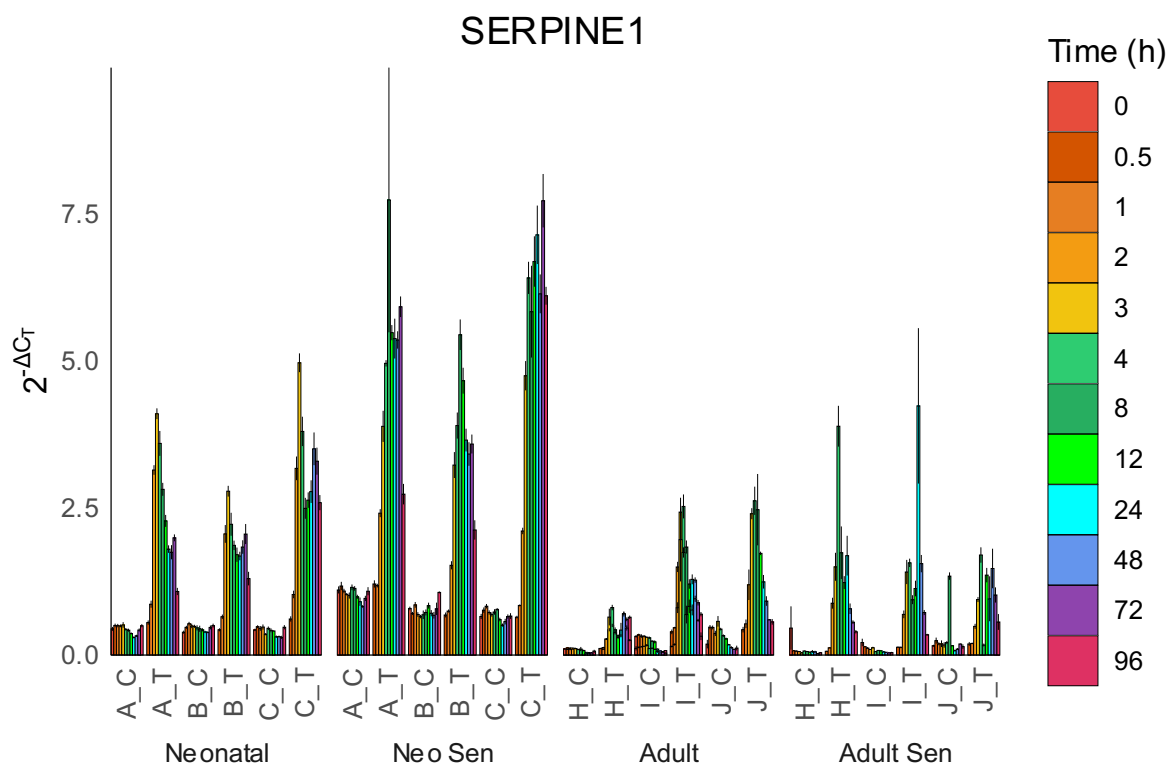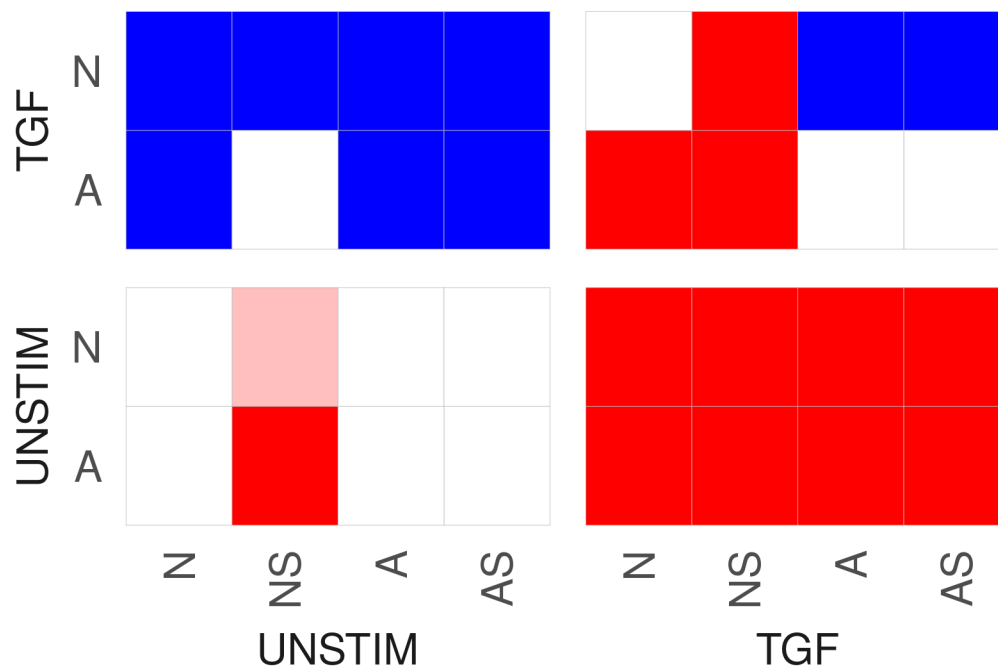

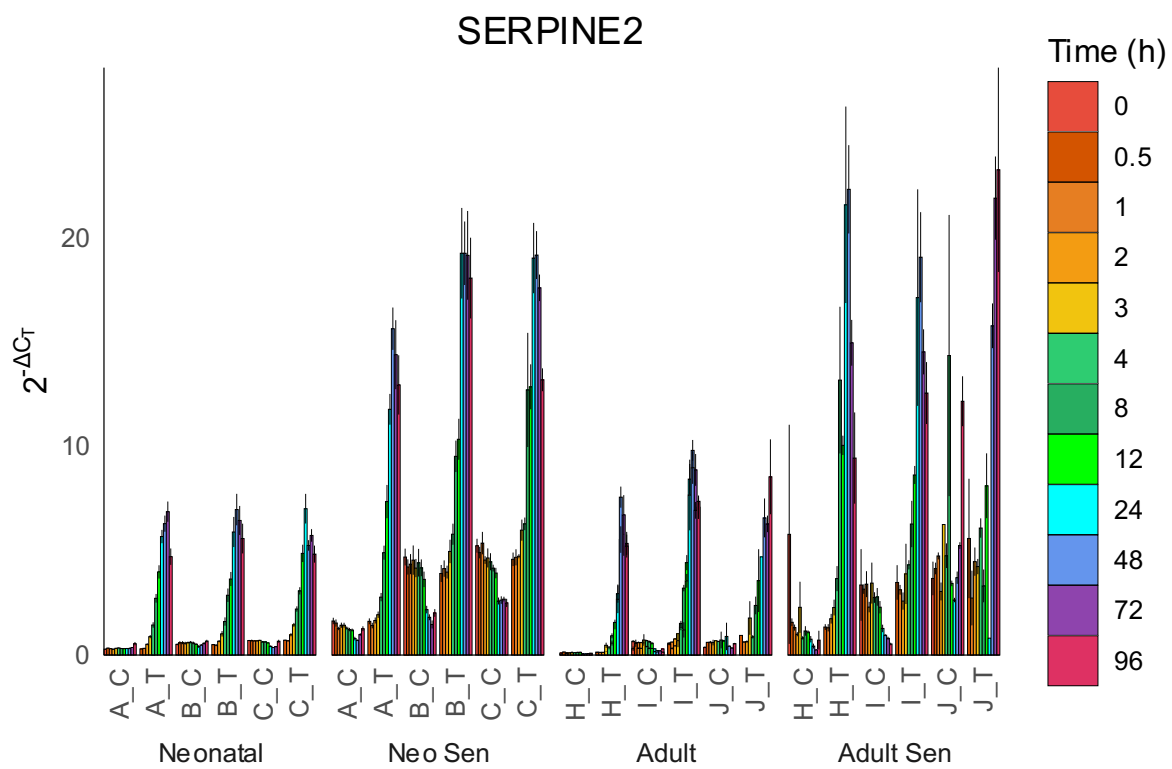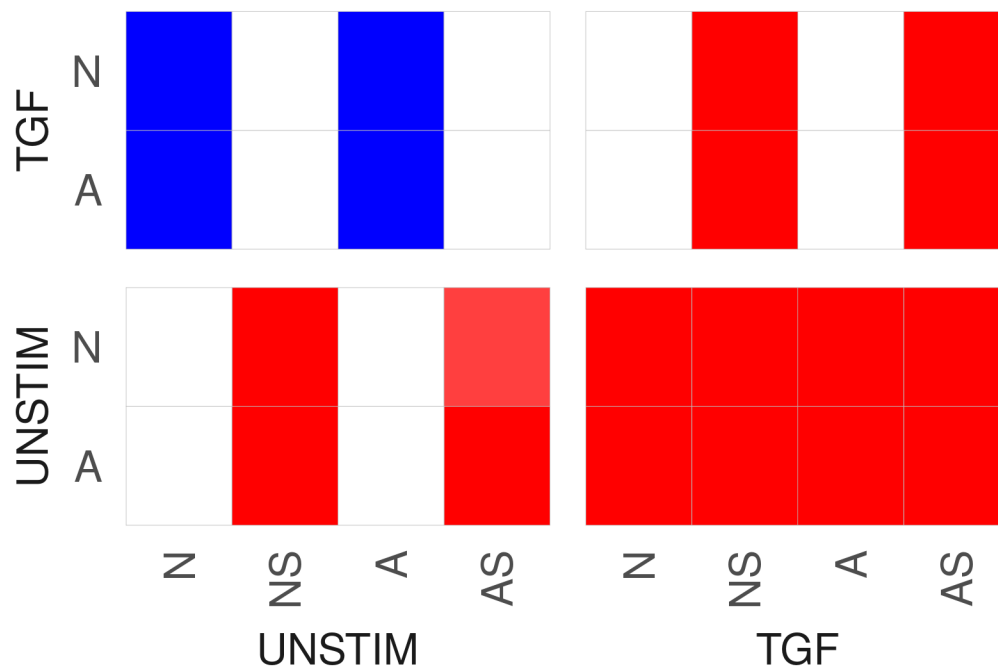

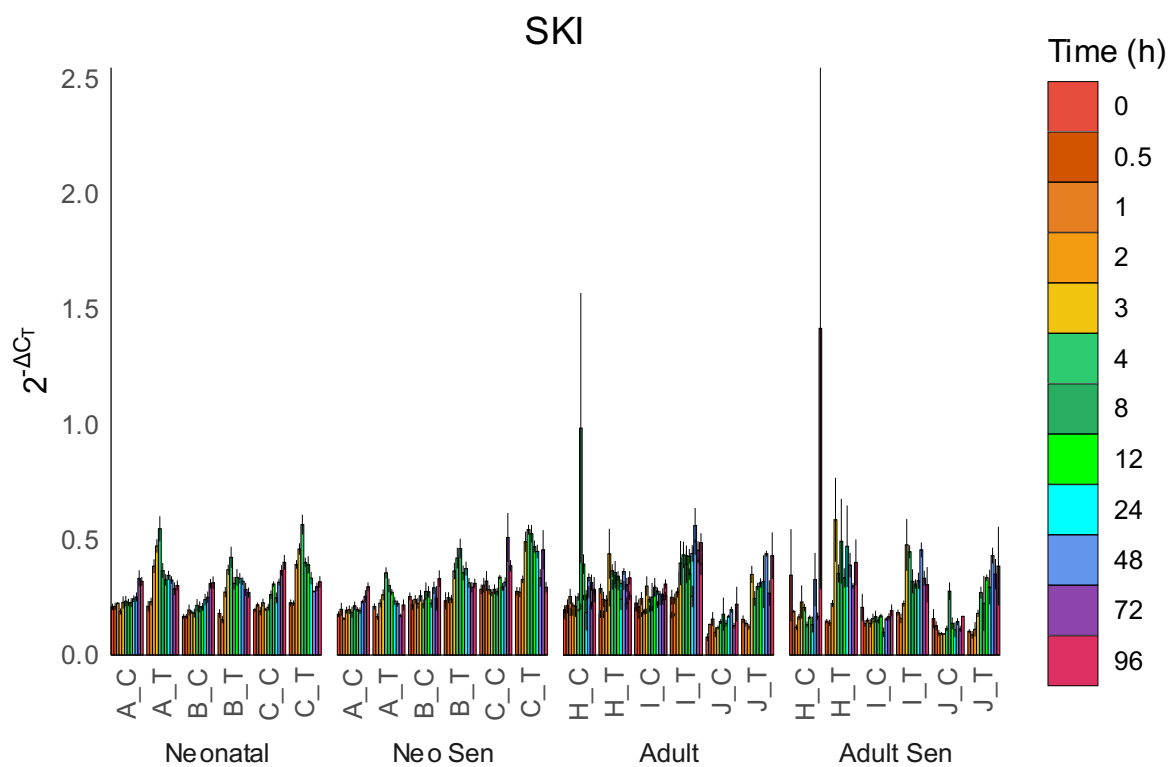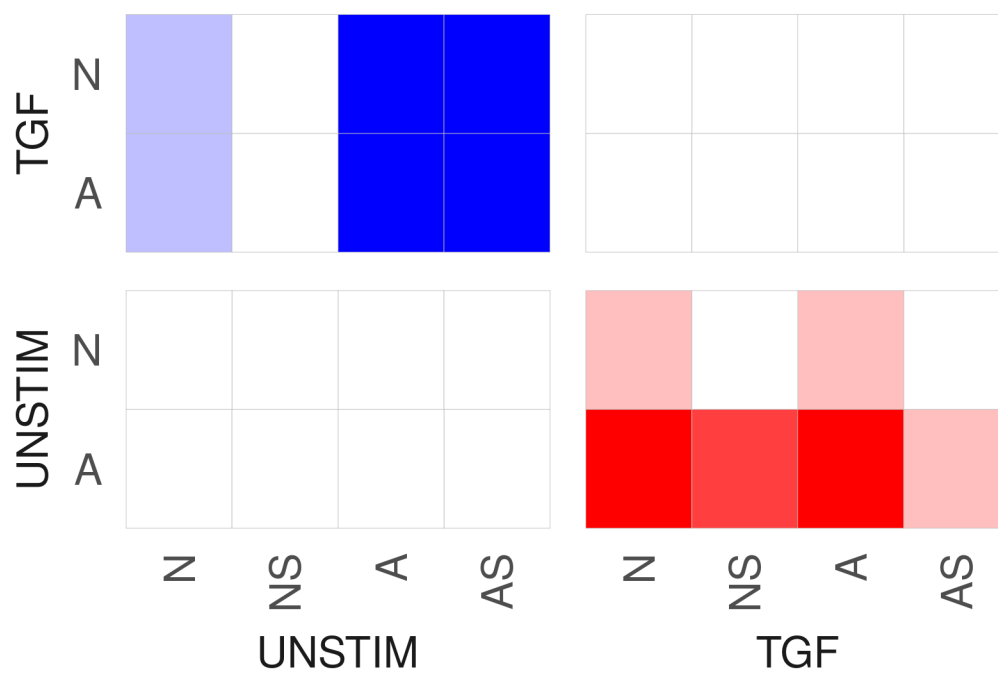

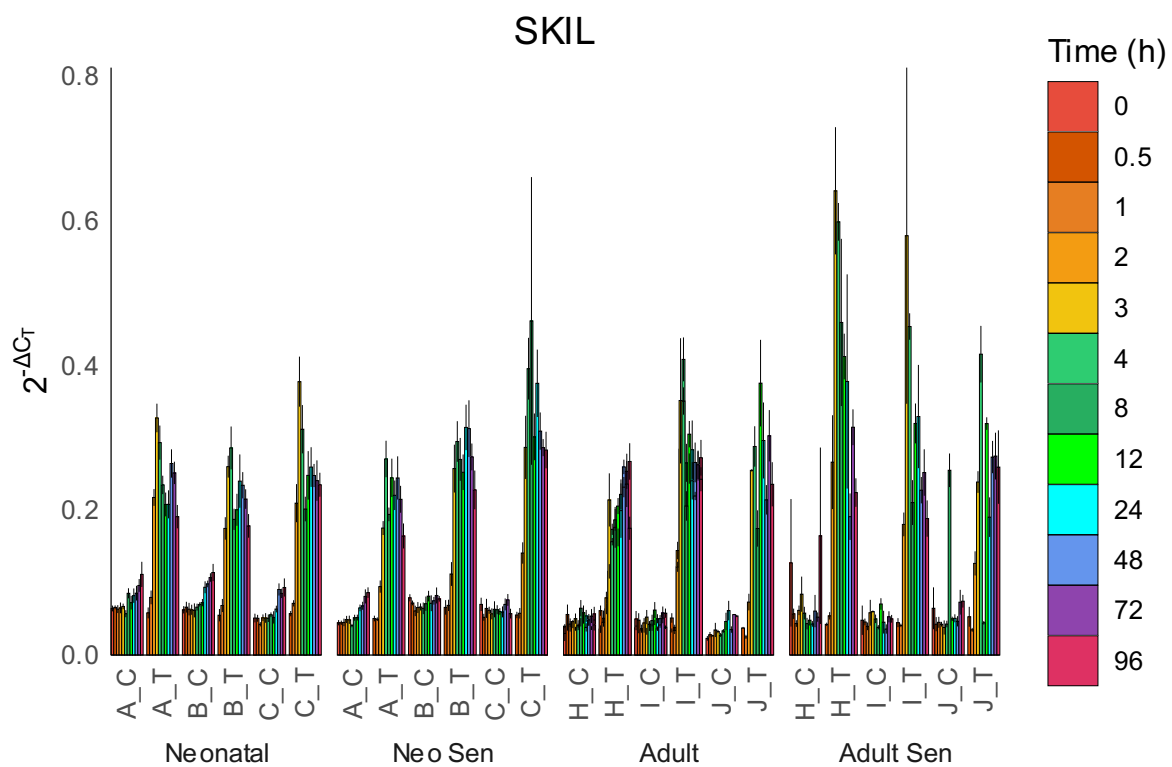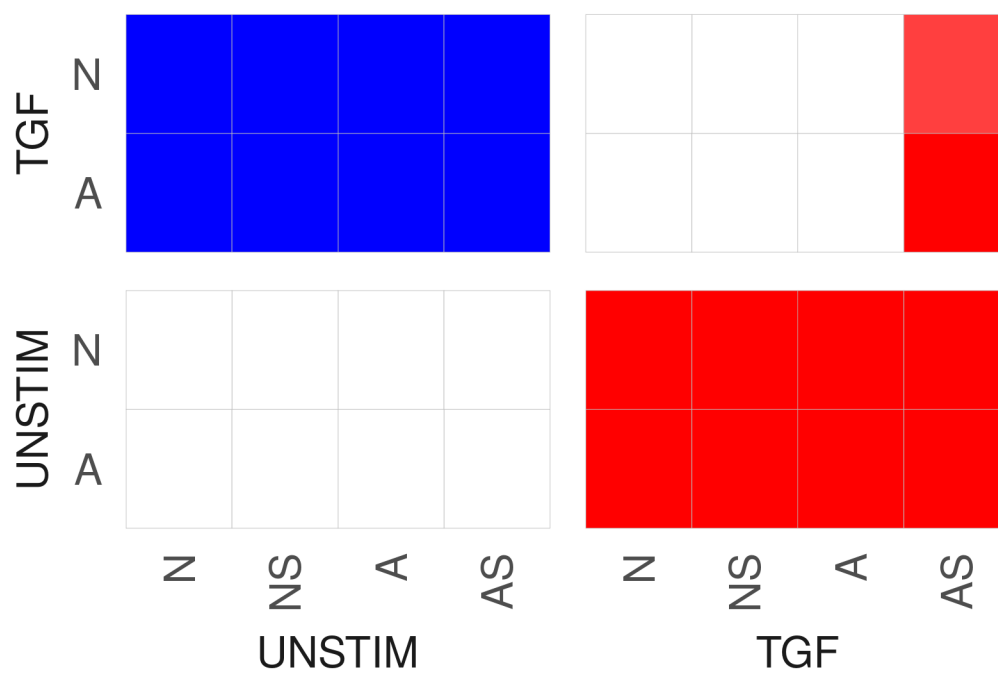

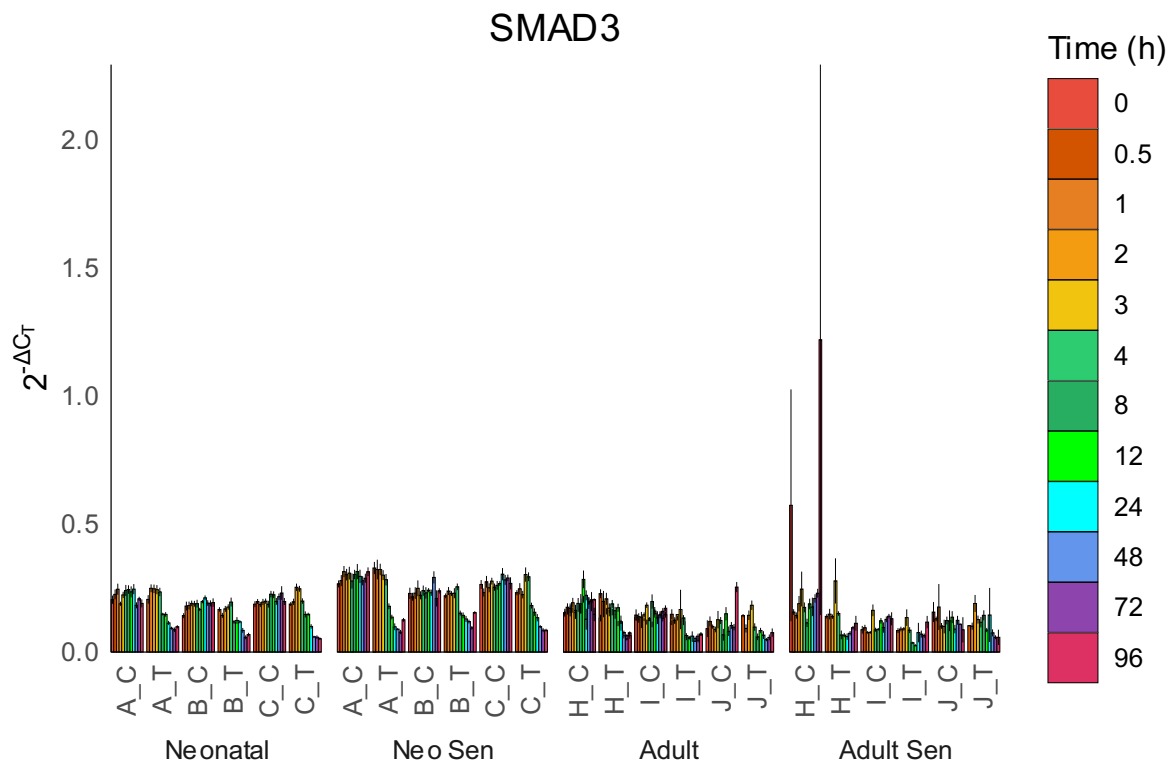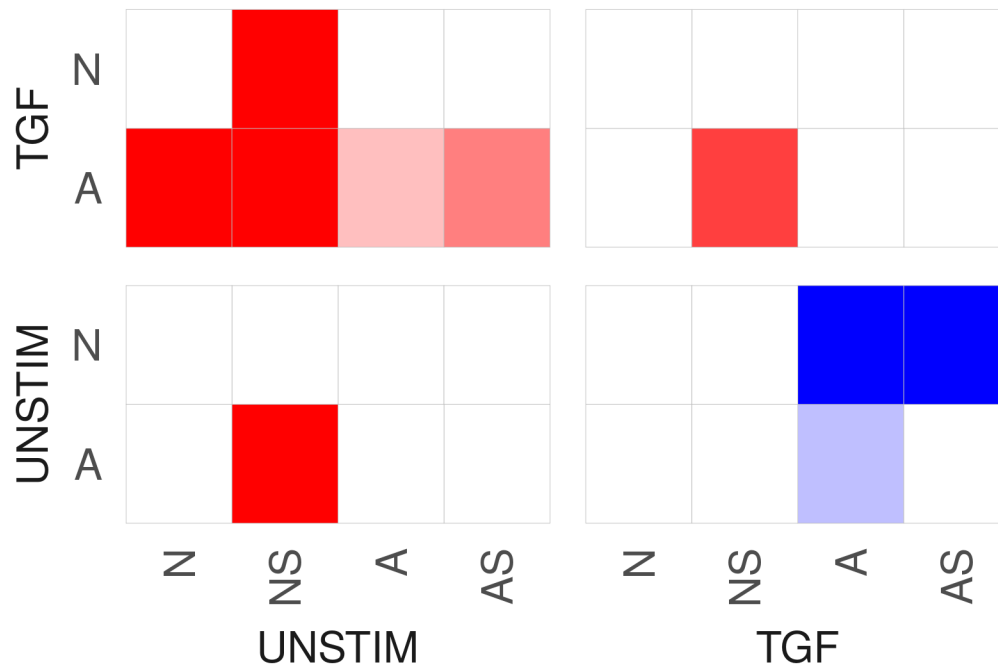

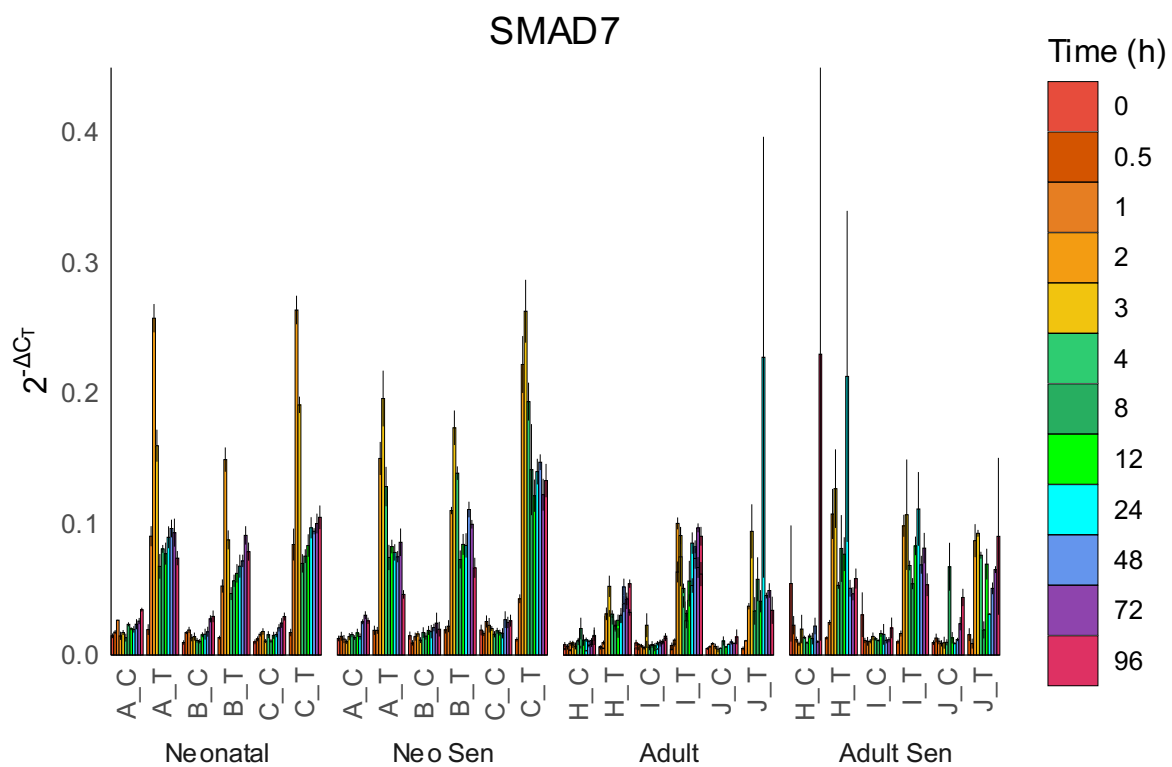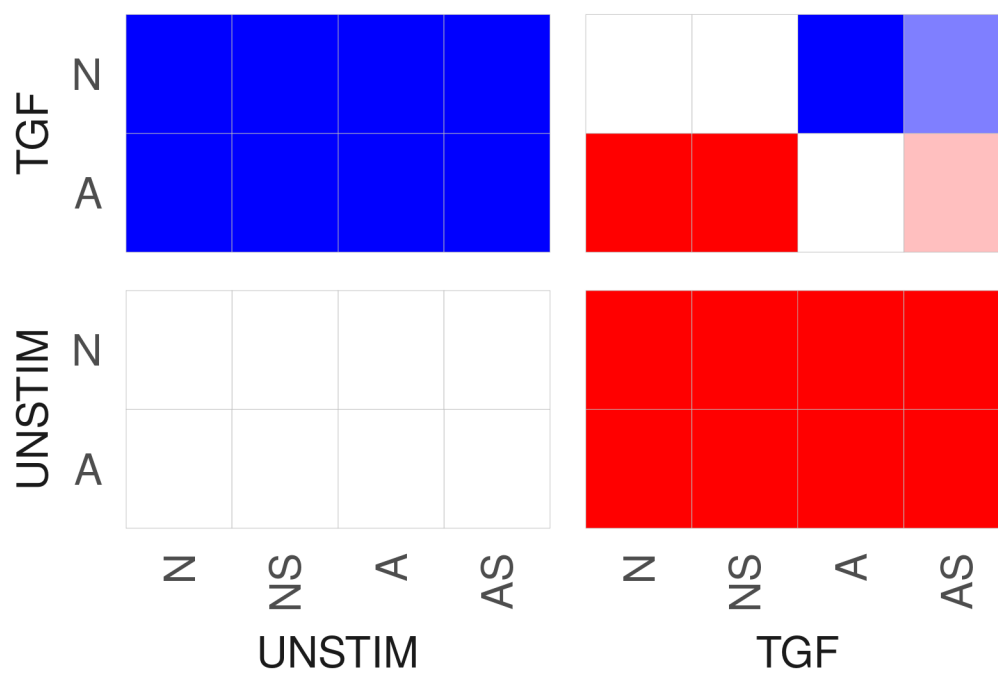

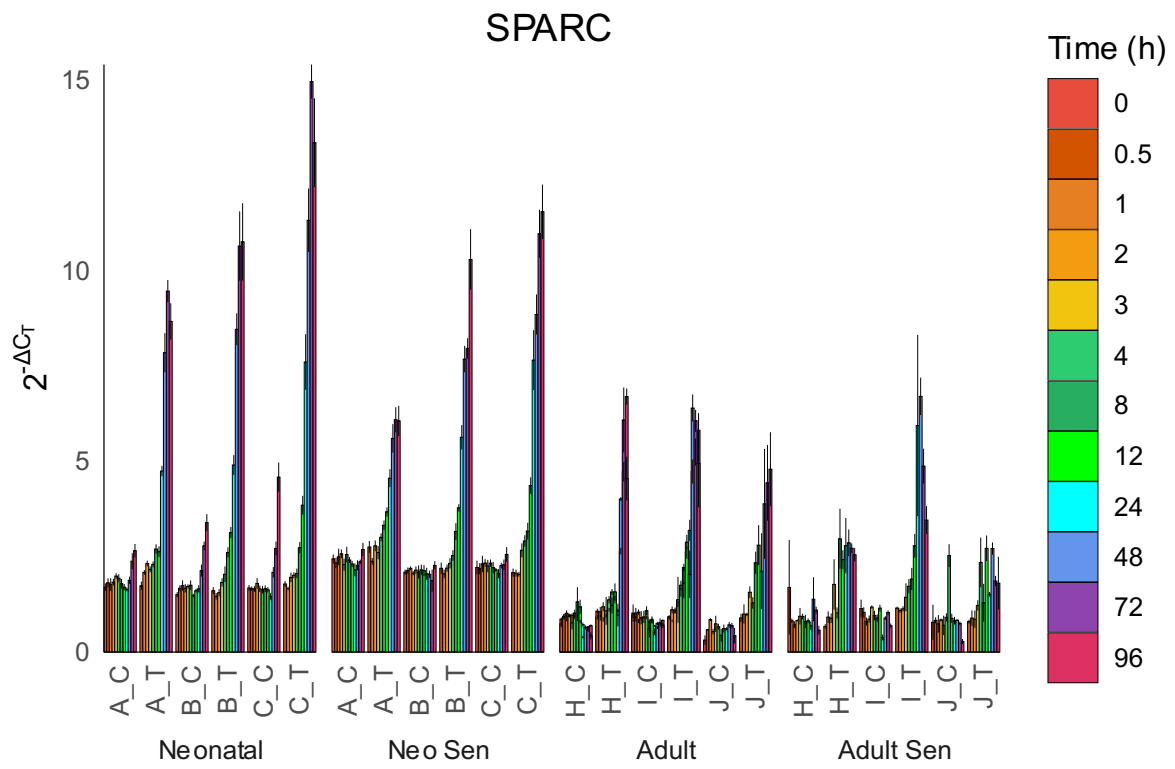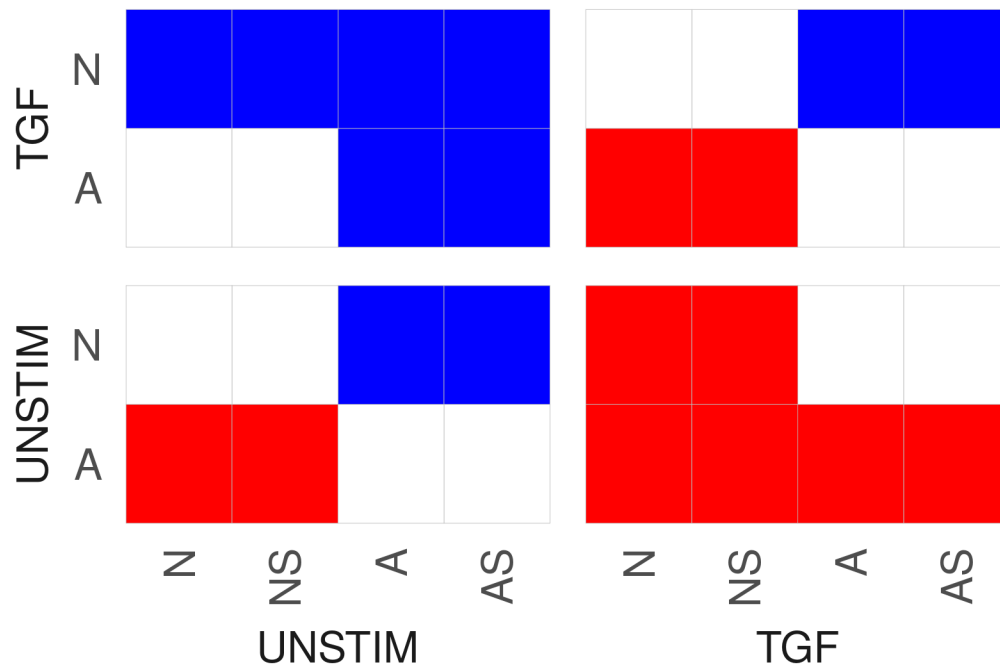

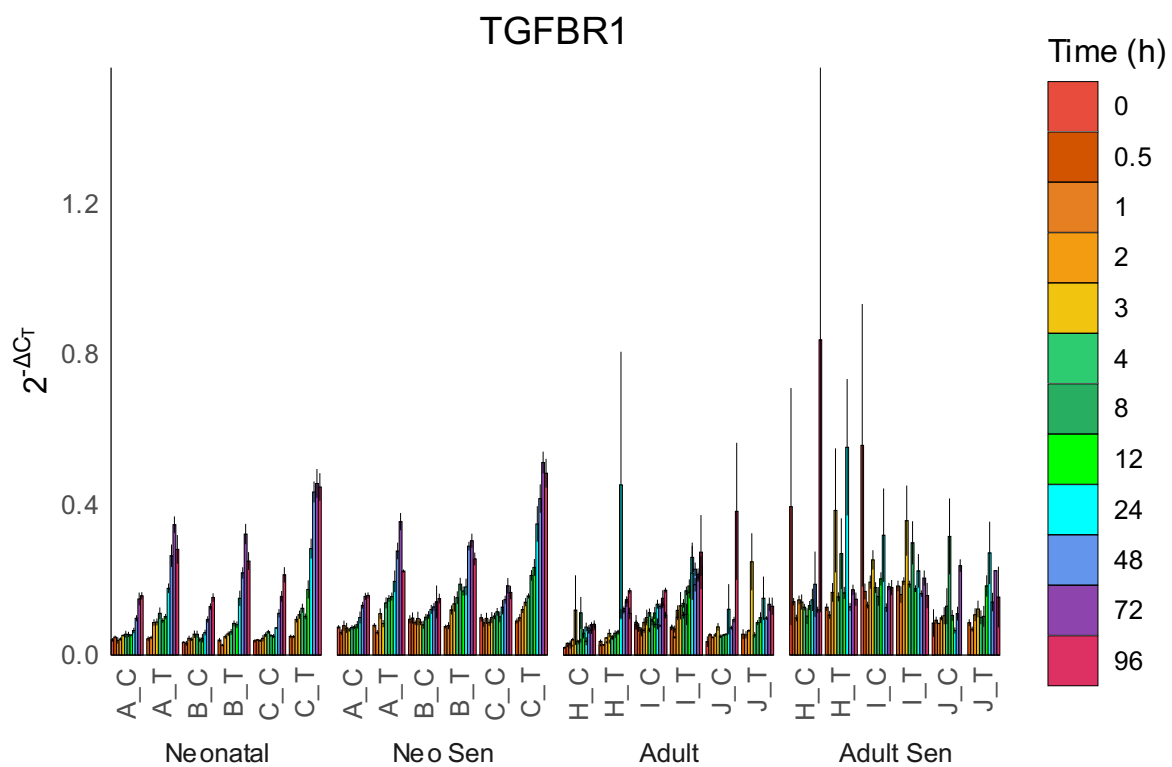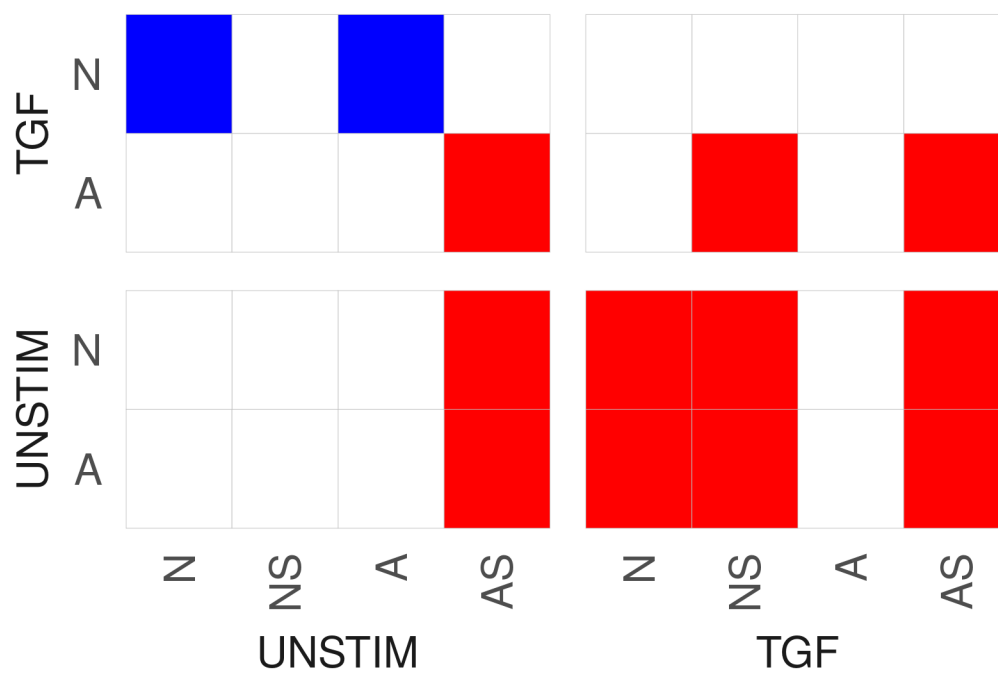

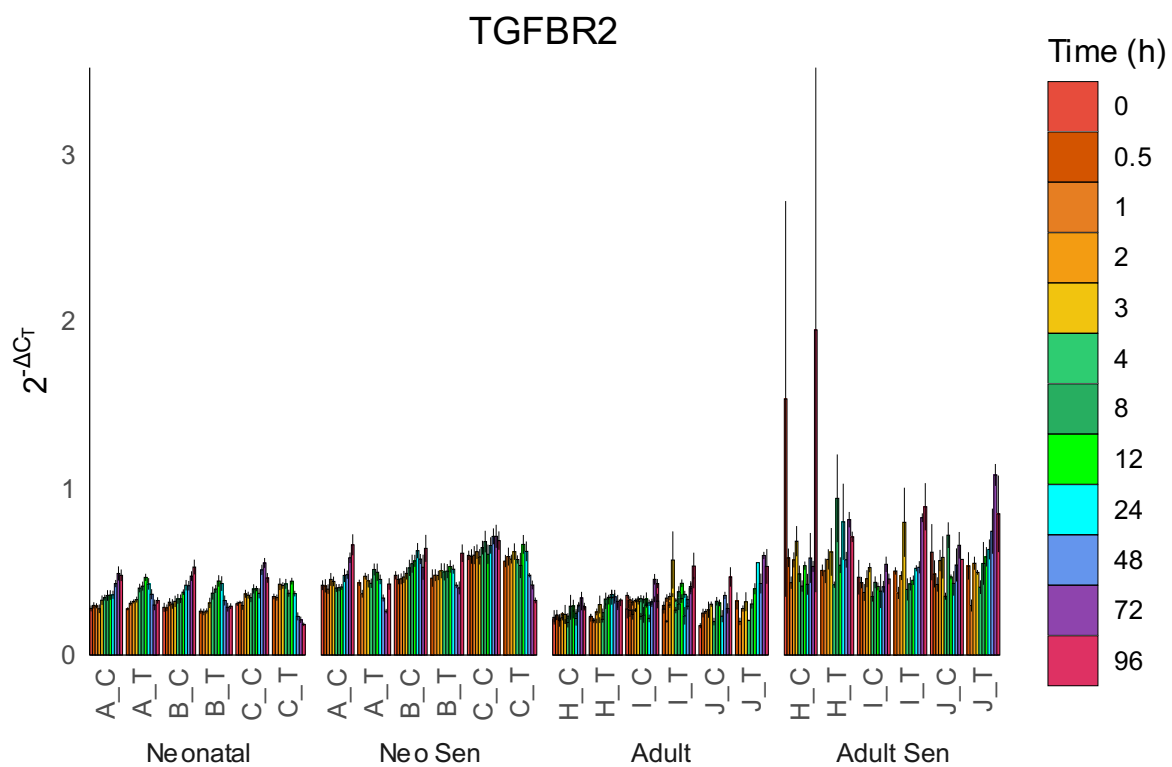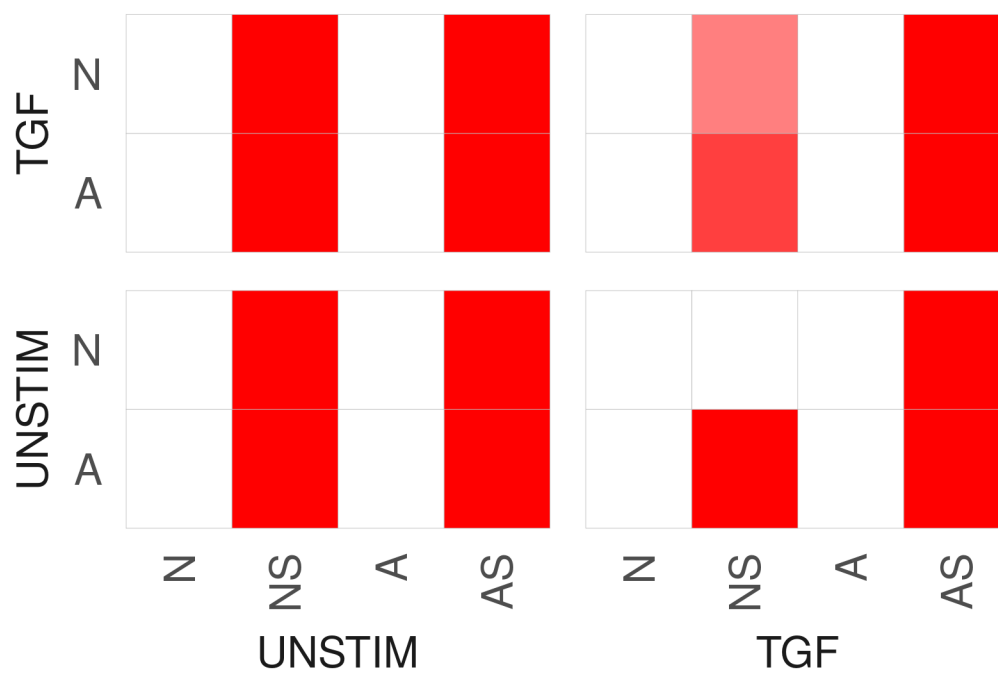

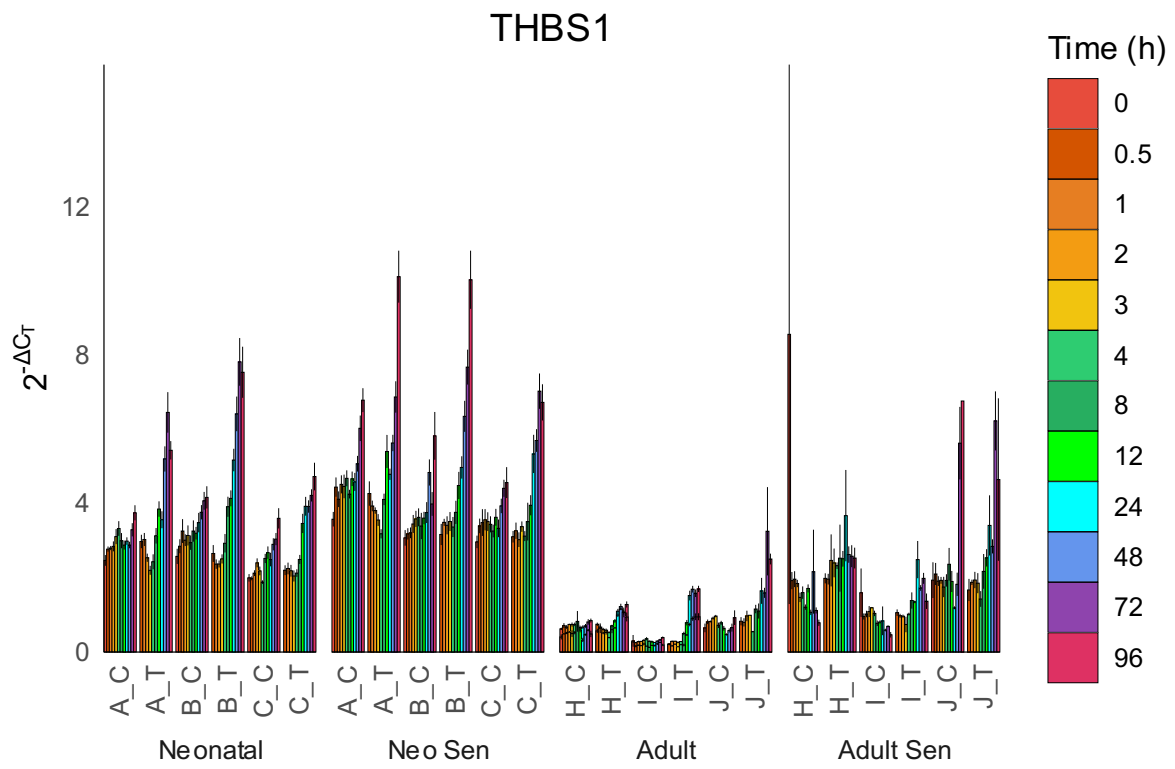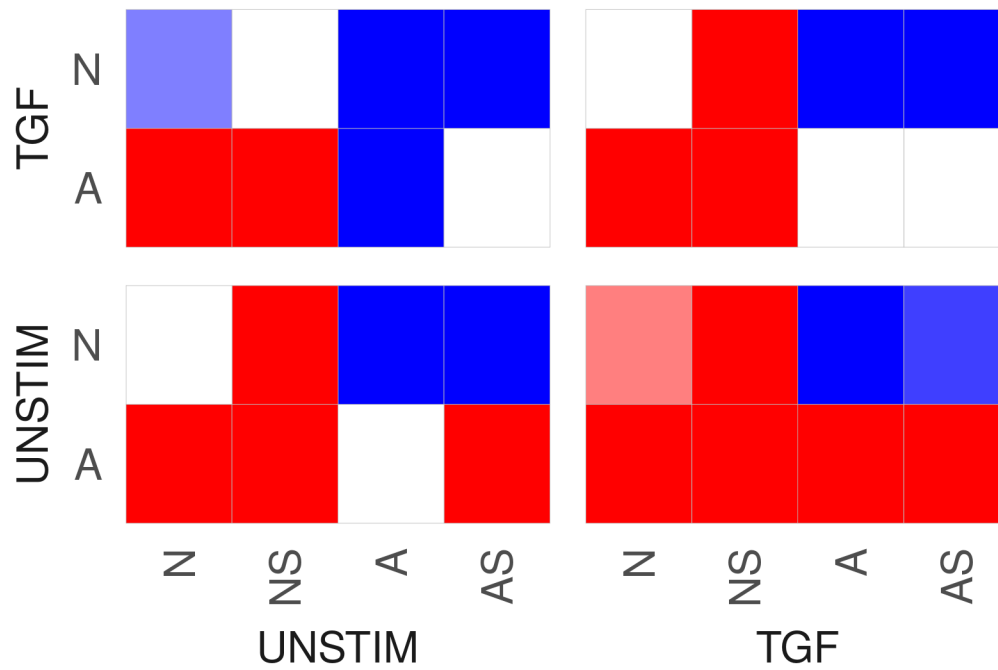

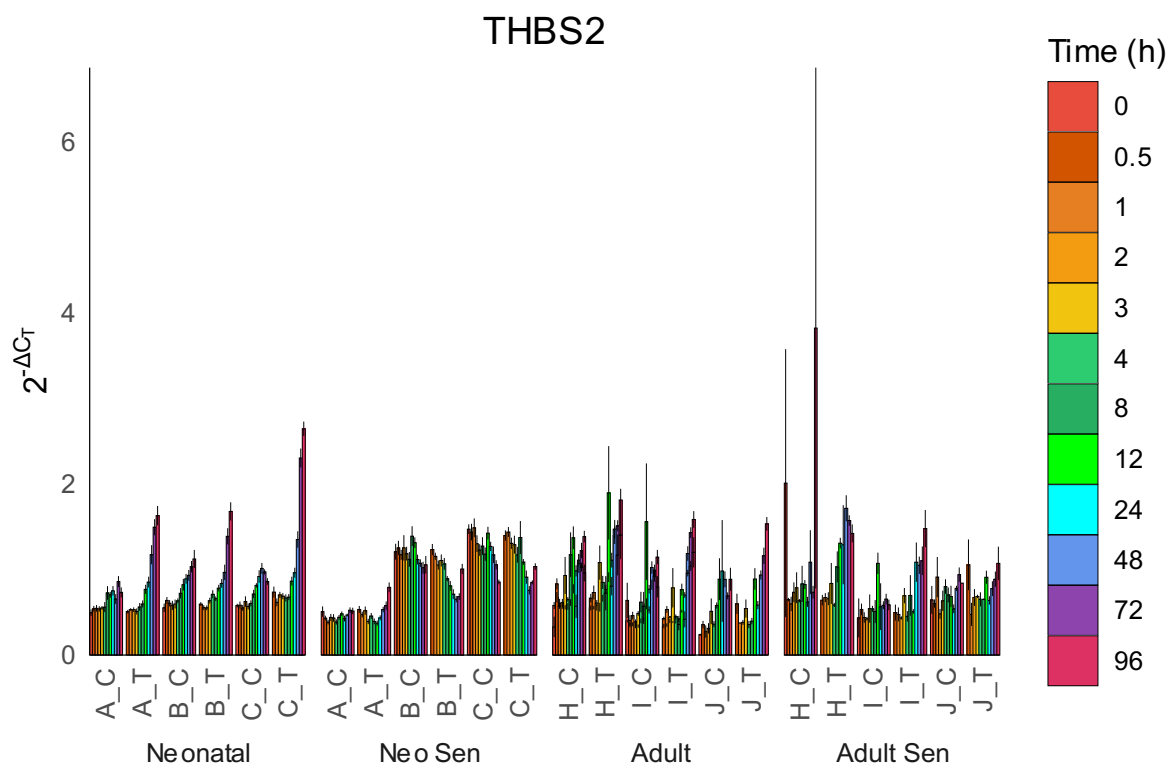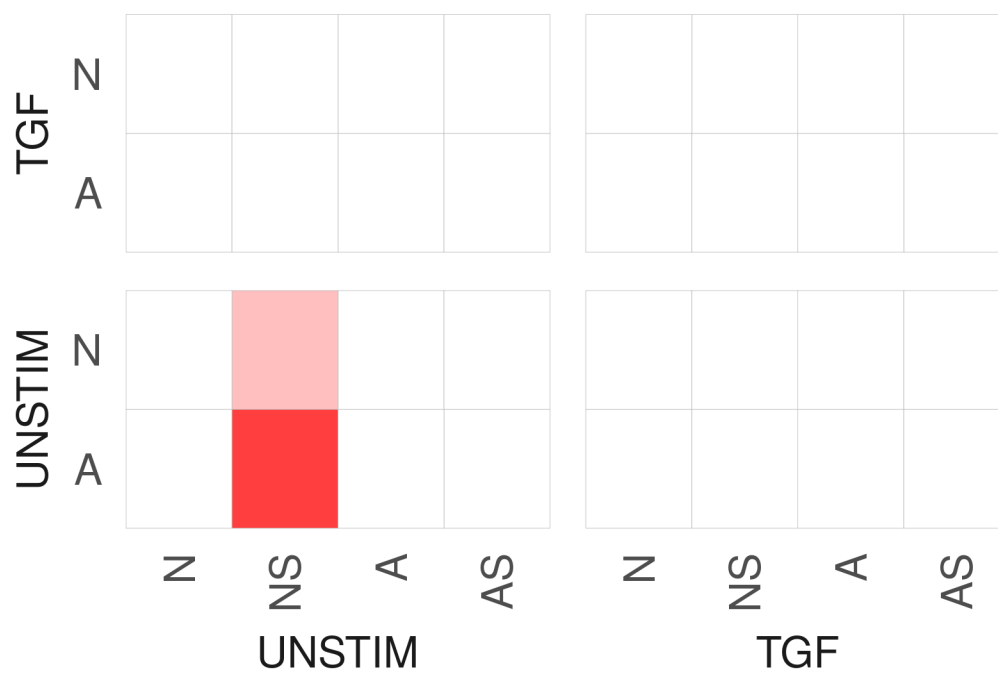

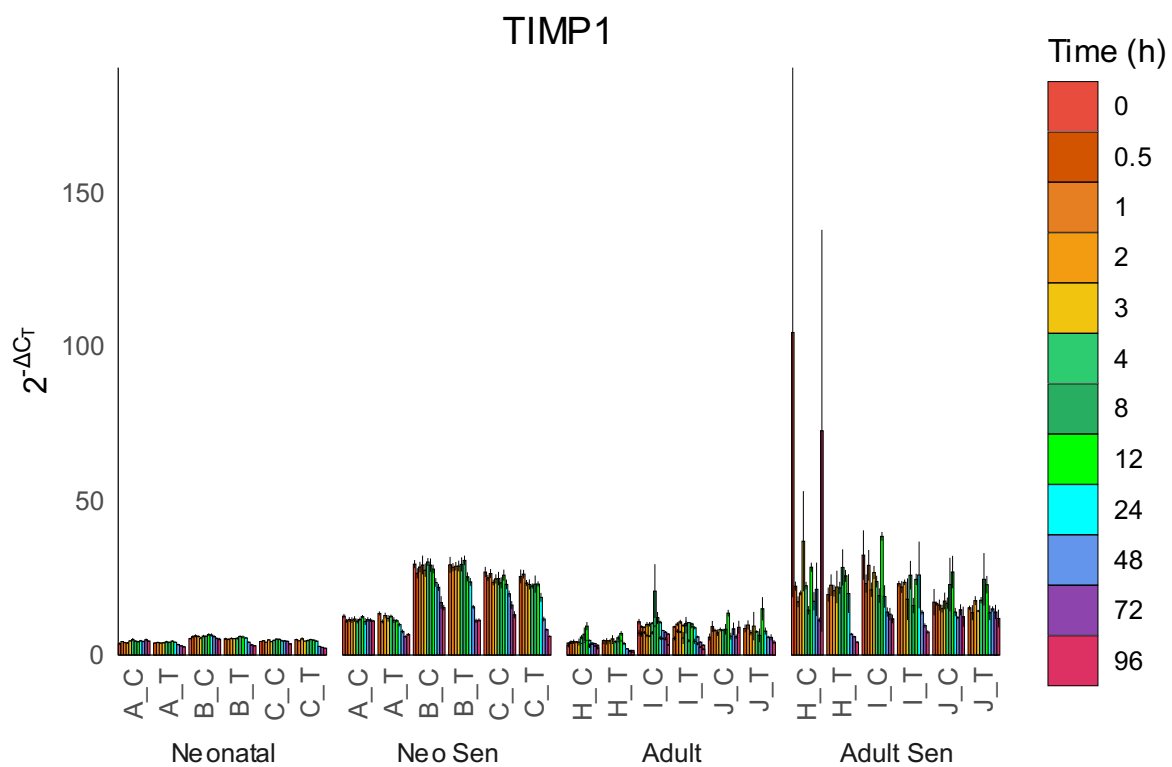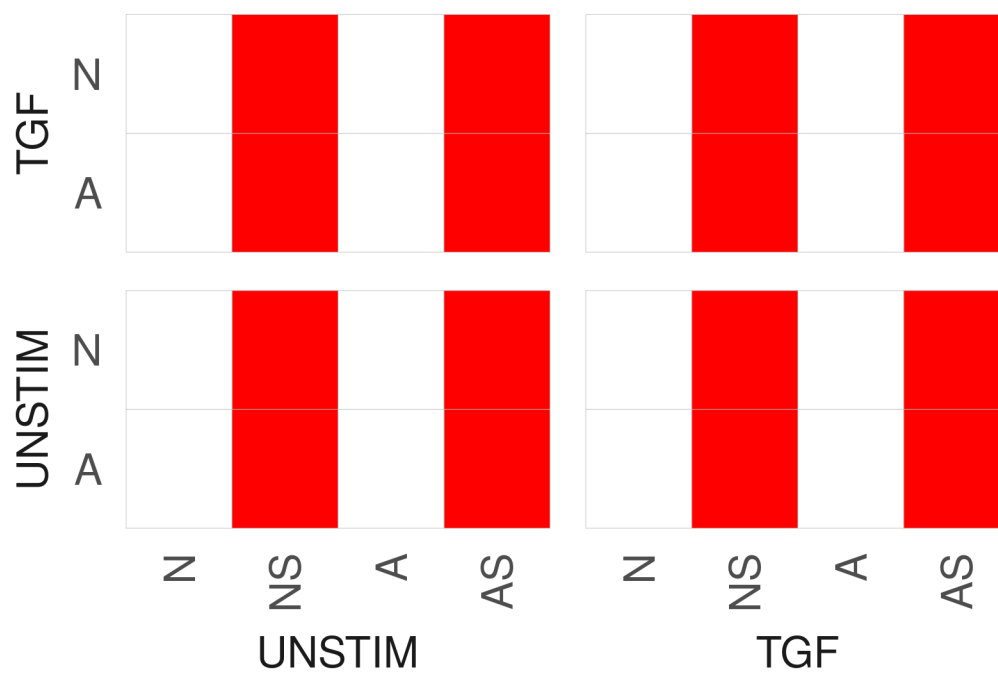

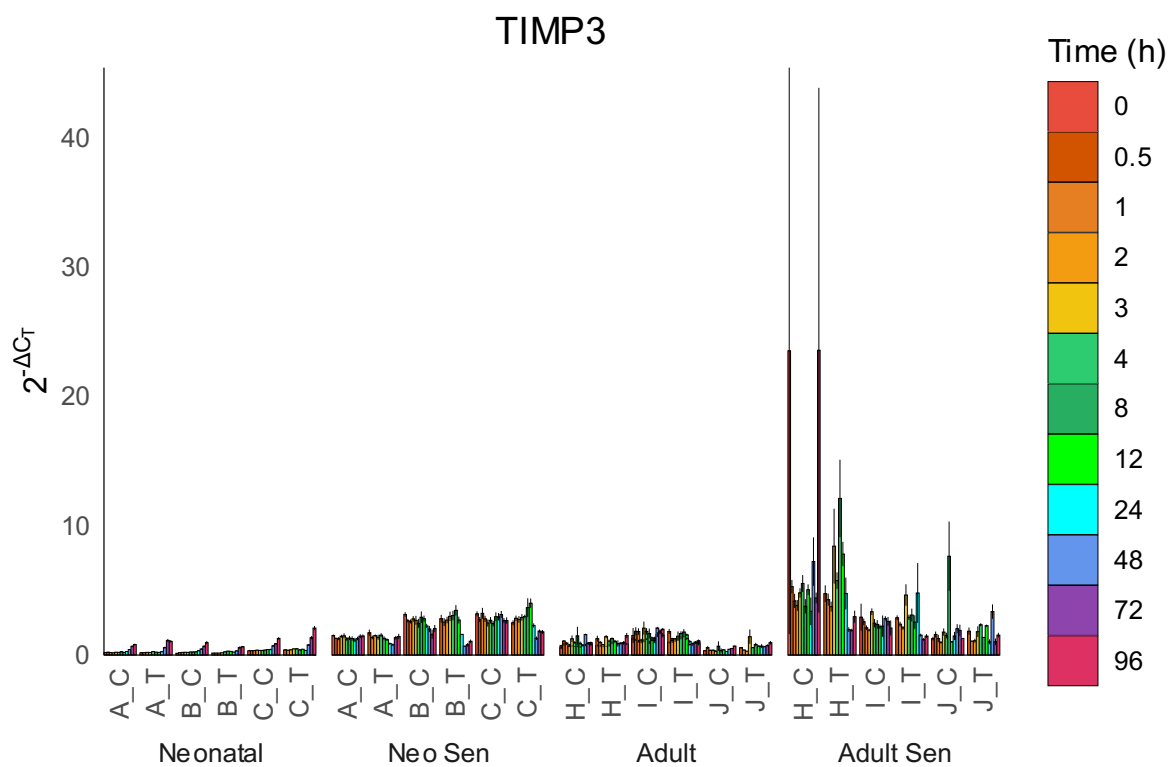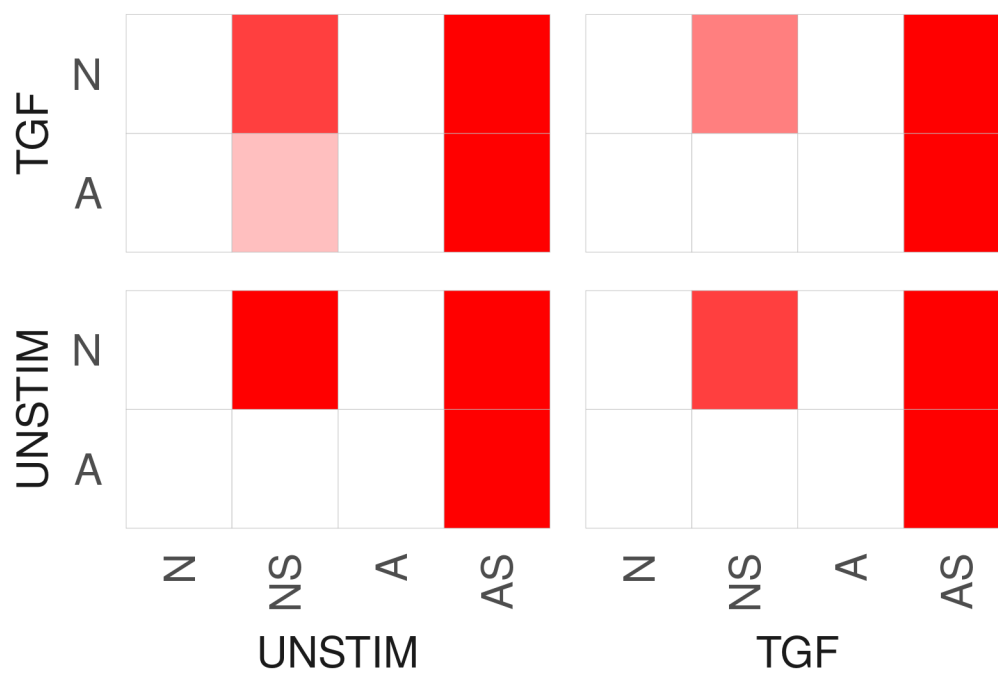

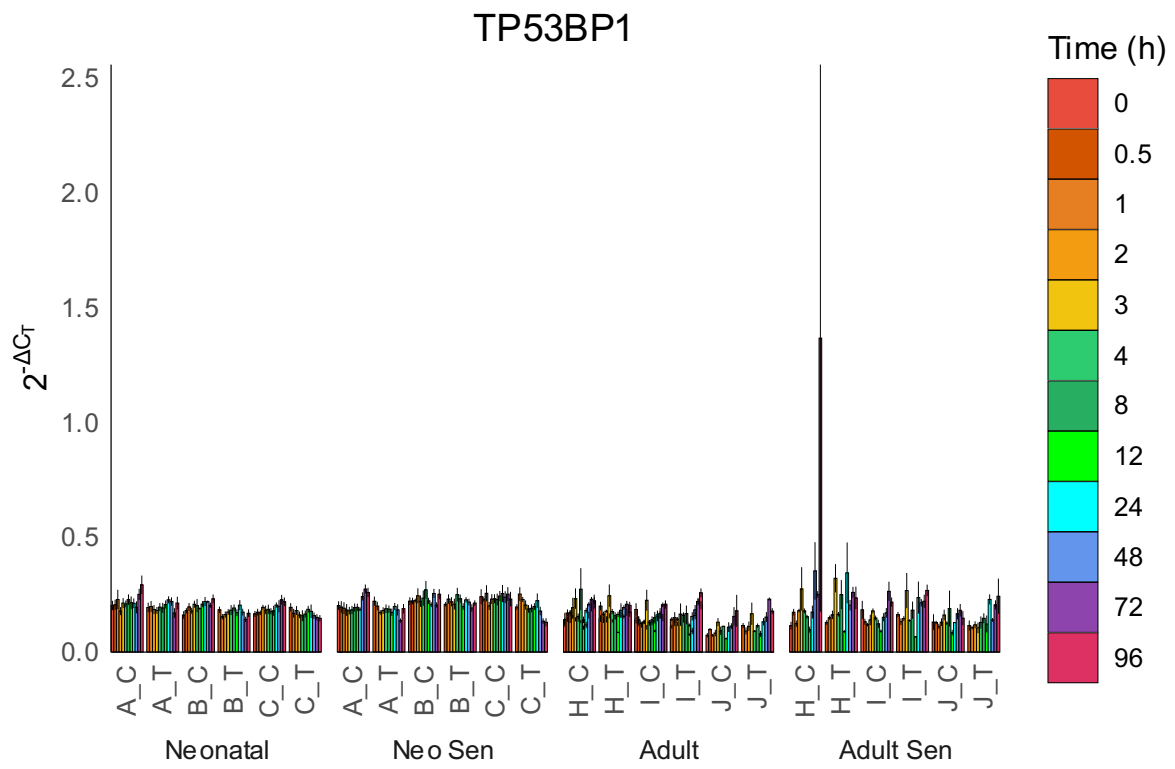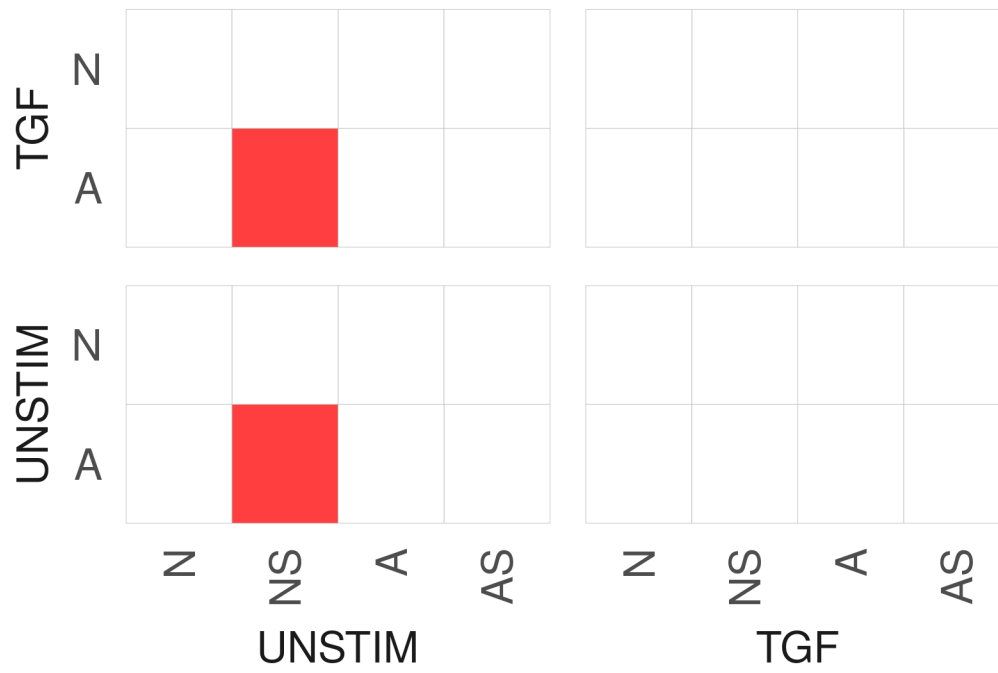

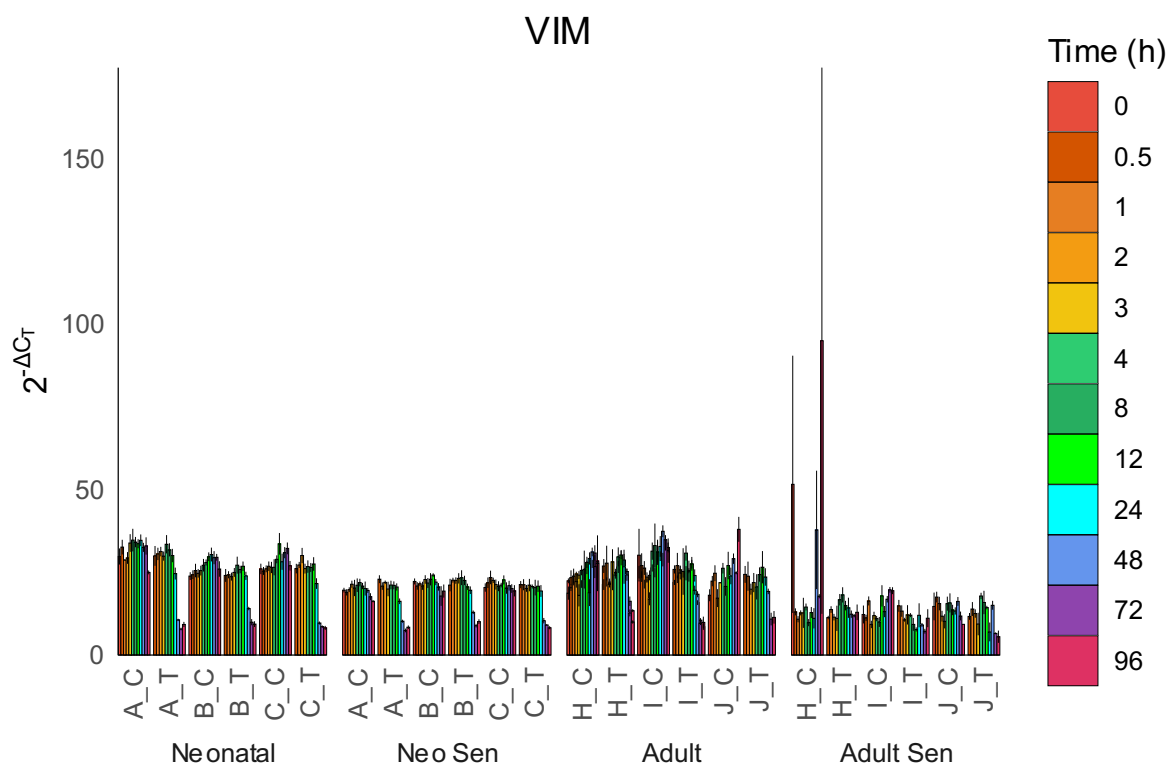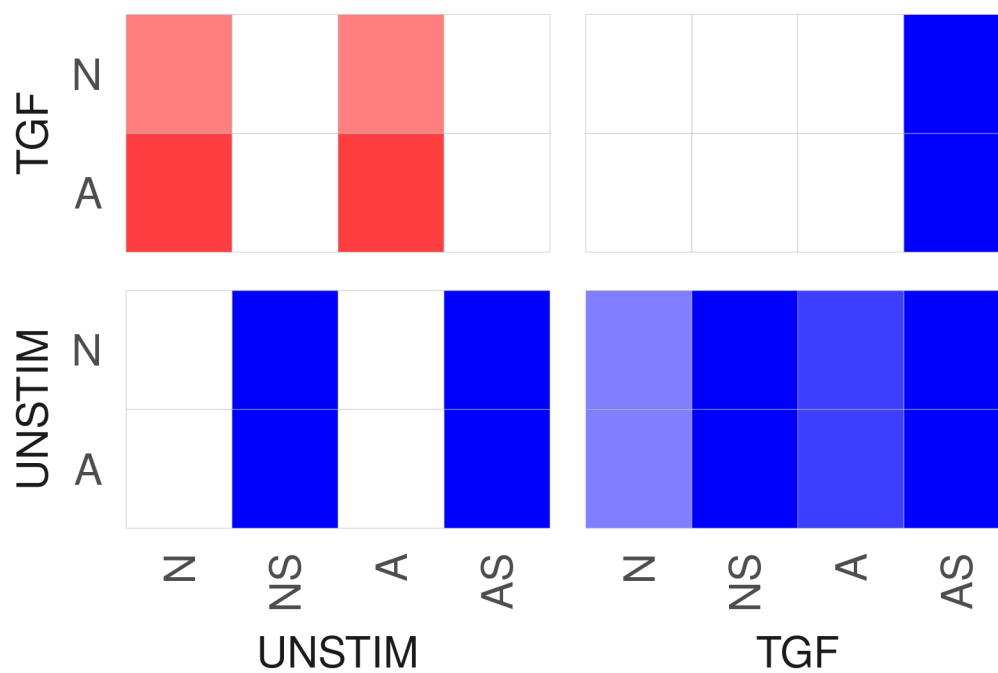

Supplement: Supplementary file 1 [file cells-13-00659-s001.zip › Supplementary file 4.pdf]

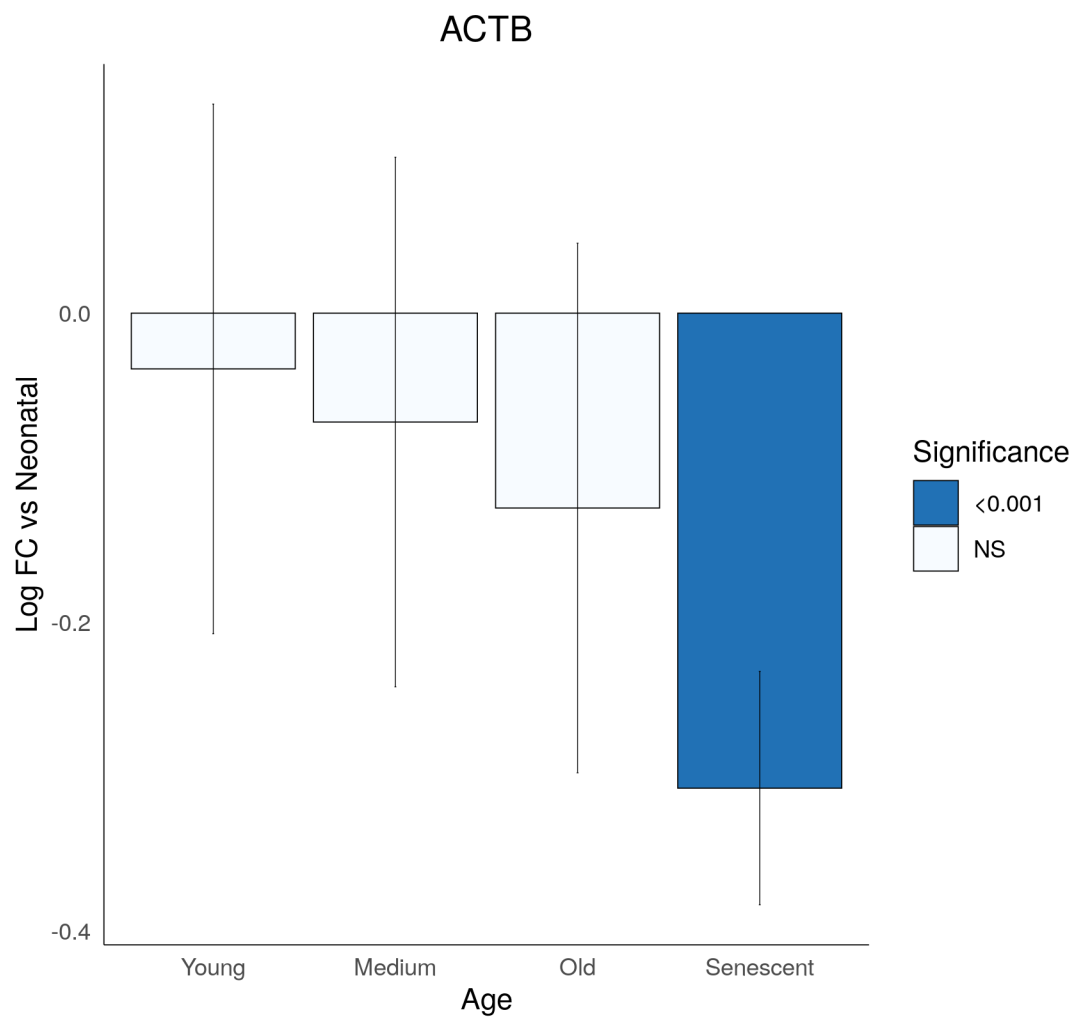

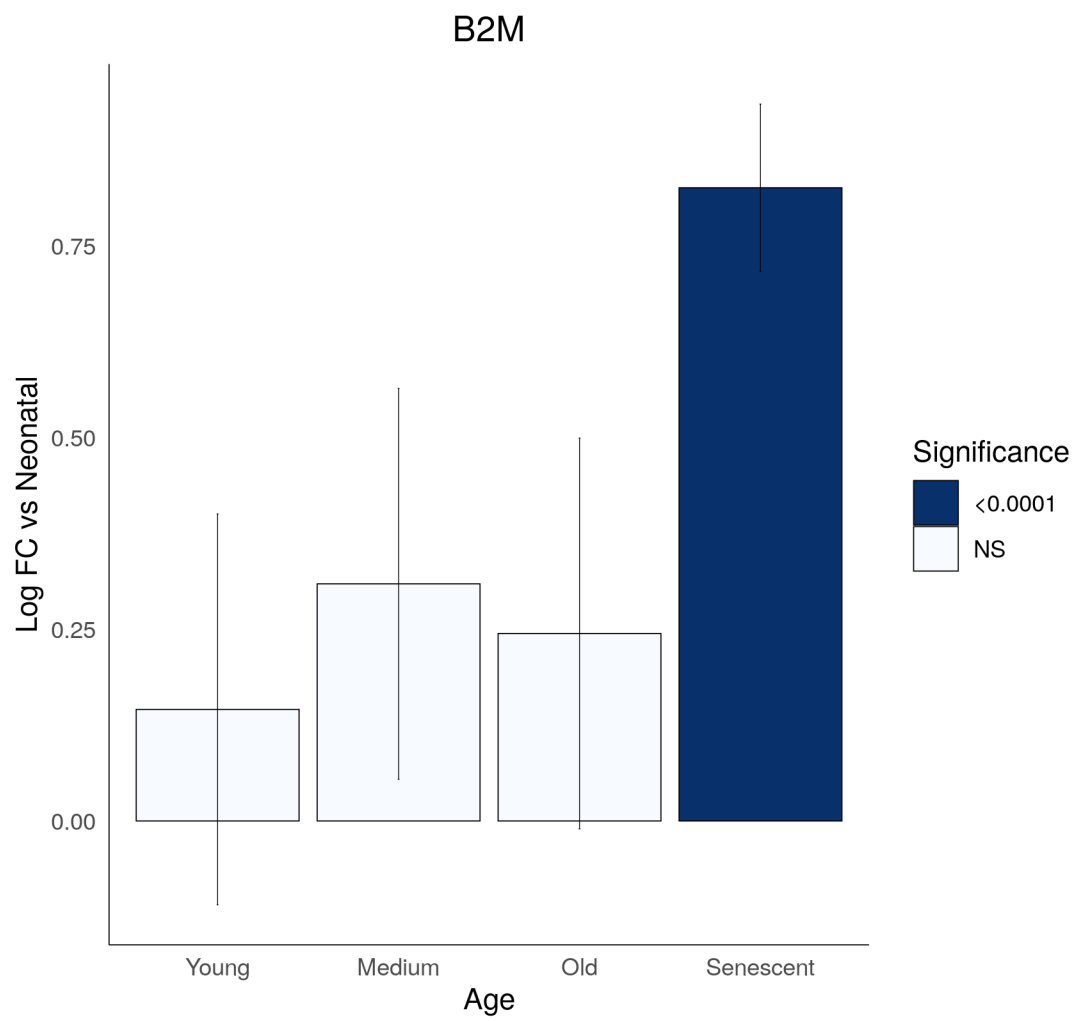

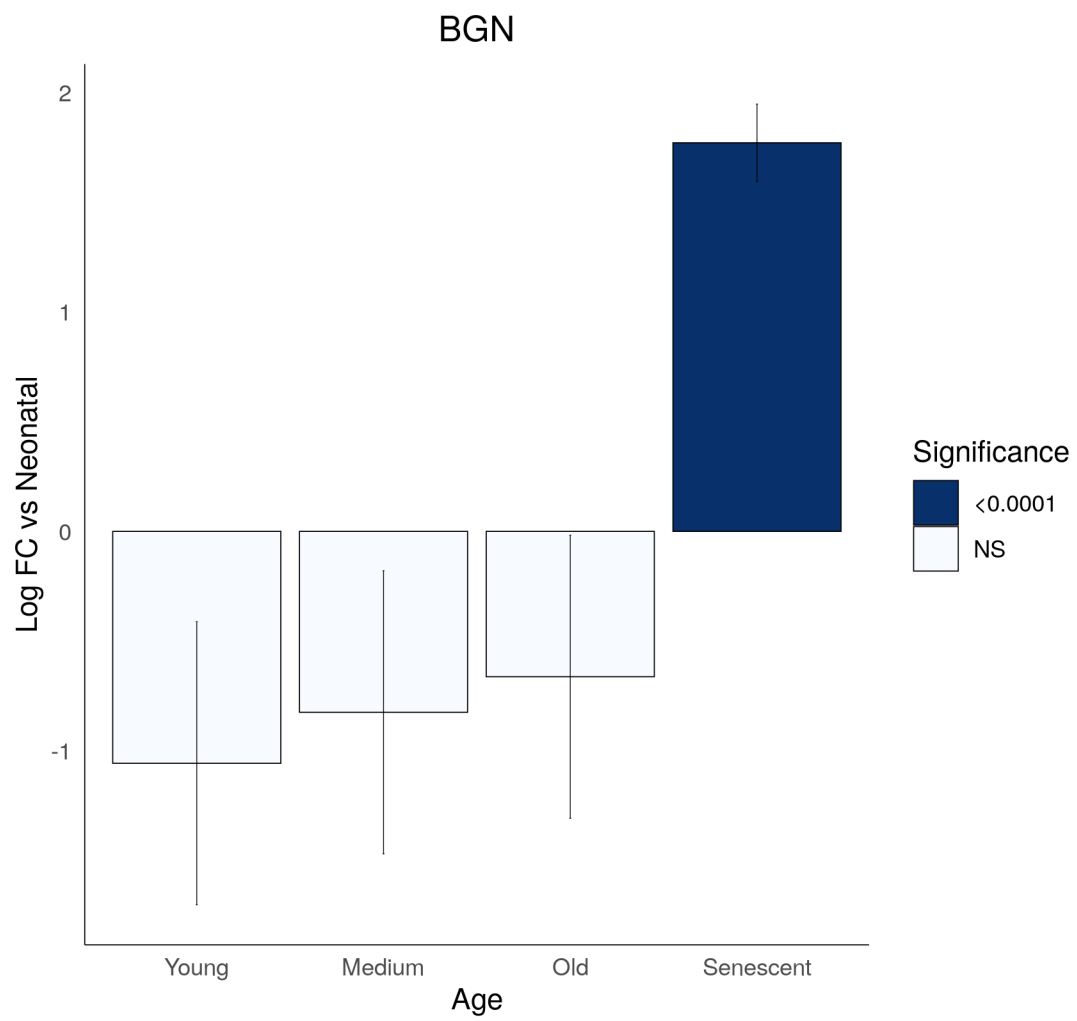

COL1A1

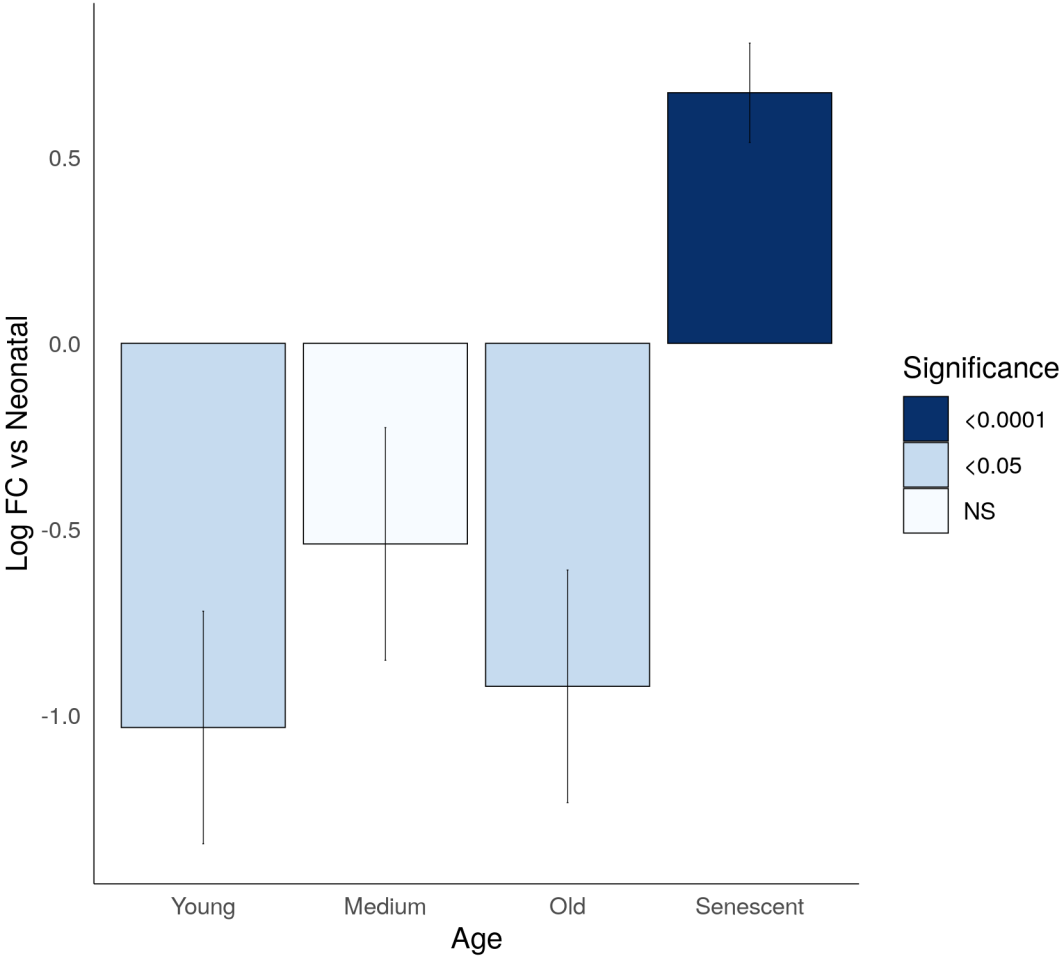

COL1A2

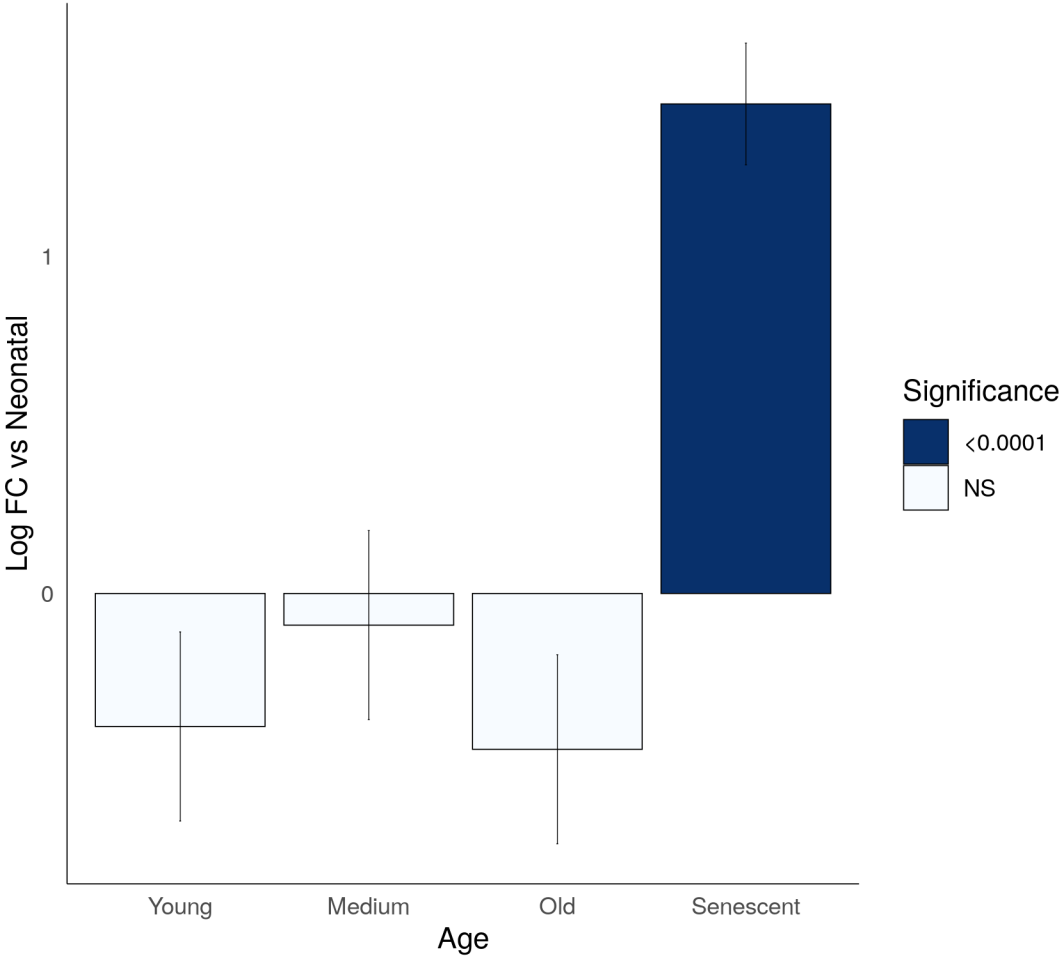

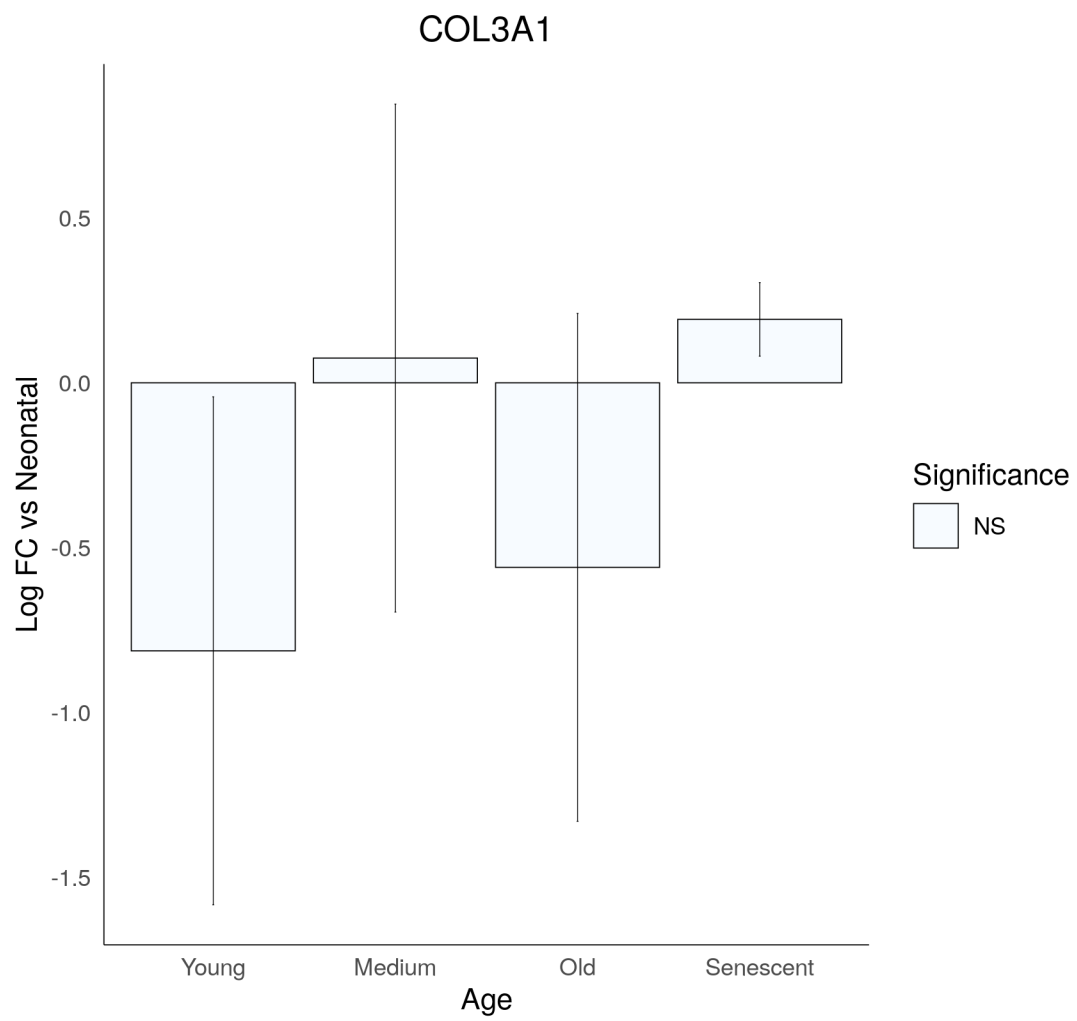

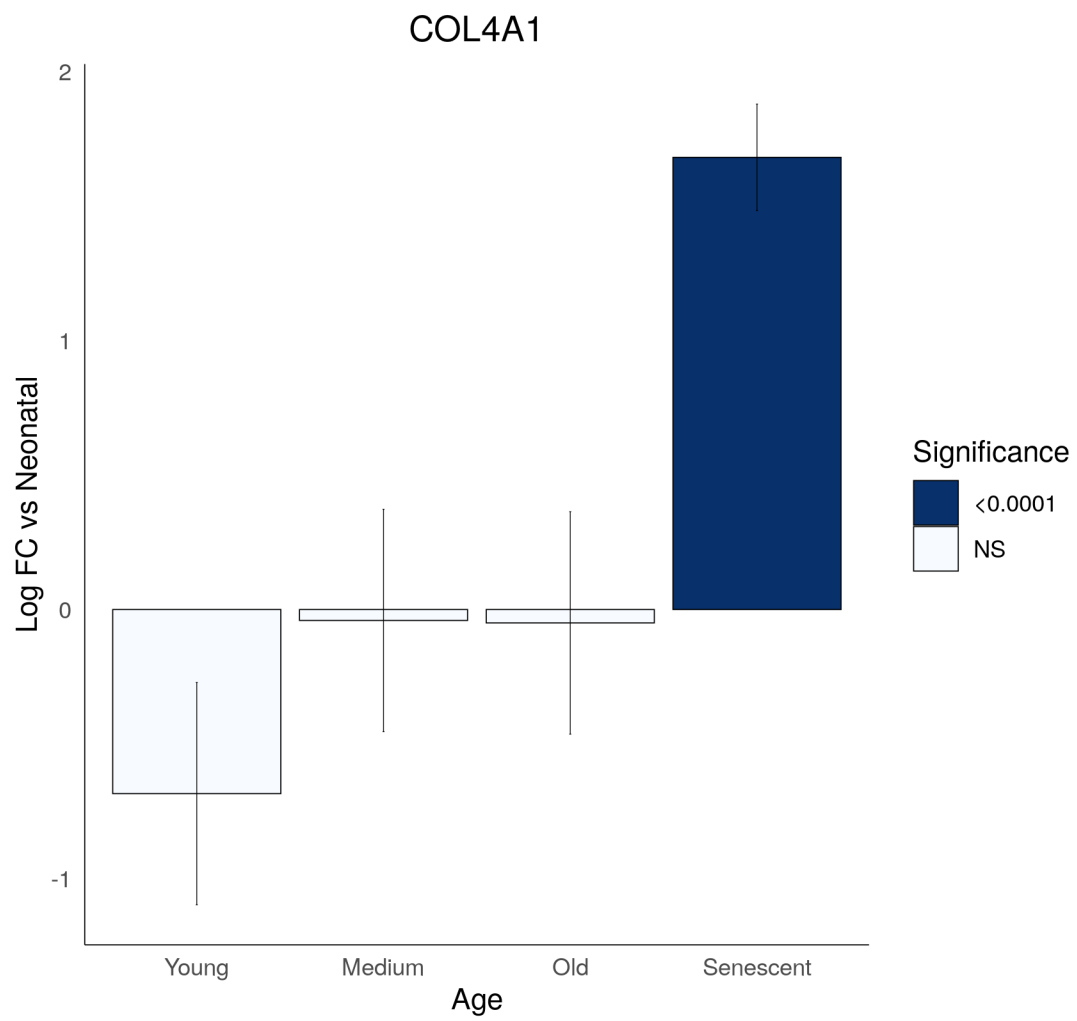

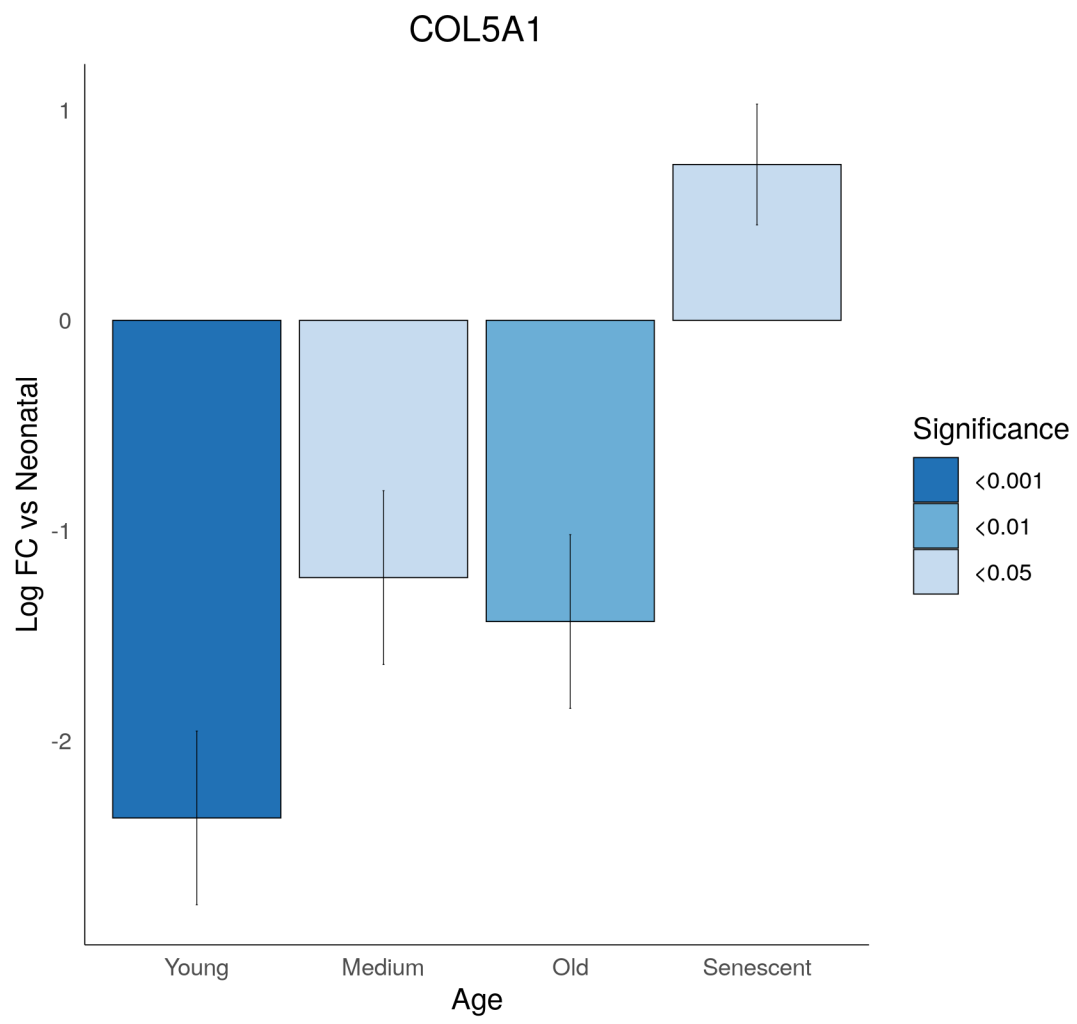

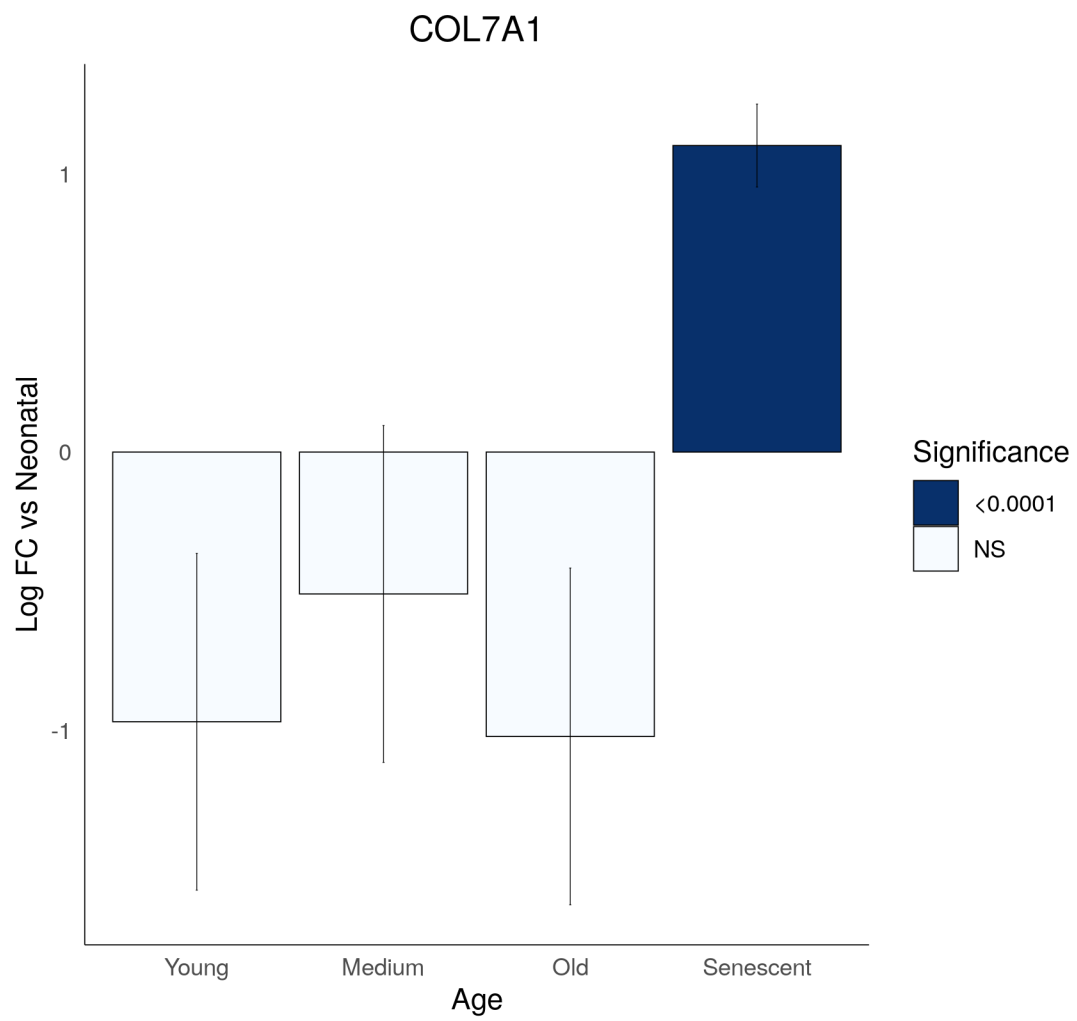

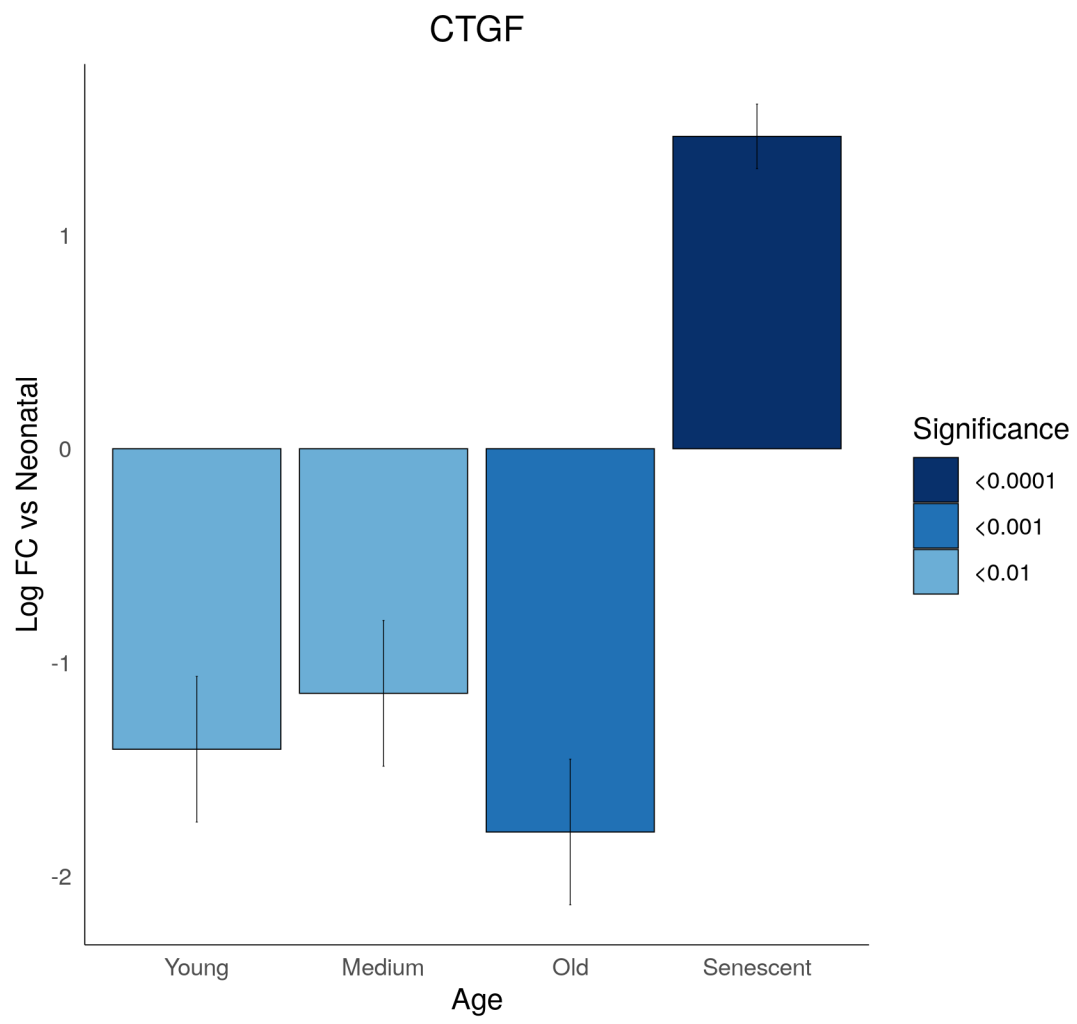

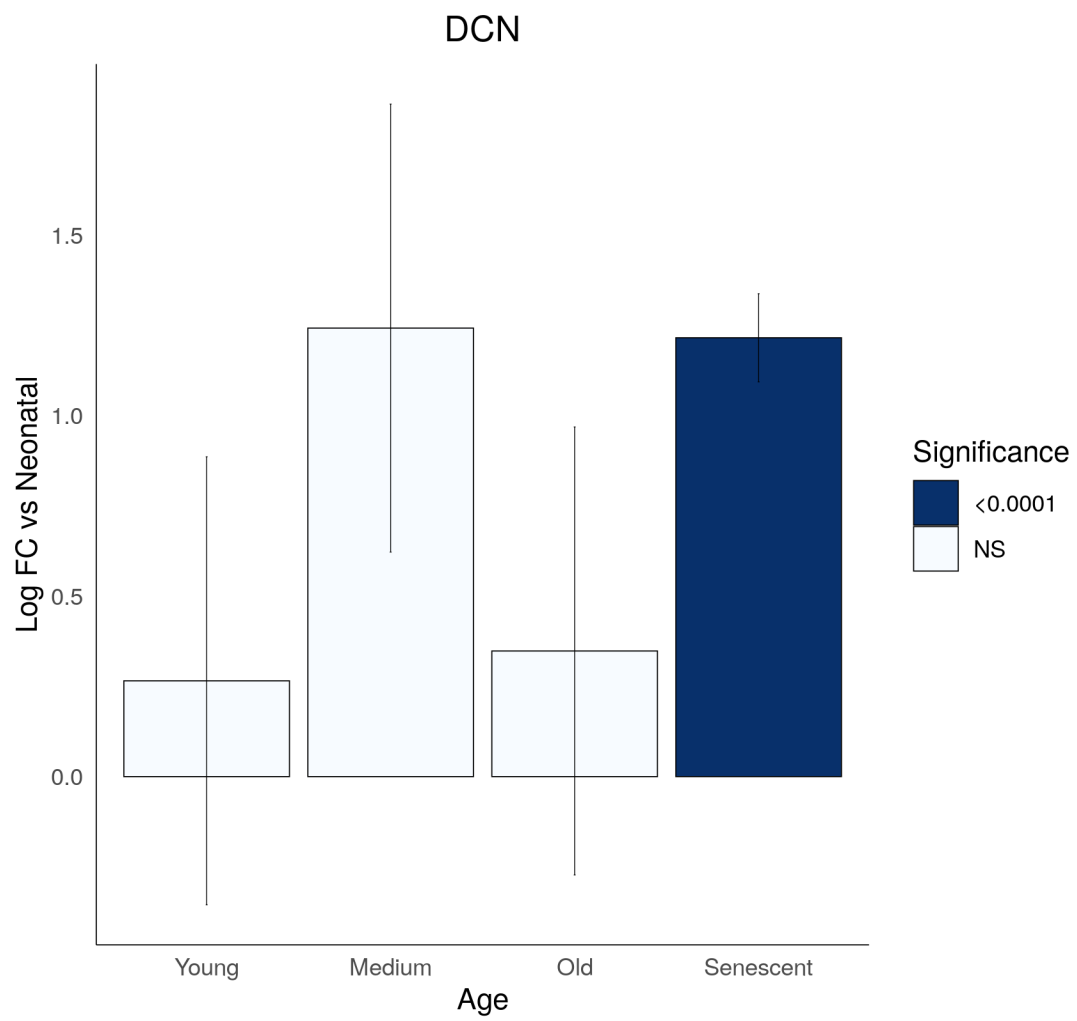

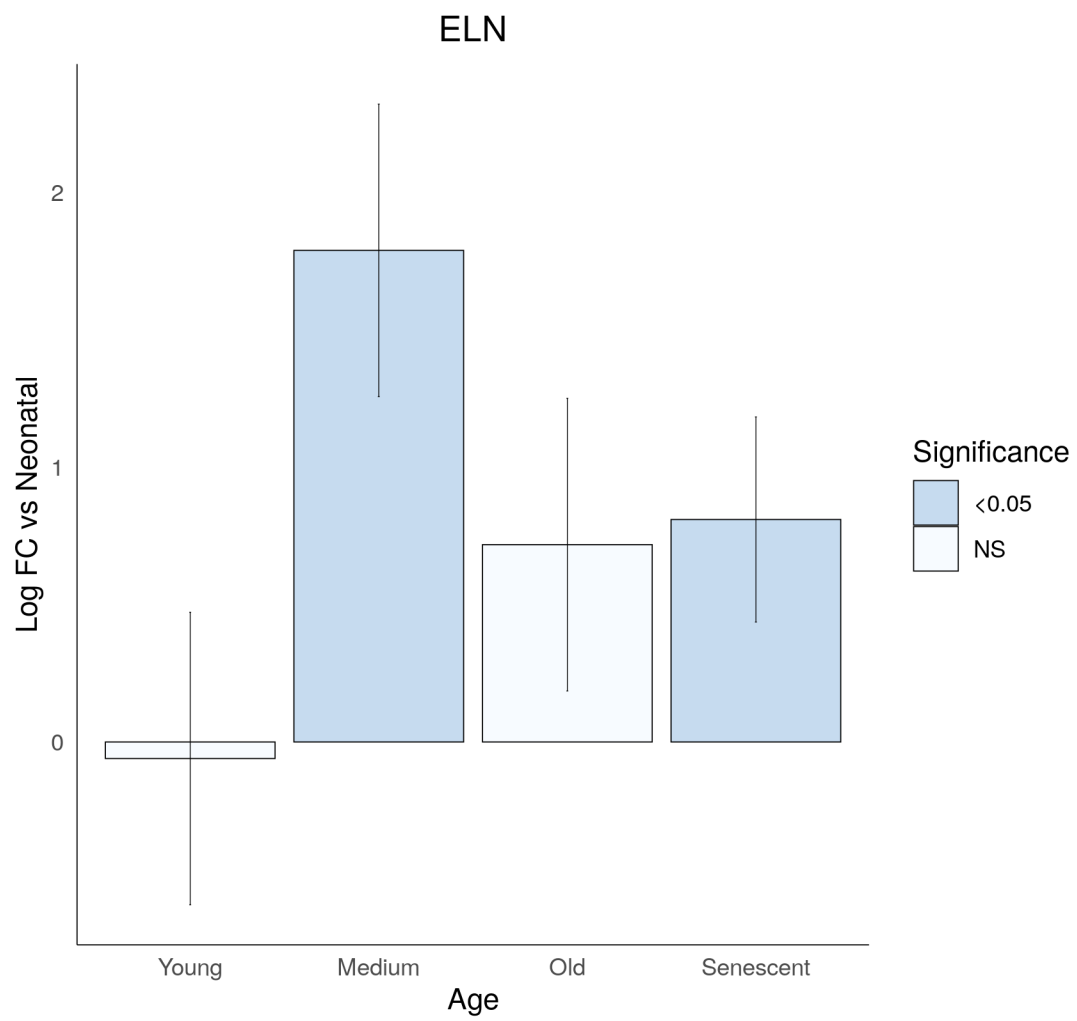

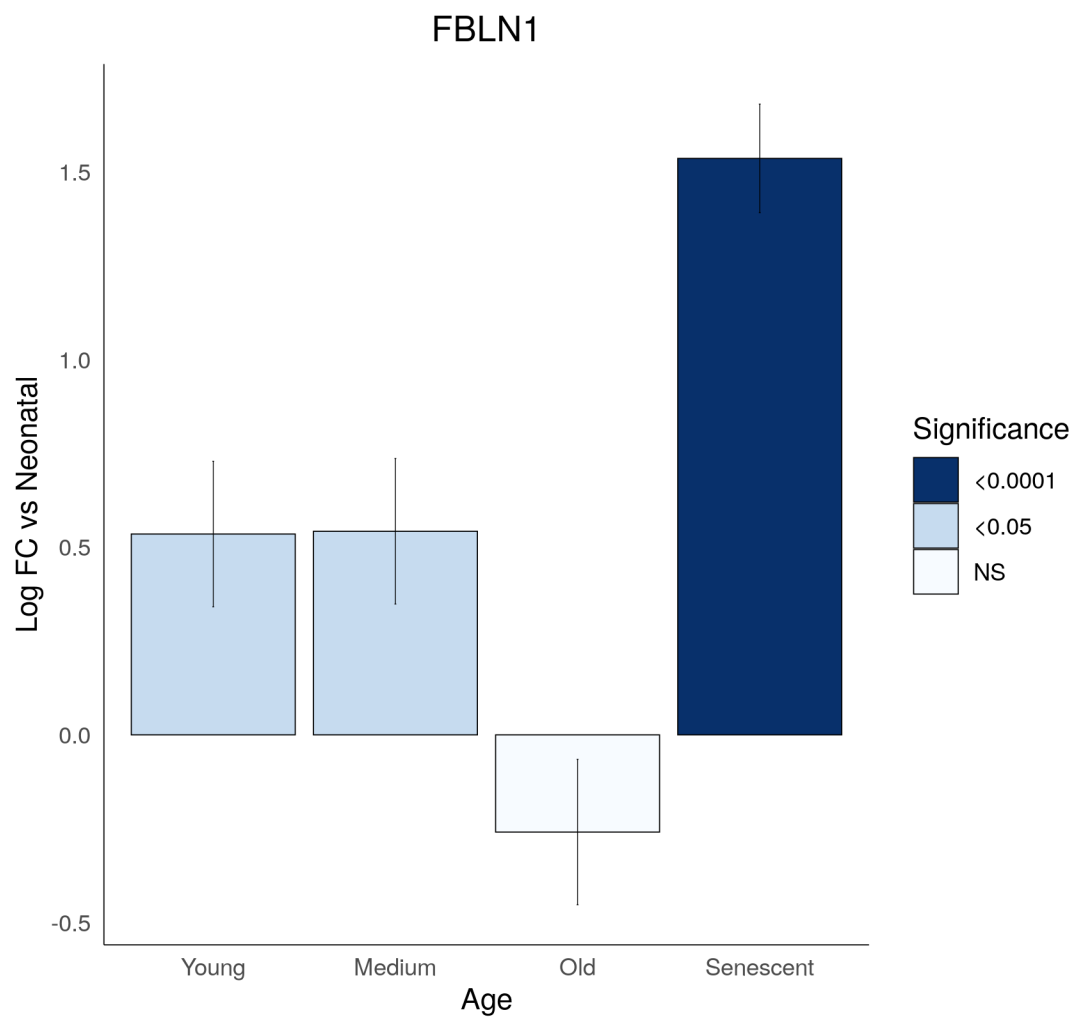

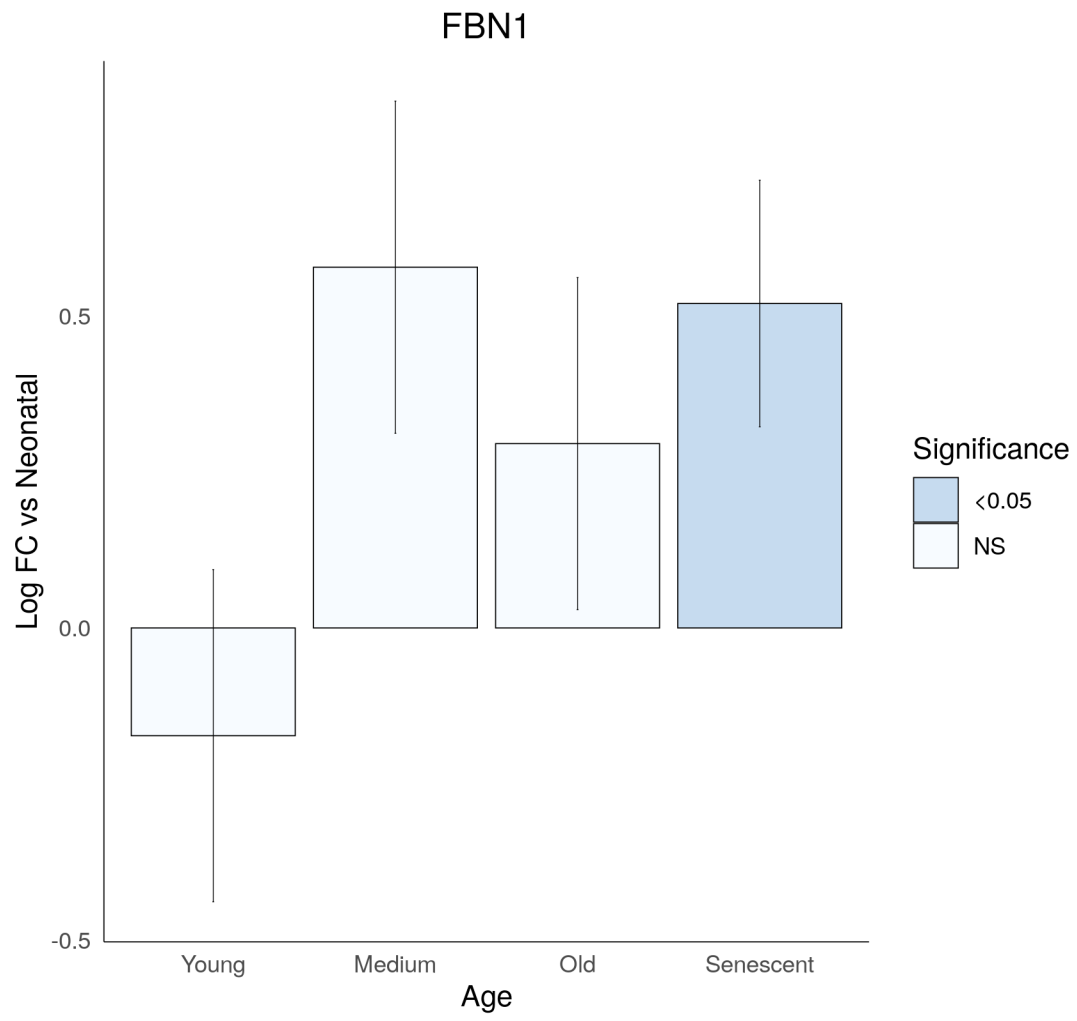

# FGFR3

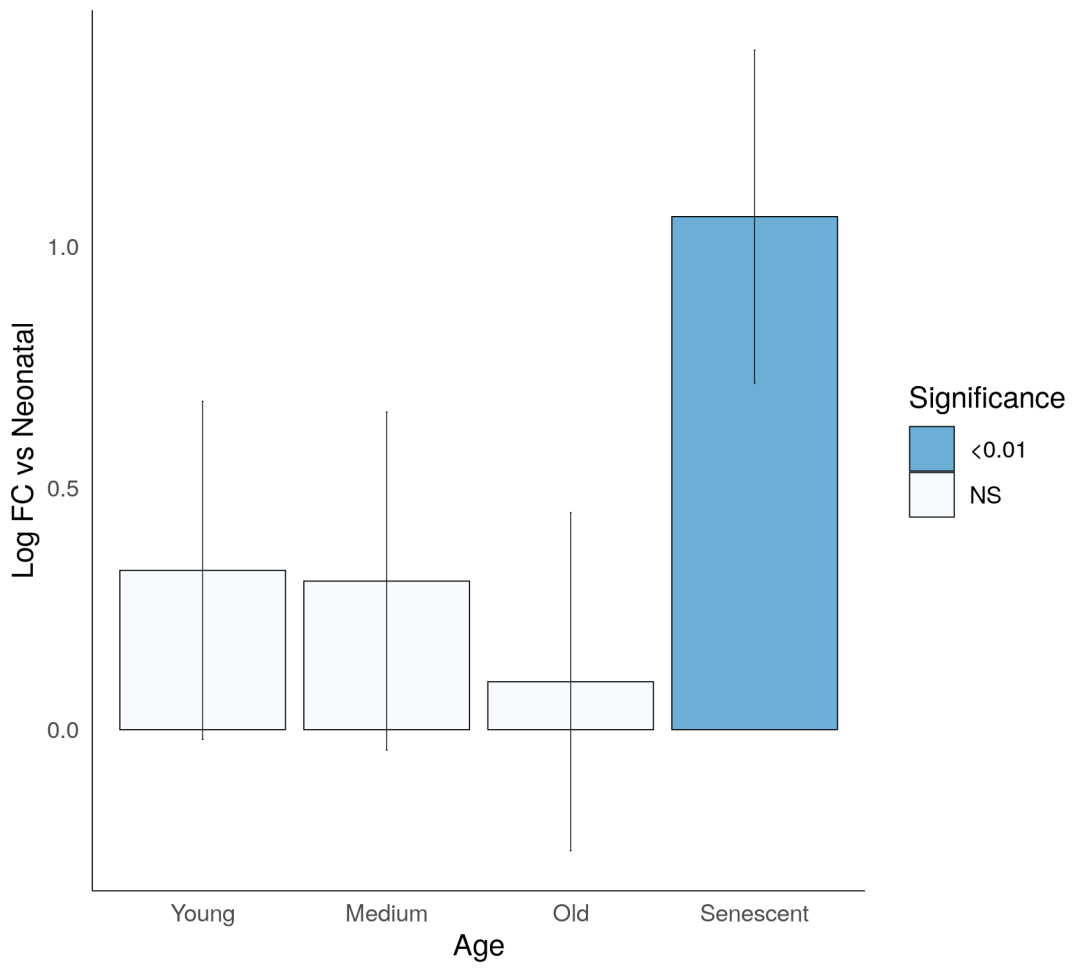

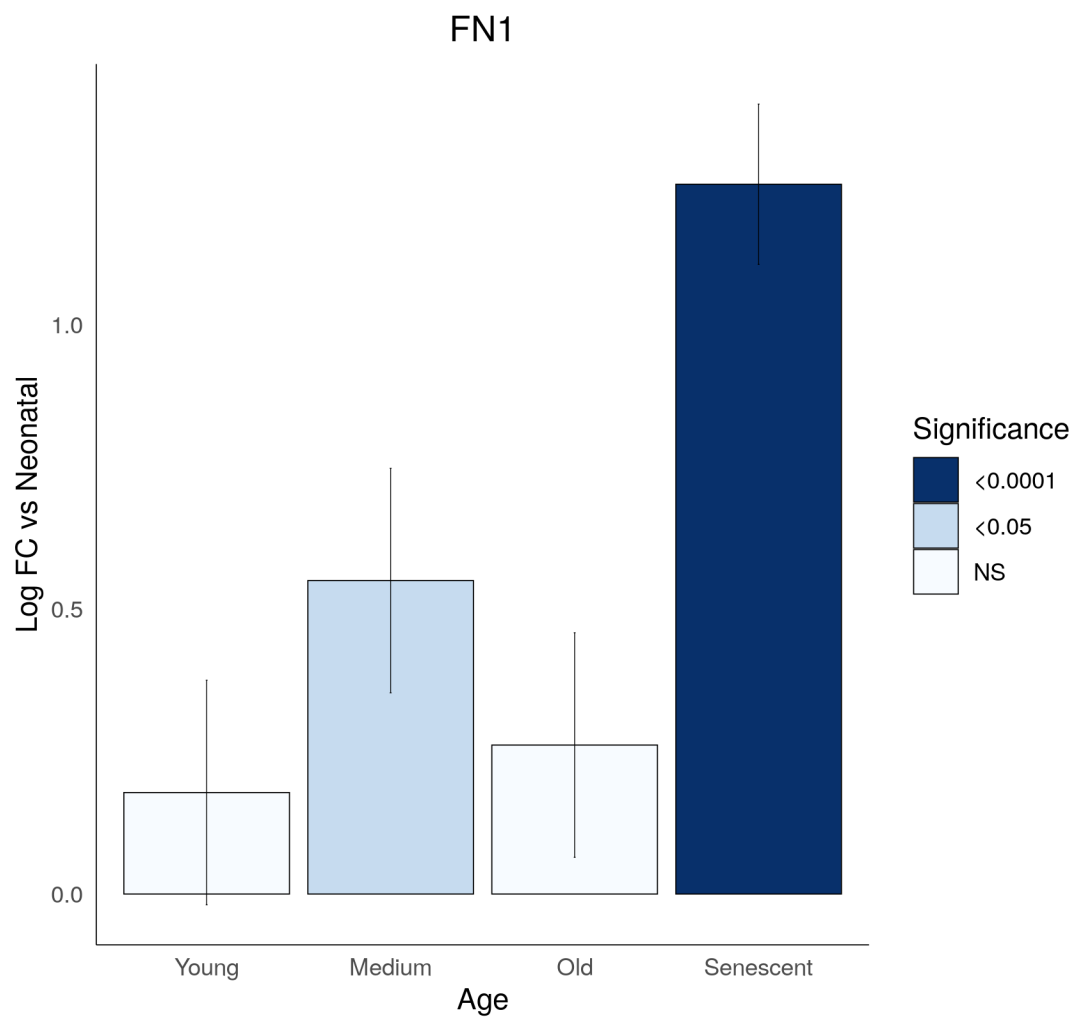

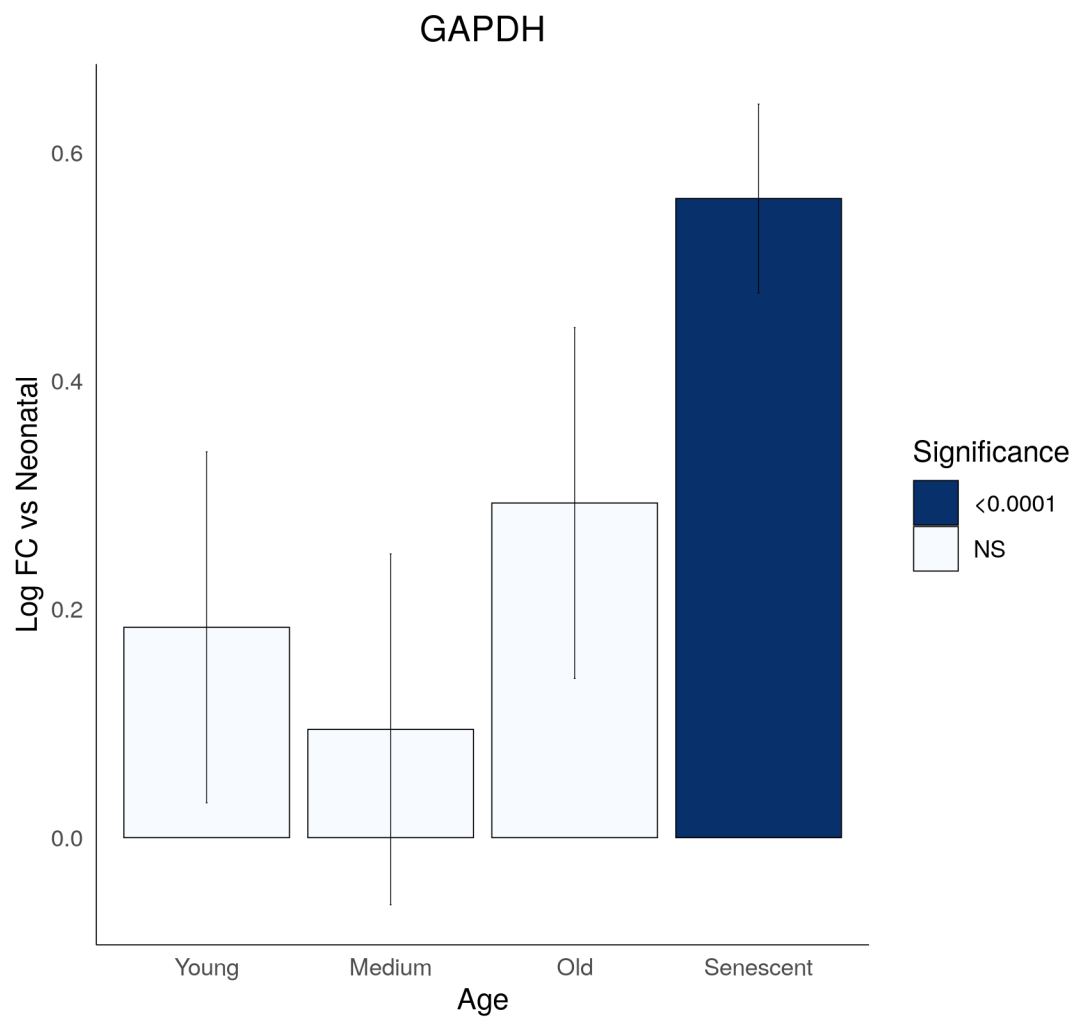

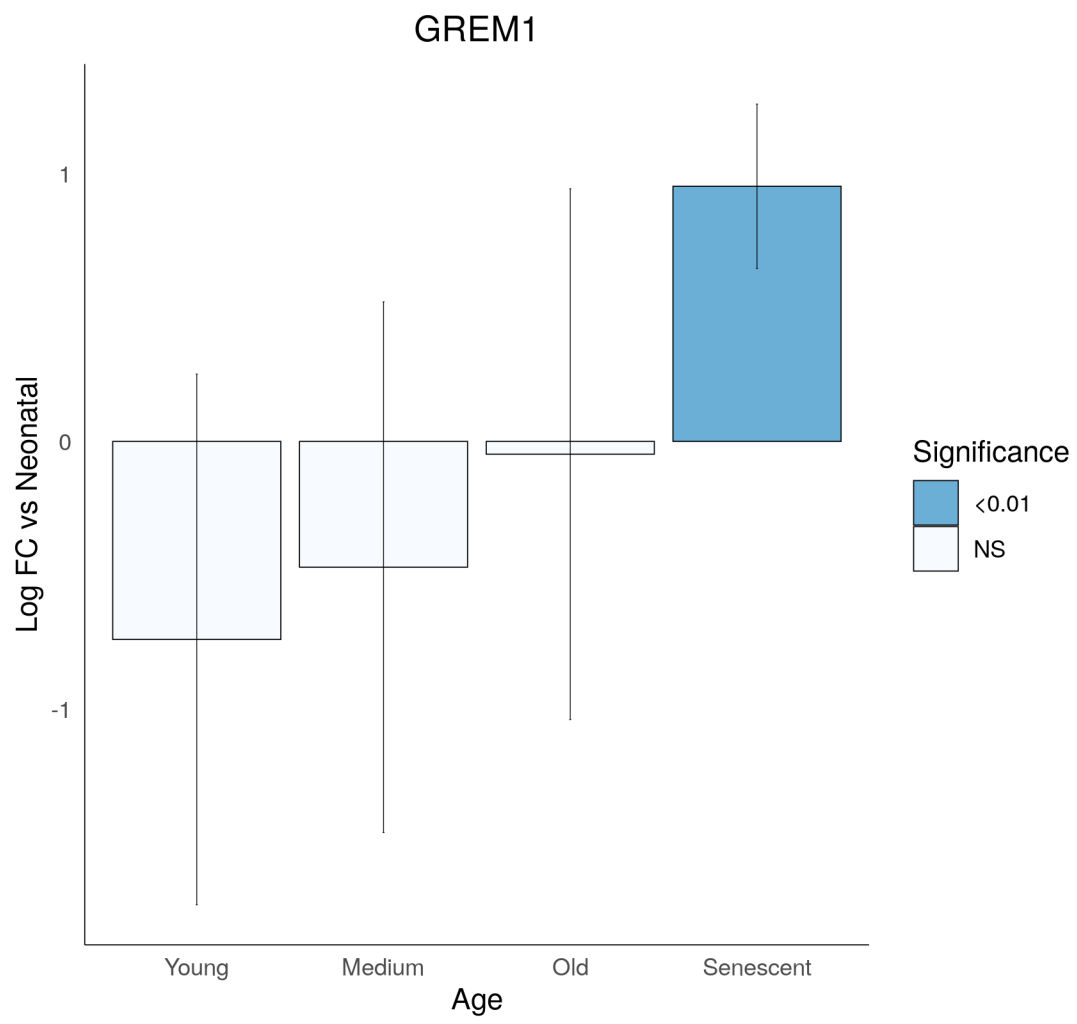

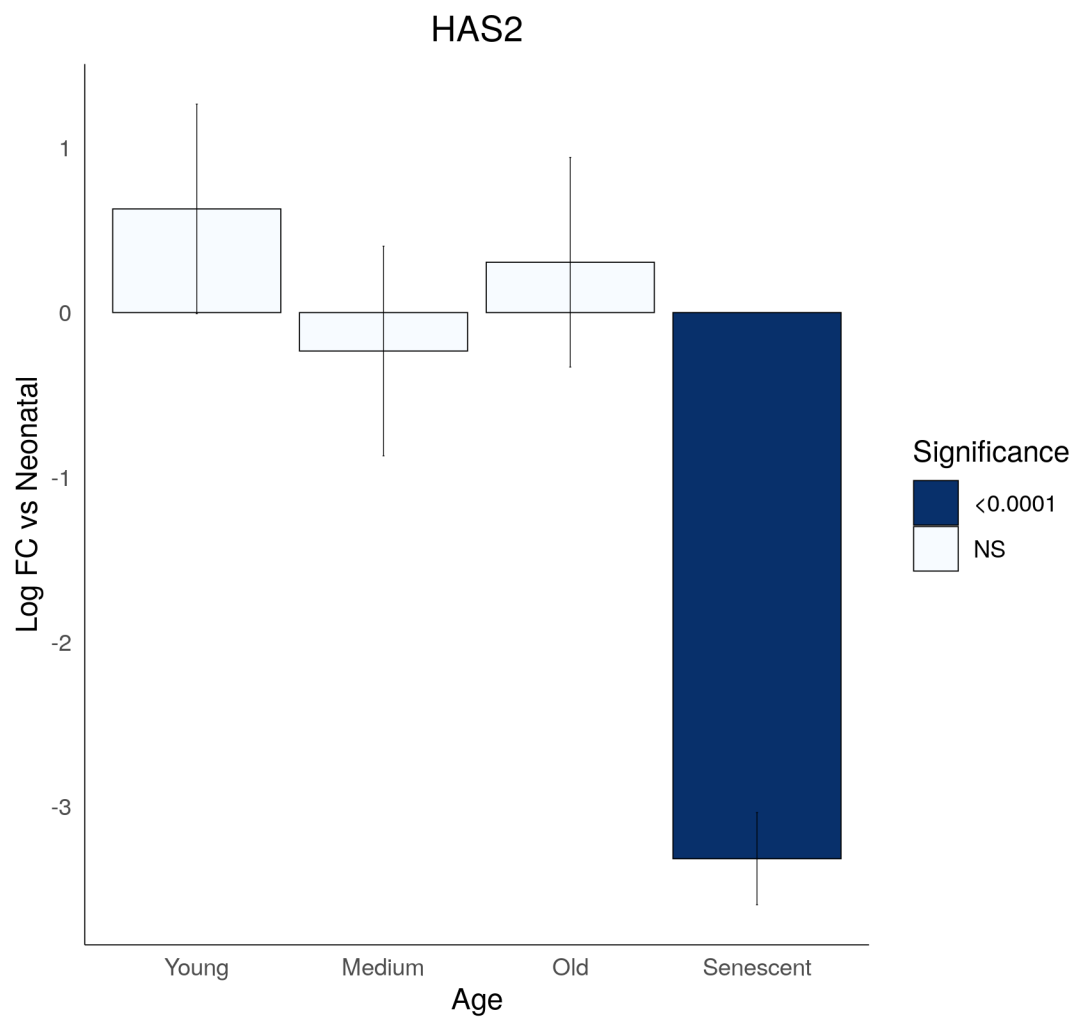

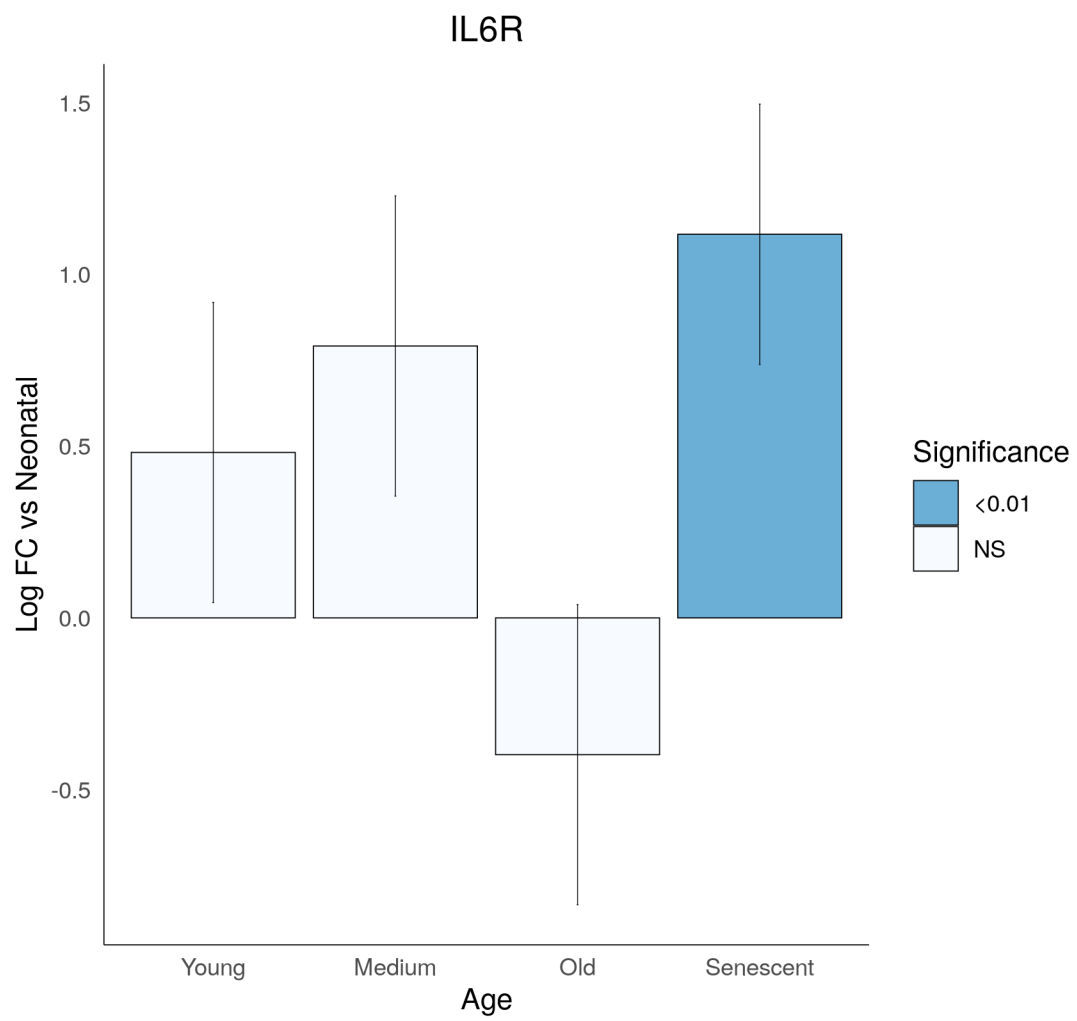

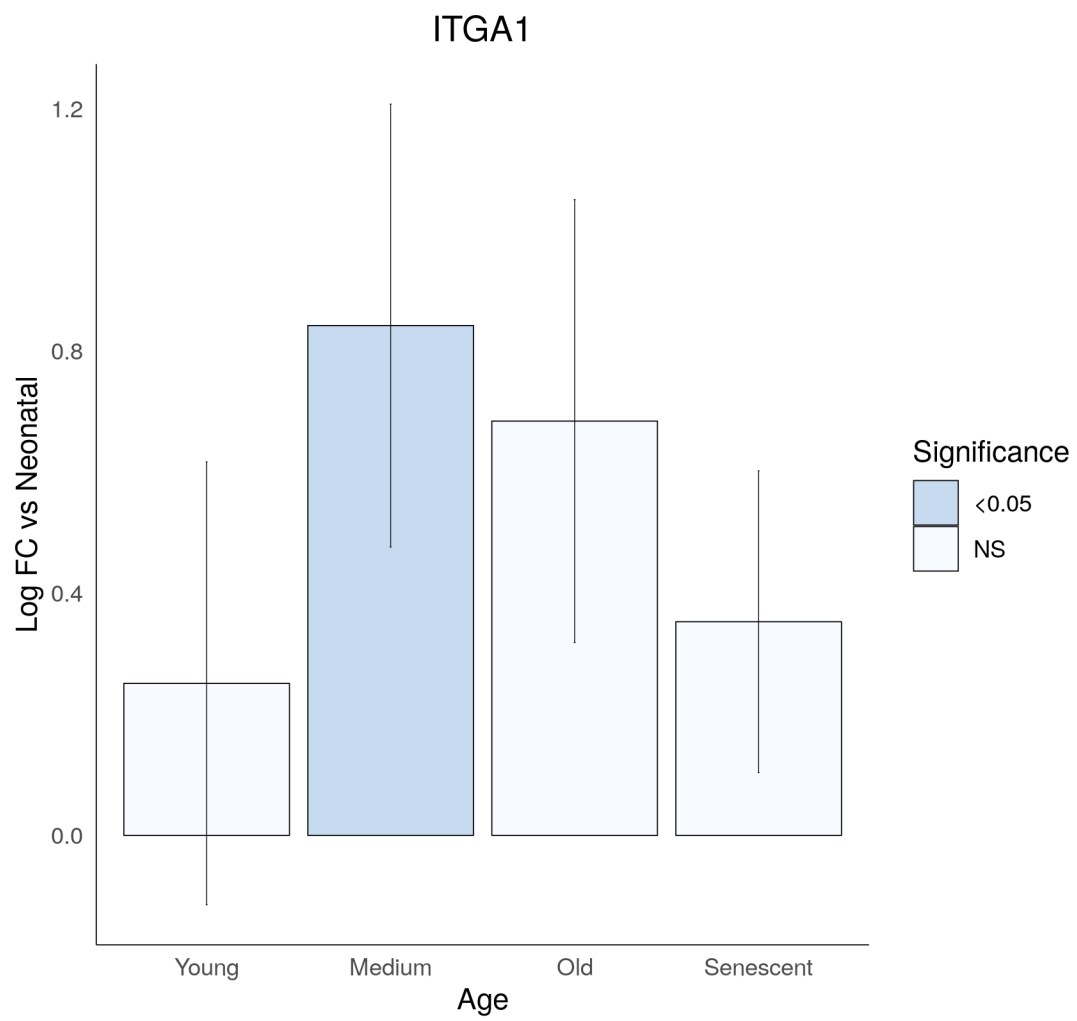

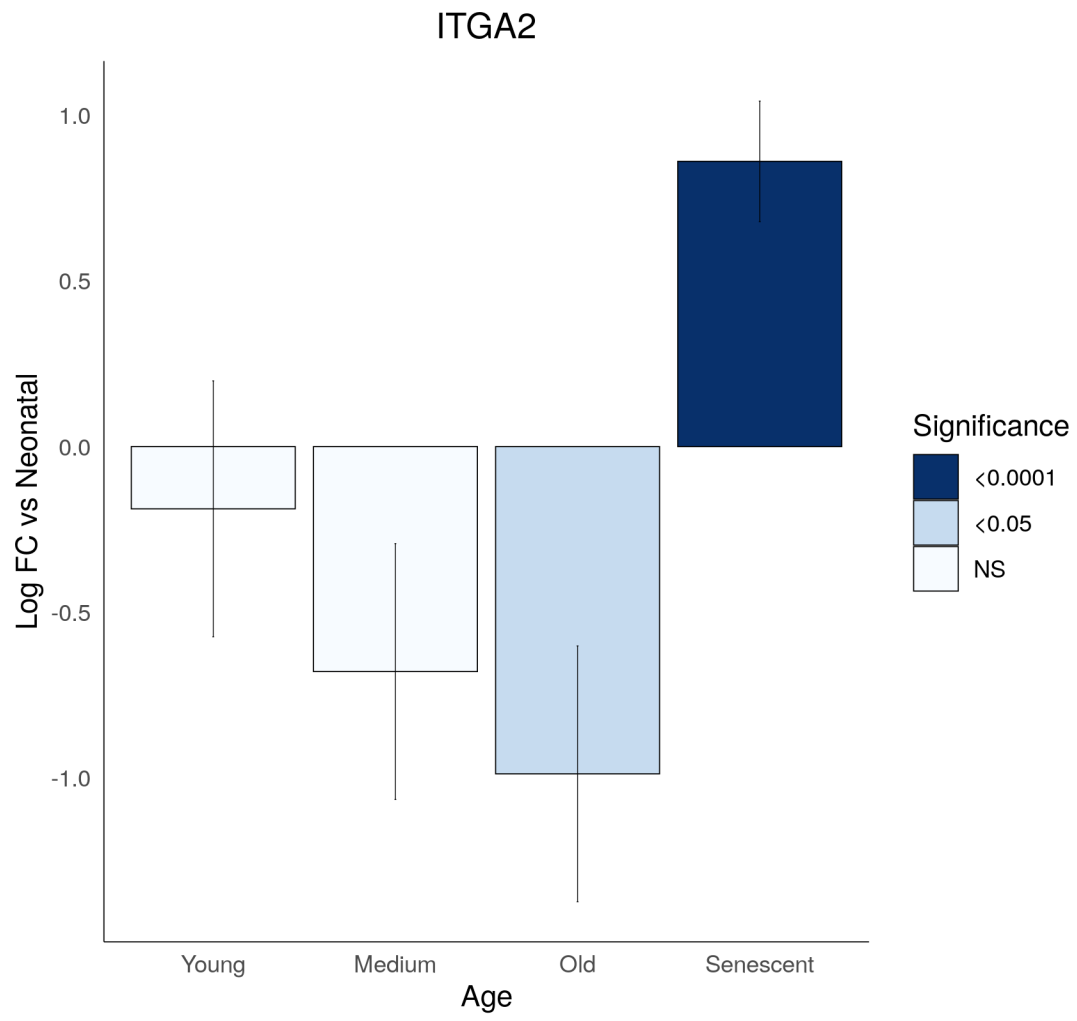

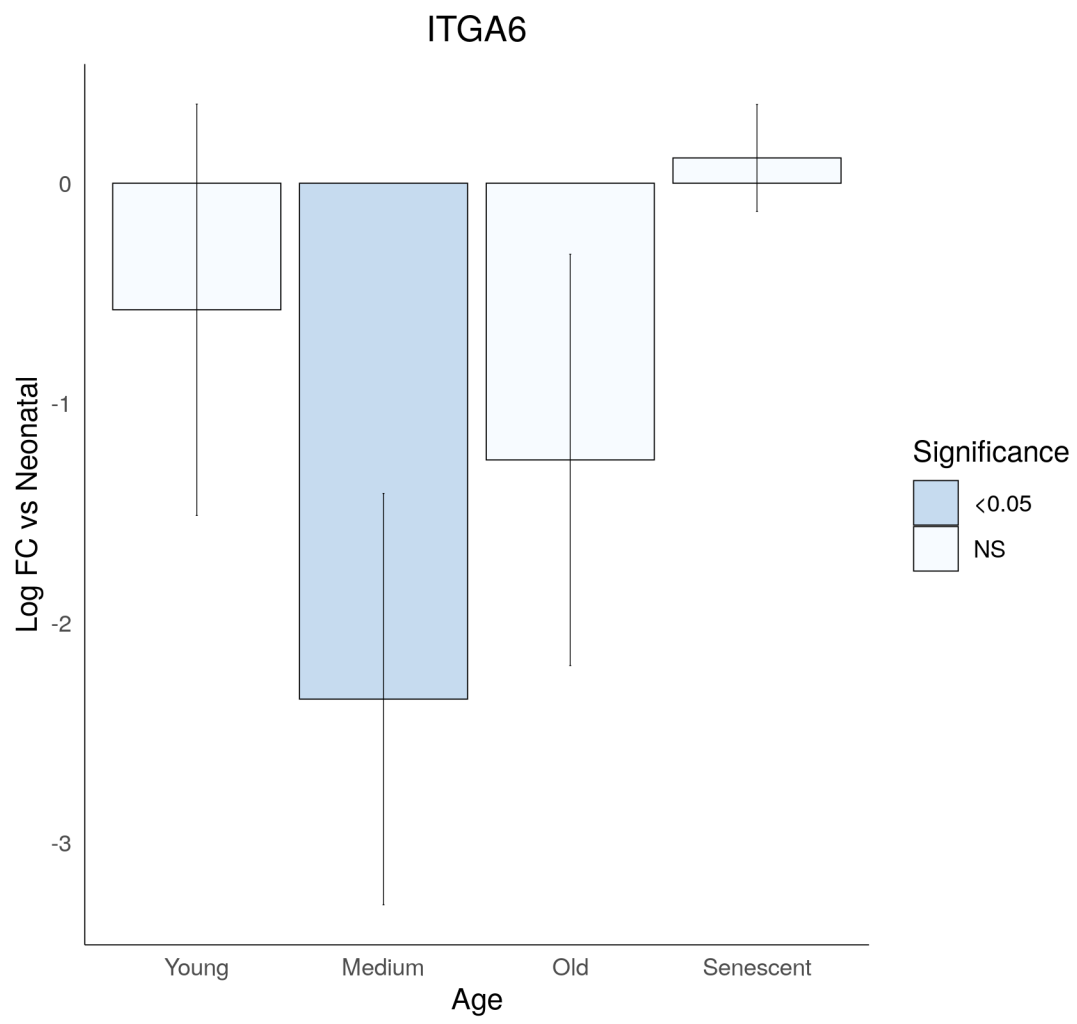

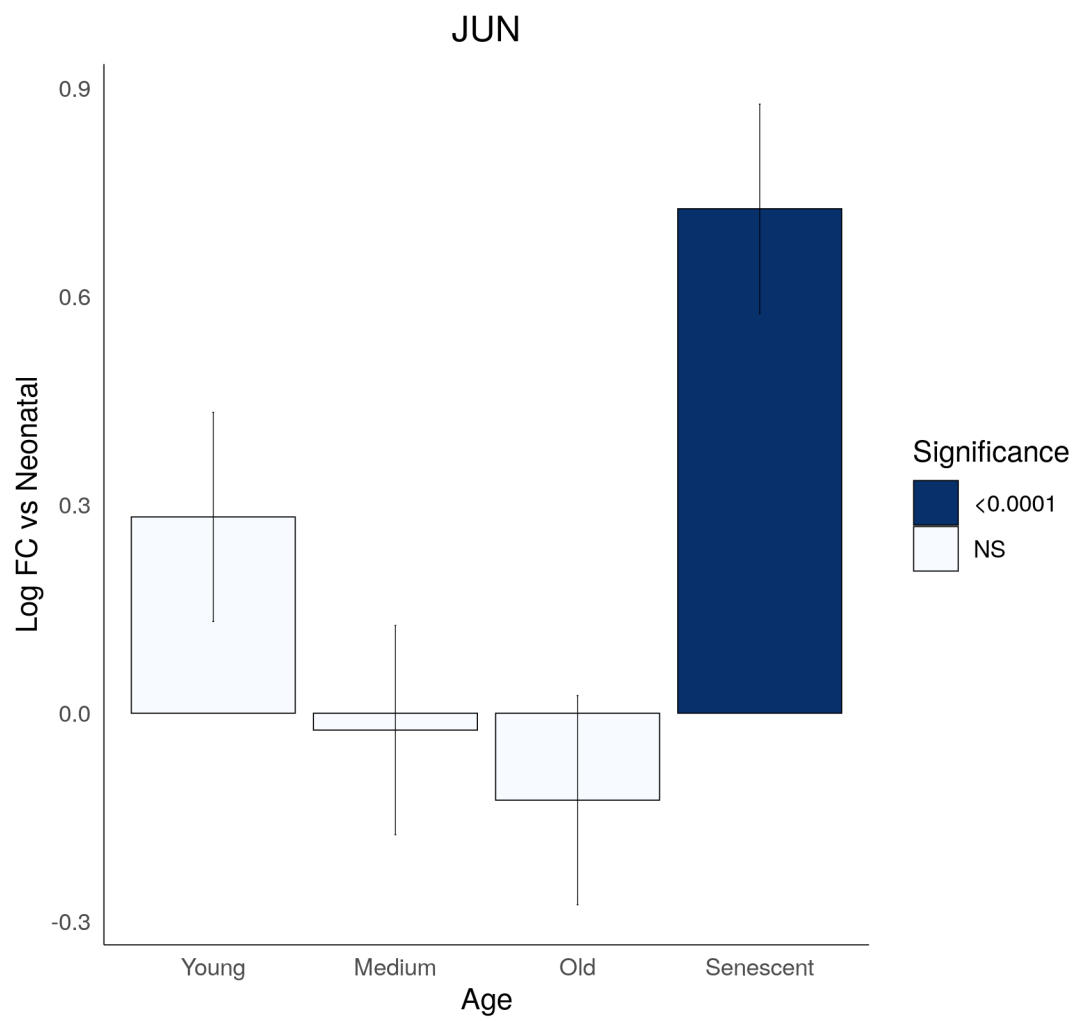

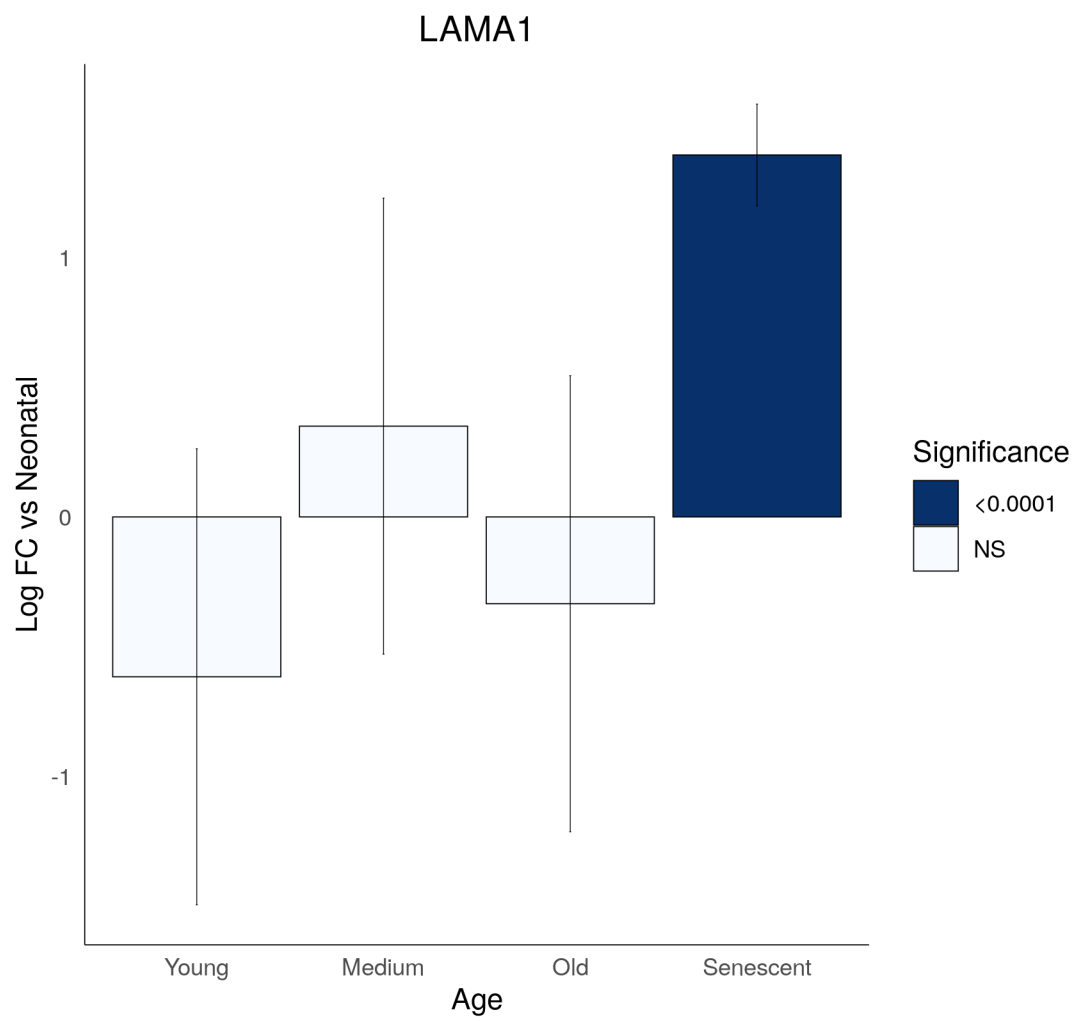

# LAMA3

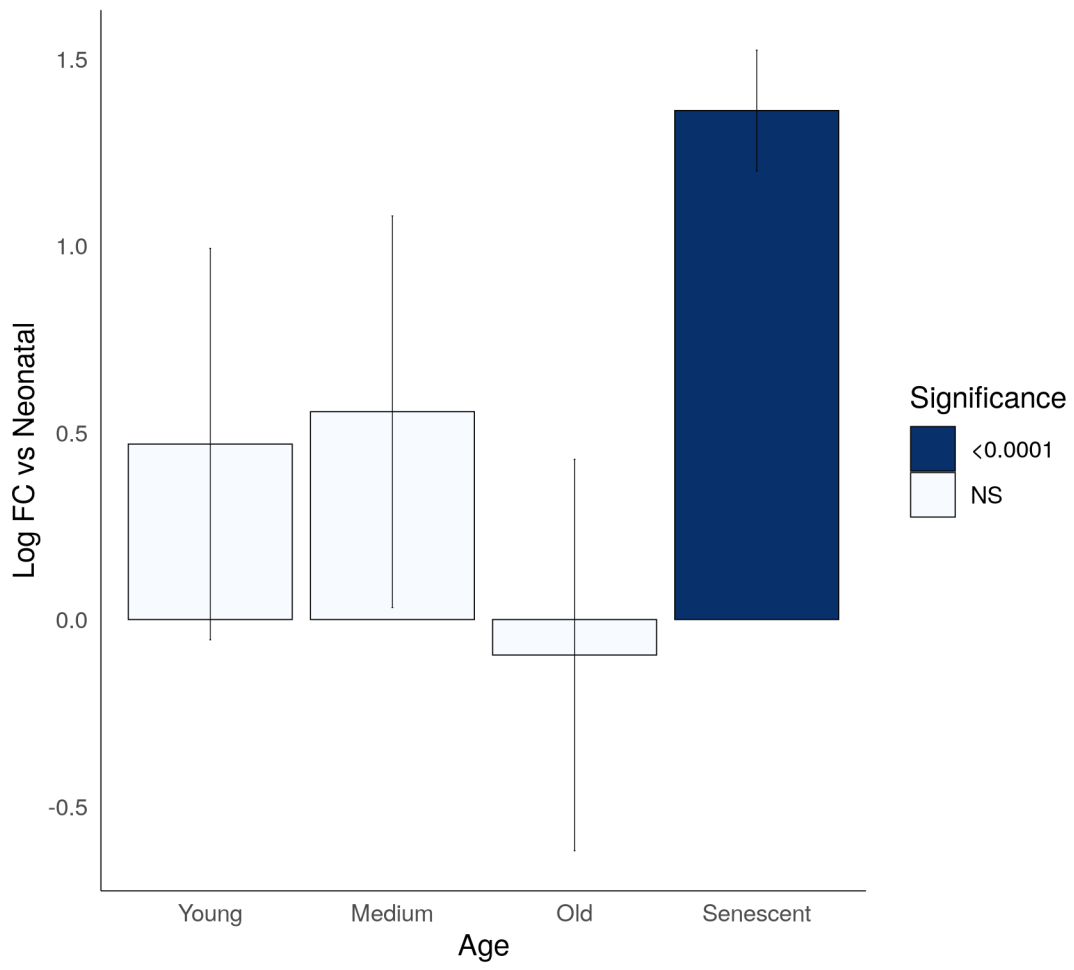

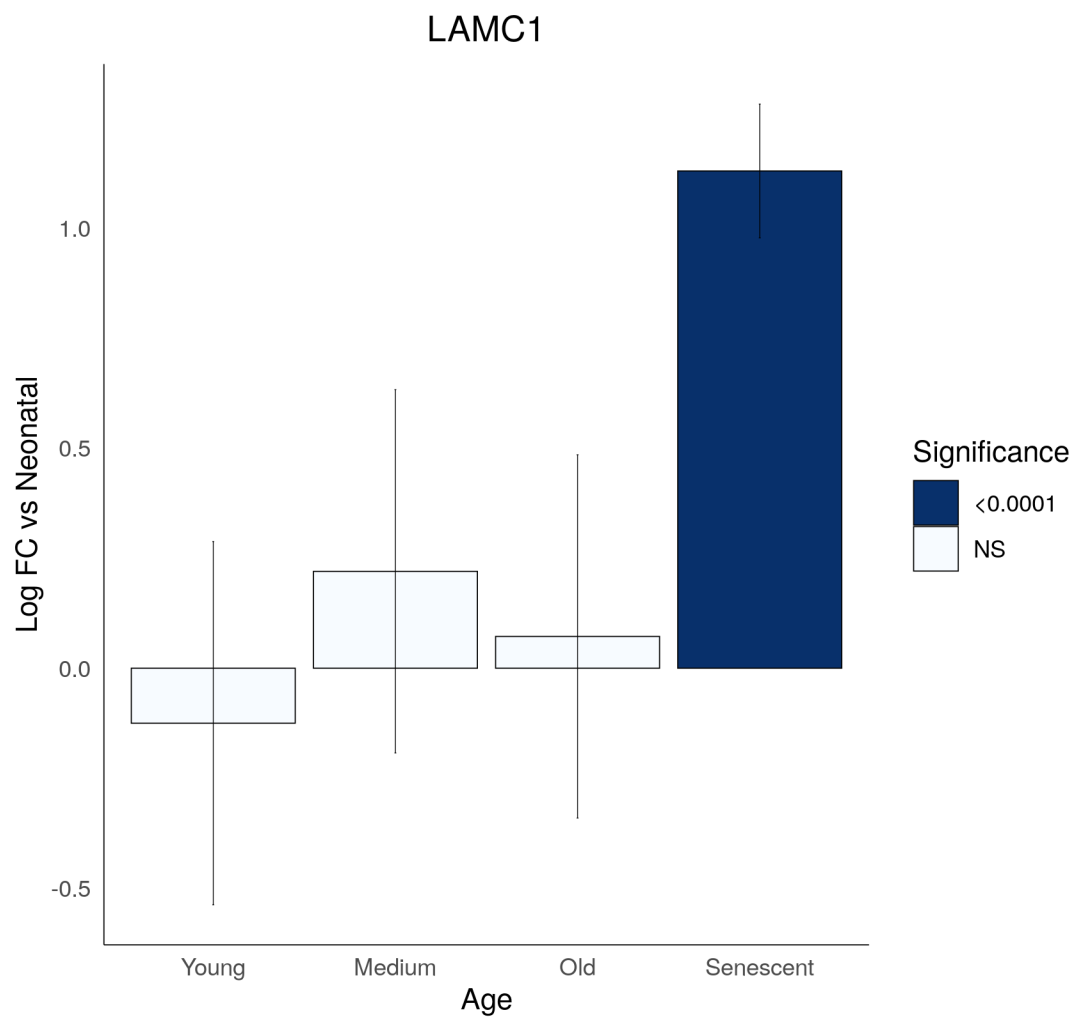

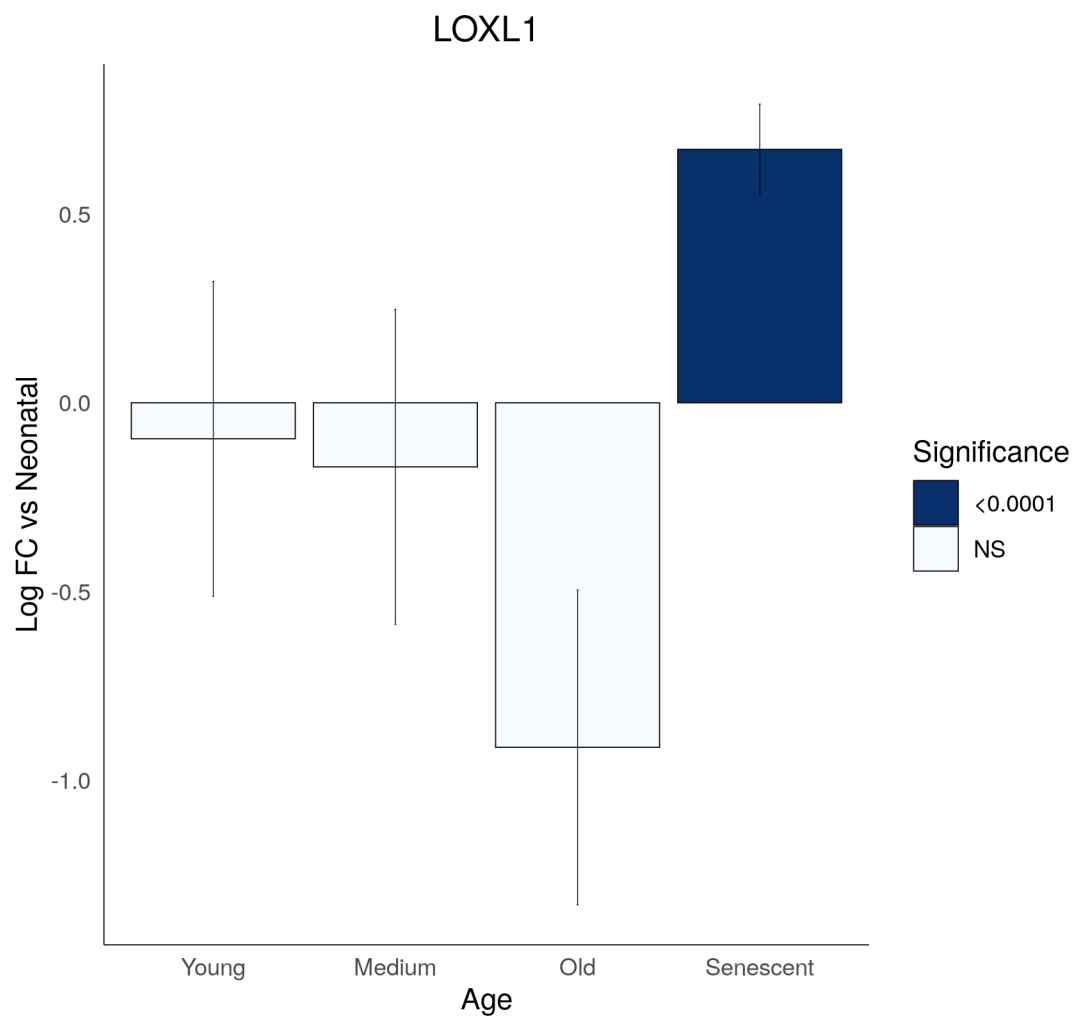

# LOXL2

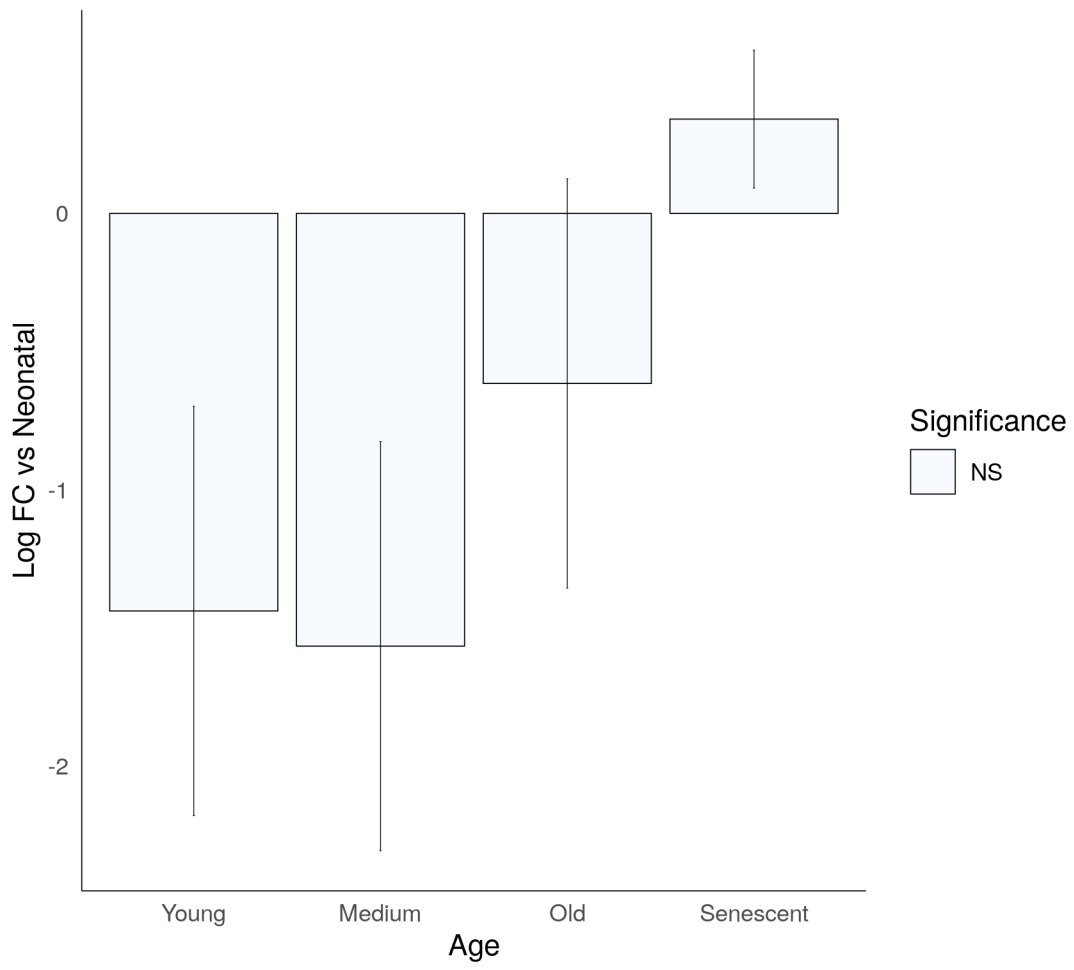

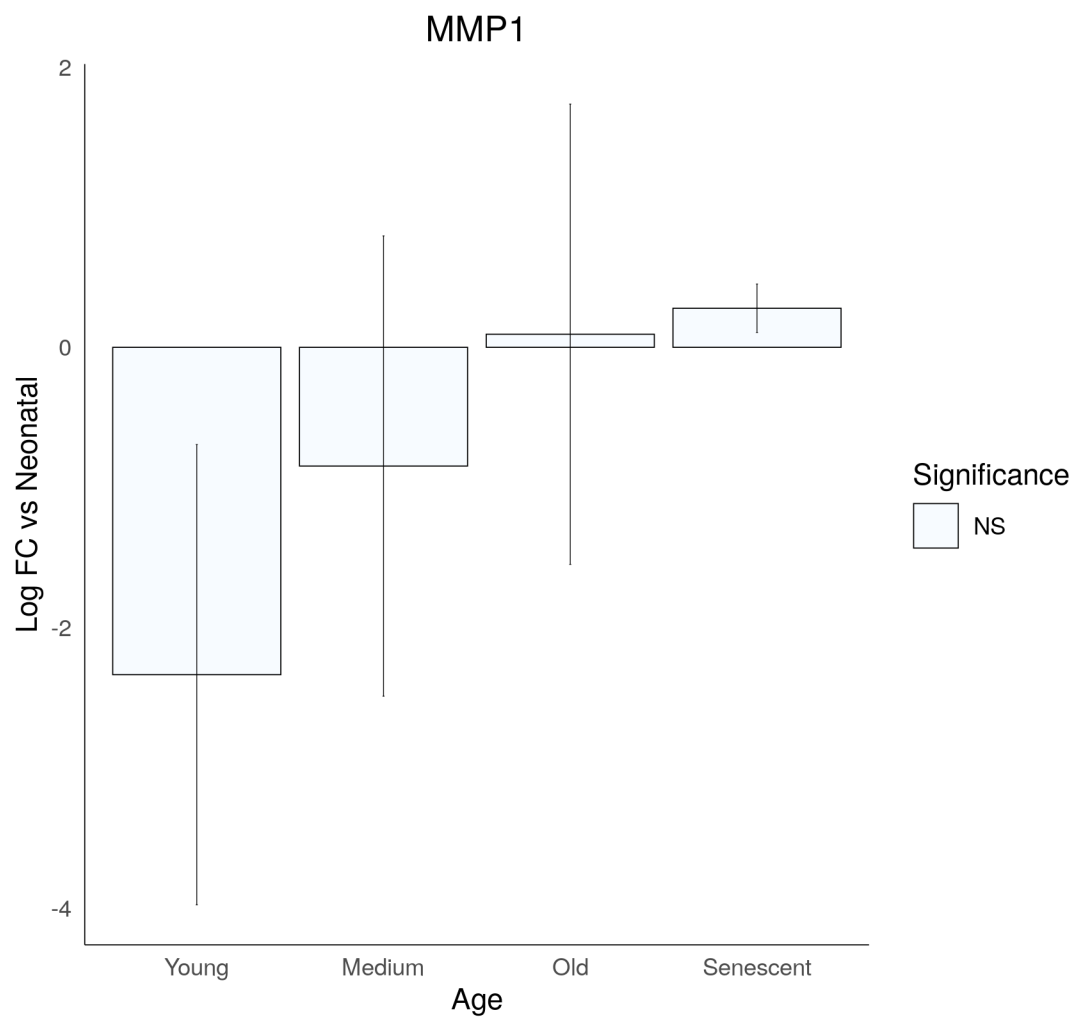

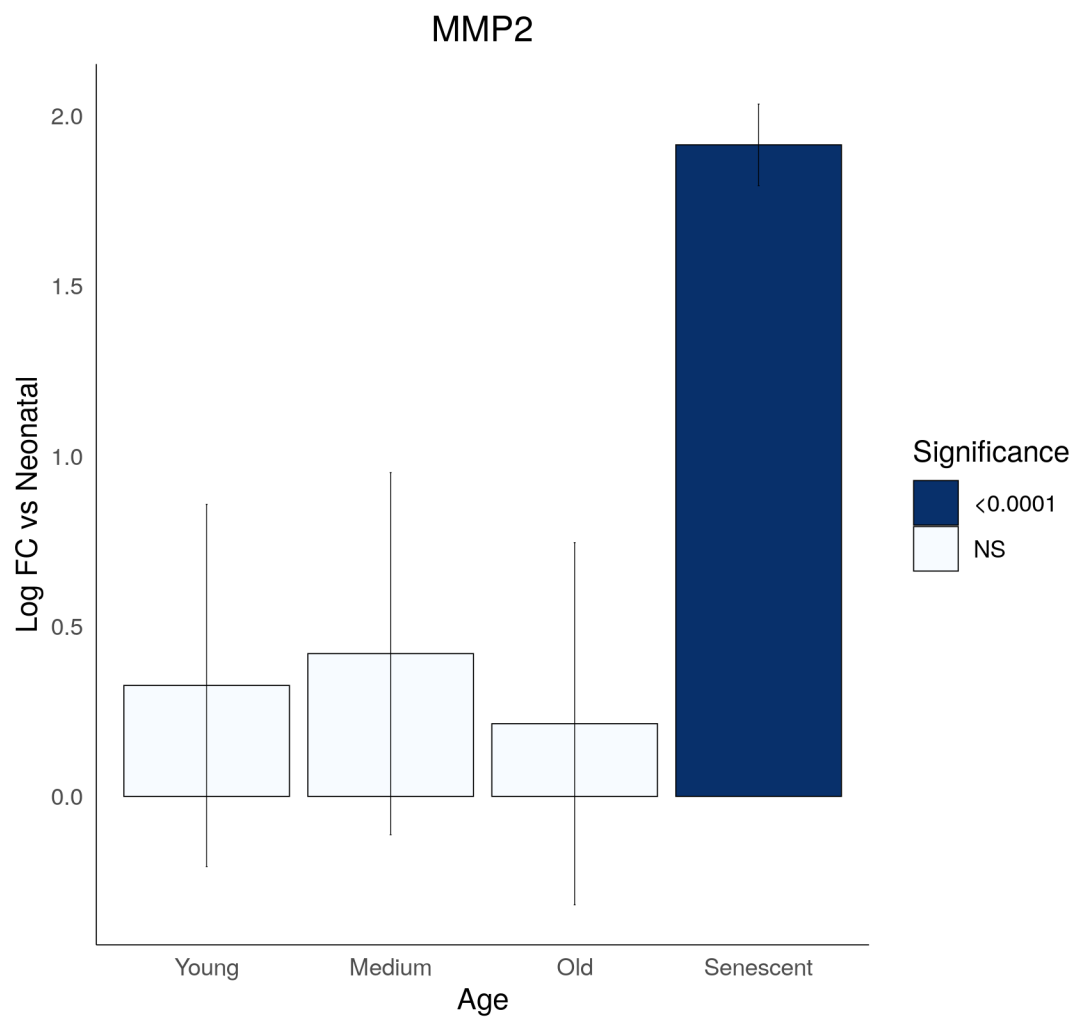

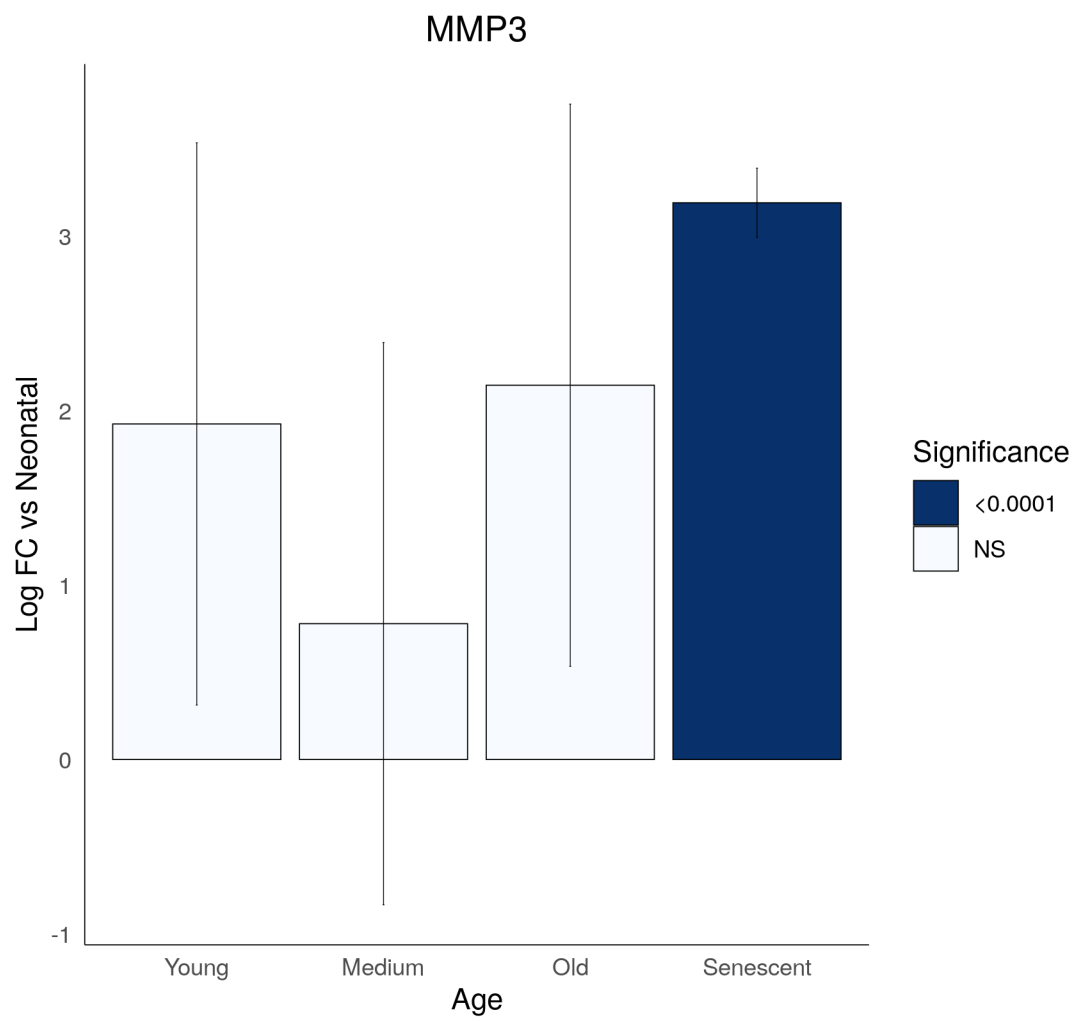

# MMP9

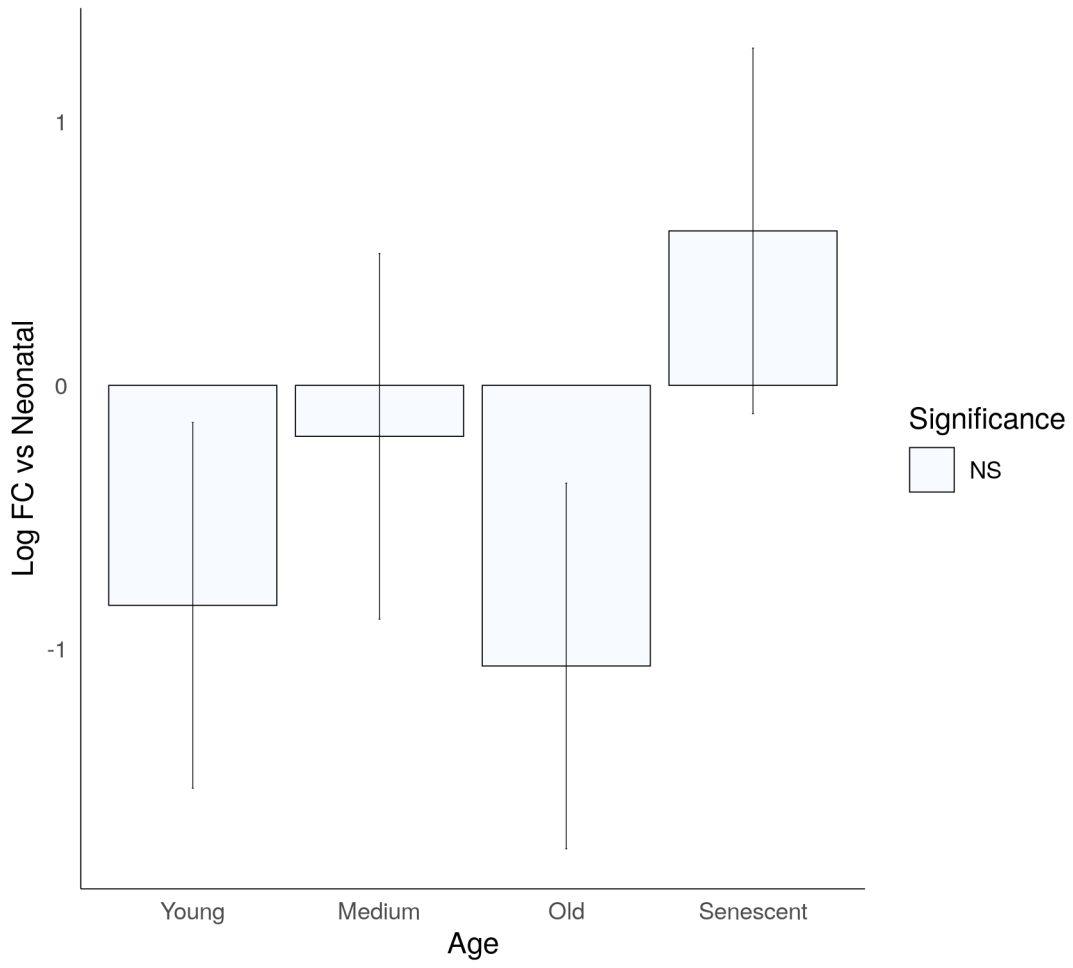

## MMP14

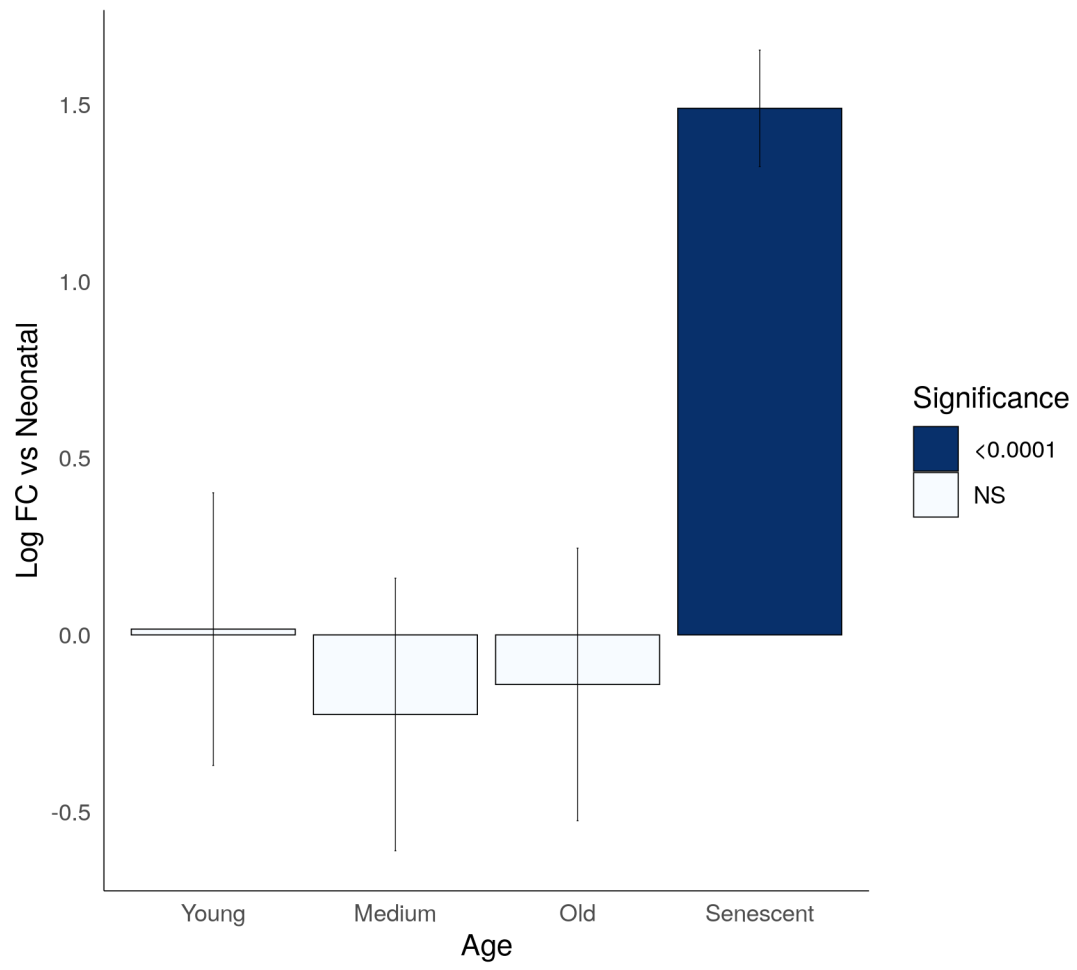

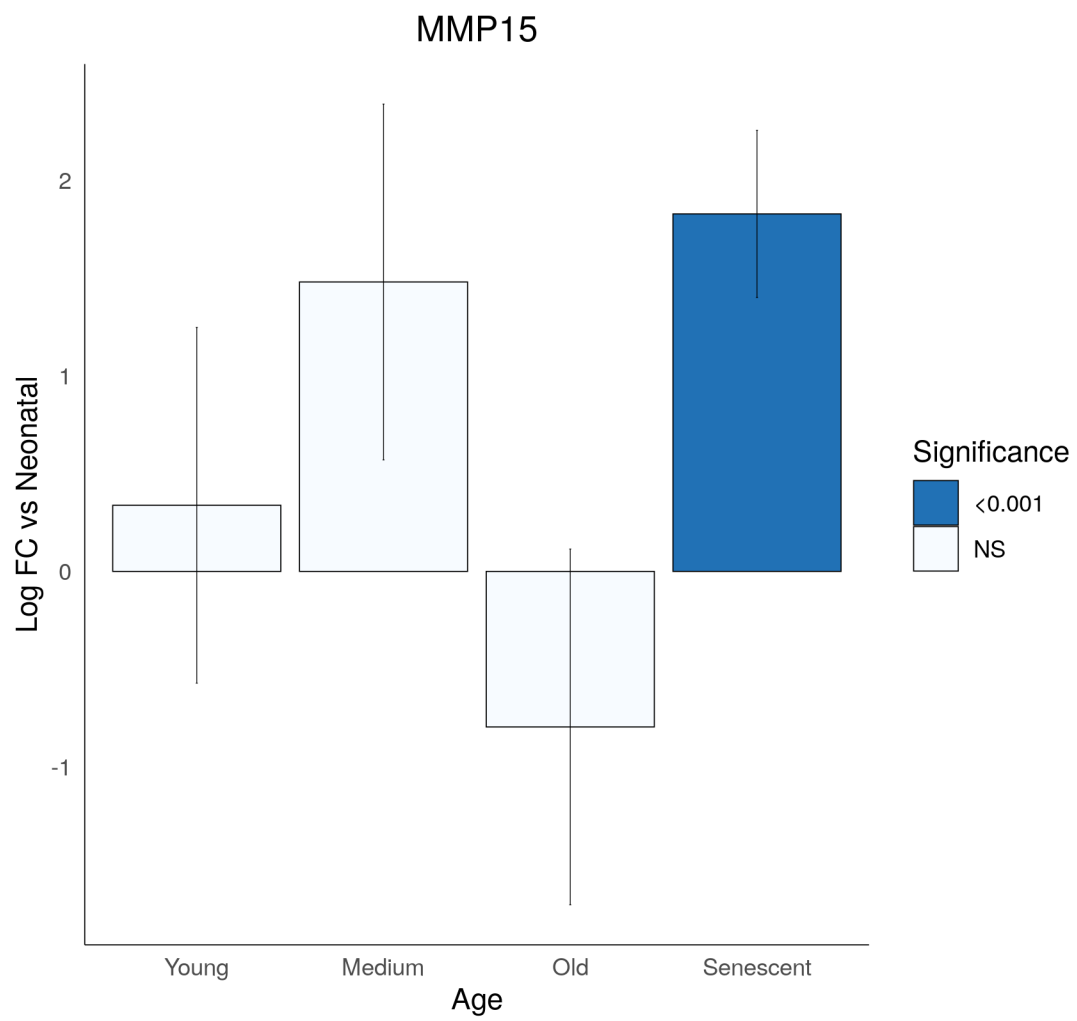

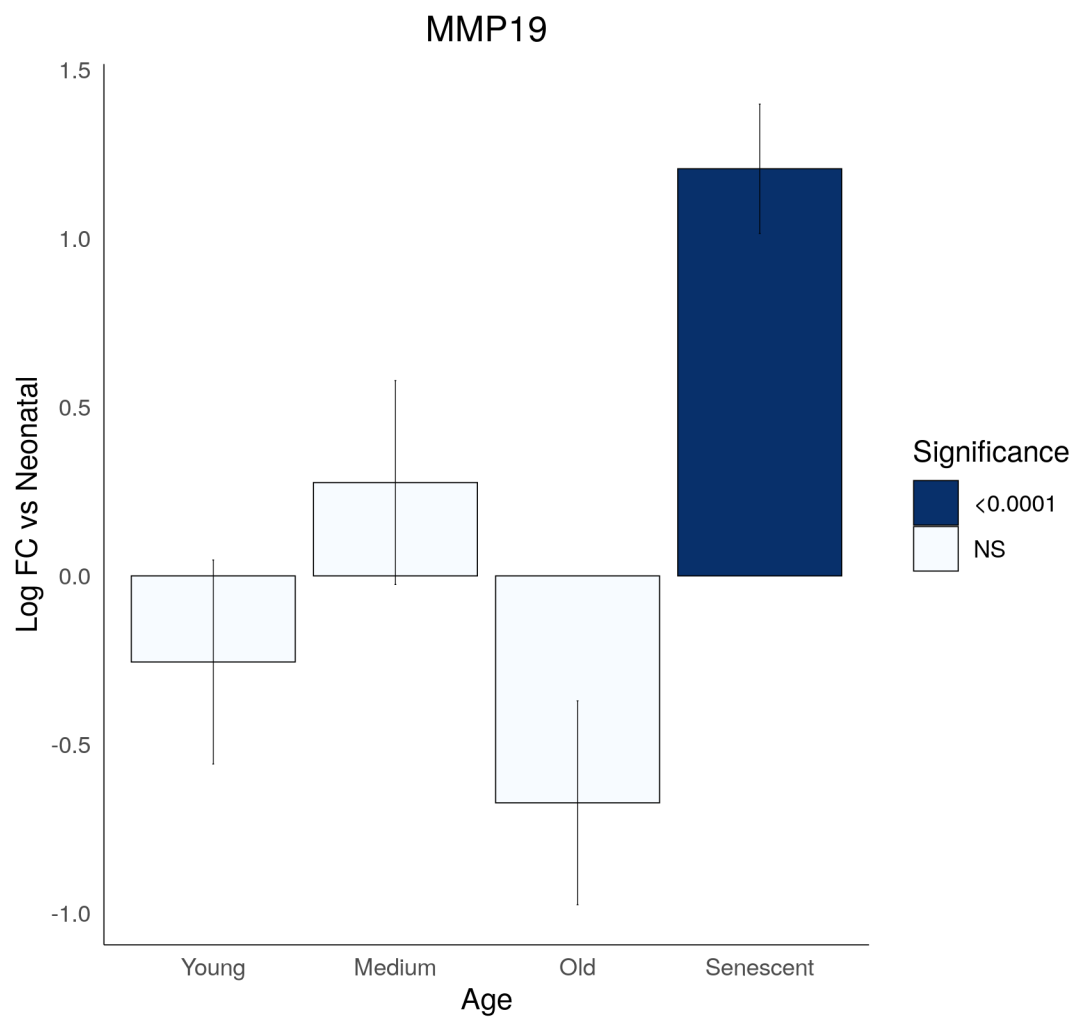

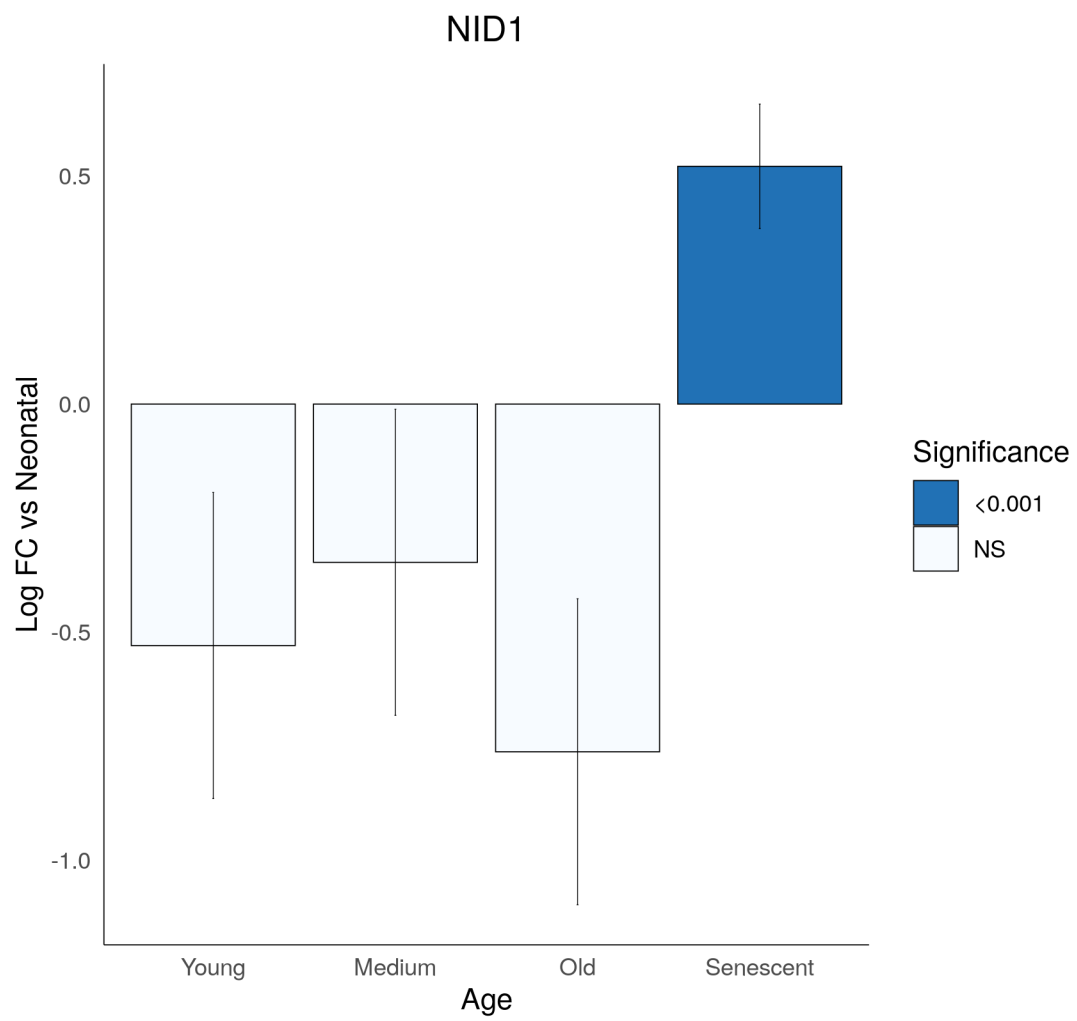

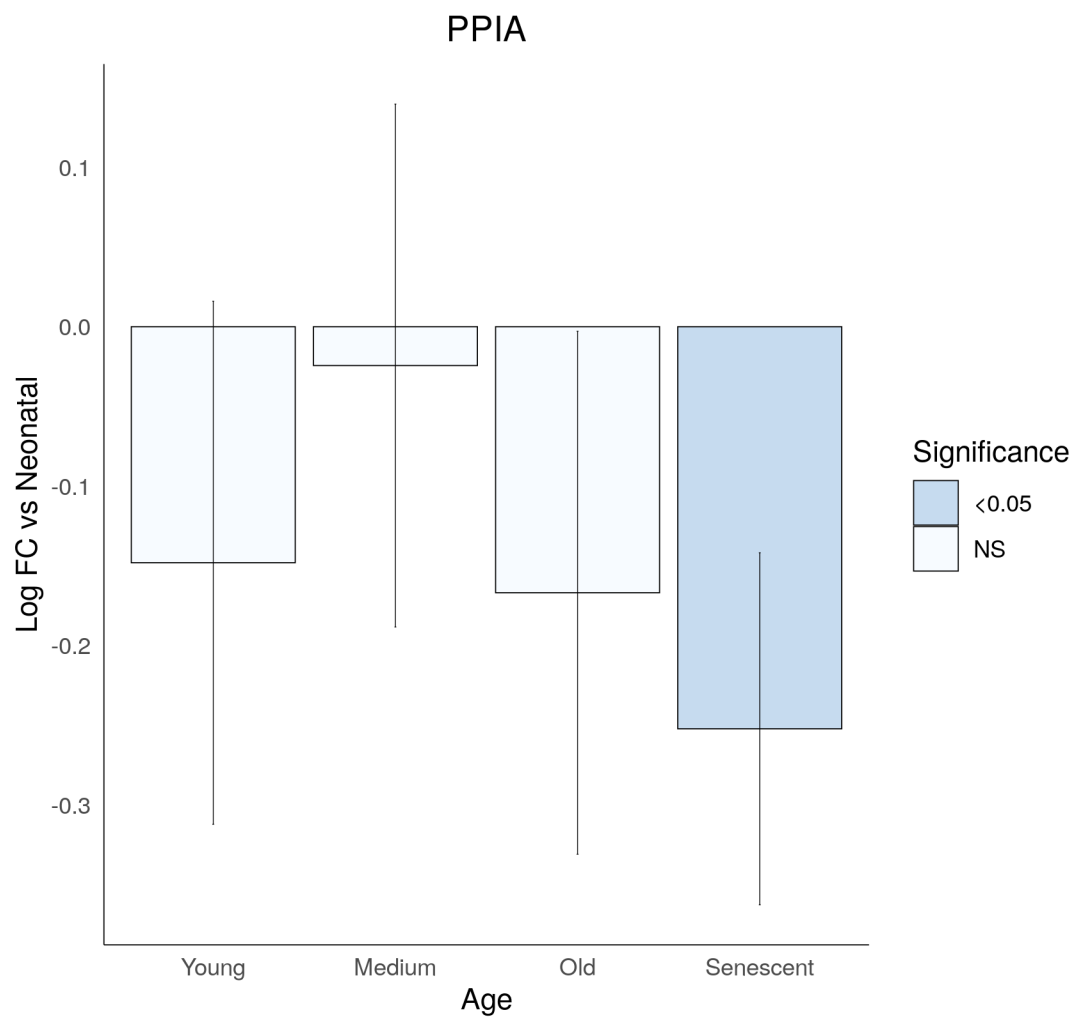

PTGER3

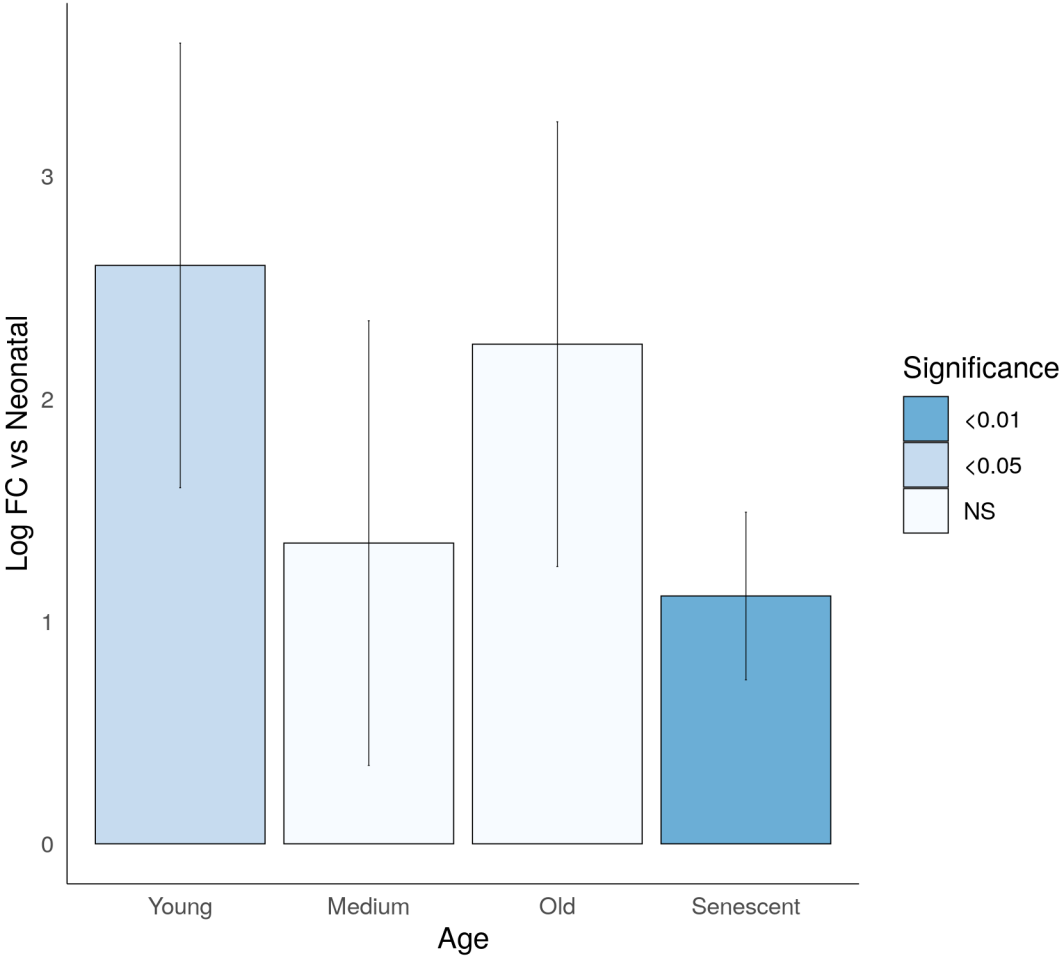

SERPINE1

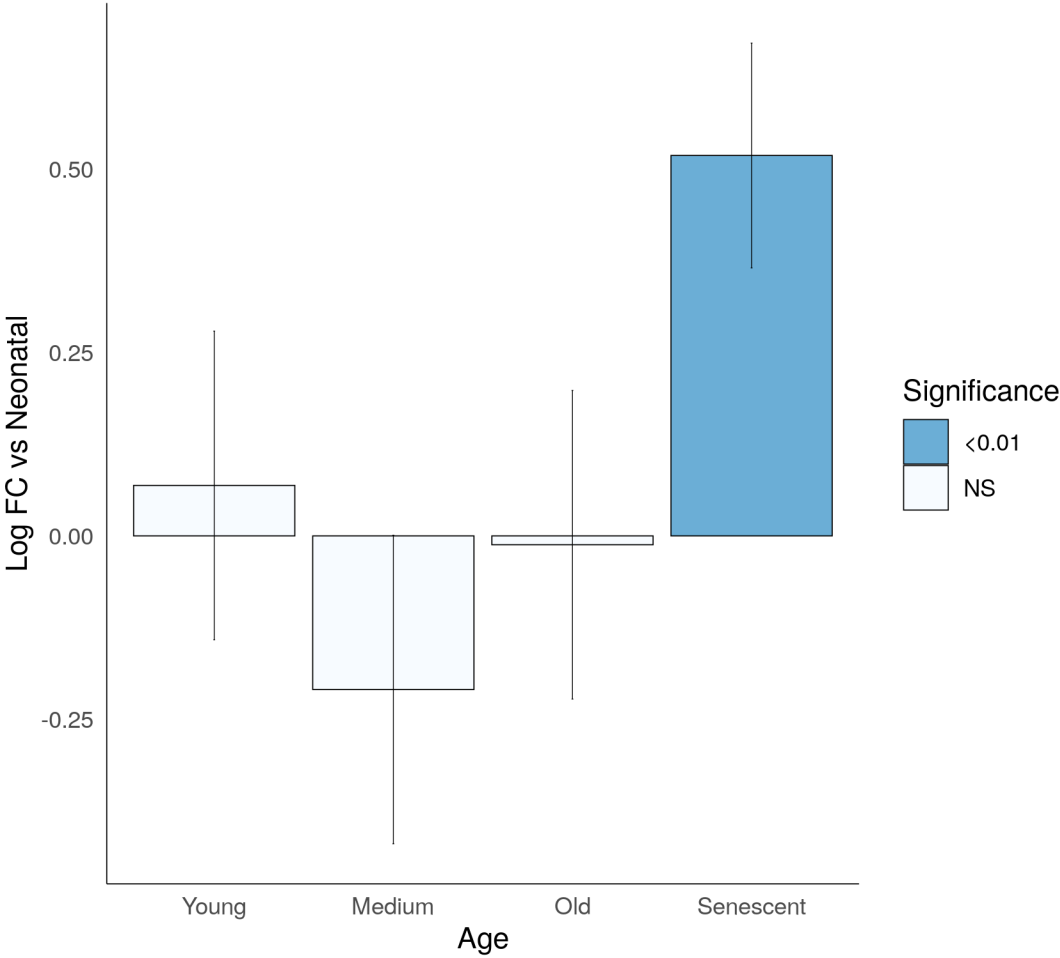

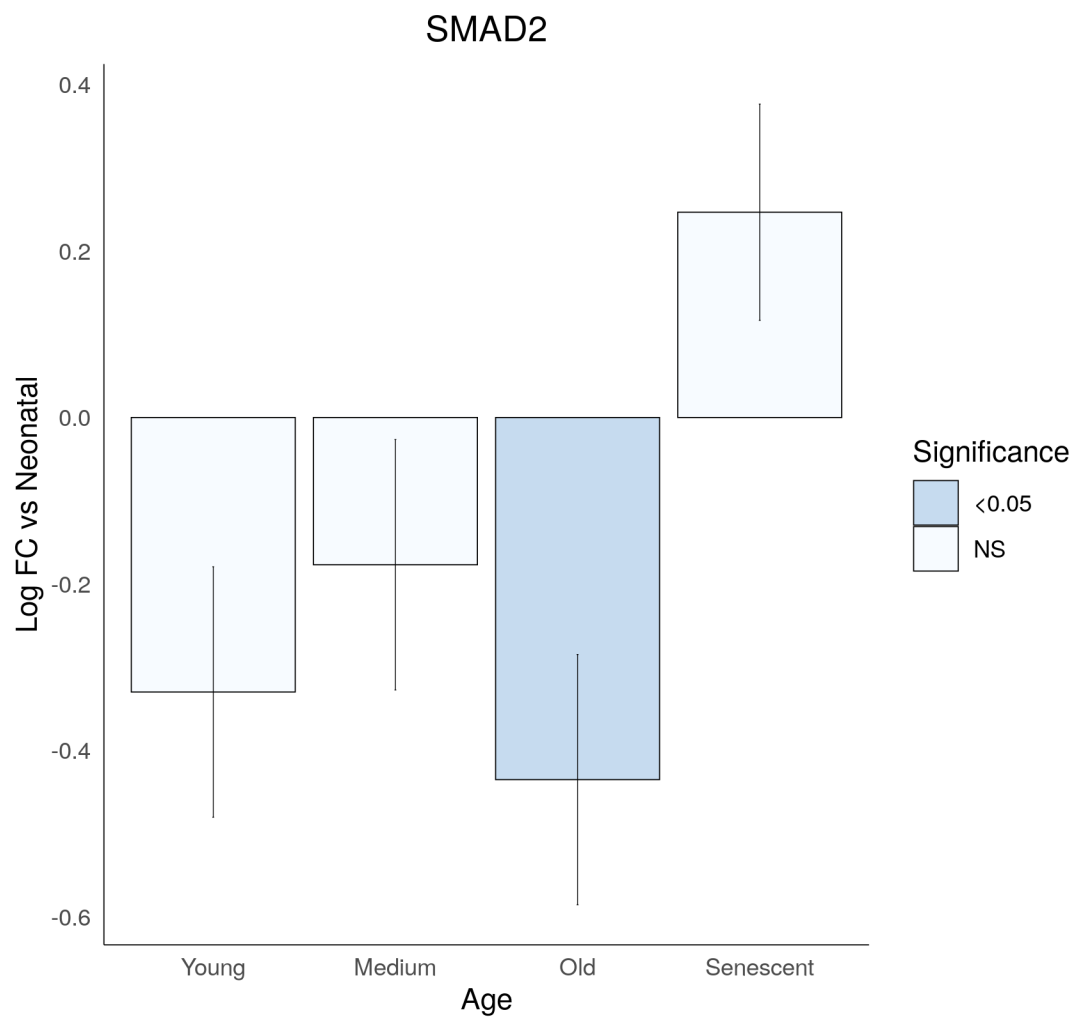

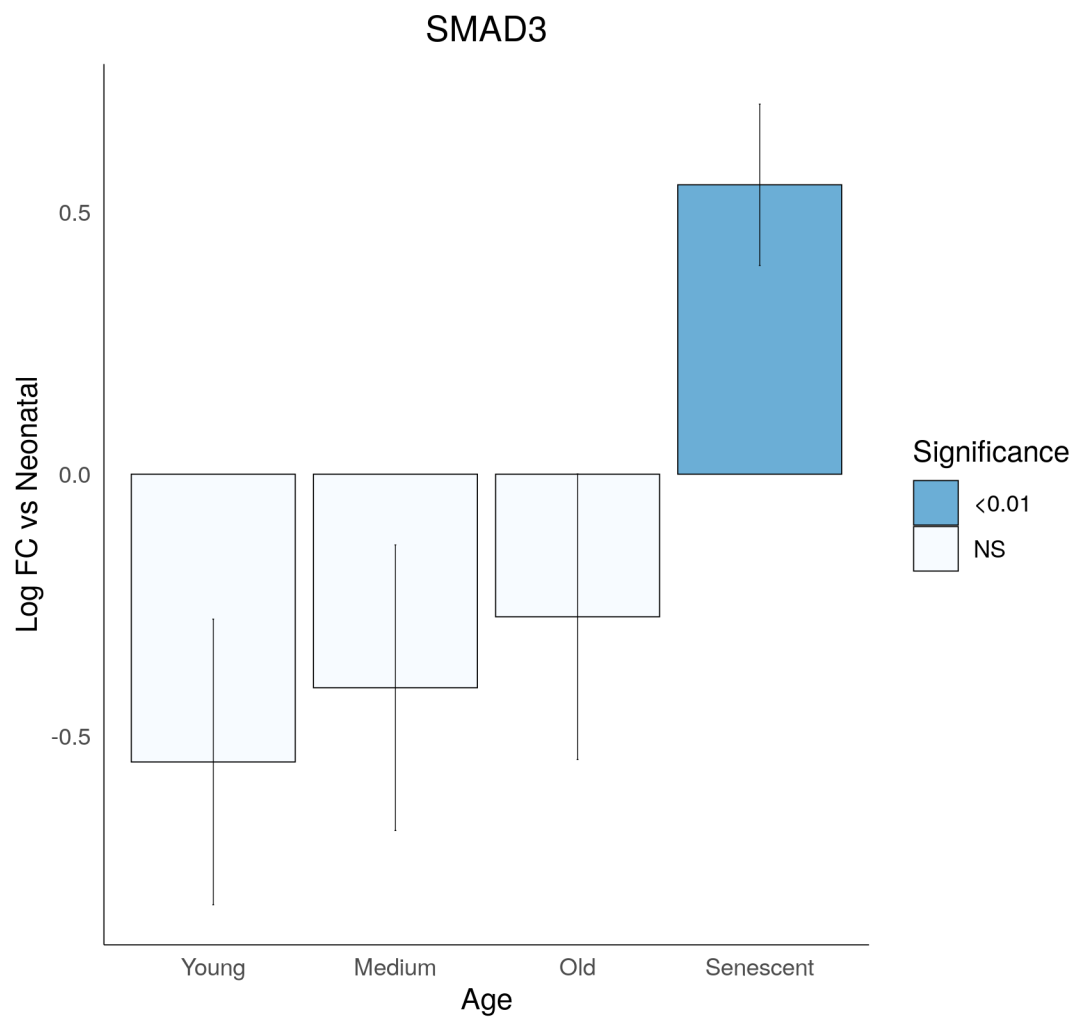

# SMAD4

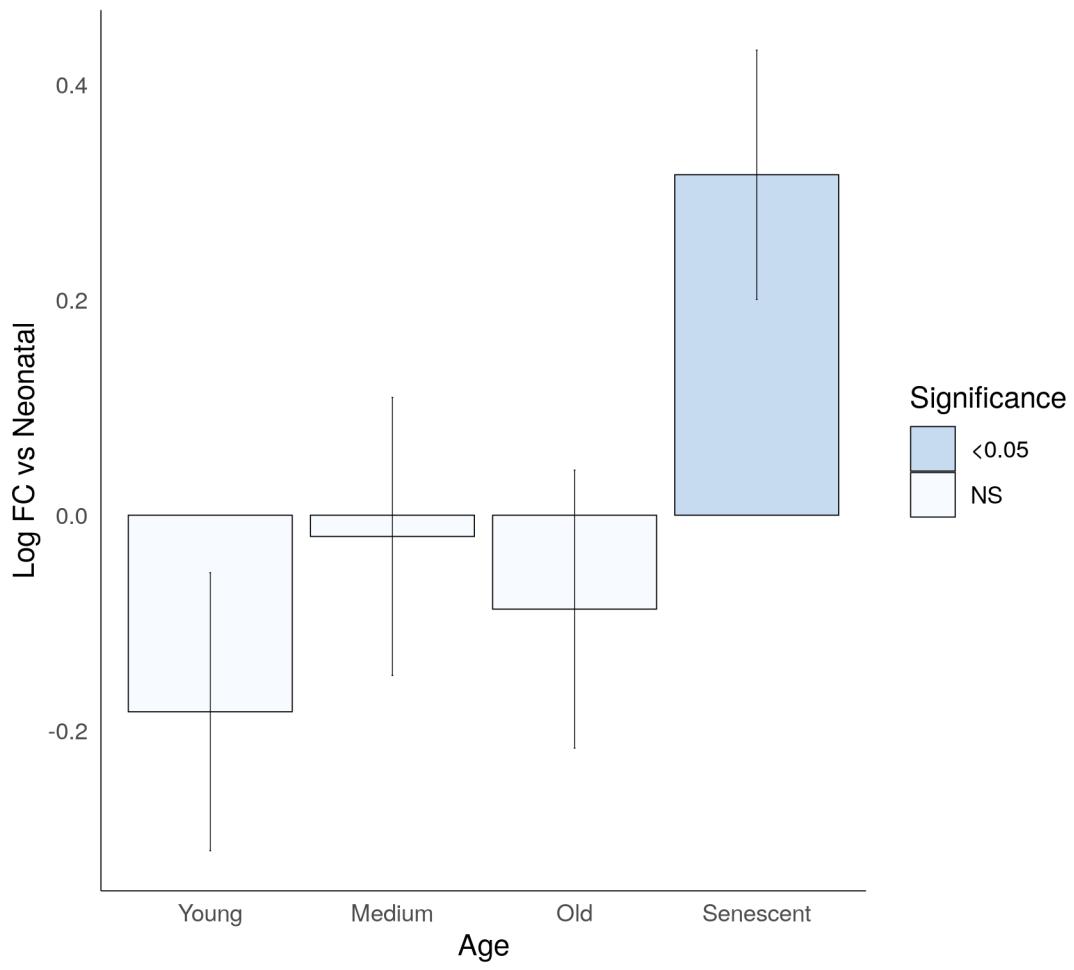

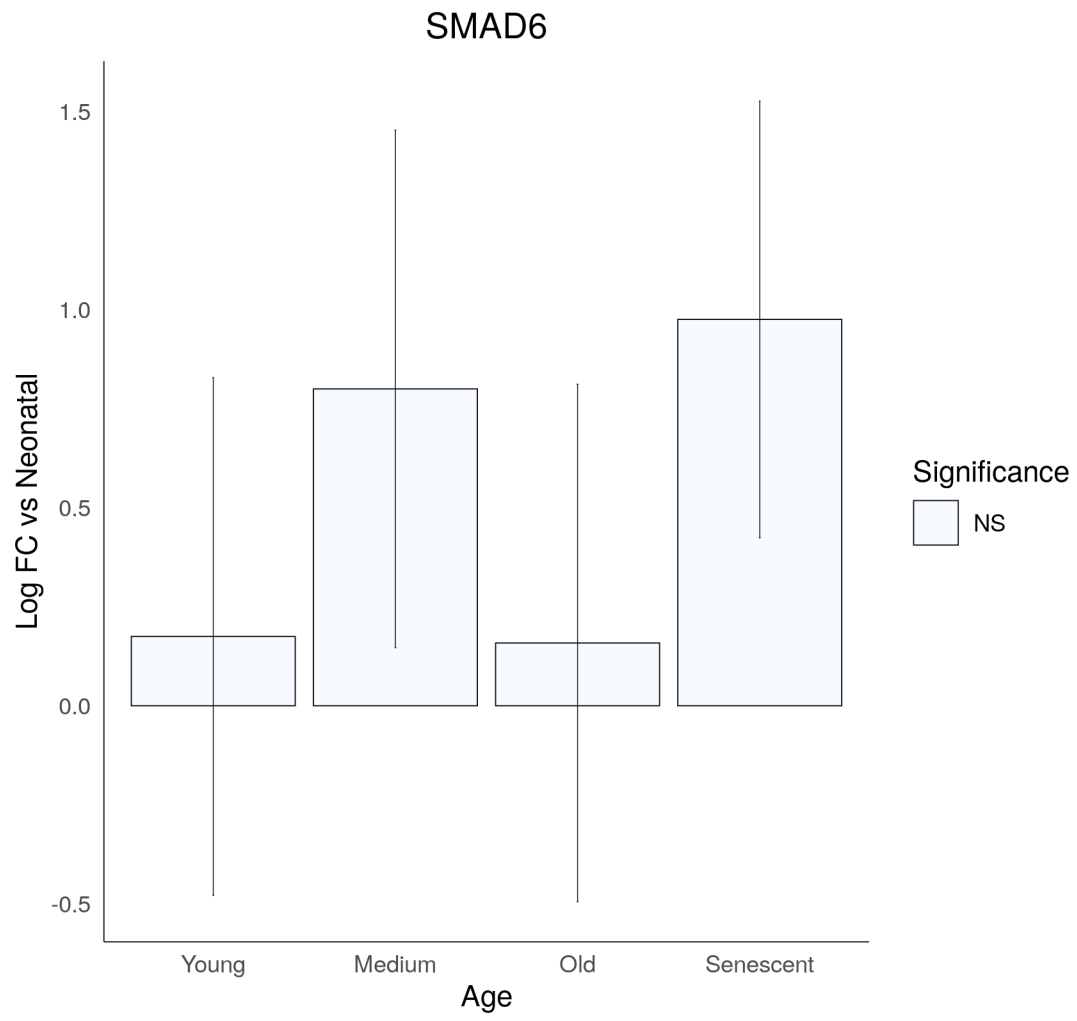

## SMAD7

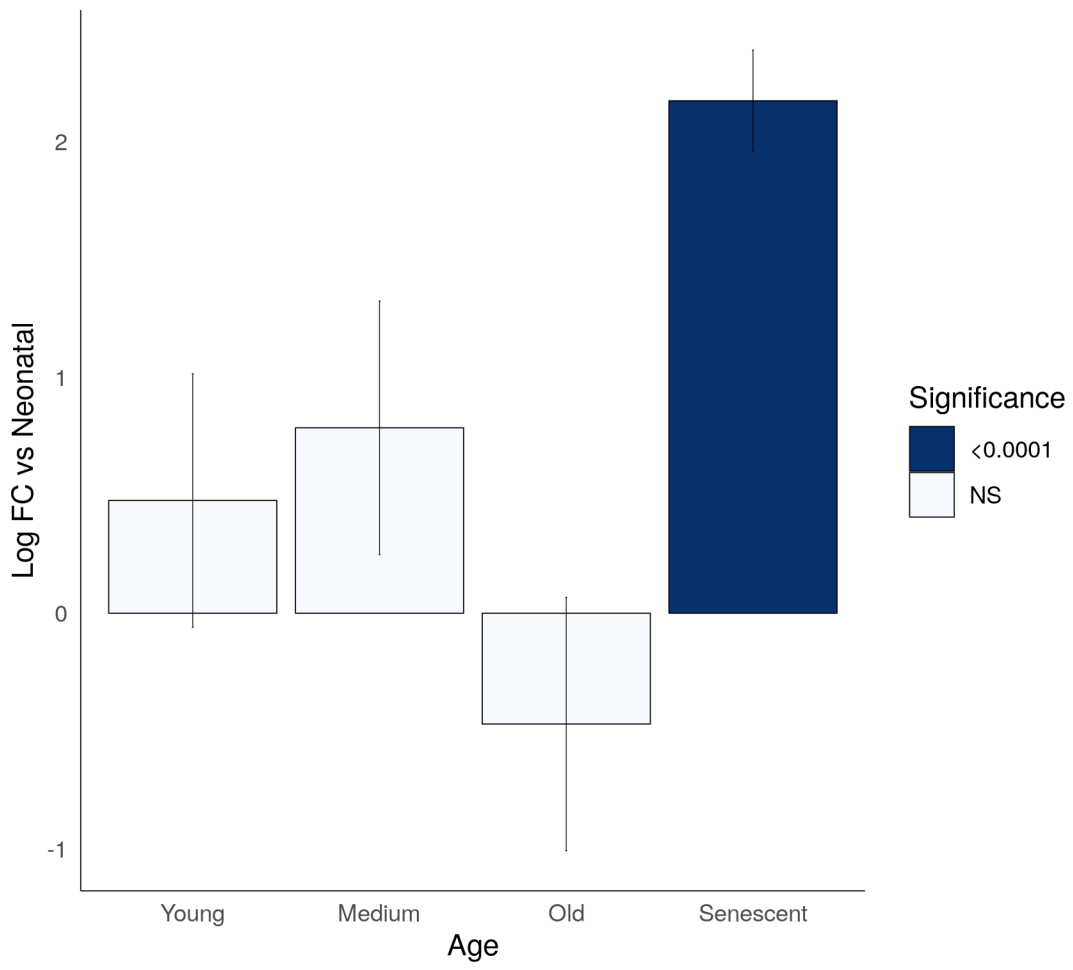

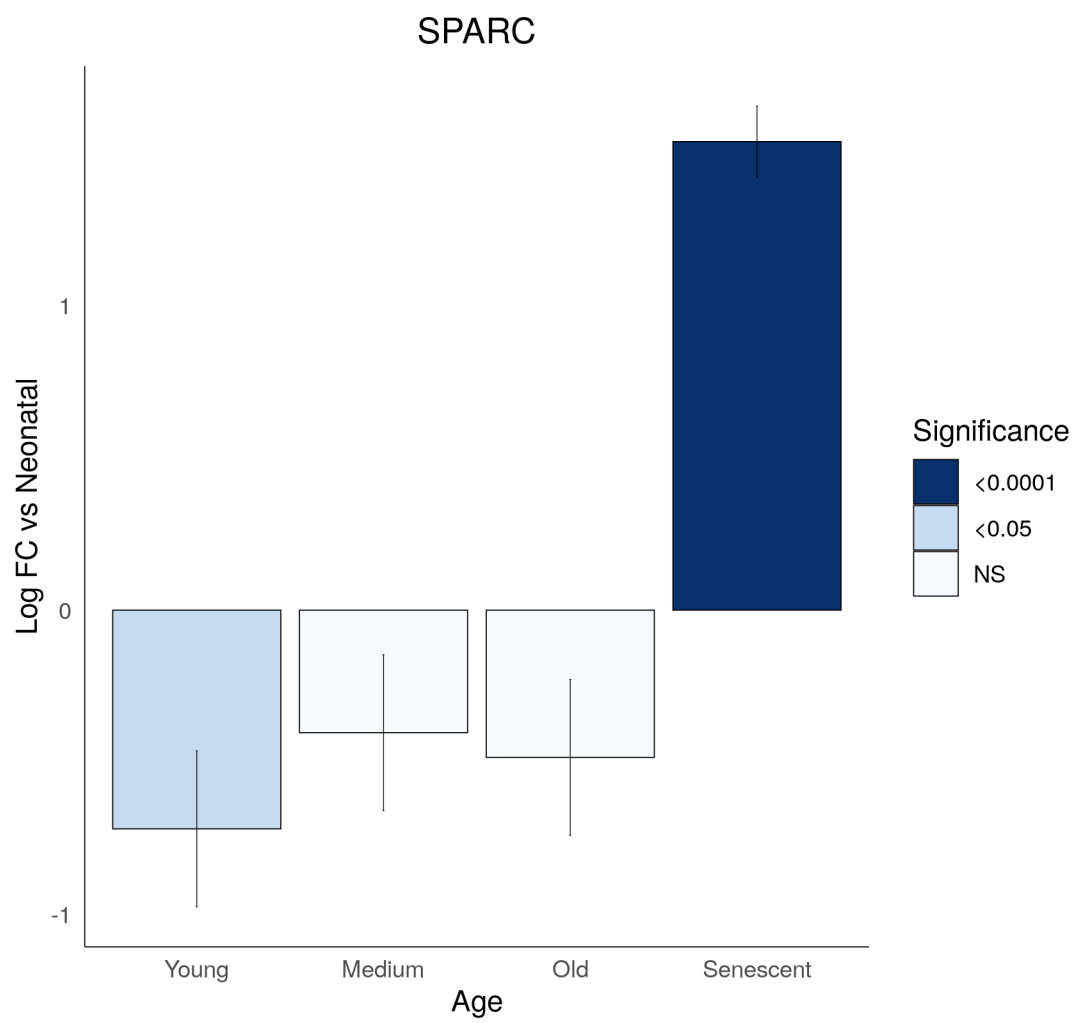

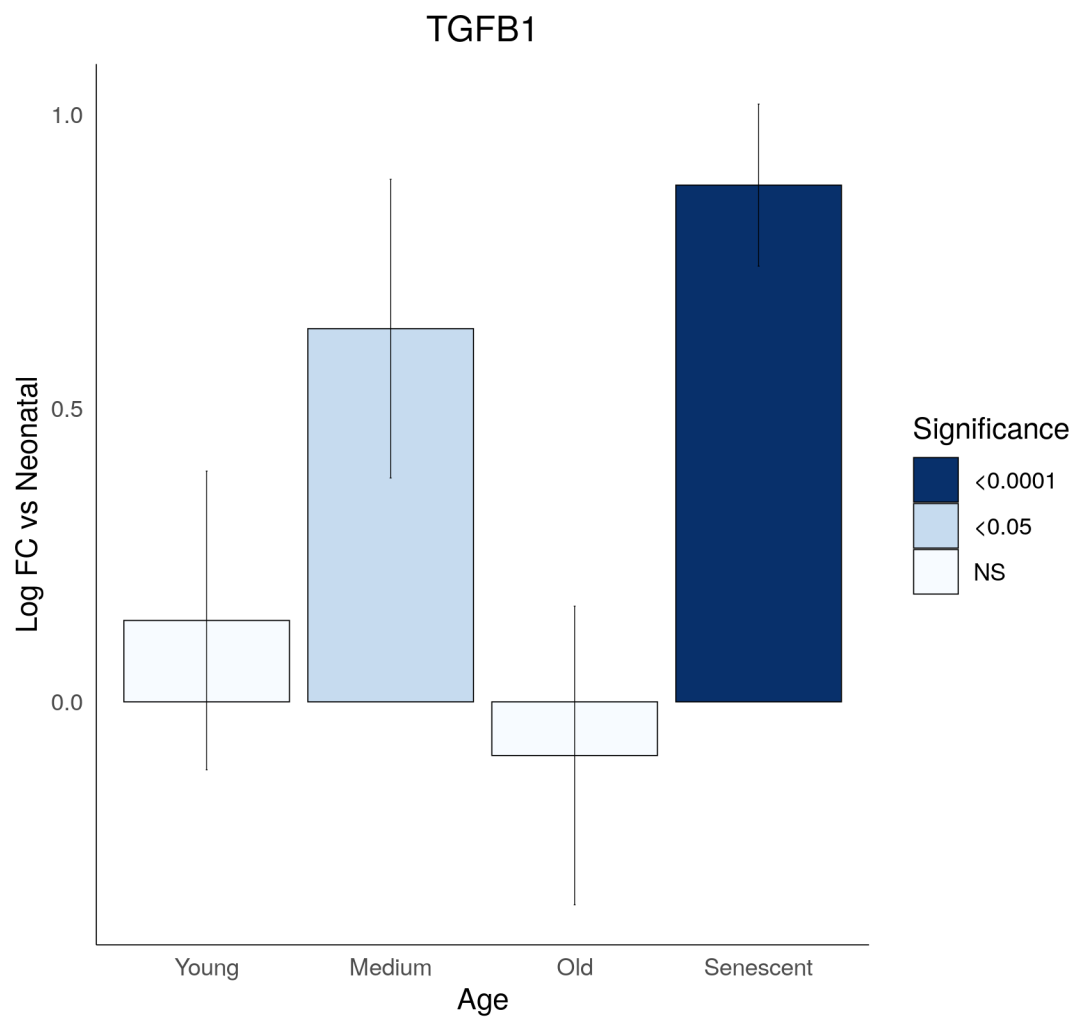

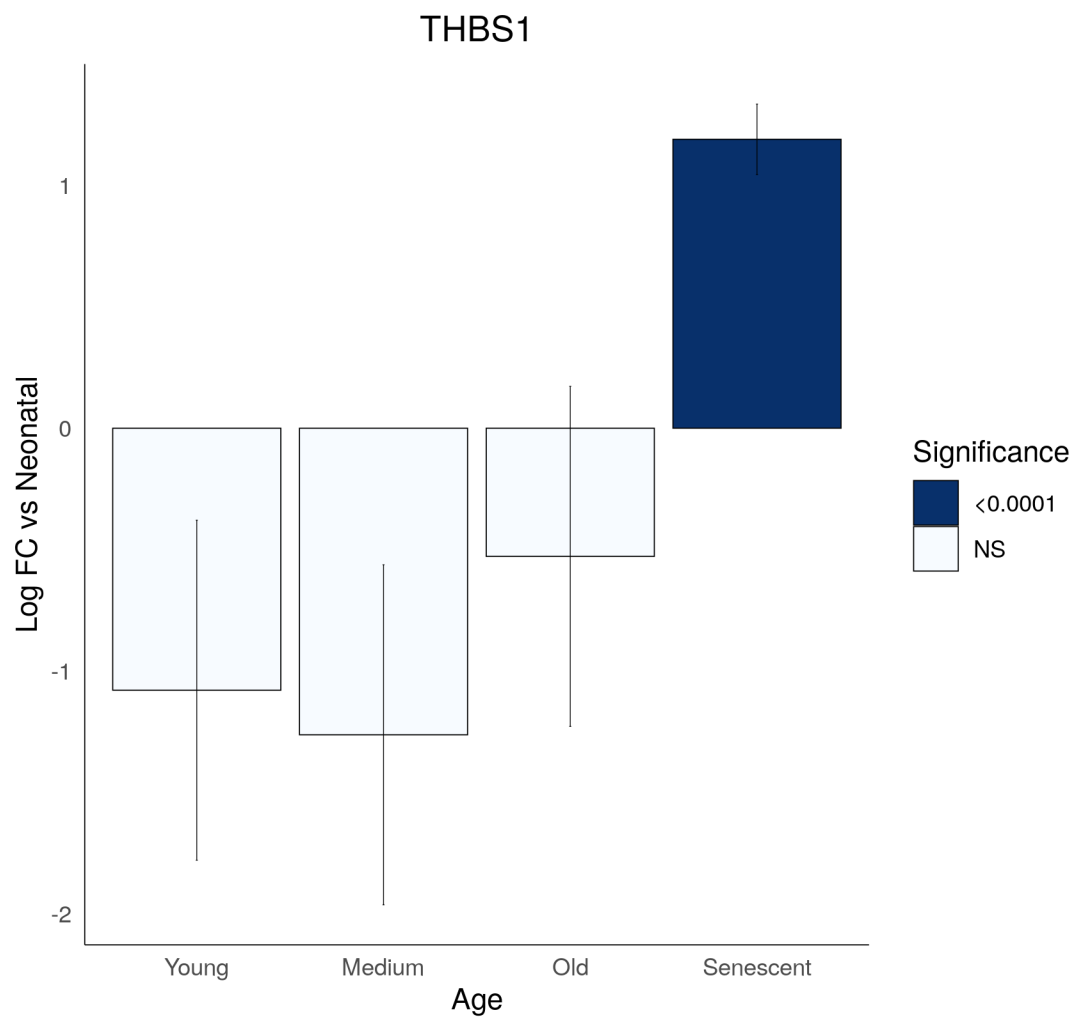

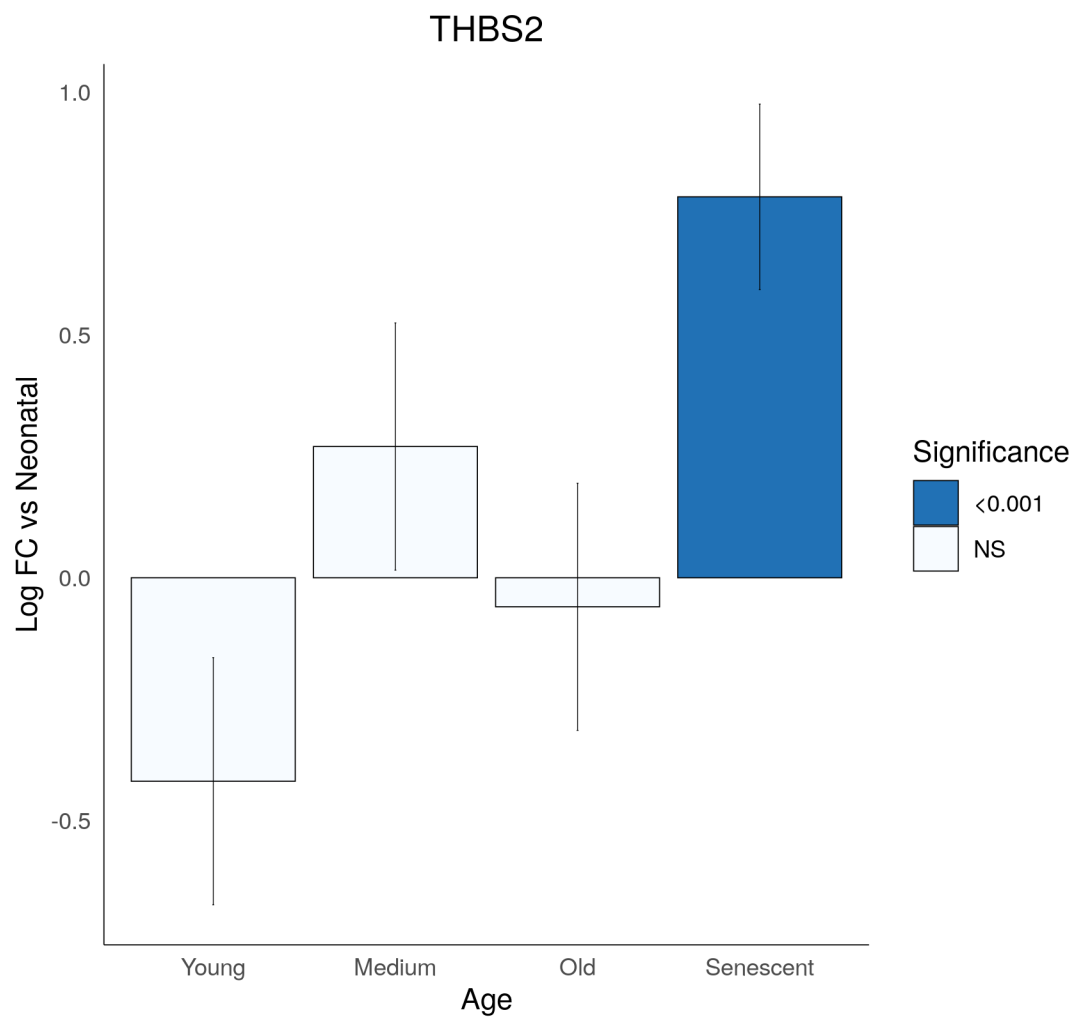

# TIMP1

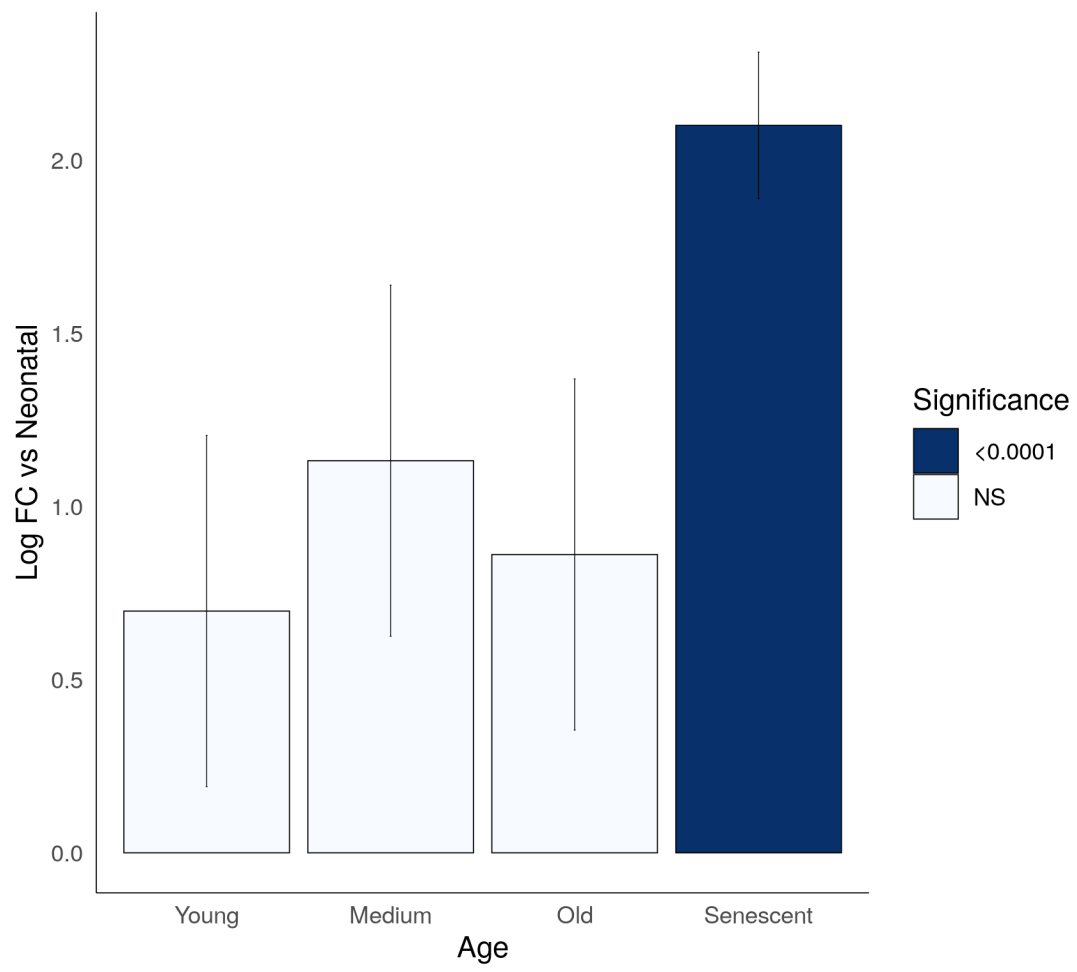

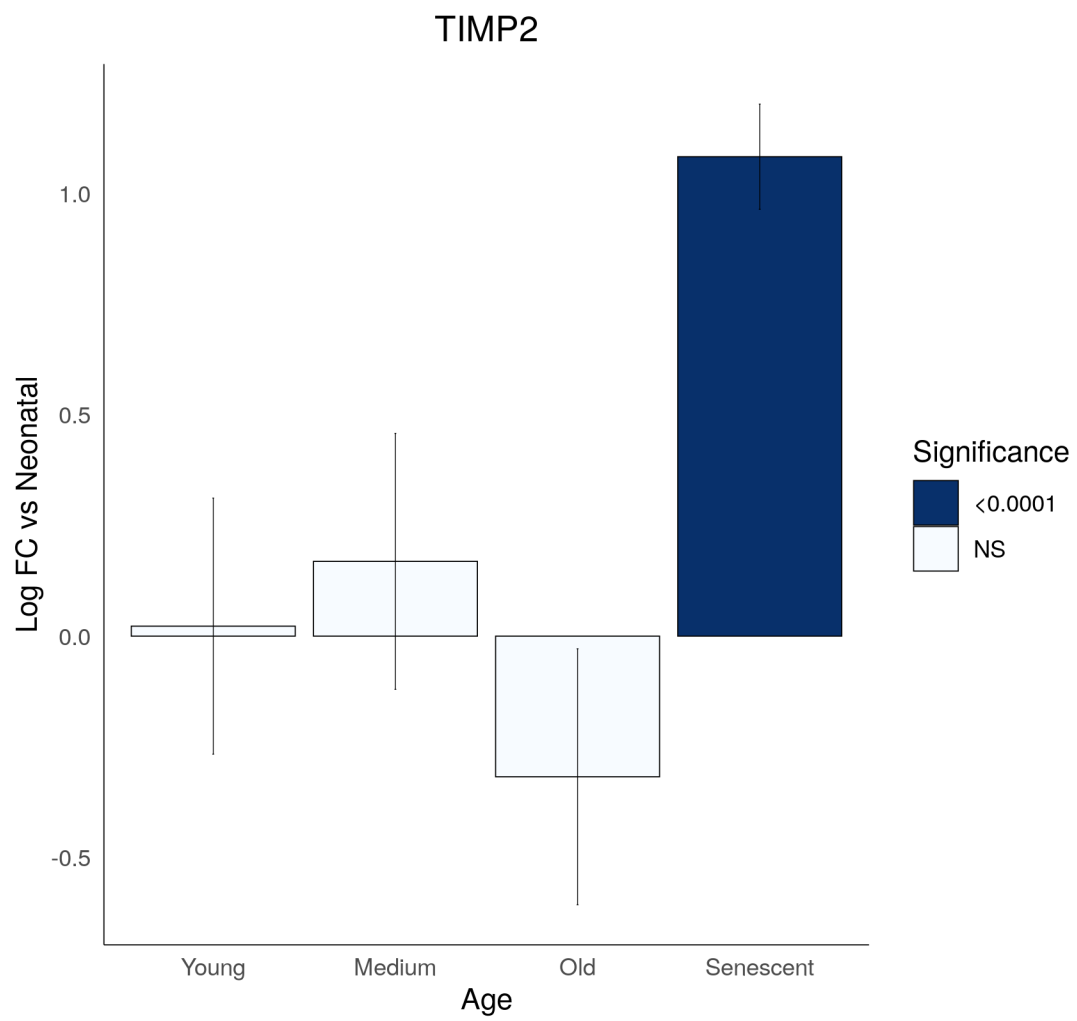

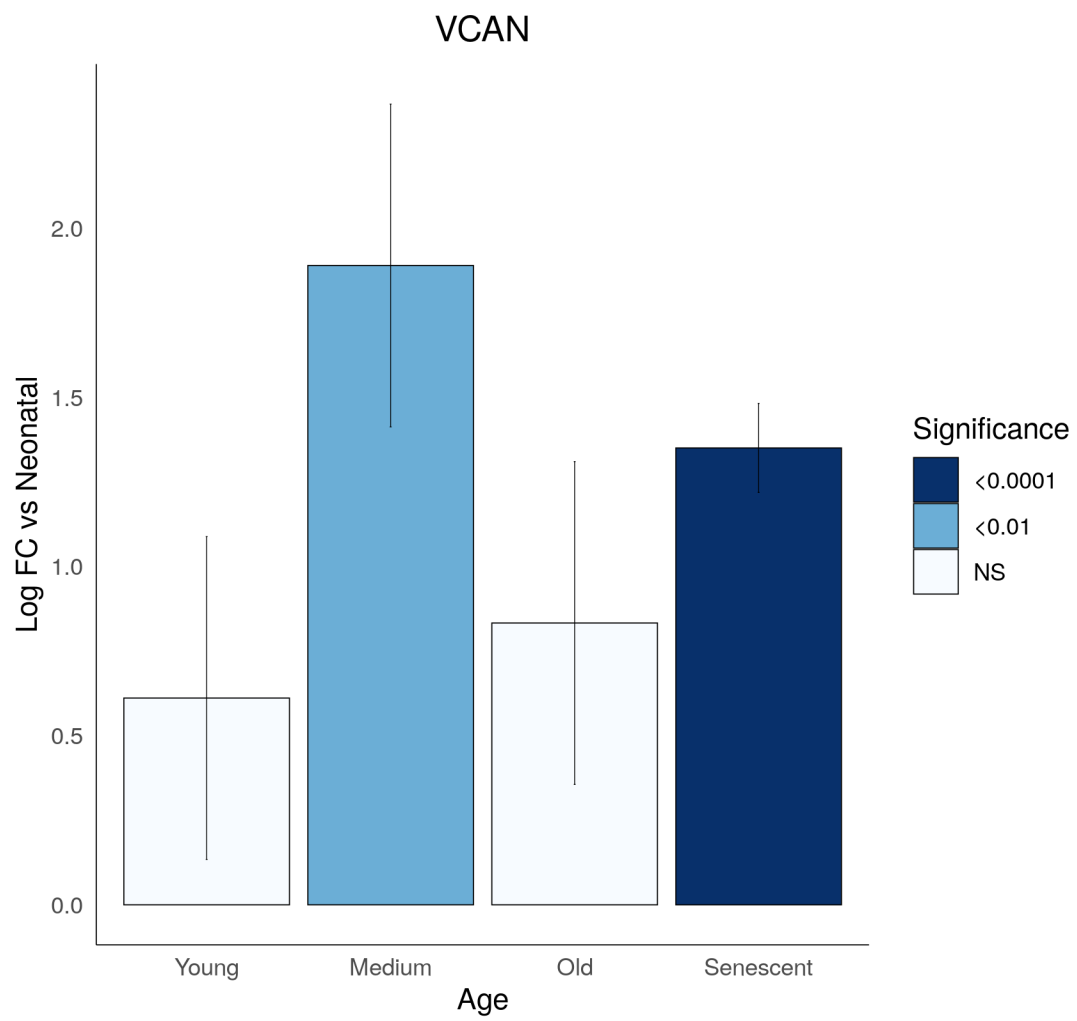

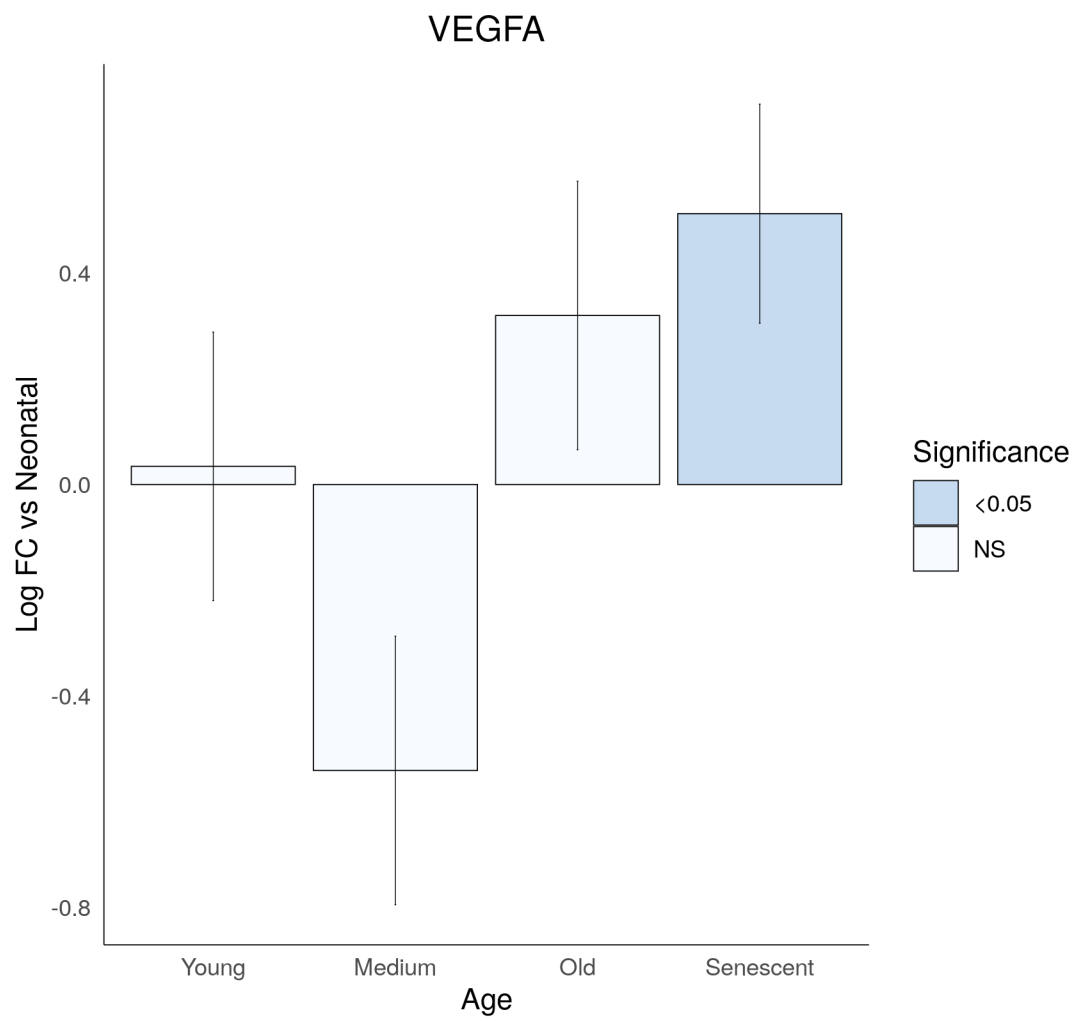

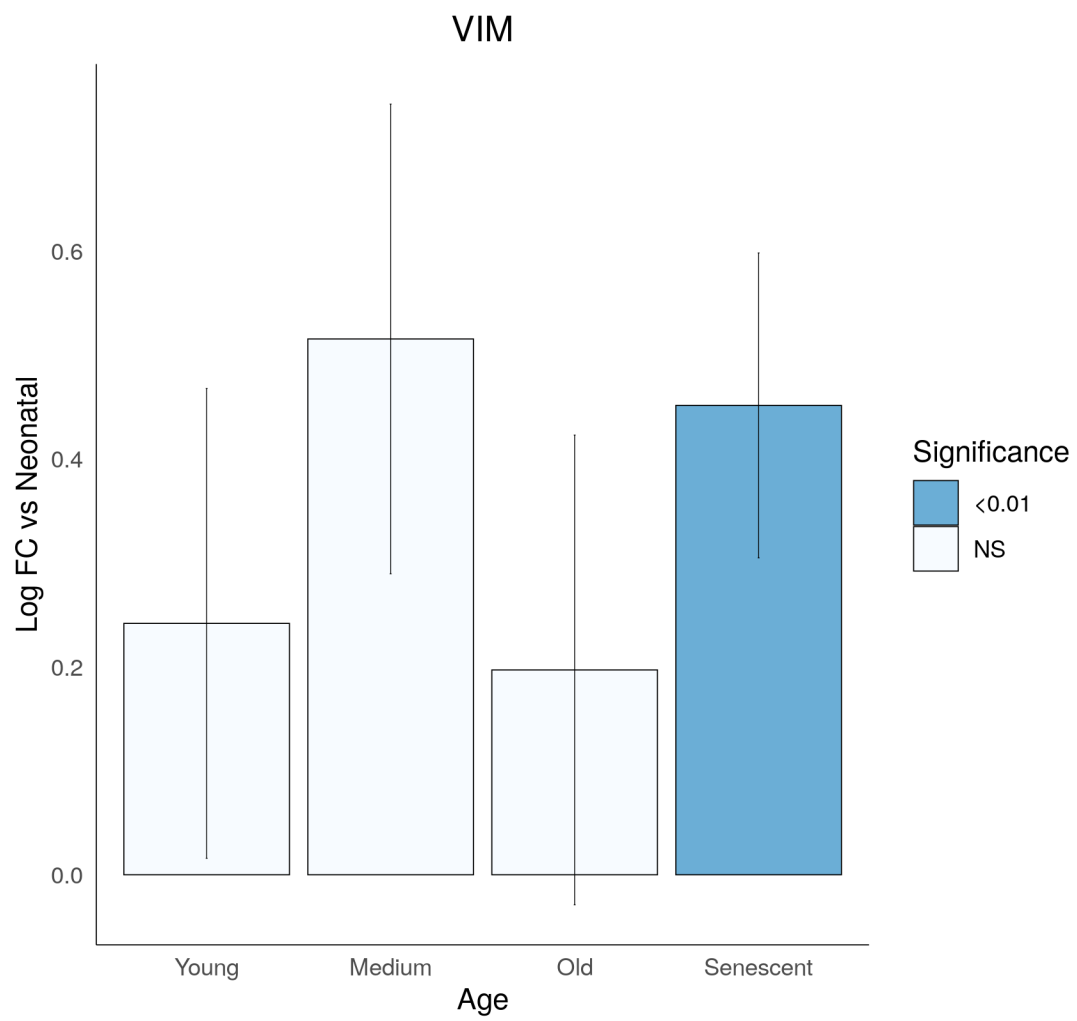

Supplement: Supplementary file 1 [file cells-13-00659-s001.zip › Supplementary file 5.pdf]

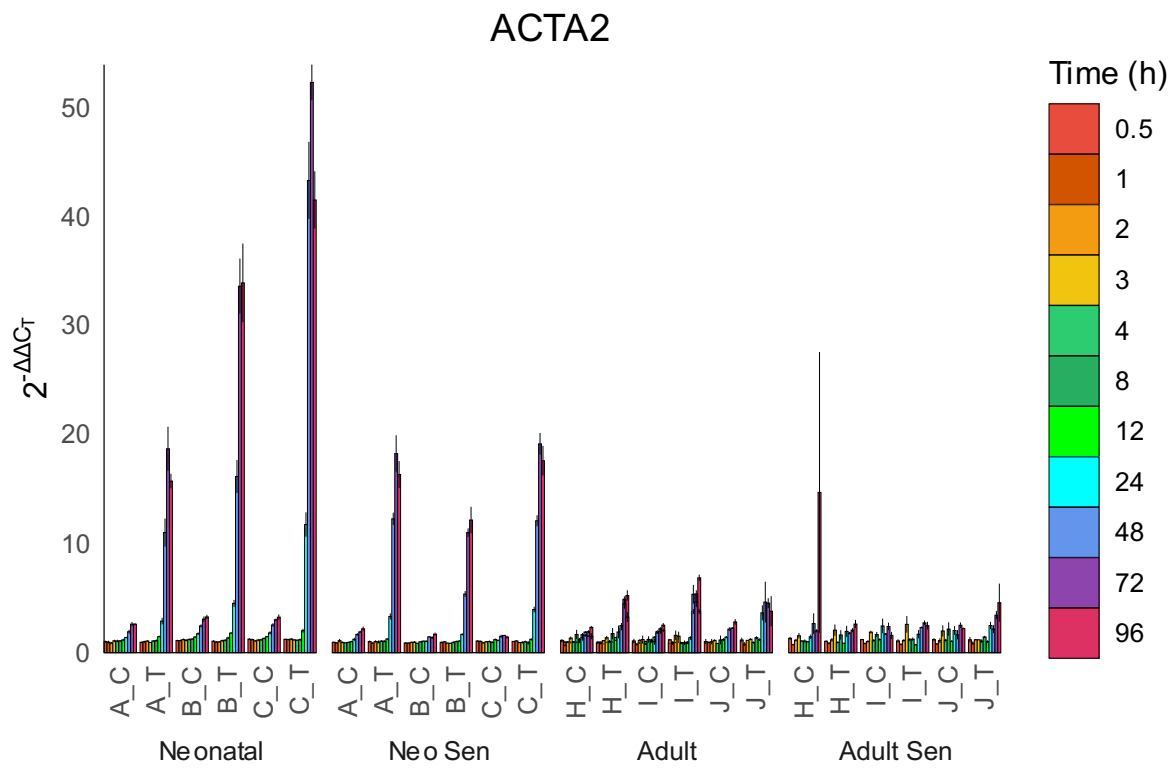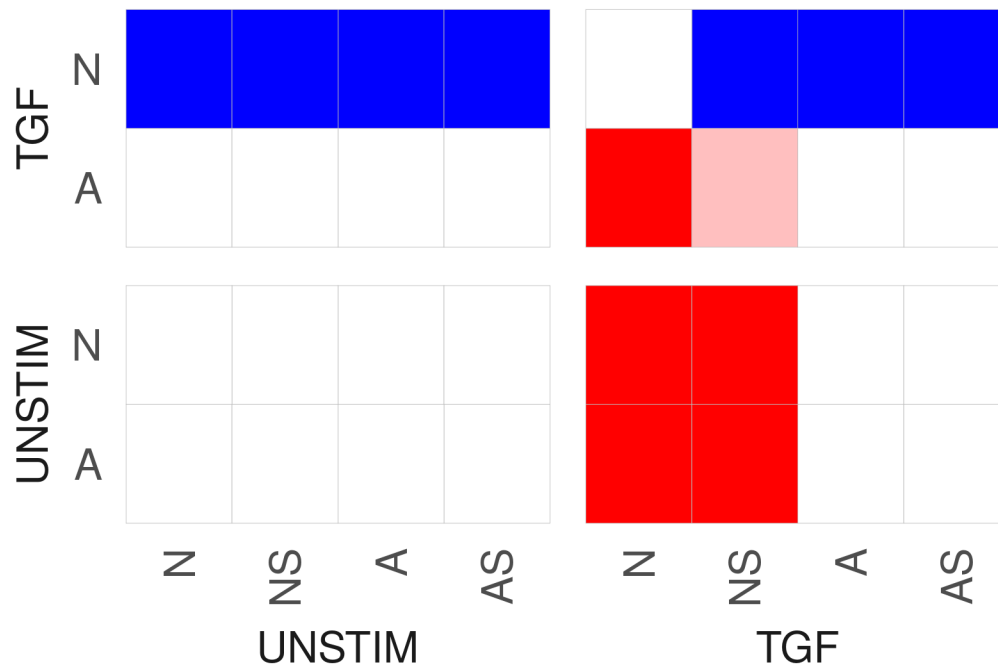

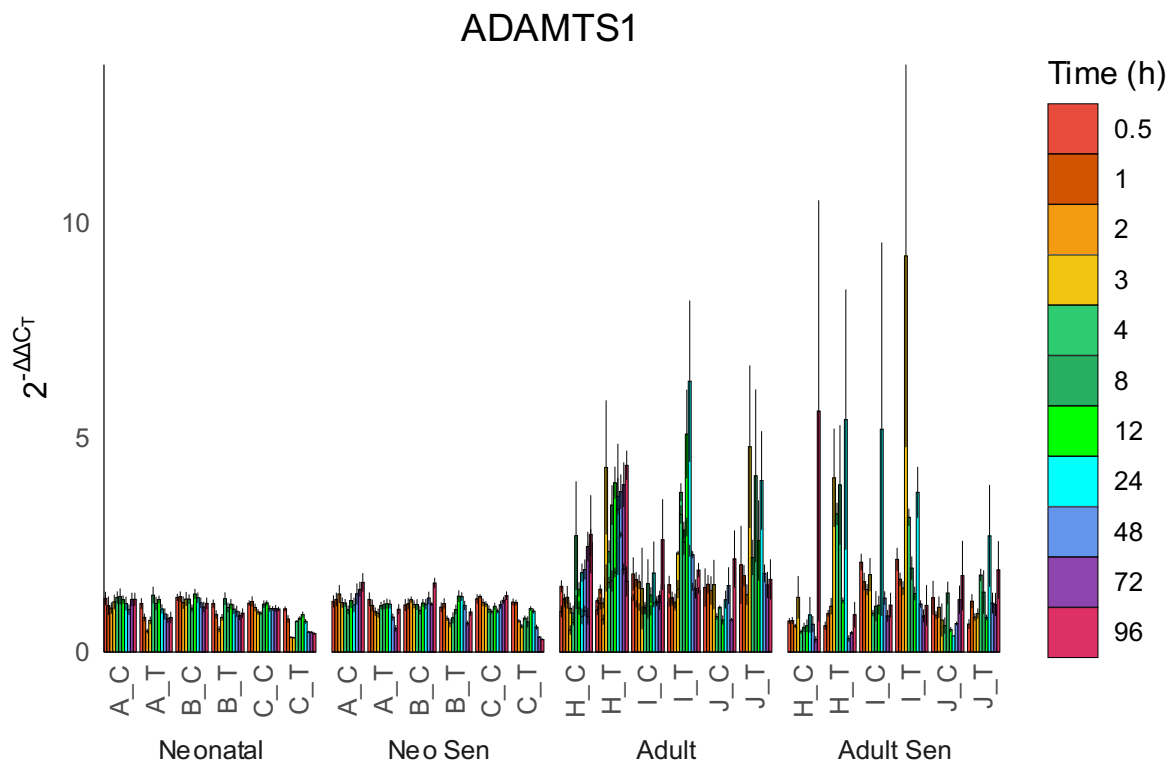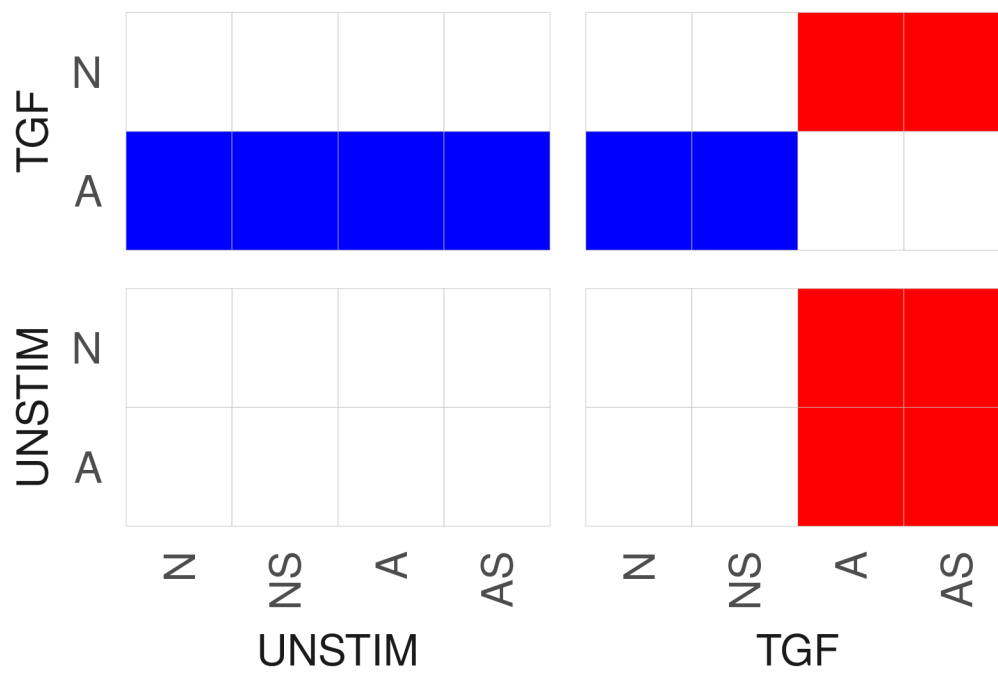

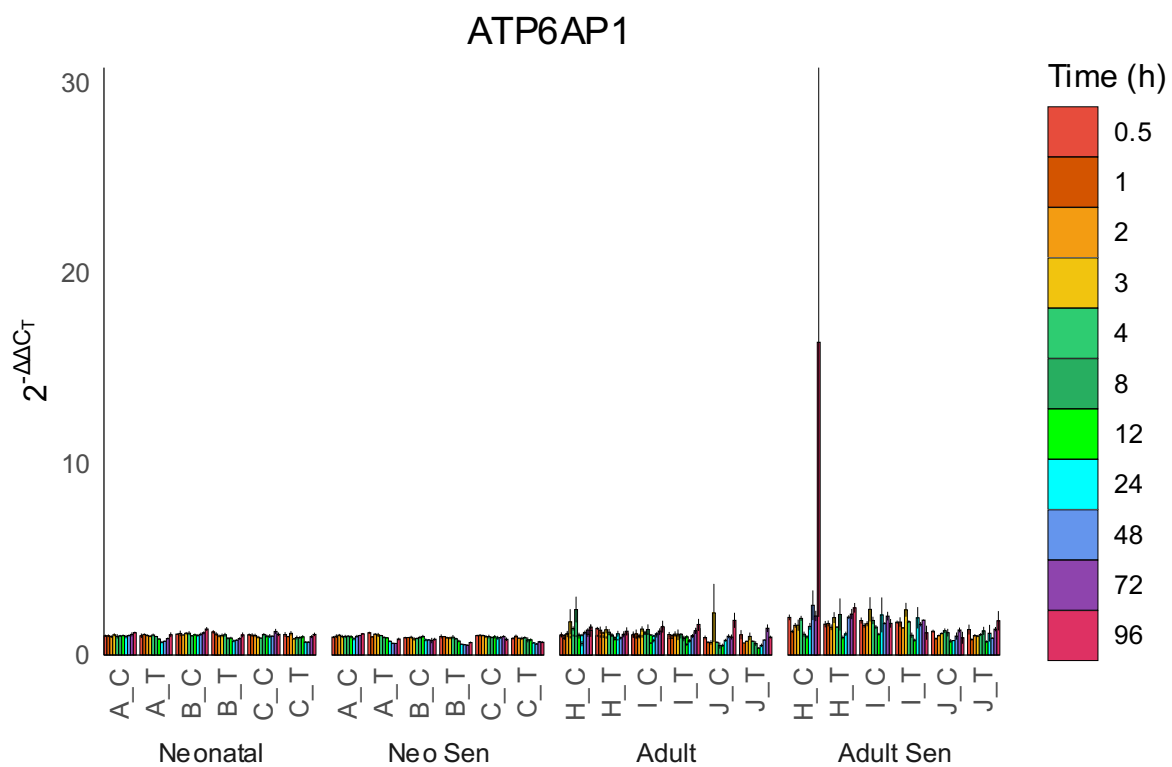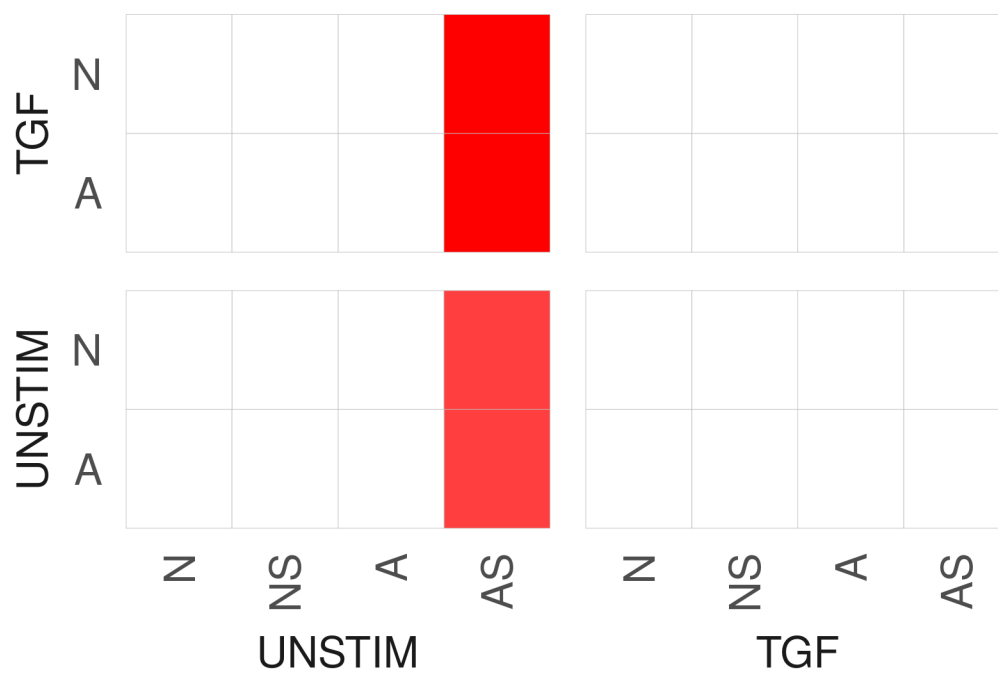

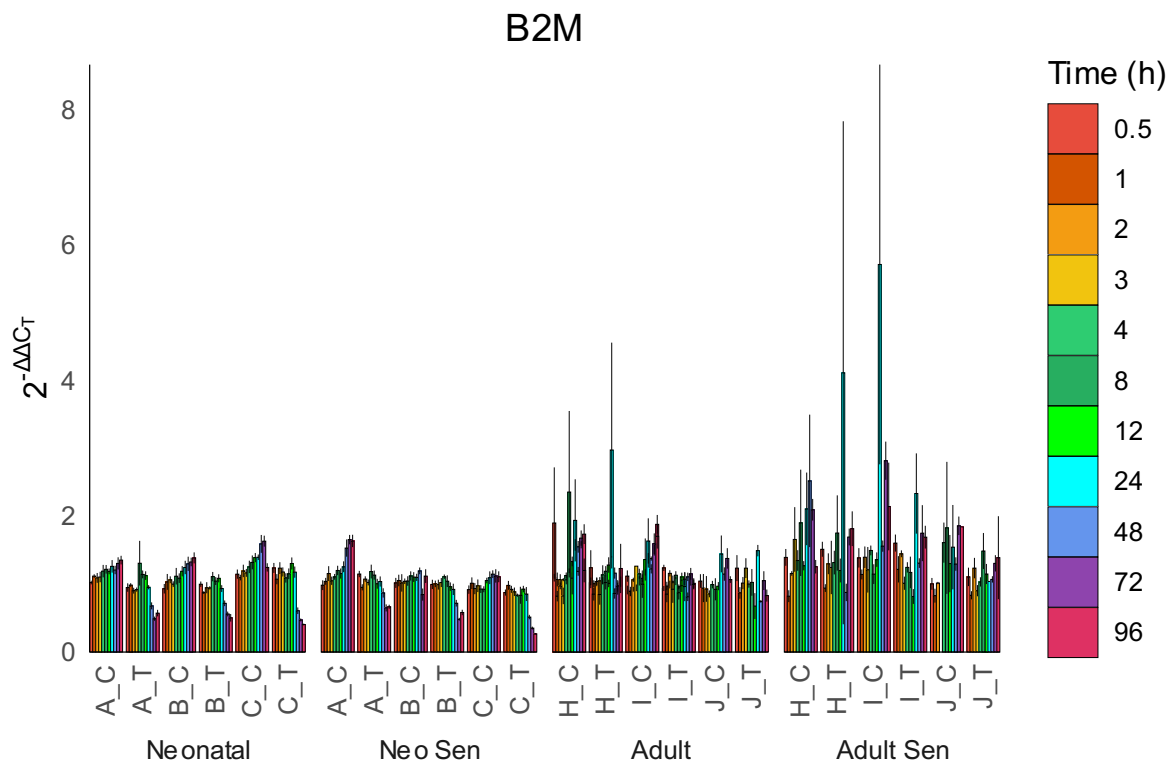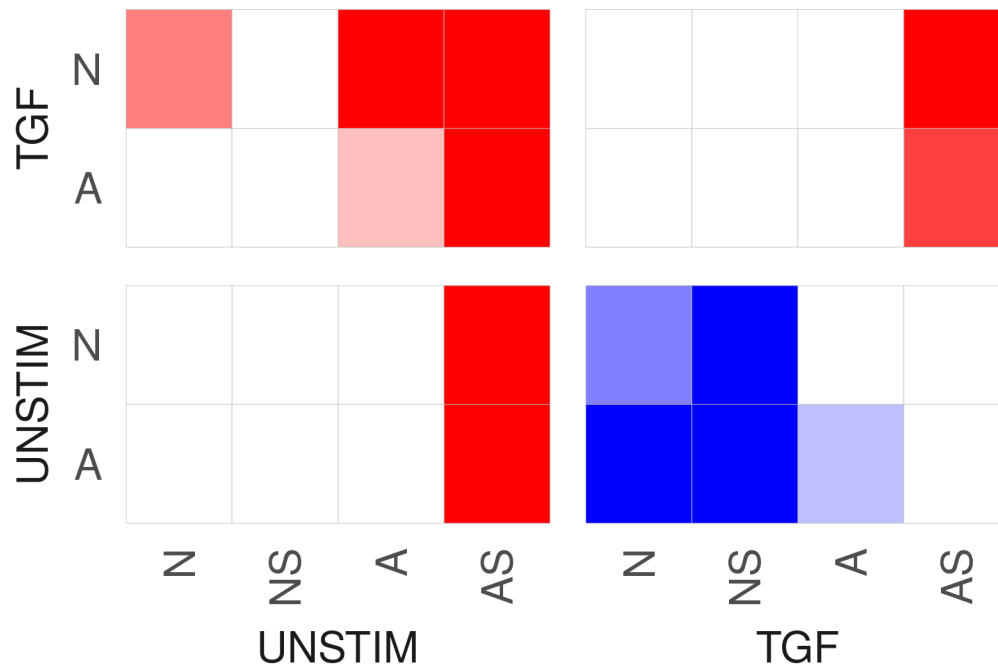

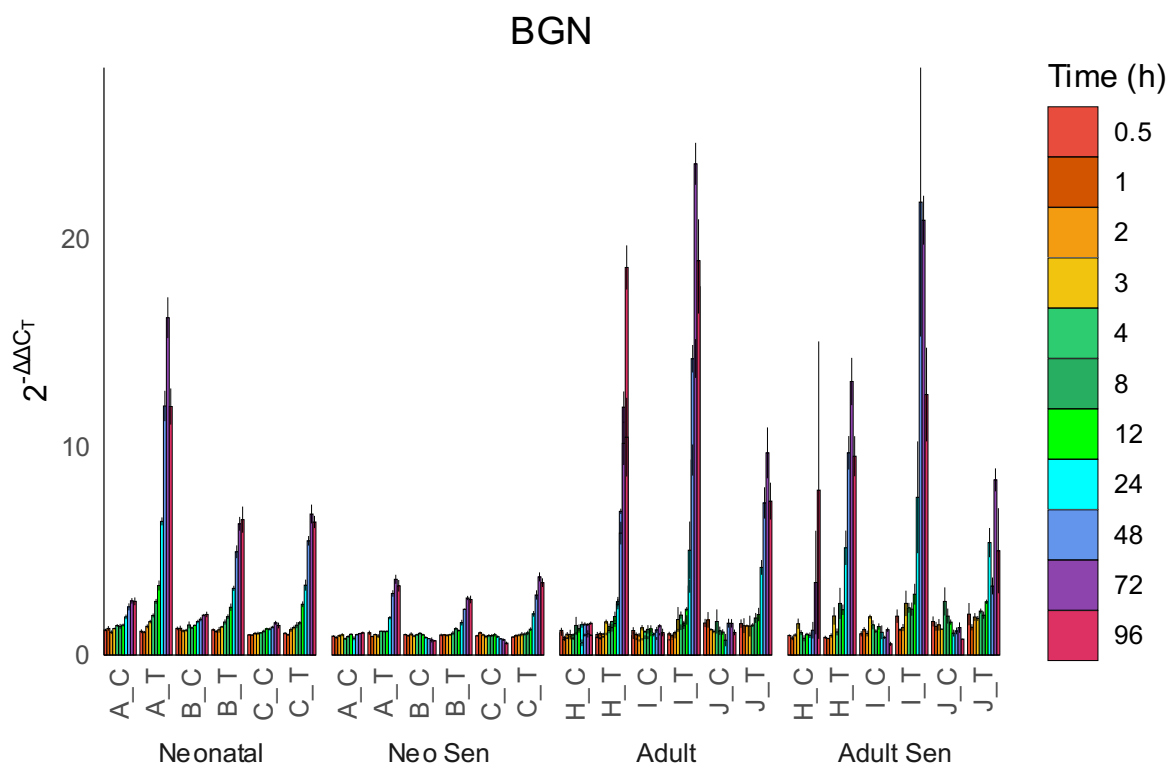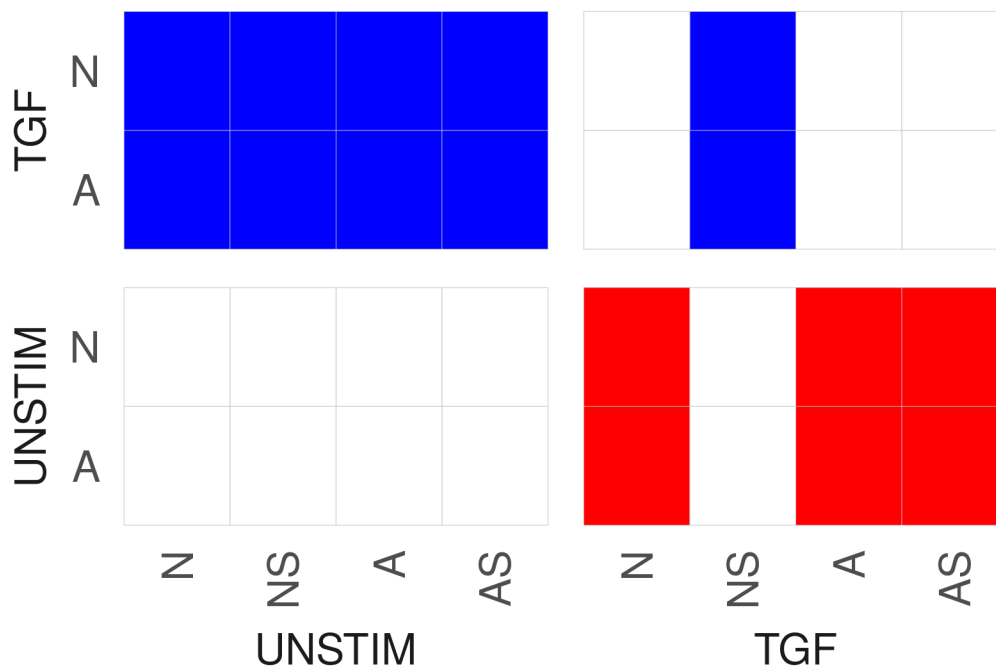

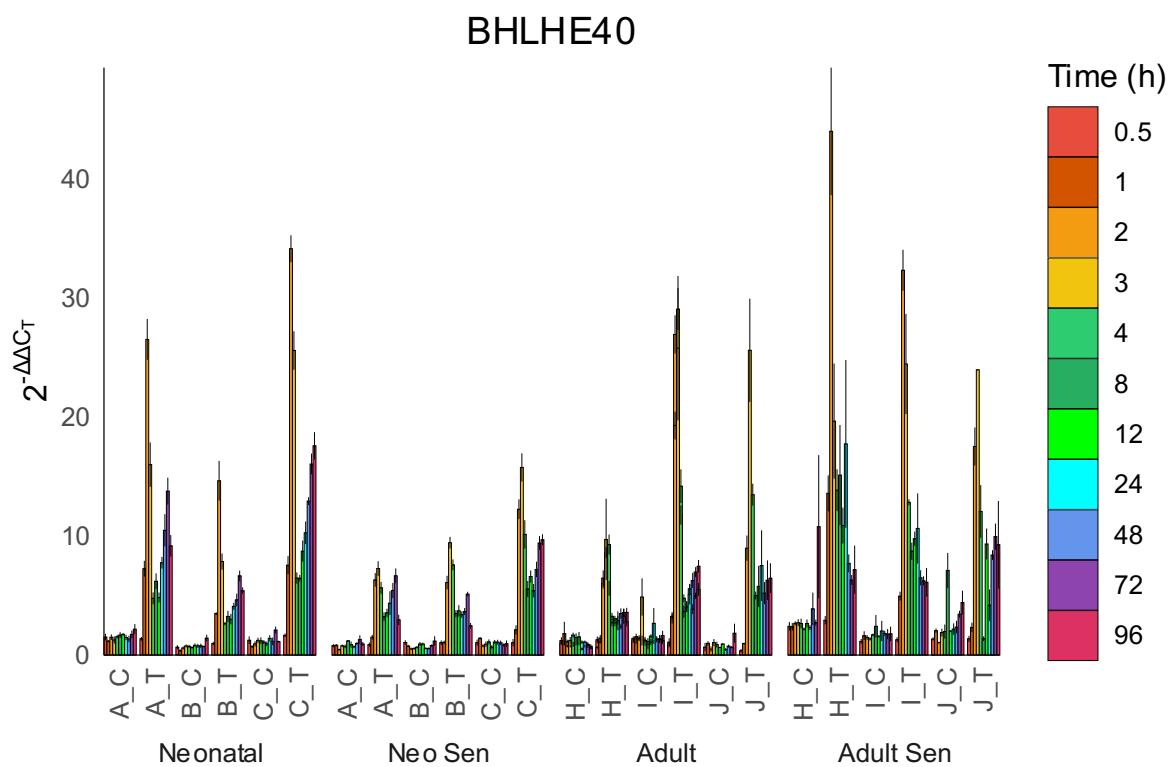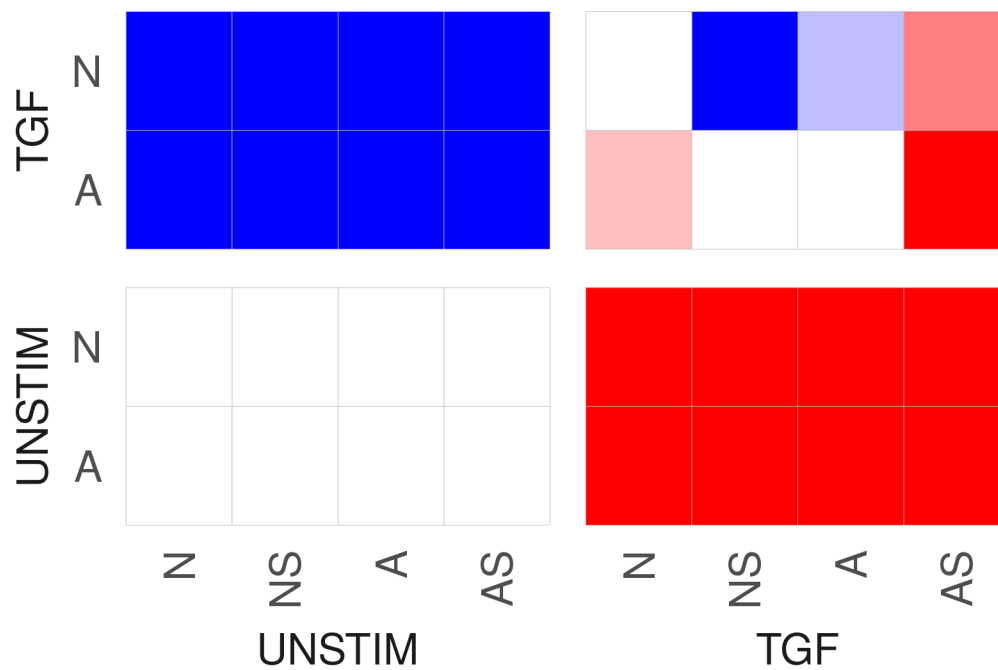

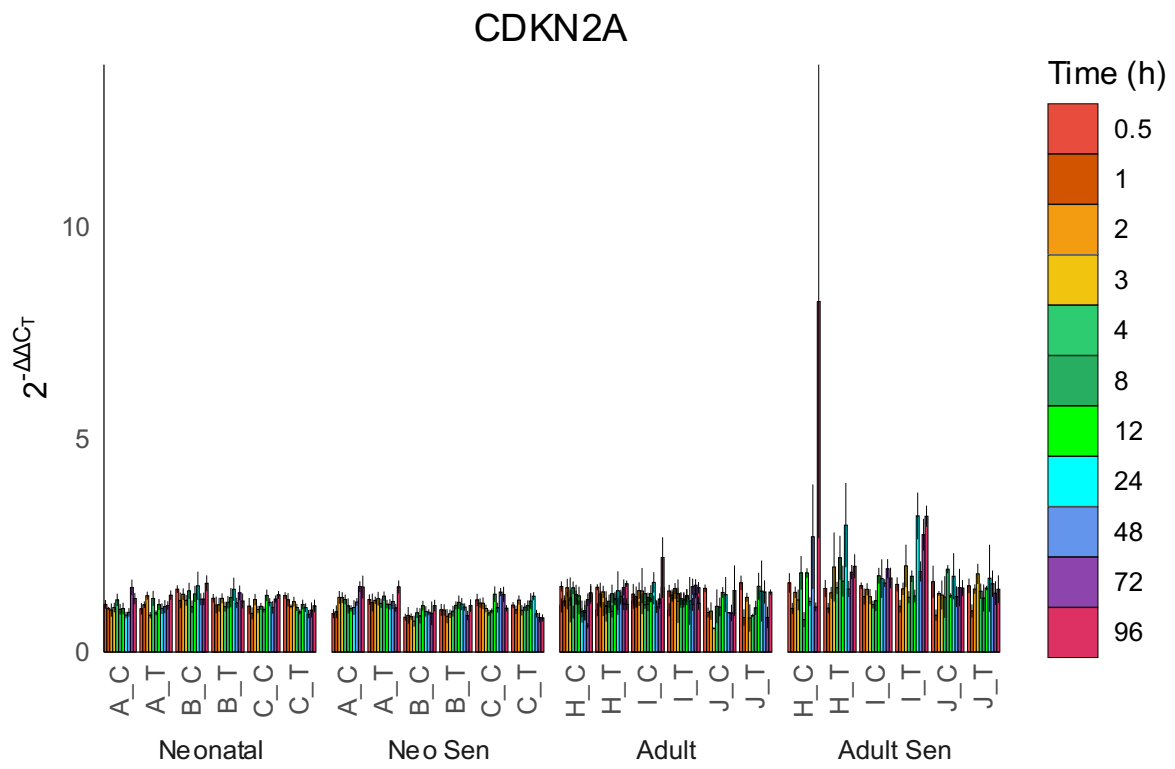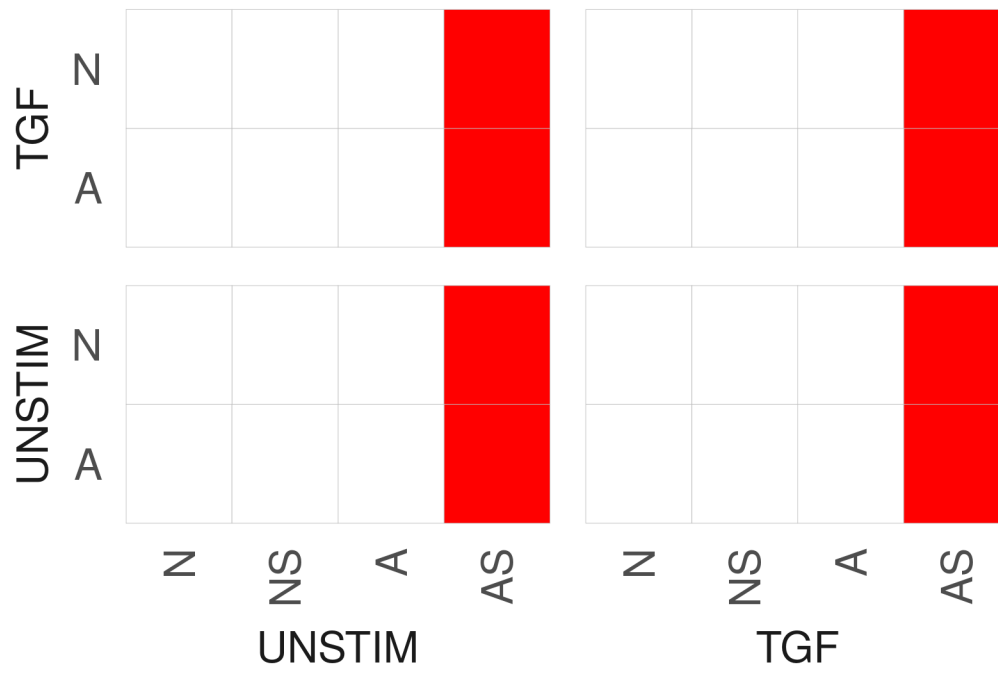

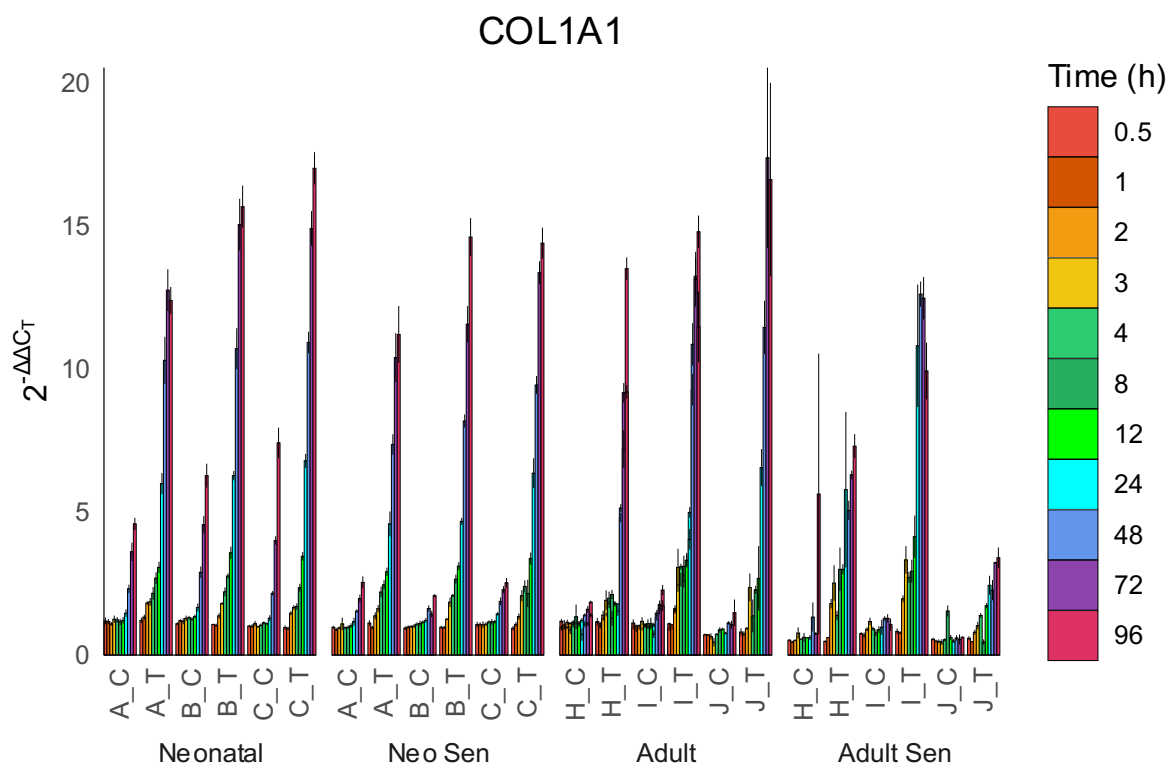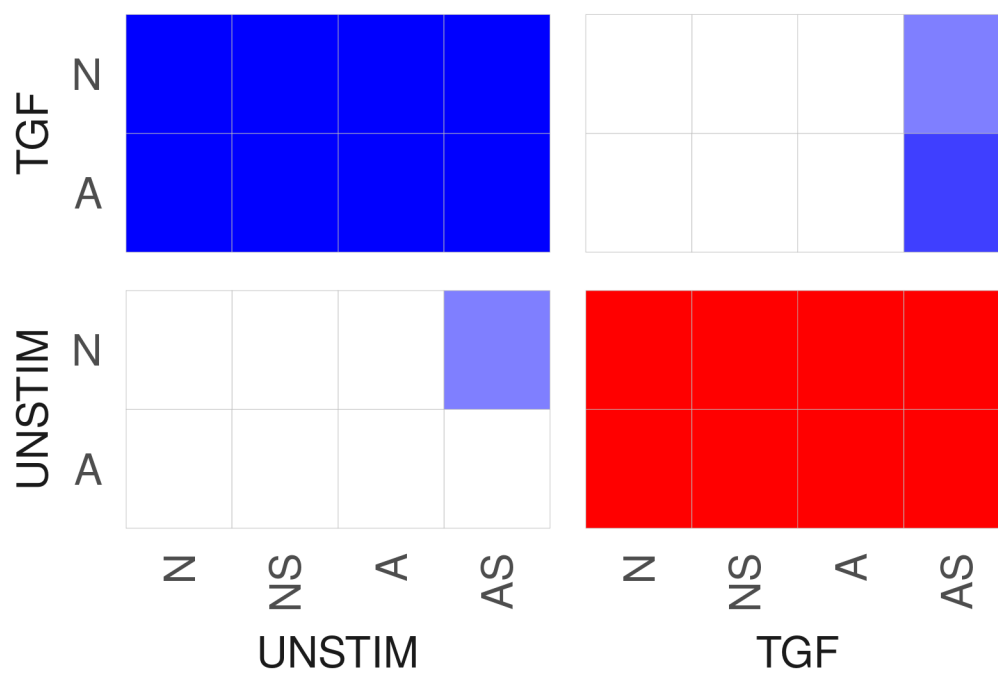

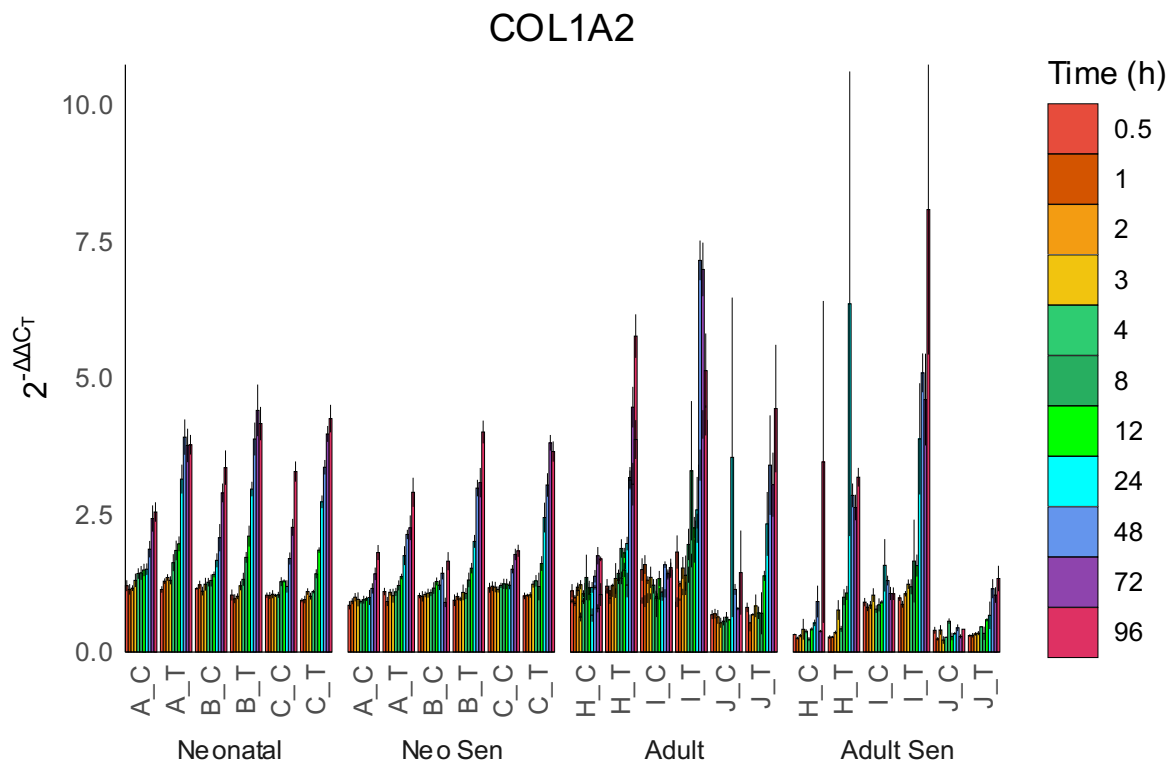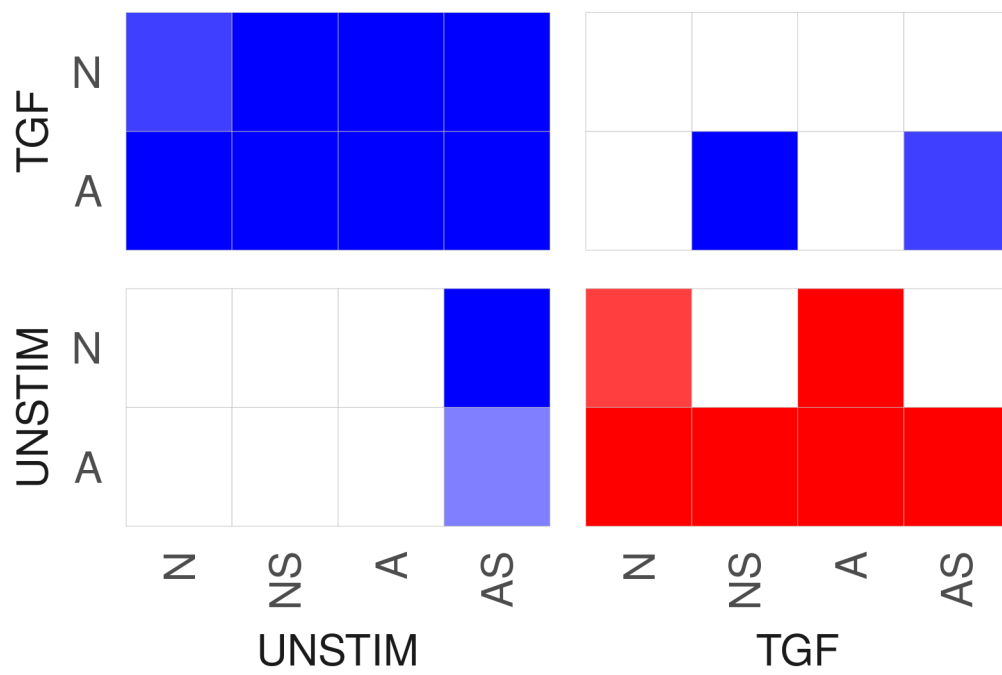

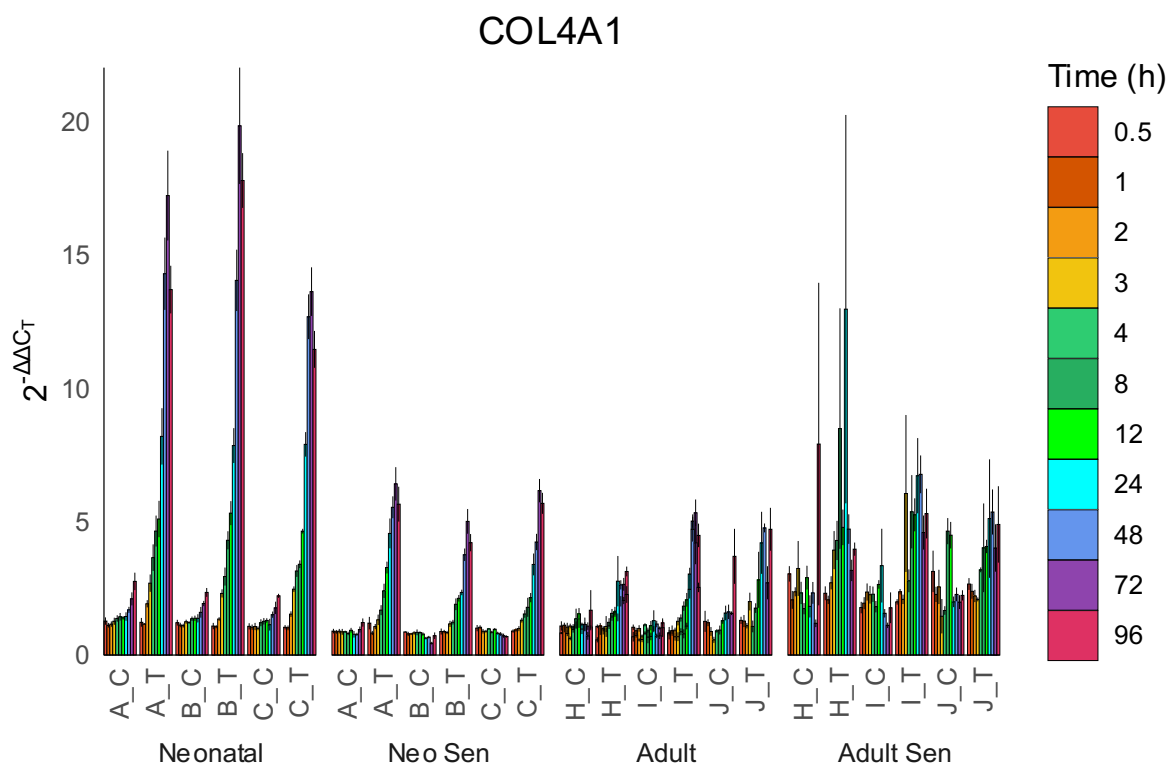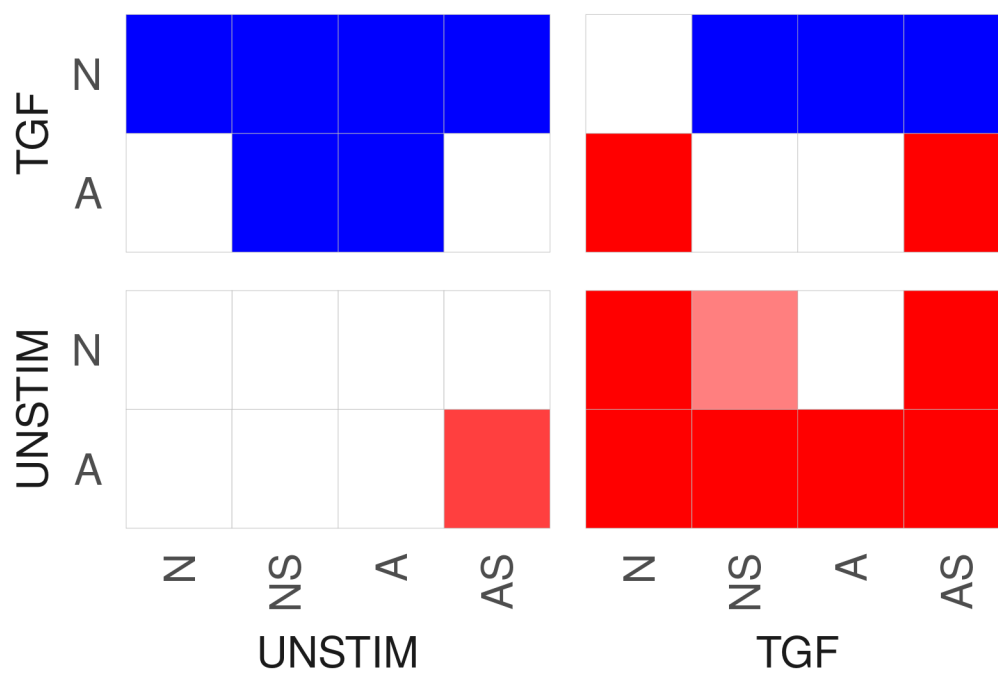

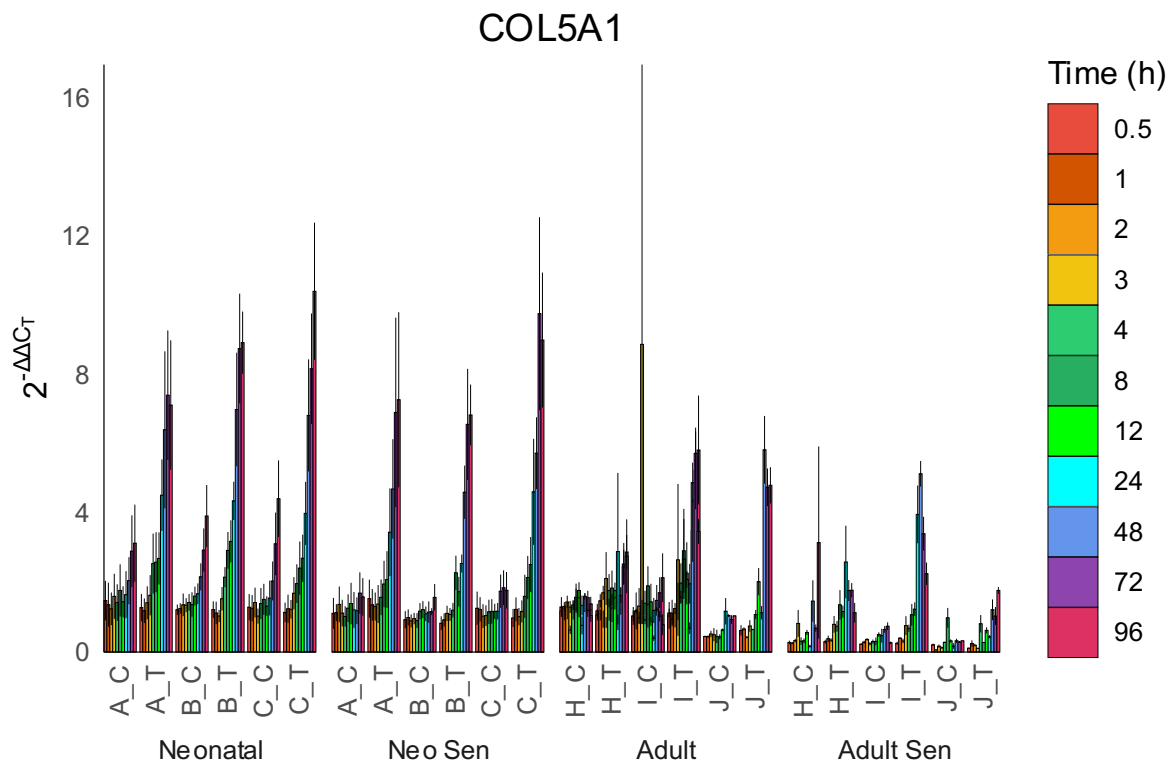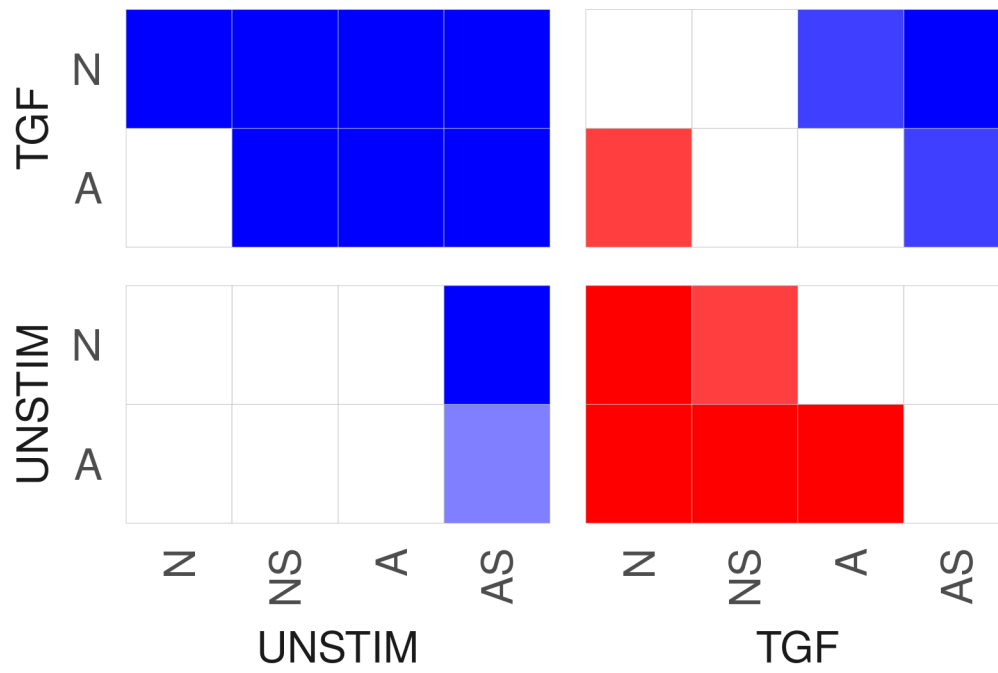

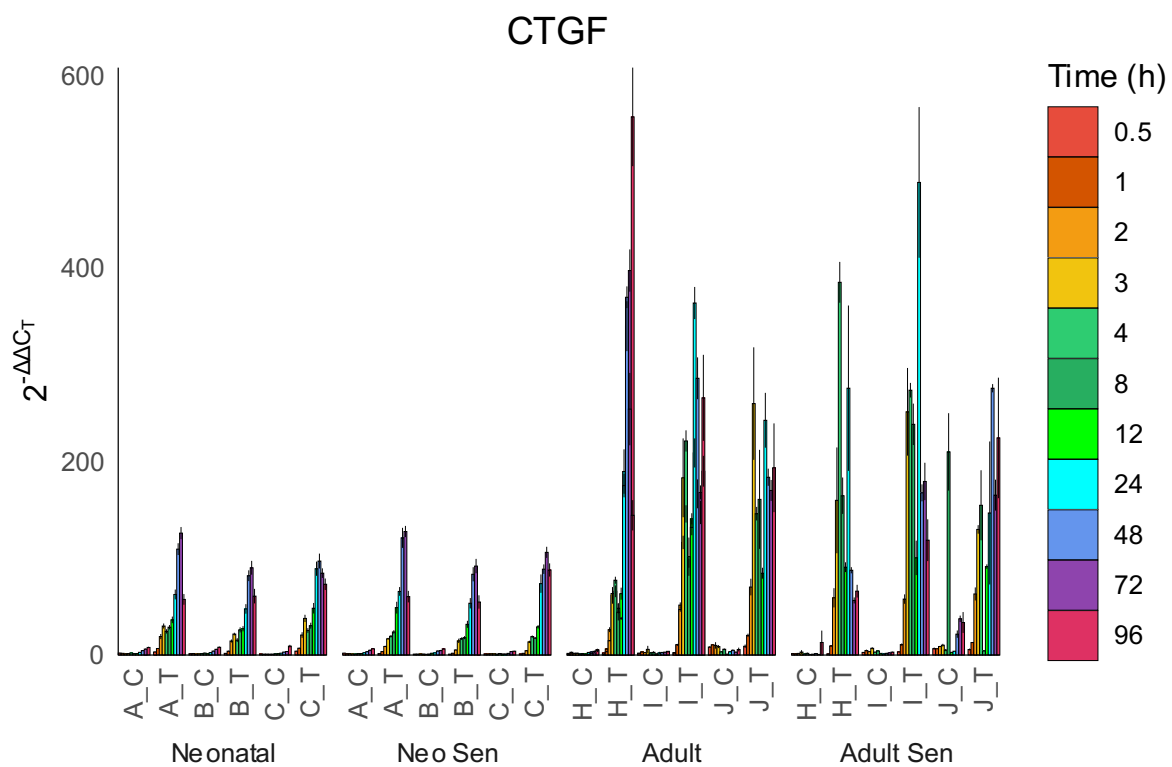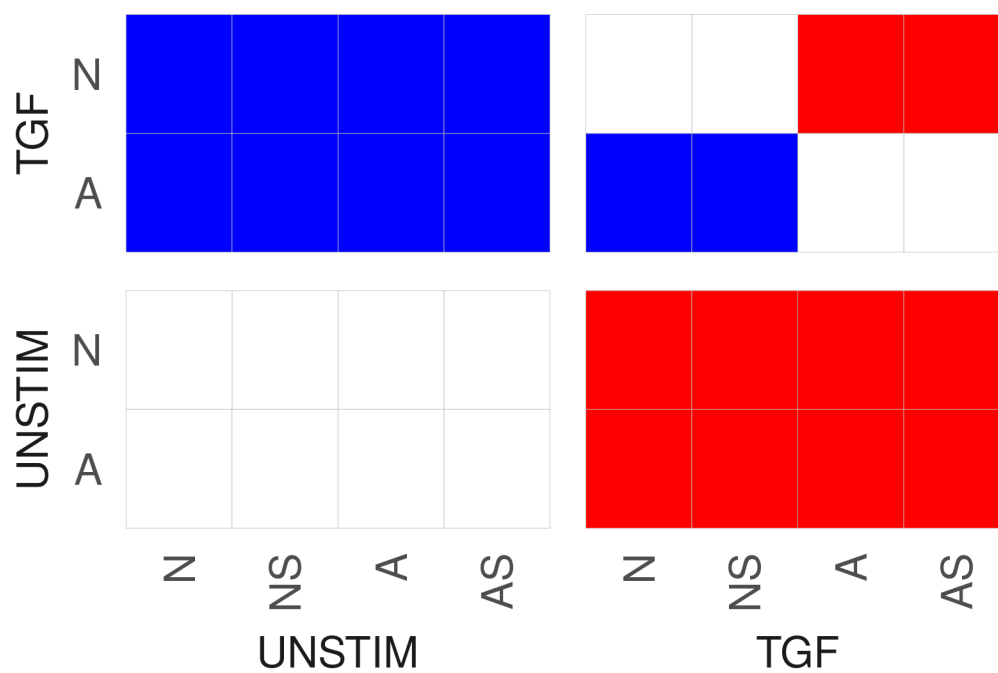

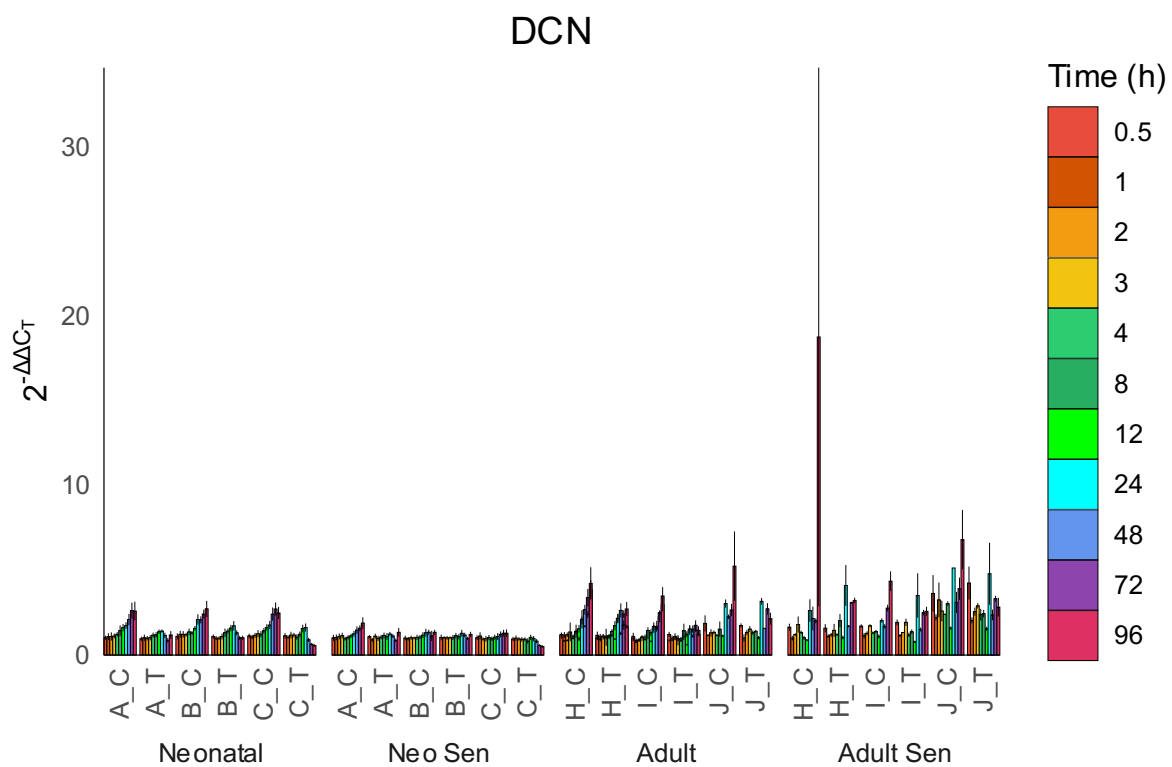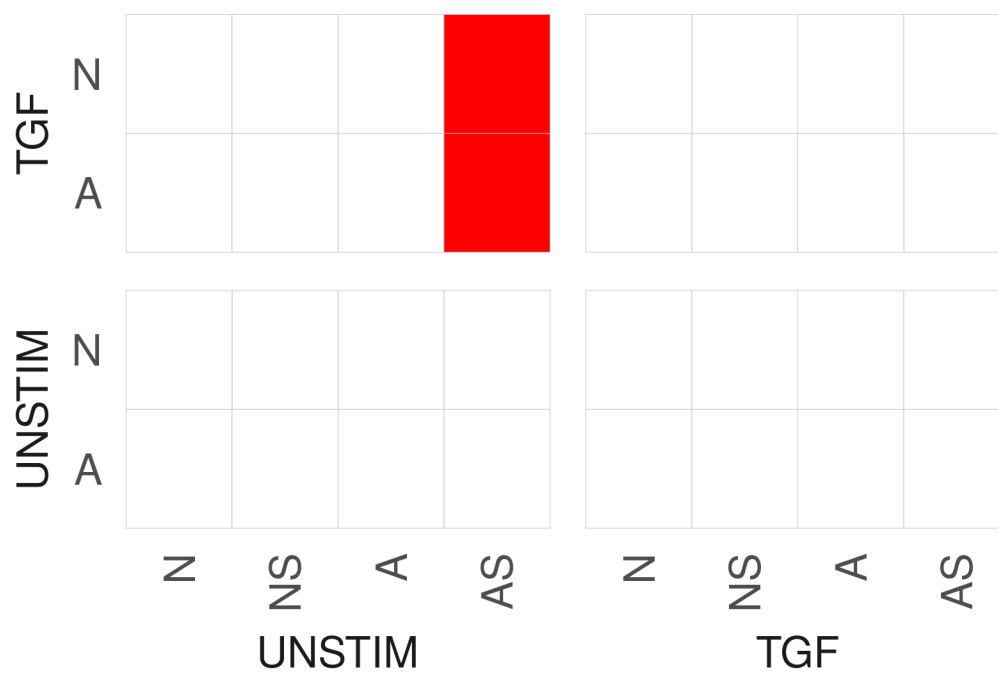

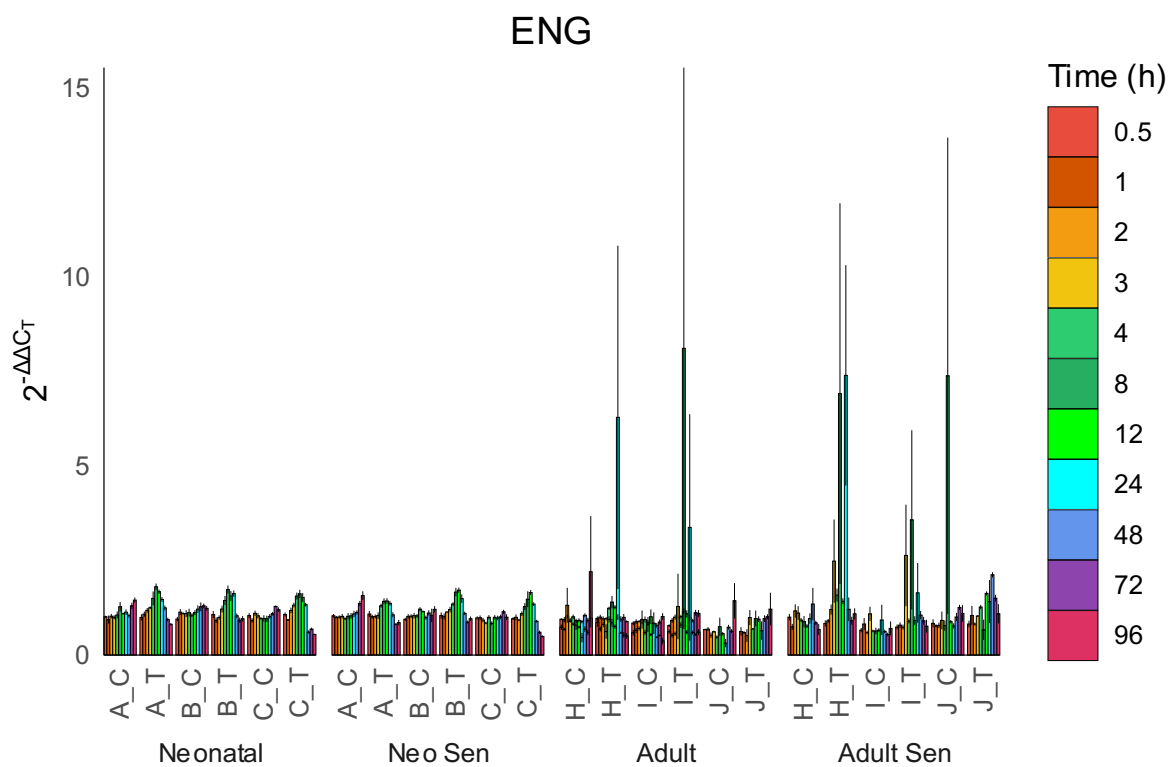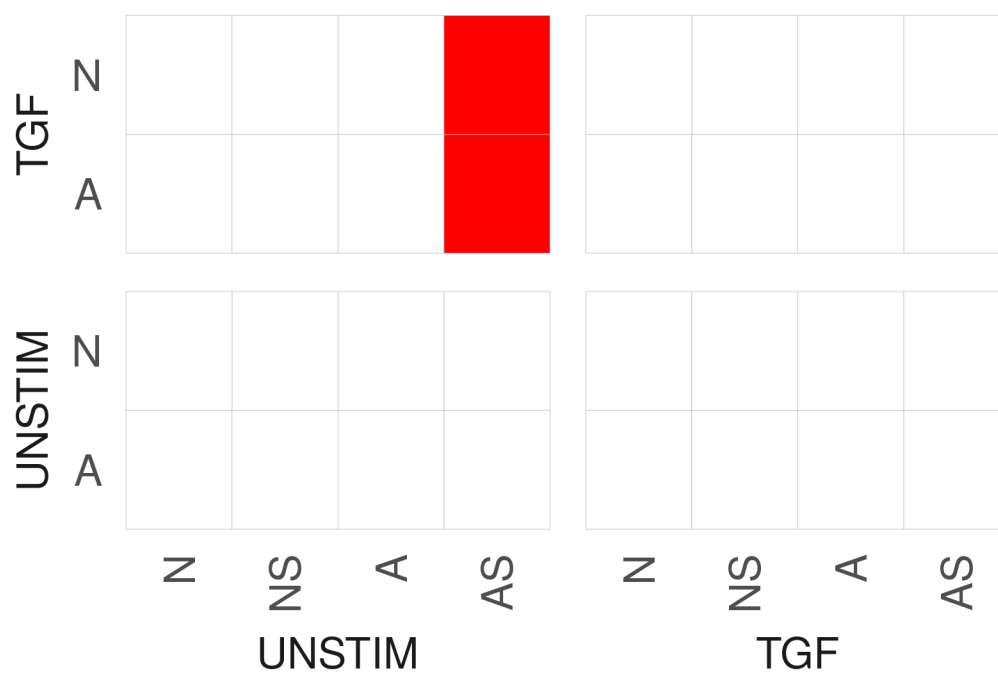

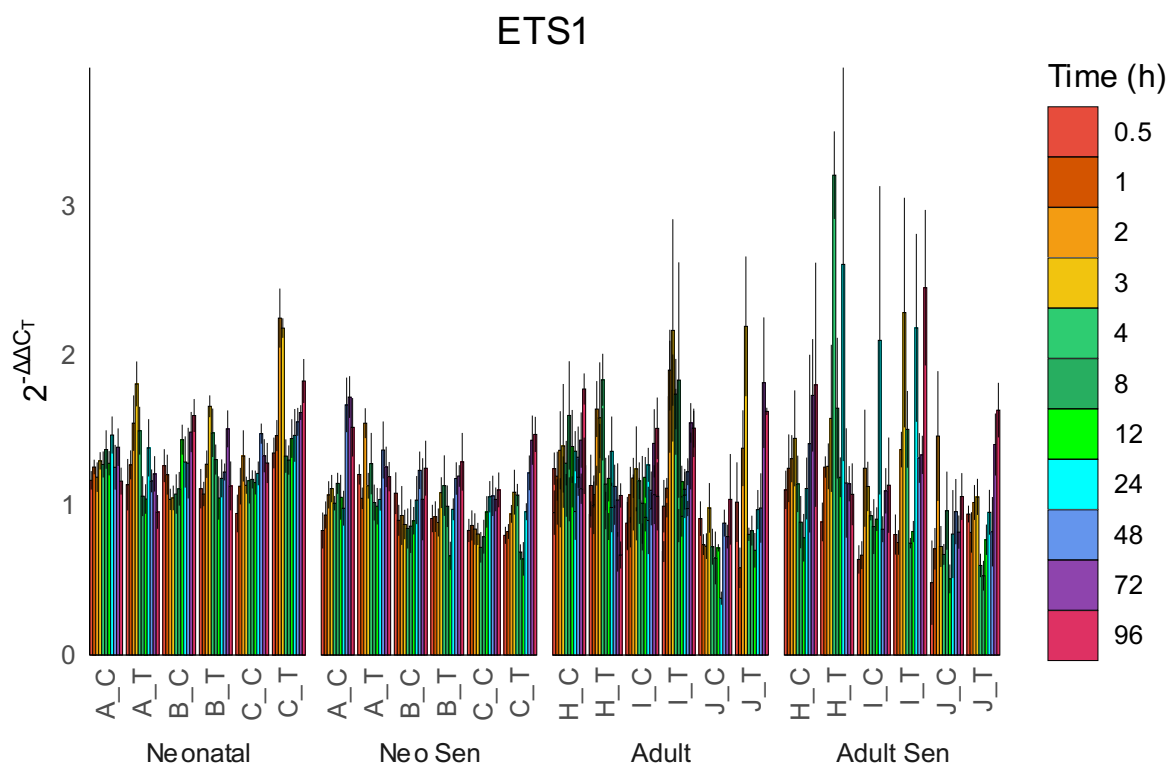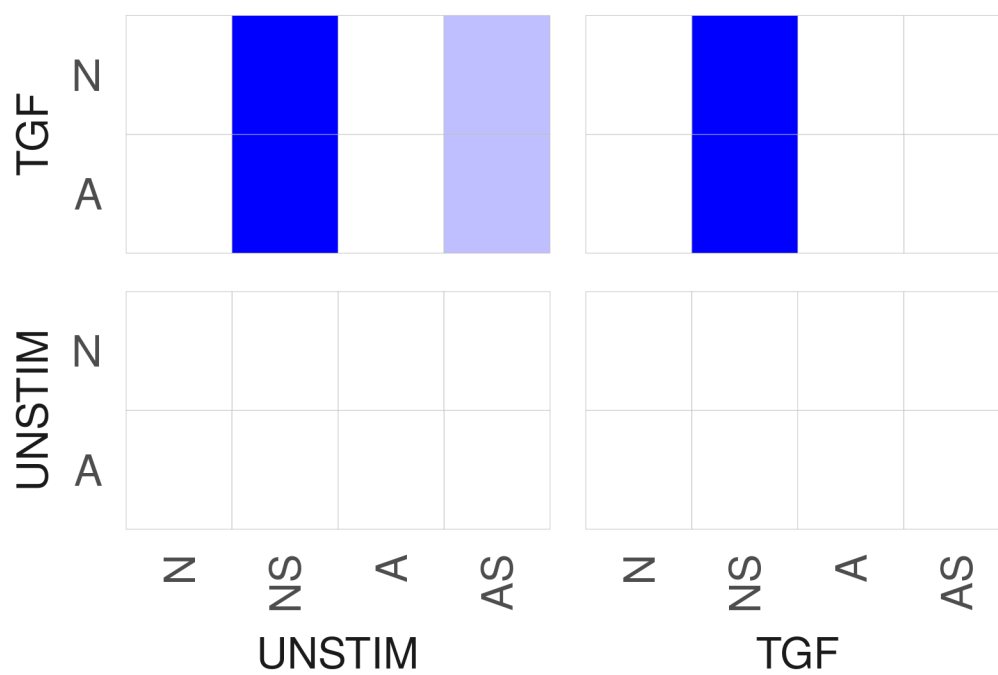

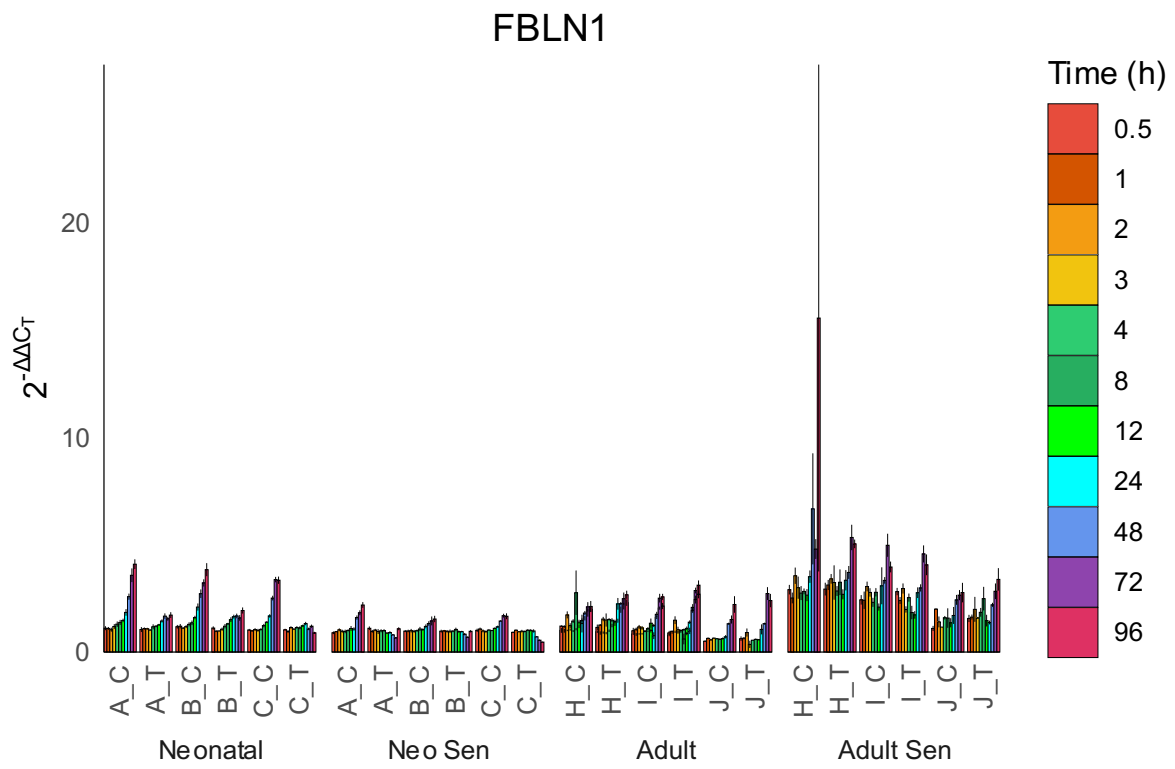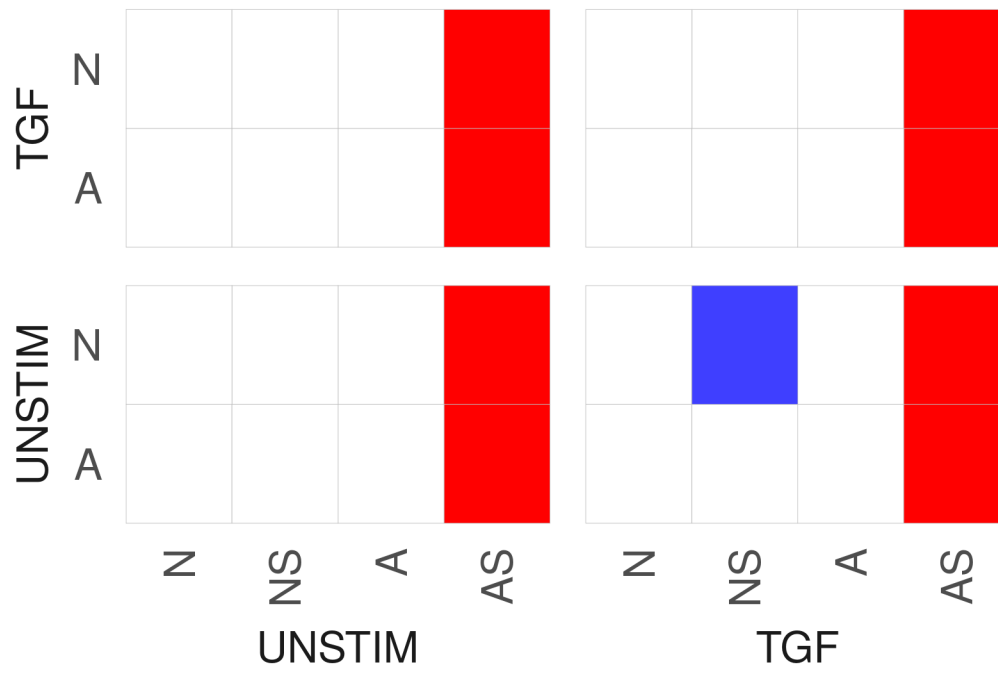

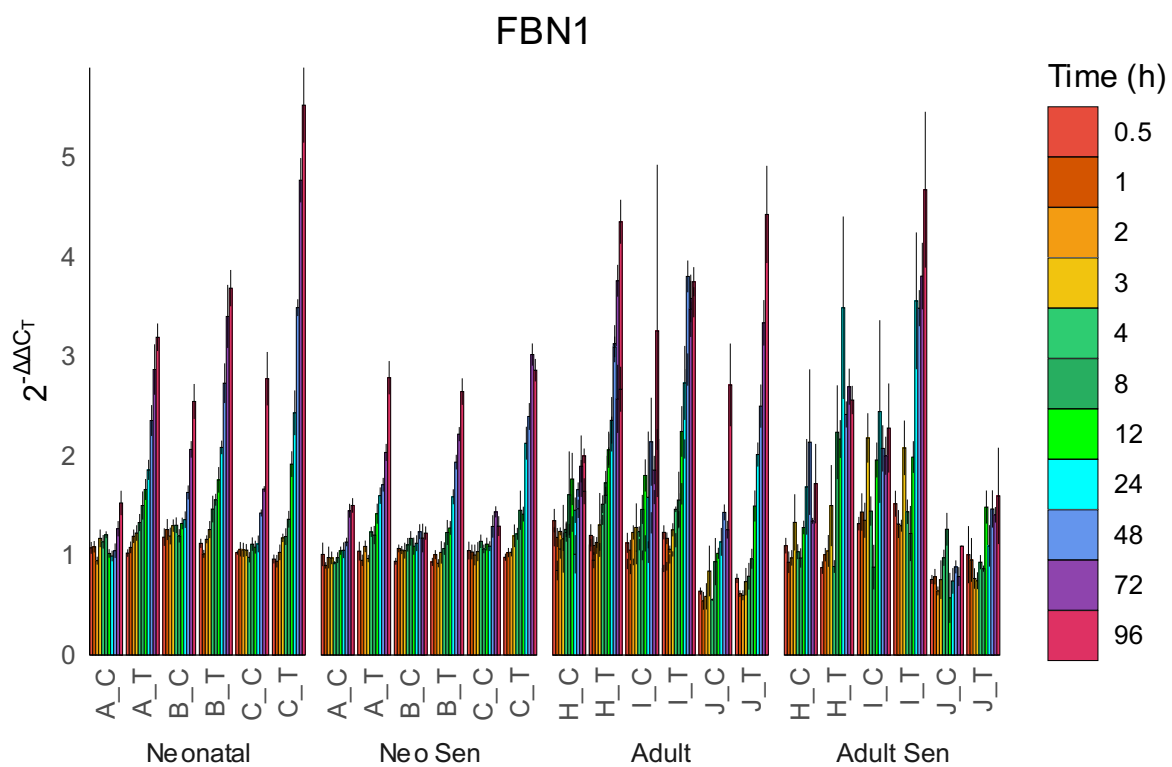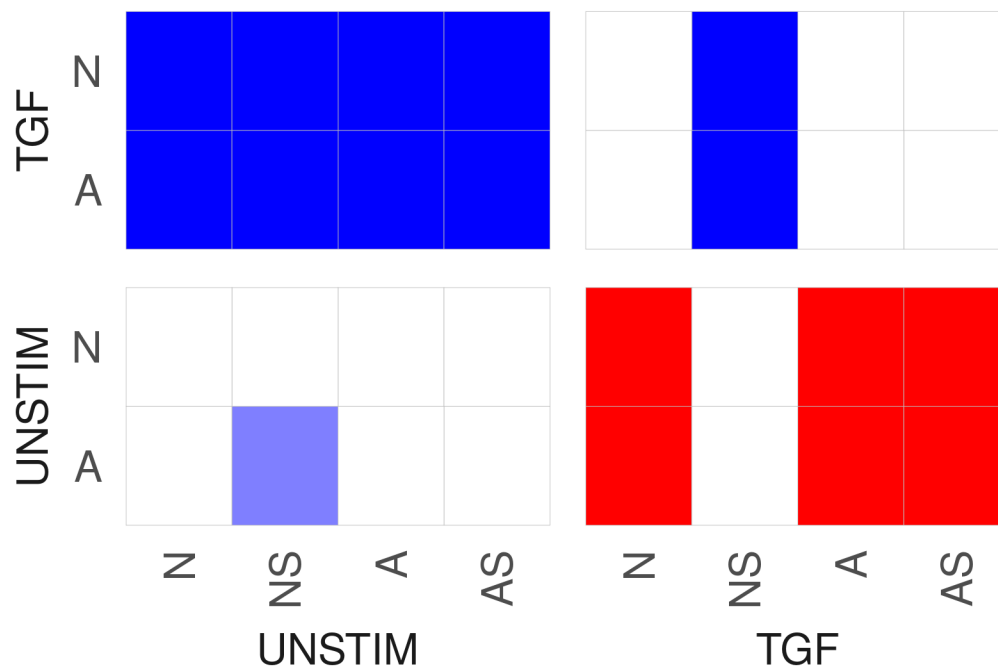

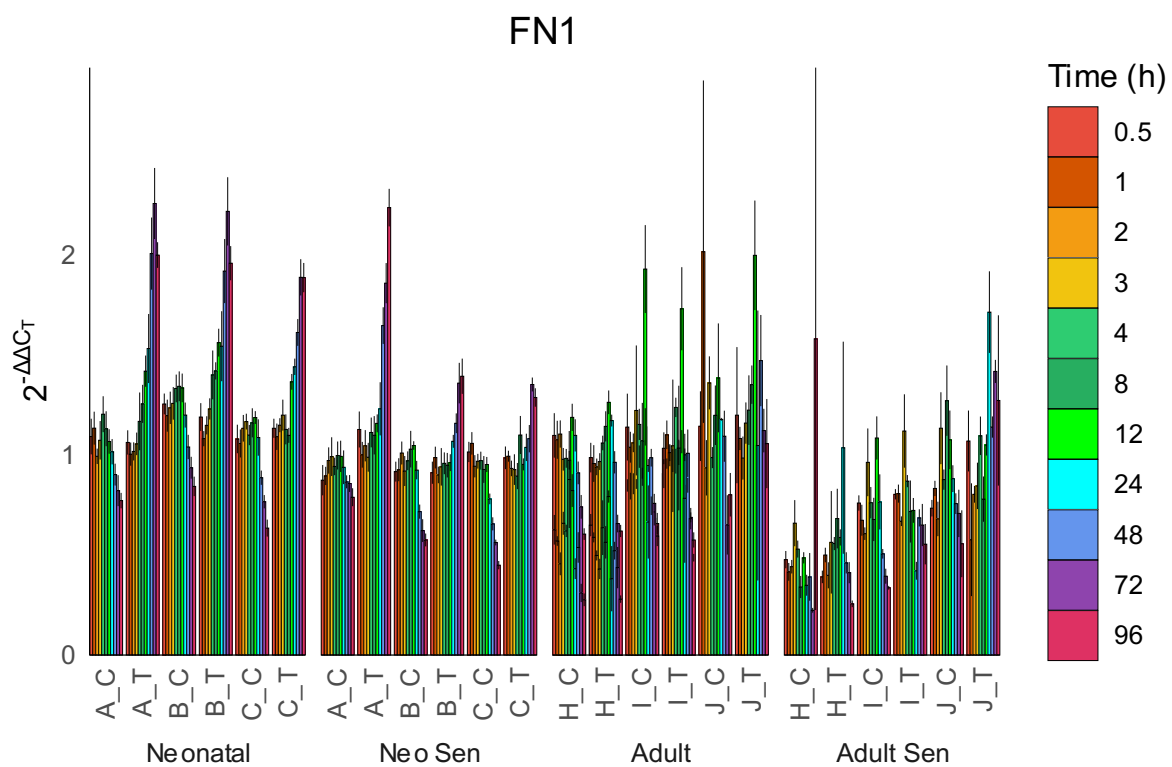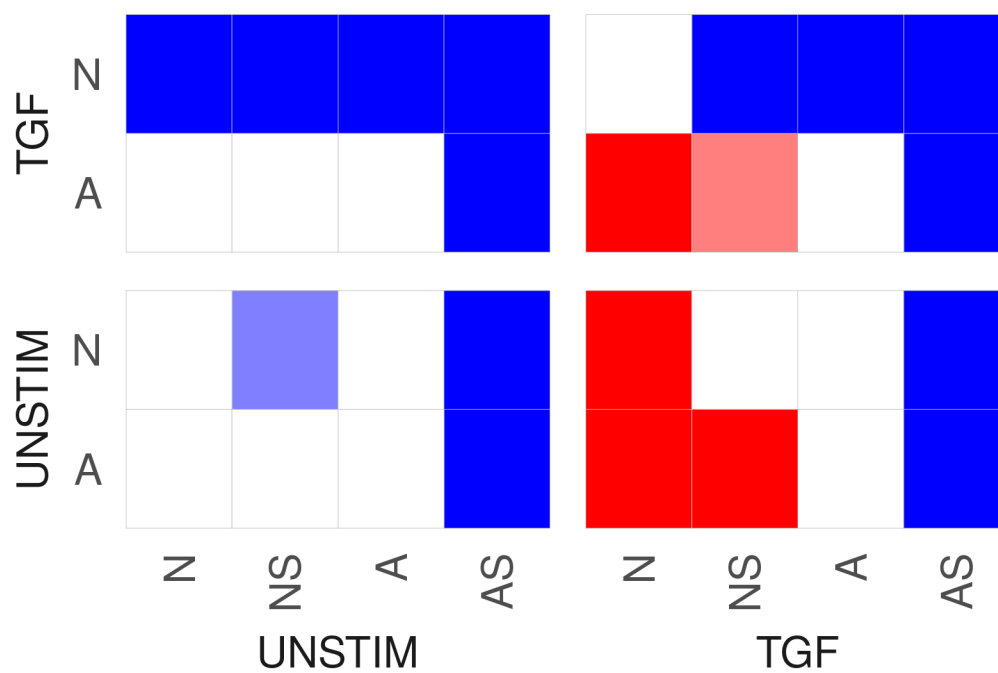

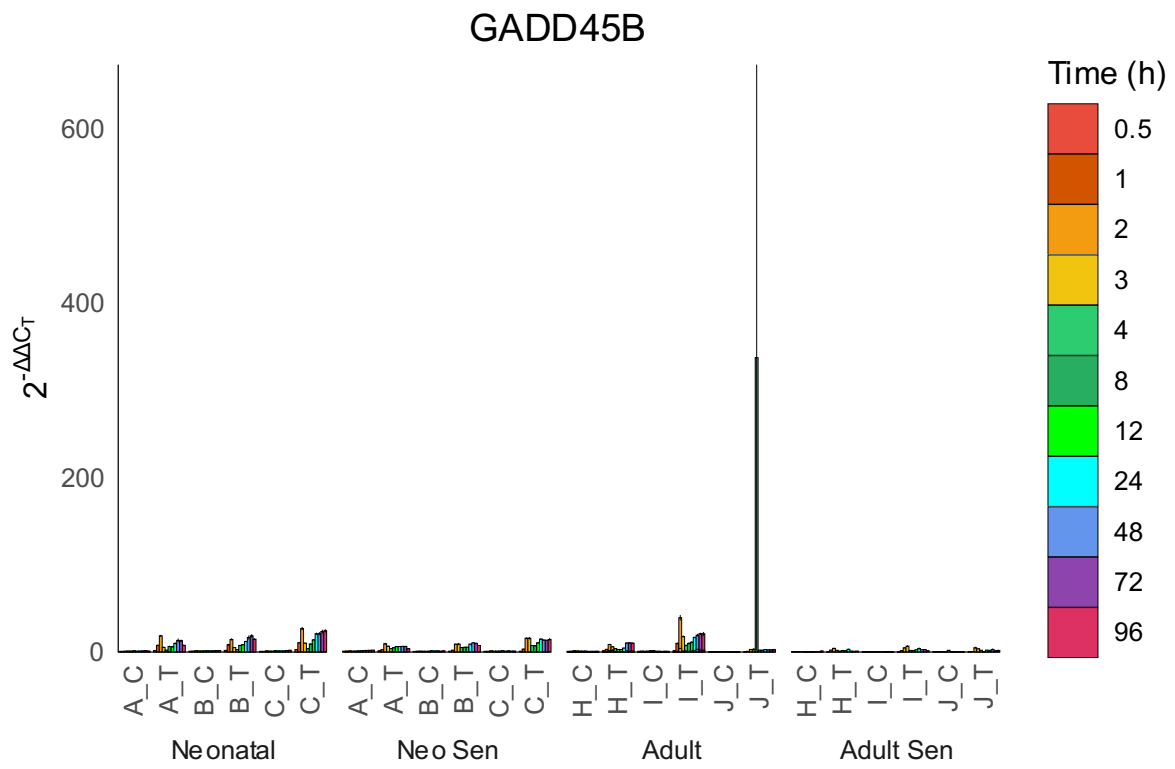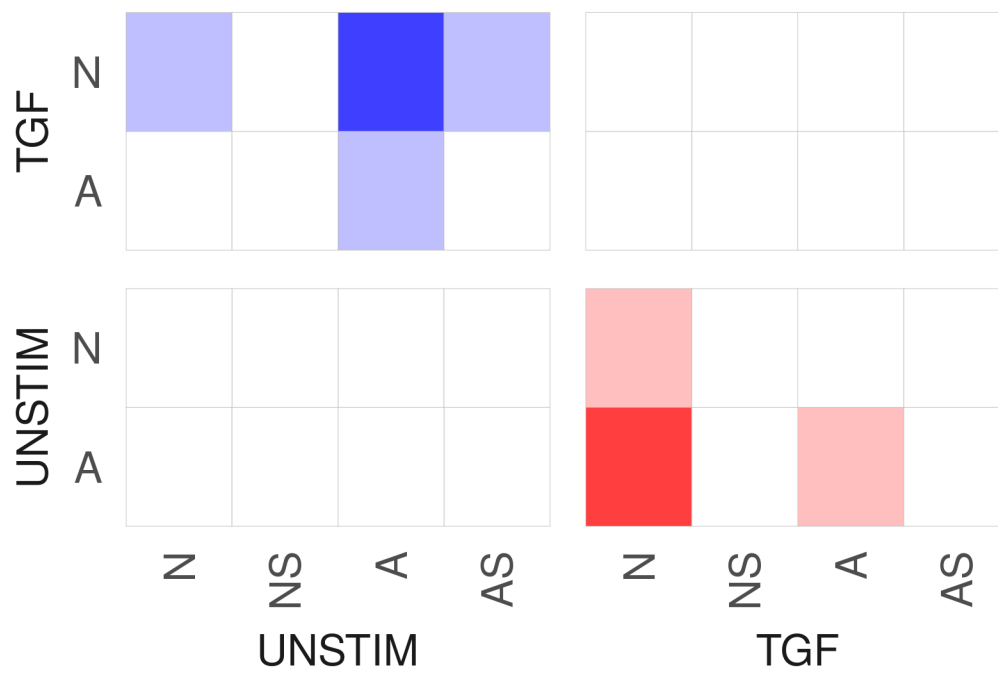

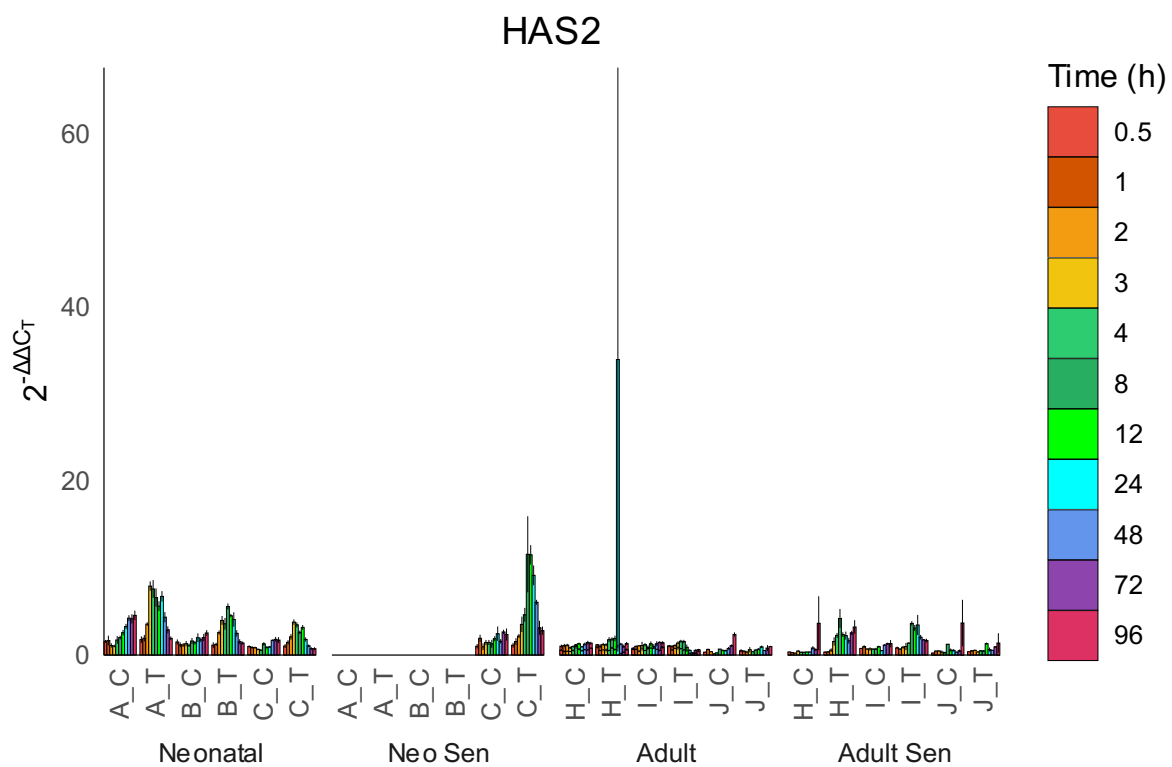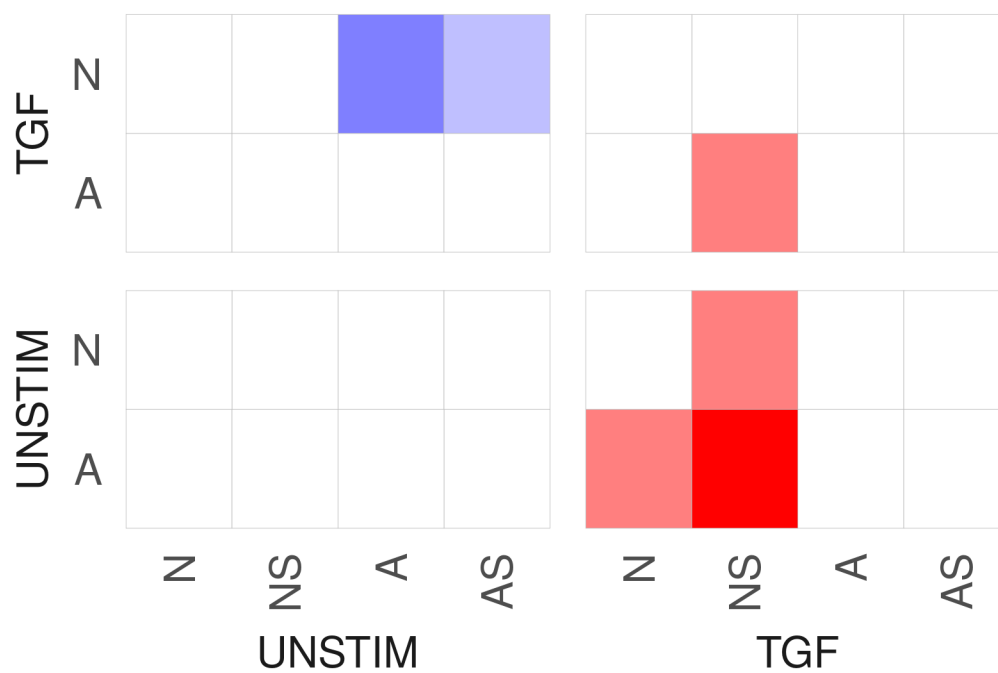

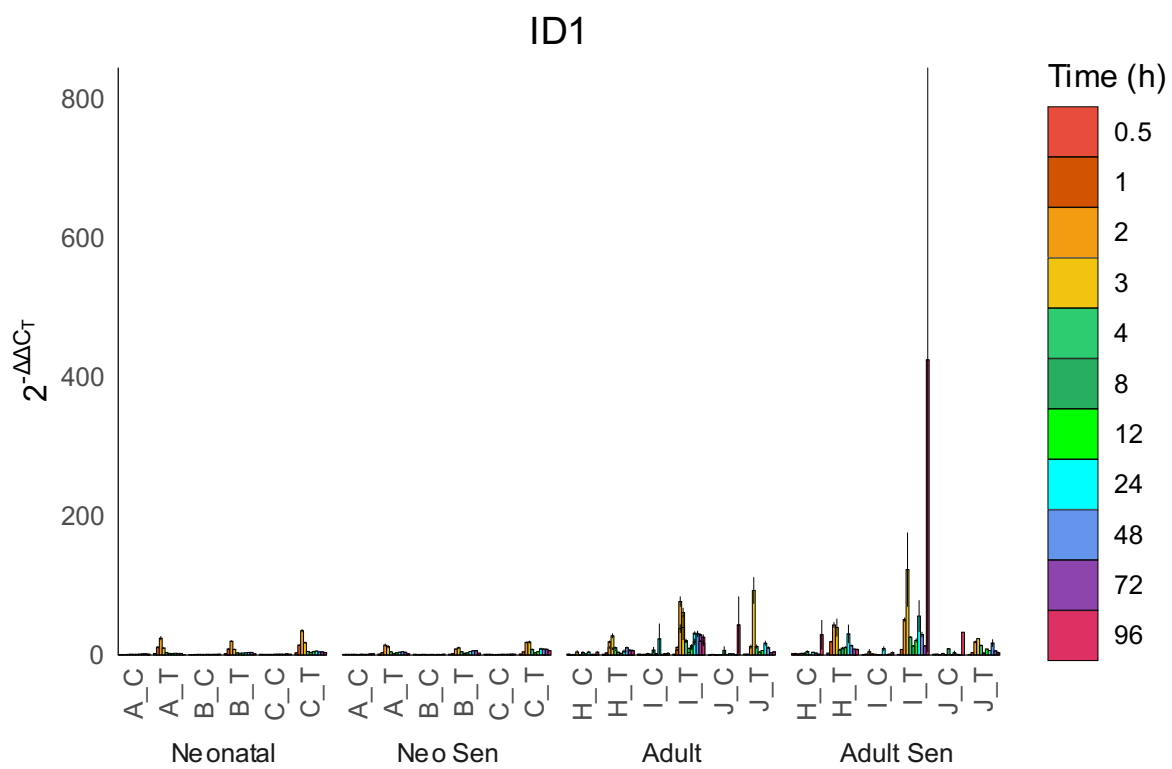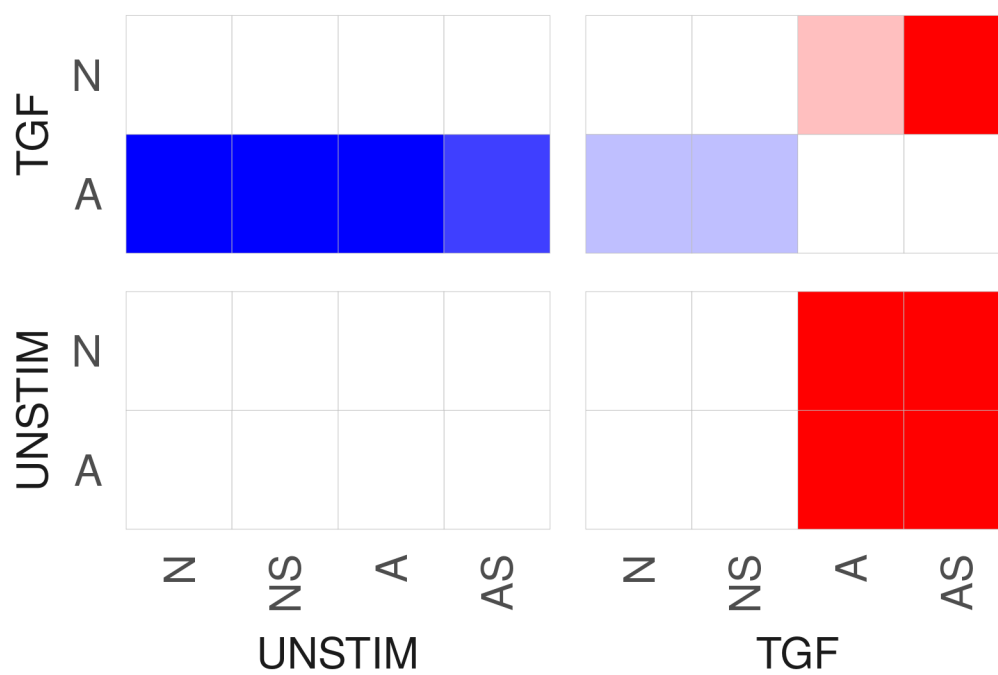

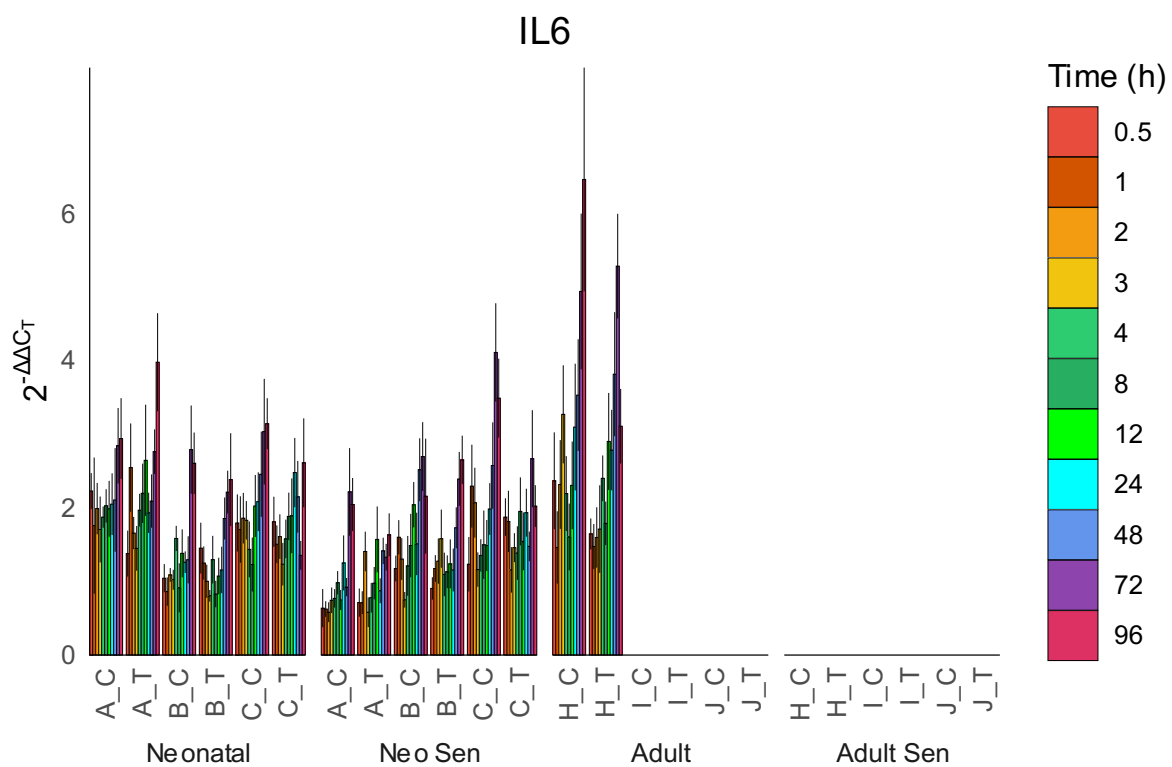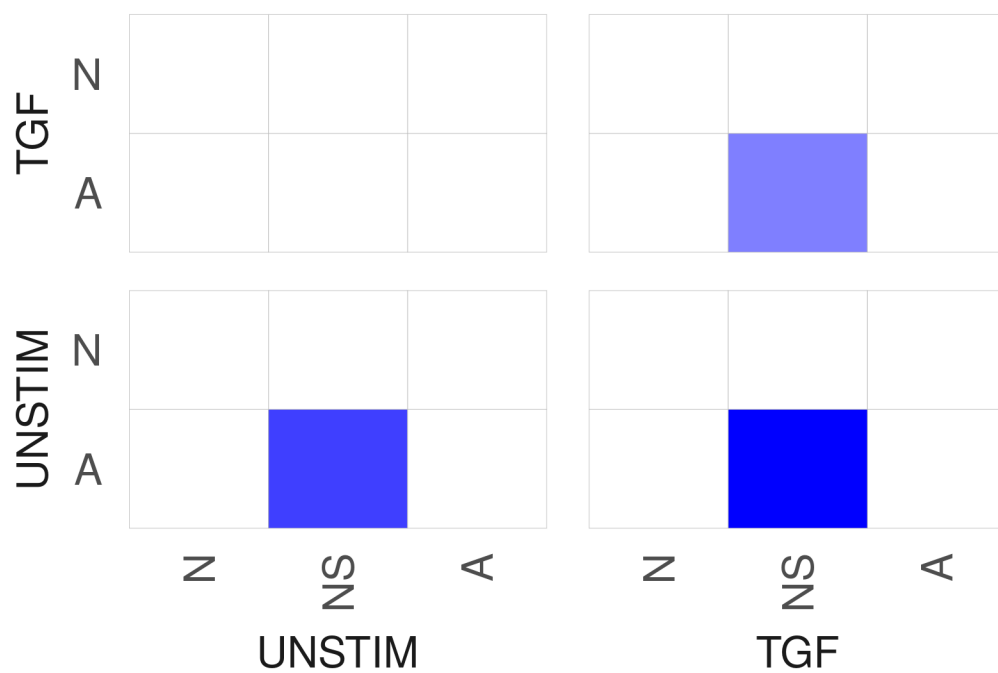

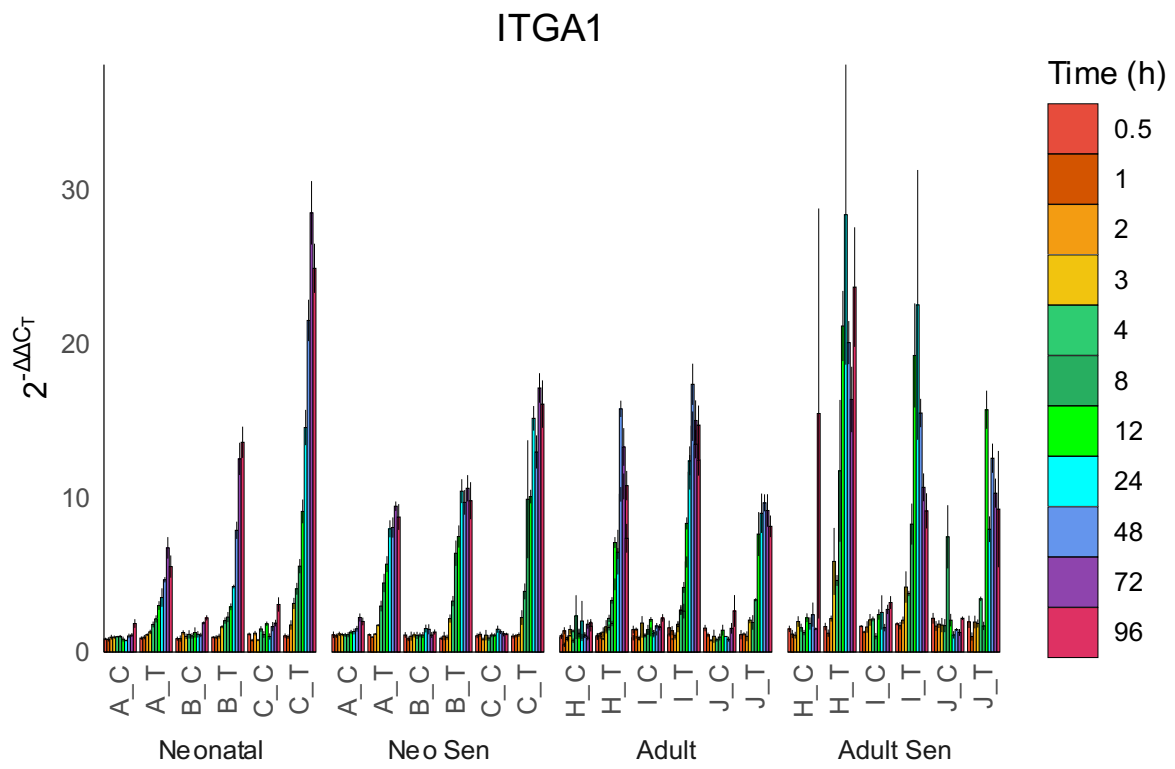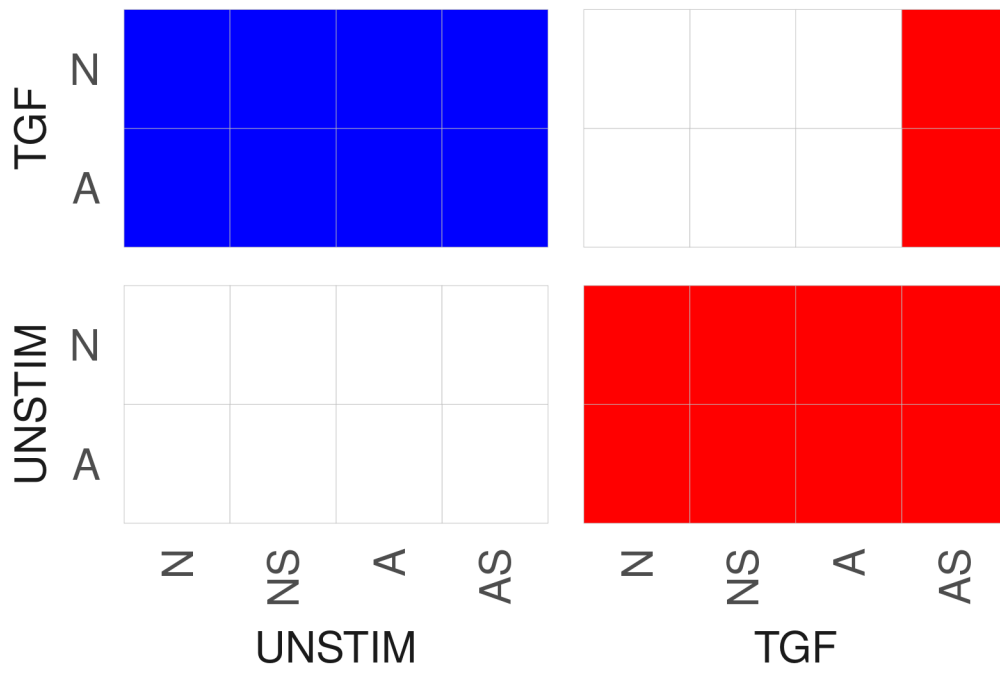

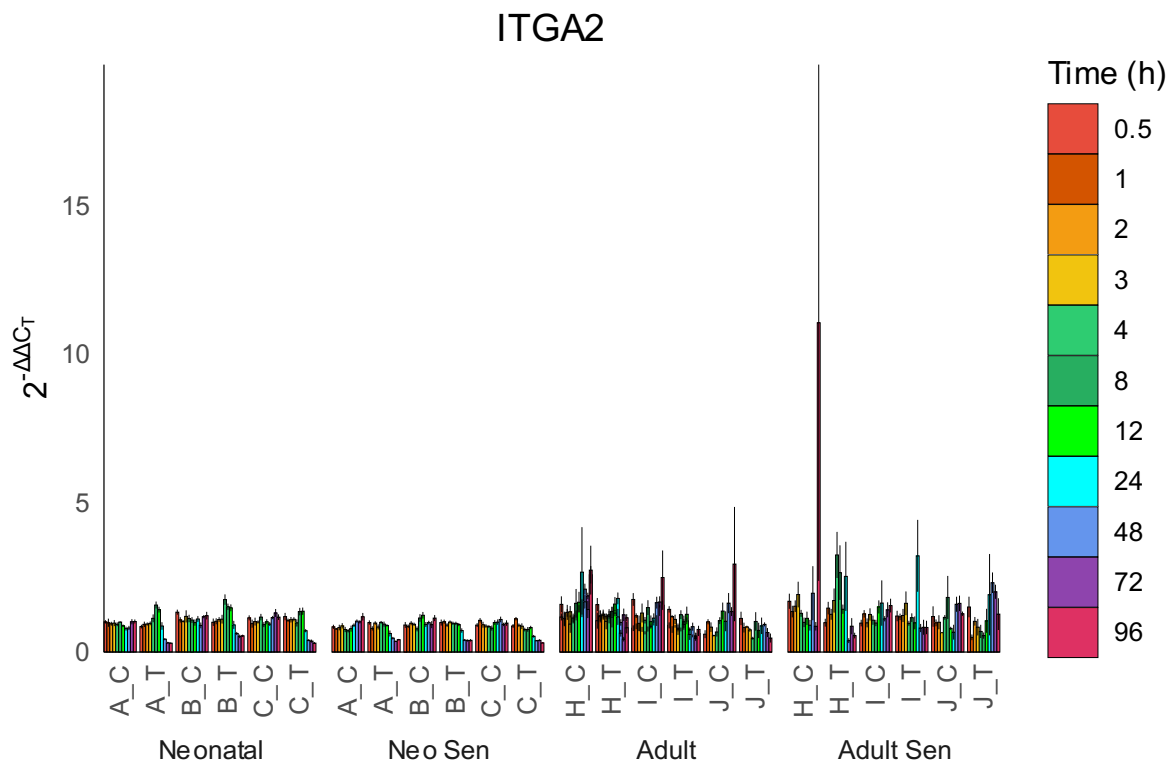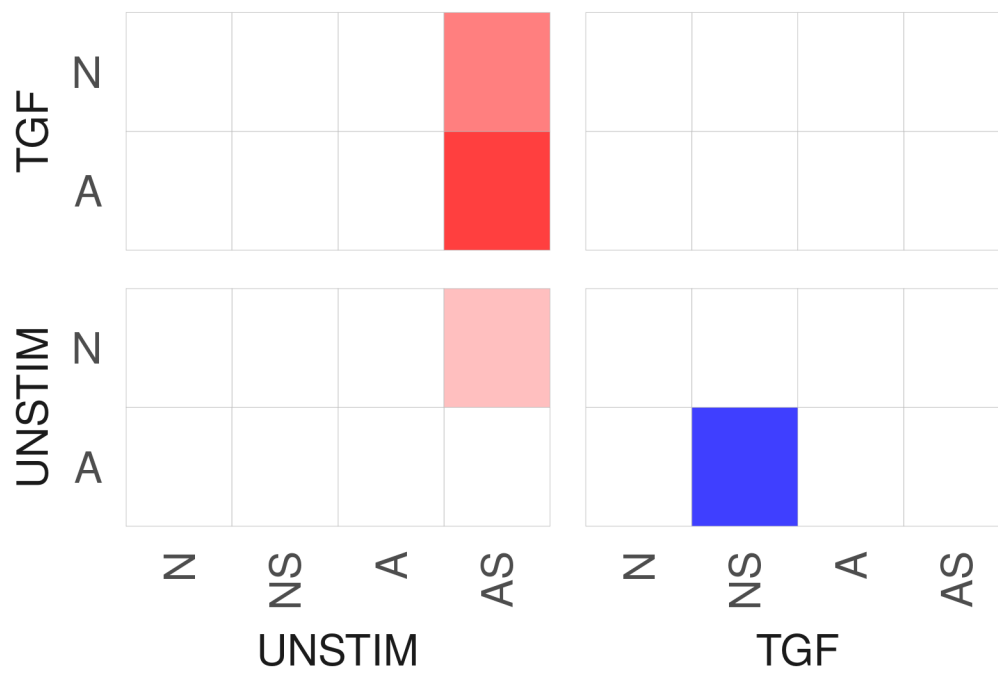

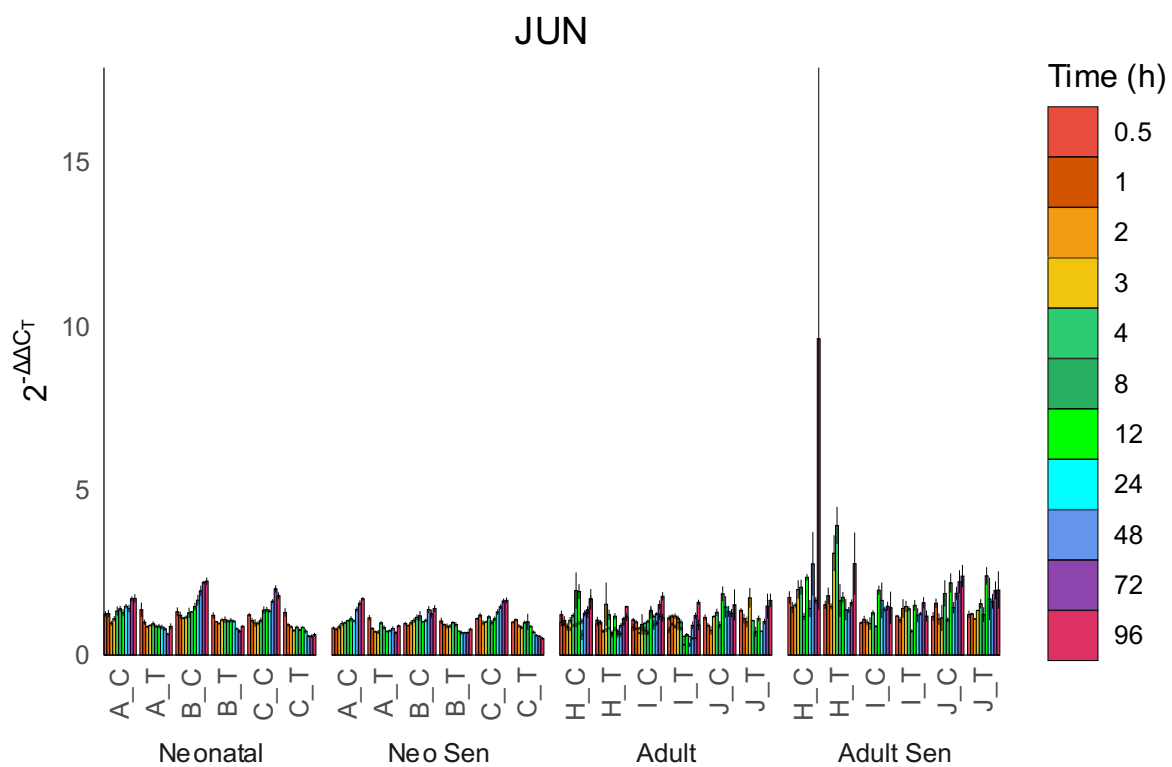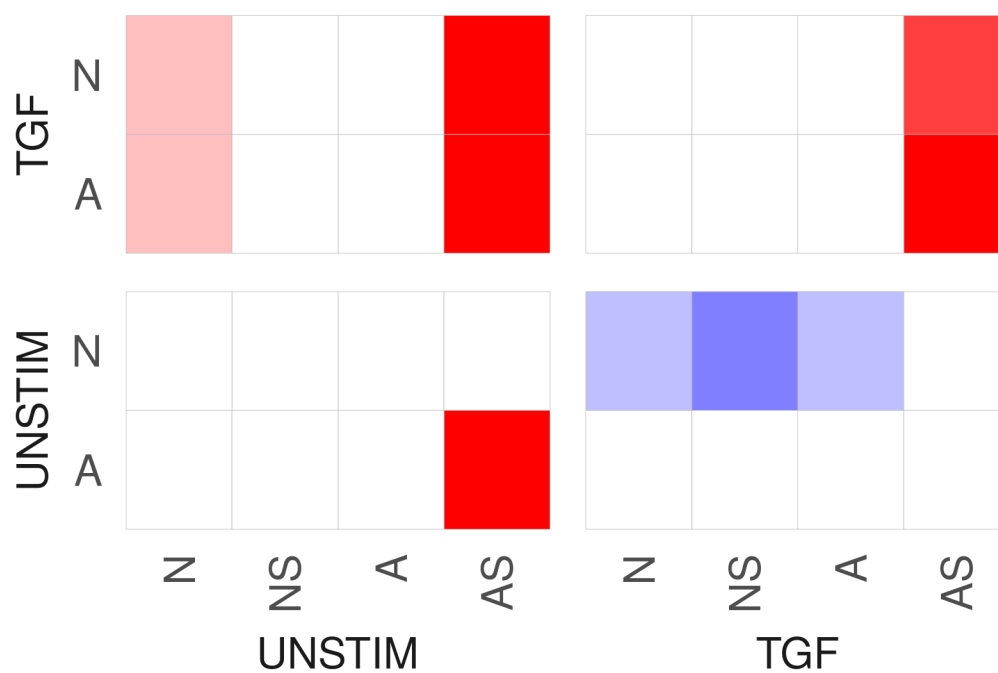

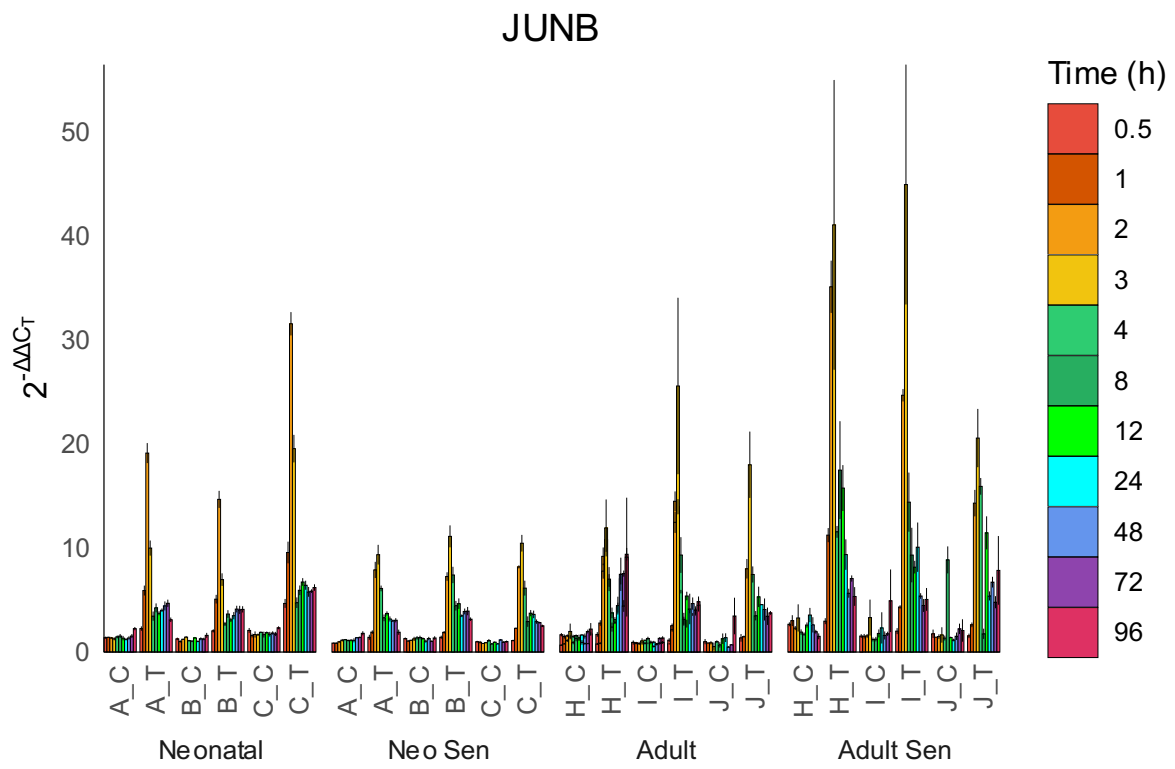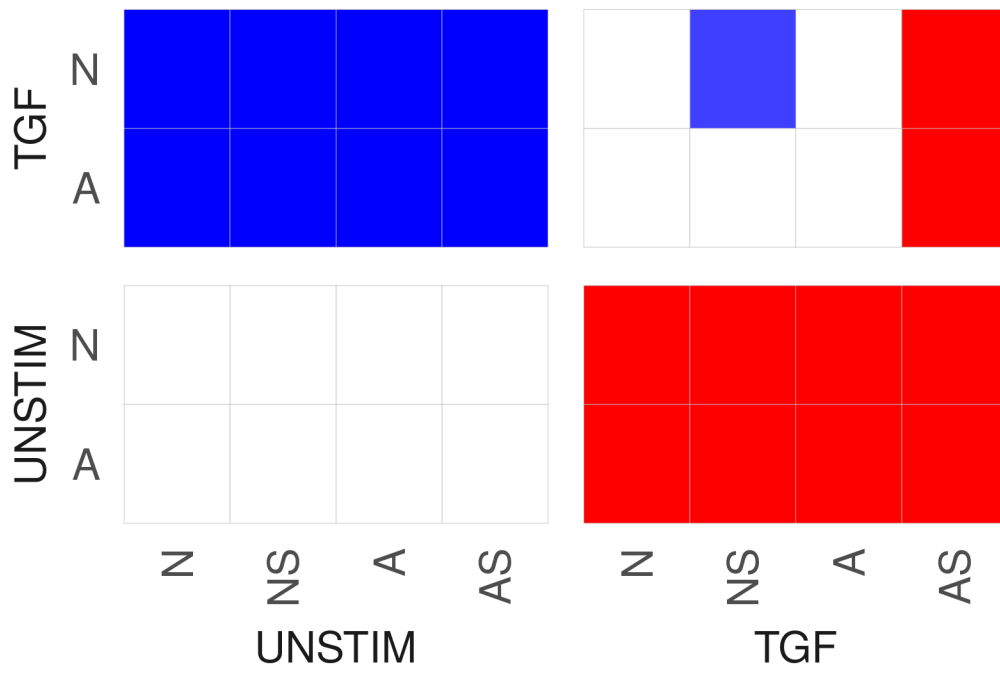

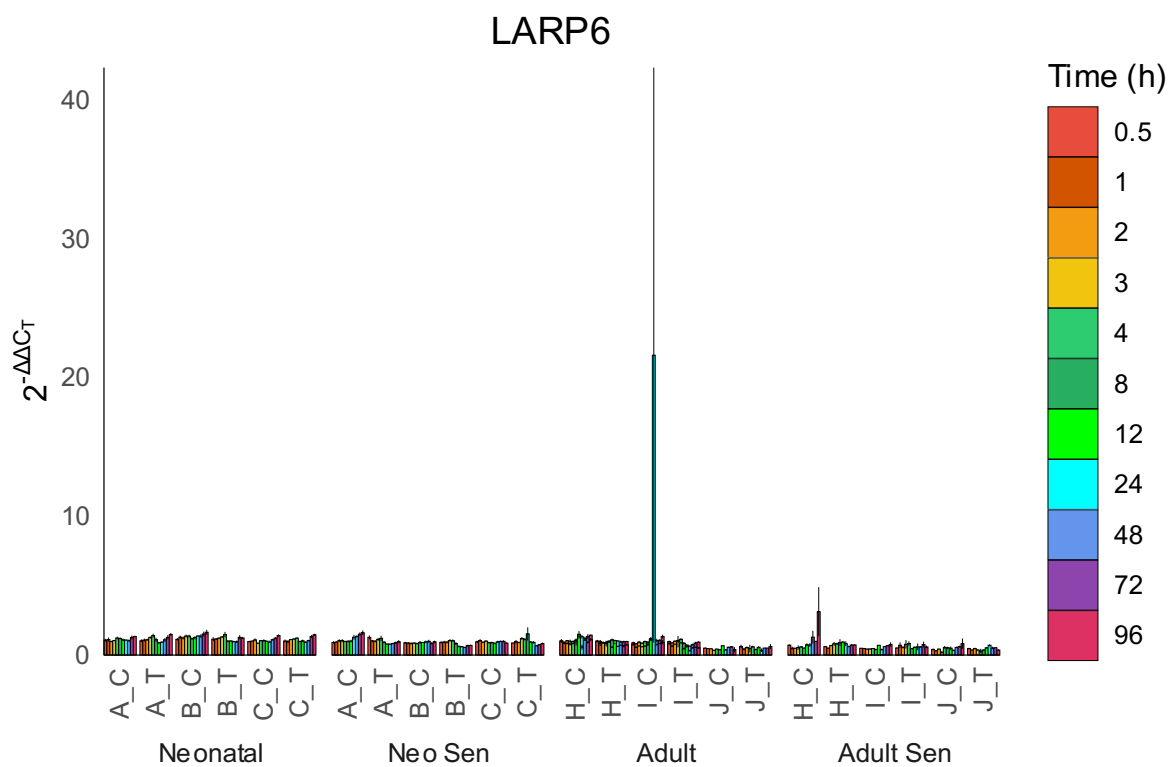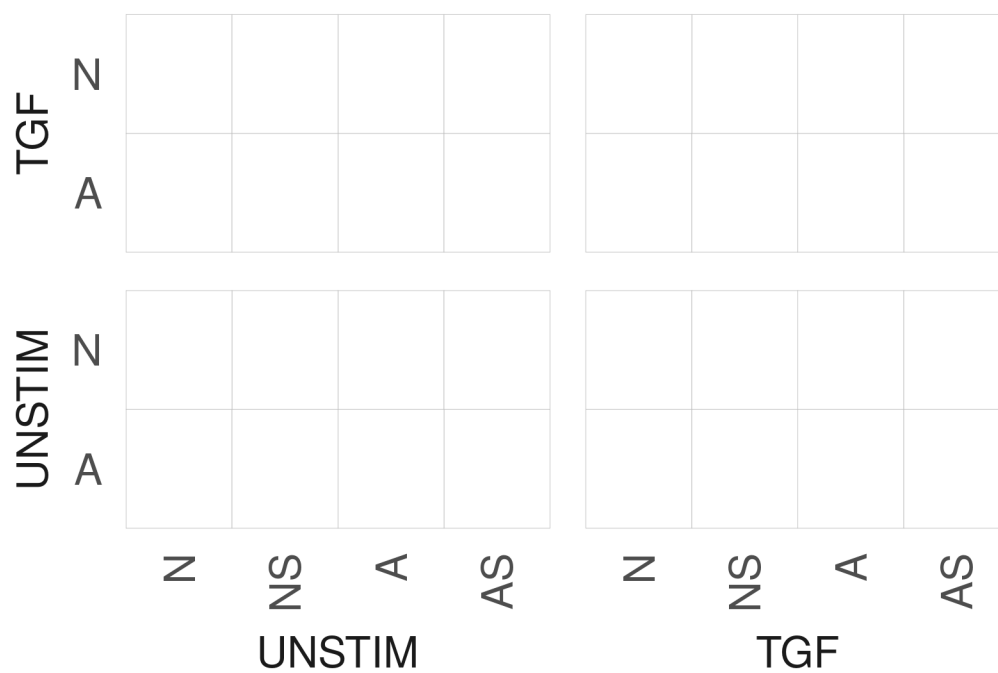

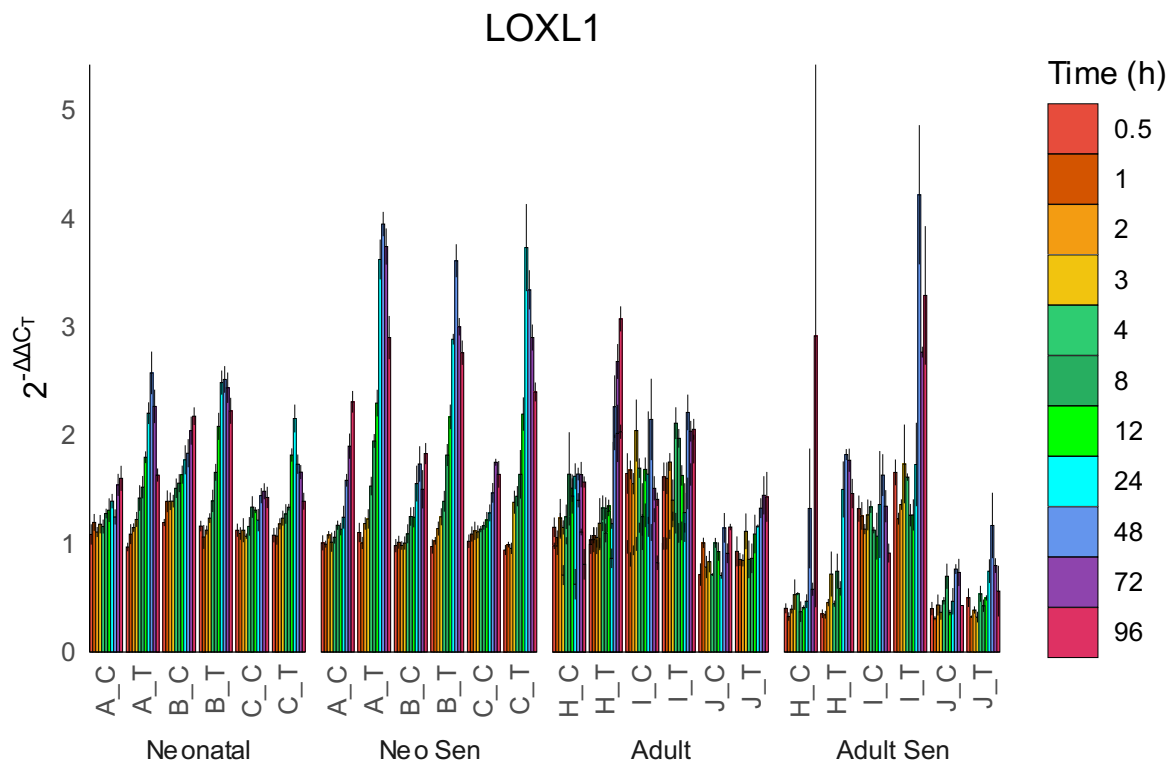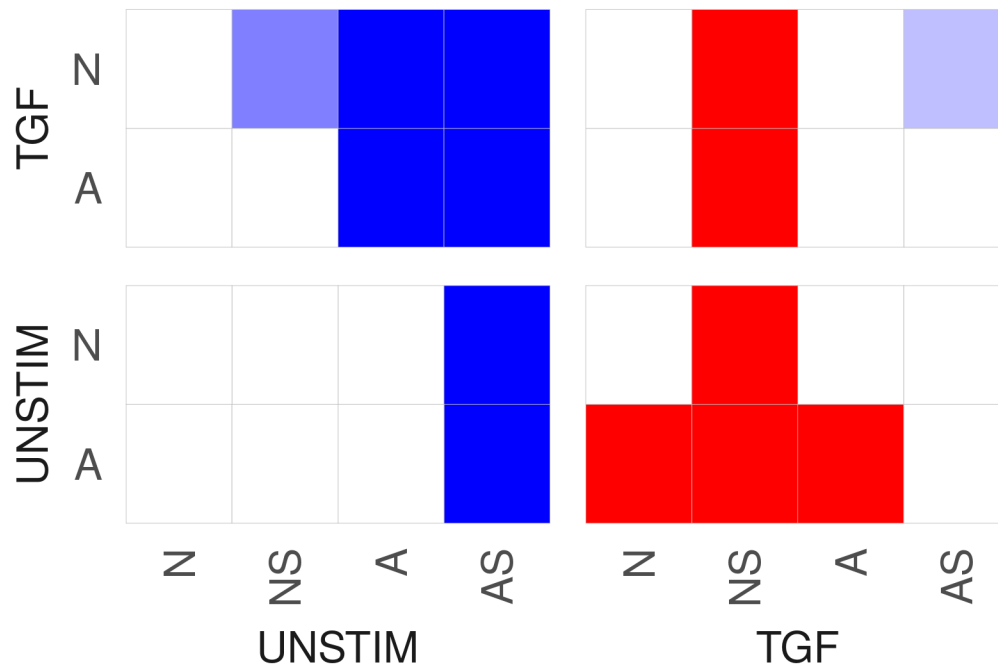

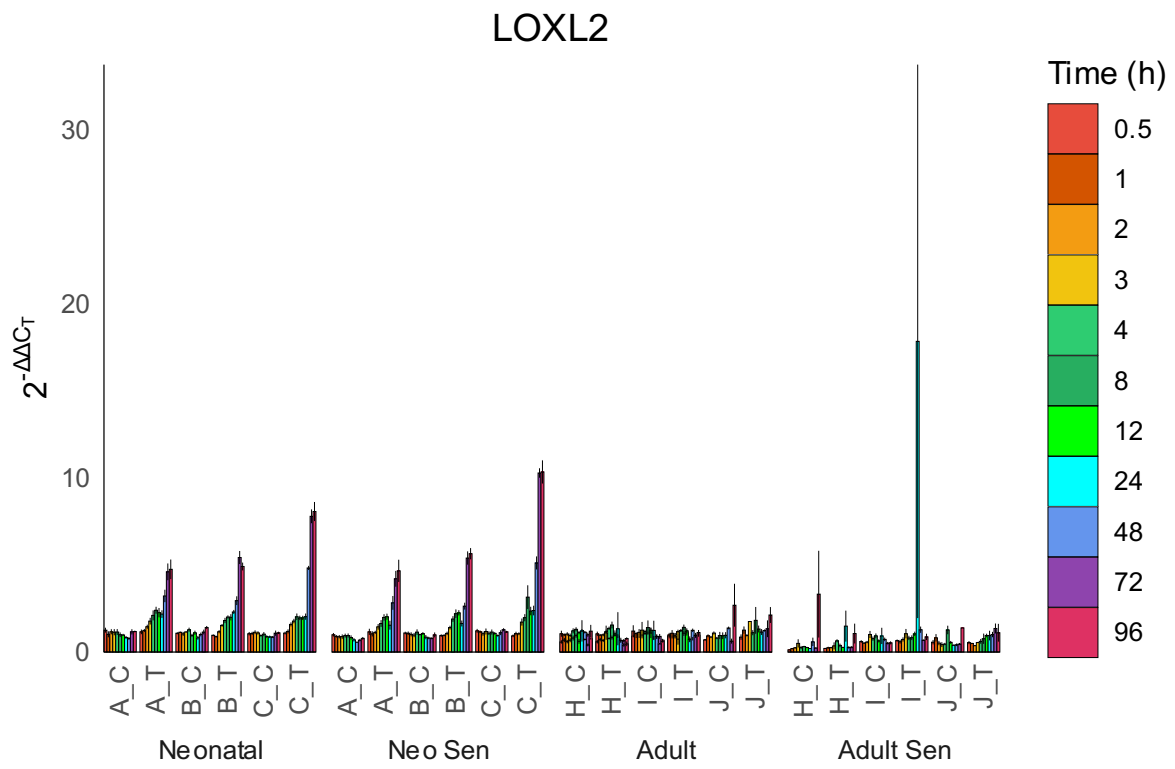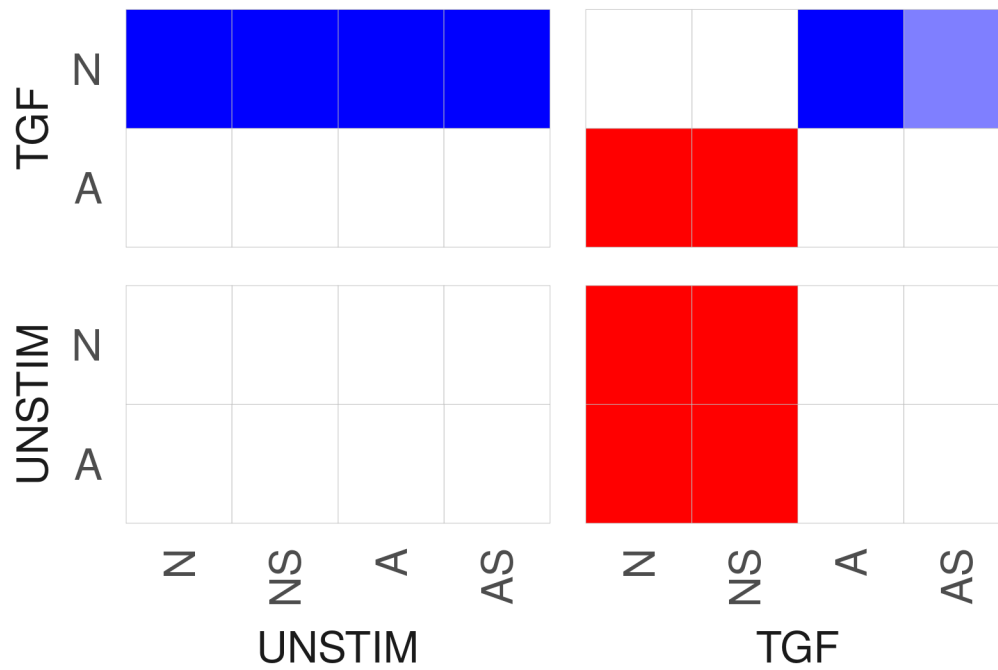

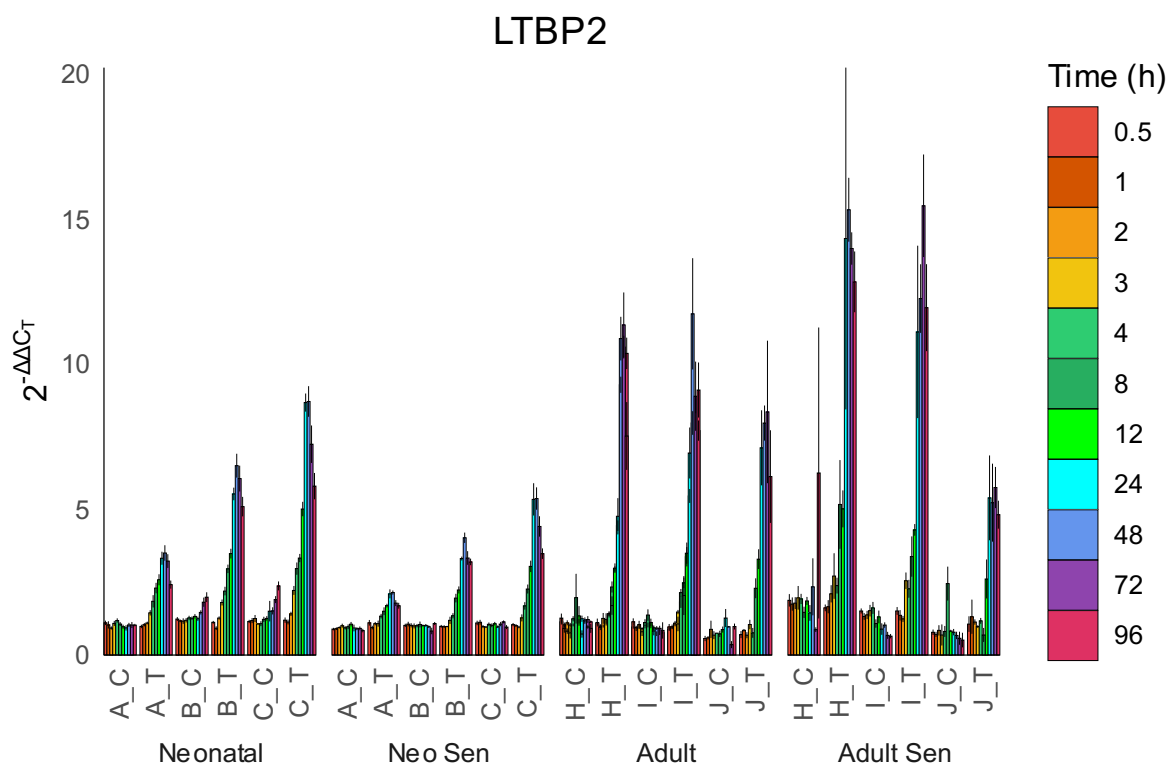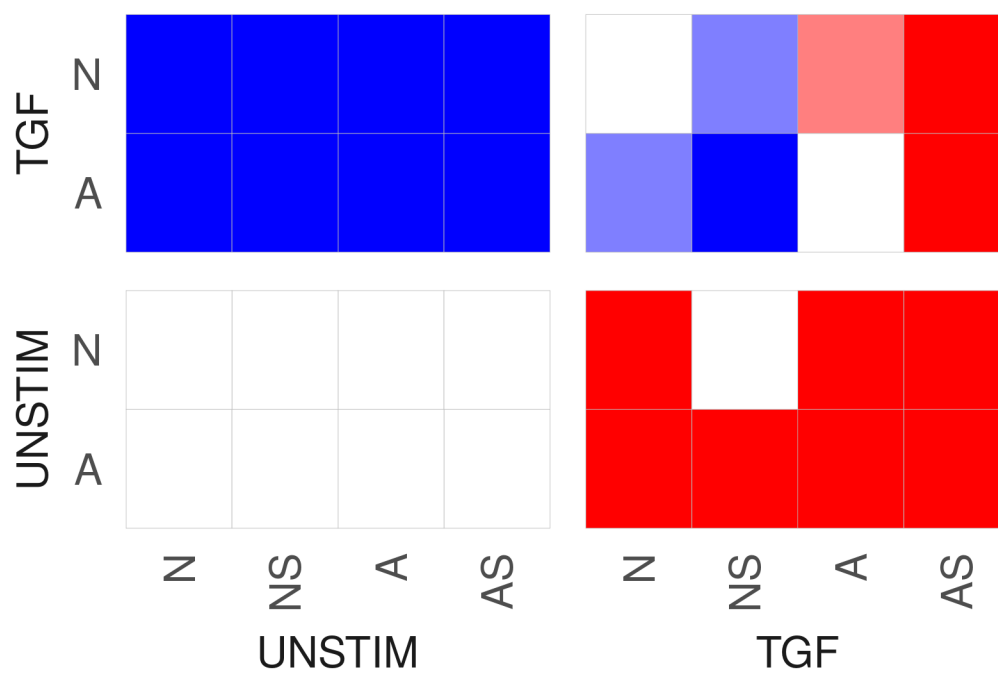

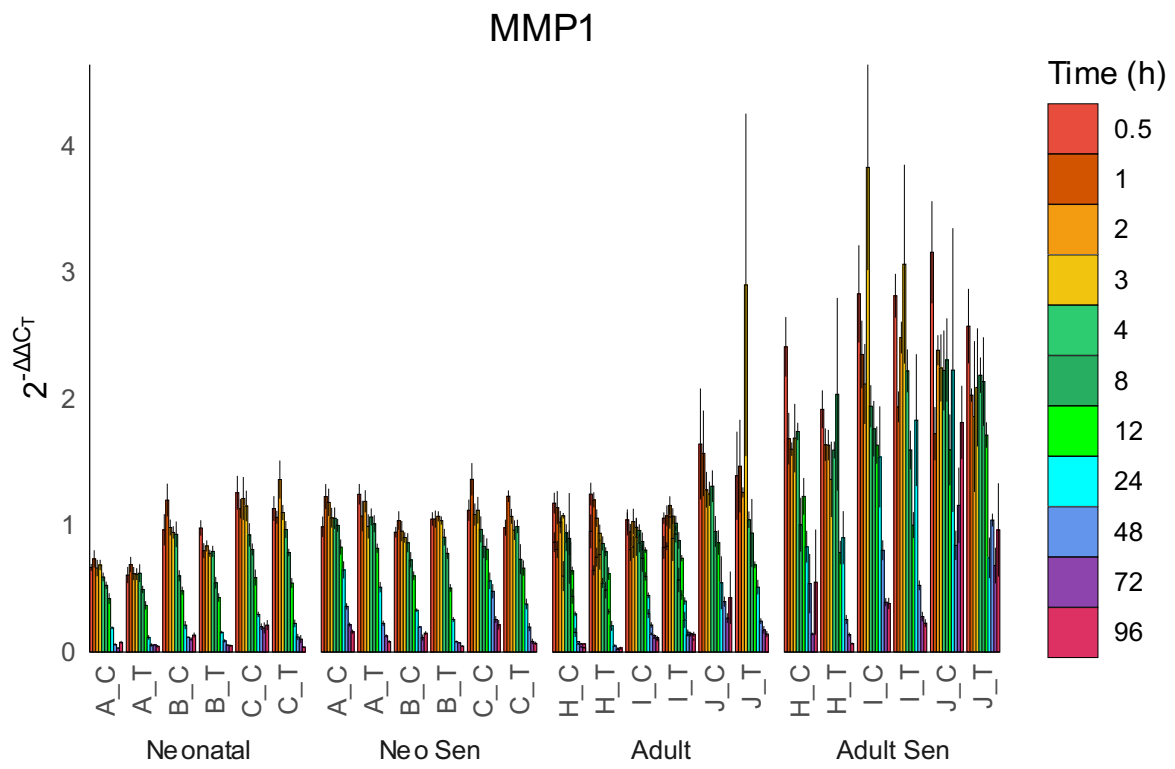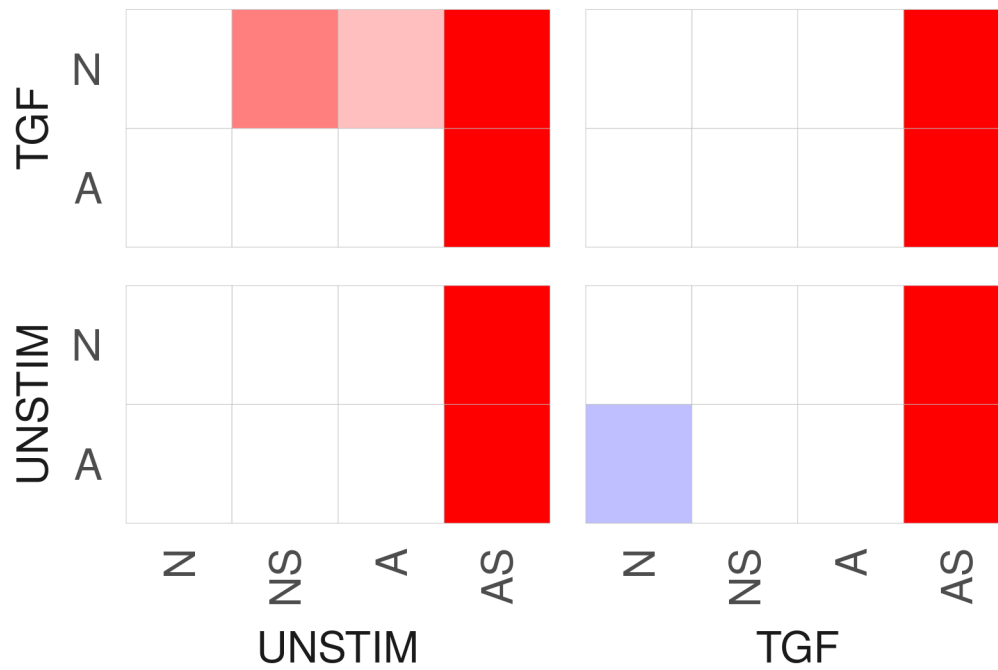

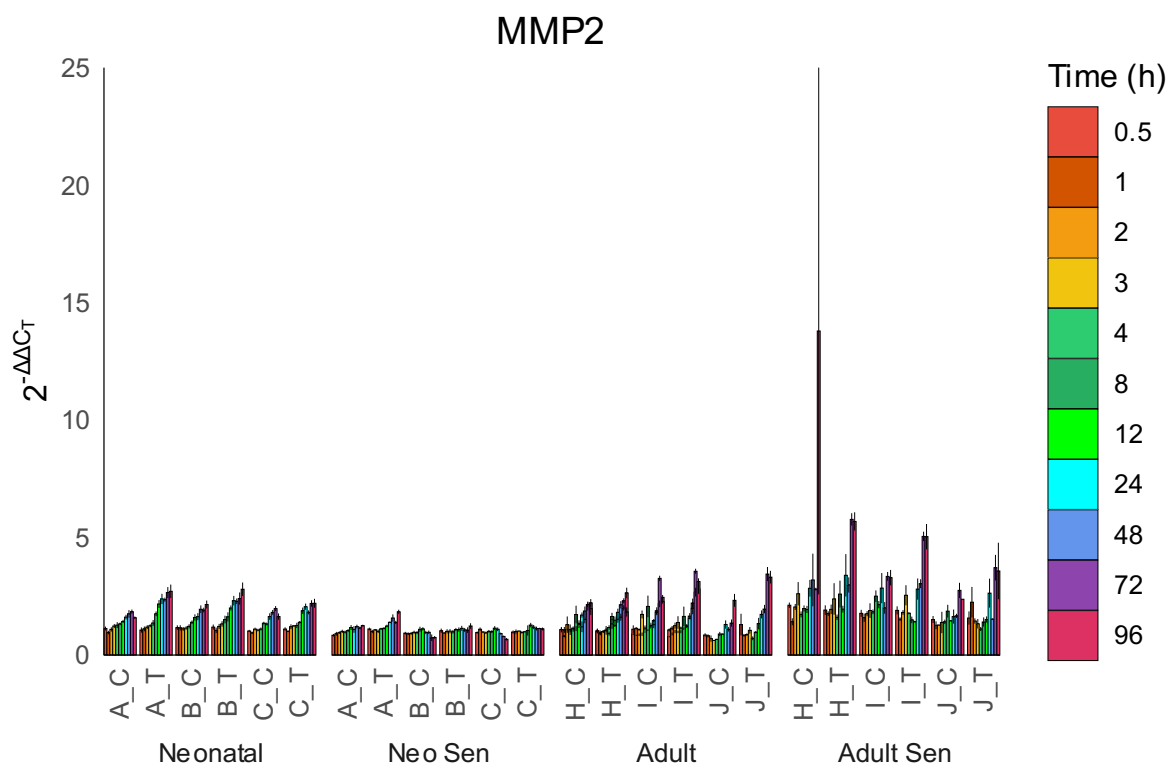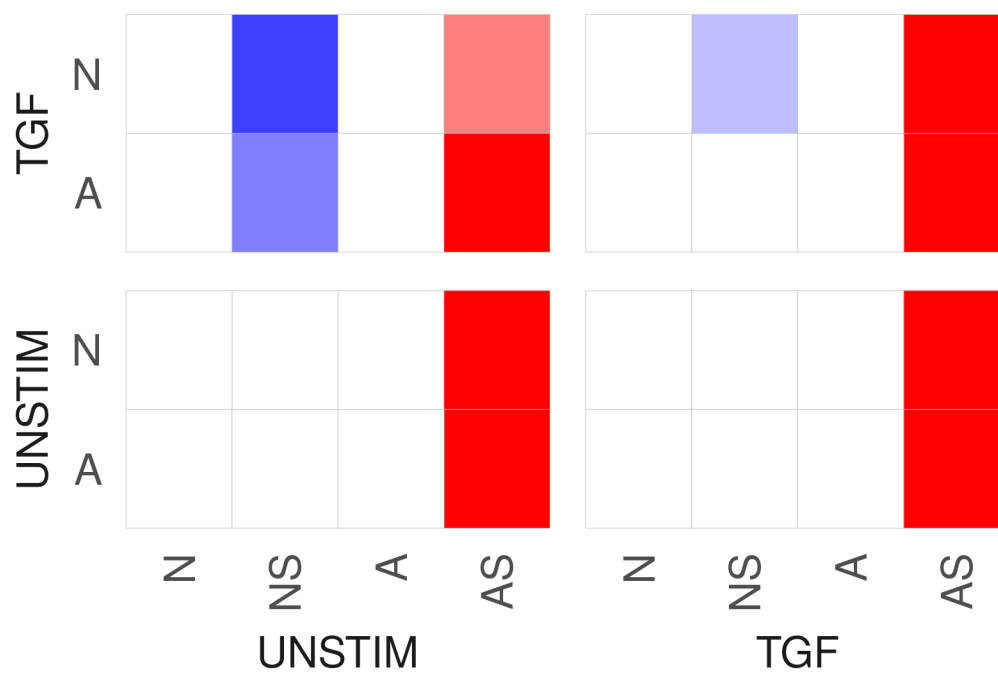

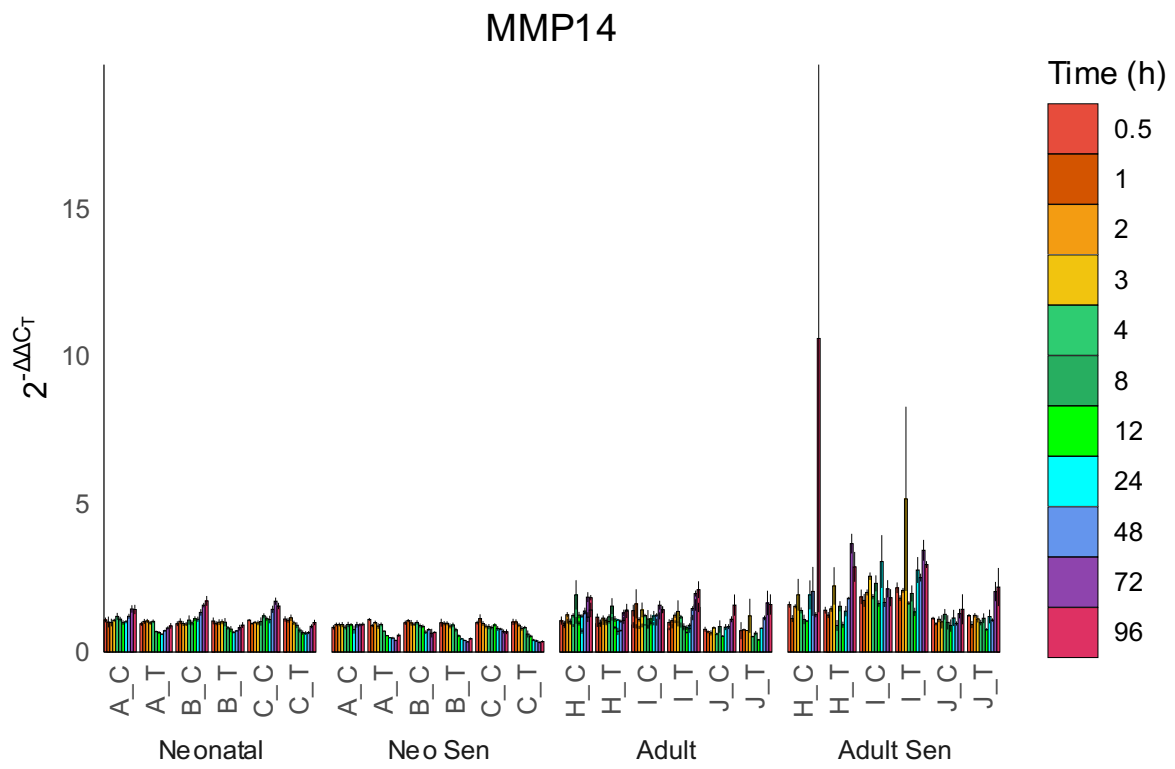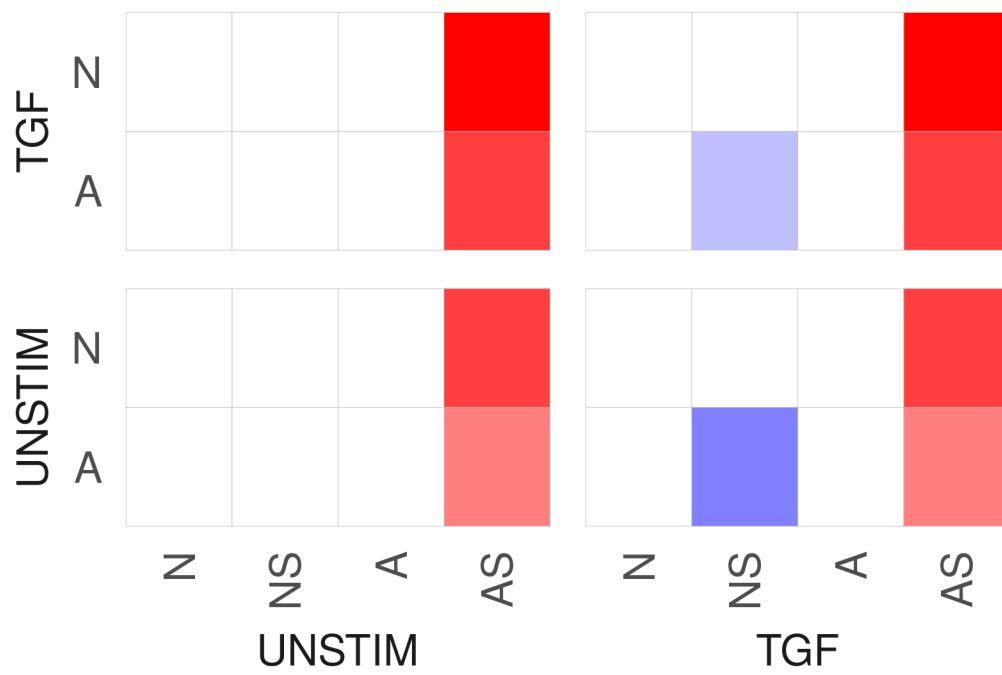

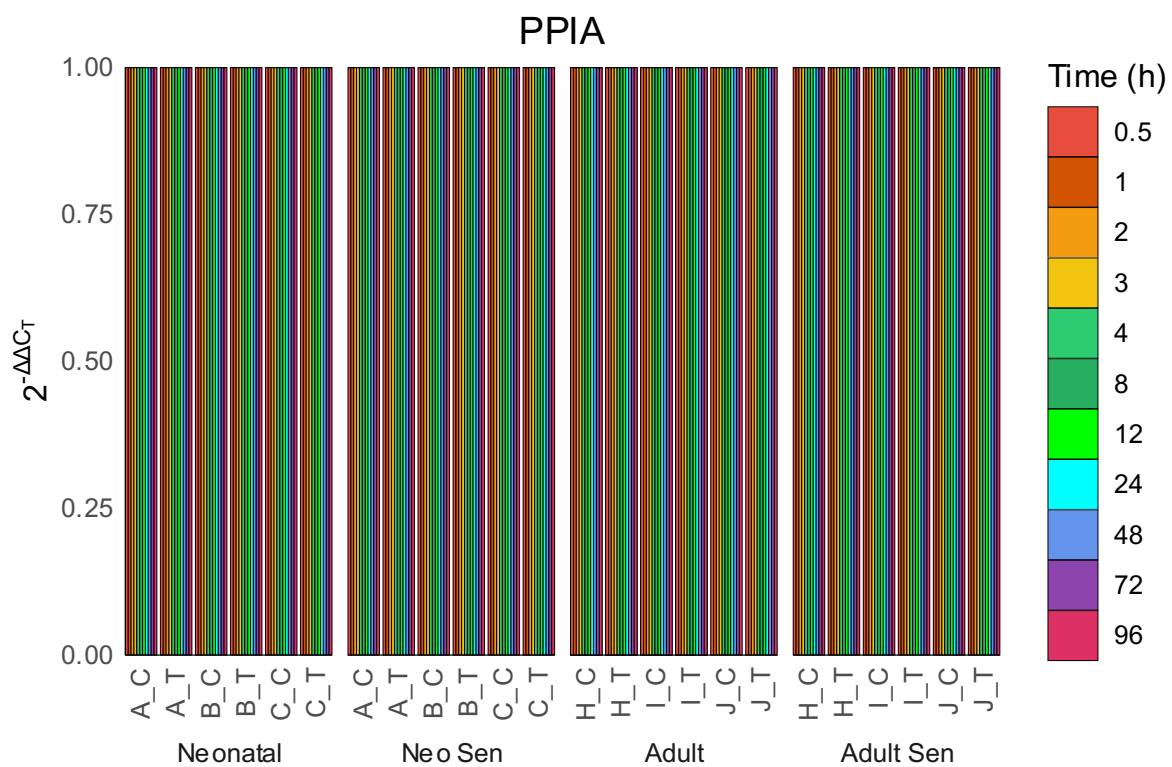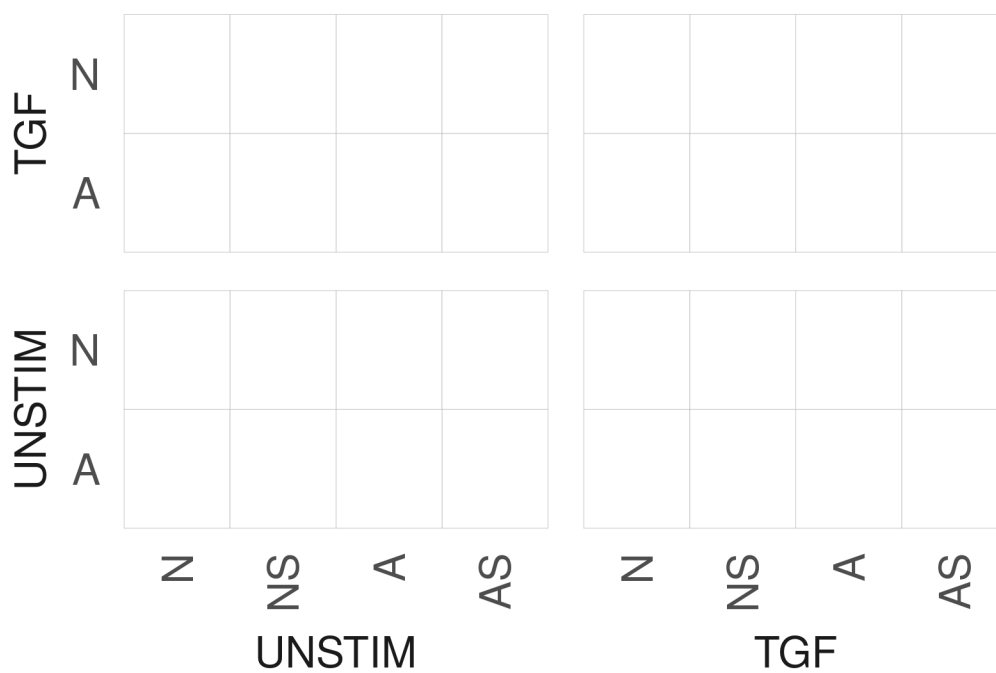

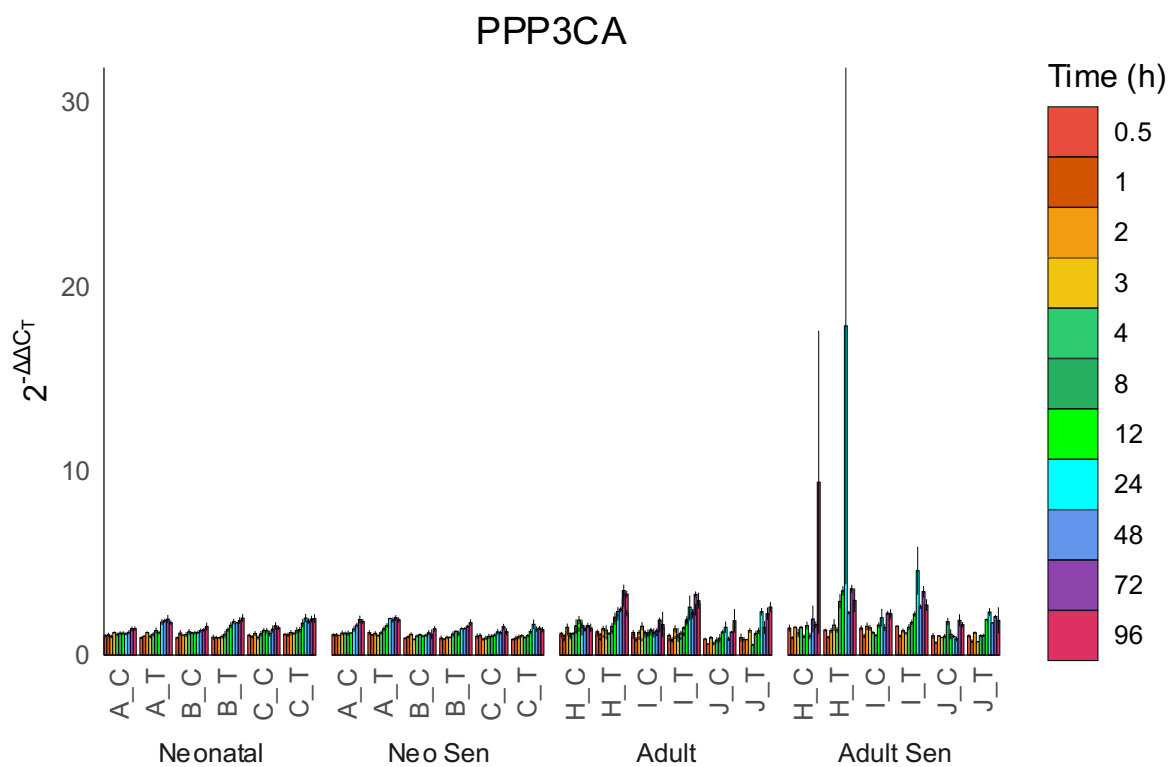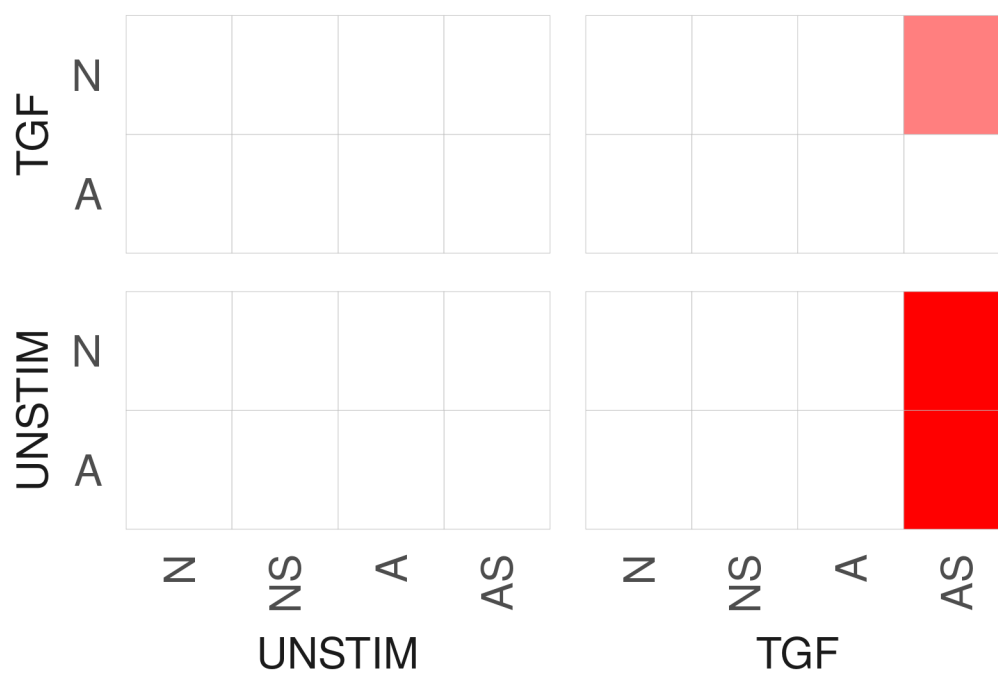

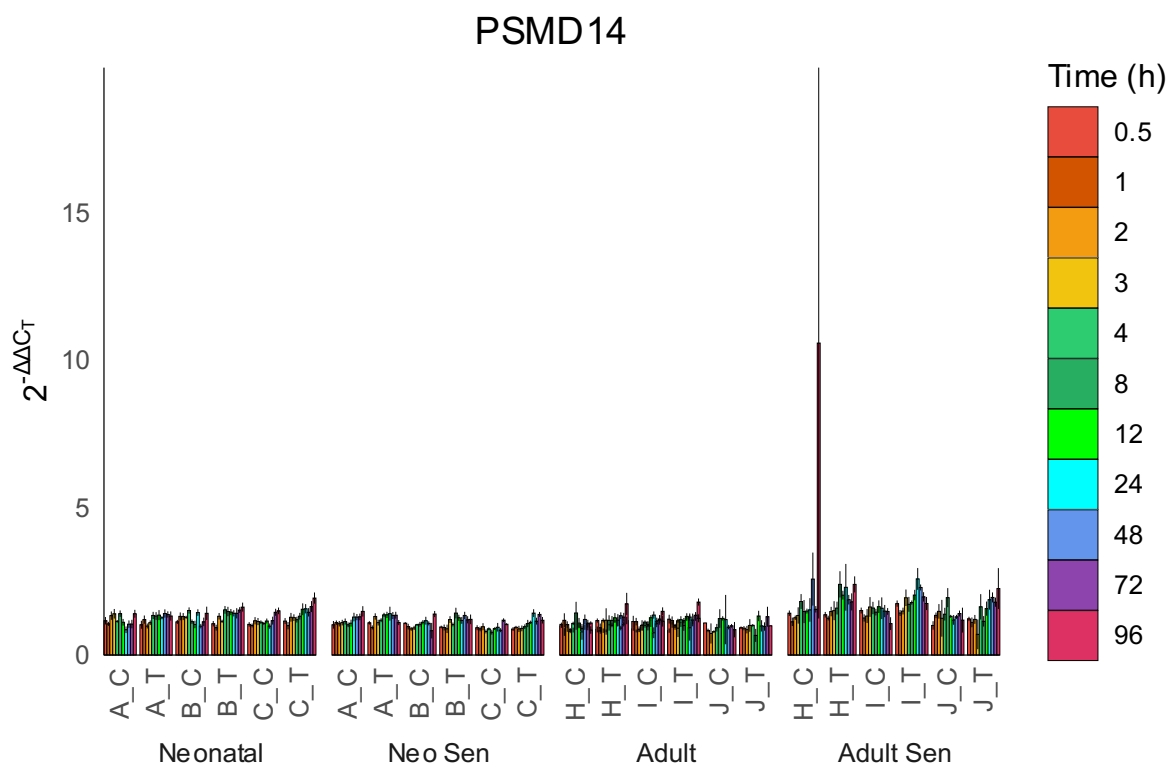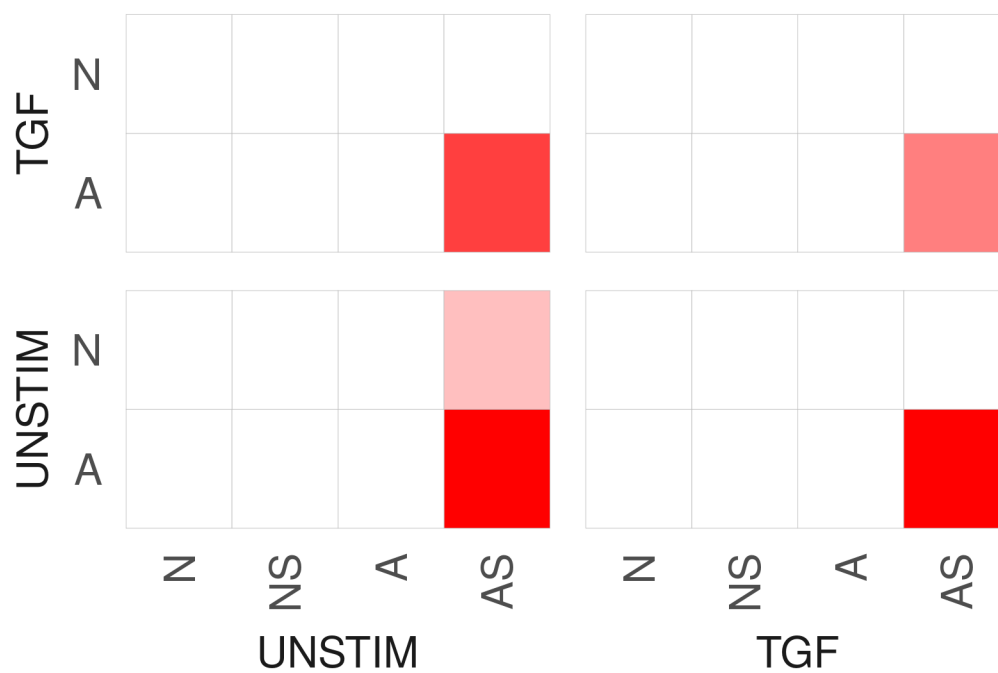

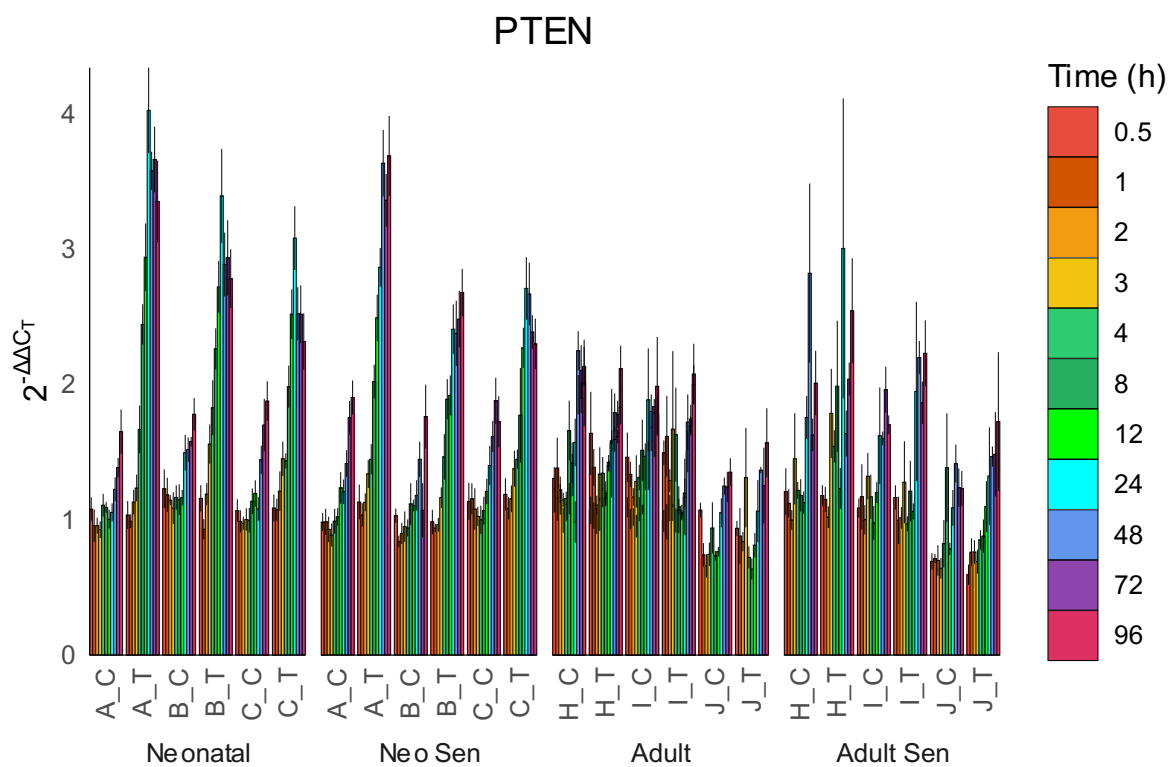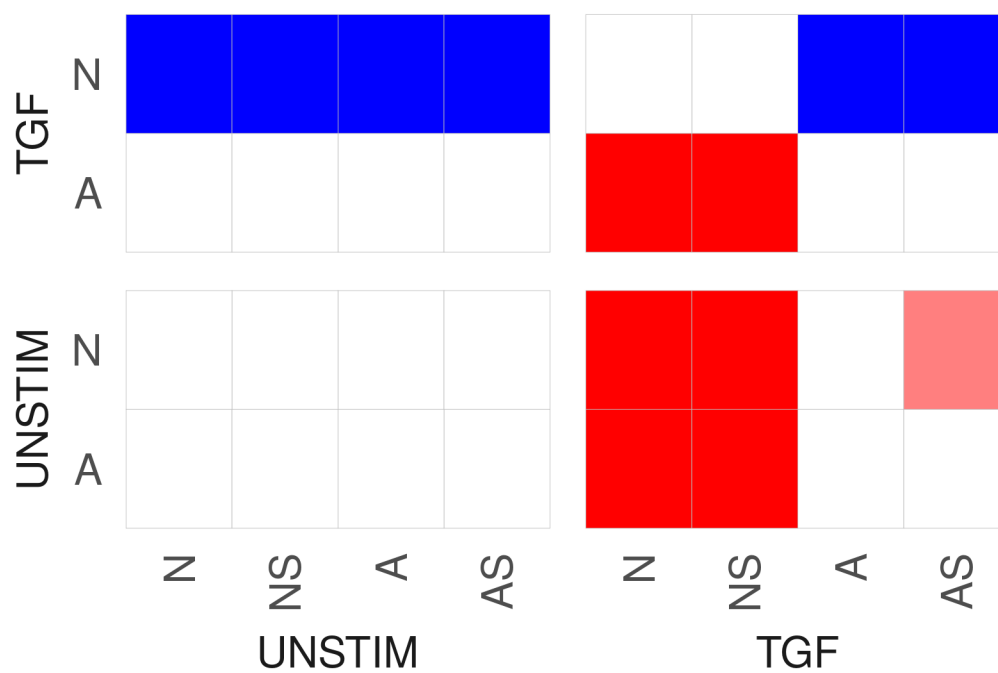

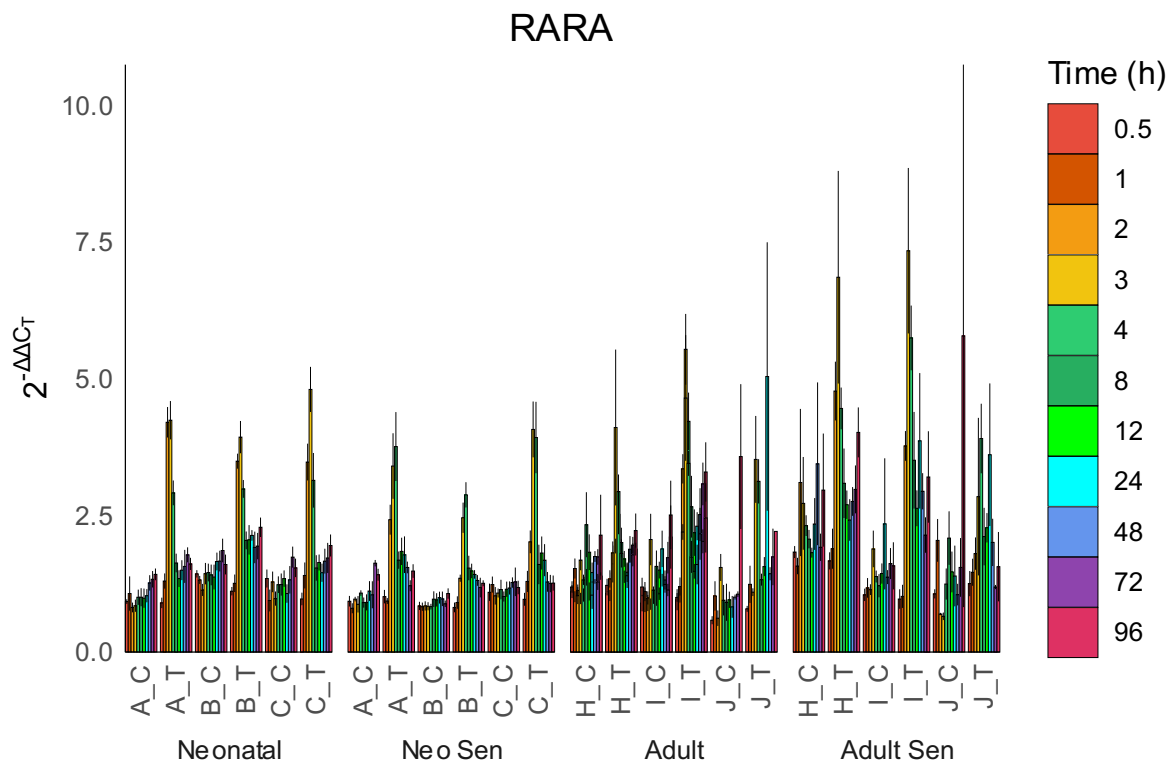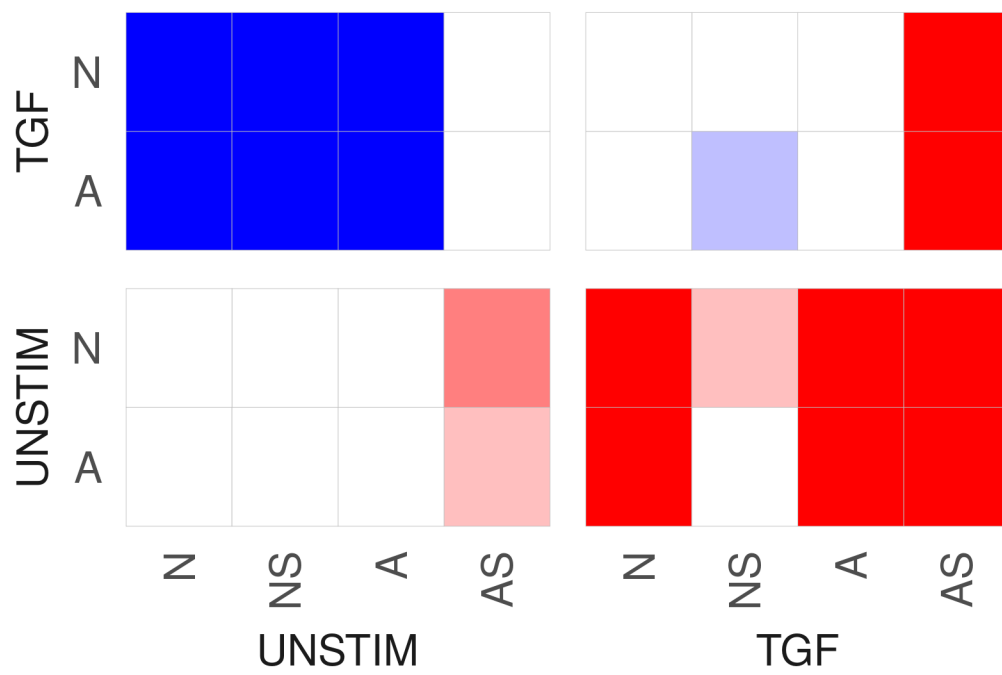

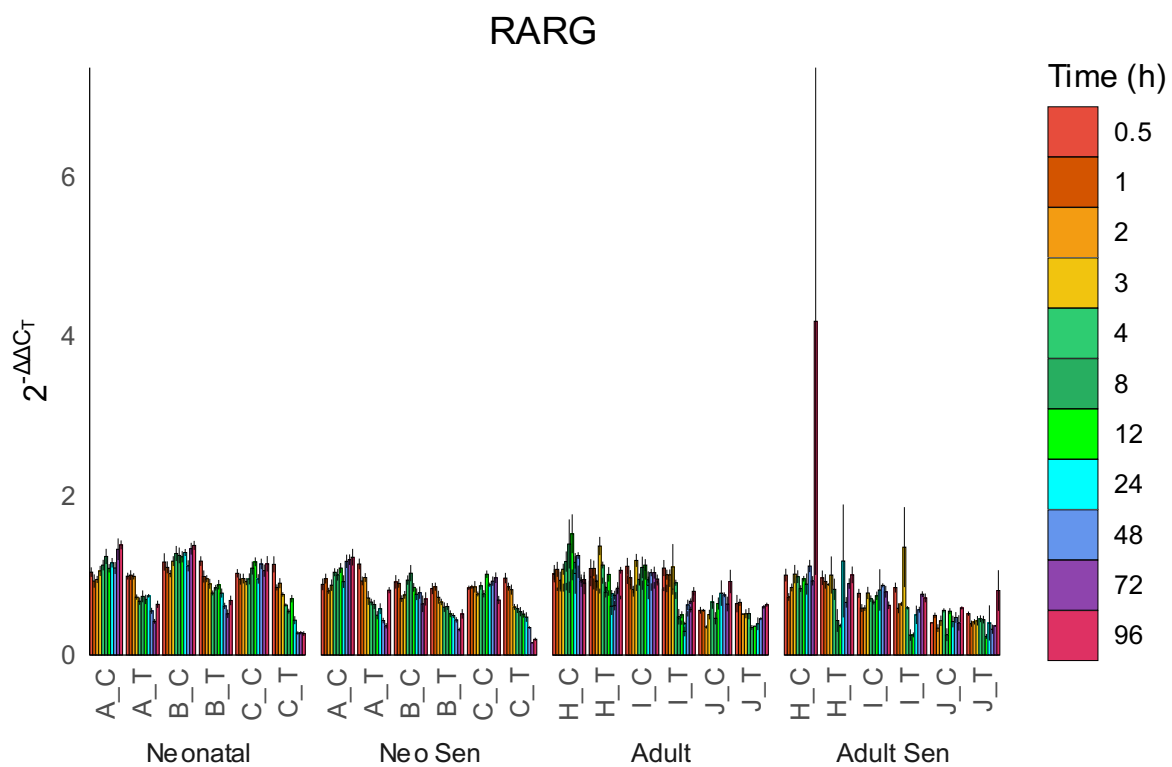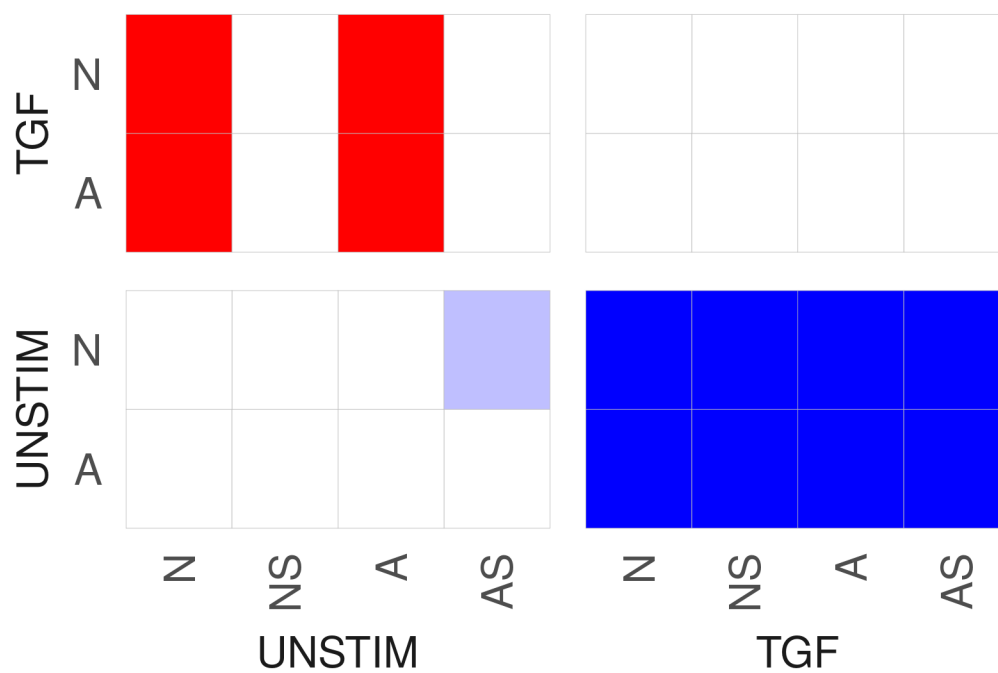

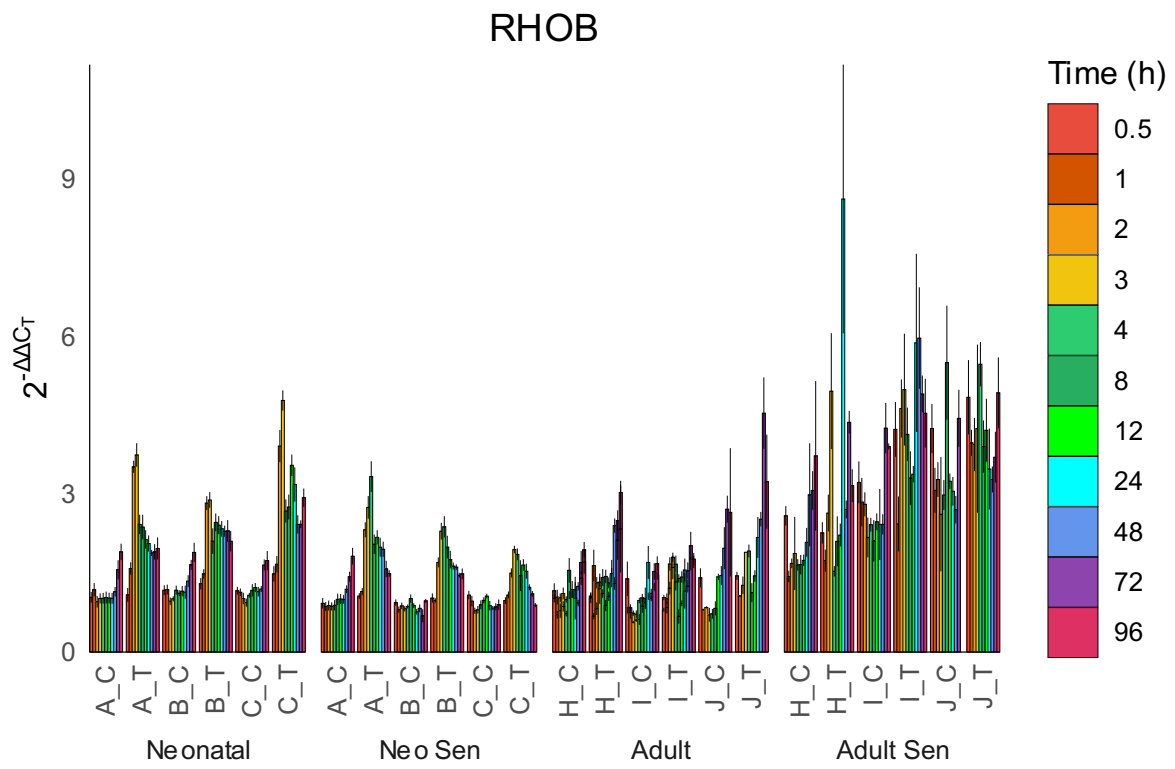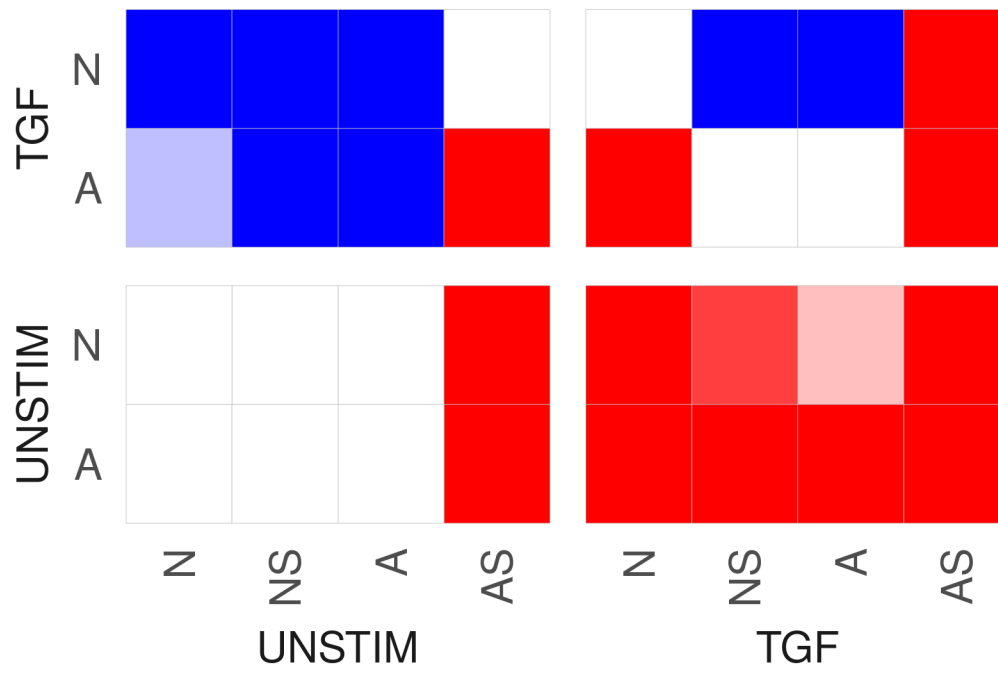

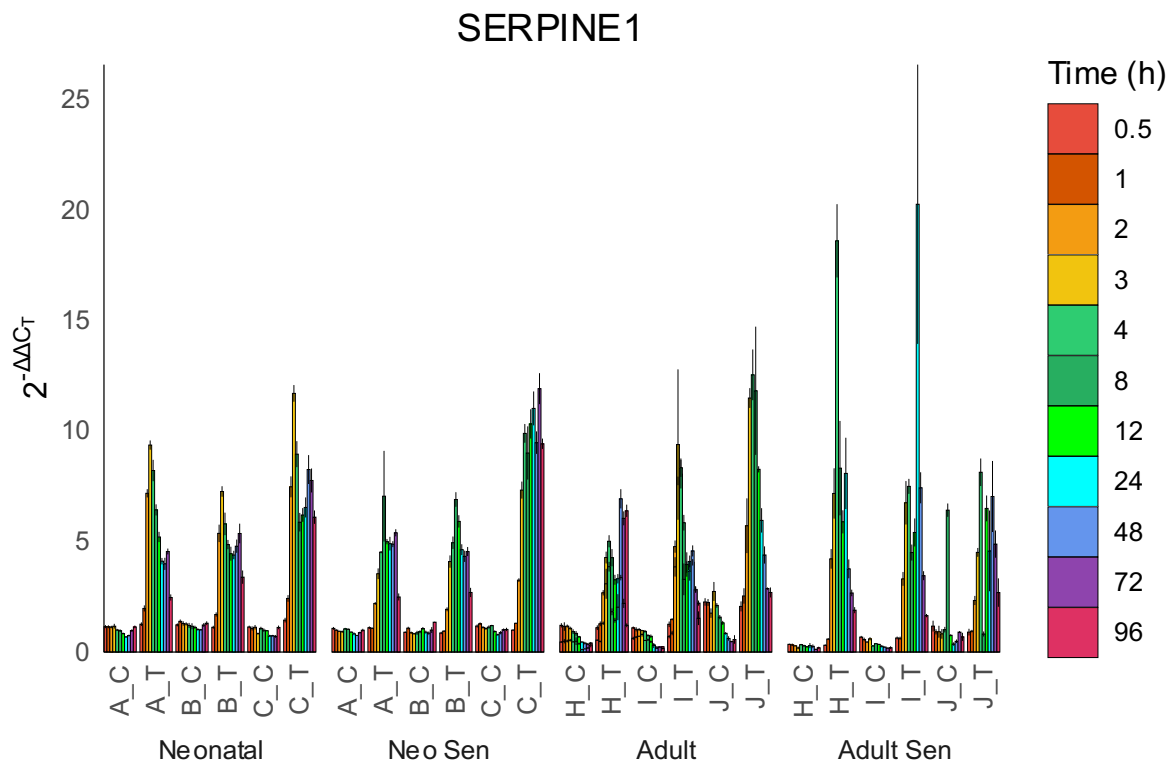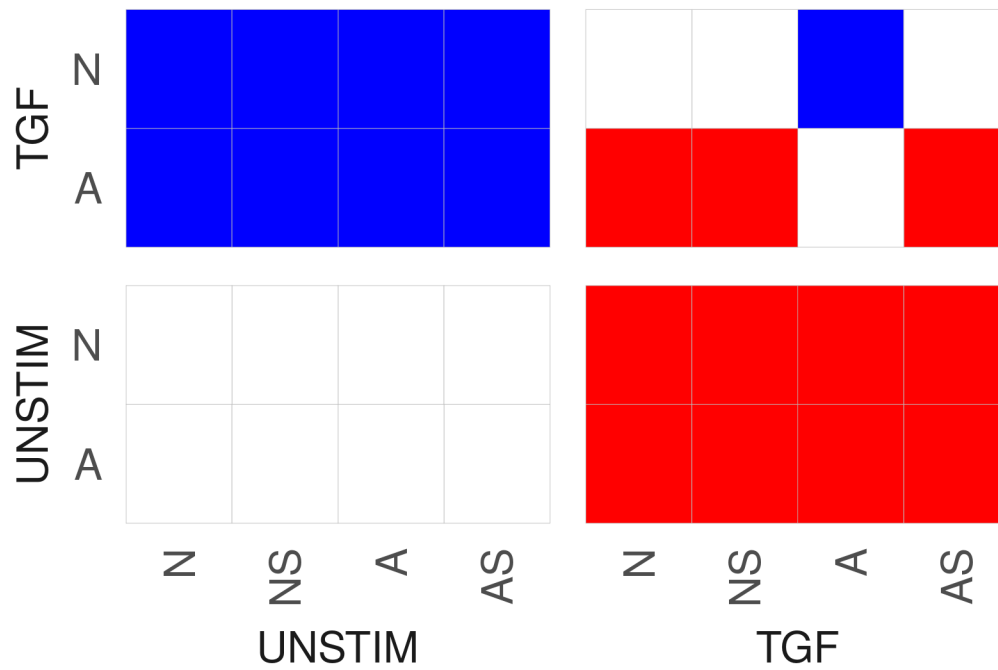

# SERPINE2

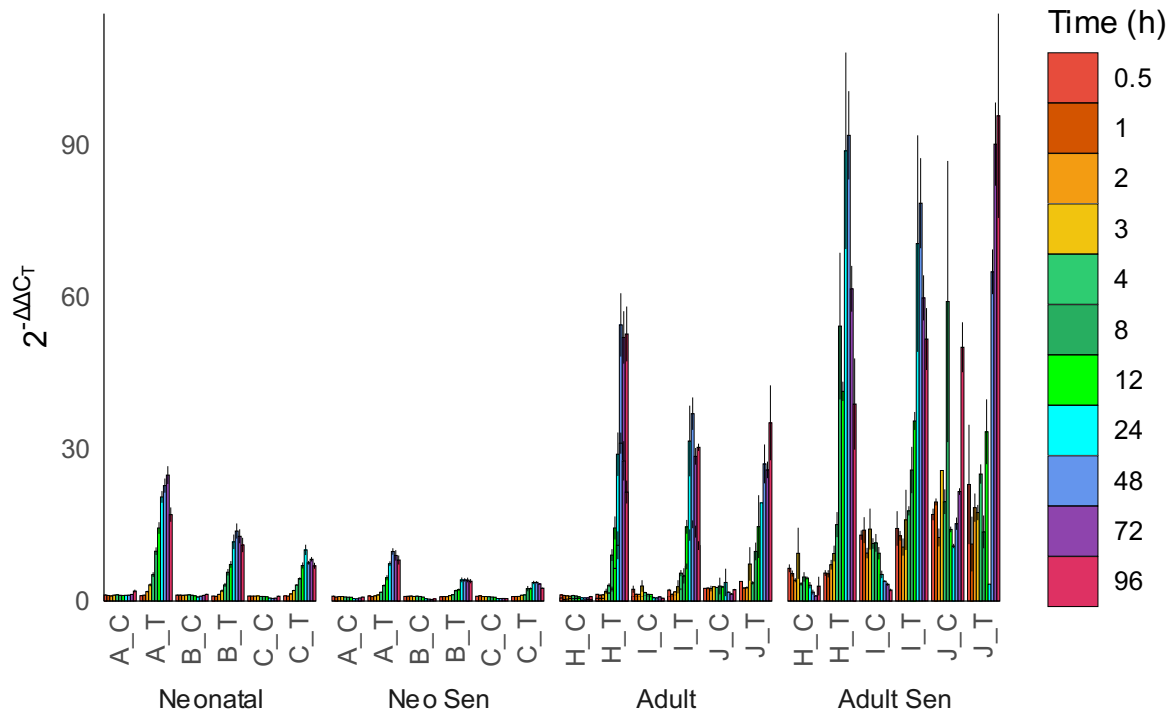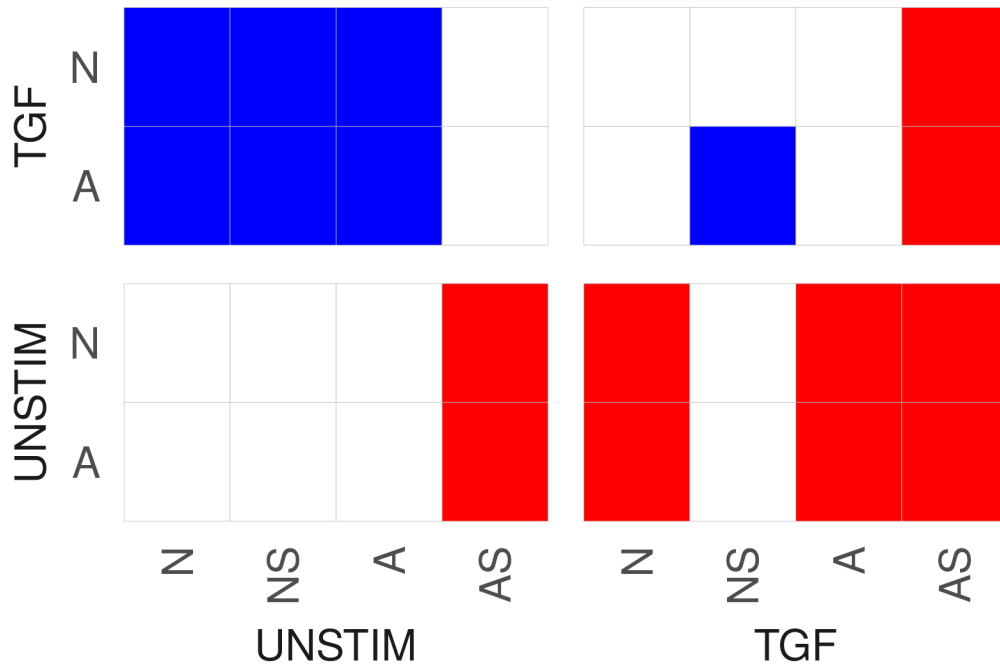

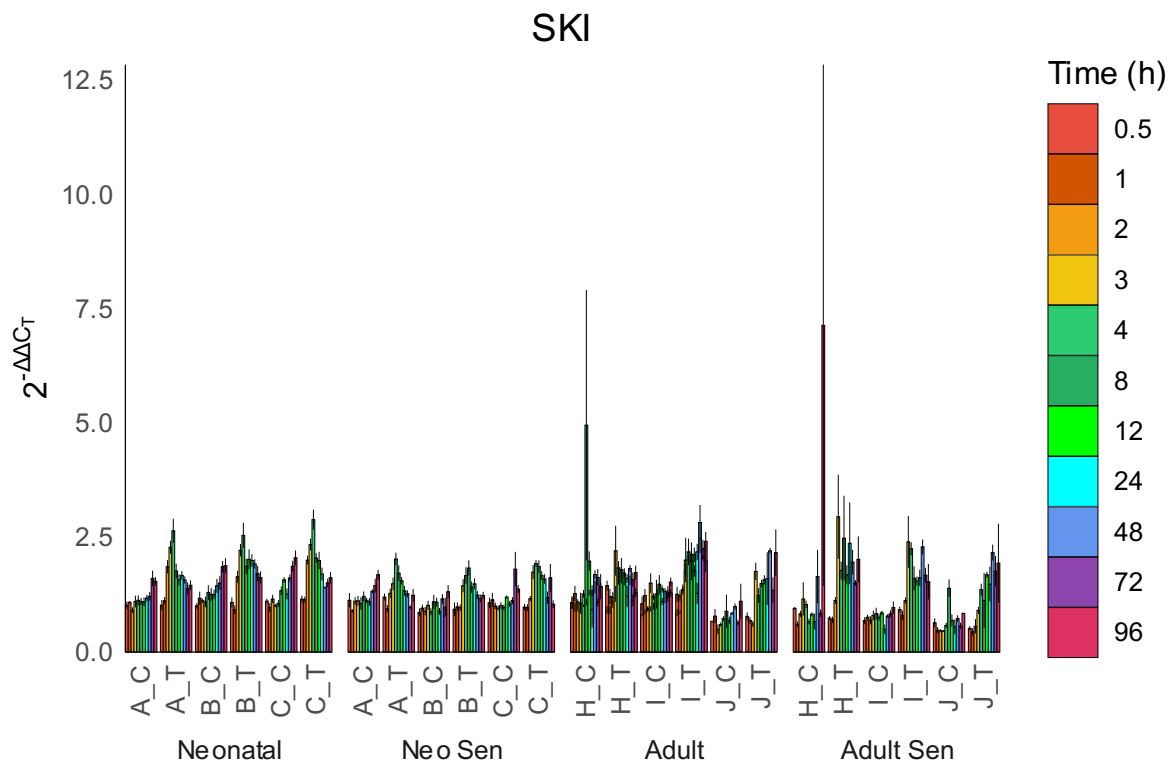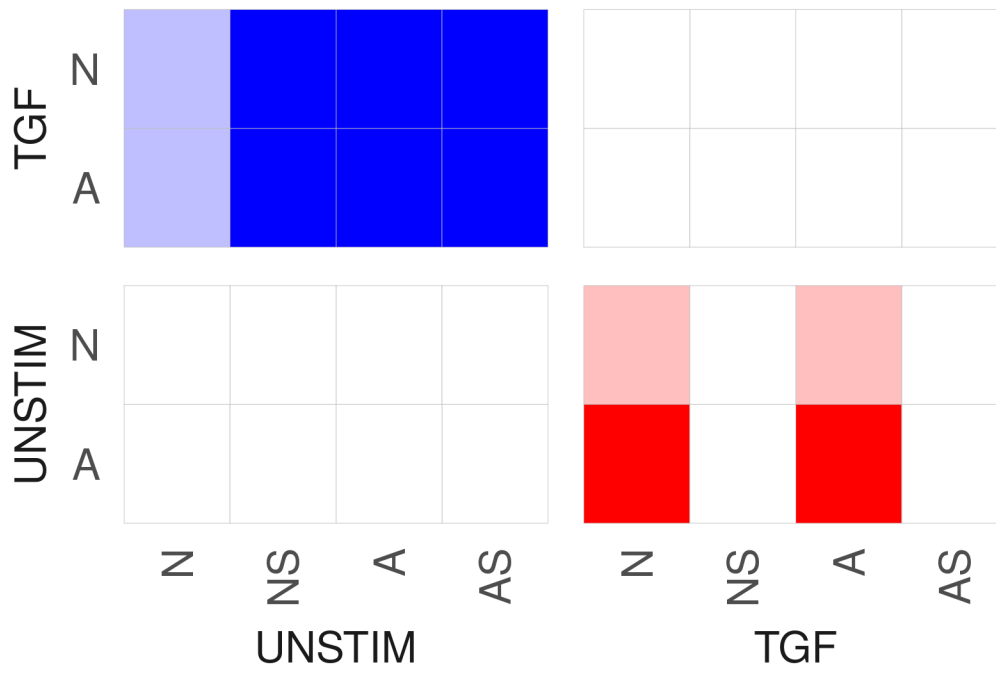

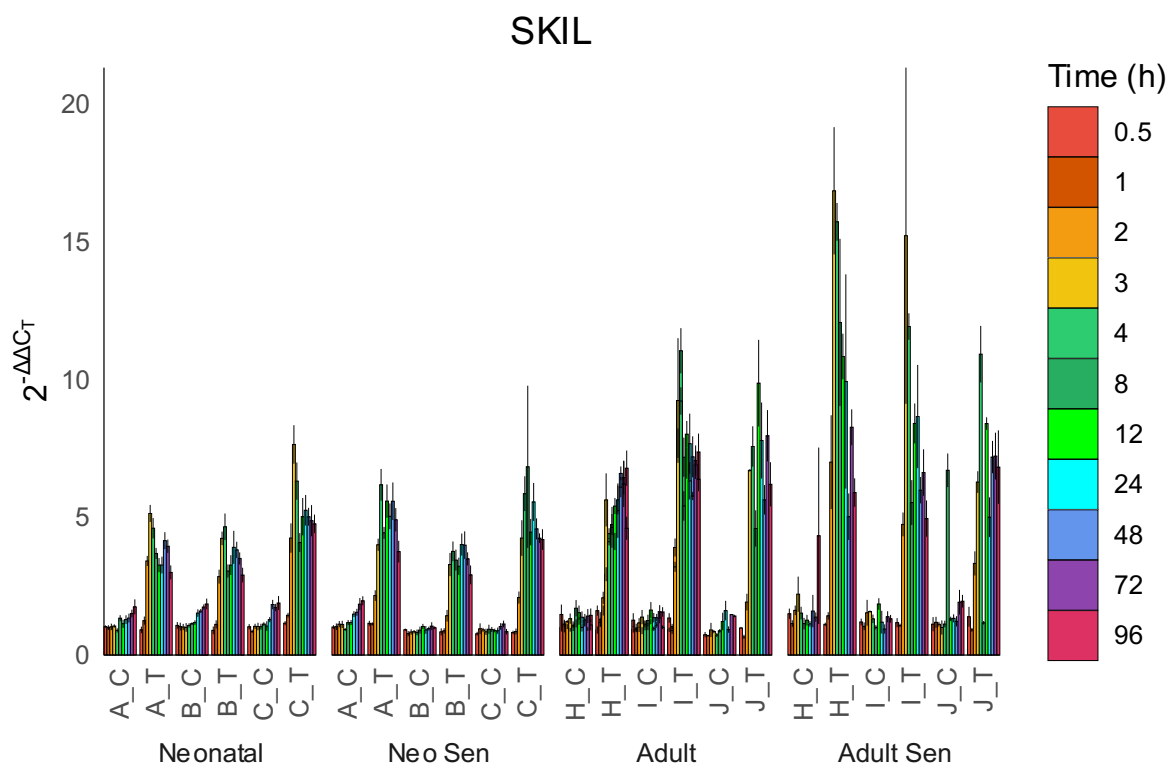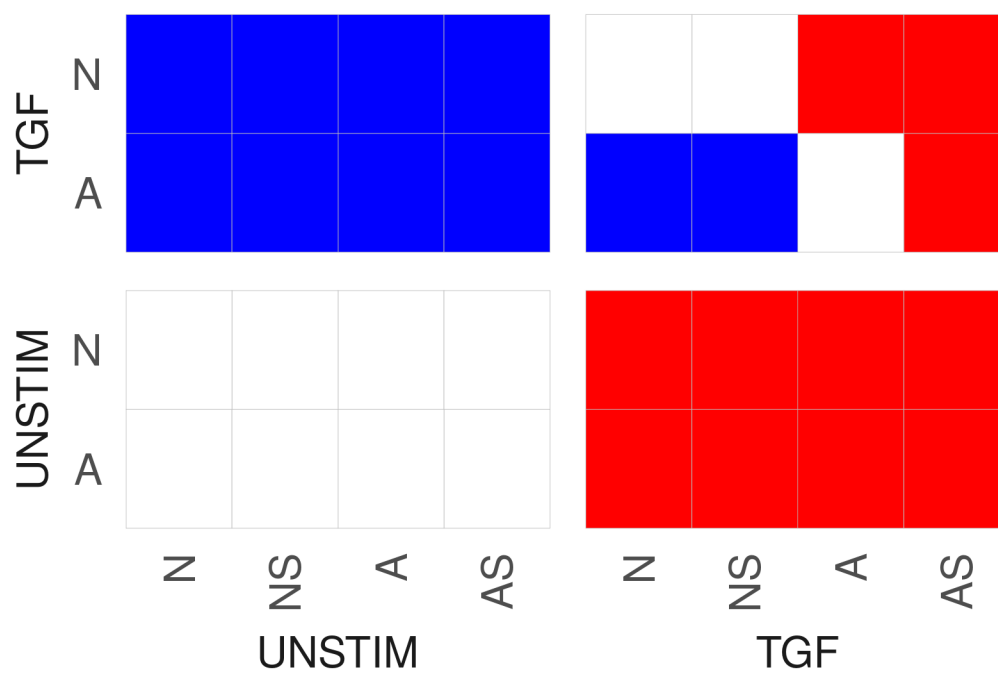

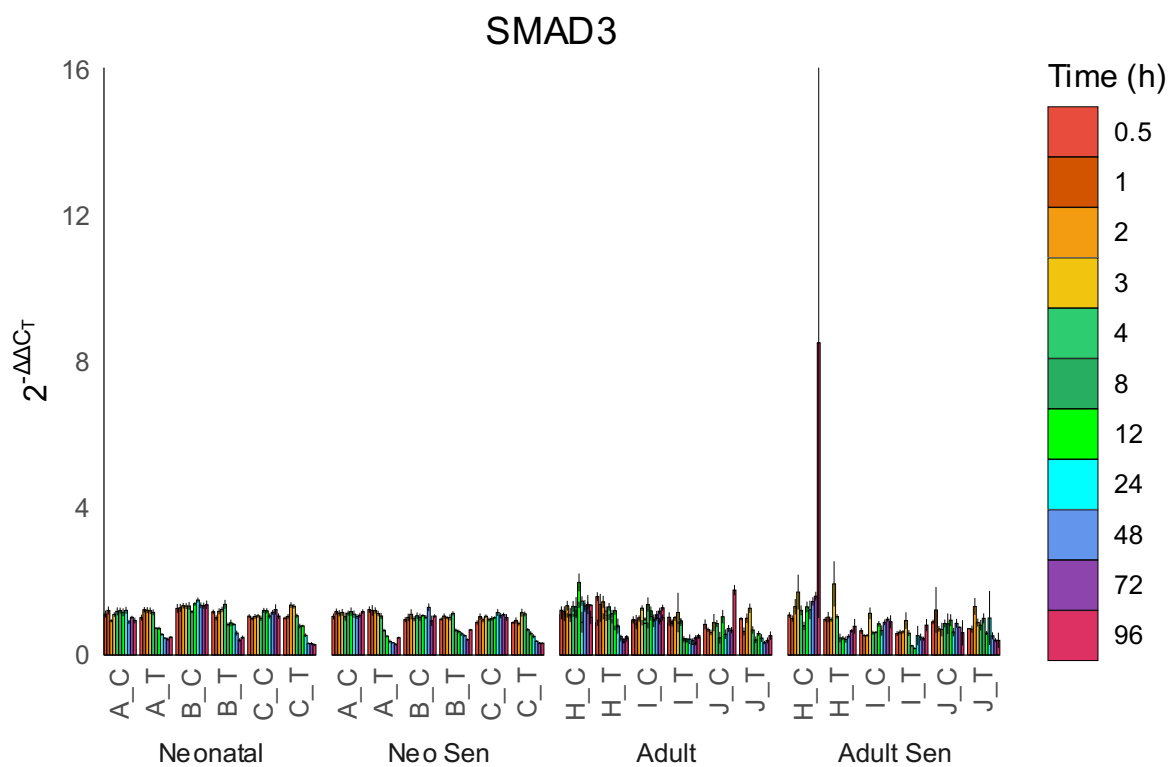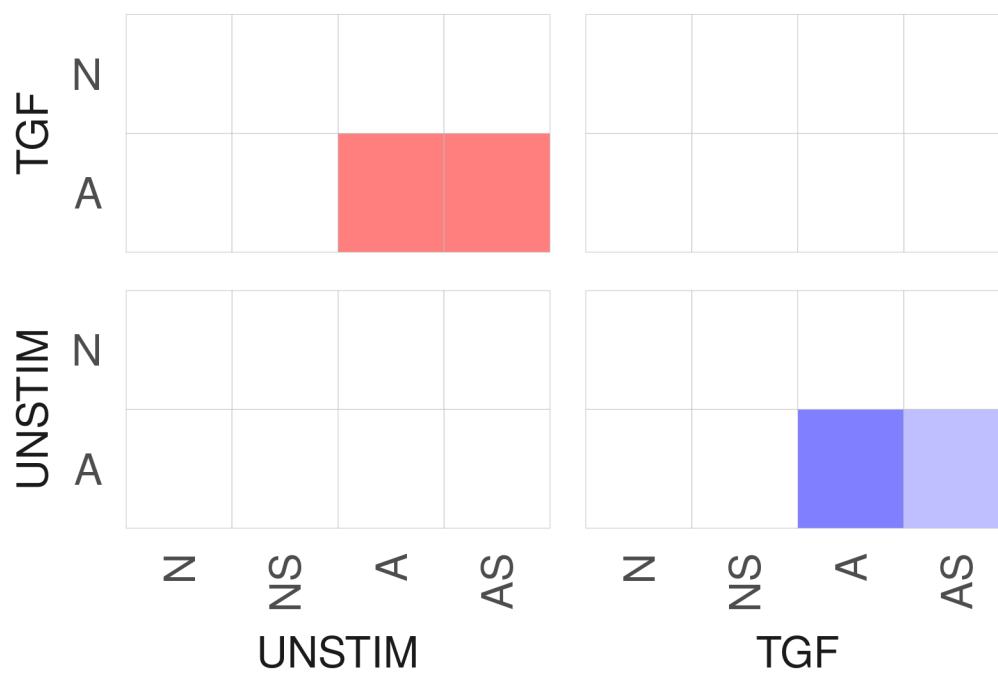

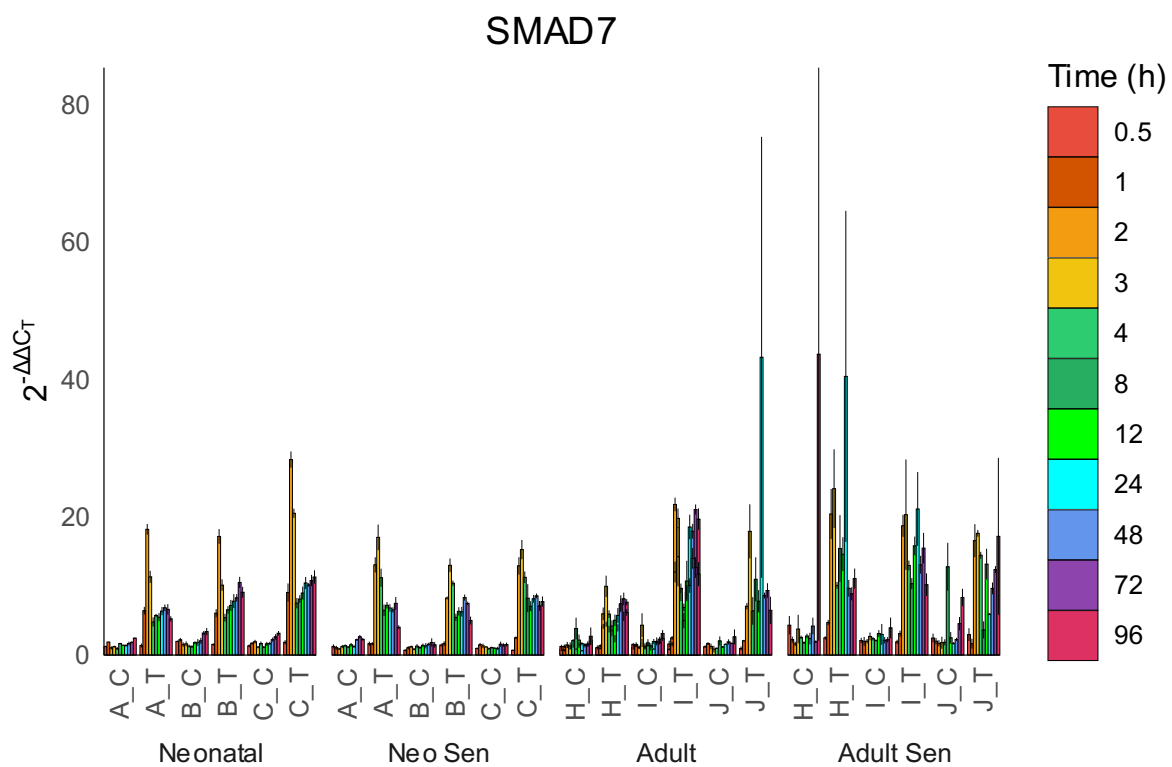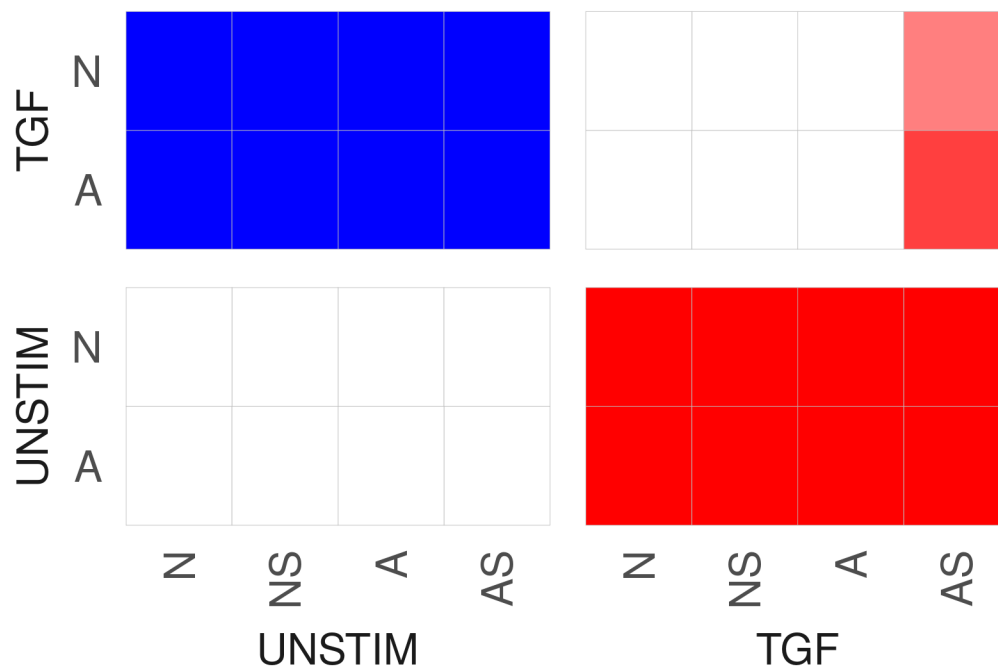

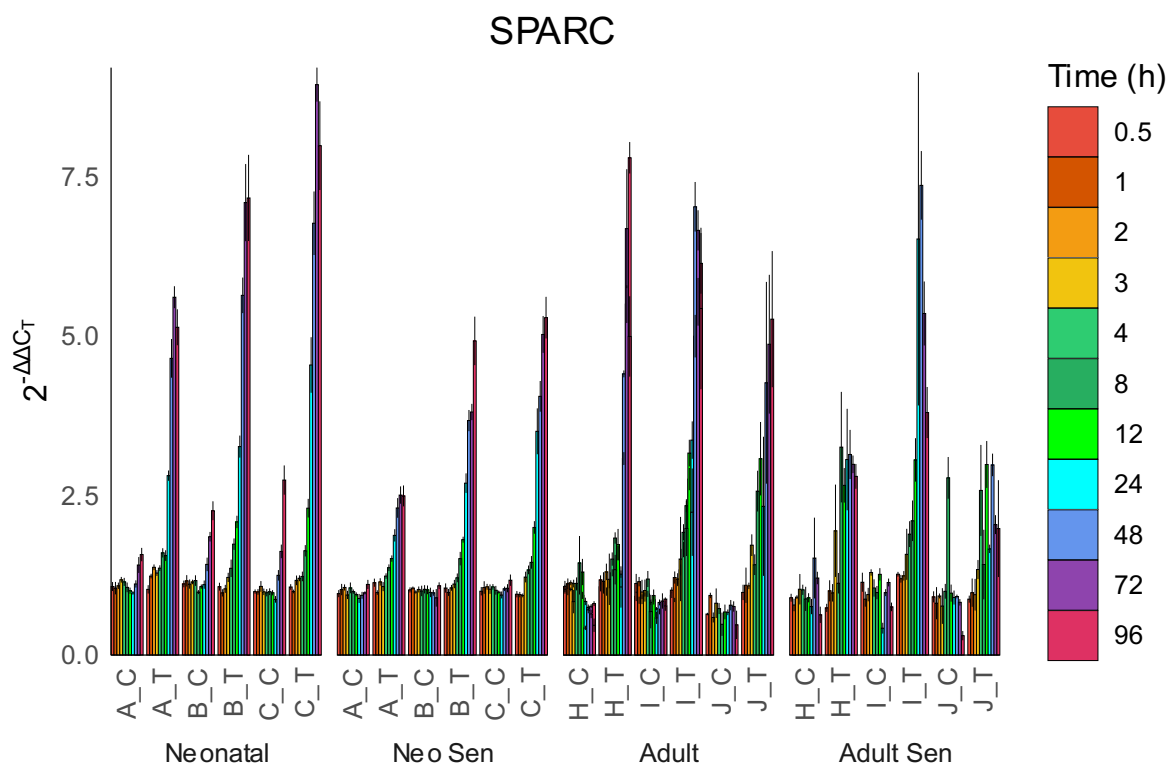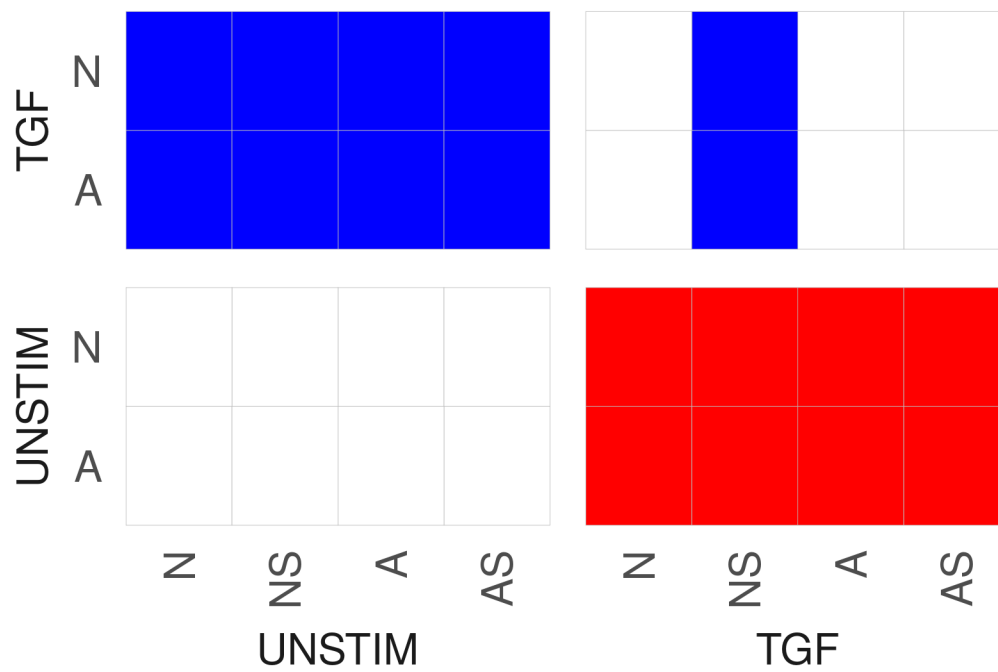

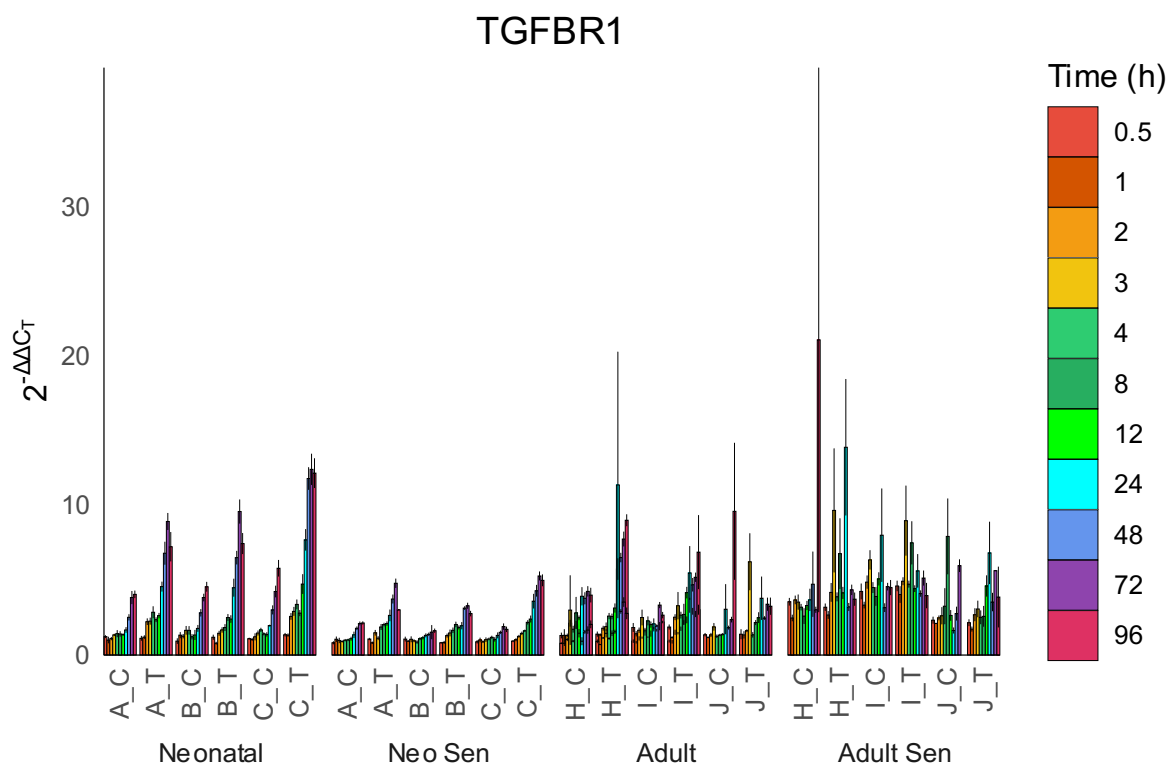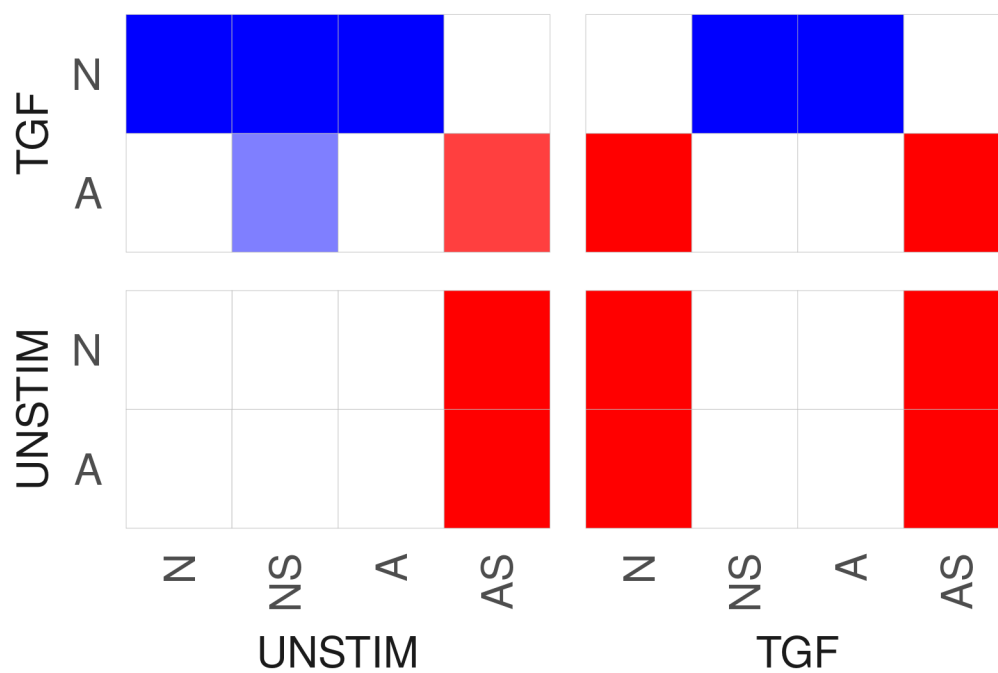

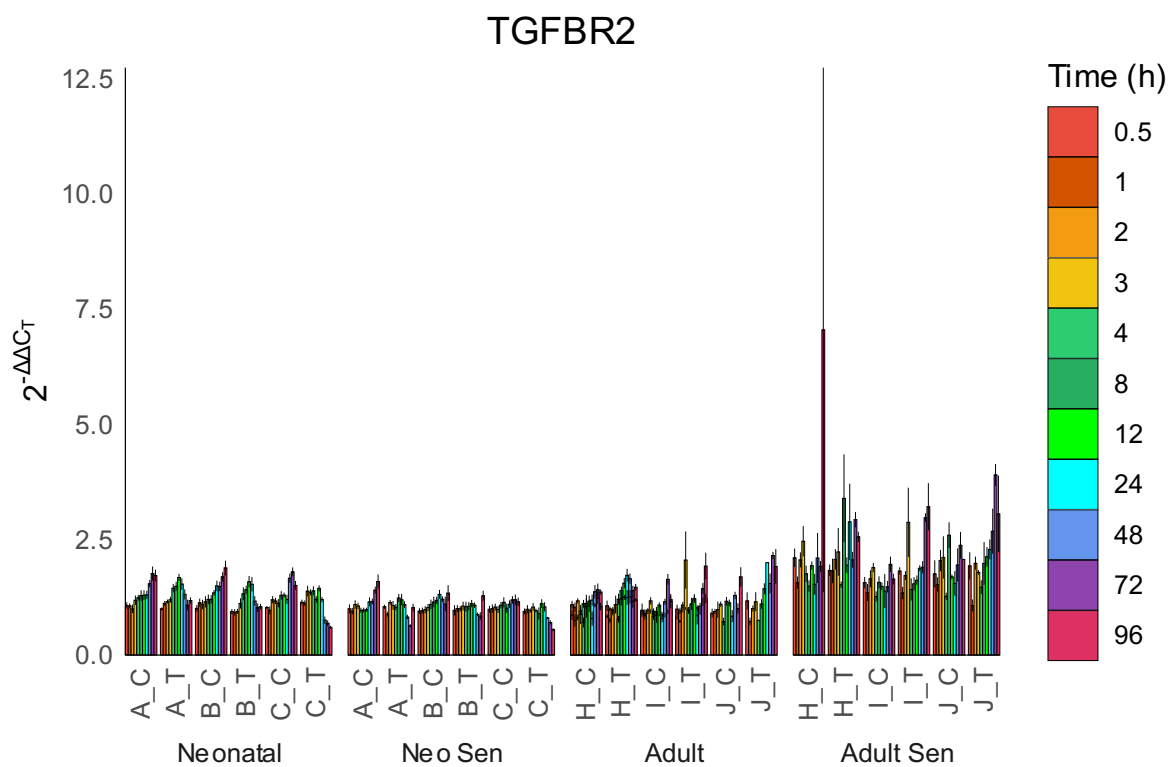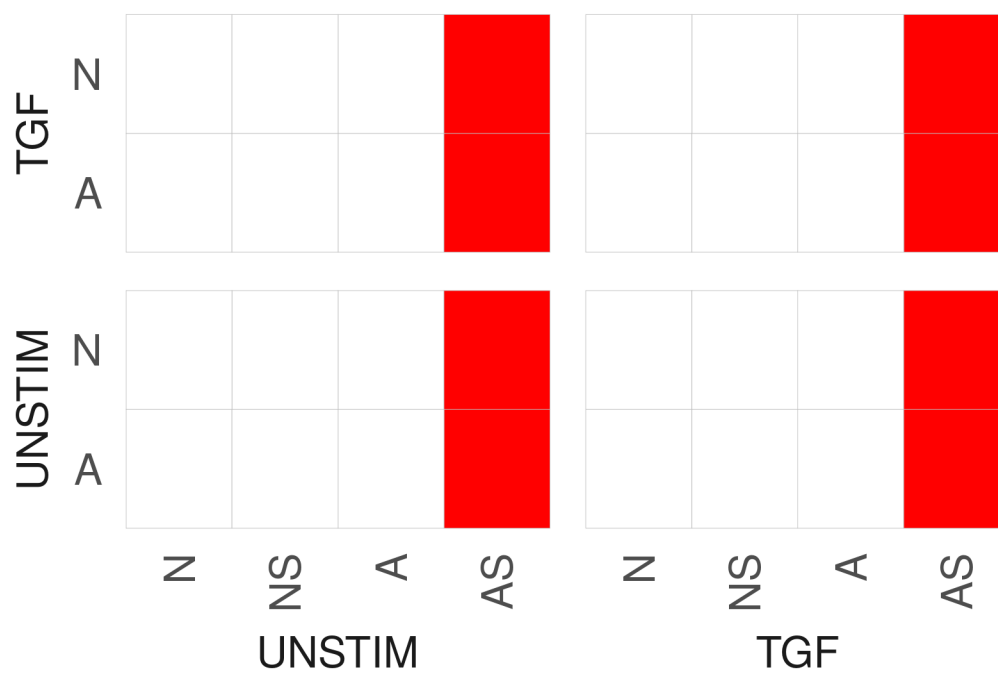

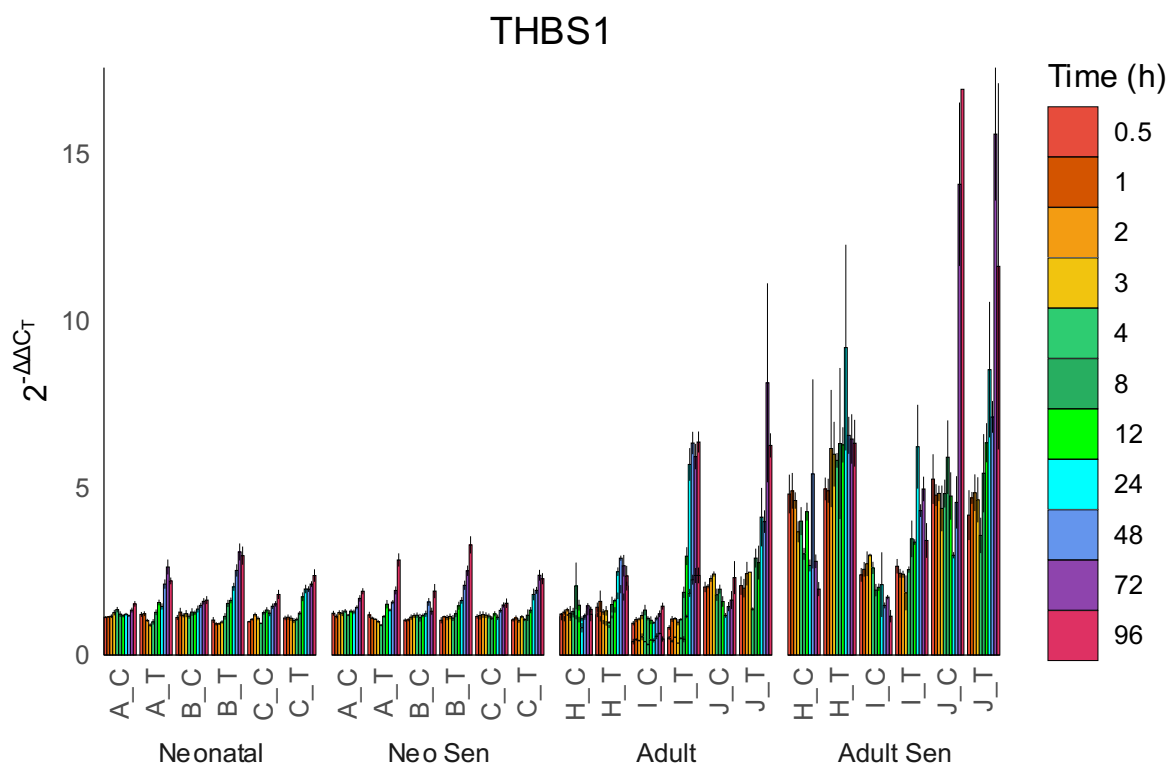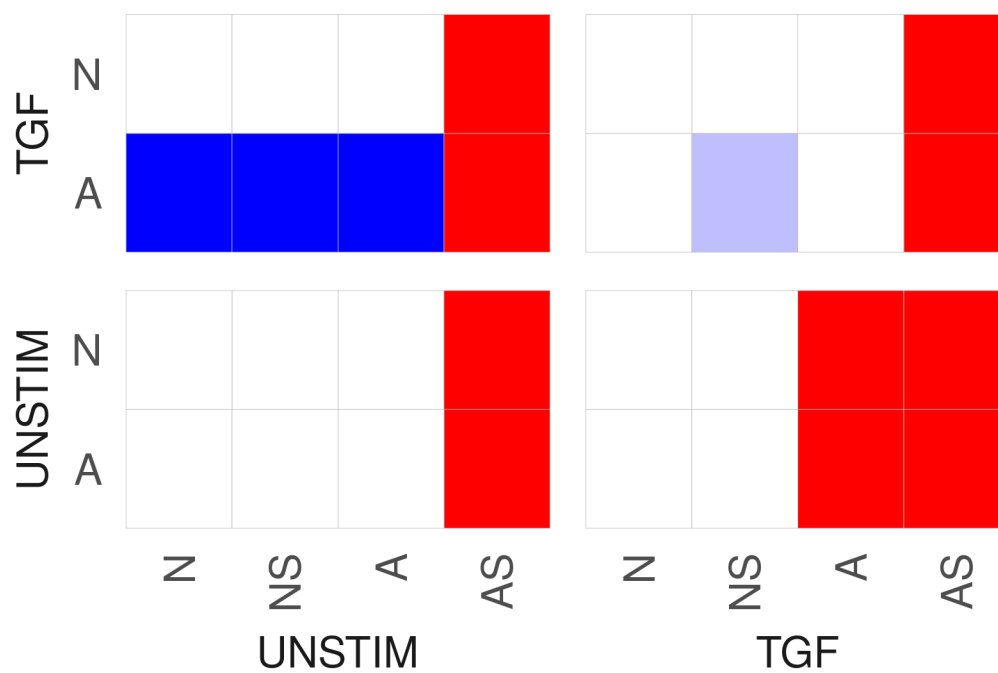

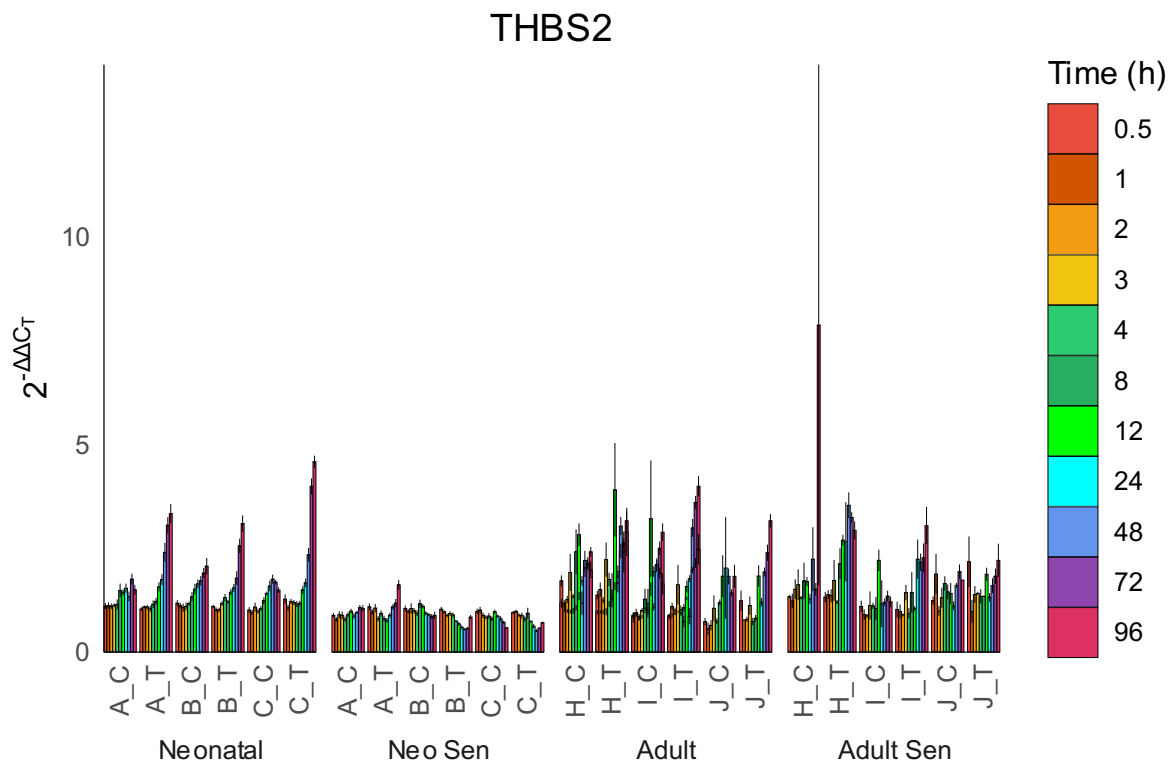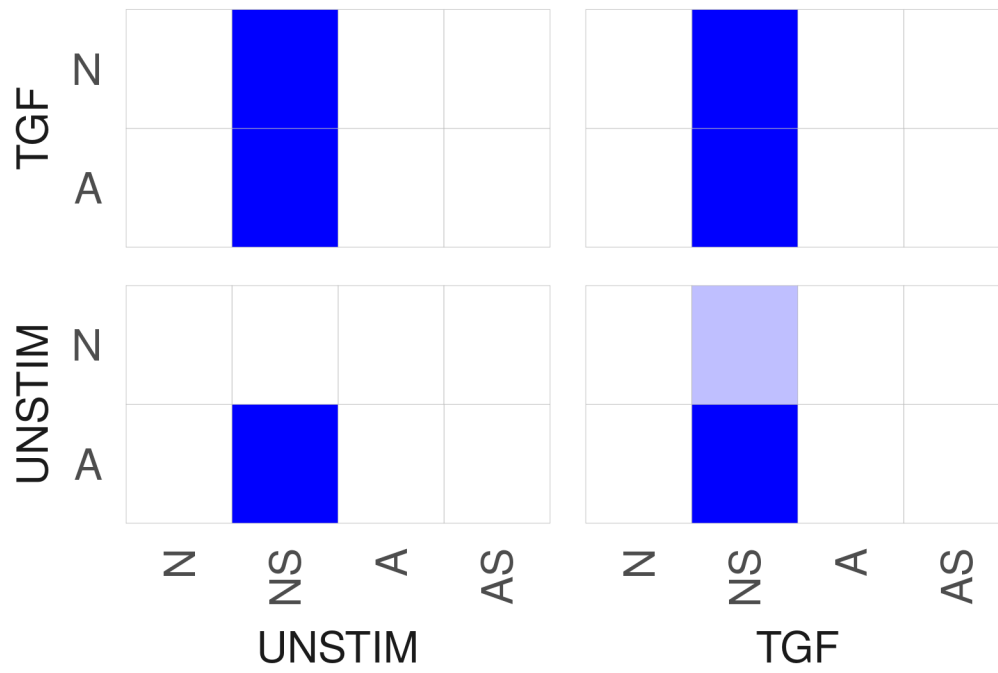

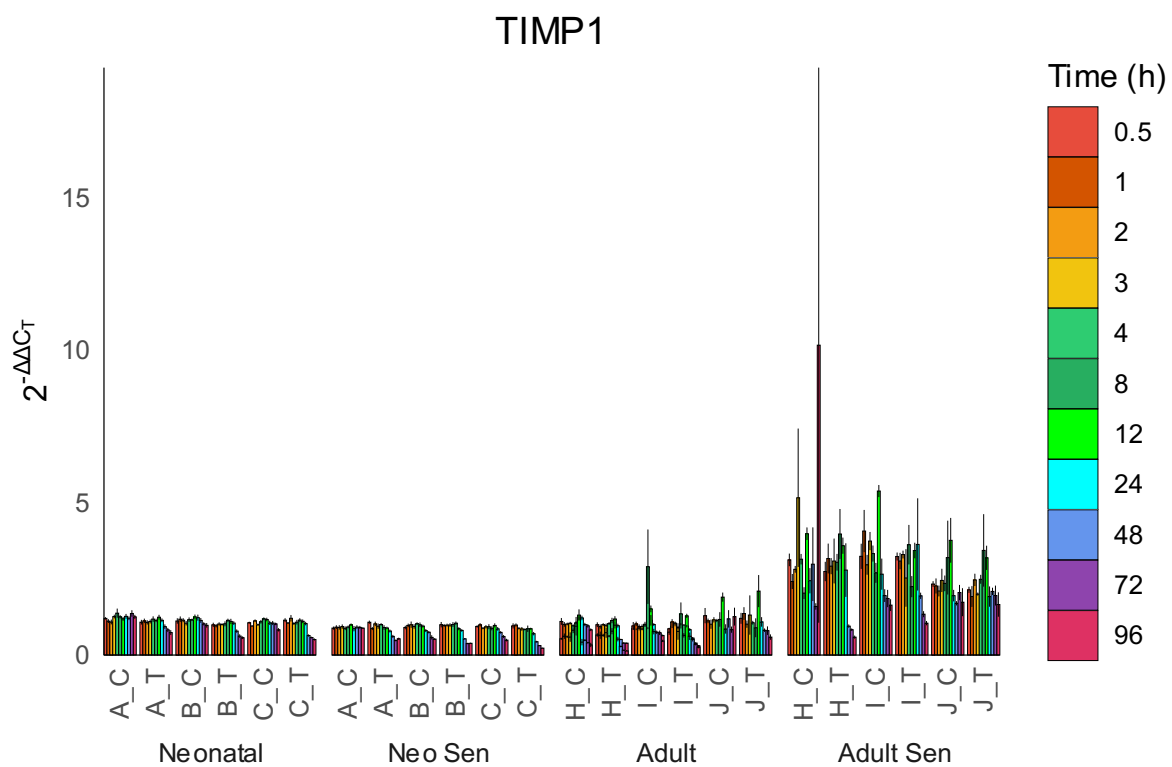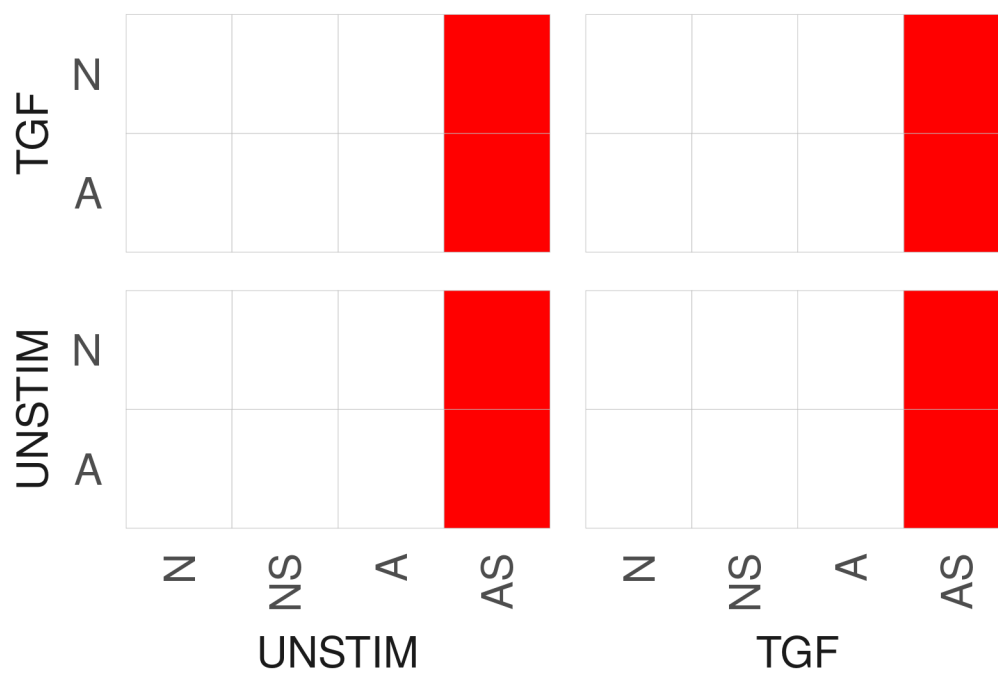

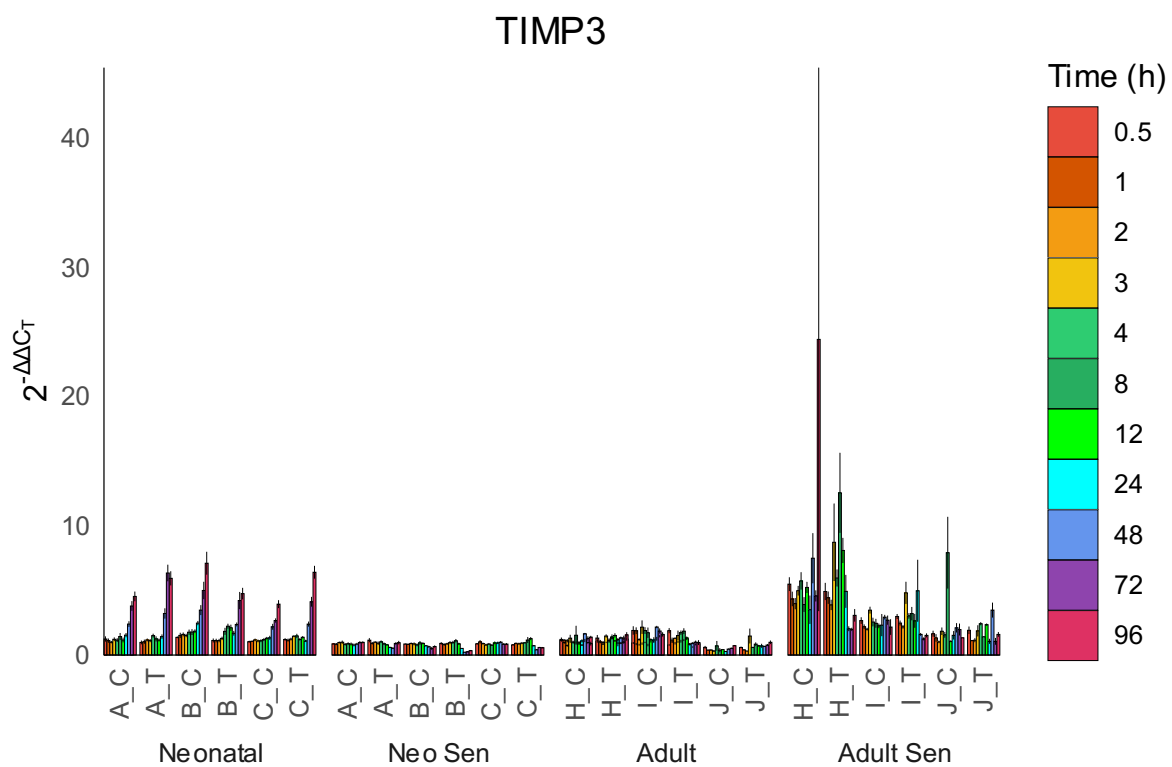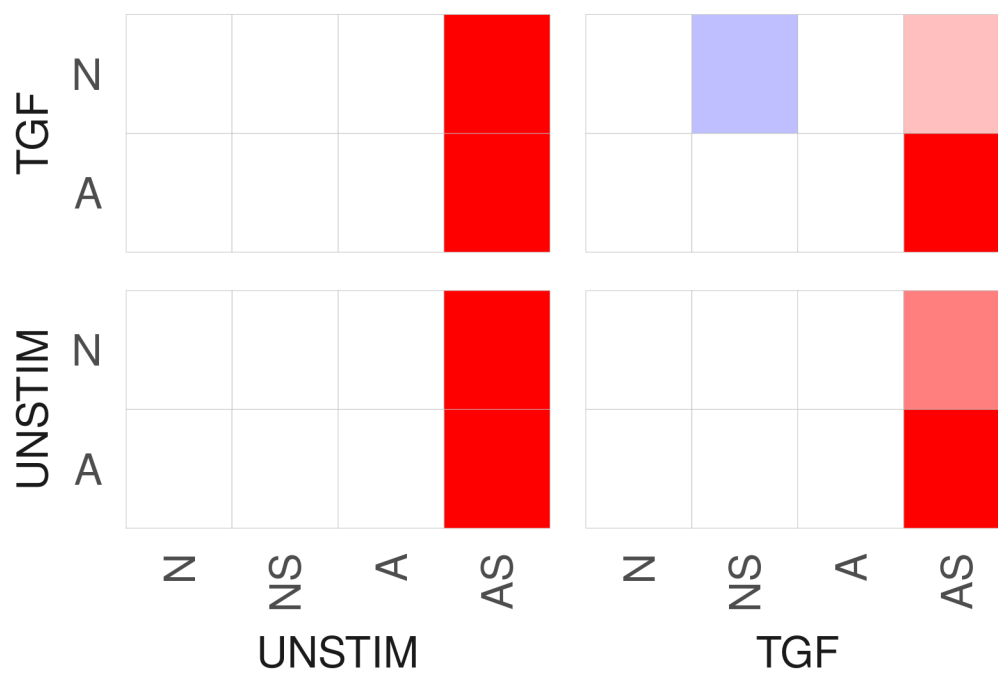

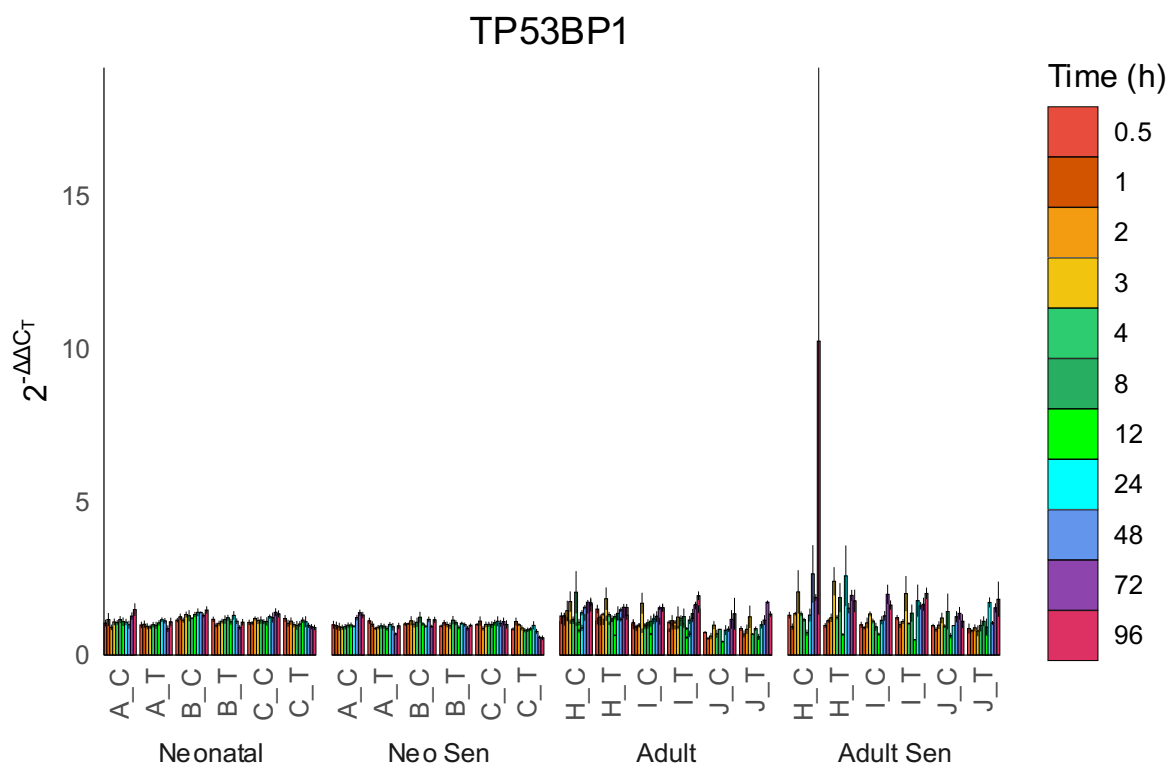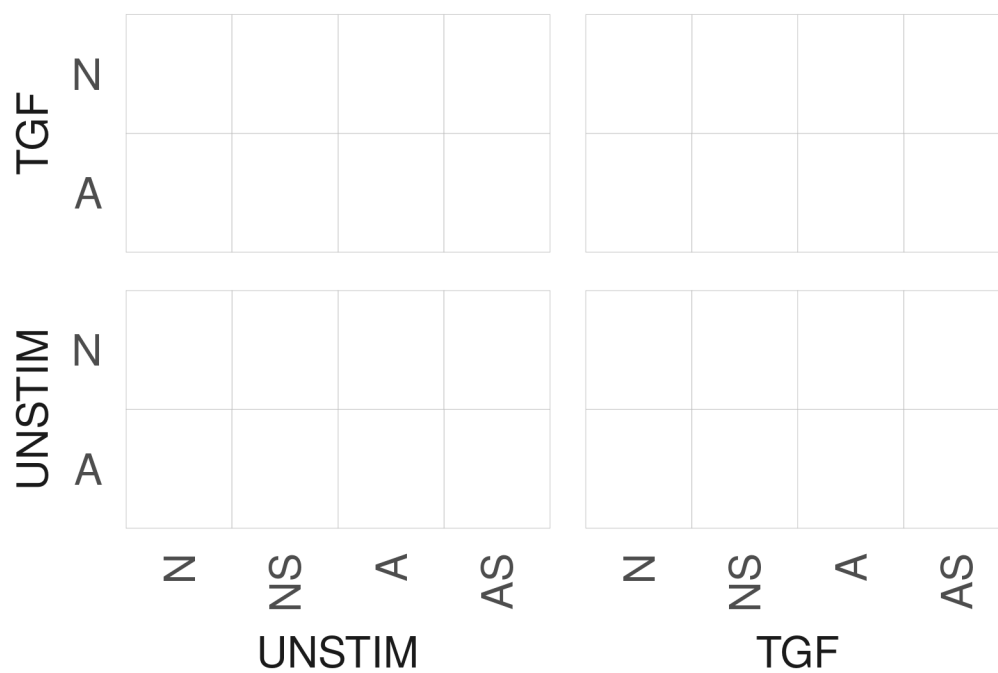

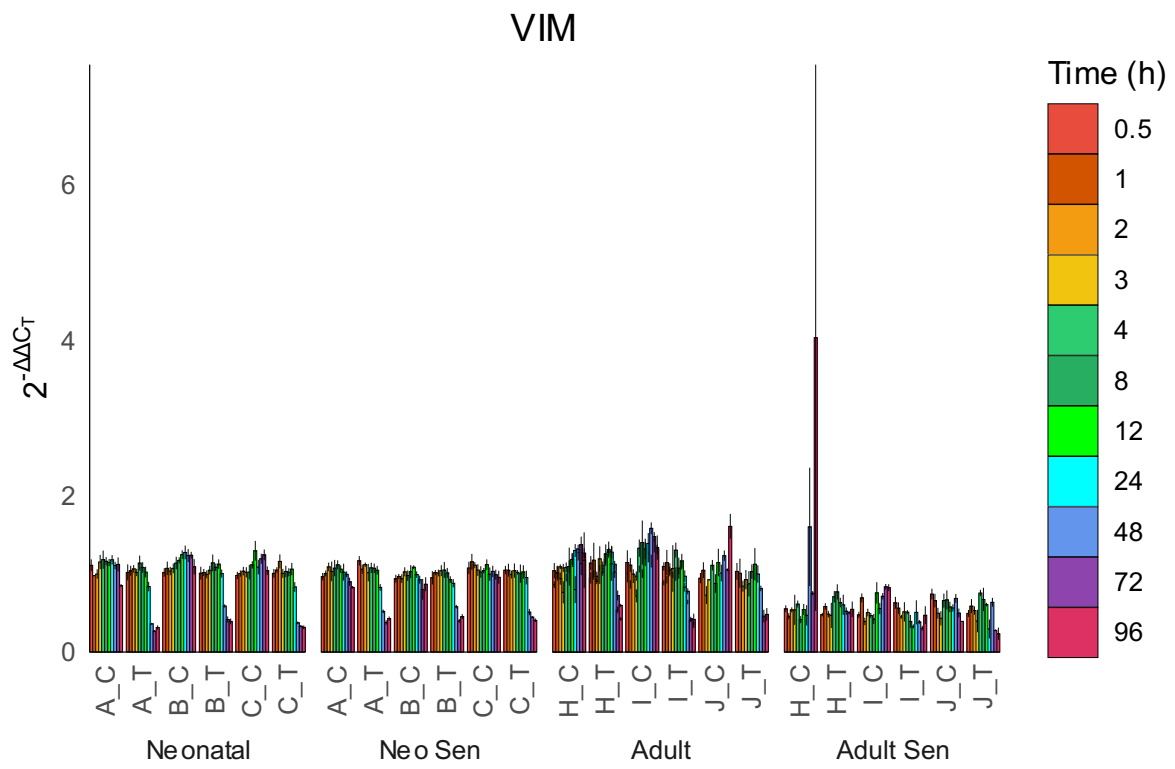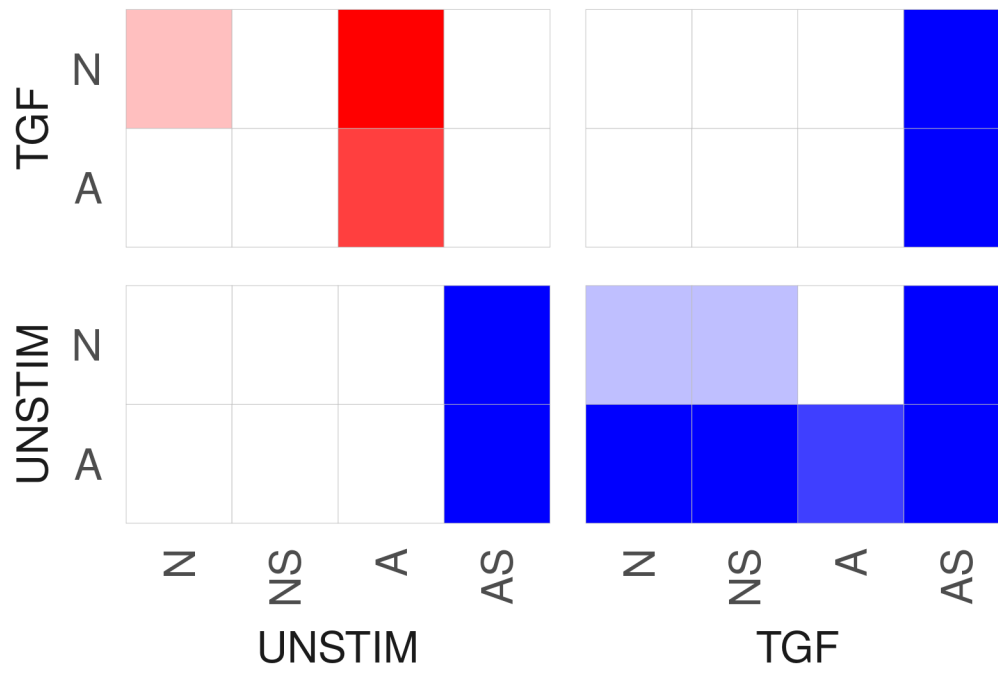

Supplement: Supplementary file 1 [file cells-13-00659-s001.zip › Supplementary file 6.pdf]
